# Understanding Stakeholder Perspectives on the Implementation and Management of Riparian Buffer Zones in the Santa Lucía River Basin, Uruguay

**DOI:** 10.1007/s00267-025-02230-1

**Published:** 2025-07-26

**Authors:** Alfred Paarlberg, Guillermo Sena, Ho Huu Loc, Jannik Schultner

**Affiliations:** 1 Wageningen University, Lumen, Wageningen, The Netherlands; 2 Wageningen University, Leeuwenborch, Wageningen, The Netherlands

**Keywords:** Riparian buffer zones, Ecosystem services, Stakeholder perspectives, Policy design, Santa Lucía River Basin, Nature-based solutions

## Abstract

Riparian buffer zones are essential nature-based solutions for protecting freshwater ecosystems globally, yet their implementation faces challenges in balancing ecological, agricultural, and social needs. In the Santa Lucía River Basin (SLRB) in Uruguay, these buffers are critical for improving water quality but face issues like low compliance and limited awareness of the policy in place. We explored stakeholder perspectives on riparian buffer implementation through 24 semi-structured interviews with government institutions, researchers, producer unions, producers, NGOs, and locals. Our aim was to identify perceptions of current and desired ecosystem services, buffer characteristics, and barriers and opportunities to successful implementation. Our results show that stakeholders acknowledge key ecosystem services such as pollution retention and erosion reduction, but they desire additional services like enhanced agricultural productivity and recreational opportunities. Stakeholders identified native vegetation and the spatial dimensions of buffer zones as important physical characteristics of buffer zones. Preferred management practices included no-tillage and extensive agricultural management practices, while policy should aim to adapt buffer zones to the specific conditions of the area they are located in instead of a “one-size-fits-all” policy design. Barriers such as producer cooperation, communication gaps, and economic costs hinder progress. To address these barriers, potential solutions include improving stakeholder collaboration, emphasizing the multifunctional benefits of riparian buffers, strengthening compliance monitoring, exploring opportunities to provide technical support to farmers, and adopting integrated environmental management approaches. By addressing these interconnected challenges, riparian buffers can become resilient, multifunctional solutions that enhance ecosystem services, benefiting both biodiversity and human well-being in the SLRB.

## Introduction

Nature-based solutions are recognized as adaptive strategies that address diverse environmental challenges by leveraging complex ecosystem processes and functions. When designed and implemented effectively, they offer sustainable, resilient outcomes benefitting both the environment and society (Dunlop et al. 2024; Keesstra et al. 2018). Riparian buffer zones are highly effective nature-based solutions for mitigating water-related environmental issues. They intercept nutrients, sediments, and pollutants from agricultural runoff before entering waterways. They also stabilize streambanks, reduce soil erosion, regulate water temperatures, and provide critical habitats for wildlife (Kuglerová et al. 2014; Patowary et al. 2025). By generating such ecosystem services, riparian buffers help mitigate the adverse impacts of agriculture on water quality and enhance the overall health and resilience of watersheds (Cole et al. 2020).

The success of riparian buffer zones, however, depends on the careful integration of multiple factors. First, their functionality is underpinned by a combination of hydrological, vegetative, and geomorphological factors influencing the provision of ecosystem services (Prado et al. 2022). Hydrology shapes the flow paths and residence time of water, with surface runoff enhancing sediment trapping and filtration, while subsurface flow supports biogeochemical processes such as denitrification (Cole et al. 2020; Tabacchi et al. 2000). Vegetation contributes by filtering particulates, absorbing nutrients, stabilizing soil, and supporting microbial activity crucial for nutrient cycling (Dosskey et al. 2010). Geomorphological features, including slope and soil texture, influence flow velocity, direction, and infiltration capacity (Graziano et al. 2022). Together, these factors determine buffer zone performance and must be considered in the design and management of riparian buffers.

Hydrological, vegetative, and geomorphological features of buffer zones vary across landscapes, affecting how well they perform under different environmental conditions (Yang and Weersink 2004; Yorlano et al. 2022). To optimize functionality, buffer design can be tailored to these factors by adjusting parameters such as width, vegetation structure, and spatial configuration to match specific environmental pressures and ecosystem service goals (Cole et al. 2020; Riis et al. 2020). For example, areas with high runoff may require wider buffers or deep-rooted vegetation to enhance filtration, while steep slopes or highly permeable soils may need modified layouts to prevent erosion and increase infiltration (Stutter et al. 2019). A context-specific, adaptive design approach is essential to ensure riparian buffer zones function effectively across diverse landscapes.

Second, sustained management is critical for ensuring long-term functionality of riparian buffers. Periodic maintenance practices, such as vegetation control, grazing management, and removal of invasive species, help ensure that buffers continue to function effectively and do not become sources of additional problems, such as nutrient saturation or vegetation shifts (Maher Hasselquist et al. 2021). By tailoring management strategies to specific environmental goals and sustaining these efforts, riparian buffers can contribute to desired outcomes and long-term landscape resilience.

Third, proper biophysical functioning and effective management of riparian buffer zones depend on meaningful stakeholder engagement throughout the policy process. Interdisciplinary research highlights the growing global recognition of stakeholder engagement as essential for natural resource management (Bendtsen et al. 2021; Han et al. 2024), as it influences land use decisions (Barletti et al. 2020), supports compliance with environmental regulations through collective behavioral change (Eaton et al. 2021), and shapes how ecosystem services are valued and prioritized (Asah and Blahna 2020). In the context of nature-based solutions such as riparian buffers, engagement improves both quality and durability of ecosystem services while fostering a sense of ownership and stewardship among land users (Kiss et al. 2022). Stakeholder values, histories, and experiences shape local knowledge and risk perceptions, contributing important extra-scientific insights to decision-making (Harclerode et al. 2016; Malekpour et al. 2021). Involving stakeholders in the design and implementation of buffer zones promotes public acceptance and ensures integration of context-specific knowledge, ultimately enhancing sustainability, long-term management, and the provision of targeted ecosystem services (Ferreira et al. 2020; Nelson et al. 2020).

When biophysical functioning, sustained management, and stakeholder engagement are overlooked, the effectiveness of riparian buffer zones can be compromised. This can lead to insufficient nutrient retention, poor erosion control, harmful impacts on water flow dynamics, and issues such as nutrient saturation, invasive species integration, and diminished ecosystem services due to poor management (Cole et al. 2020). Stakeholder disengagement can further undermine policy compliance, reducing the potential for sustainable outcomes (Wagner 2008). These challenges pose risks to the long-term effectiveness of riparian buffer zones in achieving sustainable and desired outcomes.

Although studies have highlighted the ecological benefits of riparian buffers (e.g., Jager et al. 2023; Kuglerová et al. 2014), their successful implementation also depends on understanding stakeholders’ perceptions of the ecosystem services these zones provide, and how these services can be enhanced through improved design and management. However, stakeholder engagement in the design and management of nature-based solutions is often constrained by limited inclusion of local knowledge and insufficient mechanisms to translate participation into meaningful influence over decision-making (Bamzai-Dodson et al. 2024). For example, previous research has highlighted challenges such as low compliance and insufficient implementation of riparian buffer policies in Uruguay’s Santa Lucía River Basin (SLRB) (Ministerio de Ambiente 2023). Yet, how stakeholder perspectives on the implementation and management of buffer zones influence their motivation to comply with regulations, and which strategies might strengthen compliance, remain poorly explored.

To address this gap, we engaged stakeholders to gain deeper insights into perceptions, needs, and priorities regarding riparian buffer zones, aiming to identify key ecosystem services and characteristics (both current and desired) as well as the barriers and opportunities for implementing these services to improve the design, management, and overall effectiveness of these nature-based solutions. Specifically, we addressed three research questions: (1) *What ecosystem services are perceived by stakeholders to be currently provided by riparian buffer zones, and what additional services do they desire?*; (2) *What characteristics do stakeholders associate with effective buffer zones?*; and (3) *What are the perceived barriers and opportunities for improving the implementation and management of riparian buffer zones in the Santa Lucía River Basin?*

## Methods

### Study Area

**Fig. 1:**
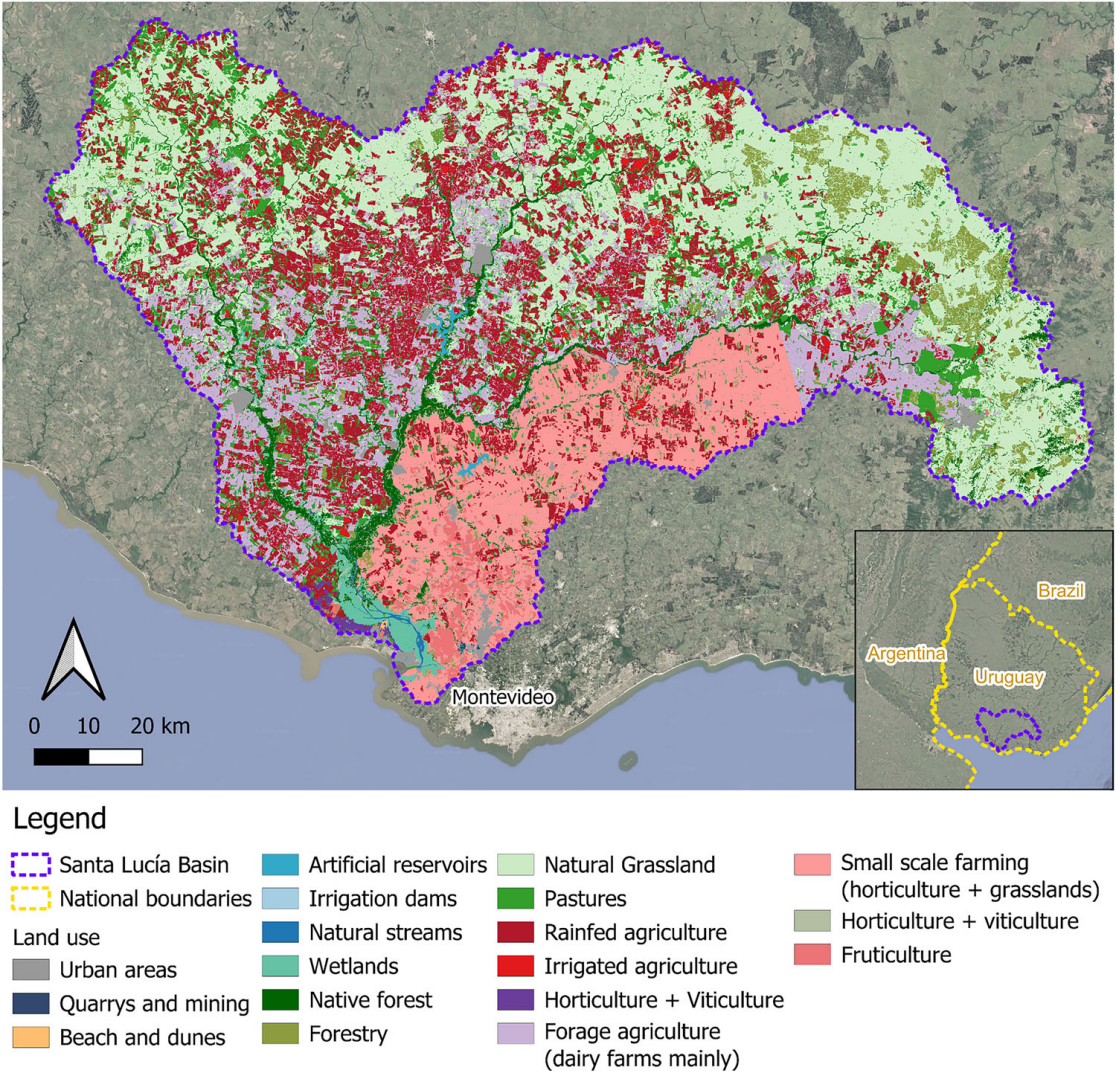
Land use map of the Santa Lucía River Basin, Uruguay. The map shows the spatial distribution of major land use categories, including agricultural types (e.g., rainfed, irrigated, and forage agriculture), natural vegetation (e.g., native forest, wetlands), and urban and industrial areas. Key hydrological and administrative features, such as natural streams, irrigation dams, and the basin boundary, are also indicated. Inset map shows the location of the basin within Uruguay and its position relative to national borders

The Santa Lucía River Basin (SLRB) is located in southern Uruguay, and is characterized by a mosaic of land uses reflecting both its ecological richness and economic significance. The basin encompasses diverse ecosystems, including riparian forests, wetlands, and natural grasslands, that are crucial for maintaining biodiversity and delivering essential ecosystem services such as water purification, erosion control, and habitat provision. Land use in the SLRB is shaped a range of agricultural activities. Extensive livestock farming, including cattle and dairy production, covers approximately 71% of the basin’s area (Fig. 1) (Achkar et al. 2012). Arable farming (16%), forestry (4%), horticulture (4%), and native forest (4%) are further major land uses. Spatially, land use varies along the river’s course: upstream regions are dominated by natural grasslands, native forests, and rainfed agriculture; the mid-basin supports a mix of arable farming, forage crops for dairy, and small-scale horticulture; downstream areas feature intensive smallholder agriculture, viticulture, fruticulture, and end in ecologically important wetlands. Despite the dominance of extensive livestock systems, the SLRB supports a relatively high diversity of productive land uses, reflecting its multifunctional landscape and the complex interplay between environmental and economic drivers (Aubriot et al. 2017; Achkar et al. 2012).

Water use in the SLRB is of high national importance, both for sustaining local livelihoods and for supplying essential services to Uruguay’s urban centers. The basin supports a mixed fluvial system with both lotic and lentic components, shaped by the presence of several artificial reservoirs. It experiences a highly irregular hydrological regime, governed by pluvial events (Achkar et al. 2014). In the SLRB, 79% of water is allocated to human consumption, as the river basin supplies drinking water to approximately 60% of Uruguay’s population, including the capital Montevideo and other cities in the Canelones department (Aubriot et al. 2017; Pirez Dastugue 2023). In addition to urban water supply, water use in the basin supports the extensive agricultural activities including irrigation, livestock farming, and dairy production (Bonilla et al. 2015). These multiple demands create significant upstream–downstream interdependencies, where the quantity and quality of water extracted or discharged upstream directly affect downstream availability and ecosystem health.

Due to intensive water use across the SLRB, the interplay between water quantity and quality in the SLRB is increasingly critical, particularly under shifting climatic conditions. The basin experiences a temperate climate with average annual rainfall ranging between 1000 and 1200 mm, and relatively low evapotranspiration rates contribute to high soil water availability (Säumel et al. 2023). While the region generally benefits from sufficient precipitation, its irregular hydrological regime marked by episodic droughts and intense rainfall events can significantly affect water quality. Periods of drought reduce streamflow and reservoir levels, concentrating pollutants and limiting dilution capacity, as seen during the prolonged dry spell in 2023. This led to the use of alternative sources with inferior water quality, resulting in months of reduced drinkability of tap water (Goyenola 2023). Conversely, heavy rainfall and flooding events, expected to become more frequent under climate change, increase runoff from agricultural land and urban areas, intensifying non-point source pollution such as nutrients and agrochemicals. These dynamics contribute to harmful algal blooms, like the toxic event of March 2013, which severely disrupted water purification and access to safe drinking water (Goyenola et al. 2021). Thus, water quantity fluctuations, whether from drought or excess rainfall, have direct and compounding effects on water quality.

To better deal with these management challenges, water management in the SLRB has undergone a major transition over the past two decades, aligning with broader national reforms aimed at sustainable and participatory governance of water resources. Once managed under a centralized model, the basin is now governed through a decentralized, integrated framework initiated by the 2004 constitutional reform and consolidated in the 2009 National Water Policy (Law No. 18.610) (Trimble et al. 2021). These reforms recognized water as a public good and fundamental human right, and introduced watershed-based management overseen by the former National Environment Directorate (DINAMA) and the current Ministry of Environment. As part of this shift, multistakeholder bodies such as the Santa Lucía River Basin Commission were established to bring together government agencies, civil society, and water users to collaboratively define priorities, inform policy, and support implementation at basin level.

**Fig. 2:**
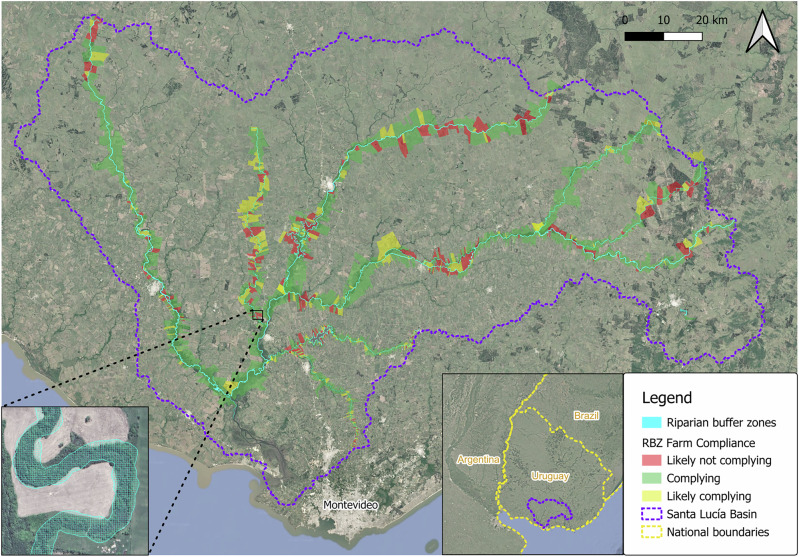
Riparian Buffers in the Santa Lucía River Basin, Uruguay. The map delineates the extent of riparian buffers established as part of the Santa Lucía Action Plan Farms are color-coded by their level of compliance with riparian buffer zone (RBZ) regulations: red (likely not complying), yellow (likely complying), and green (complying). The map in the lower-right corner provides a geographical context for the Santa Lucía River Basin within Uruguay and the broader region. In the lower-left corner, an example overview of the watercourse and surrounding riparian buffer zone is provided. Source: Adapted from Ministerio de Ambiente (2023)

The Uruguayan government, under consultation of the Santa Lucía River Basin Commission, implemented the “Plan de Acción Río Santa Lucía” to improve water quality in the SLRB. This governmental action plan includes measures to reduce point and diffuse source pollution, such as improved industrial and domestic wastewater treatment, effluent management on dairy farms, fertilizer and pesticide regulations, cattle exclusion from waterways, implementation of riparian buffer zones; and actions to regulate water use, halt feedlot expansion, and enhance stakeholder participation and data collection in the basin. Specifically, riparian buffer zones became a priority to combat water quality problems related to nutrient enrichment of freshwater bodies, as eutrophication partly caused by agricultural non-point source pollution (especially phosphorus runoff) is a key contributing factor to basin-wide water quality degradation (Aubriot et al. 2017) (Fig. 2).

These buffer zones were designed to reduce nutrient runoff, intercepting pollutants and stabilizing streambanks to enhance ecological health and hydro-morphological conditions of the basin (MVOTMA 2013). The action plan defined these buffer zones as areas where tillage and agrochemical use are prohibited, and where cattle are prevented from accessing both the buffer areas and watercourses. This approach aims to minimize the impact of livestock on vegetation and water quality by reducing diffuse sources of pollution. The buffer widths vary based on stream order, with main river courses requiring 40-meter buffers, first-order tributaries 20 meters, and reservoirs 100 meters (MGAP et al. 2018; MVOTMA 2013, 2015). Trough pollution retention, streambank stabilization, and erosion control, buffer zones are thus meant to contribute to improved surface water quality in the river basin.

Stakeholder consultation through the governmental action plan contributed to the broader implementation of riparian buffer zones throughout the basin, with the purpose of (1) enhancing the effectiveness of sustainable resource management by integrating local knowledge and interests (Pigmans et al. 2019) and (2) fostering long-term success through voluntary cooperation, which increases understanding and acceptance of management plans (Buckley et al. 2012). Despite these efforts, research indicates that anthropogenic land uses within buffer zones reflect potential compliance problems including agricultural expansion and intensification in designated buffer areas (Mary-Lauyé et al. 2023; Ministerio de Ambiente 2023). While research has assessed the physical feasibility of riparian buffer zones in the SLRB to contribute to solving water quality issues targeted by the riparian buffer zones (e.g., Calvo et al. 2024; Mary-Lauyé et al. 2023), little attention has been paid to social perspectives of stakeholders to enhance acceptance and effectiveness of these measures. Exploring stakeholder perspectives on ecosystem services, buffer zone characteristics, and the barriers and opportunities to implementation can provide actionable insights for improving buffer zone policies and management strategies in the SLRB.

### Data Collection

We conducted 24 semi-structured interviews with stakeholders involved in the design and management of riparian buffer zones in the SLRB (Table 1). Interview transcripts are included in the Supplementary Materials. Participants were identified through snowball sampling, which ensured diverse perspectives and minimized bias by starting from multiple initial contacts (‘snowball slopes’).

**Table 1:** Overview of interviewed stakeholders and stakeholder groups

Stakeholder group	Stakeholder name	Acronym	Amount of interviews	Interview numbers
Government	National Water Directorate	DINAGUA	1	6
	National Directorate of Environmental Quality and Assessment	DINACEA	2	10, 12
	Former-National Directorate of Territorial Planning	DINOT	1	21
	Ministry of Livestock, Agriculture, and Fisheries	MGAP	2	17, 19
Research	National Institute for Agricultural Research	INIA	1	15
	Scientists (including agronomists, ecologists)		7	3, 7, 8, 9, 18, 22, 23
Producer unions	Federation of Agricultural Cooperatives	CAF	1	24
	National Commission for Rural Development	CNFR	1	5
NGO	Uruguayan Center for Appropriate Technologies	CEUTA	1	2
	Vida Silvestre		1	20
Producers			5	4, 11, 13, 14, 16
Locals			1	1

The target group comprised representatives from government institutions, research stakeholders, producer unions, producers, NGOs, and local community members, based on their participation in the formulation of the governmental action plan. Interviews were conducted between March and May 2023, in both Spanish and English to accommodate participants’ language preferences. Interviews typically lasted approximately one hour, with deviations made to the interview guide based on the input and responses of the interviewees. Interviews were conducted both face-to-face and online, depending on availability and preferences of participants. Interviews were transcribed using Whisper (an automated transcription software; Radford et al. 2023) and were translated manually, with Spanish transcripts proofread by native speakers. Participants provided informed consent before engaging in interviews, granting permission for (audio) recording and acknowledging the option to terminate the interview at any point. Following the analysis, a follow-up meeting was conducted with interviewees to disseminate and validate the findings. Participants were given the opportunity to review their responses and provide feedback on transcripts, ensuring accuracy and reliability of the data.

Government institutions were represented by the National Water Directorate (DINAGUA), the National Directorate of Environmental Quality and Assessment (DINACEA), the former National Directorate of Territorial Planning (DINOT), and the Ministry of Livestock, Agriculture, and Fisheries (MGAP). These institutions played a vital role in riparian buffer policy due to their regulatory authority and responsibility for protecting public water resources (MVOTMA 2013). They were involved in every step of the formulation of the governmental action plan.

Research stakeholders and producer unions were involved through consultation in the formulation of the governmental action plan (MVOTMA 2013). Research stakeholders included local and national scientists, and were consulted for their expert knowledge and policy advice, contributing valuable insights into the design and management of buffer zones. Producer unions represented agricultural interests and ensured that perspectives and needs of farmers were included in discussions and the formulation of the governmental action plan.

Producers, NGOs, and locals were stakeholders that were being informed about the governmental action plan and facilitated the implementation of buffer zones. Producers themselves were involved as those responsible for implementing and maintaining buffer zones, playing a crucial role in their practical application. NGOs, encompassing the Uruguayan Center for Appropriate Technologies (CEUTA) and Vida Silvestre, helped raising public awareness as part of the governmental action plan. Finally, local community members were included as informed participants through local news outlets.

### Interview Strategy

Our methodology involved three key steps: (1) determining the current and desired ecosystem services provided by buffer zones, (2) assessing the current and desired buffer zone characteristics, and (3) identifying barriers and opportunities for improving policy implementation. These topics were translated into specific questions, which were compiled in a structured interview guide (Supplementary Materials).

First, we identified perceptions of current and desired ecosystem services provided by riparian buffers. Respondents were asked about benefits that buffer zones provide through open questions such as “*What benefits do you think buffer zones currently provide?*” and “*What benefits would you like buffer zones to provide?*” To determine the importance of perceived benefits, we employed a comparative scoring method. Interviewees were asked to rate current and desired ecosystem services they identified on a scale from 1 to 4, where 1 was ‘unimportant’ and 4 was ‘most important’. Based on the MEA ecosystem services framework (Millennium Ecosystem Assessment, 2005), we categorized the benefits into four types of ecosystem services after the interviews: cultural services (nonmaterial benefits), provisioning services (product-based benefits), regulating services (benefits from ecosystem process regulation), and supporting services (services that support other ecosystem services) (see section 2.4). The resulting data were analysed to evaluate relative importance and frequency of mention of each ecosystem service.

Second, we identified the current and desired characteristics of riparian buffers. This characterization was based on open questions such as *“What features characterize buffer zones currently*?” and *“What characteristics do you think need to change or be added to (the design of) riparian buffers to support the mentioned benefits?”* After the interviews, we broadly categorized these characteristics into three groups: physical attributes (observable features), management aspects (human activities and interventions), and policy elements (legal, institutional, and governance frameworks). The relative importance of resulting characteristics was determined based on their frequency of mention.

Third, to understand barriers and opportunities for improving the design and management of riparian buffers as perceived by stakeholders, open-ended questions such as *“What do you think the benefits of [the described] changes would be?”* and *“What obstacles do you think will be encountered in implementing these changes?”* were asked. After the interviews we categorized perceived barriers and opportunities based on their perceived importance among stakeholders, expressed as their frequency of mention among interviews.

We acknowledge that the use of semi-structured interviews to elicit stakeholder perceptions introduces a degree of subjectivity, both in terms of participant responses and researcher interpretation. Stakeholder views on ecosystem services and buffer zone characteristics are inherently shaped by personal experiences, professional backgrounds, and contextual knowledge, and may vary significantly across individuals and sectors. While the interviews provided valuable insights into the desired ecosystem services and buffer zone characteristics, not all possible stakeholders could be interviewed, and our results and conclusions are thus limited to represent the views of participating respondents.

### Data analysis

**Fig. 3:**
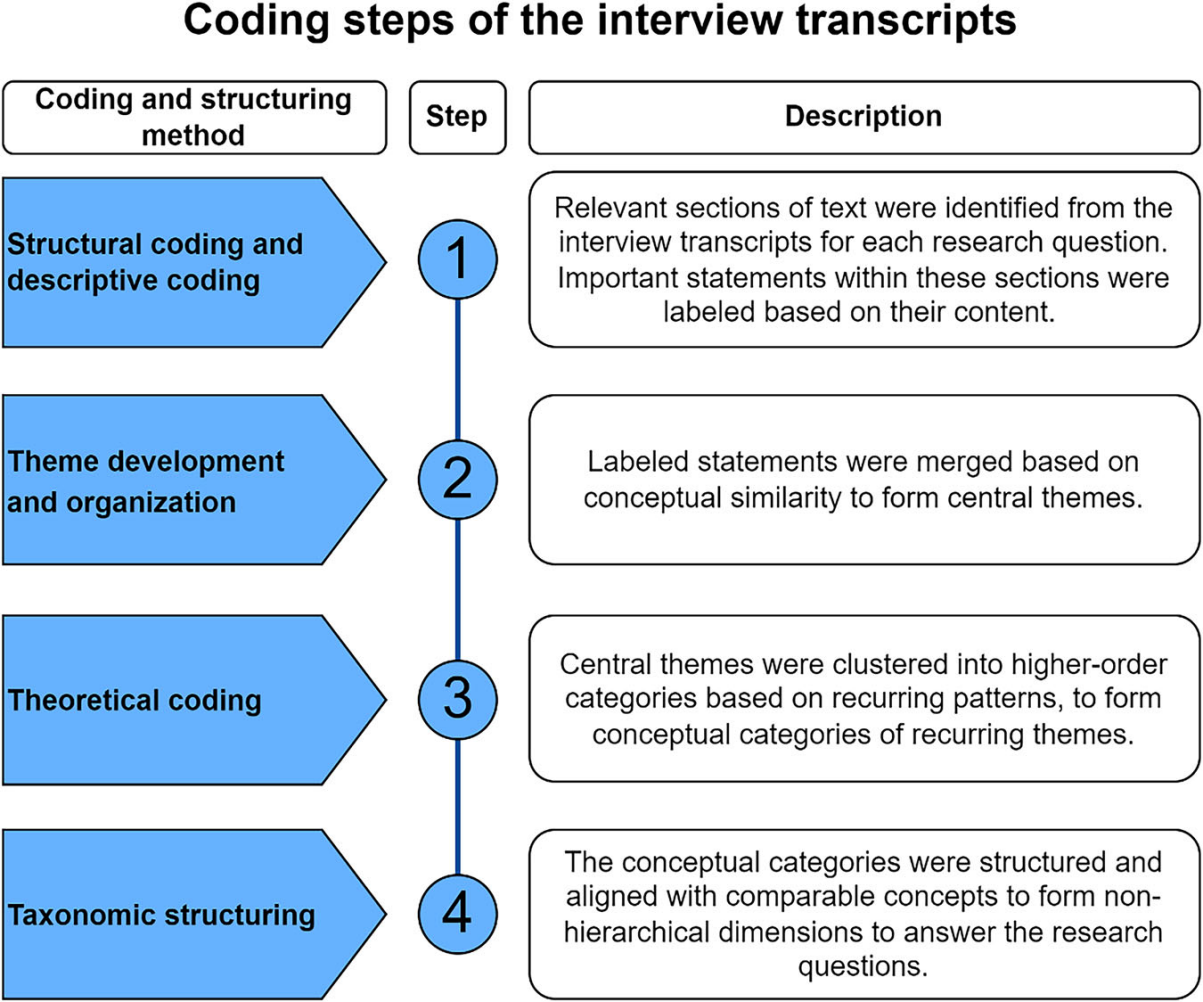
Workflow of the coding and structuring process used for the thematic analysis of interview transcripts. The process involved four iterative steps: (1) identifying and labeling relevant statements through structural and descriptive coding; (2) grouping labeled statements into central themes; (3) clustering themes into higher-order conceptual categories through theoretical coding; and (4) organizing these categories into non-hierarchical dimensions to address the research questions

Our data analysis followed an inductive thematic analysis approach (Kyngäs et al. 2020), which allows for patterns, themes, and categories to emerge from the data to reflect stakeholder perspectives. The analysis was conducted using MAXQDA, where all interview transcripts were organized into a single project folder. Within this folder, sub-folders were based on the categories of codes identified. A *code* was defined as a word, segment, or phrase that captures the core meaning of a passage or statement (Saldaña, 2013). The coding process followed four iterative stages (Fig. 3). All codes were organized into a codebook detailing the coding framework along with corresponding supporting data segments (Supplementary Materials).

In the first step, structural and descriptive coding methods were applied. Structural coding is a top-down approach where broad, research-driven topics are used to segment the data (Saldaña 2013). In this case, sections from the transcribed interviews were extracted according to major discussion areas relevant to the research questions, encompassing (1) “buffer zone benefits”; (2) “buffer zone characteristics”; (3) “barriers in buffer zone design and management”; and (4) “opportunities in buffer zone design and management.” Additionally, descriptive coding was used to identify relevant statements from stakeholders within these extracted sections. Descriptive coding is a bottom-up approach that summarizes the basic topic of each passage using short, content-reflective labels (MacQueen et al. 2008). For example, labels (descriptive codes) such as “water purification,” “grazing benefits,” and “sediment entrapment” were applied to specific quotations in a broader section related to “buffer zone benefits” to capture key stakeholder statements. In MAXQDA, this step was conducted by coding transcript segments with broad head-level codes (i.e., the structural codes), followed by applying nested subcodes for descriptive content.

The second step focused on data organization and theme development. This phase involved iterative re-coding and realignment of emerging patterns into thematic categories (Auerbach and Silverstein 2003). Labels (descriptive codes) from the first round were compared and clustered based on conceptual similarity, for example combining subcodes such as “buffer nutrient uptake” and “soluble nitrogen reduction” into the broader theme of “nutrient retention”, which were nested in the structural code of “riparian buffer benefits.” In MAXQDA, this was done by refining the coding tree structure: sub-folders were created under each structural code to organize these subcategories.

The third step involved categorizing thematically clustered descriptive codes from the previous phase into higher-level theoretical categories (Saldaña 2013). This entailed systematically reviewing each group of related codes to identify overarching conceptual patterns that could unify them under broader categories. For example, the themes of “nutrient retention” and “erosion reduction” were placed under the category of “regulatory benefits.” These theoretical categories served to abstract and organize the data in a way that highlighted systemic relationships and recurring dynamics across the dataset. MAXQDA’s code grouping and memo functions were used to support this process by enabling the visual comparison of code clusters, documentation of emerging insights, and iterative refinement of category boundaries.

The fourth step involved applying a taxonomic approach to structure the coding framework in alignment with the research questions. This process focused on organizing theoretical categories developed in the previous step under broader analytical dimensions that corresponded to the main areas of inquiry (Saldaña 2013). The aim was to ensure that the coding structure not only reflected patterns in the data but also provided a coherent framework for addressing the research questions. Within MAXQDA, this involved aligning the theoretical categories with the predefined structural coding categories and organizing underlying descriptive codes accordingly. Descriptive codes were reviewed and reclassified to ensure they logically fit within the relevant theoretical categories, maintaining consistency and conceptual clarity. For example, the categories of “regulatory benefits” and “ecosystem regulation” were merged and defined as “regulating ecosystem services.” This resulted in a multi-level coding structure in which research questions guided the top-level categories, supported by thematically grounded subcategories and detailed descriptive codes nested within them.

**Fig. 4:**
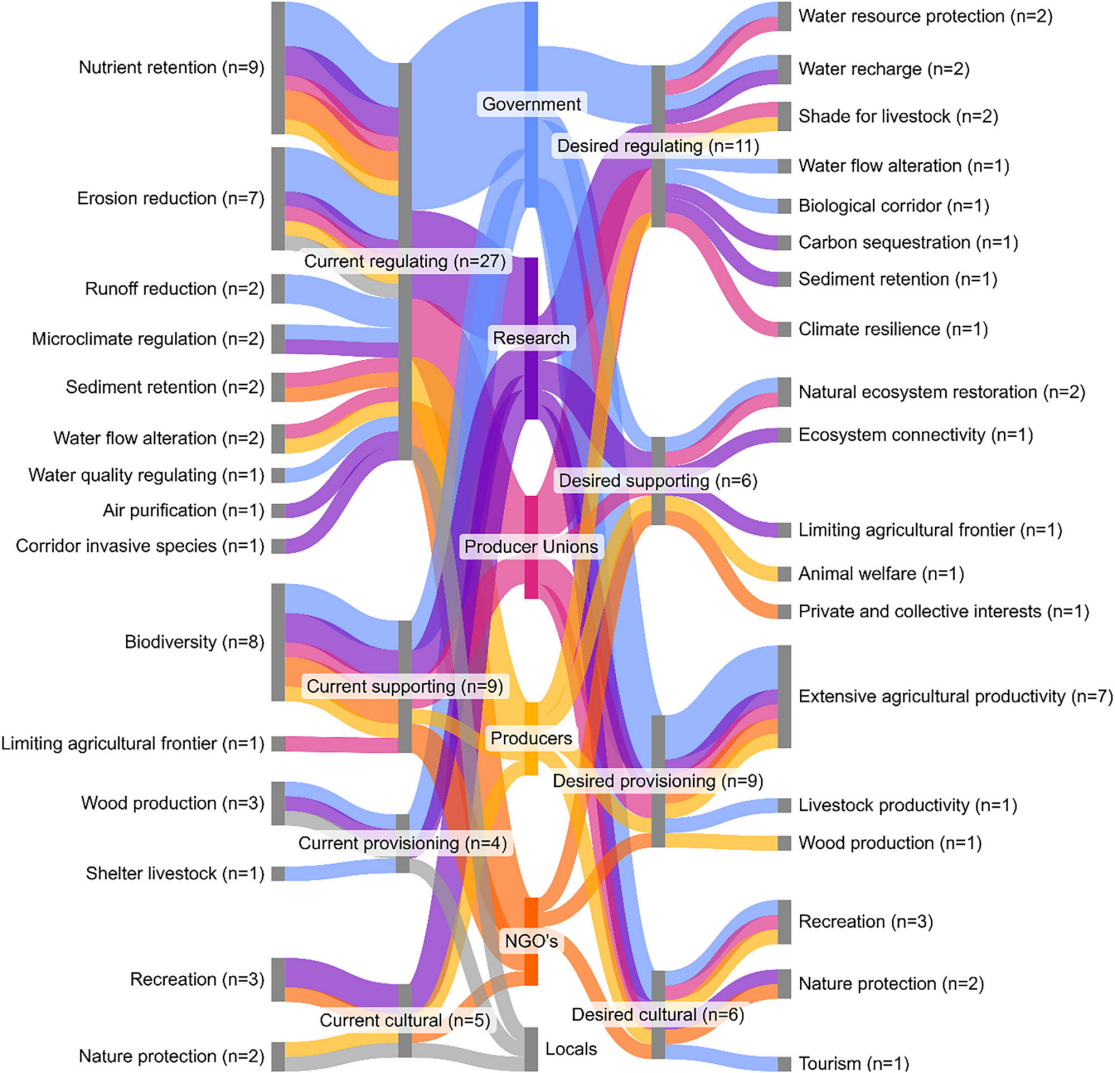
Overview of the current and desired ecosystem services provided by riparian buffer zones, perceived by different stakeholder groups in the Santa Lucía river basin. On the left, the different perceived currently provided ecosystem services are shown, for different ecosystem services categories that they are part of. On the right, the different desired provided services, that are additional to the currently provided services, are shown for their respective category. The width of the flows and nodes indicates the amount of stakeholders per stakeholder group that mentioned a specific ecosystem service or ecosystem services category, with the frequency of mention across interviews displayed with (*n* = x). It is important to note that locals did not identify desired additional ecosystem services

To assess the relative prominence of descriptive codes, a frequency-based scoring method was applied. The scores for ecosystem services and riparian buffer characteristics provided by interviewees (i.e., scale from 1 to 4) were averaged over the number of interviews in which said ecosystem service or characteristic was mentioned. These scores were not interpreted as statistical findings but rather as indicators of salience or shared perception. This quantification informed the creation of Sankey diagrams (Figs. 2, 3) generated using Sankeymatic, which illustrated relationships between stakeholder groups and perceived ecosystem services or characteristics. A final conceptual diagram (Fig. 4) was created in Inkscape to visualize the taxonomic structure, showing how categories, subcategories, and key stakeholder insights were interconnected across the four coding layers.

## Results

### Current and Desired Ecosystem Services of Riparian Buffer Zones

Stakeholders identified a total of 15 current ecosystem services provided by riparian buffer zones (Fig. 4). These included nine regulating services, two supporting services, two provisioning services, and two cultural services. Additionally, stakeholders expressed a desire for 19 ecosystem services to be provided by riparian buffers, which included eight regulating services, three provisioning services, three cultural services, and five supporting services (Fig. 4).

Five ecosystem services were highlighted as being of particular importance within the SLRB. These included three current services (pollution retention, erosion reduction, and biodiversity conservation) and two desired services (extensive agricultural production and recreational opportunities). Among these, pollution retention had the highest score and frequency of mention (mean score = 3.72, frequency of mention = 18 respondents). Stakeholders highlighted the role of riparian buffer zones in enhancing water quality, with several emphasizing that these buffers “*fulfil the function of protecting water quality through nutrient retention*” (Interview 6) and “*reduce the contribution of agrochemical substances to water quality issues*” (Interview 3). This aligned closely with the primary objective of buffer zones to mitigate impacts of agricultural runoff on water bodies. Erosion reduction was perceived as another important currently present ecosystem service, with the second highest score and third highest frequency of mention (mean score = 3.17, frequency of mention = 14 respondents). It was noted that “*soil erosion represents a significant challenge within the basin, primarily due to the transport of phosphorus through eroded soil particles*” (Interview 8). Stakeholders perceived that vegetation structure played a vital role in stabilizing soil through its root systems, mitigating erosion (Interview 8, 13). Biodiversity conservation was perceived as another significant currently present ecosystem service (mean score = 3.13, frequency of mention = 16 respondents). Stakeholders recognized the role of riparian buffers in enhancing ecosystem resilience through biodiversity (Interviews 3, 7, 13, 15), noting that “*the local biodiversity in these areas helps ecosystems recover from environmental stress and contributes to the production of high-quality drinking water*” (Interview 3).

Regarding desired ecosystem services, provisioning and cultural services emerged as priorities. Extensive agricultural production, characterized as farming practices with low environmental impact, was identified as the most frequently mentioned and highest scoring desired service (mean score = 3.25, frequency of mention = 14 respondents). Stakeholders expressed concerns about the economic viability of buffer zones for smallholder farmers (Interview 2, 5, 6). Integrating extensive agricultural productivity into buffer zone management could alleviate some economic pressures while enhancing the maintenance in these zones to preserve their ecological functions. For instance, several participants noted the risk of nutrient saturation in poorly maintained buffer zones, leading to phosphorus accumulation that could eventually enter waterways (Interviews 17, 22). Extensive agricultural productivity was seen as a potential solution for removing excess nutrients and maintaining buffer functionality (Interview 23). Additionally, stakeholders expressed concern over the threat of exotic vegetation. Extensive agricultural productivity was seen as a tool for managing invasive species, with one respondent stating that buffer zones could “*function as corridors for invasive species…[while] with grazing, you have them more under control*” (Interview 9). The final important desired ecosystem service identified was the recreational use of riparian buffer zones (mean score = 3.50, frequency of mention = 6 respondents). Stakeholders noted that riparian zones could serve as vital spaces for activities such as hiking, birdwatching, and fishing, contributing to both physical health and mental well-being (Interviews 4, 6).

Stakeholders also identified several lesser-recognized ecosystem services provided by riparian buffer zones that extend beyond their commonly recognized functions. These included enhanced pollination services, where buffer zones *“support a variety of pollinators that benefit surrounding agricultural and natural systems”* (Interview 2). Another notable service was climate change mitigation, with buffer zones acting as carbon sinks and contributing to broader climate regulation efforts (Interview 7). Buffer zones were also recognized for their value as educational and research sites, *“providing opportunities for hands-on learning about ecosystems and environmental stewardship”* (Interview 4). These lesser-recognized services underscore the diverse and multifaceted benefits of riparian buffers beyond their primary functions.

### Current and Desired Characteristics of Riparian Buffer Zones

**Fig. 5:**
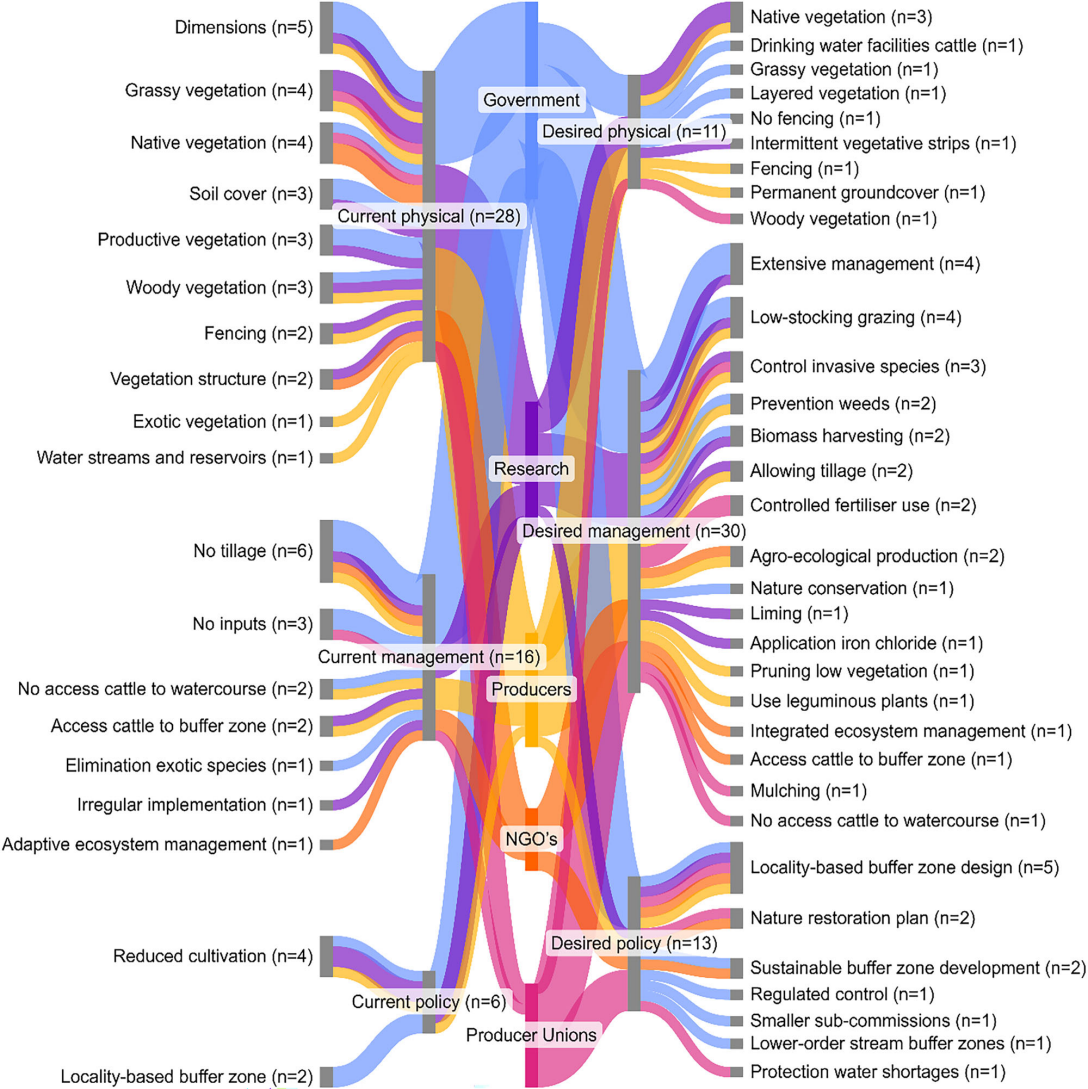
Overview of the current and desired characteristics of riparian buffer zones as perceived by different stakeholder groups in the Santa Lucía River Basin. On the left, the different perceived current characteristics are shown, for different categories that they are part of. On the right, the different desired characteristics, additional to the perceived current characteristics, are shown for their respective category. The width of the flows and nodes indicates the amount of stakeholders per stakeholder group that indicated a specific characteristic or characteristic category, with the frequency of mention across interviews displayed with (*n* = x). Note that locals did not provide input on perceived characteristics

Stakeholders identified 19 current riparian buffer zone characteristic services: 10 physical characteristics, seven management characteristics, and two policy characteristics (Fig. 5). In addition, 33 desired characteristics of riparian buffer zone were identified, including nine physical characteristics, 17 management characteristics, and seven policy characteristics (Fig. 5).

The most frequently-mentioned currently present riparian buffer zone characteristics were the presence of grassy and woody native vegetation (frequency of mention = 18 respondents), the buffer zone dimensions (frequency of mention = 8 respondents) and the prohibition of tilling (frequency of mention = 12 respondents). A mix of native grassy and woody vegetation was perceived to be most suitable for buffer zones because it provides a multitude of additional ecosystem services. Stakeholders mentioned that “*… if you conserve the native forest … the cattle have shade, and you can have firewood”* (Interview 13). Additionally, they noted that *“… when climate problems arise, native vegetation can be more resilient than productive exotic species”* (Interview 13). Furthermore, the current spatial dimensions and no-tillage management practices in buffer zones were frequently mentioned, as *“… the goal is to restore the area to its natural state, which may include measures such as no soil tillage, no application of agrochemical fertilizers, and maintaining natural vegetation”* (Interview 15).

Stakeholders furthermore discussed several frequently-mentioned desired characteristics: a locality-based design, presence of native vegetation over invasive vegetation, extensive agricultural management, and low-stocking grazing. First, a locality-based character for buffers, meaning designs that vary according to local socioenvironmental characteristics rather than a one-size-fits-all solution, emerged as the frequently discussed desired aspect (frequency of mention = 10 respondents). A common perception was that “*the functions that buffer zones should fulfil differ depending on what type of areas we are considering and the uses that are given to them”* (Interview 18). Additionally, stakeholders emphasized that locality-based buffers enhance ecosystem service provision, as they could create “*a virtuous circle of producing more biomass, generating better animal welfare, and achieving better productivity and yields. However, it has to be implemented on a case-by-case basis, according to the shape of the property, the location, and the socioeconomic level*” (Interview 2). This highlights the importance of considering more than just physical characteristics.

Second, extensive agricultural management (frequency of mention = 8 respondents) and low-stocking grazing (frequency of mention = 8 respondents) were other frequently-mentioned desired characteristics. One interviewee noted that “*with grazing, you can better control invasive vegetation that encroaches the fields*” (Interview 9). It was also mentioned that allowing cattle to graze in buffer zones could provide them with shade and shelter in the forest (Interview 17). This approach could foster a positive perception among farmers, as it frames buffer zones as beneficial to the production system (Interview 2). Additionally, stakeholders believed that extensive practices, such as rotational grazing, have a less significant impact on water quality than current agricultural contributions (Interview 11). Last, the control of invasive vegetation was another desirable characteristic (frequency of mention = 6 respondents). Invasive vegetation was described as problematic because it can lead to increased encroachment, creating areas of productive land that cattle can no longer access (Interview 4). Therefore, controlling invasive species was seen as “*crucial for sustaining and conserving the biodiversity and productivity of the system*” (Interview 3).

### Barriers and Opportunities for the Implementation of Desired Services and Characteristics

**Fig. 6:**
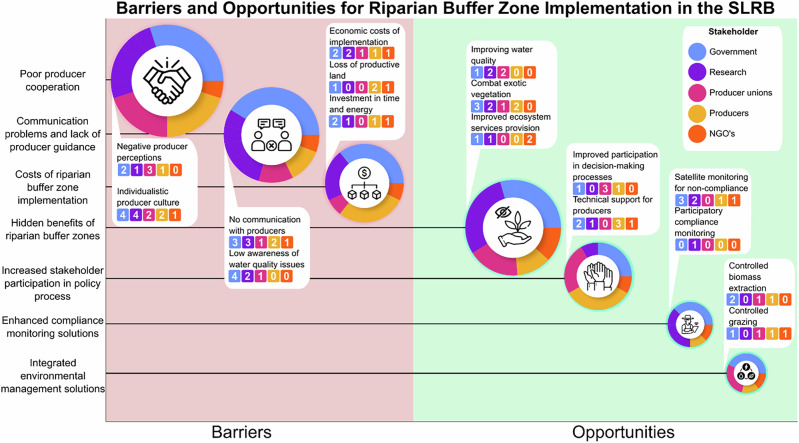
Overview of stakeholder-identified barriers and opportunities for improving the implementation of riparian buffer zones (RBZs) in the Santa Lucía River Basin. The figure presents three barriers and four opportunities. The size of each pie chart reflects the frequency with which each category was mentioned. Text boxes provide further detail on individual components, including the number of mentions per stakeholder group

Stakeholders identified three barriers and four opportunities for enhancing the implementation of additional desired ecosystem services and characteristics (Fig. 6). We first present the three primary barriers (poor producer cooperation, communication issues, and economic costs), and later the opportunities.

The most frequently cited barrier was poor producer cooperation (frequency of mention = 20 respondents). Many producers were perceived as not adhering to management responsibilities outlined in the governmental action plan; some ignored buffer boundaries, allowed cattle to access buffer zones, or used these areas for cultivation (Interview 3). This noncompliance stemmed from two key factors: negative perceptions of riparian zones and an individualistic culture among producers. Producers often viewed buffers as “*mugre*” (muck, grease, or filth) due to their belief that these zones could cause various problems (Interview 7). This perception was linked to management issues stemming from invasive exotic vegetation that negatively affected productive land (Interviews 6, 14, 17), increased wildfire risk (Interview 4, 8, 9), and attracted invasive animal species (Interview 5). One stakeholder remarked, “*These issues were exacerbated by a tendency among producers to prioritize individual decision-making, leading them to manage the land as they saw fit*” (Interview 10). This approach to land management further influenced their reluctance to fully engage with the action plan.

The second most frequently mentioned barrier related the implementation of desired ecosystem services and characteristics was communication issues between producers and policymakers (frequency of mention = 17 respondents). Producers outside of the river basin commissions indicated that “*they have never received any official communication, neither from the (river basin) commission nor from the ministry of agriculture*” (Interview 4). The absence of knowledge on existing and accessible communication channels had left many farmers unaware of the purpose, benefits, and management practices associated with buffer zones. Various stakeholder groups noted that the unfamiliarity with such communication channels contributed to producers’ unawareness of the issues caused by agricultural practices (Interviews 8, 12, 15). Additionally, there was a perceived deficiency in guidance and technical support for the farmers who were informed (Interviews 8, 12). This gap led producers to undervalue the role of riparian buffer zones, primarily because they had not been adequately educated about their significance (Interview 24).

The third most frequently-mentioned barrier identified was economic costs associated with implementing buffer zones (frequency of mention = 14 respondents). Interviewee 2 emphasized that “*an investment needs to be made in time, energy, resources, and training*,” pointing out that costs extend beyond the financial aspects of establishing and managing riparian buffer zones. The potential use of economic incentives to support implementation in the SLRB was seen as complex due to the lack of subsidies or compensation policies for producers, despite being viewed as a viable option to enhance compliance (Interviews 6, 10). As Interviewee 10 stated, “*Every time one poses limitations, damage compensation appears in the discussion. If you are limiting me, compensate me because you are restricting my free use and my economic possibilities… If I get no compensation, the policy has no support*.”

In contrast to the barriers, four main opportunities to improve riparian buffer zones in the SLRB were identified: buffer zone multifunctionality, increased stakeholder participation, integrated environmental management, and enhanced compliance control.

The most frequently mentioned opportunity was the potential of lesser-recognized ecosystem services to enhance the multifunctionality of riparian buffer zones, supporting the needs of multiple stakeholders simultaneously. Beyond improving water quality, buffer zones were described as “*areas with enriched biodiversity and pollen production that enhance certain functions of agricultural and livestock systems*” (Interview 2). Additionally, well-maintained buffer zones could “*help combat exotic vegetation, as maintaining diversity helps prevent colonization by invasive species*” (Interview 15). Furthermore, stakeholders noted that buffer zones could increase the resilience of both ecosystem and production system, as they provide permanent pastures, cooler temperatures, riparian cover for soil and water, and maintain higher groundwater levels (Interview 8).

The second most frequently-mentioned opportunity was the potential of increased participation of stakeholders in the policy process to enhance the effectiveness of riparian buffer zones in the SLRB (frequency of mention = 12 respondents). Stakeholders highlighted that *“[there is the need to] reach out to producers so that they start implementing measures in the buffer zones, and they need support”* (Interview 5). Stakeholder empowerment could consist of increased collaboration between stakeholders in the decision-making process and producer involvement in policy implementation. Government institutions could support this effort, as *“they could interact with producers through development roundtables”* (Interview 12). Additionally, it was mentioned that the government could support producers in the maintenance of riparian buffer zones by providing technical support, as “*the goal should not be to pay producers for their work but to educate them on the importance of the buffer zones*” (Interview 4). This technical support could encompass advisory services, on-the-ground training, and educational workshops. Similarly, it was mentioned that *“producers listen to the state, but … the state also [needs to] listen to the small producers, who [experience] limitations in the field”* (Interview 14). To accomplish this, *“you [need to] have a long-term policy involving the ministries, and many producers need to hear [what the results are] and that it works”* to feel motivated and comply (Interview 9).

Enhanced compliance control was the third most frequently-mentioned opportunity (frequency of mention = 8 respondents). It was believed that monitoring is important, as nonenforcement of compliance control could incentivize noncompliance with buffer zone policies (Interview 10; 12). An interviewee mentioned, *“it is possible to overcome obstacles by paying more attention to specific situations, applying resources effectively, and implementing better control measures”* (Interview 4). In addition, stakeholders believed that *“monitoring noncompliance in the field of buffer zones [could be solved] with satellite monitoring”* (Interview 3). One proposed mechanism that could be used to enhance compliance control was the use of producer participation in the monitoring process by using *“complaints from producers, who can report these violations, and authorities can take necessary action”* (Interview 17). In this way, better monitoring mechanisms could be created.

Integrated environmental management was perceived as the fourth most frequently-mentioned opportunity (frequency of mention = 7 respondents). By integrating buffer zones in the production system, producers *“start looking at different reasons for having different ecosystems in different places”* (Interview 22). Furthermore, a focus on ecological integrity was perceived to be needed while trying to integrate buffer zones into the production system, as *“the forests belong there as much as other vegetation”* (Interview 22). Specific examples mentioned of this approach included intermittent grazing and controlled biomass extraction to prevent nutrient saturation in buffer zones, which can reduce the effectiveness of riparian buffers (Interview 4; 9; 15; 23). In addition, it was perceived that through controlled grazing, *“you collect the growth of forage to encourage [vegetative] regrowth. Resulting from this is that the vegetation returns with more vigour”* (Interview 13). To operationalize integrated environmental management, it was mentioned that riparian buffers could be embedded in current land-use policies, such as the ‘Territorial Planning Act’ or the ‘Land Use and Management Plan’ enforced by the Ministry of Agriculture (Interview 21).

## Discussion

This study investigated stakeholder perspectives on the implementation and management of riparian buffer zones in the SLRB. We uncovered the diverse perceptions and desires of these stakeholders and synthesized their views on ecosystem services provided by riparian buffers. Additionally, we identified key physical, management, and policy characteristics deemed necessary for effective buffer zone functioning. Furthermore, we revealed both barriers to and opportunities for improving the implementation and management of these buffer zones.

### The Need for Multifunctional and Context-specific Riparian Buffers

We identified five ecosystem services as particularly important within the SLRB: pollution retention, erosion reduction, biodiversity conservation, extensive agricultural production, and recreational opportunities. These services span regulating, supporting, provisioning, and cultural domains, reflecting the multifunctional expectations placed on riparian buffer zones in the SLRB. Delivering these services, however, requires distinct—and at times conflicting—riparian buffer characteristics that influence core biophysical processes such as hydrology, geomorphology, and nutrient cycling.

The characteristics that underpin identified regulating and supporting ecosystem services in riparian buffers show potential synergies. Services such as pollution retention and erosion control are enhanced by wide buffers with dense, tall, and deep-rooted vegetation. These features increase the interception of diffuse pollution by extending the buffer’s distance from agricultural inputs and enlarging the vegetated zone that promotes infiltration, sediment trapping, and nutrient cycling (Knight et al. 2010; Zhang et al. 2010). Dense vegetation enhances hydraulic roughness, slowing surface runoff and increasing sedimentation, while deep root systems improve soil structure and facilitate nutrient uptake from deeper layers (Dosskey et al. 2010; Rood et al. 2014). These same characteristics simultaneously support biodiversity conservation by providing wide, structurally complex, and minimally disturbed habitats that accommodate diverse species across trophic levels (Ó hUallacháin et al. 2014; Lind et al. 2019). Wider buffers reduce disturbance from adjacent land use, creating stable, low-nutrient conditions that favor species adapted to such environments and enhance floral resource availability for pollinators (Cole et al. 2015). These overlapping requirements highlight a degree of alignment between water quality and biodiversity-related goals, suggesting that synergies can be leveraged in buffer design to support multiple regulating and supporting services simultaneously.

However, while structural characteristics that support regulating services can align with biodiversity goals, they potentially produce trade-offs with provisioning and cultural ecosystem services. Wide buffers with tall, dense vegetation may reduce visual openness, accessibility, and perceived tidiness, limiting cultural services such as recreation, esthetic enjoyment, and public engagement (Cole et al. 2020). Moreover, increasing buffer width reduces the area available for agricultural land use (Liu et al. 2008). Dense and minimally managed vegetation can also hinder provisioning services like well-timed low stocking grazing, which require open, sunlit areas to support pasture growth and allow livestock access (Broom et al. 2013; Osborne and Kovacic 1993). Similarly, maintaining cultural services often demands vegetation thinning, mowing, or the removal of invasive species to ensure safety, visibility, and esthetic appeal. These are management practices that may reduce habitat quality and compromise the effectiveness of pollution retention and erosion control (Cole et al. 2012).

To support multifunctional outcomes, riparian buffer design in the SLRB must move beyond uniform regulatory models and embrace adaptive, site-specific strategies that reflect the basin’s diverse environmental and social contexts. This can be achieved by integrating detailed knowledge of how local hydrological, geomorphological, and vegetative conditions influence the delivery of ecosystem services and shape the associated synergies and trade-offs, enabling the design of buffer zones tailored to optimize multiple service outcomes. Although current characteristics, such as restricting tillage and livestock access, are widely recognized for supporting regulating services, interviewees noted that these measures are applied in a uniform manner that does not account for local variation or acknowledge the interactions between ecosystem services. As a result, current policies potentially overlook trade-offs and synergies to optimize the effectiveness of buffer zones across the basin’s heterogeneous landscapes.

To address this limitation, the governmental action plan could incorporate key contextual variables (such as riparian slope, stream morphology, and vegetation) into buffer planning, using the river basin commission as a platform for integrating local perspectives. Tailoring buffer width and management practices to distinct environmental conditions would enable more targeted service delivery. For instance, the action plan could prioritize erosion control and habitat conservation in the upper basin, nutrient retention and filtration in the mid-basin, and flood regulation and wetland connectivity downstream. By aligning buffer design with local needs and capacities, such a framework would enhance both ecological effectiveness and practical feasibility.

### Enhancing the Co-development of Riparian Buffer Zones in the SLRB

Our findings indicate a perceived disconnect between the formal participatory framework established under Uruguay’s National Water Policy and its implementation in the SLRB. While mechanisms such as the River Basin Commission and constitutional recognition of water as a public good reflect a strong institutional commitment to participatory governance, stakeholders perceive these structures as insufficiently translated into practice. In particular, vertical coordination between national authorities (e.g., the Ministry of Environment) and local actors is seen as limited, raising concerns about the effectiveness of stakeholder engagement in supporting the implementation of riparian buffer zones in the river basin.

The successful co-production of nature-based solutions depends on inclusive stakeholder engagement, aligning diverse perspectives with clear communication, iterative goal-setting, and context-specific indicators to ensure relevance, feasibility, and legitimacy in addressing societal challenges (Van Der Jagt et al. 2023). Shared goals and objectives can be co-defined through participatory processes, reflecting stakeholder priorities and addressing ecological and socio-economic challenges (Pan et al. 2022). Clear communication is essential to bridge perspectives, build trust, and enable adaptive governance processes (Hölscher et al. 2024). Co-selected indicators must align with local realities and resource constraints, ensuring their salience and practicality for guiding and monitoring implementation (Van Der Jagt et al. 2023).

Our results indicate that some of these enabling conditions are present in the implementation of riparian buffer zones in the SLRB, particularly inclusive stakeholder involvement and shared objectives between key actors. However, we also find that other critical determinants, such as effective communication, iterative goal-setting, and context-specific indicators, are perceived as insufficiently addressed. Producers and other local stakeholders report limited engagement with technical authorities, unclear communication about buffer zone regulations, and a lack of opportunity to tailor implementation to farm-specific conditions. These gaps are perceived to be exacerbated by a lack of sustained technical support and weak feedback mechanisms between the local and national levels. As a result, the participatory character of the governmental action plan is seen as lacking, rather than substantive and empowering.

The identified opportunities represent potential strategies to strengthen the implementation of buffer zones in the SLRB by addressing institutional and communicative gaps. Capacity building, ongoing technical assistance, and the integration of suitable environmental management into buffer zone maintenance are crucial for building trust, fostering cooperation, and supporting adaptive, locally tailored solutions (Kiss et al. 2022; Nunes et al. 2021). Participatory monitoring schemes and education efforts targeting producers are especially important to reduce misunderstanding, build legitimacy, and encourage voluntary compliance (Menny et al. 2018; Fisher et al. 2021). Embedding these strategies more deeply into the governmental action plan, through formal inter-ministerial collaboration and stronger alignment between basin-level and farm-level governance, could help overcome implementation barriers and realize the participatory and adaptive ambitions of Uruguay’s water reform process.

## Research Limitations

While we identify promising opportunities to strengthen riparian buffer zone implementation in the SLRB, important limitations remain that merit further investigation to enhance the implementation of these context-specific strategies. First, although we explored stakeholder perceptions, we did not assess engagement modalities like inclusion dynamics, collaboration, or conflict resolution. Process-oriented methods, such as case studies, participatory observation, or network analysis, could clarify how these modalities influence initiatives like forming an educational sub-committee or expanding communication channels. Furthermore, although technical support and participatory monitoring systems show potential for improving accountability and adaptive management, their accessibility, scalability, and long-term feasibility warrant further evaluation in the SLRB. Second, while we highlighted the need for multi-functional buffer zones, we did not examine how to align this with current regulations. Participatory scenario planning or pilot studies could help identify enabling conditions and incentives for regulatory flexibility to provide more desired ecosystem services. Third, we did not explore why some actors fail to follow the governmental action plan, limiting our understanding of behavioral and structural barriers. Behavioral surveys, interviews, or experiments could uncover these drivers. Addressing these gaps is vital for fostering inclusive, adaptive, and effective buffer zone policies in the SLRB and advancing participatory water governance.

## Conclusion

We examined stakeholder perspectives on the implementation and management of riparian buffer zones in the Santa Lucía River Basin, Uruguay. Our findings highlight the critical role of riparian buffers as multifunctional nature-based solutions that offer both recognized and hidden ecological and socioeconomic benefits. However, achieving their full potential requires addressing several key challenges. Local perceptions and desires towards the functioning of buffer zones show that there are gaps in implementation and management of riparian buffer policy. To address these challenges, we recommend: (1) strengthening producer engagement through sustained technical assistance and participatory dialog; (2) improving vertical coordination between national and local actors; (3) integrating multifunctional design principles that balance ecological trade-offs and synergies, and livelihood goals; and (4) embedding monitoring and feedback mechanisms to support adaptive management. These findings highlight the importance of refining participatory governance frameworks to support more context-specific environmental management, helping to better align stakeholder needs with management objectives.

## Supplementary information

Supplementary Materials - Interviews

Supplementary_Materials_Codebook

Supploementary material - interview guides

## Data Availability

Data that support the findings (e.g., the interview transcripts, codebook and interview guide) can be found as supplementary materials.

## Abbreviation

SLRB: Santa Lucía River Basin
