# Supplementary material for: Understanding Stakeholder Perspectives on the Implementation and Management of Riparian Buffer Zones in the Santa Lucía River Basin, Uruguay
Source: Environ Manage. 2025 Jul 26;75(10):2596–613. doi: 10.1007/s00267-025-02230-1 (PMC12457512; doi:10.1007/s00267-025-02230-1)
Supplement: Supplementary file 1 — Supplementary Materials - Interviews [file 267_2025_2230_MOESM1_ESM.docx]

Supplementary material 1: Interviews

**Journal:** Environmental Management

**Title:** Understanding stakeholder perspectives on the implementation and management of riparian buffer zones in the Santa Lucia River Basin, Uruguay

**Authors**: Alfred Paarlberg, Guillermo Sena, Ho Huu Loc, Jannik Schultner

**Corresponding author:**

Name: Alfred Paarlberg ^earth systems and global change, wageningen university and research^

Affiliation address: Wageningen University, Lumen

Building number 100, Droevendaalsesteeg 3

6708 PB Wageningen, The Netherlands

Email: [alfred.paarlberg@wur.nl](mailto:alfred.paarlberg@wur.nl)

ORCID iD: 0009-0003-3269-8060

Contents

[Interview overview 1](#_Toc136959404)

[Interview 1 2](#_Toc136959405)

[Interview 2 5](#_Toc136959406)

[Interview 3 11](#_Toc136959407)

[Interview 4 18](#_Toc136959408)

[Interview 5 27](#_Toc136959409)

[Interview 6 38](#_Toc136959410)

[Interview 7 46](#_Toc136959411)

[Interview 8 52](#_Toc136959412)

[Interview 9 60](#_Toc136959413)

[Interview 10 68](#_Toc136959414)

[Interview 11 75](#_Toc136959415)

[Interview 12 83](#_Toc136959416)

[Interview 13 90](#_Toc136959417)

[Interview 14 103](#_Toc136959418)

[Interview 15 111](#_Toc136959419)

[Interview 16 119](#_Toc136959420)

[Interview 17 126](#_Toc136959421)

[Interview 18 134](#_Toc136959422)

[Interview 19 142](#_Toc136959423)

[Interview 20 148](#_Toc136959424)

[Interview 21 155](#_Toc136959425)

[Interview 22 164](#_Toc136959426)

[Interview 23 173](#_Toc136959427)

[Interview 24 181](#_Toc136959428)

# Interview overview

| *Interview* | *Stakeholder* |
| --- | --- |
| 1 | Local |
| 2 | CEUTA |
| 3 | Scientist |
| 4 | Producer |
| 5 | CNFR |
| 6 | DINAGUA |
| 7 | Scientist |
| 8 | Scientist |
| 9 | Scientist |
| 10 | DINACEA |
| 11 | Producer |
| 12 | DINACEA |
| 13 | Producer |
| 14 | Producer |
| 15 | INIA |
| 16 | Producer |
| 17 | MGAP |
| 18 | Scientist |
| 19 | MGAP |
| 20 | Vida Silvestre |
| 21 | Former-DINOT |
| 22 | Scientist |
| 23 | Scientist |
| 24 | CAF |

# Interview 1

**Interviewee: Local Date: 23-02-2023**

Interviewer 2: I am working on riparian buffers in the fields to prevent contamination of water courses from agriculture and the application of herbicides and pesticides. Together with Alfred, we are going to talk to different stakeholders. We are going to talk with producers. For instance, we have people from the agricultural ministry give us their vision of the subject. We do this because each stakeholder has a different vision, and with those different visions, some of them are going to be opposed, and we are trying to synthesise what is happening and what problems may arise regarding these buffers.

Interviewee: I have a son who can also give you a more scientific vision because he did a degree in environmental sciences; to be precise, his thesis was about water control in the Santa Lucia River. He did it in Las Brujas, in another area that I don't remember, and here at the mouth of the river (the Santa Lucia River). If it's good for you until next Tuesday, he's free. Well, maybe I'll write him, and we'll see if we get together. These things are an opportunity for us to get to know each other and stay in touch. And now he's on vacation until next Tuesday.

Interviewer 2: After that, if we want, we can interview his son.

Interviewer 1: What do you do for a living? Interviewee: I’m retired. I worked in the commercial sector in Montevideo, in Valley Park.

Interviewer 1: Do you know of the Santa Lucia River Action Plan?

Interviewee: Yeah. I'm a little aware; I'm in an organisation to preserve the native forests because they are very deteriorated.

Interviewer 1: Clear. The Action Plan introduced the use of riparian buffer zones along the Santa Lucia River. What is your role in water management in the area?

Interviewer 2: Oh no, that’s not relevant. He advocates more for protected areas. I think that the riparian buffer zones are not a topic that we can talk much about. So the questions here are a bit irrelevant. Could you tell us a little about the history here? How have you lived it, regarding conservation issues?

Interviewee: I started coming here in my early 20s. My father had bought that house, and he came every two months. In other words, I was seeing a bit of all the problems in the area and their growth. A very important topic in the subject of protected natural areas The city grew very rapidly. The same thing happened on the Gold Coast. It has grown very rapidly, and therefore there is no good sanitation. Structures that really should have been taken care of have not been taken care of. For instance, this house is in a flood zone. I looked it up, and I think we are 3 metres above sea level, here in this place where we are. And a lot of things, like the issue of the native forests, have not been taken care of. For example, there are many people who need firewood, and there is a lot of tree felling going on. In other words, there are a lot of woods on the other side of the river that have been greatly damaged in the entire area.

Interviewer 2: How did the deterioration of those woods go? Because that interests us. It is against the river. It is our research focus.

Interviewee: They (the government) had to construct more roads and clear the nature park. The park in Santiago Vasquez was cut in two so that a new road could pass through. There were native and planted trees because there were many eucalyptus trees, of which some still remain. And further up, if you look, there's little native forest left.

Interviewer 2: Was there more, or do you have any idea if there was more (native forests, red)?

Interviewee: No. I think there were no more. What was here has been felled, and there are also many people who work collecting reeds. Apparently, there are associations to take care of that. They have been taught to work the reed, and there are many people who dedicate themselves to removing reeds to make mats. In other words, basically, there are many small businesses in this area that make reed mats. Another great problem is that, especially in spring, the number of fishermen increases to fish corvina (croaker). Yes, they catch them before they lay their eggs, and they are really preying on them, trying to catch them. It really is something negative.

Interviewer 1: What is your interest in and your experience with protecting the environment? Interviewee: Forest. My interest is in the fact that it would be necessary to reforest and help by replanting the forest. The entire forest that was in front of the coast was degraded because of the need for firewood. And one of the measures that we plan to take is to replant the area with native trees. On the coast of the Santa Lucia River. In that part of the coast of the Santa Lucia River, right there, two kilometres from the river.

Interviewer 1: Why is it important for you?

Interviewee: Due to the fact that it was a place for birds. In other words, there are many migratory birds in the area. Furthermore, it is also for the containment of erosion. And another thing that we're trying to avoid is that the inhabitants throw away garbage (littering). But the big problem is that there is no one to regulate these issues. There are signs prohibiting littering, but there is no control at the government level. I always wanted to do a ranger course. I was trying out in Arrayanes (UTU), but I'm missing two subjects from the fifth and two from the sixth degree. What I could do, with the fourth finished, is environmental control monitoring. Why do I do it? San José, for example, does not have park rangers. The first thing I did after retiring was to look at it (the courses for park rangers), because I experienced everything. Because we are going to visit the last block, I will show you the area. The natural reserve is called Penino Beach Nature Reserve. And why is this important? Why is it a protected area? It is a protected area for migratory birds. I mean, it is a wetland that houses many important migratory birds.

Interviewer 2: Are there forests? No, these are low areas. This area here is floodable, and no forest grows here. There are channels, and there is an embankment to prevent the water from passing through the area. It's in the same condition as the rest; it's already worn out (degraded, red).

Interviewer 2: What organisations are dedicated to the protection of the natural reserve? The national directorate of protected areas In other words, at the governmental level, the environment ministry What I see in relation to the other protected areas of Uruguay is that this one (Penino Beach Nature Reserve, red) has a problem with the number of people living here.

Interviewer 2: And have you had any flooding problems lately? Interviewee: Not lately. The last flood... I think my brother was 10 years old; today he is 49. He is the one who was born here, from a second marriage. Yes, it was about 40 years ago. The water passed through the channels and came to the edge of the door. The people who lived there had to move their furniture out. Running water has the power to control We had to find that out.

Interviewer 2: We are studying the riverine forest and the riparian buffers. They have some functions. In addition to pollution contamination from agriculture, they may also have functions that have to do with reducing the amount of water that enters the river (water retention, red). This could be important if there are floods. We could say that, currently, the buffer zones are not fulfilling their functions entirely. Therefore, have you experienced any flooding lately? Interviewee: No, not lately. The last one was 40 years ago.

Interviewer 1: Okay, perfect.

Interviewee: I set up a water purifier with my son when we moved, in the back (of the garden, red).

Interviewer 2: How?

Interviewee: I will show you. We did it with the water drain. My son built a small pool where the drain ends, and it uses pebbles, sand, soil, and mulch. The water that goes in comes out purer. When the pipes broke, we stopped using them. The drain went that way, but we had to remove it. Now it acts as a greenhouse.

Interviewer 2: It's interesting. Regarding the stakeholders, do you think the fishermen, for example, can harm the ecosystem? Or those who gather reeds? Is there any other group that interacts with the ecosystem that you remember being important?

Interviewee: No, at the school and high school level, walks are made and information is given for people to try to protect the marshes. My partner's cousin, who is a teacher, works for the municipality in the library. They take kids to school, already high school kids, to teach them about the marshes.

Interviewer 1: Okay. Generally, how do you experience climate change in the Santa Lucia River Basin? Do you think the temperature changes or the precipitation changes, for example?

Interviewee: Yes, climate change I think people do not give it the importance it deserves. Either due to forest degradation or the burning of garbage. In other words, all of this is damaging the ecosystem, and I think people have not become aware of the issue of climate change.

Interviewer 2: And do you perceive any differences in this area in the last 10, 20, or 40 years?

Interviewee: No, it's just that the big problem is that there was no information before. Back then, garbage was burned without a second thought. And now there is information, but people don’t help. They don’t collaborate.

Interviewer 1: Okay. Do you have any questions?

Interviewee: No. Maybe we can take a walk and look around.

*Interviewer 1 is the student, and interviewer 2 is the supervisor.*

# Interview 2

**Interviewee: CEUTA            Date: 24-02-2023**

Interviewer 1: This interview is about riparian buffers. These buffers are strips of vegetation along the watercourses of the Santa Lucia River. Their purpose is to capture and retain nutrients from agricultural activities to improve water quality. Certain land uses are prohibited in these buffer zones, like access for cattle, for example. First, what do you do for a living?

Interviewee: I am the executive secretary of CEUTA. So, I have been working for many years at the Uruguayan Centre of Appropriate Technologies (CEUTA). I have agronomic training, but I did not graduate in it. I have a lot of empirical training in the field, working with farmers. Especially from agroecological and organic producers. Also with vegetable gardeners in peri-urban areas. I have worked a lot on the implementation and dissemination of appropriate technologies, from all types of ecological sanitation to more productive techniques and disease control in an ecological way. I have also worked on co-innovation projects, in which we do participatory research on farms. We (CEUTA, red) have done so for many years. On topics such as green manures, natural pest control, and ecological soil management. And then I have also worked as a consultant in the technological sector, more in terms of advocacy in policy analysis and research. And I have done work related to devising recommendations for public policies within the profile of what can be called policies that have to do with agroecology. Within this approach to agroecology, in fact, we (CEUTA, red) include the work with buffer zones. That is to say, we do not separate it (riparian buffers, red) as a separate thing; it is not something that is an implementation of good practise. But we see it as a gateway, a further management tool for an agroecological transition at the farm level. We envision that the producer should be integrated into the implementation process of these buffers. Not only to make the buffer zone work but to start integrating the producer into the design and into the management. We think that this is the first step to an agroecological transition, which obviously can take many, many years. Or it can be more stimulated and catalysed with policy support, advice, training, and so on. Sorry, you asked me what I was doing, and well, I jumped up and spilled the whole story. But well, basically, I have dedicated myself to these things, and I work very much from a civil society perspective. But I do so in a role that has articulation between more academic and more technical viewpoints, between producers, populations, and actors in some way.

Interviewer 1: Of course. Then, what are your activities and responsibilities in the process of the implementation and management of riparian buffer zones?

Interviewee: Let's see, it's very subjective, but in reality, I consider that the role of CEUTA has been, on the one hand, to establish and start pilot management experiments. And on the other hand, to disseminate and showcase these pilot experiments in order to have, in some way, a discussion on the subject with other civil society and public actors, like, for instance, the Ministry of Finance. We (CEUTA, red) propose ways and means to scale up these pilot experiments, amplify the effect of what we consider to be positive, and also stimulate further research, right? And I say "stimulate" because that is not CEUTA’s role, but it is very clear to us that it is something that has to be done, to have a lot of research to back up the actions that can be taken. But, as clearly seen, it is a virtuous circle that feeds back on itself. It is possible to establish, let's say, monitoring projects without having, from our point of view, strong implementation projects that feed back into each other. And neither can you have too much growth in the implementation without having scientific bases that corroborate what you are doing or that give clues as to where to go and where not to go. Those two things have to be linked. And the third thing, which CEUTA is also trying to do, is part of communication and dissemination to influence public opinion. But CEUTA is a very small organisation; we have to be clear about that. We believe that it is essential that public opinion be sensitised and take part in these actions as well. In other words, producers, that is, people in the cities, and farmers This is the key to raising awareness: to carry out this process of communicating what is being done in order to obtain positive feedback from the critical mass that can be supportive. There is no other way than to turn to these actions, linked to the buffer zones, with multiple responsibilities, multiple roles, and multiple contributions. From public and private sector producers, it has to have those three components and support. It is not enough that only one of them is strong. Isn't it?

Interviewer 1: Right. These buffer zones can be designed in many different ways. For example, using or promoting different types of ground cover and management strategies. What features do you think these buffer zones currently have in the Santa Lucia River Basin?

Interviewee: Currently, I think what's implemented is a very zone-based scheme of riparian buffer zones in which an area is delimited and exempt from human management. I don't know if there is an issue with mowing in the end, but basically, they (the riparian buffers, red) are surfaces that are protected from soil management. Then, there (on the buffer, in red), it works as a bio-filter, trapping sediments and active particles from agricultural activity. It works by slowing down the velocity of water and, therefore, increasing its capacity to retain sediment, preventing all these particles from agricultural and livestock activities, as well as organic matter, from ending up in bodies of water. And therefore preventing the alteration of ecological relationships and the functionality of these water bodies. Now, for me, it is important that when we were in the conceptual phase of riparian buffer zones, we started with this idea or with this definition of the buffer zone. Then we started talking about integrating the buffer zones with the bordering patches (of land, red)  in the agricultural property, which is linked to the buffer zone. And then expand them. But when talking about it, we said no. In reality, the working cell (land dedicated to the riparian buffer, red), the buffer zone work cell, has to be adjusted to the farm scale. And in reality, the zone that borders the water is simply the last limit of the buffer zone (i.e., the buffer should be integrated into the farm). When that was clear, we had to change the concept, and we started working on a practical level. One thing is to come up with a conception where only the buffer zones are going to work and where there are going to be other impacts in the area bordering the watercourse. And another thing is to come up with a conception of a system where the system is in the total jurisdiction of the producer. The area that we consider to be a buffer zone according to this definition is part of the agro-ecosystem from a methodological, analytical, and scientific perspective.

Interviewer 1: What characteristics support these functions? Think of the structural characteristics of the system.

Interviewee: I think there is more to buffer zones, but since you opened the topic, at first we had the idea of maintaining vegetative strips where the ground is not disturbed on one side. But also in these strips, we proposed, as far as possible, to enrich these strips with native flora that is strongly adapted to local circumstances and that has the capacity to maintain a deep rooting depth and also increase the volume of roots per square metre. Also, it could generate multiple functions, not only enriching its capacity to filter and slow down sediments and trap nutrients but also operating the buffer zone system in multiple ways to bring in pollinators, fix nitrogen, and generate fruit (i.e., enriching the biodiversity of the buffer zone). It is important that the floristic origin of the species be linked with the basin in which the work is being done. Then, in addition, at one point we also worked on the possibility of enriching the vegetation to increase the density of individuals per square metre; especially in terms of that, they could have the purpose of producing food as a fodder bank. For example, by using a combine harvester when going into harvesting at some point or letting cattle enter the buffer to do intermittent grazing. This is important to create the possibility that the farmer could see this buffer zone as something positive for the production system. But also so that it would provide positive feedback. For example, the generation of above-ground (red)) biomass and the generation of root biomass will improve the performance of the system. And finally, the incorporation of buffers in the places where the land was altered Marcel Ashkar, who is a geographer, also works on these areas with geographic information systems and with models of the evolution of woody vegetation in watersheds. That was the first work we did, and based on this work, we defined which zones were reforested with native woody species and which areas were originally more marshes or grasslands. Where applicable, we also included the enrichment with native woody species that were more hydrophilic with a profile closer to the watercourse to support the riverbank.

Interviewer 2: I have a question here to follow up on. I would like to ask you if you spoke about buffer zones with productive uses. Basically, mainly in the implantation of higher densities of grasses or plants that have the ecosystem function of retaining nutrients but also the function of providing food or fodder with certain management parameters to livestock farmers, Do you think you can sustain the implementation of such a buffer zone? For example, imagine that it is regulated by the government. How would it be possible to maintain these regulations? You can go in to graze sporadically twice a year; do you think it can be sustained by the producers' own will, or is it necessary to set up a control mechanism? And what would be such a mechanism?

Interviewee: I don't believe that goodwill has proven to be effective in these situations. I wouldn't leave it up to goodwill. Because of goodwill, we have a percentage of people who will do it, and the rest will not, causing counterproductive effects for both the ecological system as well as for the human action that is being undertaken to restore the native forests (the riparian buffers, red). Because if one producer sees that another producer is doing it better, that's where it feeds back to the environment, doesn't it? Then no, that's not enough. That's why we started to play a little bit with it. Because we could propose setting the boundaries of the buffer zone itself. That is to say, we could determine where the boundaries are established for where you can till the soil. Our recommendation could very well be to establish meadows, while in fact there could be other options, for instance, cattle ranch land to enrich pastures and maintain perennial meadows that were already pasturing. These pastures could, for instance, be enhanced with legumes. And with proper soil analysis, nutrients could be worked into the soil (through nitrogen fixation of the legumes, red). There is also the issue of iron oxide (in the ground, red) in the basin and the whole question of phosphorus trapping to the extent necessary. Summarised, what is important to do is arrive at an available package of tools for buffer implementation. My advice would be to have an initial phase of 3 or 4 years with agroecological soil management with this approach. I believe that this is the beginning of a virtuous circle of the production of more biomass, generating better animal welfare, and producing better results, better productivity, and better yields. I am sure this is achievable, but it has to be implemented on a case-by-case basis, according to the shape of the property, the location of the property, and the socioeconomic level of the property. Maintaining a buffer zone can be very detrimental to a family farmer. And that is where the possibility of intermittent grazing comes up and how it would be regulated. One possibility would be that the producer would have to notify the municipality each time they make grazing, or whatever the local authority is. And there could be a control system that could be subject to inspection. But it would seem that it could be a possibility to have that limited number of times you can do it on an annual basis. But really, that's as far as we got, and we didn't delve into that. And obviously, it requires multi-stakeholder action to maintain this at the basin level. This is like everything else; I believe that the critical phase and investment would be about the first 10 years, where the local authorities, the ministerial authorities, the organisations, and the university have to be very much in a very strong joint effort with the producers. Of course, I believe that after certain systems are established, such as certain management systems and cultures, producers take over and work more on their own. But those first years are kill or be killed.

Interviewer 2: The answer is very clear. It is good to take this vision into account and these ideas that have also emerged from your work when confronted with the problem in the field.

Interviewee: But there is no doubt that it was a crossroads. We were talking about the rural development of the municipality with the people from the ministry. And on the farm with the producers, there were many more questions than answers, right? But even very willing producers keep asking questions. With other participants, surely you will find different perspectives with multiple contacts and different actors, and you will come up with new things as well.

Interviewer 2: How do we continue?

Interviewer 1: Before, we asked you what functions the buffer zones have. Following up on that, what functions do you think these buffers should have, ideally?

Interviewee: Well, a little bit of what I was saying I think it should include possibilities for agroecological management linked to the buffer zones that would be productive. It is a battery of agroecological practises that, together, can even improve the buffer zone, producing co-benefits for the productive system. This is ideal since it is necessary to agree on what that buffer zone is. We have a clear proposal, which is largely supported by the environmental mystery. But there may be others, or there may be different functions that might be interesting.

Interviewer 2: So from what you are saying, they should have a nutrient retention function. Furthermore, they could provide an income-generating function or a productive function. Maybe a third one is that there is multifunctionality that includes other ecosystem functions such as pollination. For example, they could improve or enhance biodiversity and pollen production in the area of the buffer zone. And if you were to value these three functions from one to four, from least important to most important, how would you rate them? If you had to give each one to four, which was the first one, nutrient retention?

Interviewee: Let's see, nutrient retention would be a 4. It is obviously the most important. And then I would put... Others can be 4, too, right? Let's see if we add them up, and if they fit in with my vision of the buffer zone that has to incorporate boundary management and a systemic vision of the property, I would also add three or four to the production function. And then I could put a three next to the enrichment of other ecosystem functions.

Interviewee 1: The following questions are about the implementation of these different characteristics. What would be the benefits of these additions to the buffer zones?

Interviewee: Well, for economic benefits, with the appropriation of the buffer zone, the farmer is going to be much more involved. They could become more in favour of the implementation of buffer zones. And then there is the enrichment of other ecosystem functions; the producers would have to do training (leading to more skilled producers, etc.), but it also works for the improvement of the performance of the agricultural systems. An area with enriched biodiversity and pollen production will enhance certain functions of agricultural and livestock systems. As I said before, this enriched biodiversity has a larger root volume capacity. And it has more biological activity, which is going to improve the ecological status of the area.

Interviewer 1: Perfect. And what obstacles do you think will be encountered with the implementation of these changes to the zones?

Interviewee: There are technical obstacles to scientific information in this area. There is a need for locally adapted technical obstacles because we do not have proven technologies, and some of these changes can be costly. An investment needs to be made in time, energy, resources, and training. The process is slow, and you are not going to know the results immediately. A certain level of faith is needed to carry out this process. You have to believe that there is a joint act of involvement by everyone.

Interviewer 1: And how do you think you can overcome these obstacles? Or what possible solutions do you think we can implement to overcome these obstacles?

Interviewee: Let's see, I believe that you simply have to follow the path of experimenting with pilot projects and take the small steps that can be taken to do this research and let it permeate into public policy. That is to say, I had a call last week with the Ministry of Environment to reemphasize our ideas. I stressed the importance of the upscaling of a pilot project to make 50 to 60 areas eligible for experimenting with productive buffer zones. This experiment is based on a project in Aguas de Quito, Ecuador, where there is the creation of a trust (financial trust fund, red) that is in charge of the care of the basin that provides drinking water to Quito. This trust receives a portion of the water tax paid by the residents. From this trust, restoration, conservation, and agroecological management of the watershed can be managed. When I proposed this, OSE (the water treatment company) and the CAF (the bank) laughed in my face. But those things can change too.

Interviewer 1: Okay. What is your interpretation of the impact of climate change on the riparian buffer zones? And how do you experience climate change in the Santa Lucia River basin?

Interviewee: In truth, I mean, I don't know. I can give you my ideas, but they are not clear to me. But since the time when we were more in contact with each other, an increase in extreme events of rain and lack of rainfall has occurred. Thus, there has been an increase in torrential rains, very concentrated in time, and an increase in droughts. I think they are more frequent than before as a result of anthropogenic emissions. Some observations indicate that the peak and low temperatures are more random and frequent. We always used to have one short summer and one short winter, and now it happens that winter has more low temperatures than before. And that the summer gets longer and warmer. Those are the most noticeable effects. And regarding the effect of these changes on the performance of the buffer zones, I am from the agroecological school to the extent that there is more application of agroecological principles. We can expect that the systems (with buffers implemented, red) will be more resilient to climate change, by which I mean ecological soil management will be better able to withstand the effects of climate change. And with organic material structurally covering the soil, the system will be more resistant, whether in terms of biodiversity, slowing down wind speeds, or having different productive elements that support the system's productivity. Obviously, the effect of climate change makes it increasingly difficult for agriculture and livestock farming in general, increasing the pressure on the agricultural production system. The system is already the recipient of multiple pressures. We can see the buffer zone as a precautionary zone in the Santa Lucia basin. On the other hand, we have seen that the producers felt overwhelmed by the precautionary measures and felt that they were under attack. On the other hand, others see it as an opportunity to support a virtuous circle affecting the productivity of their land based on nature-based production. Of course, we have to support them so that this is seen as a good development (the introduction of buffers, red). It can have beneficial effects and be sustained over time, and in the end, I believe that the effects of climate change on areas would be different if the changes I proposed in the previous question were implemented. I refer to conceiving the entirety of the agroecosystem and the benefits it can have, such as gradually enriching the soil's organic matter and capturing water better. If there is more water available in the soil and more is trapped for longer, the organic matter will become richer. And therefore, the system will become more resistant to droughts or water deficits.

Interviewer 1: Clear. I have no other questions. And you?

Interviewer 2: No, I think it was quite well compiled. The vision and experience of CEUTA in the interview were clear. I don't have any other questions.

Interviewee: Great. Well, thank you very much. And tell me very briefly how the work continues.

Interviewer 1: This was the interview, and the recording has ended.

*Interviewer 1 is the student, and interviewer 2 is the supervisor.*

# Interview 3

**Interviewee: Scientist        Date: 28-02-2023**

Interviewer 1: The riparian buffer zones of the Santa Lucia River Basin are strips of vegetation along the watercourses of the river. Their purpose is to capture and retain nutrients from agricultural activities to improve water quality. Buffer zones prohibit certain land uses and the access of cattle, for example. What do you do for a living? What is your job?

Interviewee: My work… I have a Ph.D. in biological sciences, I am an assistant professor at the Faculty of Science (Universidad de la Republica, red), and my work is focused on limnology, which is the study of inland waters and the functioning and structure of inland waters. But in particular, within limnology, I study the dynamics of inland waters. I study nutrient dynamics and how nutrients behave, mainly nitrogen and phosphorus, in aquatic systems as a function of other environmental variables. I also focus on how environmental variables interact with these nutrients. Think of the human anthropogenic effect on these dynamics. What are the effects on aquatic ecosystems? And within these effects, one of the main ones we are studying is the blooms of cyanobacteria, which is a symptom that has been widely studied at a global level, is difficult to solve or control, and generates health problems due to the toxicity of these organisms. Not only the effect on people at the level of recreational use of watercourses but also the use of the water for watercourses and also the use of the water for drinking water. So, they are organisms that are easily visible; that is to say, they also have a very big social impact when they are seen. It is not a type of pollution such as bacteria, heavy metals, or other types of pollutants that are invisible and that are only measurable at the laboratory level. So, on the one hand, they are organisms that help in some way to give away what's going on in the aquatic ecosystem, as well as being harmful, obviously.

Interviewer 1: And did you participate in the creation of the action plan for the Santa Lucia River Basin? No, we only contribute at the level of meetings and commissions, but the action plan was elaborated directly by DINAMA, as far as I understand, by their technicians.

Interviewer 2: And what role did you have in these meetings of the commission?

Interviewee: I no longer participate in the Santa Lucia River Basin commission. There is a delegate from the Faculty of Science, now it's Ismael Díaz, the current one. Before it was Patricia, I don't have the surname, and with Guillermo Chalar we were the delegates from the Faculty of Science, until approximately 2019. Then there was a break due to the pandemic, and they also resumed with the changes of authorities in the Ministry. But until then it was our participation.

Interviewer 1: Of course, the action plan for the Santa Lucia River Basin introduced the use of buffer zones in the basin. The next questions are about your understanding and role in the design and implementation of these buffer zones. What is your interest in the design and management of buffer zones?

Interviewee: Yes, I remember that at the beginning of the commissions, a specific sub-commission was formed for buffer zones that met about three or four times. It was a specific commission to deal with the particular issue. When it was announced, it was extremely novel for Uruguay that these buffer zones were being promoted and implemented. It was news that was very well received by colleagues. But once the news was received, they began to see criticism from one side within the academy and from the other side from the agricultural sector. So the main question that arose here and there was: how effective are these buffer zones? We were proposing to expand their range or their size while the producers were proposing to say, "Well, let's define to what extent they are useful and to what extent they are not useful. To what extent do we access that extension, and up to what point is it not useful at all? So what was missing at that time were studies and evaluations. What we and our colleague Gilles Aguichet-Morsellado were suggesting was that, in many cases, the flood zones were not being included. That is to say that the buffer zone in many cases, in which it is obviously very complex to map a zone of this type in a flood zone, was not included. So we proposed that the buffer zone had to be outside the flood zone and that the flood zone was a zone of its own.

Interviewer 2: The flood zone is the alveo, which is what it's called, isn't it? As far as the water reaches.

Interviewee: Yes, and then once we had this, it was seen very favourably that the buffers at the reservoirs should be 100 metres long, and then there were areas of 20 metres so that for a part of the reservoirs, the entry of livestock was prevented. And that's when the issue of to what extent this is going to be fulfilled came up, right? On the one hand, I was beginning to evaluate its usefulness and that we had to wait for the vegetation area to recover, right? For that recovery to be active, it must be significant. And on the other hand, to see to what extent the regulations were being complied with, So, a student of mine, Hernán Holano, worked for a while with surveyors at DINAMA, and they carried out inspections of compliance. So, they realised that this was obviously very difficult to sustain and that it implied a continuous review and control of whether or not they (the producers) were in compliance. This data started to be uploaded to the website of the environmental observatory, and if you, obviously, have seen it, I don't know to what extent it is up-to-date. I don't think it is up-to-date. There were areas that were not complied with and areas that were. So there, as a buffer zone, it starts to lose its meaning, doesn't it? That there are so many gaps between its implementation in the field and as a zone, it is too permeable. From these discussions, while a group of people focused on the buffer’s cover, others started to have an agreement with DINAMA to empirically evaluate the functioning of a buffer zone in the Paso Severino reservoir. We began to discuss in that commission what was lacking or missing here and how to continue advancing towards the smaller watercourses. And that's where it started. DINAMA, at that time, received it very well. We were also surprised because they told us, "Well, this is a first step; let's take the second step when the waters calm down a bit (the political discussion on the buffers, red). Because of the resistance that this (the implementation of buffers, red) had, they wanted to move forward on the second generation of measures aimed at the smaller streams and creeks. So, the second generation was going to increase the buffer surface, let's say, or buffer zone's functioning in a much more significant way. And on the other hand, we wanted to make progress on land use in the headwaters, right? Well, when that was then left in the proposals, there was a lot of resistance from the Ministry of Agriculture. And until then, I don't know what happened to the implementation of the second generation of measures. Perhaps you know about that. I have consulted with someone from the basin commission about whether the control of the buffer zone continued after the change of authorities. From what they told me, there has been no intensive monitoring of these places, nor have there been any fines. No fines, no warnings—I don't know. I have the feeling that these buffer zones and their control have been abandoned. And the current government's motto of responsible freedom was left a little bit behind. Each producer makes their own buffer zone if they want to or not. This at one point surprised us because, before it was implemented, it was decreed in the Santa Lucia River Action Plan. According to satellite images, there were some plots in which producers had done it, some in which nobody had done it, and nobody told them anything. There are all sorts of people, obviously, as you know from the production side. There are people who are very responsible and others who are not so responsible.

Interviewer 2: Alfred, do you understand? He talks about the meeting between the Minister of Environment and the Ministry of Agriculture and then about the second generation of measures that the environmental authority was able to advance, which have not been implemented.

Interviewer 1: That's perfect. Thank you. These buffer zones can be designed in many different ways, for example, using or promoting different types of land use, and different soil and management strategies. So, there are different ways of defining these buffer zones. What functions do you think the buffer zones currently have in the Santa Lucia River Basin? How would you define them? Or what functions do the buffer zones serve for you?

Interviewee: For me, and also from what I've read, a buffer zone has to be adjusted to the local conditions. It has to be evaluated according to the type of basin, the type of slopes, the type of runoff, and the topography of the terrain. But it would need to be a sufficient area to retain a significant percentage of the nutrients produced by agriculture in the basin and also suspended particles. In other words, both should be retained in a way that is neither absolute nor total but significant, with a reduction. This implies a specific design, shall we say? At the Santa Lucia basin level, what I find has been a good attempt, but it has been a bad implementation that has fallen by the wayside. It has been abandoned, at least in my opinion. The implementation of buffers is a continuous advance in the improvement of these areas because they require the recovery of the area and cover an area until a retention target is reached.

Interviewee: As you said, there are several types of buffer zone designs. This is not only the dimensions, but it can also be the type of cultivation that is done on another strip above the buffer zone, which in many cases in the U.S. is unfertilized areas that are harvested to remove nutrients from that site. In some buffer zones, the vegetation is actively reclaimed; in others, it is left to recover. There are experiences, for example, in the municipality of Canelones in the Laguna del Cisne basin where measures have been taken in the stream. This includes the buffer zone sub-commissions and, in some cases, showed progress a little beyond what had been proposed for the Santa Lucia River, with certain criteria in low-lying areas where there is water accumulation and also with the planting of native fruit trees. That is to say, there were several alternatives that were used up until that time in another basin. But the main objective is to keep the intensive agricultural activity away from the watercourse and to make a vegetative barrier so that everything that runs off the surface passes through that vegetation barrier. Yes, I have seen, for example, a gully that cuts through that vegetation barrier and enters the watercourse. So, what I saw of the Santa Lucia river basin was that it was a buffer zone with many punctual inflows (causing point sources, red). So, of course, the fear was that when the Ministry of Agriculture asked for results, the results were not going to be very significant. In regards to the main characteristics of buffers, that would be plant density. Also, one of the characteristics of the thesis of my student was the type of rooting that was in that area. They saw that rooting depth caused the entry of the water from the stream at depth into the soil, whereas the more shrubby or grassy areas retained a lot of suspended particles. So it is possible to think about management beyond natural recovery, as had also been proposed, to have transitions of certain types of vegetation. One thing that we had also discussed with Carlos Perdomo was the overloading of nutrients in the soils, which was also very difficult to control.

Interviewer 1: According to the situation you just described, what function do you think buffers should have, ideally?

Interviewee: It would be ideal if that buffer zone covered all the margins of the watercourses that enter the system. In addition to covering a certain margin, it needs to be defined topographically as well, which also includes the flood areas. This way, the buffer zone should not be within the flooding area but should be in the dry zone, which receives the surface runoff from the land (e.g., on the transition between the dry zone and the floodplain, red). To make this happen, more information on what the river order limit would be, the size of the river, and the size of the buffer zone is needed. Similarly, there are regulations for the use of agrochemicals. That could be applied directly to a buffer area. These regulations, at least, apply at 10 metres to any watercourse and are monitored by satellite with GPS. That is to say, there began to be much more detailed control and monitoring. And the next thing is a practical use to improve the quality of the water in the river itself, or of the water intake. And the other utility is biodiversity, right? Trying to recover the biodiversity of not only the river itself but of a margin of flooding area that is in continuous contact with the water And that also gives the system a certain stability, doesn't it? So, it also functions as a biological corridor, that is to say, to recover the system from an environmental point of view, from an environmental health point of view. And one thing that was raised and that we see as something that has not been directly supported is that it has not been possible to achieve buffers in a watercourse used to make water drinkable, which is a great argument, isn't it? In other words, let's stabilise or  recover a system from an environmental point of view to produce drinking water of the best possible quality. That is to say, recent developments have been such that this has been abandoned.

Interviewer 2: Here, you mentioned three functions. One was biodiversity, which was the last one. And then there is one about water quality. Nutrient retention and nutrient uptake would be one, and the other would be the absorption of suspended particles or erosion. Furthermore, biodiversity or the environmental health of the river.

Interviewee: Right.

Interviewer 2: If you were to rate the importance of each of those functions from 1 to 4, could you do that for biodiversity, retention of nutrients, or the biodiversity of ecosystem health?

Interviewee: Yes, I think on a practical level, the retention of nutrients and pesticides would be the most important for the water quality of the river. So those are 4. Otherwise, the functioning of the ecosystem as a connected system would be second, let's say. So that one is a 3. That goes more to the conservation of the system or the ecosystem than to a practical end, right? But that is important in terms of the sustainability of the system.

Interviewer 1: Perfect. The next questions are about the implementation of these different functions and features. What do you think would be the benefits of these changes or additions to the buffer zones?

Interviewer 2: You talked there about expanding the area to cover all the watercourses with buffer zones, including smaller watercourses, and adapting them. I think the answer is clear, but it's good that you also say, "What are the benefits or changes that these recommendations that you highlight would bring?

Interviewee: Of course, I'm making a comparison here. It’s a bit silly, but it's like taking antibiotics. One thing hurts you—let's say it harms you—and you take antibiotics. It does nothing to you if you take too little; it doesn't achieve the objective. And, well, the other thing is, yes, if it works, it might hurt you a little bit, but it does achieve the objective, right? The objective is to control bacteria. It can only be effective when taken properly. In this case, what we were afraid of was that the buffer zones would be left with half non-compliance and the other half compliance (of landholders, red). So it's really the incomplete implementation that would have been reflected in inconsistent and unencouraging results. This has led to the pressures that led to the abandonment of the buffers because they (landholders, red.) considered them to be useless and were losing land to cultivate. So, by not really moving towards more complete and controlled areas, we were going to be left at a turning point, and we're going to go backward.

Interviewer 2: That comparison is very good. I liked it. It's like an antibiotic. I understand it's very good. Your speech is very clear.

Interviewee: Well, thank you.

Interviewer 1: What obstacles do you think will be encountered when it comes to implementing these changes to the buffer zones? For example, in terms of regulation, management, or any other process that you think is relevant.

Interviewee: Right. The obstacle is that there has to be active work on the Ministry's part, control, and a system that supports this work from a legal point of view, right? For those who do not comply. If there is no support of that kind at the legal level, I think it does not work completely. And that implies inspection in the field, right? Monitoring noncompliance in the field of buffer zones with satellite monitoring is very easy nowadays. So this requires active work with people working behind it, right? At the moment, I don't know if work is being carried out.

Interviewer 2: Certainly, do you anticipate any discrimination or prejudice based on the unique functions we discussed earlier?

Interviewee: Yes, that's right. It's like a chain reaction, isn't it? The one who didn't perform, who never complied, suffered no harm. Then, the next person sees this and decides not to do anything, assuming that they don't have to, and so begins the chain of non-compliance. It's an issue that's often neglected and overlooked, and it's something that we as producers must work hard on to combat, often by word of mouth. One such issue that concerns our colleagues is the Neptuno project, which involves obtaining drinking water from a different source, potentially leading to the progressive abandonment of Santa Lucia as a primary source. This is something we need to address.

Interviewer 1: How do you think we can overcome these obstacles? What possible solutions can we develop?

Interviewee: One obstacle is the Neptuno project, which diverts attention away from the Santa Lucia River and generates a shift in the interests of the River Basin Commission. It's an issue that needs to be addressed, as it has stopped discussions about the Santa Lucia River. The Neptuno project is being studied by another basin, possibly the River Plate Basin Commission or the Maritime Basin, but it seeks to take water from the Rio de la Plata, prepare it for consumption, and store it in a reservoir. This diverts attention from the Santa Lucia River and could lead to its being forgotten.

Interviewer 1: And how do you experience climate change in the Santa Lucia River basin? Think of changes in temperature, precipitation, droughts, or floods.

Interviewee: Regarding climate change in the Santa Lucia River Basin, it's difficult to assess it objectively. Climate variability, influenced by the Pacific Ocean, temperatures, and other variables, has led to periods of flooding or drought. Currently, we're experiencing a phase of three years of intense drought, causing the Santa Lucia River to reach a minimum flow, relying only on reservoir reserves. And therefore, the water of the Santa Lucia River is not impacted much by diffuse source impacts from the basin. It receives more punctual impacts (inflow) due to the low rainfall, which is why the diffuse impacts are very low.

Interviewer 2: What do you think would work to control these impacts?

Interviewee: Well, it wouldn't work during the implementation of the buffer zone, but it would be very important at the onset of precipitation. This year, we're moving from a drought phase to a neutral phase, and the first precipitations would generate a huge inflow of substances in dilution and suspended particles. So, the diffuse effect would be very large at the beginning, and the erosion would be very high. Despite being less effective during this whole period of drought, the buffer zone would be essential at the onset of rainfall to control the inflow.

Interviewer 2: How do you think these changes will affect the performance of the buffer zones in the Santa Lucia river basin, especially since it's a natural vegetation system?

Interviewee: The buffer zones are not systems that can function in a stable way. They are natural biological systems that will have certain moments of more optimal and less optimal functioning. They have to be accompanied by other measures to improve their functioning, such as reducing productive farms, overfertilizing, and overloading the soil with chemical products. Climate change will affect the buffer zones differently if changes are implemented. If the buffer zones were functioning properly or had been implemented in a more integrated way in the basin, they would be fundamental to reducing erosion and reducing the contribution of chemical substances, mainly nutrients. This would be desirable, especially in the buffer zones surrounding the reservoirs, which are where the reservoirs receive a large number of substances and chemicals that are going to be retained in those reservoirs.

Interviewer 2: And how does the behaviour of the river compare to that of the reservoirs in terms of agricultural activities and seasonal changes?

Interviewee: The river is very susceptible to agricultural activities and seasonal changes, and the nutrients behave quite closely with what is happening in the soil. On the other hand, in the reservoirs, there is an accumulation effect. It is an upward trend in the concentration of nutrients that we have seen over the years. They are received, processed, and recycled, and they are accumulating in the sediments or in the water column. There is less renewal. For example, in a place like Paso Pache on the Santa Lucida River, which is where Route 5 passes and covers an area of the river, with only a small reservoir there in Minas, it behaves differently with the oscillations of the river and changes in nutrients, nitrogen, and phosphorous.

Interviewer 2: Is the Paso Pache example referring to less intensive watershed management or a smaller reservoir?

Interviewee: A smaller reservoir. In Paso Pache, there is practically no large reservoir that cuts off the river, but rather, there are small productive reservoirs deployed in the basin. And that area of the river, like Centinela, responds very directly to agricultural activity. That is to say, it responds in a very coupled or correlated way to agricultural activity. It's because the flow is more fluid, and the flow of water is more connected. And obviously, it is a watercourse that circulates, so it reflects quite well what happens in the basin in normal regimes. Now we are in some quite atypical years where we have regular droughts, so we do see that effect on the water quite directly. So then, the buffer zones would have a very important function because, of course, they would have to smooth out the peaks to soften them during rainfall. And in the reservoirs, what we would have is that these pulses of rainfall would diminish what was accumulating in the reservoirs. Perhaps over the course of successive inputs with a lower concentration of nutrients, we would achieve a certain "flushing" of the reservoirs in periods of very high rainfall. Going to the extremes of what would be the functioning of a buffer zone is also something that one of my students saw in her thesis. She worked with several types of runoff water supplies because, of course, they are not treatment plants. They have margins in which the rainfall is so high that the buffer zones no longer function as such; they are overwhelmed by water transport, and they don't have time to retain water. Also, when there is drought, there is obviously no surface water to be found. So, they have to function very well in a normal regime, don't they? If we think about climate change, like here in Uruguay, which is predicted to experience an increase in extreme precipitation, then we would see a negative effect on the functioning of the buffer zones.

Interviewer 1: Are you aware of the effect of invasive animal or plant species on the use of buffer zones?

Interviewee: Yes, of course. In the basin commission, the control of invasive plant species is also a very important problem. The Faculty of Agronomy has worked on this, and I think it has worked on it with the aim of control. Of course, from a chemical-biological point of view, buffers are walls where we only focus on nutrient dynamics. Perhaps we don't care what type of vegetation is there, but that there is vegetation and that it retains what we want to retain. But from a conservation point of view, it is important that there is control of invasive species to sustain biodiversity and conserve the biodiversity of the system.

Interviewer 1: As a last question, do you have any other contacts that you think are relevant to this research, for example, from producers or other researchers?

Interviewee: Yes, someone who might be relevant is Ismael Diaz, who is currently on the commission. The geographer has done a lot of work with nutrient transport in watersheds and transport models. I think he's worked on the Santa Lucia River, and he's still working on the Rio Negro as well. Well, they are more up-to-date on what is going on now. For the little bit of science as well, Ismael. And another one would be Carlos Perdomo. I don't know if you have spoken to him.

Interviewer 2: Carlos, no, not yet. You're right; I missed Carlos Perdomo.

Interviewer 1: Perfect. Those were the questions. Do any of you have any other questions?

Interviewer 2: No, I want to thank Luis again for his valuable contributions.

Interviewee: Well, thank you very much to both of you. Your work is very interesting and necessary for this evaluation. I look forward to the results.

*Interviewer 1 is the student, and interviewer 2 is the supervisor.*

# Interview 4

**Interviewee: Producer Date: 02-03-2023**

Interviewer 1: Buffer zones in the Santa Lucia River Basin are strips of vegetation along the water that capture and retain nutrients from agricultural activities. For example, the zones prohibit certain land uses and livestock access to the water. What do you do for a living?

Interviewee: I am currently retired from my professional activities, but I am still active as an agricultural producer. I already have an agricultural business that includes livestock farming, and we are starting a fruit tree production activity.

Interviewer 1: The Santa Lucia River Action Plan introduced buffer zones in the Santa Lucia River Basin. What is your role in buffer zone management?

Interviewee: As a private producer, my role is to respect the regulations and conditions that are set for these zones. I don't know if there is anything else that needs to be done. These buffer zones can be designed in many ways. With regard to management, we have the obligation to have everything fenced so that the cattle cannot go to the river. I have to check that the fence is in good condition and repair it if the wire breaks. It's like a barrier that we all have to comply with.

Interviewer 1: What are your tasks with regard to the management of buffer zones?

Interviewee: The buffer zones can be passive or active. I don't really do any agricultural activity there. The cattle come to eat up to the limit of the wire fence. I should keep the bush a bit cleaner inside the buffer zone than outside of it. In reality, the buffer is a limit; I don't have a separate buffer here; it's all within an area that is all bush. And there are cattle there too. I don't separate the buffer zone from the rest of the area; it's a large pasture that has the characteristic of being a forest. You can't farm there. I don't do any activity in that whole area, and therefore I don't do anything in the buffer either. But I don't have a separate buffer. I don't know if that's an obligation. I don't know. It hasn't been, as far as I know.

Interviewer 2: Regarding what you were saying about having a cleaner bush, do you mean a better-maintained bush?

Interviewee: Yes, it's very dirty. I have two boundaries at the wire fences with my neighbours that we have to fix all the time, and so on. And we are opening up a bit to be able to fix the wires. I want to make some paths, but they won't let me sell that land. There is a 5-metre buffer on each side of the boundary wire. I can even install a fence there to keep it wired. But they (the government) won't let us sell the timber generated at that buffer. There are none. In other words, the bush is registered. Supposedly, the first step to selling the timber with a permit is to have the forest registered, but they don't give us the permit to sell the timber. That is a big limitation to being able to have better things. And the fence's wire would also have to be fixed.

Interviewer 2: What about the wired fence along the river?

Interviewee: We are going to fix it. I'm telling you that it's an obligation to have the river fenced. We get there on horseback, up to a certain point, and then on foot. Pablo, who is the boy who does my maintenance work here, had to tie the posts and the pegs to the horse, and we have to go a whole stretch like that because he can't get there. And the fence, as far as I know, is before we enter the watercourse. What happens is that the animals can create a gully that runs parallel to the river. The thing is, the animals would access the river by going down the gully. So the direct path to the river is the gully.

Interviewer 2: I see. And the wired fence—does it open or not?

Interviewee: No, it never opens. Even for better access. Nowadays, the way to get to the river is by horse up to a certain point, and then you have to get off. In other words, you can't get in with a tractor. If you want to maintain the fence, for example, you have to go through the gully. But you can't go with a cart or a tractor.

Interviewer 1: Do the buffer zones currently serve any other ecological or economic purpose for you? Interviewee: No, not really. It doesn't benefit me in any way. We have some invasive species.

Interviewer 2: And do you manage these species?

Interviewee: No, not really. It's a very messy process. I had a person cut and clean some for me, but it's not really a sustainable method.

Interviewer 2: I see. So you can't really control the invasive species, right?

Interviewee: That's correct. In fact, it's an impediment to good management. If you can't get there, you can't control the invasive species effectively.

Interviewer 2: And what's the percentage of woodland on your property?

Interviewee: About 30%. It includes a strip against the river that is more diverse and an area that is mostly Espinillo (*Vachellia caven*) and some invasive species. The Espinillo is regrowth, and it's not very big. Cattle don't have access to it. So, the management is limited. You only get a few kilos of firewood, and you can't really do much else.

Interviewer 2: I see. So, it's an obstacle to managing the property effectively, right?

Interviewee: Yes, it is. It's a knot that forces you to not do anything. You don't see it yourself, and it leads you to do nothing. And that develops, you see? Because now, in these dry summers, these species, like the Espinillo, flourish.

Interviewer 2: Alfred asked whether the area had any ecological or economic purpose.

Interviewee: Yes, they are the ones that dominate.

Interviewer 2: Regarding the cattle, do you have any purpose for the area with trees? Does it generate anything?

Interviewee: So, the ecosystem of the trees and the pasture below for the cattle is wonderful. And I'm particularly interested in preserving it because it provides comfort for the livestock in both winter and summer. It's a very sheltered area in the winter, and it's cool in the summer because of the shade. Even at the driest times, there is grass. In other words, the combination of trees and pasture is great. I want to preserve and improve it because it has direct benefits for the livestock, without a doubt.

Interviewer 1: Do they generate any harm?

Interviewee: No, for me, no harm. The area is untouched, and if you don't touch it, nature moves towards very different things. These hills are all covered with trees and invasive species; in other words, they are not pristine forests. Clearly, if you don't intervene, it moves towards deterioration.

Interviewer 2: So, doing nothing is negative for the bush?

Interviewee: Yes, because in this situation, what tends to happen is increased encroachment. It builds up like a clump of good, valuable trees surrounded by invasive plants, and there are areas that cattle can no longer access. Cattle clean up the bush a lot, but they have to be able to access it. In order for this to be well maintained, there has to be well-managed human intervention.

Interviewer 2: And what about biomass?

Interviewee: Yes, there is a lot of biomass there, and I thought about it this year because of the fires. That's a lot of biomass that has a high fire risk. Added to that, there are some areas that are intermingled with the Pajonal (*Paspalum quadrifarium*), so even more so. And you see that more with the invasive than with the native.

Interviewer 2: Do the native plants still survive?

Interviewee: Yes, in general, I always saw them as greener than all the others, which don't cover as much. But today, everything that's dry, I don't know if it's going to die. All the invasive plants have dried up. It's super powerful, isn't it? Of course, that's wonderful if you control it. In two years, there could be areas where the animal no longer enters. I mean, I emphasise this because I know that there are people who are of the opinion that this is not the case and that if you want to conserve, you don't have to manage. For me, that is a big mistake. In the state that our forests are in, I have an aerial photo from the year 2000, and I have an aerial photo here from 1969. You can't see a tree down to the river. Everything has been cut down. Everything in these forests has been cut down.

Interviewer 2: Yes, in almost all of Uruguay.

Interviewee: In all of Uruguay But, well, that's based on what I know and what I see from my experience and that of other people, right? I think you can go to the river to see it.

Interviewer 1: And what purpose do you think the buffer zones in the Santa Lucia River Basin actively serve for society?

Interviewee: The purpose is to maintain that zone, which is a filter zone, right? In other words, to preserve the quality of the water It also seems that this purpose is very valid. It is a very important source of water. I mean, I agree with that purpose. For me, that's the main purpose. I don't know if there are others.

Interviewer 1: And what are the characteristics of the buffer zones associated with the current buffer zones in the river that support these purposes?

Interviewer 2: If we say the purpose is to reduce nutrients, what characteristics do you, from your knowledge as a producer, think support that nutrient filter, that nutrient reduction function?

Interviewee: Well, the presence of species—that is, in order for the buffer to work well, it has to have species that are active in their uptake, right? In other words, species that photosynthesize take nutrients from the soil, right? In other words, it's like a circle. The plant takes nutrients, produces biomass, and so on and so forth.

Interviewer 2: Now, how does that work? Precisely so that the plant and the system remove nutrients?

Interviewee: The plant has to grow. If it's a tree, it grows. If it's a pasture, somebody has to eat it, and it has to grow again. If it doesn't, that circle comes to the point where it stops. In other words, if there is no permanent growth, it gets to the point where the nutrients are no longer removed from the soil because there is no one to consume them. Do you understand?

Interviewer 1: Yes.

Interviewee: But I don't know if I answered your question.

Interviewer 2: No, and maybe that’s lacking.

Interviewee: Yes, I don't think there is. I mean, as a producer, I know a lot of these things because I worked in this area. As a producer, I've never received any official communication, neither from the commission nor from the ministry of agriculture. I don't know what I have to do in that area. Does that make sense? To me, that's a major flaw in the system. There's no recommendation, suggestion, or obligation. What is well known by the ordinary people here—some comply with it and many others don't—is that animals cannot go to the river to drink water. This was publicised when the Santa Lucia measures were taken (when the Santa Lucia River Basin Action Plan was implemented, red). But then, compliance with these measures depends on the individual. There are people here who don't have fencing. These regulations were already in place before, such as the fencing against the rivers.

Interviewer 1: For you personally or for your company, what purpose do you think the buffer zones should serve?

Interviewee: As for me, the purpose they have is fine. But it seems to me that there should be more emphasis on informing and providing management guidelines. That's what I think is lacking today, especially in areas where the buffer is part of a very large area with the same management. If I were to fence the buffer, I'd have to clear out a whole area of bushland to put up a wire fence. That's actually a whole area that I manage. And if you were told, for example, to put up a wired fence and you couldn't have access to cattle, you would be losing something. In my case, the buffer is 100 metres, but over there it's only 50. It's a little bit of an alteration. From a business point of view, it's not much, and it wouldn't affect me. However, it would be a tremendous expense because I would have to fence 250 metres, as you can see, in length by 10 metres wide. When we made the fencing, we calculated that 10 metres wide is what you have to do to make an exclusion. In other words, experimental fences are obligatory. And it allowed you to have a strip of 50 metres on each side. In concrete terms, the implementation did not affect me from a productive point of view. It's a very small area. If it has to be done under the conditions as they are now, then if I do it, it's going to cost more.

Interviewer 1: Of course. And what purpose do you think the buffer zones should serve for society? Interviewee: For society. And well, for me, I mean, that concept of having an area where you preserve the forest and ensure the quality of the water—for me, that's a public good, let's say, that is useful for everyone. Look, the Santa Lucia River is a river that is very popular for canoeing and other recreational activities. I think it's great. And the way we can all have that is good; I think it's more enjoyable for everyone. Another one is for the birds. I think that's a good idea. And what's difficult is how to implement it so that it's really done and maintained.

Interviewer 1: And what features need to be changed or added in the design of the buffer zones to support these functions?

Interviewee: It depends, because here it has this particularity. This buffer has some advantages and some disadvantages. It has the advantage that it is an area that is already afforested, so I think it's very good as a buffer. It would be difficult if you had to isolate it or an area that's tucked inside a native forest. It is difficult to maintain if you can't sell the firewood. If they tell you, "Look, you can sell the firewood, maybe the costs will balance out”. Because in reality, that's the biggest cost; it's not the fencing (the maintenance, red). It's having to clean the forest. The thing is that the buffer in other areas is located in a cleaner area of the countryside. And maybe today, people have it integrated into an area of farmland. And maybe that's where you have an impact more from the point of view that there is an area that you used to have integrated, where you planted corn and sorghum, and that you can't plant today. I mean, the buffer doesn't affect me very much because of the proportion of coastline I have in relation to the area. If the field had a different shape and I had a lot of coastlines, maybe those 50 metres would imply a larger area. In other words, the first thing that stands out for me is the importance of differentiation. Not all situations are the same, and there are certain circumstances where production remains unaffected, as is the case here, for example. However, in other places, production may be impacted. This topic is crucial in relation to the previous one, as until now, we have been managing it within the crops and the area that is typically sown. The buffer is also a cause for concern, and I am unsure whether certain crops can be planted. I know about the sowing of annual crops, but I have no knowledge of fertilisation. I have worked in places where complaints were made on the San Ramón side, which resulted in them being forced to do certain things. However, the problem is that there is no clear communication, and there may not be a consensus. Perhaps there is a place where they (the government) could inform you about the native forests and suggest a combination of shrubs and trees. Ground cover needs to be permanent, or active felling is necessary for nutrient uptake. I cannot recall whether animals have to come in or not. Pastures should be taken into account, as vegetation, shrubs, grass, and trees need to be present. Nowadays, with modern facilities, it is possible to control a 10-metre or 5-metre strip with ease. The adoption of certain technologies could help maintain and supervise the area, and this would benefit society as a whole. The benefits of these changes are numerous and include a more aesthetically pleasing environment, a decrease in the economic loss of grazing areas, and the prevention of animals' access to restricted areas. As for me personally, I would like to see more beautiful surroundings. There are valuable, old trees and many Coronilla trees (*Scutia bluxifolia*) with significant growth. It is crucial to maintain the area permanently, as the buffer is an active entity that advances continuously.

Interviewee: Did you see the people who live here? The cattle come in; they're always there, you see. They control them badly. The only way to make that profit is to be able to sell some firewood. So, I made an arrangement with the people here. There are a lot of people who do that. And if you do, you keep the forest clean for me. You help me out here, and you cut a certain amount of firewood and take it with you. And with that, you do your work. But the ministry is currently refusing to do that. In other words, it would be like economic support for the management. And for me, just having that enabled helps. It's allowing an activity for them to earn something and for me to control the buffers. Enabling an activity within the same ecosystem as self-financing I’m waiting for the Ministry of Agriculture to support these management strategies. That's not good. Because do you know what the rule is? It's that people who already have a permit are still taking firewood. And they don't give out our new permits. They don't have them. We were saying that more accurate control with technology would be something else that is needed. And I think it would be something that would make it feasible for you to allow new permits. To take the firewood out and sell it legally, the person who comes to work also works in a place that is regulated. In other words, I think it's a win-win situation. Everybody wins. They should permit integrated management, and that can be taken out.

Interviewer 2: And if you had to rate these characteristics from 1 to 4 in terms of importance?

Interviewee: I think they are almost on the same level. I mean, in these situations where you are very close to the water intake of the main roads in the country, in a river that is very demanding, It is natural and logical that it should be controlled. It seems to me that today, maybe 20 years ago, it would have been unthinkable, but today you have technology that would allow you to do it in a reasonable way for everyone. That it is controlled. For instance, I'm going to remove the invasive ones. I'm going to remove some deadwood. It seems to me that with these things, you can have proper control nowadays, allow the producer to maintain the buffer zone and areas surrounding the buffer zone, and have integrated management while the area is being cared for. That it is protected from invasive species. It seems to me that what you have to do is incorporate control technology. What needs to happen is that in order to reach that stage, at least here in this situation as we are in it, it requires a lot of work. It requires a lot of work, and someone has to pay for it. Maybe some support would be needed at the beginning of the proper implementation. I'm telling you, it's very dirty here. Well, I have a field that has been leased for years. The government exploited all the forest until the end and leased it to a dairy farmer who never did anything to it, so I am in a situation of zero maintenance. I told the forestry department to put the inspector at my door to come and see me every day and give me a drone to fly. I have no problem with complying. I want to maintain the forest; I'm interested in it. But on the contrary, as you saw, I'm still waiting for it.

Interviewer 2: Do you see the benefit you get from registering?

Interviewee: I have a benefit from registering the native forest. Here, there are 16 hectares registered for that area, but for the native forest that was already there before. It had been there for many years, and it has two benefits. I particularly don't pay for the contribution of those 16 hectares. The other benefit is that here the tax does not apply because of the number of hectares. The other benefit that it gives you today is that you are exempt from the contribution of that area, and they exempt you. I don't know how it exactly works; they give you some of the BPS (Banco de Prevision Social), but you need to have 500 hectares with the BPS. Of course, I have a contribution of 50 hectares here, 70 with BPS, so I pay 150. No, no, it's 50. And if you take it with BPS, where the BPS contribution is 70, then there is a minimum BPS contribution, which is what you have to pay, so there is no exoneration there.

Interviewer 2: I think that has been the benefit and the disadvantage.

Interviewer 1: And what drawbacks do you expect from the implementation of these changes for society?

Interviewee: Well, if it is decided that an incentive should be given so that these areas are really implemented, it is possible that we have to make a contribution of monetary resources, let's say from somewhere else, in order for this to be carried out by way of subsidy or by way of incentive. This would be a disadvantage in terms of allocating economic resources. On the other hand, for this to be implemented and with the benefit of having better water quality.

Interviewer 1: And what obstacles do you think will be experienced with the implementation of these changes in the preferred areas? Obstacles in general, for example, for regulation, management, or any other processes that you think are relevant for the implementation of these preferences.

Interviewee: There are many obstacles. The first obstacle is ensuring that there is a clear and adaptable guideline in place for what needs to be done in different situations. Without this, it is impossible to effectively convey ideas and fulfil them. Once this hurdle is cleared, the next challenge is implementing the measures and ensuring that they are followed by producers. This can be difficult because each producer may encounter different difficulties depending on the specific situation they are in. Another potential obstacle is determining who deserves support and who does not, as there may be situations that require support and others that do not. This requires differentiating between situations with more or less change and considering the area that is affected, as this can have a significant impact on productivity. To overcome these obstacles, it is necessary to establish clear parameters and use a matrix to differentiate between situations that require support and those that do not. It is important to start with a long-term goal and work towards it, rather than focusing on short-term solutions. Control is also crucial, as there are technological tools available to ensure compliance with guidelines and measures. However, control in Uruguay is currently lacking and needs to be improved. Overall, it is possible to overcome these obstacles by paying more attention to specific situations, applying resources effectively, and implementing better control measures.

Interviewee: The first thing to do is set yourself a goal and a timeframe. Is it 10 years old? Is it five years? By that year, we should all be complying with these regulations. For that to happen, interdisciplinarity is needed. It is necessary to agree on what the management is, what the recommendations are, and to set up an implementation stage. I believe that we are lacking in all of these things. Making a wired fence is something that is done quickly, but managing invasive species is a process of control, so you can't expect everything to be done at the same time. You can consider the first stage to be getting the cows out of the river, and then it's like a sequence. Yes, yes, that is the first obstacle to the stated action plan.

Interviewer 2: I want to ask a question about You were saying that there are situations in which there are fields where crops grow close to the water and fields with forests. And you mentioned that there is a forest here because someone didn't cut it down, and in other places, there is no forest in some cases because it was cut down because that control was maintained. Why do you think that is?

Interviewee: I think that if you look at the photo of my grandfather in the 1960s, there are very few places in the Santa Lucia basin that had more than 50 metres of trees 60 years ago. It is obvious that there are no trees in the places where today's crops are grown because they were cut down. Here is what my mother told me: she had a rented herder who did the rearing and managed it as if it were a paddock on its own with the gates open. He simply brought the cows; there was no need to do anything. Maybe it is influenced by the topography of one side of the river towards San Ramón. Right here, you see the countryside next door, and it is located much lower than this one. And at a distance from the river, 300 metres apart, there is a pile of Espinillos almost up to the house. I take the photo of the 60s as an initial stage because that kills the thought that you are not working on a pristine that no one has ever touched, which is that it was only intervened on by the grazing cows. The reality is that you are starting from a forest that was cut down; if you go back 60 years, there were no trees. And remember, in the official figures, native woodland has been increasing in the area, according to the ministry. These data are increasingly refined because they are done with satellites.

Interviewer 1: The next questions are about your experiences with climate change in the St. Lucia estuary. How have you experienced climate change here in the St. Lucia estuary? Think of temperature changes, rainfall, droughts, or floods that have been noted in the basin over the years.

Interviewee: I've been in the countryside for eight years, working it myself. That is to say, I don't have a very good perspective from a broader point of view. I don't know if I can associate what I've experienced with climate change because it's a controversial topic. Here in this field, flooding is normal when there's a lot of rain. We're close to where the river meets the tributary that comes from Florida, from Paso Severino. So with very big rains in Florida, they have to open Paso Severino, and the water comes up here and causes floods. It happens relatively often, about once or twice a year, which is normal. This is due to the normal river flow and the rainfall upstream, where the Santa Luca river rises, or the management of the Paso Severino Reservoir. It's an issue that's typical for the area and the topography that we have. After three years of being here, we've had a drought situation with a drop in the water level of the surface water table of two metres around here. In fact, in Pozo Viejo, we often don't have water anymore. If that's due to climate change, it would be very much discussed. We had three consecutive years with an impact (due to droughts, red). The water in the well went down but then recovered a little in the first year, but last year it didn't recover, and this year it disappeared. The well is five metres deep, and so is the water table there. It used to have a good flow, and we were able to get around six thousand litres per hour. We irrigated with that water until October. The floods were short. In fact, last year in the autumn, there was a very heavy rain in the headwaters of Santa Lucia, and the water came up. It cut the field, but two days later it went down. That's a normal occurrence to be expected.

Interviewer 1: What do you think about the effects of climate change on the buffers in the area? Do you think that the changes you propose to implement would make it more resistant to drought and flooding?

Interviewee: Yes, I believe that if one manages the buffer right, it should be more resistant to climatic changes, at least to drought. The more competition there is between plants, the more they will suffer from water. If you have fewer trees, that goes in favour of having a plant assemblage that is more resistant to drought or flooding as well. Because when the water rises, the clearer the ground, the faster the water runs off. Today, you have a lot of barriers because of all those dirty places. But thinking about a situation with a drier summer or spring, good management would favour having fewer large trees with less competition between them.

Interviewer 1: Have you had any experience with invasive animal or plant species on your property, such as wild boars?

Interviewee: I'm not sure about animals, but there are certain trees here that rival the native trees that are drying up. And the wild boar. The wild boar is a big problem here because it breaks the wire fences. There are places over there where they are just passing through, like this one with my neighbour. I learned to recognise that because, to be honest, I didn't recognise anything at first, but the people here teach you how to look. The wild boar breaks the fences and brings the hunters another invasive species. If suddenly you feel that the cattle are all clustered together, the humans can't divide that big area, which is about 30 hectares. If you leave the cattle tightly packed in the bush, the wild boars will come and go, and the hunters will come around and scare the cattle. They need to be able to escape if this situation arises. Well, if you were to make a paddock with the buffer zone, divide it with electricity, or do something that is not permanent, that would be problematic. The other one here is deer, and I don't know if there are people who hunt deer. There might be. I think that it is an animal that, in itself, is less destructive. They eat grassland. I don't know, here on the side where there is grassland further down, but yes, this is the wild boar with the hunters behind, which is a problem.

Interviewer 2: And the deer or parrots?

Interviewee:  I don't know if I feel it. I don't know how I feel about the parrot. And then the vegetation we talked about today: Zarzamora (Rubus ulmifolius), Ligustro (Ligustrum lucidum), and Gleditsia (Gleditsia triacanthos). I don't know; don't order there. For me, first, the Zarzamora is  more difficult to fight. You see, Ligustro, you can cut down the individuals. The cattle eat it. If they are hungry, eat it all. No, what it has is that it seems to be a kind of allelopathic medium that doesn't grow in other vegetables, and the Gleditsia doesn't seem to have less; of all that, there is less. And then the hawthorn, which I don't know, and the spinach are also there, and there is little brake, let's say, in comparison to what you see in the rest of the river. If you stop there at the bridge on Route 5, then you see the Espinillo (Vachellia caven), and the truth is that the Espinillo is a species that can be a friend. It can be a friend or an enemy. If you hit it, it is an enemy, I think. Did you see all that light green stuff you see there? All that is Espinillo, and those are resprouts or advanced. There are old trees, and there are many sprouts. In fact, they grew here.

Interviewer: 2 Can you tell me about the idea behind encouraging the presence of scavengers, hunters, and carrion?

Interviewee: The idea is to encourage the presence of scavengers, hunters, and carrion by putting up tall poles and a place for them to separate and encouraging them to come with food. When you walk into their area, it doesn't smell anything. When the hunter goes away, the parrot comes back to eat. At the top of the tree, where there is a parrot's nest, there's a scattering. You see, there's not one left after that happens. It's very funny.

Interviewer 2: What about falconry in Uruguay?

Interviewee: There are falconry services for cultivation, for the airports in Montevideo, for squares, and for the cathedral. The presence of Espinillo and Zarzamora above all is caused by the exclusion of pastoralism. Currently, there are difficulties for some species in the buffers. In fact, today, in what is called the buffer zone, there is no hawthorn. If there is any, there is very little. In general, the Espinillo makes it very difficult for animals to enter the buffer zones. I don’t think it's right that invasive species are respected as being part of the natural diversity of native species in buffer zones. For me, it depends on the criteria with which you define the management of the buffer area. If your intention is to wire it up and leave that to pass the time, for me, that is a source of contamination because what's going to happen is that it's going to fill up the buffer. It's going to cause competition between the good trees and all the invaders. That's why I was telling you that, for me, management is fundamental because it defines the buffer area. For me, the management is going to define the impact that the buffer has on the area. If you don’t touch the buffer, then that is a source of problems because it's going to get dirty. You are going to have a multiplicity of exotic species. Invasive fruits may arise, causing the buffer to potentially be full of birds that will eat the fruits and scatter them. That will become a source of problems.

Interviewer 2: Do you have any contacts with producers or production companies who can collaborate with us?

Interviewee: I don't know; maybe they want to collaborate without any commitment. I have some neighbours, but they are busy. Yes, Esteban; for me, it can be yes. Let me call him and ask him. He always says yes; I'll pass the contact information on to you.

Interviewer 2: Yes, no strings attached.

Interviewee: How many people do you need?

Interviewer 2: Between 4 and 5.

Interviewee: This person seems to me to be a good reference because he is an agricultural engineer, but that field belonged to his grandfather. He is a person who has this problem; he experiences it very much, if you know what I mean. He has a history that I don't have. I haven't been here very long. I can't tell you if the dynamics of the river have been changing. I don't know if what I've been told is true, but he does because I'm telling you that field belonged to his grandfather, so he has experience because of his family and everything in the area, which I think is good. Furthermore, one producer is more like a typical producer who has fields on this side as well as on the coast. He can give you an insight into a typical producer who is not a technician and has no other source of information because the other one is a very knowledgeable guy who passes things on to me from the Washington Post. You know what I mean?

Interviewer 2: It seems to me that Juancho can be good if he has the vision of a typical producer. That is the type of source we are looking for. How is the time?

Interviewer 1: Good. Thank you for your time. The recording will be terminated.

*Interviewer 1 is the student, and interviewer 2 is the supervisor.*

# Interview 5

**Interviewee: CNFR        Date: 02-03-2023**

Interviewer 1: Buffer zones in the Santa Lucia River Basin are strips of vegetation along the water that capture and retain nutrients from agricultural activities. For example, the zones prohibit certain land uses and livestock access to the water. What do you do for a living?

Interviewee: Well, I'm an agricultural engineer, and I've been working in the Promotion and Development Department of the National Commission for Rural Development (CNFR) for 17 years.

Interviewer 1: And what is the goal of your organisation?

Interviewee: Well, I am part of the Promotion and Development Department, which would be more of a rural extension area. We work with our affiliated organisations, and the National Commission for Rural Development is a national organisation that has more than 100 local entities. It is a second-tier entity made up of more than 100 local organisations in the 19 departments of the country. The function of the Promotion and Development Department is to be in as much direct interaction as possible with these 100 organisations to promote development projects and also to be able to generate training opportunities at different levels. Both in terms of production and rural development, as well as in terms of representation and defence of the interests of the associated producers, who are mostly family producers. We are an organisation that brings together a significant number of producers—more than 17,000, according to a census carried out by the University of the Republic in 2017, and most of them are family producers. We work directly with the organisations, especially through development projects, which can cover multiple areas. And we try to have coverage throughout the country, which is not so easy for an organisation like ours.

Interviewer 2: One question. In Flores, which organisations are members of the Commission?

Interviewee: I remember at least three, including the Sociedad de Fomento Rural de la Casilla, which is an organisation of dairy farmers. The Sociedad de Fomento Rural de Chacras del Porongos, which is a small producer from the outskirts of Trinidad, is from the capital. Most of them are cattle ranchers, farmers, small animal breeders, and cattle breeders; there is a bit of everything. And then we have the Cooperativa Unión Rural de Flores, which is mainly dedicated to agriculture and some livestock farming. There are more medium-sized producers. The Unión Rural is also affiliated with the Cooperativas Agrarias Federadas. It is affiliated with both organisations. Those are the three that we have.

Interviewer 2: Well, I'm from Flores, so I have more or less an idea of the scope there. I think it's all there, in the whole department. I don't know if there are any left.

Interviewer 1: The next questions are about your understanding of your organisation's role in the design and implementation of buffer zones. What are your or your organisation's responsibilities and objectives in the design and management of buffer zones?

Interviewee: Well, that's a good question. The first thing I need to clarify is that I personally did not participate in the buffer zone formation process or the Santa Lucia River Basin Committee, which I know was in operation for a long time. I did not participate in those instances. I have some information on how it has happened because I have participated in river basin committees in other places, but not precisely in the area of the Santa Luca river. But equally, to continue answering your question, what is fundamental for the National Commission for Rural Development is the definition and position of local producer organisations in these spaces. Citizens have to participate. And since it is a rural area, in this case, those are the rural producers of the area where we want to intervene, which is especially the lower basin of the Santa Lucia River. It is somewhat in the area closest to the metropolitan area, where the water intake is located. So, what the National Commission has always tried to do is get producer organisations in the area, and we encourage our affiliates to get involved in the management processes of the territory where they live. We think it is healthy to convene committees to discuss how we are going to manage a sensitive watershed. And that has always been our aim: to try to make the organisations aware that decisions are going to be taken there that can affect them and their associated families. And therefore, it would be good to have an incentive to participate and get involved. And generally also by proposing opportunities, and that is where we often get support from the National Commission. For an organisation of family producers, it is difficult to participate in all the existing spaces. One of our areas of work is the River Basin Committee with the buffer zones, but we have many other spaces, especially in Canelones, where we also have the Rural Development Roundtable, and sometimes the Municipality also invites participation in other spaces. So in local organisations, there are often few people who can be available to occupy these spaces and really have an impact. Because sometimes, well, some people go and don't dare to speak because they find it difficult to talk to a technician who has a lot of training and who provides information that sometimes overwhelms them. So, well, it's a difficult situation, but the important thing is to make the effort to participate. I think this is our main objective: to try to get our organisations to participate in local development processes, such as a committee that defines the management of a sensitive area. This is very important for us, and whenever we can, we support it. We try to get the local organisations themselves to do it because they are the ones who know the territory, the ones who are there. I live in the department of Rocha, and I know little about what is happening in that area. But suddenly, if they ask me for help and say, "Can you help me look for information on a particular issue that is being dealt with?" we try to help, and we always try to help with that. We are technicians, and we have more training than some producers or leaders to deal with these things.

Interviewee 1: And what relationships do you or your organisation have with other stakeholders in this process?

Interviewee: Well, the National Commission as an organisation has always had a tendency to generate alliances and agreements and to engage in constructive dialogue. This is a policy of the organisation and has always been so. The National Commission, as well as having this department of promotion and development, that is, this technical team, which is quite small, but we are available to work with our affiliates, also has a trade union function, that is, to defend the interests of our affiliates, the family producers. So, in this strategy of defending the interests of family farmers, we always try to generate agreements and understandings with all the actors involved in a given situation. So, for example, the National Commission participates in all inter-organisational dialogue bodies. The National Association of Milk Producers (ANPL, red), for example, is very interesting because it has many dairy farmers associated with it. So, we tend to try to reach common positions with other associations to the extent that we have common visions. Sometimes we don't share the same visions, but we always try to have dialogue. And the same with the authorities, the same with the Municipality of Canelones or the Municipality of Montevideo. We have cooperation agreements with several municipalities, which means that we try to agree on plans and projects and support each other. And it also happens with the Ministry of Agriculture in its different areas. Specifically, to intervene in this area, today we do not have a specific agreement with any institution, but we do have a very good dialogue and a very good understanding with any of these institutions because we always try to build dialogue. We search for dialogue, not confrontation, which does not mean that at some point it may not be necessary, but the political tone of the National Commission for Rural Development has always been dialogue. So, we always try to get closer and reach an agreement with all the institutions that are acting, in this case, concerning the creation of the buffer zones. Sometimes we also have disagreements. Sometimes things happen that are really imposed, where someone tells people what they can and cannot do. And sometimes, when that happens, we need to have a dialogue. It's not good that things are imposed because there are people who say this is what you have to do. On the other side, the people who live there don't agree. And that is where we try to help generate those agreements. At least from the point of view of our leadership—the producers who are leaders—we are always committed to that. That is to say, "Well, let's try to reach an agreement because we are not going to solve it by confronting each other. There are times when we don't reach an agreement, and, well, we mark our differences with what is defined and the way things are going. We are not the owners of the truth either. But we always have this tendency to build agreements with all the parties that are involved in a local development process. Furthermore, I would like to add one piece of information that might be important for this. I don't want to forget it. The National Commission for Rural Development was founded in 2008; we are talking about 15 years ago. It made a cooperation agreement with the National System of Protected Areas at the time when the National System of Protected Areas was being created. This agreement, which lasted about 4 years, was very interesting because it allowed us to generate processes to support the inhabitants of the protected areas that were being defined at that time, the first protected areas to be integrated into the national system, and to promote participation in the involvement of the local population in rural areas. In the case of the Santa Luca area, which is specifically the Santa Luca wetlands protected area, we had the advantage that there were several producer organisations linked to the National Commission for Rural Development. So, although we intervened and accompanied the process so that they were involved in the management of the area, we developed our strength in other regions, much further away, where we had less institutionalisation of producers. And well, when we focused on getting people organised, we were able to reactivate some rural development societies. But well, I didn't want to forget to mention the agreement we had with the National System of Protected Areas, where one of the areas where we worked involved local actors linked to producer organisations. Well, one of the areas was the wetlands of Santa Lucia, which is one of the areas where they're considering creating buffer zones, right? And I thought it was important for you to know about that. So here goes. That's two things.

Interviewer 2: A protected area that you saw as important, where the rural commission was important, was Santa Lucia. The National Commission has been accompanying the processes, especially the implementation of protected areas, right?

Interviewee: I didn't mention any specific ones, but I can mention them to you. What I was saying was that we had focused on five areas, the first four being the Santa Lucia wetlands and their streams. Those were the first four areas where we proposed to work. Then we incorporated Farrapos, and I think that was the only one we incorporated. So, what I was saying was that in the wetland area of Santa Lucia, part of the work was quite well done because many producer organisations were active at that time. Because other programmes in the Ministry of Agriculture, such as Uruguay Rural, had done work to reactivate rural development societies, people who were on standby, so to speak, were not functioning. So we were able to capitalise on the mobilisation that took place through the Ministry's projects to get people to organise again so that people could get organised again and take control of their organisations. And yes, during the creation of the protected area in the wetlands of Santa Lucia, we had the producers quite organised. This was not the case in the other areas. For example, in the area north, there were fewer rural institutions, so we had to lend a hand to begin the process of getting people to come together and lose their fear of protected areas. There was a lot of fear. People thought that they were going to be thrown out and that they were going to build a nature reserve. But it wasn't like that; the idea was that the protected areas were managed by the people; they are the ones who have conserved the place in such a way that it has values that merit its conservation. So, as all places have their particularities, we worked more intensively in that area where the local community was less organised and less developed, and in wetlands in general. And in wetlands, we had a more organised framework; for example, several civil societies were active and already had interests and capacity to get involved in the processes in the wetlands.

Interviewer 1: Great, now I understand everything very clearly. Thank you. The Santa Lucia River Basin Action Plan introduced the use of buffer zones in the Santa Lucia River Basin. These buffer zones can be designed in many different ways. For example, using or promoting different land cover types and management strategies Therefore, there are different ways to define these buffer zones. The following questions are about how to define actual buffer zones. What functions do you think buffers currently have in the Santa Lucia River Basin?

Interviewee: Well, the first point is that we understand that it is correct that public policy or the public sector should promote differentiated management in ecologically sensitive areas. We agree with that, and that is why we also made an agreement with the national system of protected areas because we saw it as something good. After all, the country is having very severe environmental problems and has to start solving them. Therefore, defining a sensitive area, such as the Santa Lucia River and its wetlands, to be able to promote forms of management that mitigate the problems that are occurring there seems important to us. In this sense, we agree with the creation of specific buffer zones for the controlled management of the different activities that are carried out there. Not just agriculture, but livestock farming is another important thing. It is often said that the main people responsible for the contamination of the watercourse are the rural producers who use herbicides and pesticides. We also have others; in an area like the Santa Lucia wetlands, there is a very high population density. There are industries, so the first thing is to say, "Well, maybe we have to look at which are the main agents that are generating environmental problems in the area. This does not mean that rural producers are not doing it; we know that part of the problem is the rural producers themselves, who use certain practises. So first of all, we need to agree, and secondly, we need to diagnose as much as possible which are the sources of the problems and who are the people, institutions, or companies that have to contribute to solving them. And as long as they are rural actors, we are willing to collaborate in the search for solutions. In fact, we understand that it goes hand in hand with the incorporation of certain practises for which the National Commission for Rural Development has been working for many years in different processes of participation in different participatory research processes and with different institutions. Maybe I am anticipating some questions that will come later. But for us in the buffer zone areas and whenever we are talking about intervention on rural properties, the most interesting thing is to try to ensure that those technologies implemented by the producers that are generating problems can be transformed in some way. We can discourage the use of practises that are causing problems and replace them with much less polluting and more environmentally friendly ones that we have evaluated and validated on the farms of rural producers. This does not mean that what works in one place can be transferred to another with the same results. Each production system is a different world. But at least it gives us certain guidelines. So what seems to us is that these transformations that production systems require, for example, to start using less fertiliser in one place, should be transferred to another place. One example is to try to promote other types of fertilisation agents or to change some crops that are very demanding in terms of agrochemical use for others that are not so demanding but that the producer family can manage and that production is really viable. It is quite a complex problem, but there are solutions, and I think that through certain practises that have already been tried and tested, we have clear clues. For example, in the area of the Santa Lucia River, in the fruit-growing area, integrated pest management has been implemented for quite some time now as an alternative to reducing the use of pesticides. Other growers are avoiding the use of herbicides by using mulch. Others are trying to avoid permanent fertilisation with chemicals through the incorporation of green manures or legumes that fix nitrogen in the soil. So these kinds of technologies are available. Not all farmers know about them, and not all farmers apply them. Well, maybe the buffer zones are the key areas to start promoting these types of practises, so that the changes in the production systems that have to take place have to be gradual and adjusted to their real situation and their real possibilities. And they should not be imposed, because if you say to someone who has a dairy farm, "No, you can't do dairy farming anymore because you are polluting with effluents", a person who has dedicated themselves, a family that has dedicated their whole life to it, you leave them out in the cold. In short, this reconversion is not so simple. So that's why we say, "Well, we are going to see as many cases by case as possible to understand which are the necessary transformations in the systems and which technologies are proven and which we should start to promote. You must talk about the productive unit when you are already looking at the problem, that is to say, the potential problems, as if we were looking at solutions.

Interviewer 1: What functions do these buffer areas currently serve?

Interviewee: Well, there are appeals to information as an agronomist and as a person sensitive to these things. What is clear is that what is intended is to respect the areas that rivers naturally occupy and the vegetation that is nurtured there, including its flowers and fauna. In other words, all the biological diversity that is nourished by that riverbed It has been very common in Uruguay that producers, in their eagerness to conquer new areas to produce, build, or develop projects, advance on very fragile areas, and this then generates problems. Now, for example, when people naturally start to get into the areas that they can't enter when the causes are normal, But what happens? I cultivate, I do something, I intervene, and then when the water regime normalises, everything floods. and it floods even more because I eliminated the vegetation that was protecting me and that acted as a containment for the riverbed. In addition to that, I affected the biological diversity, the fauna, and the services provided by these natural environments, especially in terms of containing the overflow of the rivers. Not to mention all the functions they fulfil for society in terms of cleaning the water, conserving soils, preventing erosion, and transporting soil soluble (which are pollutants). I understand that there is a reason why they are there; naturally, they are there. Human beings have to learn to say, "Well, the limit of what seems to me to be the agricultural frontier or the productive frontier reaches a certain point. And I think that the buffer zones aim to reconstruct that point. Well, if we go too far into the river margin, we have to move back and leave the areas free so that this continues to act as it naturally has to act and does not generate problems in extreme situations. And that they also fulfil the ecosystem services that these places provide. I get it from that place and from that idea.

Interviewer 1: What functions do you think buffer zones should have in the Santa Lucia River Basin?

Interviewee: The buffer zone seems to me, first and foremost, to be areas where the nature of the place can be rebuilt so that it can fulfil its ecosystem services. From an environmental economics perspective, it may be best to let things be the way they naturally are and give up part of the productive area to avoid running the risk of generating problems as serious as those that are being generated. It seems to me that what the buffer areas have to do is recreate the natural system as it once was in those areas. I am not talking about going beyond the areas that are already defined as buffer zones, but that a strict conservation or restoration plan can be implemented in those areas so that they can comply with those ecosystem services, prevent rivers from overflowing, and prevent soil erosion. In other words, they act as conventional barriers. If we also implement the practises we were talking about today in the areas close to the buffer zones at the level of agriculture or livestock farming, we will be helping to minimise the problem. But it seems to me that the function of buffer zones is to allow the ecosystem services of these fragile areas to be fulfilled and, above all, to prevent complicated situations from occurring, especially in times of climatic crises or climatic overflow. In the Santa Lucia River, it is clear that if you clear the land, you remove the forest from the riverbed. When it rains a lot, it overflows, and people start to have problems. And it washes away soil and nutrients from fertilisers and generates serious problems. So, let's leave it as it was and limit ourselves to producing in the area where we know we are not at risk of upsetting the natural balance here. That is what I believe the buffer zones should be. In short, protected areas within each property It is very healthy, and I believe that there is a growing awareness, or I would like to believe it, that in each productive unit, we have to start respecting these spaces. That is to say that it was not common a few years ago; the normal thing was not to gain space for the bush, to gain space for the marshes, to burn them down, try to dry them out, or make canals so that the water drains away. So there, I can plant a meadow and take the animals there. And in a few years, there would be a flood, and I would lose everything. And then the farmer would often go out and ask for support. He would say, "Well, the field was flooded, but you went where you didn't have to go. So I think we also have to do the work that is already being done. I think that on a social level, it's not just the merit of one institution or another; people are gradually becoming aware that nature is stronger than us. That is what I believe, and it seems to me that more people are beginning to understand it.

Interviewer 2: You talked about recreating the natural system within the buffer areas to avoid the overflow of rivers, allow the rivers to overflow, avoid soil erosion, avoid the transport of nutrients, and carry out friendly agricultural and livestock farming practises in these buffer zones. If you had to rate these functions from 1 to 4, how important are they?

Interviewee: I am going to do it based on my perception. I'm not a specialist, honestly, so I'm going to be more of an actor. I would give avoiding soil erosion at least a 3. Avoiding soil erosion, transporting fertiliser, and transporting nutrients are the same, let's say. Exactly, and I think we can give it a 3 there as well. Furthermore, recreate the natural system, which I see more as something associated with the diversity pathway. For me, that's the most important thing of all. Some producers may say, "You're crazy, but I'm talking on a personal level there. There's a neighbourhood where nature acts. If not, everything gets out of hand. I think like that.

Interviewer 2: But if you were to say that you could produce within these buffer zones and that they are sustainable, how important would you consider it?

Interviewee: I think it is very important because otherwise, people perceive them as lost areas. They are no longer lost areas. In other words, it is a question of knowing how to value the services they provide, isn't it? It's very common; for example, I've seen areas cleared—not in that area, but in other places—by people who destroy the forest and then go around planting trees so that the animals have shade. So, the forest is providing a shaded service. The bush allows the cattle to have access to a place where they can suddenly put a watering trough so that, by drawing a little bit of water from the river or from some springs, the cattle can drink in the shade, and it's more comfortable. But that doesn't mean that we have to eliminate the bush. For me, that's a 4. In other words, this area is useful. They are. Whoever needs firewood has it. Whoever needs shade has it. Anyone who needs access to water has it. Those who like to collaborate with nature have it better than ever in those places. In other words, they also have cultural, recreational, and natural value, which is almost intangible. If you study economics, concerning ecology, it's like putting a number on these things. It's the world we live in. For me, the value goes beyond the fact that you can put an economic value on it, as the world we live in demands it. There is a value that is not transferable. And that is worth gold. That, I believe, is what human beings can never lose sight of. Life is at stake. Life. So that's why, for me, they are the most important thing. That is to say, these areas are worth a lot. And to be able to develop activities, whether they are productive or rational, is very important. Because if not, otherwise it's annoying. That is a lost area. People call the native bush "la mugre" (grime; dirt; filth; muck; red). That's what bothers me. I can't go into that pasture because I have native bush, and the cows get lost, they get punctured, and they get caught by snakes. It's like something that gets in the way. However, those who have become aware of its importance manage it reasonably. Some thinning, the elimination of exotics, allows some cattle passages so that they can get to the stream and drink water and have shade in the summer. So I think it is extremely important. I think that is perhaps the most important thing: that people learn how to make good use of these spaces and integrate them as such, as part of the productive system. Not as something that is on the outside because it is better. And if I can, I'll fill it. I think the idea is just to start to avoid that way of looking at natural buffer areas, which naturally already exist. We have to recreate them because they have been affected. And I think that the wetlands also fulfil a similar function, especially in terms of water purification, don't they? And not to mention, I'm not talking about biodiversity, because I think that's one thing. But it's probably not one for most of the people I'm representing. So I say that more in a personal capacity.

Interviewer 1: What obstacles do you think will be experienced with the implementation of these buffer zones?

Interviewee: I think the first obstacle is the cultural barrier between the people. I don't think all people are willing, or at least willing to comply in a very rigorous way, with these kinds of things. All right, I'm going to define that from this line towards the river, I don't touch anything else. I don't think it's very easy for me to say it because I am not a rural producer on the Santa Lucia River. If I were a rural producer, I don't know what I would think. I would imagine I would because it's a matter of conscience, but I'm becoming aware of someone else's resources. So, I think there might be an obstacle there—to say well, maybe not all the people who reside in those areas are willing to comply with a plan of conservation. Well, with the plan that is designed so that those areas can have a particular type of motivation, In fact, I'm not speaking for the National Commission; I'm speaking for the whole community that may be involved in this. From large producers to small industrial producers to residents, you name it. The first obstacle is going to be getting so many people in agreement that we have to begin to intervene in these areas and that, as a first thing, we may come to believe that we are going to lose. I lose a part of the productive area, and I lose sovereignty over a piece of territory that belongs to a different entity. Well, there is often the fear that if I leave the forest up there, then the forest is going to come and will continue to advance towards where I am. So, I think the main obstacle is, it seems to me, more of a cultural issue because people understand that this is necessary, whereas they may see it as something that is going to harm them in the short term. That it has no benefit for them; that's why it has already been studied. Otherwise, we wouldn't be talking about recreating or managing buffer zones. And furthermore, many of these management practises within the buffer area and outside the most productive areas have also been studied and found to be beneficial. Not only for the environment and biodiversity but also for the producer family. These are longer-term processes. For example, today, I think you should know that it is a great opportunity, not only for the Santa Lucia river basin but for the whole country, that we are working on the implementation of a national agroecology plan. This national agroecology plan, which is being influenced by public bodies such as producers' institutions or technical institutions, is beginning to generate agreements on the practices that need to be promoted. And these practises that they are trying to promote, especially for production systems, are already validated, and it is already known that they give better results. So, they may not be seen quickly in the producer's pocket. For example, it could be the transition to organic production. Most producers say, "Pah! Where do I start?". However, many are already doing it, and all of a sudden, to be able to get better water, instead of pouring 100 kilos of urea, pour 50 and let the chicken graze. But now it is not eligible for agroecological certification; let's say he is not a producer who wants to be certified. I gave that example. So, these cultural barriers, which can be the main obstacle, which can be the ones that you would expect, take time, a lot of time, and a lot of dialogue and capacity to reach those agreements that we always talk about as being essential for things to happen in the best way. And it's not about reaching a conflict where they say, "Ah! These guys are forcing me to close down the forest, and they also ask me to convert, to stop using fertiliser, and to stop using medicine." Well, if I'm going to demand that, I'm going to help you. In this case, above all, family producers are perhaps the ones who find it most difficult to incorporate new technologies, not only for economic reasons but also for cultural reasons or access to technical knowledge. They often need help. We are going to give them a hand so that they can make the transition and begin to incorporate these practises that are also required in lowland areas. Another obstacle could be the lack of resources or the timing of the processes for these things to happen. There are bound to be conflicts in the middle; there always are. But if we have patience, know that these are long-term processes, and manage to sustain them over time and provide the necessary resources, we should get a better result in the long run. So, here I added two more obstacles, in addition to the cultural aspect: the availability of resources to be able to implement the plans that are agreed on in these areas, and then time and long deadlines. Some things cannot be summed up overnight. And that implies sustaining this policy over time. Tomorrow, a departmental government or a national government can change. There are things that, if they are long-term, have to be preserved. They have to be maintained; they have to continue. The National Agroecology Plan, fortunately, has been one of the cases where, although it has had some clashes and explosions during the change of government, it has continued. And that speaks well of the country and of the fact that the processes continue, with some differences. We were able, because we didn't agree, to give continuity to the National Agrarian Plan without anyone getting off the boat. In other words, these kinds of examples are good. Let them serve as a reference model for this type of intervention. It's not easy, and that too is not easy.

Interviewer 1: What possible solutions do you think can be made for this? How do you think these obstacles can be overcome?

Interviewee: Well, there's the question. Let's see, with processes, how to sustain medium- and long-term processes. How do you do that with political will and resources? There has to be the will that this process of developing the Santa Luca river basin has been going on for 10 years; it has been going on for 15 years. We have to aim to give more strength to the younger people, who are the ones who are going to last the longest and will be the future managers of the area. We have to provide resources. Organisations are there for a reason. They can also be good channels for those resources. If I want to reach out to producers so that they start implementing measures in the areas that we define as buffer zones and they need support, give them technical support and some economic support. And above all, these processes are sustained, and we have enough patience so that they do not disappear over time. So that what is being done today in three years is not erased. Because this also generates frustration among the people. If a decree is introduced in a few years that reintroduces the buffer zones after it failed once, many people will say, "Ah, they came 10 years ago, they made us meet about 40 times, they told us they were going to help us, and then they left”. And that sometimes happens. I'm not saying it's happening now, but it happens in some cases. So, sustaining a long-term process by providing resources and giving time And when I mean resources, I don't just mean money but also knowledge, technological alternatives, and technical support to be able to support producer organisations. In this case, they can participate in the management of these areas. Well, where does the money come from? Well, let's theorise. Countries have resources. But I believe that the solution should always be through dialogue, right? Always trying to reach an agreement with the other party. Because if we don't drop the bomb and leave, some are going to be left out, and we won't be able to manage the area in an integrated way. So the resources have to be democratically allocated.

Interviewer 2: A little question in line with that. How do you see the role of the National Commission in this type of environmental policy? I don't know if anyone has experience with that.

Interviewee: Yes, of course, yes. If I'm from the Ministry of the Environment and I want to develop a buffer zone plan in a watershed, I can go and knock on the door of each of the houses. That's going to take a lot of work, or I can go and meet with the institutions that represent them. And I'm not just talking about producers; I'm talking about any other actor that is in the area. That's the easiest road, to the extent that these organisations fulfil their real function of representing their members, which they do quite well. We always say that for local actors to organise themselves, the organisations have to be strong, and that also implies management capacity, resources, time, and political capacities, which not all rural dwellers have. But we are working very hard on the training of resources and leaders. We are not going to talk about resources. But to say, "Well, if today or tomorrow I live in an area in the rural development society and I have to talk to the environmental engineer because the plan for buffer areas is coming up, I have to know how to handle him. I need to have developed the capacity for dialogue and not feel like a wet blanket, as we say here, when a technical guy comes in who speaks very well and I don't even know how to respond. So, developing those capacities and skills is part of what we want to contribute. As leaders, we are always inviting others to participate in the training process. And the focus is mainly on women and youth. So, this is one of the big contributions. And after that, everything that is, the flow of information, what we were saying, we only have experiences generated in appropriate technologies for family production together with R&D departments and organisations. We are working on a process of innovation for agroecological transitions in as many production systems as we can. Especially in livestock, horticulture, and fruticulture. We are working in alliance with the agroecology network. In other words, we have knowledge and capacities that we are trying to bring back to the territory, or it is the territory itself that is generating them as its own agent. So, I think that as a union, we can also contribute to training processes so that people can prepare for these types of actions that are going to happen, both at the level of political negotiation as well as at the level of the implementation of concrete things in the territory. It's like this double game—the productive, but also the political dialogue—to come to an agreement and know how to lead an organisation democratically.

Interviewer 2: We have five minutes left.

Interviewee: Yes, my colleague is waiting for me.

Interviewer 1: Let's talk about the last question. They are about your experiences with climate change in the Santa Lucia River Basin. How do you experience climate change in the basin? Think about the changes in temperature, rainfall, droughts, or floods that have been recorded over the years.

Interviewee: Good. Well, I haven't lived in the area, and I don't do a lot of work in the area. So, I don't have accurate knowledge of what is going on in the area. But I do talk to people who live there, and I can get an idea. Anyway, it seems to me that what is happening in the Santa Lucia River is not very different from what is happening when climatic events occur in other areas of the country. So, I believe that one of the most sensitive issues in this basin continues to be the supply of drinking water for the metropolitan area of Montevideo. It has nothing to do with productive activity but, above all, with providing an elementary basic service to two million people. So, I think that this is where we have a very strong effect of climate change, in the sense that it can affect the quality and quantity of water in the metropolitan area. This is an effect of climate change because this drought that is happening now is almost unique in history. From what I've been able to analyse, three years in a row of water deficit is very abnormal behaviour; it doesn't usually happen three years in a row. This has been the worst. So, therefore, this drought also affects the productive area, mainly because of the lack of water. So, I think that this affects both the river area and others. Then, in particular, I will go into what the effect is on the Santa Lucia River. I'm not so sure. I don't know if this drought has not been an opportunity, perhaps, for what I was saying earlier, to expand the agricultural frontier in some places. I don't know, but probably yes. In general, when watercourses retreat, humans take advantage of this to gain ground, but I don't know if that's happening. And well, I think it should also be an opportunity for people to become aware, right? What always happens is that I have the problem identified, and I run off to look for a solution. I need water, I need a dam, I need food for livestock, and I need water to fix the crops. Well, maybe what we always talk about, and that's the big discussion I saw today, is that you have to get ahead of those kinds of problems. And climate change is here to stay, one that's going to get more and more, isn't it? And more frequent. So, I think that I don't know if this is what is happening in the area, but it should be seen as a threat or a weakness and as a future opportunity to start taking mitigation measures, right? And it surely has to do with using more efficient risk systems and better pasture management, in the case of people who work with livestock, to begin to take a little more care of the soils so that when the dry season comes, they have more capacity to retain water. And this goes hand in hand with certain practises, which I believe always provide opportunities for change. I know that in the area above the Santa Lucia River, a special effort has been made to promote these practises. More than in others.

Interviewer 1: And do you think that the implementation of these buffer zones can affect these climate change impacts?

Interviewee: Finally, the sensitive basins were the first where attempts were being made by people to transition to more sustainable practises, right? So, well, I'd like to think that that's happening in that area. It's not entirely clear to me, because what I'm telling you is that I don't work in the area. Yes, I may have a contact. I often go to Montevideo with my colleagues at the National Commission. At some point, it's going to happen that the river is going to flood its banks because it's going to rain a lot. One day, it's going to happen. And we also have to be prepared for that. And I think that the buffer areas seem to me to be fundamental there—to have natural areas that contain the overflows of the river and the different streams. And also to take care of the watercourses, because we know that with every dam that is built, less water reaches the river. So, this is also an issue of chaos. All these kinds of measures have been studied to go back to restoring degraded areas on the riverbanks. I believe in science and in things that have already been proven. We know that good agricultural practises improve the quality of the soil, decrease erosion, conserve water in the soil better, and increase crop yields. We know that substituting chemical inputs with biological inputs avoids water and soil pollution. I mean, I don't say it myself; the research says it, and we were able to do it; we were part of it, because many of the things that have been researched in agriculture and livestock, as a rural organisation, where we are most involved, we know that they give results. So my answer is yes. I believe that if we manage to implement these measures that we were talking about, the impacts of climate change would have to be reduced, and the production systems would have to be much better prepared for climate change. Production systems would have to be much better prepared to withstand them. In other words, building resilience I have a system that is going to adapt better to climate shocks, whether it's because there's a lack of water, whether it's too hot, whether it's too cold and it's raining a lot, or whatever the situation is. These are technologies that are proven or management measures that are proposed for buffer area restoration that are proven. So I believe in that, and in some cases, I was part of it; that is to say, I can say it with confidence in all the processes of innovation and transition towards best practises. I don't know; I believe in it, and I think it's very necessary to scale it up. In other words, it's not enough to have just a few experiences where things are done well or in this way to improve results and improve the climate resilience of production systems. It is time to scale it up. At least in the National Development Commission, we are already very convinced that these kinds of things need to be scaled up. So it is no longer just a matter of starting in pilot areas or with pilot experiences; at some point, to the extent that these results work, we have to scale them up. And these are also long-term processes. There are different people, different production systems, and different natural resources. But the processes, let's say, of implementation of good practises on the farms go hand in hand with long periods where the farmer can try them out, see the results, be encouraged, have someone to accompany them in making decisions, and have someone to measure the results. And say to me, "Look, I used to use such and such a thing, and I got so much product. For instance, now with the change, we are achieving 20% more production with less spending, which is often what ends up happening. They (producers, red) save resources on inputs and produce the same or more. This is what many of the experiences that have been developed say. It is not to say it to you, because there are all sorts of things. But in general terms, productive results have improved by implementing good practises that go hand in hand with the restoration of these areas. I don't get involved so much with native forests and wetlands because that's more the domain of biologists, but the vision is a transition between the systems. And both visions of water management and forestry are needed to make this transition.

Interviewer 2: So thank you very much for all the information and for all your shared visions and experiences.

Interviewee: Well, a pleasure and success with your work to both of you.

Interviewer 1: Thank you!

*Interviewer 1 is the student, and interviewer 2 is the supervisor.*

# Interview 6

**Interviewee: DINAGUA Date: 07-03-2023**

Interviewer 1: Buffer zones are strips of vegetation along watercourses. Their purpose is to capture and retain nutrients from agricultural activities to improve water quality. What do you do for a living?

Interviewee: I am the technical secretary of the commission of the regional water resources councils and the basin commissions, which are spaces that are regulated in the National Water Policy Law, 18610. It is an institutional framework for the participation of civil society. This is a constitutional principle that establishes that civil society, which in Article 47 of the Constitution of the Republic, says that civil society and users must participate in the planning, management, and control of water resources. Then, the National Water Policy Law, which regulates this article 47 of the Constitution, creates the regional water resources councils, of which there are three for the river-wide basin, for the UNAMERIN basin, and for the Rio de la Plata and Maritime Basin, which are the three large transboundary basins of the country. And in each of these regional councils, the river basin commissions are tripartite bodies made up of government, civil society, and users, aimed at building local, regional, or local policies linked to water resources. I am in the Technical Secretariat of the Regional Council of the Rio de la Plata and Maritime basins and the southern basin commissions, which are within this Regional Council, including the Santa Lucia River basin. I am a lawyer by profession. I am a specialist in land-use planning.

Interviewer 2: And in terms of organisation, do you depend on any organisation like DINAGUA, for example?

Interviewee: From the National Water Directorate That's where it is. The Technical Secretariat is implemented through the National Water Directorate of the Ministry of Environment (DINAGUA, red).

Interviewer 2: That's perfect; thank you.

Interviewer 1: The Santa Lucia River Action Plan introduced the use of buffer zones in the Santa Lucia River basin. The next questions are about your understanding of your organisation’s role in the design and implementation of the buffer zones. What are your organisation's responsibilities and objectives in the design and management of buffer zones?

Interviewee: There it is. Let me tell you, then, how we came to have buffer zones. During the decade between 2000 and 2010, the St. Lucia River was being studied. It was seen that there was a deterioration in the quality of the water, especially the Santa Lucia River, which is the source of drinking water for the metropolitan area. So, it is one of the most important and strategic watercourses in the country. So, the Ministry of Housing, Territorial Planning, and the Environment, at that time, today the Ministry of Environment, with Japanese cooperation, spent almost 10 years studying the causes of pollution in the country's rivers and the deterioration of water quality. The results of that study had been exchanged at the level of the environment authorities, which were not planning to implement measures to stop and reverse the situation of water quality. But in March 2013, there were episodes of bad smell and taste in the taps of the inhabitants of the metropolitan area, which was an event that had a lot of media impact. That put the issue on the public agenda and led to the rapid adoption of the measures that were being discussed on the basis of these studies that were carried out in the previous decade. This led to the adoption of what was called the action plan for the protection of the water quality of the Santa Lucia River." Parallel to that, in 2004 and 2009, the Constitution had been reformed; in 2009, the law had been passed; and in 2011, the regional councils had been created. The institutional framework linked to the areas of participation was being developed. In 2011, the regional councils began to function, and in 2012 and at the beginning of 2013, I started to work to set up the Santa Lucia River Basin Commission. So in March 2013, this happened to launch the organisation. So these events in March led to the creation of the River Basin Commission, something that had been brewing rapidly, and in June of that year it began to function with the draught action plan. With the action plan for the protection of water quality as the main component. These are emergency measures because, in the face of this situation, the bodies that were responsible could not fail to act, so these are top-down measures taken in the context of an emergency. The Basin Commission begins to function, the measures are put forward for consideration, and they are very well received by civil society and the users. But we wanted more; we were not satisfied. The first buffer zone, note that we are talking about 2013, was going to be established in the upper basin of Paso Severino, which is where the current water intake is, which is in the middle of the upper basin of the Rio Santa Luca, which is currently distinguished as zone A. This set of measures was established and was only regulated from 2015 onwards because the decree dates from February 2015. So, from 2013 to 2015, there is an exchange period. There is an internal discussion on how the ministry should regulate these measures. So there was a whole study, which was mainly carried out by Luis Reolón, and I think it is very important that he be interviewed.

Interviewer 2: Yes, I tried to send an email to him a few months ago, but he didn't answer me.

Interviewee: But he already gave you his mobile phone number? I write to Luis and tell him, "Look, I've just been interviewed; it's essential that they interview you. It's for research. I'll pass you his mobile; he's very busy. The ministry is very complicated, so he has a lot of work to do, and it's probably because of that. So, he did a study, and he's going to tell you much better, on comparative law: what are the experiences, what are the objectives? It was very detailed and very conscientious. And then there was the whole challenge of the legal and boundary part, from where it was drawn, because it was an encroachment on the right to property, which is very well protected in our country. It elaborated on how it was established in order to leave no doubts and give the greatest possible certainty (regarding encroachment on other legal grounds, red). One thing that I find very interesting about the evaluation was that this measure, in the end, was established in a ministerial resolution within the framework of the General Environmental Protection Act. This tool came out as a resolution of the ministry within the framework of the General Environmental Protection Act. It was also assessed that it could be a land-use planning measure, a tool that could be sanctioned by a land-use planning instrument, because the use of land can also be limited through Law 18.308, which is the Land Use Planning Law. But the relevant authorities were the intendancies, the departmental governments, and the Junta de Departamentales. They had to be instruments sanctioned by the Board of Departments. Santa Lucia has six intendancies, six departmental governments, and they were of different governmental parties. So, to reach a consensus and pass the same measure was a path that was incompatible at the time. So, they opted for a centralised resolution of the living and territorial planning at that time. That is because it is a ministerial resolution and not a land-use planning instrument. Well, there were exchanges with legal authorities on where the limits of the watercourse were (from where the buffer started, red). But that's when it starts to become a more complex issue for lawyers and land surveyors—a boundary issue. So in February 2015, that ministerial resolution was sanctioned. We intervened with a team from the National Water Directorate and a team from the National Directorate for the Environment at that time. Today, this is the National Directorate for Environmental Control and Assessment. Well, there was a discussion in the River Basin Commission about the dimensions of the buffers: that they should be 100 metres, 70 metres, and 20 metres. So, a working group was formed within the Basin Commission, which had the objective, first of all, to inform or discuss why this ministerial resolution had been made with this content. Therefore, it was possible to study comparative law, comparative experiences, and what the function was in those experiences. The function was to mitigate the impact of runoff and nutrients in the watercourse. They determined that 20 metres, 50 metres, and 75 metres were reasonable lengths. Because that was what was in that research, but for the protection of dunes, not with this function that we are implementing in Santa Lucia. So, like a first moment, it was to understand the tool and what the tool was about. And a question there is: what is the definition? Do you measure from the floodplain or from the watercourse edge? Because you designed an instrument that is not feasible for producers, at some point some producers are going to be affected by a potential reduction in their production. Of course, the thing is that the general environmental protection law gives it the power to determine these things; that's the convergence between private law and collective law. Of the general interest and the private interest. That's what I want to tell you: that the environmental protection law has important powers to limit the right of property in the general interest, which is the protection of the water quality of the St. Lucia River. I don't know what your opinion is, but well, I understand that in our legal system, let's say, there has been a paradigm shift with the last battery of laws that have been passed, where the general interest begins to impose itself over private law. I believe that in the first century that the civil code was in force in our country, it was particularly privatist. And the latest regulations, the ones that have been passed in the last 20 years, have made a change to property rights and made them more limited. This is very common in the city because, for a lot of reasons, your plot or your house is limited. It is not common in the countryside, and this is what is new: now that there is a general interest in the countryside, it starts to sort of become more prominent. So, in that sense, in this regulation, this ministerial resolution under the protection of these regulations that I am telling you about, they start to become concrete, and the buffer zone is one of the subjects. So this one thing that is very important is that at first the water quality problem measures were an emergency thing that was implemented, and from that point on, in 2015, that was the real implementation of the policy, and there was a discussion regarding how to implement this.

Interviewer 2: Your story is very interesting, especially from a legal perspective, which I believe is an important part of it.

Interviewee: After it was sanctioned, the group was created, and I will send you the minutes, Guillermo. These minutes detail the work that was done over the years, up to around 2018, in about five or six meetings. During these meetings, we discussed what the instrument (buffer zones, red) was about, how it was being managed, and the difficulties that the administration had in managing it. This made things more complex, as it involved investigation and decision-making as well as understanding the rationale and challenges associated with management. The river basin commission helped to articulate the discussion, and we supported the technical contributions. Through our discussions, proposals were made to expand the buffer zone, which were reflected in an instrument that was eventually included in the Santa Luca river basin action plan, as well as the second-generation action plan, which was sanctioned in 2019 but has not been implemented yet.. I have all the documents related to this, including the discussions, presentations, and exchanges that took place. However, not much has happened since then, and we are still at the stage where we have identified difficulties and made recommendations. We wanted to broaden the spectrum up to the courses, expanding in some places, like Arroyo de la Virgen, by 25 metres on the side. I also want to highlight a couple of interesting phenomena. Well, in the discussion, the director of natural resources was Mariana Hill at that time. At one point, the most resistant were the agricultural producers and the Ministry of Livestock. I listened to her in a meeting where she said, "I think it should be implemented in the whole country. Let's go to the whole country (national government, red) because it is a measure that is very important and it has to be protected. It's one of the most efficient in the medium term, but it's something that has to be done. So, I'm in favour of putting it in place in the whole country." Maybe she told you that she didn't say that, but I heard her say it. It is something that has to be done openly because during the period in which she was director—five, six, seven years—she pushed towards sustainable development and sustainable production, and a lot of measures were implemented, like land use plans. For me, they are a qualitative change in agricultural management. Several argued that it was a tool for the Ministry of Environment to get control because they had problems getting control regarding satellite monitoring systems and other things that were implemented, but they were only half-implemented. The land-use plans are the right tool to control this measure because when you control crop rotation, you control the compliance of buffer zones. So, there was a natural communion between sectoral policy and environmental policy. Parallel to that, I received a cousin of mine who is in a group of agronomists in the East, and he got in touch with us, especially my colleague, Amalia Paniza, who is the one who is linked to the basin commissions and the regional councils of the East and North of the country. She said that they aimed to incorporate it (buffer zones, red) from now on because they knew it was coming. It's like a tool that is not questioned, that works, and that is fine, so we want to get involved in this issue. So, I'll pass on the information. Roberto Lima, 33, an agronomist in the framework of the grouping of agronomists, was studying and learning more about this measure in order to implement it before it was made compulsory. This is rice farmers and big parts of the national economy speaking. So, I find that interesting.

Interviewer 2: I have a question that's been nagging me for a while. Before, was there any measure where you, for example, knew the measures were defined because the general interest was said to be held more important than the private interest or to be above the private interest? If I go over some strip of land, I don't know, more than 100 metres, is it necessary to compensate land owners? Or was it discussed if there is any point where it says I have to compensate the producers for the lost production or not?

Interviewee: That was never said. It was never held before the court. The subsidy policy is my responsibility, and the policy of subsidies, compensation, or expropriations is not there. Look, the former national director of water, Daniel Graves, said one thing that was a fact: Uruguay does not have a culture of property rights for reasons of general interest, and it is complied with and enforced. That's like the standard of our institutional culture. The same thing happens in the east; well, it's like a cultural issue of Uruguay’s environmental policy management because it is historically one of regulatory management; there are no economic incentive instruments, as the literature tells you.

Interviewer 2: Exactly. And is there something on this that you can give me? Interviewee: No. I don't have it; I never read it. Daniel listened to it, and I think it's reasonable, and that's it, but I've never read it, and I think it's good.

Interviewer 2: Neither have I; I wanted to make sure with you because that's the feeling I get. I work with issues of environmental economics where these types of incentive instruments are top-down, and in Uruguay it's difficult.

Interviewee: The former national director of water, Daniel Graves, said that Uruguay does not have a culture of property rights for reasons of general interest. The right to property for the general interest is above private interest, and it is enforced as part of our institutional culture. In the east, environmental policy management in Uruguay has historically been one of regulatory management. There are no economic incentive instruments, as the literature tells us. This type of instrument is down the sleeve of environmental economics, and Uruguay needs to implement a paradigm shift. For example, in the River Basin Commission, the most radical people say that the basin must be expropriated and turned into a protected area, like the Hudson River in New York. However, in Uruguay, this is not even considered. Neither incentives nor exemptions were foreseen. An agronomist named Amalia Paniza, who works with me, said that the producer is the first one who wants to take care of the soil and carry out sustainable management. They are the first interested parties because they want their soil to have the properties to pass on to their children and grandchildren. Producers are very interested in incorporating new practises and changing old paradigms of property rights for use, enjoyment, and abuse of the land. They are no longer in the business of using and abusing the land but rather want to adopt sustainable management practises.

Interviewer 1: What do you think are the current functions of riparian buffer zones in the Santa Lucia River basin?

Interviewee: Regarding buffer zones, their most important function in Uruguay is to act as a barrier to mitigate the transport of nutrients through erosion. It is only present in Santa Lucia and specific areas of Rio Negro. I am unsure if it is enforced throughout Rio Negro. Other functions of buffer zones and their characteristics that favour these functions are worth investigating. For example, the coastal defence strip is a buffer zone that is not catalogued as such, but it is regulated in the water code and a little bit in the law of territorial planning. With the buffer guidelines, it was modified because before it was a straight strip of 150 metres, and now it accompanies the coastal geography. It goes in a little bit and gets into the entrance of the mouth of the rivers at the La Plata river or in front of Maltimo. There, it varies a bit, but this is a buffer zone that functions as a kind of defence from anthropogenic action on the coast. The buffer zone protects the coast of the main watercourses in our country. Canelones also incorporated a buffer zone in the law on territorial planning and land use planning for all the courses that are sources of drinking water. The ban on tilling was implemented, which led to problems with buffer zone management because the lack of tillage led to weeds. For example, in the basin commission of the Laguna del Cisne, where they were discussing the buffer zone that was implemented by the municipality, a very humble producer started to cry because she said that she was worried that robbers would hide in the weeds and that they would steal from her. This was a situation of great anguish because of the bad design of the buffer zone. Then the whole buffer zone management was left pending, and dialogue was needed to exchange ideas and articulate the management of the buffer zone.

Interviewer 1: And what functions do you think buffer zones should have, ideally?

Interviewee: There are things that can be done in practise as they are being implemented, but the whole buffer zone management was left pending. To protect water quality, the function of the buffer zone seems adequate, and it fulfils its function, according to what the technical specialists say. However, it should be extensive and regulated. The issue of management should be studied and regulated. The loopholes or things that are more far-reaching should be preferred over other functions in that. The function is adequate, and the approach to the problem is serious. The tool is being used at its minimum expression when it could be much more powerful and could protect water harvesting. To protect the water harvesting areas, it is important to protect the springs and the water harvesting zone. Another thing is that there is a pilot programme in Santa Lucia that DINAGUA is implementing, which is also a buffer zone. This buffer zone is for the protection of water sources and should also be implemented for the protection of sources used for consumer use, irrigation, etc. It seems to me that this concept of a protection zone or buffer zone of watercourses for drinking water should also apply to water harvesting and the protection of the quality of sources. This concept is here to stay and is moving forward. Producers and the administration are seeing the need to use this protection because it also goes hand in hand with the law of territorial planning and the regulation of land use. This is of the utmost importance and has an impact on land use and the quality and quantity of water. So it is a cultural change as well. There is a technical change in the technical paradigm with integrated management and integrated visions of the approach to the territory. In parallel, there is a change in legislation. The law of territorial planning and the water policy law appear. And then at the individual level, at the producer level, the producer is realising that it is reality; you don't plant leaves to the edge of the watercourse, and he loses a few hectares, but he gains in water quality and in quality of other things, right? There are mentions of the regulation of the quantity of water flow.

Interviewer 2: Isn't that another function?

Interviewee: Yes, there are those who understand that water harvesting zones could have the function of regulating water quantity. But I don't get involved in that because I don't know of relevant (red)) studies. There is a whole group of people who have taken it to the basin commission and believe that there should be no forestry at the headwaters of rivers because the eucalyptus trees affect the absorption of water (infiltration). A meadow is not the same as a eucalyptus forest in relation to water absorption, which affects the water harvesting zone. And there is a very barren area. In short, what they are trying to do is protect the water harvesting zones with the function of recharge. One of the problems that existed was that of different visions; the first cries were that they were against this measure in Santa Lucia because they have small plots of land. So you would be practically annulling the right of ownership if you put in the buffer zone. But in the end, when they started to implement the buffer zones, the effect was not such; there was no problem with that issue, and in fact, there were no lawsuits either.

Interviewer 2: Well, it's good to comment that there because one of the hypotheses I had also was to check it if you can look at it with a map of patterns crossed with the buffer zones. Did you see that in the environmental observatory? There are those affected and there are those not affected by the buffer zone, and there I think we could make a percentage. A nice statistic could be what percentage of the population is in the buffer zone, and then we could order it and see how that number behaves. That would be interesting. You mentioned the water quality as the main and only function, and you also mentioned the two other functions, the management of the ecosystem and the infiltration increase. I think we can go on to the next question.

Interviewer 1: What do you think would be the benefits of these changes to buffer zones?

Interviewee: Well, regarding extension and management, I mentioned that nothing has an impact in the short term, but it is a medium-term measure. However, in the long term, it is very important that the buffer zones cover as many areas as possible in order to improve the overall water quality and quantity of the Rio Santa Lucia. The reservoirs directly associated with the intake are protected, but protection is only provided in the easternmost areas closest to the water intake. Unfortunately, the farthest areas are not protected, but if they were protected to their full extent, there would be a more profound improvement. Regarding management, there are things that can be done to improve the buffer zones. For example, reinforcing the buffer zone function through the cultivation of certain plants that not only mitigate but also reinforce the function of the buffer zone If certain weeds were prevented, it could strengthen the retention function of the buffer zone. Although these issues may escape me as a lawyer, I believe that implementing layers with vegetation would provide a greater advantage to the owner of the area and improve its use.

Interviewer 1: And what obstacles do you expect will arise when these changes are implemented?

Interviewee: There are obstacles. First, it is a challenge for the management, which is clearly not prepared for it. The Ministry of Environment does not have the resources to implement these measures, while the Ministry of Agriculture does. The biggest obstacle is clearly the ministry, not the producers. There are interests that are taking precedence over the general ones, and this is conspiring against the environment. There is a reason why they don't have the resources. The same thing happens with the newly created minister, who is given practically no facilities. So the money is going somewhere else. The political power doesn't care about justice or strengthening the judiciary. OSE has a lot of budget difficulties, and their budget is only a social tariff (non-privatised, red). While it is good that it is social, they don't pass on general revenues to implement the policies that have to be implemented. Therefore, drinking water and the protection of watercourses are not as important as they say they are. The budget needs to go to hiring technicians for the ministry, and the political power needs to care about these issues. The budget was 9 for housing and 1 for the environment and water when the MVOTMA was in charge (ratio of 9 to 1, red).

Interviewer 1: And how do you think these obstacles can be overcome, or what possible solutions do you think can be found to them?

Interviewee: In terms of solutions, I believe that with a transparent and honest country, these issues could be addressed with justice and fiscal policies. However, it seems that the political power doesn't care about these issues and that the agenda is not prioritising them. Despite the severity of environmental issues, the press fails to take a serious approach to them. It is as if it were a game, with the press only reporting one side of the story and ignoring the other. The treatment of OSE is also problematic, as they are not given sufficient investment. This issue is not whitewashed, and it is frustrating that opportunities are missed to address it properly.

Interviewer 1: How do you experience climate change in the Santa Lucia River basin?

Interviewee: Climate change has caused significant changes in temperature, rainfall, drought, and floods. It is a dramatic issue that needs to be addressed seriously. Although floods have occurred in the past, the severity and frequency of flooding have increased drastically in recent years. However, no measures are being taken to address this issue, and early warning systems are being developed to improve the situation. The Santa Lucia River is of particular concern, and Euroclima+ is a project designed to help manage the issue. However, the challenge lies in communicating the correct information to the public. For instance, there was a discussion in a watershed commission that blamed the OSE for not managing the flooding effectively, which the OSE quickly defused. Unfortunately, the situation is polarised, and it is challenging to change people's opinions. Environmental issues, particularly water issues, are highly polarised. There is a kind of complexity in the Santa Lucia basin, and the margin for change of opinion among the parties is very minimal. Of course, there are discussions as separate entities.

Interviewer 1: And how do you think buffer zones are affected by climate change?

Interviewee: There are no buffer zones that affect flooding, and flooding does not affect the performance of the buffer zones in the basin. I don't think that they aren’t affected at all. There are discussions. But it is not foreseen yet. You tell me that cities would miss the buffer zones, but that's not the case. It's not that the river overflowed; it's that man got in the way of the river. Every 100 years, the river has this behaviour, so it is mankind’s fault. In the case of Santa Lucia, there was a flood 100 years ago, and the river behaved like that. However, people built in that area, so today this happened. Then there is the issue of buffer zones in the cities: there is a tool called risk maps that they are trying to implement in land-use planning instruments, which is a very interesting tool for identifying high, medium, and low-risk zones. It is used in cities, and management is regulated on the basis of this. In cities, risk maps are a kind of buffer zone, but they are a regulation of land use depending on the type of risk, which includes other measures but is not implemented in all cities.

Interviewer 2: For example, are there risk zones within the cities?

Interviewee: In the upper basin, for example, there is no zone that says you can't do agriculture because it does not change the water flow. The buffer zone for agriculture is, as I told you, In the cities, there are risk maps, which are something else, but they fulfil the function of a buffer zone, of course.

Interviewer 2: I have another question: do you have the contact information for the director of DINAGUA?

Interviewee: Yes, I do; I will send it.

Interviewer 1: Thank you for your time. The recording will now be stopped.

*Interviewer 1 is the student, and interviewer 2 is the supervisor.*

# Interview 7

**Interviewee: Scientist        Date: 09-03-2023**

Interviewer: Before we begin, please know that you are free to refuse to answer any question or to leave at any time if you feel uncomfortable. Do you understand?

Interviewee: Yes, I understand. I will let you know if I don't want to answer a question.

Interviewer: Thank you. Let's get started. Do you have any questions before we begin?

Interviewee: No, I don't.

Interviewer: Perfect. What did you do during your professional career, and what organisations did you work for?

Interviewee: I worked for the University of the Republic, specifically the agronomy department, as a science professor. During my last 10 years, I had research projects focused on quantifying the losses of phosphorus from soil to water. While I didn't work directly with water resources, I did work on the interface from soil to water. I tried to develop best management practises to reduce the losses from soil to water, and then I retired in 2020.

Interviewer: Thank you. In what capacity did you encounter riparian buffer zones in this research?

Interviewee: Although I didn't work specifically on riparian zones, I knew that they were an important factor in reducing the losses from soil to water. I learned about the importance of buffer zones during my research, and I also learned that they could become a source of contamination if they were themselves contaminated. However, I never worked directly on this subject.

Interviewer: Thank you. In your research on soil and phosphorus, which different organisations did you collaborate with?

Interviewee: While I received grants from different organisations, we mainly worked on our research by ourselves. We had some contact with other organisations and had talks and presentations with them, but we mostly worked within our group. We had some collaborations with people from the University of the Republic but also from other departments in the science faculty, not only in Montevideo but also in Maldonado and Rocha, who were mostly working on surface water research. We also collaborated with people from the economy department and wrote a report trying to quantify the cost and benefits of the best management practises. We also had some projects to work on with seniors, but the project was continually postponed, and they called me a few months after I retired to say that the project had been approved but delayed.

Interviewer: I see. You mentioned your research on best management practises. What kind of management practises did these encompass?

Interviewee: We found that due to the no-tillage planting management practise, which is the practise that is used in most of Uruguay and involves not ploughing the soil but planting seeds and applying pit fertiliser on the soil top, the concentration of phosphorus (P) in the first centimetre of soil was incredibly high. We measured the differences in losses between areas under agricultural management and those under natural rainfall, and the losses were much higher in agricultural soils. We then ploughed the soil and found that the losses disappeared, were zero, or were less than those in the natural area. We also compared the differences between putting fertiliser on top or a few centimetres into the soil and found that the difference was huge too. We applied iron, ferric chloride, on the top of the soil at a low rate to see if the iron interacting with phosphorus on the first few centimetres of soil could reduce the losses. We were successful, and the results were clear. We arrived at a set of recommendations to reduce the losses, but these were contrary to common farmer practises. Farmers didn't want to change their practises because it was cheaper and more convenient for them. The other recommendation I have is time-consuming and much more expensive. So they never applied anything; they just kept doing what they were doing. So the authorities, for example, DINAMA or the military, don't have any power to do something different. You know, because all these practises go along with a very large territory, nobody could control that. There are not enough means to control that, or it will be very expensive to control that. So all my research is just information that is there, but some farmers that are more conscious are starting to change these practises and follow these recommendations and other people's recommendations. But it's a very small group, so there is no impact on the sources. I think that the sources are well-known, and some other people repeated my work because they thought that it couldn't be like that, and they got the same results. So they didn't see it; they didn't say anything to me; they just said that what I was saying was crazy or something like that. And then, when they did the same research, they arrived at the same conclusion. This is the situation, and I read about other places in which the situation is similar, like in the United States. Some parts of the United States experienced the same thing. It's very difficult to change farmer practise unless there is a very coercive set of missions or you put the police in the field.

Interviewer: During the interviews that Guillermo and I did for this research, we also heard about similar problems in the field of the implementation of these buffer zones. For instance, a large portion of farmers are not willing to adopt these buffer zones for a multitude of reasons. And that it is difficult for governmental institutions to nudge farmers towards more sustainable practises. How do you think that such a nudge can be made? How do you think that you can try to interact with farmers in a way that will increase the implementation rate of such measures?

Interviewee: I think one problem is that the people, in general, in these areas don't see that water as a threat. There were a few events that happened in the Santa Lucia River basin (flood events, etc.). I mean, one in 2011, I think, and another in 2013, and then there were no more problems. So they never had a real problem with the water. And what they think and perceive is that what we are trying to do is complicate their lives and make their work harder and more difficult. And also, for example, if a large dairy farm is going to be installed, it could bring more cows to the area. But if the government starts to place constraints, the farm will probably not be installed, and then there will be fewer cows available for the people. And they think that we are a problem for them, not a solution. And I think that the only way to advance slowly is through education. But there are no resources for education. It's only some sporadic efforts, and the people don't go. And they make a meeting, and only all the people go because they don't have anything to do with the people that are involved in agricultural tasks. They don't have time, and then they bring, you know, food and refreshments. And some people go for the refreshments, but it's not to work on the education process. They don't know what you are talking about, and they are also intelligent. Because if we go and ask something about the buffered zone, they will say to you, "Oh, it is very important." I know this, but they don't want to care about it. They want to use all the fields and farms that are helping to let us do research on their farm. They seem to know more than the average farmer about the problem, and they're more conscious. But they still have crops up to the water. So, I'm not going to ask them why they're doing that because they're letting me do my research. However, they don't follow any recommendations either. I think it's very difficult because the government wants to have good relations with the researcher on one side, and they act like they support us. But on the other hand, they don't want to fight with the farmer because they need their votes in the election. They want to be on good terms with both sides, so it's like a charade. Because the people in political positions want you to be happy there, and then they move to a different scene. It's very difficult. Maybe some people are more conscious and want to do more real stuff, but they are very few.

Interviewer: It's really interesting to hear this, so thank you very much. If you think any topic is relevant to discuss, there's no problem whatsoever with discussing it. I really appreciate your insights. Next, I want to talk about the functions of your perceptions of what the buffer zones attribute to. What do you think the buffers of riparian buffer zones are, according to your experience?

Interviewee: Well, it could be different things. For one part, it would be important to get the natural vegetation that grows along the rivers; that has native vegetation, and there are also animals that live there. People here used to know all the life and all the other things that were in the water. For example, in some meetings, some people told me that when they were children, they could swim there, and now it's empty. But these buffer zones could also be used for gathering firewood. So some people come collect it and sell it in the city. And this is one of the reasons that, in many areas, there is no more natural vegetation. It was eliminated, but I think that with the current agricultural practises, these buffer zones will be contaminated sooner or later. Also, the cattle are going to the water to drink water. So this is another source of contamination. And if I stay in this area, I contaminate the area with excrement. So the cattle should not be allowed to go there to drink this water. They should put a fence there to avoid this. In one area, they put fences in our reservoir, but the farmers cut the fence and nobody reacted. So my view is that we should maintain this natural buffer zone, but also before the buffer zone, we should put areas where the farmers can plant crops but not let animals there either, only for these areas that are also contaminated, going from the river up. In this area, only silage and this kind of stuff should be produced because they will remove the buffer of nutrients from the dry matter of the biomass that they have. In this way, there will be an area low in peak P near the water. I think this will be the best management, but this is my idea, and probably applying iron chloride in these areas could be added. Because it will retain the available peak and the leaching. The movement of phosphorus with water flowing down on the surface will be contained much less in the buffer. I think this will be a good management practise, especially in areas that have heavy contamination.

Interviewer: And what benefits do you think that such management practises would bring along with decreased phosphorus levels in the water? Is there also a way, for instance, to make it beneficial for farmers to implement it?

Interviewee: Well, these areas will give farmers another benefit. Not only farmers but also other people from the cities will go fishing and swimming, and the biodiversity in these areas will be very important. Also, you know, water quality for drinking water, because the phosphorus reduction in these streams will result in less phosphorus in the reservoirs from where cities are taking water for consumption. So everybody will benefit from this.

Interviewer: Okay. And if you had to express these different functions in characteristics that can be found in buffers, such as grassy vegetation or woody vegetation, or if you previously talked about fencing in these buffers, what kind of characteristics do you think can support those ideal management practises that you described?

Interviewee: I'm not sure if I understand you, but I think that the buffer zone near the river should be the natural vegetation that always grew there because these plants are adapted to the ecology of the region, and the animals that live in these areas are also adapted to this vegetation. Not change the vegetation; just replant these species there and let them recover these areas with the same vegetation that was there when the Indians were there. And then, you know, like I said, use another practise near the border of these buffer zones to reduce the impact of agriculture and the amount of pee and other nutrients that reach the buffer zone, but I think that this has a global effect, not only in water but in everything.

Interviewer: Clear.

Interviewee: I don't know if I answered the question like you wanted to.

Interviewer: Yes, you did. Thank you very much. In regard, I do have a follow-up question though, because you talked about that native vegetation is preferably the kind of vegetation that would be optimal for these kinds of buffers. In this you have any experience with invasive vegetation? And if so, what do you think the effect of such vegetation would be on the functioning of the buffer?

Interviewee: I know that there are some exotic plants that produce seeds, which are spread by birds, and then they put these seeds into these natural areas, and they start to dominate over the natural species. After some years, the native species disappear from there, and instead of a diversity of species that always grow together, you know, in these natural areas, some trees and shrubs always grow together like a group. They disappear and only these species from Japan or from other places start to grow. I never studied this, and the government knows that this is happening, and they try to eradicate these species, but the efforts are very small. It's more for the press than for real (combating exotic species, red).

Interviewer: Okay, then I have one question that is related to the different functions that you described for these buffer zones. So, for instance, biodiversity or the ability to go fishing—what of these different functions do you think is the most important?

Interviewee: Well, I think that the most important is that they reduce the amount of P that goes into the water because this is the more immediate problem, but the other functions, in my heart, I think are very important, that nature goes back there, and the water will not only be clean and transparent, but also there are animals there, different vegetation, and fish there. I heard that there are some areas that look natural but don't have as many fish anymore, probably due to chemicals such as pesticides. I was in a meeting where we compared fish levels and buffer levels. People from the science department showed that there is good water quality regarding sulphur in natural areas compared with areas under milk production. However, a man said he used to fish there until 1985, but then he couldn't fish anymore. We saw one aspect but missed the others. I'm not an expert in buffer areas, so what I'm telling you is just my feeling.

Interviewer: That's exactly what we're trying to uncover: the different perspectives of different people with different expertise in different fields.

Interviewee: You work in a more scientific or social science research field. Is this research an addition to Guillermo's doctorate studies, which are more social science-oriented?

Interviewer: My research is usually more naturalistic and scientific, but this research is an addition to Guillermo's doctorate studies, which are more social science-oriented. It's to gather more qualitative data on the views that underlie, for instance, the choice model that can be made for certain additions to these buffers. It's for my study programme in international land and water management, which is a combination of soil sciences, hydrology, and water quality sciences. Have you noted any experiences with changes in temperature, precipitation, droughts, or floods that you've noticed over the years that you've worked here?

Interviewee: This question is difficult to answer because I think that everyone is seeing changes, but they are not scientifically based. They are self-perceptions, sometimes saturated, but I don't want to do the same. What I noticed is in the data. For example, I was analysing rainfall during another type of research that I did, trying to select the best cover crop species. Everyone was testing a species that is used in the Southeast United States as a cover crop (legume, red). So we imported many species from Brazil and tested all these species in the same field, including the species that everybody else was testing. And we found another species that was much better than this so-called species that everybody was using without considering the ecological difference between here and the Southeast United States. I analysed the weather in four years of research in comparison with the national averages. In the four years, it was always higher than the national average. And I said, "Well, this is surprising because four years in a row, not in summer but in fall, it got warmer, and I don't know why. I am writing a paper about this research. I don't want to say anything about this being proof of climate change because, you know, it's a noticeable trend. They're always higher at this time of the year (temperatures and precipitation rates, red). Another thing is that all the agronomists said that here in Uruguay, every month has the same amount of rainfall as the long-term average. But after more than 10 or 20 years, farmers who plant late corn receive more rain. The rainfall in December and January is always lower than in February and March, which is not what people say is the natural weather in Uruguay. I was at a conference where farmers were saying, "No, we always start to plant late corn because there is rain." Well, this is not true because we have data from 1940. The farmer said to this researcher, "But now we are not in 1940, and it's always worked for me." Another thing I noticed is that during vacations in February, many people rent houses, and it starts to rain more frequently then. So I think those could be, you know, signs of climate change, but I'm not sure. I mean, I think that we should base our knowledge on data, not perception, and I haven't seen any real changes; maybe there are, but I haven't seen any real information, I mean, just data. Some people here, you know, that work in science, don't know what science is, really, because they think that if they say something, they are the authority, and nobody could, you know, doubt their assertion, and they say, well, I am the professor; you know, who are you? But this has nothing to do with science. Science is data, and one student could challenge a professor; he has new, more credible data, but many people don't know that. And if you tell this to them, they think, "Who is this crazy person?" I mean, real science is always controversial, and new findings are tested to see whether they are true or not under some variables. So, it's sometimes frustrating to work in science, especially in some other places in Uruguay where there is still a knowledge gap and scientific authority is not recognised.

Interviewer: Yes, and I think that's one of the main goals of this research. Before conducting these interviews, I did a climate data analysis on the river basin to see trends in temperature and precipitation, and as you noted, the significant trends are very small. Our goal is to compare how people think they experience these changes and compare it to the data, and eventually go to policymakers and show them the reality versus people's perceptions. This is social research that aims to shift the paradigm of thinking for these people. I have one last question. You based your findings regarding climate change on data and noticed that there are no large, significant trends noticeable. If these kinds of trends were to progress, how could they impact, for instance, the riparian buffer zones? In what ways do you think these changes can affect their implementation or effectiveness in the field?

Interviewee: I'm not really sure. Maybe they will change the species that are adapted to the buffer zone, but I'm not an expert on this subject. I think it's important to study what happened, not try to make a guess. Based on our guesses, we will miss the reality. In my culture, we call people who try to predict without good data "payadores" (clowns, red). I don't want to be one of them.

Interviewer: Do you have any questions or concerns that you want to share about anything that you find relevant?

Interviewee: I appreciate your interview and your line of work. I try to ask people and contrast their ideas with data, and I'm sure that you are much more advanced than us in the Netherlands. It could be helpful for you to take a look at our reality because people from outside are sometimes considered more serious than people from here.

Interviewer: Thank you, and I think it's also interesting to see that the problems we face in the Netherlands regarding communication between the government and farmers and the mindset of producers are similar to those here. It's nice to see the similarities and differences. Thank you very much for everything—for your time, knowledge, and input. It was a very interesting interview, and I really enjoyed it.

# Interview 8

**Interviewee: Scientist        Date: 14-03-2023**

Interviewer: During this interview, you're always free to not answer. If there's any point in time that you have to leave, you're always free to leave, so please do as you like. Do you have any questions regarding what we're going to do or what we do in general?

Interviewee: No, I think Guillermo will explain a lot to me; we've been in contact for the past couple years, so it's fine.

Interviewer: Perfect, thank you. Could you explain to me what you do for a living and how you are involved with riparian buffer zones in the Santa Lucia River basin?

Interviewee: Yeah, so I'm an adjunct professor at the University of the Republic in Uruguay. I coordinate a laboratory here in Paysandu that focuses on fluvial ecology, which has to do with everything from forest ecology to stream river ecology, fish ecology, etc. So my work is mostly research, and then I also do graduate and undergraduate training, teach courses, do some extension work, and stuff like that. In terms of riparian zones, I've worked in floodplains and riparian forests since I did my PhD on the Amazon River region. So I'm not an expert in anything that has to do with policies or how people should implement riparian forests. I'm more of a forest ecologist who's been working with all aspects of the ecology of riparian forests. So forest succession, forest regeneration, and how forests recover following some kind of disturbance And then I've been working with people where we do things like mapping of land use in riparian zones, mapping of watersheds, and things like that.

Interviewer: Okay, really interesting. What functions do you think riparian buffer zones have in general?

Interviewee: Well, so I give a class to my students on the function of riparian zones. So we talk about riparian zone function in terms of nutrient sequestration and nutrient absorption. We talk about riparian zones as modifying the microclimate and affecting stream temperature, temperature extremes, and temperature regimes. We talk about how the riparian zone also affects erosion rates and the maintenance of the stream margins. So it has a lot to do with the geomorphology of streams and preventing soil erosion. Then we also talk about riparian zones as habitats for fauna and flora.

Interviewer: Okay, and are there any specific characteristics of buffer zones in regards to vegetation types or management types that support the majority of these functions? What characteristics are most important to support these?

Interviewee: I was on the committee for a study that looked at how different vegetation covers affect these different roles. So one kind of mistake is that we might think that it's mostly forests that are playing a key role in all of these functions, but so far what the research is finding is that you can also have grassland cover, native grasslands, and obviously they don't behave exactly the same way as forests do with tree cover, but they do play a critical role in capturing sediments. So each of those vegetation covers can play a different role, but so far it's been important not to use necessarily one European model of 100% forest cover in all riparian zones as the healthy option. But we try to focus on that with our students and support research that looks at the role of native grasslands and shrublands, as well as forests, in playing these roles.

Interviewer: Are you familiar with the trouble that the Santa Lucia River has in regards to water quality problems?

Interviewee: Yes. I mean, I've been following it in the news, and then we have colleagues—many colleagues—that work in the Santa Lucia Basin. So even though we are pretty far away from that here in Paysandu, it's definitely a national issue that we're aware of.

Interviewer: Do these water quality problems, according to your knowledge, also spread further than the Santa Lucia River in other basins?

Interviewee: Yeah, perhaps not to the same extent or with the same impacts because there are so many people that depend on the water supply from the Santa Lucia Basin. Here you have people that do depend on other rivers and streams for drinkable and potable water, but it's probably not quite to the same extent just due to the number of people that rely upon the Santa Lucia Basin. But definitely, we see agriculture or different land use activities affecting water quality in all streams and rivers throughout Uruguay, just at different extents, different times of year, and different situations.

Interviewer: Okay. And when you look at the problems that the Santa Lucia River basin has in regards to water quality problems, mainly the overload of nutrients in the water courses, what functions of riparian buffer zones do you think should be prioritised in the design of these riparian zones in the basin?

Interviewee: So I don't speak from any experience having actually worked in the Santa Lucia Basin, and I know that it's definitely kind of one of the worst-case scenarios in terms of large basins that face nutrient overloading. So my response mostly comes from, I guess, reading the literature, because I don't have land there, I don't work there, and I don't know the system up close. But from what I understand from the literature, from Brazil, the United States, and Europe, the main focus in the Santa Lucia Basin is going to be establishing riparian buffers that are able to take up phosphorus, phosphate, and nitrates as well. And if they're going to be responsible for preventing more phosphorus from arriving in the watercourse, then those riparian zones are going to have to be able to capture sediments. There's going to be, you know, a prevention of erosion because phosphorus is usually transported with soil particles or sediment. So in that case, the literature says that you should have both forests and grasses if you want to capture sediment. In terms of the thermal regime of the Santa Lucia River Basin, I have absolutely no idea. We do some research here in the Uruguay River Basin on extreme water temperatures, and I have no idea what that situation is in the Santa Lucia Basin. Whether there is a lot of, say, warming of water courses that could contribute further to algal blooms So it seems to me like the most important role is creating that buffer of vegetation and soil microbes that are able to capture, sequester, and process these different forms of nutrients before they get to the water. That seems like goal number one.

Interviewer: Okay. One other problem that we hypothesise in the Santa Lucia River Basin regarding the implementation rate of these riparian zones is that farmers are not always willing to implement them for a multitude of reasons. For instance, that they lose productive land or that their cattle cannot longer access the water. What functions or characteristics of buffers could be potentially implemented to kind of mitigate this line of thought? How can you design buffers in a way that can also support other functions that farmers might find beneficial?

Interviewee: So again, I think that, like here, where we work in fisheries management, I have people that I work with in fisheries management, and most of the time, the answer to these kinds of questions really can come from the people that have to implement these changes. I don't feel as an ecologist or a biologist that I really have any authority to say how they might be able to implement these things because it really is a cost for them economically. Culturally, it might be something that people are not used to doing, especially with the drought that we're having now. Access to water is really, really important. So I mean, I feel like I could throw out some ideas, you know, about how to maybe work with agronomists to figure out how to better manage cattle in riparian zones where people could have forest cover and maintain forest cover and grassland cover and have low-density cattle ranching within those zones. But in Santa Lucia, I know that they ended up having an area where they just excluded cattle at 100 metres with a fence due to whatever densities that I suppose were high enough that it was still causing erosion and whatever undesirable effects the cattle had on the functioning of the riparian zone. I mean, how people are going to move forward and adapt to these kinds of new habits really has to come from the sector itself. The only thing that I can say is that I have seen experiences like those in the United States where people try to have buffer zones that also have some kind of economic benefit. It might be a zone where you can't plant crops, but you can plant trees that have some other positive economic impact. But you know, in Uruguay, the only thing I can think of is honey production. I mean, what lucrative activity can you have in a riparian zone that compensates for losses like cattle ranching, planting soybeans, or what have you? So, yeah, I mean, it's the big question, right?

Interviewer: Yes, it is. And from your perspective and your expertise, if farmers got the chance to change riparian buffer zones in whatever way they deem suitable for their own production while retaining the function of their original purpose in the sense of retaining nutrients, what characteristics do you think could be most important in these changes? What characteristics of the riparian zone would be most important?

Interviewee: I think we're just having a mix of different vegetation, so we know that grasses can play an important role. I'd have to look back at the data from different theses that have been done in the past couple years, but I think that it seems like a mix of forest cover, grassland cover, and native grassland cover would be important. There would have to be a recovery of soil structure. I mean, you can't just have the vegetation without having the bacteria that are going to be playing those roles. And then also, hydrology. I mean, if you have a bunch of dams and the hydrology is very altered, then let's say you have a riparian zone, but you don't have a period of inundation, then you're missing that kind of loop in the nitrogen cycle, for example, where you have bacteria that are going to be able to do denitrification during the anaerobic bacteria that are able to do that. So it seems like, yeah, I mean, it would be great. It would be ideal if you had a diversity of species. You could have species that also have some kind of economic value. I mean, native and exotic species are big deals in the Santa Lucia basin as well as many other regions. So as much as you could have riparian buffers that weren't dominated by exotic species, that would also be beneficial.

Interviewer: Okay, so from what I understand, there are three main functions that should be taken into account in this process. The nutrient retention, the soil quality, and the hydrology

Interviewee: I don't work in soil ecology, but I do have projects with people that work in the laboratory of soil microbiology or something like that. And it's just been fascinating, and they have courses on the health of soil. And really, it's opened my eyes because I've never worked with soil bacteria, mycorrhizae, plants, or plant-microbe relationships. And I've started working in that area with a student of mine who's looking at growth-promoting bacteria that are associated with native woody species. And it's just amazing—the diversity of organisms and the roles that they play. And Adriana Montanez is a professor who has a series of class materials and different tools online on this concept of healthy soils. And I think in the riparian zones, we tend to think that it's the plants that are doing all the work, but really, the soil microbes that are associated with plants, native plants, really have a huge role that they're playing, and we don't know a lot about it. So it seems like anything that we can do to kind of evaluate indicators of healthy soils in riparian zones would be important. And then that probably leads back to the question of, "If you have cattle in these zones and you have soil trampling or erosion in them, then what is the real role that this buffer is going to be playing? So it's a little bit outside of my area, but it seems like something really important to look at.

Interviewer: Yes, and regarding the importance of these different functions of nutrient retention, the construction of soils, and species diversity, If you had to rate them on a scale of one to four, with one being unimportant and four being most important, what scores would you give them?

Interviewee: We'll go one by one. What's the first one?

Interviewer: The ability to retain and capture nutrients

Interviewee: Yeah. Is number four the most important or the least important? The most, four.

Interviewer: And the construction of soils?

Interviewee: I would say four. Okay.

Interviewer: And species diversity, or biodiversity?

Interviewee: I mean, for management, I would say I don't have equivalent biodiversity. And just having native species. So I would say two or three. Okay. I think biodiversity is important, but it's not really the principal goal of the riparian zone. And when you have water that you can't drink, I think that having a burst forest is going to contribute to many of those roles, but it's not really the principal goal.

Interviewer: Okay. And if we also take into account the potential productivity of riparian zones for producers.

Interviewee: Oh, the potential productivity of riparian zones for producers—you mean for landowners or people?

Interviewer: Yes. And how important is that?

Interviewee: I mean, it's kind of a difficult question because I would say three because it's not really that important per se that these are highly productive zones. You know, however, that the productivity of the trees will directly influence the other roles. It directly influences nutrient retention. It directly influences the development of the soil because if you have a highly productive forest, you're going to have more production of detritus. So it's not unimportant. If everything grows faster, your riparian zone will recover more quickly.

Interviewer: Yes. In another interview, we mentioned that the potential productive use of riparian buffer zones might also be a positive reinforcement for the tree felling that is needed to retain the retention capacity of the buffer zone, for instance. So there are some interesting feedback loops that may occur in that process. The next few questions are about the potential benefits and difficulties of riparian buffer zones from a societal perspective. You already mentioned that your perspective is more on the ecological side and not so much on the policy or social side. So if there's any question that you think you cannot answer, please just say so, and we can move on. It's completely fine. If you think you have any input, it's always appreciated. What benefits do you think would be generated if these riparian buffer zones were successfully implemented in the entire basin?

Interviewee: Increasing water quality, better water quality, better aquatic habitats, and, I think, a better kind of basin-level management because people who are using water downstream are going to directly benefit from the management of riparian buffer zones upstream. So you could potentially have a better water source even if you're still in agriculture, cattle ranching, or what have you.

Interviewer: Okay. And are there any potential downsides that can be expected in the implementation of these buffer zones on a basin-wide scale regarding societal effects, ecological effects, or any other dimension that you deem relevant?

Interviewee: Yeah. I mean, I can imagine a lot just from having gone out with the forestry technicians here. I mean, when you have cattle, let's say that you have a riparian zone. I mean, if you had people sometimes referred to as "mugre" (muck; grease; filth; red.), you know, so I mean, whenever you have a riparian zone, especially one that's recovering and might not have like nice tall trees and an open understory, you can have all sorts of problems. I suppose you can have snakes. I suppose that if it's an area where you do have cattle ranching with high-density forest, I know that people have lost cattle in the forest. It can be more difficult to manage livestock if you have dense vegetation. I think, I've just seen a lot of, I don't know why access to water is such a big deal because most people have certain paths that they maintain to have access to water. So you can have a broad riparian zone and then just have a path that you maintain that's, you know, cut, for example, to make sure that there's easy access to a water source. Because you do hear people saying that, this is not the question, but another positive aspect of having riparian zones is that you have cooler temperatures and riparian cover of the soil and water, and the water table is higher. So some neighbours will say, "Oh, well, my neighbours are benefiting from the fact that I have riparian zones because I have more water underground than my neighbours do, which don't have forest cover, for example, in the riparian zone. So I mean, I think that managing riparian zones and closed vegetation brings its challenges, definitely, and that can kind of be a cost for people. But I think that a lot of people here, where we have a lot of forest cover in riparian zones, have kind of found ways of living with it.

Interviewer: Okay. You already touched a little bit on it, but do you think that there are any obstacles regarding the implementation of these zones?

Interviewee: Definitely. I mean, one would have more information and more local examples where people can go and see how other land users have implemented riparian zones, learning from other people that live, not from a biologist but from another person who has successfully been able to incorporate riparian zones into their own area, and that should be a teaching laboratory. I don't know what kind of economic incentives there are, if there are any. Something else that I've seen that is deterring is when one's landowner protects their riparian zone and then they feel frustrated because the landowner next to them, for example, cut the whole thing down and there were no consequences. So there can be incentives that encourage people to maintain riparian zones, but there can also be kind of the opposite, where they see that there are consequences or penalties for people who remove riparian zones. That's an incentive, too.

Interviewer: Actually, right now in the basin, regarding the management and control of these buffer zones, there are no financial incentives. There are financial penalties, but they're not enforced. So that is indeed one of the problems that hinders the implementation of these zones from a larger perspective, so to speak. Thank you very much. The next few questions are on a different topic that might also be a little bit outside of your expertise, namely the influence of climate change on buffer zones. Again, if you don't know or don't feel comfortable, there's no need to answer it. If you can, it would be perfect. How do you experience climate change in Uruguay? Or more specifically, if you have any experience in the Santa Lucia River basin regarding changes in temperature, precipitation, droughts, or floods over the years,

Interviewee: All of that Increasing temperatures, increasing frequency of heat waves, and longer periods of very, very hot days So this year, we started having temperatures over 36 degrees Celsius, like the first week of December, and today it's going to be 37 or 38 degrees, and we're in the middle of March. So we do a lot of research on climate variability, and we do use tree rings to study historical climate variability. So you can definitely see this in the records, as well as increasing precipitation, particularly in the summer overall, not this summer. And that's also related to increasing stream flow. So, for example, in the Uruguay River basin, two bridges have broken down due to very high discharge rates, and so one has been fixed and the other one's still broken. And then there's something that we've seen here that we thought only happened in places like Santa Lucia or the Negro River: algal blooms. We didn't have them; there's no recollection of anybody seeing algal blooms in the Uruguay River basin. And then just last year, during one of the major heat waves where we had those huge fires that swept across Uruguay, we had record temperatures with a daily average water temperature of over 30 degrees. And so, all of a sudden, there was an algal bloom. And some of those species can produce toxins. So we're dealing with a kind of interaction between climate change and the fact that we have all these other impacts on streams and rivers that have to do with land use. So what's most prevalent in people's minds right now is probably the drought, and, you know, there's a whole discussion about what percentage of the drought is related to just regular climate variability. We've seen other periods of time—you know, two or three years of drought like in the 40s. I am not an expert in this area either, but I know that I have read that this drought in particular could also be related to deforestation in the Amazon, but I have no idea if that’s true. But there are kind of multiple hypotheses out there about this lack of humidity, which could be related to the greenhouse effect or to natural variability in climate. It could be related to a lack of moisture coming from other regions that are undergoing deforestation. So we feel climate variability in many ways. And part of that variability is due to climate change. So and then the ways that we feel it are directly through these different, you know, climate scenarios, but also how it really affects riparian ecosystems and rivers in terms of stream flow and water quality.

Interviewer: That's a really nice explanation. Regarding these events or trends, so to speak, these developments, how do you think that these developments will affect buffer zones, if in any way they do?

Interviewee: I mean, it's very interesting because you have all kinds of research where people are asking if, you know, increased carbon dioxide will increase plant growth. So and then the interesting thing is that when we have these fires here, I mean, the fires were mostly in pine plantations and in eucalyptus plantations. And native forest was a buffer for the fire. I mean, I think that in terms of climate change in Uruguay, we have a climate history where Uruguay was relatively drier in the earlier part of the 1900s and has transitioned to, you know, a slightly wetter climate. So, you know, I think on the one hand, you have tree species and ecosystems in terms of riparian ecosystems that, if they've evolved in this climate scenario in Uruguay, are pretty resilient, and we do see that in the growth, you know, that dry periods can prevent tree growth. But with species like Algarrobo (*Ceratonia siliqua*), the flooding is actually detrimental to growth, and the dry periods favour growth because these are species that are adapted to these kinds of soils that can dry out very quickly. So, obviously, when we have extreme storm events, extreme flooding, and drought, All of those can potentially negatively affect these systems. But if you have intact forests with relatively diverse species, because it's not as diverse as all the Amazon river basin, you do have an interesting level of diversity. The forests have a potentially really important role to play in terms of climate variability because they buffer extreme temperatures. They're potentially preventing fire spread when there are fires. They can actually buffer against really cold temperatures in the winter. So I wouldn't necessarily place them as one of the more vulnerable systems with respect to the climate variability that we have now. On the contrary, I think that they'll be really important. And potentially, with increasing precipitation, you might even see more favourable conditions for, for example, forest expansion, which is good or bad, depending upon who you are.

Interviewer: Yes. You already touched a little bit on it in an earlier question. But how do you think that invasive plant or animal species can affect the functioning of buffer zones?

Interviewee: Yeah. So, well, if you have a monocrop, like here in the agronomy station where I work, there are sections where you have a riparian zone with trees, but they're not necessarily providing habitat for a diversity of species. There's a hypothesis that they can potentially alter soil chemistry, which can deter other species from regenerating. And it's really hard to manage in terms of the ecology; you don't really have a diversity of species that are able to, you know, play multiple roles at different times of the year because it's kind of dominated by one exotic species. And then the other role is that, while you only have one species that flowers at a certain time of year, in terms of, you know, honey production, that's pretty important here in terms of riparian zones. And you're going to have a big part of the year where there's no honey production because there are no flowers. So I think exotic invasive species are really a problem, both ecologically and economically, unless I'm mistaken.

Interviewer: Okay. And regarding the management of these species, is there any way to control their existence in these riparian zones?

Interviewee: Yeah, so that I've just been able to observe other colleagues and people that work in management trying to control, for example, *Gladistia trianthos*, which is from the United States and from the Mississippi River Basin, which is one of the more problematic species in riparian zones here. So they've had success with having goats to control regeneration, and they're able to chew the seeds, which prevents the germination that happens when you have cattle, and cattle swallow the seeds, and then they all germinate in the cow pies. So I know that they've had some reasonable success with managing goats. There are a few examples of using girdling and then treating these exotic species with an herbicide. That's had some good results, but it requires a lot of man hours to physically go to the forest and be actively girdling the trees and then apply pesticides in a way that doesn't introduce pesticides into the whole environment but directly affects the specific trees. But it's just—I mean, you're up against such a high rate of fertility, development, and propagation.

Interviewer: Okay, sure. Then one last question, which is about this management, How do you think this could be managed? Who should be able to do these practises? Is it something that the government should take upon itself, or is it something that the farmers themselves should be involved in?

Interviewee: I think that what I've learned from living here is that it is interesting when either the government at some level, whether it's the Ministry of Environment or the Ministry of Agriculture, I mean, it is interesting when the government is able to provide examples or programmes that support innovative ways to treat exotic species. I have also seen subsidies where they are encouraging people. I think in Braveda, this would be Beatriz Sosa, but I think they have a project where people need fuel wood. I can't remember if it's actually government or not, but it's a project where people are incentivized to go cut down exotic species, and they're somehow paid for that. But on the other hand, you have extensive areas where the government doesn't really have a huge presence. And so in that sense, you really need both the government and landholders working on this together.

Interviewer: Okay. That was my last question. Thank you so much. Is there anything that you want to share? Do you have any questions at this point in time? Interviewee: So you're doing your master's or PhD, or? Interviewer: I'm doing my master's at this point. And I'm helping my supervisor because my thesis is a contribution to Guillermo's PhD.

Interviewee: Okay, perfect, perfect. And your master's is in environmental science?

Interviewer: It's in international land and water management and climate studies, different masters. Interviewee: Okay. Great. So, well, it would be interesting to see what comes out of this study. I think it's really important.

Interviewer: That's good to hear. Thank you. I also hope it's going to be interesting, and I hope that it can contribute even a little bit to the potential re-instigation of the importance of buffer zones in the river basin. Because Guillermo and I went on certain field visits, it became apparent that the implementation rate wasn't that high anymore. And also that from the river basin commission, it's kind of the topic that's taken back to the background because other things like the drought and the flooding a few years ago were more prevalent at the time.

Interviewee: Yeah. Exactly. Interesting. Very good. Great. Well, we'll look forward to seeing the results.

Interviewer: Yes. Thank you so much for your time and for your input. It's really appreciated. I will stop the recording now.

# Interview 9

**Interviewee: Scientist        Date: 14-03-2023**

Interviewer 1: Buffer zones are strips of vegetation along watercourses, and their purpose is to capture and retain inputs from agricultural activities to improve water quality in the Santa Lucia River basin. What do you do for a living, and how are you involved with buffer zones?

Interviewee: A long time ago, we did different research approaches to buffer zones, analysing satellite imagery and doing some exercises with different widths of buffer zones to propose management means in Rocha. We have even done experimental plots where an experimental design has comprised a part of sown pasture and another part of natural grassland vegetation as buffer zones. So we had mixed agricultural control, grassland control, and treatment. It had both coverages, so we could see what nutrients (red)) were circulating in the stream. We observed what water was coming out with what concentration of nutrients. We saw the effect of the buffer zones directly; it was not an experiment that went well at all; it was very short; we had some problems with the quantification of the volume of water flow; and we didn't have the money to continue with the experiment. It's a pity because the results are good, but it was only six months, and we couldn't continue. And then we worked more with others, which was also a challenge. For instance, in the Paso Severino, there is an artificial rain simulator, which is a small device that I think is Dutch or Danish. What we do there is come up with artificial rain that generates runoff, and this runoff is collected and analysed. We collect it, and we analyse not only the volume of water but also the nutrients in that water. So it's very easy to handle, and in a short time, you can have a lot of data and be able to compare different coverages. So, we had a design that we did for an agreement with the Ministry of Environment, which was to carry out simulations upstream in agriculture and immediately inside the buffer zone. Of course, the results are very interesting, but there is no direct relationship between the samples we took upstream and downstream. The analysis wasn't like the one where we used the same field and you knew that the water that came out was actually the water that had passed through the "zona baja". Then we also had groundwater collectors in that project that had the same design, and we collected them in the same way. We collected them, we installed them in the agricultural zone and in the immediate buffer, and we looked at the outflow of water, which also had very interesting results. But we can't be sure that the water that was coming out as outflow was coming exactly from what was in agriculture. And then we continued to work with the rainfall simulator, comparing different natural and artificial agricultural covers, and now we continue to do a lot of things with the simulator because it is very cheap and very easy to generate data. It's not the best experimental design, but it's what we can afford with the projects and the funds we have. So I have quite a lot of experience, and last year we worked with DINAMA on this. So all this work was done to reduce the livestock footprint, and we worked on the buffers.

Interviewer 2: By farming in natural fields as a way to capture nutrients.

Interviewee: Yes, and as well, natural fields have a smaller footprint regarding nutrient levels, not only for us but also for small farmers. Then we also did some other models; we have worked quite a lot with some models, including the one that focused on ecosystem services. If you want, I can pass you one of the papers that we have.

Interviewer 2: Yes, very good. Interviewee: But I have to check to see which ones I have and which ones I don't have.

Interviewer 1: Yes, great, and what organisation do you represent?

Interviewee: All this is within the framework of the university (Universidad de la Republica).

Interviewer 1: Perfect. There are many different ways to design buffer zones, for example, by using different types of land cover and growth strategies. So, there are different ways of defining these buffer zones. The next question is about how to define the current buffer zones. What functions do you think the buffer zones currently have in the Santa Lucia River basin?

Interviewee: The definition of buffer zones is always a natural transition zone between land and water bodies, but in reality, buffer zones can be interspersed with other types of vegetation. For me, the idea would be that every time you have a crop, its transition zone or crop boundary would have buffers in between. You could always have the entirety of the slope interspersed with the buffer zone, and that could be forests, pastures, or whatever you want, and that would be much more efficient than waiting here for a buffer zone of only 20, 30, or 50 metres along a watercourse. There are some studies, but not many, that show that when you have strips of vegetation interspersed with the crops, it is more efficient in retaining nutrients than when everything is limited to one buffer zone around the stream. To me, that would be very healthy if you had buffers within the crops or on their vegetation boundary, beyond the fact that streams should maintain a buffer zone around the stream. This is for many reasons, first of all because the buffer zone is often elusive in the stream. It is dependent on the intensity of agricultural use, and because of that, it is also going to be the last filter that this body of water is going to have from what ends up in the stream, so for me, the design should incorporate strips of vegetation inside the crops. Buffer zones are most important at the headwaters and in small streams, as large streams have a greater surface area, agricultural use, and nutrient arrival at the watercourses. Once water has reached the main watercourse, a buffer zone is not necessary, as the water has already been reached by the springs and smaller streams. However, there are complex issues with the use of buffer zones in other places. I believe that buffer zones do not have to be complete exclusion zones. In fact, a master's thesis conducted by a student with us in Paso Severino showed that buffer zones can also be potential sites for nutrient export, particularly nitrogen in the form of ammonium and nitrate. This is because the biomass of herbaceous plants in the buffer area, which accumulates nutrients such as nitrogen, decomposes and releases nutrients almost as fast as what is brought to agriculture, especially nitrogen. Well, I don't remember whether it was ammonium or nitrates. So for me, the buffer zones have to be managed, especially in Uruguay. And then I also think that buffer zones have to have management adapted to the environmental realities of the ecosystems and the productive uses of each locality. So in Uruguay, where livestock production is the basis of everything, I believe that buffer zones should be managed with livestock at certain times of the year. That is to say that you put a high load of cattle, I don't know, once or twice a year to reduce the height of the vegetation and remove nutrients, and, well, some dung is going to be left there, but they're going to remove some of the biomass. And you prevent all that biomass from decomposing and ending up dissolved or associated with dissolved organic matter. That could end up in the water as well. So you need to not only install buffer zones, but you also need to have a mechanism for extracting the nutrients that are in those zones. Because those buffer zones are permanently receiving nutrients from the upstream zones, the nutrients don't disappear. They are associated with the soil, or they are associated with the biomass, or they leave in a particular form, or they leave in a soluble form, but they go somewhere. Except for denitrification, which is the only process of loss, denitrification is the only process of loss. So, we need to do some management. This management has to be well thought out and designed with the objectives of the buffer zone in mind, not the productive ones. You can't put cattle in the buffer zone when it's wet and muddy or during heavy rainfall. Because of this, you have to wait until the soil is firm so that the cattle don't trample on it, which is a problem of cattle trampling, and you have to manage it in a certain way. You could do it with machinery, but it's not practical and it's expensive. For me, there has to be intelligent management of livestock in the buffer zones. This could be once a year, once every two years, or twice a year, but we will have to assess and test that. There are ways in which you can have cattle without them grazing in the buffer zone. For instance, you can put mobile watering places and shade there, so the animals will go there to drink and eat. They will go to the most comfortable place, which is where they have access to better-quality water. They don't like to go into the water so much if they have a better supply of water somewhere else. So they can leave the buffer zone to rest. However, you don't have to define or decide what the best way is. There's another issue to consider: if you have a buffer zone with a lot of vegetation, especially grassland, the cattle start to circulate along the wire fence trying to eat it, which ends up generating some current grooves that during the runoff generate more erosion, and in the end, the water avoids the buffer zone. So you also need to have some kind of strategy for that. Sometimes, when you don't cut the vegetation, it accumulates and generates a cushion of grass, causing the water to circulate along the buffer zone until it finds a place to exit and ends up in the buffer zone. Studies show that most of the nutrients are lost in places where preferential currents avoid the buffer zone. Therefore, you need to have fine management of the buffer zone, which I believe can be done more efficiently by using livestock in places where it's possible, which is 90% of the country.

Interviewer: What function should the buffer zones have in the Santa Lucia river basin?

Interviewee: Well, for me, the buffer zones fulfil the primary function of reducing the nutrients and sediments that come from more intensive land uses, such as agriculture and pastures, and to a lesser extent, the natural countryside. Then, they play a role in protecting the native zone of the watercourse from erosion and other factors. Additionally, they have an extremely important function, which is to provide a refuge for biodiversity. If managed properly, they can fulfil several functions. Furthermore, there is another function for me, which is aesthetic. The aesthetic part of the landscape also fulfils a function. In terms of recreation, you can also go for a swim in a stream in the summer, which you can't do today because agriculture up to the edge is not going to make you feel like getting into a stream or going fishing in a place where you are among the leaves. Maintaining a buffer zone not only protects the water quality of the course and maintains some biodiversity, but also gives a bit of life to the people. For the Severino Pass reservoir, we insisted a little bit on this, although I didn't ask the ministry to do so. It was the only place with some recreational attraction for a good part of Florida, where everything is agriculture and where the forests and the littoral zones of the streams have been razed to the ground on the basis of artificial pastures. They have a social function in an area that has nowhere else to go. The buffer zone would no longer be just a matter that farmers have to do to not pollute your water; it has another important social aesthetic function and value. It would have much more support among the people. That is another important function. There are also negative functions, such as being corridors for invasive species, which is a significant problem in the Paso Severino reservoir. If you don't have livestock, invasive species are likely to thrive. With grazing, you have them more under control, but it could be a problem. But with the Fresno (*Franxinus lancelota*), it is a problem, and surely for other species it could also happen for the Ligustro (*Ligustrum lucidum*), in many places, and for the Gleditsia (*Gleditsia triacanthos*) and for other herbaceous plants. Another function that I was going to mention but forgot about is ecosystem services or functions.

Interviewer 1: And how would you rate them on importance from 1 to 4, with one being the least important and four being the most important?

Interviewee: I think the order in which I named them is the order in which I consider them. I would perhaps put the social function in second or third place because in places that are very agriculturalized, like really, really agriculturalized places, it's a very ugly landscape, isn't it? I think it would fulfil an important social function. It would need to be worked on, so I would move it forward a bit more. I mean, you could give nutrient retention a four, watercourse erosion protection a three, biodiversity a three, and aesthetics at the same level as these other two (a 3, red). For me, aesthetics are at the level of biodiversity. Having only buffer areas is like having a cemetery for biodiversity. On the other hand, in some places, having sites with a little more natural vegetation for recreational use can be important. Maybe I would put biodiversity in fourth place and aesthetics in third. Then there's the whole issue of pest control and advertising, right? Well, you have all that covered, no doubt. These insects can be kept inside the buffer zone. They can nest and shelter in the buffer zone, and one of them can even be in the middle of the artificial field. In some places, if you use strips of vegetation interspersed between crops, you could have a higher value because you could cover a larger area. A strip of buffer zone in 100 hectares of agriculture is the minimum contribution it can make to pollination. If you have another interspersed strip, you have another support. I missed the one on corridors for exotics. It is the one that is important for Santa Lucia. You could give it a value of three; in some places where you already have problems with invasives, it could be a game changer.

Interviewer 1: The next question is about the implementation of these different features or functions. What would be the benefits of these changes to the buffer zones? You mentioned a lot of things that are different from how buffer zones are applied today. You talked about management and buffer strips that can be interspersed with agriculture, and that's where the questions come from.

Interviewee: Well, it seems to me that one of the major benefits is that you will have greater retention of nutrients and less export to the waterways. That's the main advantage. If you have buffer strips intercropped with the crops and you increase the area ratio between the surface area of the crop and the surface area of the buffer strips, which is an indicator that shows nutrient removal is more efficient with a lower ratio of crop surface area to the surface area of the buffer zone, you would be improving nutrient retention and removal. With management, you could increase the removal of nutrients from the nutrient system, or at least from the manure, by moving it away from the water and eventually using it for re-fertilisation. But for the time being, there are nutrients. Instead of getting into the water right away, they would always be moving away from the watercourse. Agroforestry often promotes diversity in managing loads and also reduces the risk of invasives, especially with trees and many herbaceous plants in livestock farming, which control dispersal and maintain populations of invasive species. So, these management measures for buffer zones would also be important. Yes, I think the biggest benefit is that you're going to reduce nutrient inputs. You're going to make the buffer zones more efficient. To me, that's the most significant advantage. Then, if you look at it from a more biocultural or sociobiological approach, thinking about the other cultural, social, or reiterative values that can be generated by the buffer zones, what you're going to generate is less resistance to the implementation of the buffer zones or more support for them. Greater social support for the implementation of the buffer zones will reduce the resistance that producers have. Producers have a lot of resistance due to the increased costs and complexity of farm management, and above all, what they like the least is someone coming to say what they have to do, which is what bothers them the most. After they understand and see that the cost is not too high and that there are other benefits, they become more receptive. But what they like the least is someone from outside telling them what they have to do. So, if you have more social support because the workers in the locality see in the buffer zone an incentive to determine things that enrich or improve, you will have a counterbalance to reduce the resistance of the producers. Sometimes, producers are unaware of certain things. It happened to me, for example, while working at Paso Severino, that people would say, 'Ah, down there we used to camp with my grandfather, go fishing, and bathe in that place, and that's the place where very rough farming is now.' And the guys never sat down and thought that, well, the truth is that we could have been farming until a few metres before, and today we would still be spending the afternoon there, wouldn't we? If you put the issues back in the buffer zones, I think there would be less resistance because even they themselves would realise that it would be good to keep the small shallows where they went fishing as children in a more sanitary state. However, when you approach this from the angle of cultural and social valorisation, they respond differently compared to when you focus only on the utilitarian aspect of reducing contamination, which can lead to contamination.

Interviewer 1: And what obstacles do you think there will be when these different features are implemented? Well, for me, there are three obstacles that are very clear. There is an economic one, in that making wire fences is very expensive. Although electric fences are cheaper, they are still very expensive, and you have to do a lot of maintenance. So what you save on the installation, you then have to invest over time in maintenance, which is something that people are not aware of. For example, the environmental mystery is not aware of how expensive it is to make fences and how expensive it is for a producer. Even for a not-so-small producer, a medium-sized producer, or a large producer, it is expensive anyway. The other issue is the change in management of the farm, which implies another variable. Whether I am going to put them here, whether I am not going to be able to, whether I keep them, whether the cattle do not go to the buffer zone, which is something else, and really the traditional farmer who does cattle ranching has less; he has simpler management, unless they are very organised producers, Like these new natural pasture producers, who manage the loads and categories to improve the natural pasture, which are few, the others basically keep cows and go every now and then to see how they are, so everything that involves buffer zones implies not only wire fences but also open drinking troughs. The water troughs have to be maintained; they break; the cows break them, so it is not only an economic cost but also a management cost in that you have to do more things. You have to be aware of more variables, and that is another thing that many times Montevideo does not understand or the institutions, especially the environments, do not understand. The part that the minister would understand the most, and well, on the contrary, they get a bit paralysed because of that, but the environment minister often does not understand that for the producer, it is an extra management that implies work, not only money but work that sometimes is not so easy to do, and then the cost is the cost of decisions, and the cost is above all the resistance that the producer has to the management. So there is a very big resistance, and for me, one of the biggest mistakes made by OSE and the Minister of the Environment with their severe steps is that they did not do any kind of work with the producers. That is, they went one day and put up a wire fence; you can't work like that, even though the land belonged to OSE and people had been using it for 30 years. You can't just go one day and put up a wire fence; you have to work with the producers and explain to them the importance of things. They don't consider that the impact of the water quality is their responsibility, so you can't just make a measure obligatory one day and not the next. You have to work with the producers, and the producers in Uruguay are very organised because they are all linked through some rural association or some group; it is not that they are all loose around. They are very organised, so sometimes it is not so difficult to start working with producers; of course, you have to put people in who know how to work with producers. I am not a biologist who works in a laboratory; to work with producers, you have to work with extensionists. I believe that some of the proposals are working in a much more interesting way in SAUCE than in Paso Severino. We interviewed the people of Paso Severino; I can pass them on to you as well, and they hate the buffer zones. Because they were imposed on them, they don't even have any reason to hate them, but they don't like the fact that a wire fence has been put in without anything, without having spoken to them first, without asking. I remembered the other non-function of the buffer zones, which is the accumulation of dry matter that can generate fire risk. A producer came out the other day, and they raised the issue of fire risk in Paso Severino because there are some dry grasslands and so on, and if some capybara hunters or fishermen go and have a barbecue and set fire to them and set fire to the wire fence, it's not going to generate a forest fire like in other places, but you've used up the wire fence so they can set it on fire. You have to work with extension people and extension agronomists who are committed to or convinced that the buffer zones are good and useful and that they work. I think that in the OSE, they are working in a much more interesting way than in Paso de la Plata.

Interviewer 2: It's good that you brought that up; that was brought up by a producer the other day. They raise the issue of fire risk in Paso Severino a lot because there are some dry grasslands, and it is a question of some capybara hunters going there, some hunters making hunters go and have a barbecue, and everything catches fire.

Interviewer 1: How do you think these obstacles can be overcome? What possible solutions do you think can be found?

Interviewee: For me, the solutions are to have long-term policies, so that Paso Severino is not, for example, an isolated issue, but rather that you have a long-term policy with a ministry and that you work in an articulated way with the other institutions, with the ministry, with the other institutions, with the Ministry of Livestock, with the municipalities, with the corresponding strategies, and that you hire the personnel that are doing this work with the producers. And that there is a follow-up on the buffer zones. And in addition, the other strategy for me is always information. Many producers always tell us, "Look, if you have to do so many things, tell me that what I am doing is helping someone. If you tell me the water is getting better, then I'll continue. Now, if I am doing all this and they are not really serving the buffer zones, or if I see that there are others that are not complying, why would I do it? So, I don't do it. So information is extremely important, especially scientific information. So much money was invested, but only so many nutrients were removed; the quality of the water is the same, it is not golden, or it is slightly improving. So they want information, and when there is information that says that things are working or that explains exactly how things are, people are willing to listen, and they're willing to make improvements. And after all that, comes the financing. There are certain facilities to determine things where there is a subsidy given by the state and another part that the producer has to put in. So, there was a part that the state put in and the producer also put in, but well, he didn't put in 100%, but that's not what they demand most in terms of money. What they want most is to be able to participate and be involved in the decisions that are made, to be given information, to be explained, to be shown the numbers and the data, and to be shown that things are working. And then they ask for some money, but not much. They are willing to put in money with the collaboration of the state, but it's not like they want everything to be done for them because it's not solely their responsibility.

Interviewer 1: Okay. And how is climate change being experienced in the Santa Lucia River basin?

Interviewee: Well, it's not a subject that I have studied. The sub-basin of the Santa Lucia Chico was already dried up, and there was no water left for five days. So we are experiencing it like the rest of the zones. The problem with the zones is going to be climate change, in which the variability of extreme events is what's going to make the buffer zones less efficient. So, having an extreme event where it rains 150 millimetres in an hour or two hours tremendously lowers the efficiency in the zone, which is designed to work when you have a relatively slow circulation of water, giving it time to slow down the speed of the water in a certain process. If the water flows through the zone too quickly and in too large quantities, the zone will not be able to perform its function effectively. And if there are extreme flood events in the rivers where the watercourses pass over the top of the zones, then we will have to consider what the long-term function of the zone will be. Obviously, if the river passes over the buffer zone, whatever is in the zone will eventually end up in the river in an unrestricted way. So, if extreme events become more frequent, the efficiency of the zone will be reduced. Therefore, wider buffer zones are needed, which is what the biography is proposing. These buffer zones will be interspersed between the trials so that they have a greater capacity to retain the land uses and not just lose that strip of zones at the river.

Interviewer 1: How do you think these changes will affect the performance of the buffer zones, as you proposed in principle?

Interviewee: Well, I think that what I proposed in the beginning will give more resilience to the buffer zones and make them more efficient in the face of increased climate variability. Buffer zones should be as wide as possible to provide more surface area, as this will increase resilience and efficiency. If the area is managed well and nutrients are removed from the area, they will be moved outward when there are floods, and the biomass will not be available for the stream to pull into the flood water pulse. This will make it less of a threat to the water pulse.

Interviewer 2: Okay, I have one last question. When you talked about ecosystem management and the idea of allowing cattle to graze in the buffer zones once or twice a year, how do you think that would work with the control mechanisms in place, and what potential problems could arise? If it doesn't work, do you have any other ideas?

Interviewee: Well, that's one of the things I discussed with colleagues when we wrote the report, and they suggested that livestock management should be implemented. In addition, one thing that I suggested was to have staff in the field, similar to park rangers, especially in Paso Severino. These people would work with the producers, go around the area, and verify certain things, making it easier to monitor management in different ways. For example, if a producer plans to put cattle in the buffer zone, they would have to submit an affidavit stating when they plan to do it and under what conditions. With little in-field monitoring and satellite monitoring, we could keep track of what is happening in the buffer zones. It's not enough to simply look at the buffer zones and say, "Something happened here" or "Something happened there." We need to explore other alternatives to ensure that they are feasible. You could have some kind of grazing service, for example, so that the cattle that would graze in the buffer zone would not be owned by the producer but by a kind of lessee who would be the owner of the cattle that were eating in the buffer zone. That is more complex because the buffer zone is a belt around the producer’s land, but it could be another way to get a person specialised in that management. But the control can be like plans and satellite tracking for field inspections, that is, to visit regularly. If they find cows in damaged buffers, responsibility lies with the producer. They will be able to say that the cows made the marks, but if the barbed wire fences are broken and nobody is going to bring the cows back, it's on the producer.

Interviewer 2: And another thing that I was wondering about from the interview is that you told me that they had interviewed producers and that there was resistance that they were showing. Can you share with me the results or anything to do with that?

Interviewee: Yes, I'll pass you the thesis where it was. It's an undergraduate thesis that did a lot of things, so they interviewed three producers, I think, which were the three where we had the experiments. Well, just a clarification: the producers that were hesitant about the buffer zone were the ones with the severe passage. After being in contact with some other producers who were about to implement and were implementing the buffer zones, they were not angry, on the contrary. But there was a whole project with technicians that promoted the implementation of buffer zones. So, the producers were much more consulted on the issue and willing to find solutions together. But this was not the case in Paso Severino because all this work was not done. I'll pass that on to you.

Interviewer 1: That's perfect. I don't have another question. Thank you very much for your time. It's very interesting. And I finished the interview.

Interviewee: Very well done. Well, good luck to you.

*Interviewer 1 is the student, and interviewer 2 is the supervisor.*

# Interview 10

**Interviewee: DINACEA        Date: 14-03-2023**

Interviewer 1: Riparian buffer zones are strips of vegetation along watercourses, and their purpose is to capture and retain nutrients from agricultural activities to improve water quality. This interview will discuss the buffer zones in the Santa Lucia River Basin and will focus on the role of your organisation and your personal perception of the implementation of these buffer zones. Can you tell me about your involvement with buffer zones and the organisation you represent?

Interviewee: Well, thank you. I am very happy to cooperate with the work you are doing. I am the director of environmental quality in the Ministry of Environment, and my department is responsible for monitoring and evaluating the quality of the environment in different matrices, including water, air, and soil. However, our main focus is on water and soils. We also have an important role in monitoring air quality. Besides evaluating, we propose improvements in areas where we find environmental quality problems. For example, in 2013, when there were water quality issues in the Santa Lucia River, our area promoted an action plan to improve the situation. The plan included 10 comprehensive actions, and the buffer zone was one of them. Our goal was to stop the deterioration of water quality in the Santa Lucia River and propose solutions to improve it.

Interviewer 1: And what relations do you have with other parties in this process?

Interviewee: As for our relations with other stakeholders, the Santa Lucia River is a crucial water source for Uruguay, providing drinking water to approximately half of the population. Therefore, we work closely with various interested parties to address issues related to the river. I hope I have answered your questions. Please let me know if you need me to speak more slowly or clarify anything further. In addition to having an important wetland in its final stretch, which is also a river of vital importance, we need to coordinate with different actors involved in the care of the basin and its use. We need to work closely with the Uruguayan water supply agency, the livestock, agriculture, and fishing communities to manage the land and livestock properly. In other words, we cannot do as we please, but we must coordinate with these stakeholders.

Interviewer 2: Which organisations are working together with DINACEA in this process of implementing buffer zones?

Interviewee: Ah, ok, buffer zones; we are talking only about buffer zones. Yes, to answer your question about the organisations involved in implementing buffer zones, we tried to work with the Ministry of Livestock, Agriculture, and Fisheries, but with limited success. We also worked with the intendancies, as they have territorial surveillance. However, these are the primary organisations involved in this process.

Interviewer 1: And how do your activities and responsibilities affect the buffer zone design or management process?

Interviewee: Well, the design and control of buffer zones is not solely in my area of responsibility, as the control of buffer zones heavily relies on satellite images, which fall under the area of environmental information. We work closely together because the fundamental support for the control of buffer zones lies in the information system. They analyse images before and after to observe changes in land use, and that's where we take action. When we have to design a buffer zone, we work more closely with the Ministry of Livestock, municipalities, and producer associations. We work a lot with what used to be RENARE, now the natural resources department, specifically on the design.

Interviewer 2: Which directorate of the Ministry of Agriculture do you work with? Interviewee: It's the natural resources department, formerly known as RENARE. We worked a lot with them on the design. The satellite information part I mentioned earlier falls under the control directorate, which is part of the national directorate of environmental quality and evaluation, where I work.

Interviewer 1: What functions do you think buffer zones currently have in the Santa Lucia river basin?

Interviewee: For us, the primary function of the buffer zone is to retain pollutants, primarily nutrients, from the soil and prevent them from reaching the watercourse. While buffer zones can serve other purposes, such as biological protection, we've primarily focused on pollutant filtering. We've drawn heavily on literature from North American research, which we consider an excellent reference for buffer zone design. They suggest buffer zones can serve multiple purposes, but we've designed ours primarily to filter pollutants.

Interviewer 1: What are the characteristics that support these functions?

Interviewee: In order to retain contaminants, we believe the buffer zone should maintain good soil conditions, specifically a natural soil structure. Ideally, it should be a strip with no intervention, although we recognise that there are other ways of managing the buffer zone, as Alfred mentioned earlier. For example, let's say we harvest the biomass to reduce the nutrients. In short, we are looking at this as a new and improved phase of the buffer zone. Currently, we define the buffer zone as an untouched area without tilling the soil or using agrochemicals. One of the few things we allow is the elimination of exotic species.

Interviewer 2: What is the role of livestock in these areas?

Interviewee: That's a good question that we have discussed many times. As we don't have any form of control, we understand that grazing would be allowed at low stocking rates. Low stocking means minimal grazing, and we don't worry too much about it if the activity is not intensive. Moving on to the functions of the buffer zone in the Santa Lucia River Basin, we think that it should serve as a pollutant filter. It can also have other functions, such as biological corridors, but our design is not primarily intended for that purpose.

Interviewer 2: And if you had to evaluate the importance of these three functions on a scale of 1 to 4: nutrient retention, protection from erosion, and biodiversity protection.

Interviewee: As our design is made to filter nutrients, I rank nutrient retention as the most important, followed by erosion protection and biodiversity protection. However, we believe that protecting fauna and flora requires something different from the buffer zone we designed. For example, it may need vegetation patches and not a strip like a coastal strip. The coastal strip serves the nature of the river, but it doesn't have that filter. Therefore, for this buffer zone proposed in Santa Lucia, we prioritise its function as a filter first, followed by the other two functions. It's worth noting that Santa Lucia has a second-generation plan that includes complementary actions to the buffer zone. This concept is part of the second-generation plan. It was not present in the first plan, which was implemented in 2013. The first plan only involved reactive actions, which were aimed at stopping the arrival of pollutants into the water from soil runoff.

Interviewer 2: Is there a specific time frame for implementing the second-generation plan?

Interviewee: For instance, one of the proposals I came across was to extend the plan to lower-order streams, as stated in the document. However, there has been no implementation of the proposed time frame yet. We are currently working on it and have made some progress. Our plan is to extend the plan to smaller streams, as we have concluded that they are the main source of nutrient runoff. Although we will not extend the plan to all small streams, it is still necessary to take care of the land use. We are also considering extending the plan to lower-order streams, possibly with a wider range of land uses. This might include activities such as grazing or harvesting, but we are still discussing the specifics. We are at your disposal to contribute from our position of investigating these issues. You can count on us as well. As far as our perspective, we have many interesting points, not just a lot of things. Many of these points are difficult because putting a measure in place can be unfeasible in practise. It's not easy, especially given the idiosyncrasies of the Uruguayan countryside. There is a strong culture of land appropriation by producers, as if they can do what they want. Not everywhere, but we do need to work against that culture a little bit.

Interviewer 2: We are also interviewing producers, and some very interesting things come up, especially with exotics and things like that. It will be nice when we bring this to light, but I don't have a preview to share right now. Tomorrow we have a series of interviews, so can you tell me more?

Interviewee: Of course, we are short on time because we don't have a survey of all the opinions. We only receive opinions from isolated people. The producers who don't like the measure are the ones who reach out to me. Sometimes it's someone whose own private life limits them, but not always.

Interviewer 2: We should also interview those who understand that they don't like the measure, so we can understand why. Is it ignorance, uselessness, or something else? But what we have will give a good overview of the different socio-economic strata and understanding.

Interviewer 1: What are the most important differences between the measures for the first and second generation buffer zones?

Interviewee: The main difference is that we are extending the buffer strip to smaller courses of smaller size. It's something like a 5:1 ratio in length. In other words, we want to propose adding about five times more buffer than we did in the first phase. Additionally, in this second phase, we want to have a little more permissibility for the use of buffer zones. For example, the biomass can perhaps be harvested. We may allow some uses that do not go against nature in any way. When we enter very small courses, we are entering the individual producer's standard. The large courses we put in the first phase are already recognised as protected courses because they already have a riparian forest and people are used to going to a beach or forest. So it's easier to work with them. When you approach small watercourses, you are essentially interfering with a producer's private property. They don't see it as just a small course, but rather as their property. For instance, the Santa Lucia River is recognised as communal property, but a small stream on my land belongs to me.

Interviewer 1: Now, moving on to the implementation of these changes and additions, what benefits do you think they would bring? Specifically, what benefits would these changes in characteristics offer?

Interviewee: Well, the main benefit is that it would reduce the amount of nutrients that enter the water from the soil. This is crucial for the environment and water quality, especially in the Montevideo metropolitan area, where 1.5 million people rely on drinking water purification. By extending the buffer to smaller watercourses, we estimate that we could retain up to 40% of the nutrients. However, it's important to note that buffering alone won't solve the problem. We need to implement clear land use measures and practises to address the source of the problem. Buffers are a temporary solution, as they tend to become clogged over time and lose their effectiveness as filters. Therefore, we must address the root cause, which is land use.

Interviewer 1: And what obstacles do you think will be experienced with the implementation of these changes to buffer zones?

Interviewee: What obstacles?

Interviewer 1: Yes.

Interviewee: It is not going to be easy to admit interference with a public good. There is a problem. It is clearly stated in the constitution of Uruguay that water is public, but the little course of water that passes private land is not protected as such. So, we have a problem with competition. The land is private property; the water is public; but the coastal border is public because the border is also part of the water. So, how does this work? I think that in any case, the obstacle is going to be there, but I think that we are not talking about compromising important areas of land. They are very minor strips, and we are talking about 15 to 20 metres of protection on each side of the minor courses. They are practically strips that should not be used because it is also a somewhat floodable area at times. So, in short, what I think we are proposing is to do what should be done naturally. I don't think it's natural to plant and farm right up to the water's edge. Any sensible person realises that this is not right. There has to be a buffer zone because one also applies agrochemicals, so there has to be a distance where there is protection for the natural resource that is water.

Interviewer 1: What possible solutions do you think can be developed for these obstacles?

Interviewee: I think there is a question of whether it should also benefit the producer. We need to provide a subsidy or, in some way, a lower rate of taxation on land, at least in the area that has been set aside as a farming zone. It seems to me that it is even fair because you are changing the rules of the game a little bit for a producer. Until recently, nobody said anything to you, although you had to protect the course with a margin. Now we come to say that you have to do it, and then I think that the state should at least give you an exemption for the payment of the tax that you have to pay for the land. I think that would be the way to go. I don't see much else after that, but it seems to me that it would be fair. What happens is that the Ministry of Economy tells you that it’s not possible. We've had all kinds of things, but in general, they are not very open to it. Although now there is a certain openness because the Ministry of Economy has brought up the issue of carbon financing, Maybe there can be a little more vision. Guillermo, you are a member of the economists, but in general, it would be good to have people who are more open-minded and who understand that all of this is a discussion that they know better than me. Ultimately, one should pay for the environmental service that the site provides to those who, in some way, own it and carry the burden of it. But it is a concept that economists find quite difficult to understand. In other words, on the one hand, you lose money, but on the other, you gain. However, sometimes you need to create an environmental account. Environmental accounts are not popular, but in Uruguay, I have seen a few people do it, like Caffera (a colleague, red). I know Caffera well; he was the first to work on this subject. When I am ready, I will inform you of a possible solution that is being discussed, because where does the money come from to make it fairer? If you pay someone, where do you take the money from? I have a proposal for water: put a tax on fertilisers. I am exploring the theoretical possibility of putting a tax on fertilisers that attacks the source, the cause of the pollution, and somehow transfers it. Then the downstream land is purely under public management. At least we can do the calculations to see if it is enough or not, if it can be explored or not. However, there are issues about whether it is taxed as general income. It cannot be. It is a legal mess. There is also the issue of water collection. It has never been implemented by Uruguay. It has always been discussed—I don't know how many times. Even in the previous mandate, they were on the verge, but it didn't come out either. You could implement it, as you could perfectly well turn it towards the protection of the environment. That is to say, the subsidy of this type of thing But obviously, you have to manage it better. We even managed to charge for water in the first-generation plan in 2013. We discussed the possibility of charging for water in the Santa Lucia River, at least. We even charged OSE for the water. In other words, it may be a public company, but it uses a public asset. It could pay, for example, one peso per cubic metre or one cent per cubic metre. That gives around two million dollars a year, and you can subsidise all the protection of the watercourse itself. It's an idea that closes on all sides. Of course, the consumer pays, but the amount is minimal, right? There are issues of justice to consider as well. The consumer pays for a lot of things, including all the inputs and the OSE tariff, which includes the cost of the water, employees, treatment, and inputs. As a result of its use, it is a circuit. I charge the user a little for the water, but I'm going to reduce it because with this system I'm going to have to use fewer products. However, it's not an immediate solution. It's not like that tax that the government announced in the last few months—the low network of its tax reduction bill. You won't see the improvement process tomorrow or even after three months. We are talking about processes and raising the cost of water by one hundredth, and the truth is that I will see the improvement process within five years from then onwards. Well, I got off topic, didn't I?

Interviewer 2: No, no, no. I think it's interesting to hear your opinion as well.

Interviewee: I'm sure we've discussed it at all committee meetings. In all the committee meetings and all the governments, nobody quits. I think it is a mistake—and I say this sincerely—that no government, no matter what kind of government, assumes that water, for example, should be charged for. It should be charged for, even for ethical reasons. Because you can use a public good as precious as water for free and still, in some cases, sell it like premium water, bottling it and selling it, right? I mean, it's not even water for irrigation, for the plants, or for later selling a product. No, no, it's water that has already been bottled and sold.

Interviewer 2: Yes, it is a complex issue. Sorry, Alfred, but we went a bit off topic.

Interviewer 1: No, it's very interesting.

Interviewee: Yes, all this is so interesting that one has already left with a lot of desire. But every idea and pressure you have will be welcomed, because sometimes pressure from groups of researchers is important. Sometimes, we within the administration can't do much lobbying because we are within the administration, right? Anything else?

Interviewer 1: And how do you experience climate change in the Santa Lucia River Basin? Think about the changes in temperature, rainfall, droughts, and flooding that have been noted over the years.

Interviewee: With this heat that we have and it being dry, it couldn't be a better question, could it? In other words, if anyone doesn't believe in climate change, let them see what is happening today, right? With this dry reservoir. I have never in my life seen a dry reservoir like Canelón Grande. So, if someone is not convinced whether to believe in climate change or not, I think that there is clearly an issue of extreme weather that no one can argue with. I think that we are ill-prepared; everyone is ill-prepared, but Uruguay is very confident in its water resources, thinking that it has surplus water resources. I think that this is a mistake. In fact, the drought is proving it; it is dry. There is no water anywhere. There is water in the La Plata River because, fortunately, we are in the basin of the entire Paraná Paraguay, which is an enormous basin, and there are also floods at the moment in the north. But on the other hand, if these things don't convince us that we have to protect the water source, then what will? When I say protect, I mean more than just an area that is an asset. When we say protect, we mean to protect the quality and quantity. When you protect a watercourse, you are talking about the biology, the quality, and also the quantity. For example, there are uses of water that are perhaps excessive today. Maybe you are making excessive use of the source for drinking water that can't hold any more. And then comes the whole other way of life from another source that doesn't give me enough to put in. Climate change permeates and distorts all of this. Clearly, we are immersed in that, and clearly, the actions we take will be crucial. Now, the floods didn't come, but in between, we are floating everything with the amount of water and floods. So it has a different connotation, doesn't it? We no longer have a problem of quantity, but we can enter into a problem of quality. We can enter into an issue of deterioration of land use, sediment runoff, and nutrient runoff, in short.

Interviewer 1: And do you think that climate change could affect the functioning of buffer zones in the future?

Interviewee: I really don't know. Basically, we deal with floods; droughts don’t affect the buffer zone. However, when there is flooding, we have had many discussions about the fact that some propose the buffer zone should be a flood plain, meaning it should be a strip or a figure that is a plain where it floods with a return period of 10 years, for example. This should be the buffer. The problem is that this is complex to implement and has much higher costs. So, when we propose a small buffer, like the one we are proposing, of 30 or 40 metres, we assume it will flood every two or three years. This may significantly dilute the possible nutrients retained. We haven't measured this, and we haven't seen it in the literature. Also, many of the nutrients are immobilised in the sediment, so not everything is taken away. However, flooding can be counterproductive to the idea of a retention buffer. Flooding is always there; it depends on the return period. If the return period is 1,000 years, two kilometres on each side of the watercourse must be taken. If it's 100 years, 500 metres are taken. If it's 20 years, 200 metres are taken, and if it's 3 years, 40 metres are taken. Somewhere, that sets the limit and the risk, so we value that a little bit. Do you have any other questions?

Interviewer 2: I have one last question. On this issue of the drawbacks of the implementation of the second generation, you mentioned the issue of the producers who are going to be against the implementation. Do you see any other drawbacks on the control or management side? Any constraints in that sense? Because you are expanding and multiplying the buffer area.

Interviewee: Effectively, everything becomes a line with a border that you have to look at with images. But, of course, there is also this elegant algorithm that works half alone, and that is what the ministry is working on. Sometimes I'm not so convinced about why more is needed. If it's ten times more, it's ten times more work. But, well, it has to be assumed. The state has to assume it has a cost, and it will require a higher cost in terms of people or instruments to protect the environment and water. But that's another job. I'm not going to go into that because I would have to go into the United Nations next to the issue. I mean, how much can governments, or I don't know if governments are the same as states, influence state policies? How many are willing to protect the environment despite the higher public spending costs? I really don't know. I don't think anybody knows, and nobody can answer that, especially when it comes to things that have invisible benefits, right? Benefits that cannot be monetized or that are very long-term escape the attention of one or two governments. So, let's say I make a great plan for the Santa Lucia River, but the benefits of that will only be seen in 20 years. You may think that's funny, but it's true. We are talking about decades here. When we published the first action plan for Saint Lucia, I was one of the people in charge of making it clear that the results would only be seen in a decade or two. That was demarcating and devastating, especially for decision-makers and politicians. They are doing something, but they are being told that the benefits may only be seen 10, 15, or 20 years down the line. It's true that things may improve after that time, but maybe not, because if we only focus on that one thing and neglect other important factors, the deterioration will continue. If we don't control the fertiliser in the soil, for example, no matter how much we put into an easy zone, we won't be able to control the nutrients in the soil, and things will only get worse. Political timing is another major constraint when it comes to environmental measures. The environment has the great disadvantage of being subject to political timing that doesn't always fit in with other things. I may be making a monetary policy, but it's very difficult to see the results of environmental policies within political timeframes. When it comes to management, I may launch a programme to reduce plastics, and people can see it. However, the reduction of plastics in the ocean may only be visible once every 50 years. I can't base a policy solely on reducing plastics in the ocean because politicians may not be interested in that. We need to be nuanced and balance visible actions with necessary actions that may not be immediately visible but are important for future generations. This issue is a clear case of intergenerational concern. What we do may not be immediately visible, but it's important for the future. We are about to make a presentation on the Saint Lucia River Basin, and we are seeing improvements in the quality of the environment. However, we have reached a plateau, and that's good news. It's not a small thing. If someone told you that the only thing we achieved in 15 years of process deterioration was to slow it down in 10 years, that's still a significant accomplishment. For me, it's a success. But it's relative, and it needs to be sold as such.

Interviewer 2: Very well, I have no more questions, Alfred.

Interviewer 1: Me neither.

Interviewee: When you have something halfway worked out, give me the information to see what conclusions you've reached and how you plan to solve this mess. Good luck.

Interviewer 1: Goodbye. Thank you very much.

*Interviewer 1 is the student, and interviewer 2 is the supervisor.*

# Interview 11

**Interviewee: Producer        Date: 14-03-2023**

Interviewer 1: This interview is about the implementation of riparian buffer zones in the Santa Lucia River basin. What do you do for a living, and how are you involved with riparian buffers?

Interviewee: I am an agronomical engineer, and I work as an advisor for producers in the Santa Lucia basin area, some of them in the buffer zone as well. I also work at the Faculty of Agronomy, where I am a teaching researcher. Additionally, I am a producer on a farm situated in the Santa Luca river basin area of Canelón Chico. The Canelón Chico stream is one of the tributaries of the Santa Luca, which belongs to the basin. My contact with farms and this issue comes from being one of the two delegates for the Comisión Nacional de Fomento Rural (CNFR). It is an organisation of producers from Segundo Obrado, the most important one in Uruguay, that brings together small and medium-sized family producers by definition. I was one of the two members who participated in the Santa Luca River Basin Commission in 2013. In 2014, a mixed commission was created between civil organisations and social movements, groups of producers, neighbours, and people who fought for the issue of natural resources and were very concerned about water quality, the well-being of the area, and biodiversity. Many groups of people and citizen producers, as well as other associations like the National Association of Milk Producers, which has many dairy farms and other farms in the area, participated. I represented the National Commission for Rural Development as a delegate, representing the interests of a civil organisation and a private organisation. Public bodies, ministries, projects, different agencies, and the mayors' offices of Canelones and San José were also involved. You must have already surveyed them in the survey. There are primary or local development societies in the area that you can interview. I can give you the names of referents and some producers that are well-versed in buffer zones and have been affected by them. Maybe they can give you a more experiential account.

Interviewer 2: Your vision is also very good from the point of view of the representation of producers who are located there. I think that you also have to deal with producers to have a vision of the problems and the things that are generated.

Interviewee: Yes, even the action plan that was to be drawn up and that was consulted with civil and private organisations was based on defining different zones—zone A and zone B—where stricter measures were to be taken for the care of natural resources and water in the buffer zones, as well as the whole Santa Lucia basin drawing up a management plan with regulations on what could be done. This plan includes many rural development societies and producers, and since I live in the area, I am also part of the basin. When you asked if I was in zone A or B, I confirmed that I was in the smaller zone. At the back of my farm, there is a gully that flows into the Canelón Chico stream, which in turn flows into the Santa Lucia river. So, the runoff water from my fields ends up in the Santa Lucia River, just like that of many of my neighbours and other producers. If you want, I can give you more details about producers and union leaders of organisations that are very involved in wetlands in the area we are interested in. However, I think my greatest experience was participating in the Basin Commission. Regarding buffer zone management, I can provide information both as a producer and as a representative. However, I cannot give you a definite answer on how I would be affected if the environmental authority came and declared my farm part of the buffer zone. It would depend on the specific regulations and how they are enforced. Well, here we were perhaps called to participate in a commission, and the character of our participation was consultative. We did not have the right to make normative changes. The commission invited social organisations for consultation but did not give them the power to decide on subsequent regulation, application, and drafting. We were there in a consultative capacity, which is the first definition of the commission's purpose. I don't know if Guillermo, who has studied these issues a bit, agrees, but it was a commission in which organisations were invited, but we didn't have a vote, and our opinions were not brought together.

Interviewer 1: These buffer zones can be designed in many different ways, for example, using different land cover types and management strategies, so there are different ways to characterise these buffer zones. The next question is about how you would describe buffer zones. What purpose do you think buffer zones in the Santa Lucia River basin currently serve for you?

Interviewee: With the implementation of the action plan, measures were taken to prevent the degradation of water quality and natural resources in the basin. An action plan was created. What happened is that, it seems to me, I lost track of this because, after 2014, I stopped participating. I think that in the case of the protected area of Santa Lucia, they didn't manage to approve a management plan that other protected areas in the country have. Because if one enters the Ministry of Environment's website, which has now changed a bit, I never see that the management plan has been approved and that it has the signature of the authorities, unlike, for example, the management plan for the Laguna Rocha basin or other protected areas. So it seems to me that we worked on it, we participated in an action plan, agreements were made, but then the executive and the government institutions had to move forward and design the management plan, which obviously the government approved by decree, but now I was checking, and I did not find the management plan. I don't know if a management plan was approved at the national government level in the case of Santa Lucia. I am saying this because, from everything I am going to talk about later, it seems to me that what started off with a lot of strength later became deflated, and that is my final summary. It started off with a lot of force, but then they couldn't keep up with the pace of the measures they wanted to implement, let alone control. In other words, today there is probably no supervision or control. Nobody controls any of the measures that were promoted in that action plan. The truth is that I don't know either because, as we also concentrate on zone A, I think that the wetlands of the protected area are in zone B, which has another purpose. It was separated and has more to do with water quality but also with biodiversity and ecosystems for birds that come to look, etcetera. We are not looking at it very closely, although it is important. We are looking at the interaction of the riparian forest, basically in that area, which is not part of the protected area for now. But I don't know; I honestly haven't delved into whether there is a management plan. It seems to us that the work has not been completed and approved because when one enters the presidential web page of gub.uy, enters DINAGUA or DINAMA, searches for riparian buffer zones or the protected area, and gets to the Santa Luca river, it says credit proposal presented, but it doesn't say management plan approved, while in others, it does. But well, that is my impression: that the implementation of a plan was slowed down and not really implemented and carried forward.

Interviewer 2: You mentioned that the buffer zones were called upon to reduce pollution or improve water quality.

Interviewee: It seems that they were called upon implicitly for a measure that protects water quality, such as the buffer zones, which are at the boundary of the watercourses. We, as actors in situ, are the producers who are interacting and whose actions end up having an impact on the runoff of our fields and the land that goes to its tributaries and ends up in the Santa Lucia River. So, when we identified the possible actors or activities that could have an impact, we were among those possible actors. In other words, we obviously had a negative impact. These include agro-industries, locals, cities, and the OSE itself. The OSE was also one of the first actors to do wrong with the issue of sludge and everything it dumped. But then we were invited to participate to say, "Well, what can we do to make produce in a way that has the least impact on the quality of runoff water that is going to go to the Santa Lucia?" The government says, "Do you think that we can implement a strip of the main streams and rivers of the basin of so many metres to do agriculture, horticulture, and livestock?" Well, that was our work as a committee. Well, at what level of phosphorus or nitrogen, according to a soil analysis, is it forbidden to make new additions of fertiliser? 32 ppm of phosphorus, 15, 10. Give your opinion. There was academia, there was research, and everyone had an opinion. We, the producers, raised and defended our rights and our vision of each of the measures in the action plan that was being drawn up. For example, the installation of cattle feedlots was prohibited. Some cities were also forced to improve their sewage systems. A lot of measures, right?

Interviewer 1: And going back to the buffer zones, do you think they have any other purpose apart from the watercourse margin zones, such as retaining nutrients, fertilisers, and runoff from agriculture? Do they serve any other functions?

Interviewee: The latent idea is that producers can differentiate their production. In other words, it could not be a designation of standard agriculture, but it could favour organic or agroecological production. This type of production would find the ideal place to develop if it were going to be promoted a lot. Some projects have already been implemented, such as the Development Society in Melgarejo (Sociedad Fomento Rural de Melgarejo, red), which had a project, I don't know if it was with the United Nations or not, to organically produce some agricultural products and see if it was possible to apply fewer inputs on traditional farms. However, my impression is that no regulation or legislation has been disseminated regarding the buffer zones. I have never seen a formal presentation of the management plan for the Santa Lucia River. Secondly, there is no capacity or proposal to develop each of the projects contained in what could be a management plan to achieve the final objective of not having a negative impact on the quality of the water. I did not see the Ministry of the Environment or the Ministry of Agriculture promote projects to help producers implement ways of producing and reconverting the way in which each of the chakras is being worked. In other words, there is a lack of proactive attitude to generate changes in development projects. I don't know if there were any oversight measures. Apart from specific programmes like those run by the UN and FAO, which are very specific and small in scale, I haven't seen any oversight. The state bodies themselves have contradictions between them. Another technician and I presented a project at the National Institute of Colonisation for a development society facing the Santa Lucia River, which has a coastline of about 400 metres and very fertile land. We proposed beekeeping in native forests, livestock farming in reconversion as far as possible, and organic and seed production. However, the National Institute of Colonisation prioritised another project proposing agriculture and calf rearing without considering the action plan for the Santa Luca River. It was approved by the National Institute of Colonisation as something disconnected. The public structure itself has not incorporated the plan into its lines of action or its priorities. It was surprising to see that, in my opinion, in the case of the Santa Lucia River, it was discontinued. Even the SNAP (Sistema Nacional de Areas Protegidas, red.), for which a consultant was hired, terminated it after the term ended without a good relationship. Then, the authorities, governments, and priorities changed, and other administrations perhaps did not prioritise it. Within the Ministry of the Environment and DINAGUA, this issue seems to have been diluted without any resources or human resources being allocated to it. Everyone talks about it, but in reality, there is no monitoring or control. It even seems that there are no projects to promote the sustainable use of the land in this system. There is no promotion to help properties convert to more sustainable agronomic management measures. We need a management plan for Santa Lucia, but I don't know what you have explored, seen, or heard. For now, we are focusing on promoting and studying the issue of limiting land use to a margin of 40, 50, or 100 metres, so that neither ploughing nor fertiliser application can take place and it functions as a natural barrier.

Interviewer 2: In any case, this vision you are sharing with us about the lack of control and attention that has also appeared in other actors is good, isn't it? I would like to ask you what you think should be managed or changed in these measures. For example, what things would you change or how would you implement a measure like this: a vegetation zone where neither agriculture nor livestock farming can take place, covering an area of around 100 metres? I'm unsure how, but considering the aspects you mentioned, how would you ideally implement this?

Interviewee: We agree with the action plan that was drawn up, as it was the result of a negotiation process. For instance, we saw our producers making a pact there. If, for example, I have a farm that is 200 metres by 300 metres and a gully runs along one edge, a 100-metre measurement would mean that I could not use one-third of my farm. This has a significant impact on a productive unit that has 4 or 5 hectares to work with. Our participation aimed to establish measures that would not affect the structure or productive matrix of the farms. It's one thing to have a property in front of the first line of action, which has a coastline against the Santa Lucia River. It's another thing to have a property with a coastline against a main stream, and there are also many properties on secondary tributaries or streams that are further away, that are part of the basin, and in which there are many smallholdings and small properties. When it came to regulating, our concern was that putting very large distances between small plots could render this productive structure unviable. There are even smaller plots, such as 3 or 4 hectares. If you establish a 100-metre measurement, it could mean that half of the plot cannot be used. I hope you understand. The last part was a bit difficult to explain. A 15-metre line was established on the banks of the streams, in which the producer had to put a wired fence or a movable wired fence, limiting the access of the cattle directly. That's actually theoretically fine, but practically, it has some implications. For example, land between 15 and 20 metres in front of a small watercourse far from the Santa Luca, a secondary or tertiary tributary. If the producer doesn't put livestock on it, who looks after it? Who maintains it? Because it turns into a forest, it becomes semi-woodland. The water source doesn't flow because plant debris starts to collect and the streams become clogged. Who takes care of this maintenance? It's easy to say, but then there has to be maintenance of the watercourse. Because if the producer cannot exploit it, then who cuts the grass there and who maintains the tapestry? Because then it becomes bushy and attracts pests like foxes and hares. Many animals interact with the cultivated land. So, with small and intensive farms, it is difficult to see who manages that area. If the producer cannot access or manage it, then who can? This has always been a latent issue for me. For example, it happened to me on my farm. I had some animals, and we put up a fence, and over the years, we could not enter the stream. It is now a jungle (wild, unmanaged forest, red). When it rains a lot, it gets blocked and invades the land. If the water channel gets blocked and invades the crop fields, it goes out of the channel, and the solution is worse because it invades crops and land. If it would go along its natural course and the grass was short, there would not be any upward dragging of nutrients or sediments. That is why some measures, sometimes when there are public policies, are easy to regulate, but then you have to see how to manage the practicality. Because there are many young people who do not live directly in the countryside, who are not in contact with the ecosystems, and who do not have much idea of how this evolves.

Interviewer 1: I have a question: what characteristics could be added or modified to the buffer zones to make them viable for small farmers, for example? You mentioned earlier how it could be improved to fulfil a specific function, such as nutrient retention. How do you think this could be achieved?

Interviewee: It seems to me that direct access to water for animals through watercourses would have to be prohibited, but grazing would have to be allowed. Otherwise, the strip of land would not be managed, which is a problem. Who manages that strip of land? Nobody. I think the argument that there may be grazing in that strip is insignificant compared to the contributions of agriculture or a production area. Obviously, there is no feedlot, concentration of livestock, chicken farm, agricultural cultivation, or fertilisation, but grazing should be allowed on a low carpet with some trees that does not interfere with the runoff. There should be no dirt or residue from trees or branches, no obstruction of natural water supply routes or watercourses, and a small undisturbed tapestry of a few metres that in some way filters the runoff of the soil that carries nutrients. This would be the right thing to do.

Interviewer 2: Do you see it as viable to do intermittent grazing, for example?

Interviewee: Yes, I could make a solution with controlled, intermittent grazing, a certain number of animals, set thresholds, and a certain number of animals per path, certain times yes, other times no, perhaps in flood zones. Not in winter periods; in summer, yes.

Interviewer 1: What do you think the benefits of these changes to the buffer zones would be?

Interviewee: Nowadays, the regulations are as they are; nobody controls them, and in some situations, they do not contribute to the objective and can have the opposite effect. For instance, if I'm not allowed access to my strip of land in the last 15 or 20 metres against the stream, in 4 or 5 years it will become a jungle of trees and everything. When the water comes, it will strip it, flooding my land and reaching farming areas it didn't reach before, taking all the phosphorus and soil, which could circulate freely due to its natural cause if it were kept with a low cover.

Interviewer 1: What obstacles or disadvantages do you see with the implementation of these changes that you mention? Thinking about the changes we are discussing, such as allowing or reducing the stocking rate or making grazing more intermittent in certain months, what disadvantages do you see in general? Firstly, as a producer, and then in general?

Interviewee: I only see benefits.

Interviewer 2: Are there any disadvantages that can be implemented from a political point of view? This is more general. As a producer, you only see benefits, so for the producers, you only see benefits, right?

Interviewee: On a technical level, I don't see anything negative. You have to differentiate between the various protected zones or areas at the country level. For example, in other protected areas or other basins where there is very little human intervention and they are very natural basins, where the soil is not degraded, where there is no tillage, where there is not such intensive production, and where there are not such small properties, the measures can be different. That's why, for me, sometimes making a rule for everything the same doesn't work. We have to be aware that the Santa Lucia river basin is dominated by smallholdings and highly intensive production, such as horticulture, fruit farming, dairy farming, and agriculture, in which the input of nutrients is high. The volume of production that comes out of this basin, be it grain, milk, meat, fruit, or horticulture, is very high. It is different from the Rocha basin, for example, or the protected area in the area of the Quebrada de Los Cuervos in the Lunarejo valley, where the number of inhabitants per square metre and the productive systems are low, and even the systems are very extensive in the addition of nutrients in the ploughing of the soil. The Santa Lucia basin and wetlands are, I think, the most intensive basin in the country. If you look at the gross production, the amount of added value that comes out of that basin is enormous. The problem that we have in Santa Lucia is the same problem that the rich areas of the world have. Any highly productive watershed in the world, even in Europe, has associated pollution and nutrient recycling problems. So, in the case of Santa Lucia, the management plan should take these circumstances into account. We have to be aware that we are in a highly food-producing area. On a different societal level, it's difficult to balance because I always ask myself, "Why do we producers have to be responsible? Many times we are identified as polluting watercourses when people throw rubbish out of the window or in a dump truck, throwing whatever they want. Producers often produce food for a thousand families. So, of course, the system produces some surplus, but we have to balance what it also contributes.

Interviewer 2: So the issue is who is ultimately responsible for the pollution: the producer or the consumer?

Interviewee: We often see that it is the producer, but when you buy a bag in a supermarket with a lot of packaging or when you have leftover food and you throw it away, you are polluting. Of course, it goes to the sea; it's further away, and we don't notice it. In a productive area, we do notice it. I think that at the societal level, we really have to consider that, in reality, it is everyone's responsibility. If we are going to question the production model and the responsibility of society as a whole, not just the producers, then we should also question the consumption model in cities. We should ask ourselves how society views producers, for example. No citizen thinks that they are a potential polluter when they flush the toilet or put waste in the bin, but if a producer drops in three more pebbles of fertiliser, they are polluting. If someone orders twice as much food as they need and puts half of it in the bin, nobody questions it, but that's phosphorus and nitrogen going somewhere. It's going to destroy some water source or the sea, yet nobody questions it. When someone turns on an air conditioner all day long or public bodies leave all the lights on, nobody questions it. But on the other hand, if a farmer is driving a tractor and burning fuel, people think it's horrible. But we have to understand that if the consumer likes to consume such things, they also have to realise that in order to produce them, there is an energy cost and waste left over. And that's why, although I think awareness is important, I don't mention it often. When it comes to dealing with the problem, many of the measures to mitigate the impacts of water quality have costs. So ultimately, who bears that cost at the state and social level? Does the producer have to bear it by moving 100 metres away from the stream? Why? Society as a whole consumes what that producer produces. It's like saying we will remove 50 square metres from your flat so that you can grow trees or an organic vegetable garden, so you don't pollute as much. I don't think we've matured as a society in Uruguay in terms of questioning whether watersheds and production systems are polluting. This message hasn't reached the city level yet. However, more developed countries in Europe have had these watershed problems for 200 years. All the rivers in Europe are polluted, as it's difficult not to pollute due to human activity. Nevertheless, establishing a balance between producing sustainably and investigating the impacts on water sources or basins is a challenge. At the governmental level, administrations are avoiding the issue as it generates many conflicts of interest and harms many actors. But, well, fortunately in Uruguay, it seems to me that over the last two or three years, the issues of agroecology and organic production are beginning to gain more awareness among producers, consumers, and decision-makers. They are starting to take on some elements that help to design public policies for the management of productive and natural resources, with a focus on taking care of natural resources. However, we are still very new to this in Uruguay, and in the region, I would say that Uruguay is a leader in managing its resources. I just came from Brazil and was shocked by how they are polluting all the tourist beaches, rivers, and streams. There is no urban planning at all; it is a disaster. The truth is that if I was disappointed in Uruguay, I was even more so in Brazil. The rivers in the city, the streams, and the beaches have no sanitation, and every landowner does what they want. The oil companies, the ports—it's all a disaster. I went to Recife in Pernambuco and saw no urban planning whatsoever. It was a disaster. When I came back to Uruguay, I felt happier because, despite all the negative things I just mentioned, which I do to promote discussion and try to generate changes, we are still better off than many other societies. However, we are still very backward compared to Europe, which has been facing these problems for 150–200 years. For example, any of their rivers that have been around for that long are already polluted. We are discussing the management plan for the Santa Lucia River, and while I understand that the OSE intake needs to have quality water, it was built by the English in 1900, when Montevideo had only 300,000 inhabitants. Nobody thought about building a water intake in an area higher up in the Minas Sierras, where the population has not grown as much and where the country's intensive productive activity has not grown as much either. OSE also dumps sludge and other chemicals and waste from recycling water into the Santa Lucia River and has been fined by DINAMA on many occasions. Therefore, I think that only focusing on the responsibility of producers is only seeing a small part of the discourse. Most of the cities that you mentioned, such as Santa Lucia and Canelones, do not have any overtones, and they dump directly into the Santa Lucia River, even though there are some sewage treatment plants and sewage networks and many industries are under control. However, I don't want to lose Alfred, and I want him to continue with his questionnaire.

Interviewer 2: Alfred, I have one last question. What possible solutions do you think can be developed for these obstacles?

Interviewee: Well, for different obstacles, there should be different solutions. Some involve the producers, and others do not. At the level of institutions, such as what we were just discussing, control of industries, that has nothing to do with the producers. It is a matter of public policy and control. Urban planning depends on the local government does not depend on the producers. One problem, for example, was that the river basin committees stopped functioning, and civil organisations were no longer invited to participate. Another issue was that I don't think they ended up approving a management plan. In those projects, I imagine there were plans for reconversion, information, and training for producers in the area on how to manage their productive systems in the buffer zones, and support for productive alternatives. On the one hand, there are plans to fulfil the objectives of the management plan, and then there is no progress made in terms of control. For me, an aspect that is very important is the governance of the plan. Who is responsible for all of this that we are talking about? Ultimately, it is DINAGUA and the Ministry of the Environment. Who is responsible for livestock farming? What is the executing unit in charge of this nowadays? For example, one of the measures in the plan was that producers could not fertilise if the soil analysis of the plots within the zone showed more than 32 ppm of phosphorus. However, nobody has ever checked that in their lives. It is wrong because producers do not even hire a technician. They just apply whatever they want. There has never been any monitoring, training, or awareness-raising on this issue. No workshops or alternative measures have been put in place. That is to say, in the commission or development society in my area, no one has ever received training or information from the government. There are three rural development societies: one in Lunarejo on Route 49, another in Bella Vista, and another in Velázquez. There are producers and directors who are there, and as far as I understand it, this issue was very popular at a certain moment, but then it didn't move forward. Obviously, if you take a tractor against the coast of the Santa Lucia river and work all over, and someone takes a photo of you and publishes it, the municipality comes, sees the dynamics, and fines you. But that's not how things work because you have to be caught or reported by someone.

Interviewer 2: Well, all the things you have told us about are so good. I'm going to have to leave because I have another meeting at eight o'clock, and I'm already a bit late. But Alfred, I don't know. Where do you want to go?

Interviewer 1: I don't have any other questions either, just to thank Sebastián for all the ideas and things you have told us, which are very important. You mentioned quite clearly the disarticulation between organisations and communication.

Interviewee: I think you will have to interview representatives of DINAGUA, the Ministry of the Environment, and the environmental management of the municipality. The director of environmental management of Canelónes participated in the development of Canelónes (Leonardo Ur, red.). If you ask him for an interview in time and he agrees, I think he used to participate in the Basin Commission. Today, he is part of the executive of the Canelones Municipality since the Basin Committees began to function. I remember that Leonardo participated. It would be good to have some information about how producers in the area feel. They are right there in the area, so I can pass on some contact information to you.

Interviewer 2: It would be good if you could share the contact information of a producer, especially in the higher part, who is affected by some kind of buffer zone. It would be helpful to know if they know anything informal, whether someone has arrived or not, or anything at all, because everything is useful. So if you could share that with us, maybe later I can ask you for a contact on WhatsApp. That's impeccable. Thank you very much. Have a good end of the day.

Interviewee: Well, thank you very much. Good luck. Interviewer 1: Thank you very much! We'll let you know when you get the result.

Interviewee: Cheers, Alfred, Guillermo.

Interviewer 2: Well, I'll see you. Thank you very much.

*Interviewer 1 is the student, and interviewer 2 is the supervisor.*

# Interview 12

**Interviewee: DINACEA            Date: 15-03-2023**

Interviewer 1: The Santa Lucia River Action Plan introduced the use of buffers in the Santa Lucia River basin. What are your organisation's responsibilities and objectives in the design and implementation of the plan, specifically in the design and management of the buffer zones?

Interviewee 1: Well, the buffer zones were established in 2015, and the main objective was to prevent nutrients from entering the city's waterways as they started to see frequent explosive algae growth events. Anything else?

Interviewee 2:  And what are your responsibilities with respect to this plan, and who do you represent?

Interviewee 1: We represent the Ministry of Environment, and we work in the area of environmental control and performance. Our main role is to ensure compliance with the regulations set forth by the ministry. The regulation prohibits any modifications to be made in the buffer zones, including agrochemical application, modification of vegetation cover, and soil loosening. Our responsibility is to ensure compliance with these regulations. We use satellite control to check whether there is compliance, and if necessary, we visit the field to verify. This has been our main task since the first generation of measures in 2015, which covered the entire Santa Lucia River basin and comprises approximately 2,900 farms.

Interviewer 1: Great, that's more or less what I was looking for. What kind of relationship does your organisation have with other stakeholders involved in this process, such as producers or other ministries or organisations?

Interviewee 1: As the responsible ones, we work closely with other stakeholders, such as producers and other ministries, to ensure compliance with the regulations. We communicate and collaborate with them to achieve our shared objectives of protecting the Santa Lucia River basin. The Ministry of the Environment is responsible for controlling the buffer zones. This includes this restriction in land use plans, but they do not control it; they simply inform the producers. Control falls under our supervision and responsibility.

Interviewer 2: I have another question, but I'll ask it after.

Interviewee 2: We don't have a direct link with other organisations, but we do have exchanges with them to receive comments, complaints, or warnings. However, control remains the responsibility of the Ministry of the Environment. Regarding the land use and management plan, the producers declare what they plan to do with the areas under their management. They are not allowed to make modifications to the buffer zone or apply agrochemicals or tillage in these areas. The land use plans include this regulation (buffer zones, red). The Ministry of Livestock controls the soil use management plan, which aims to prevent erosion of the entire productive unit from exceeding a threshold. However, compliance with this plan falls under the Ministry of Environment's jurisdiction. They receive the plans, and if the producer is not complying with them, they call attention to it. The Ministry of Environment controls and handles the legal part of the process, and they require the producer to comply with the regulations.

Interviewer 1: The function of buffer zones is to retain nutrients. What other functions do they have?

Interviewee 1: The width of the buffer zone in Uruguay varies according to the sub-basin's load and what is expected to arrive (in terms of sediments and nutrients, red). The width of the buffer zone is adjusted to fulfil its objective. The natural (e.g., native, red) vegetation is the most suitable for this purpose, and there is no management of the buffer zone to improve the uptake of nutrients or solids. At this stage, the natural or artificial vegetation is not modified. Ideally, there should be no livestock in the buffer zones, but in practise, there is often livestock grazing in these areas. In the second-generation plan, it is being considered how to manage grazing in the area. It is not yet clear how it will be managed, but grazing will occur at low pressure in the area, not in the watercourse, to take advantage of it.

Interviewer 1: Do they currently have other functions as well?

Interviewee 1: The area was designed with other functions, and indirectly, there is conservation of biodiversity in that area by not modifying it, but that is not the main objective. The main objective was always to improve the quality of the water, which was quite complicated at the time or looked like it was going to get worse. Today, the issue of diversity as a biological corridor is being discussed. The area has other functions, but when this measure was initiated, it was not with that function in mind. The original idea was to use it as a barrier to prevent nutrients from reaching the course. Now the whole issue is being rethought. There are challenges, and the second generation of measures, which have already been approved, have not been regulated or put into practise. Other measures, such as restoration of native forests in some parts, are also being considered. The lowlands are very degraded and can act as biological corridors with more ecosystem functions than we are currently seeing, but we need to focus more on biodiversity.

Interviewer 1: Are there characteristics that support these functions that differ from the others that have been mentioned?

Interviewee 1: The main characteristic is the variable width. For the second generation of measures, the focus is on conserving the areas that have priority from the point of view of the biodiversity of species. The idea is to divide or create strips to protect certain environments. Wetlands are also important because of the functions they have. Buffer zones can be used to facilitate their application by producers.

Interviewer 1: What functions do you think the buffer zones should have in the Santa Lucia river basin?

Interviewee 1: It is important for producers to have full knowledge of the importance of the function they fulfil before applying them. The issue is that the producer may lose a productive area when applying this buffer zone. And as a consequence, it loses economic income, let's say. So, that's where a cost-benefit ratio comes into play. Today, only the producer is paying for it. There is no support from the government to exempt the contribution. The only precedent that is appearing and that we are seeing are some situations carried out by the National Institute of Colonisation, which has asked its settlers to apply a differential rate to the cost if their registration is within the buffer zone. It is not yet general but rather on a case-by-case basis, where each settler goes and asks the National Institute of Colonisation to apply the differential rate to the cost. They pay rent for that area. So, if the registered area instead has a rent of 8,80 dollars, so to speak, they multiply it by one. But if it has an average rent of 8, they multiply it by 0.75. In other words, they receive a discount that somehow doesn’t feel like a payment for them but rather a waiver of rent in that area. What they are asking for is some sort of exemption. We have seen some background. Out of the 3,000 registrations, we have seen 4 or 5. But, well, it's opening up that way, and there's something written about that possibility in some regulations. You would suddenly have to talk to someone from the National Institute of Colonisation. We have received minutes from the board of the National Institute of Colonisation where they say that if the renter complies with the measures needed, that is to say, no modifications to the plant cover, no tillage, and no application of agrochemicals, it's okay to receive that applicable waiver. With regard to that, we can pass you a contact who works in colonisation so that you can talk about it in more depth.

Interviewer 2: Do you have any idea how many colonisation plots there are in the basin, or at least how many are affected?

Interviewee 2: They must have a piece of land that should be rented, but we don't have the exact number. However, the National Institute of Colonisation is part of the government that rents land to farmers at a very low price, so it is quite common. We talked about possible changes in the function of the buffer zones.

Interviewer 1: What features do you think need to be changed or added to the design of buffer zones to support these changes, for example, to allow for horticulture, the introduction of new species, or ecosystem management?

Interviewee 1: In principle, the regulations need to change to allow for it. Currently, the regulations don't permit it, so legally, it cannot be done. Knowledge needs to be generated to establish a baseline, which we don't have. The question we frequently ask is whether the buffer zones are working today. This initiative began in 2015, eight years ago. Are the buffer zones retaining or contributing nutrients to the water resource? Are they sources or sinks? We lack this information at the ministry or country level. Although it may be a bit late, we should start with a survey of these buffer zones. This will enable consideration of different management approaches, such as animal grazing, authorizations for crops that extract greater amounts of nutrients, or vegetative felling. However, there is nothing definite, as the regulations currently prohibit any intervention at this stage. The benefits of these changes or additions to buffer zones include moving animals away from the water source and vegetation to harvest nutrients, which are then transported elsewhere. In the case of vegetative felling, harvesting the grain or plant and taking the nutrients for food or sale outside the farm or watershed would be ideal. It would also decrease the nutrient preservation that the buffer has (e.g., nutrient gathering), which would be a positive outcome of that kind of management.

Interviewer 1: And what obstacles do you think will be experienced with the implementation of these or additional changes?

Interviewee 1: First, legal—that's the first one. And then there are changes in habits. People have adapted to doing their work and tasks in a certain way, and they will have to adapt to the proposed changes. For instance, asking the producer to graze animals at a low stocking rate means fencing or delimiting that area. The producer will not be able to move all the cattle that they had in a lot to the new grazing area, but they will have to rotate some animals. This will mean more work. Change is always difficult, and I think that the obstacle is changing the culture as a whole. It's not just the people; the animals also have a certain culture. They are used to taking on a certain behaviour with the water, and it's natural. In the river and in the streams, nobody sees it as a problem, and in reality, I don't know if it is a problem, but it is an important nutrient source for the body of water when the animals are there. We were talking about it today, and it seems crazy to say that cows can't drink directly from the river without getting sick. So, there needs to be a pretty important cultural change.

Interviewer 2: In terms of the legal aspect you mentioned, what would the changes be? What are the barriers, for example, in the built environment?

Interviewee 1: In order to apply new things, the ministerial resolution that is regulated today says that you can't do anything. So, you need another regulation or ministerial resolution that enables grazing to be downgraded or enables the sowing of maize and legumes for the extraction of phosphorus. There needs to be a regulation that allows it. All of this takes time. You have to create new regulations, disseminate them, incorporate the producers into the change of culture, and get all the actors in society and the government to agree so that when they reach the authorities, they can be signed. Management is not easy, and agreements between political forces can be complicated. It takes time to get all the actors to agree.

Interviewer 1: How do you think these obstacles can be overcome, or what possible solutions do you think can be found for them?

Interviewee 1: Well, generating knowledge and providing education, awareness, and appropriation of the measures and knowledge is crucial so that whoever applies them has a full understanding of why they are doing so. I believe that's the most important thing. Additionally, gaining support from all parties involved is crucial. Of course, as environmental care is sometimes opposed to production, achieving a balance is necessary. One crucial aspect of achieving this balance is communication, dissemination, and cultural change. However, it's difficult to pinpoint whose responsibility it is to drive this change. From a regulatory standpoint, the Ministry of Environment should take control and ensure proper dissemination and communication of regulations, but this was not the case in 2015. The Ministry of Livestock should also support this effort. The Ministry of Livestock has the best relationship with agronomy producers and has more contact with them through development roundtables. They work together a lot and have crucial contact with the producers. Therefore, it's important to achieve an alliance between the Ministry of Environment and the Ministry of Livestock. Recently, they have begun working on a sustainable production plan, but much more needs to be done. Achieving this cultural change will require teamwork, with the Ministry of Environment controlling environmental regulations and the Ministry of Livestock supporting and disseminating them. Additionally, communication and environmental education are critical, especially as we are entering a new era of the Santa Lucia River Action Plan. There are small projects in different areas. For instance, in Media 5, which is located in the Southside lagoon, there was work done with producers and tremendous awareness was achieved. However, these are just small impulses in small groups. Other projects have achieved successful implementation, and it was implemented before Media 8. It has more history and is smaller, which changes the perception of the type of producer. In the Santa Lucia basin, there are many milk producers, which is another change. The Rocha lagoon (where these projects were located, red), also known as the Swan lagoon, is the most progressive. The Ministry of Environment has taken that forward. There is also someone in the territory who works for the Ministry of Environment and is known to be in the Rocha lagoon. One colleague has been working for many years in the whole of Maldonado, for about 10 years in the ministry. She has managed to establish a good link with the livestock people, and they work together. However, the Rocha lagoon is smaller, and there are more livestock producers and a bit of culture that sets it apart from other areas. Therefore, the conflict is probably not so big, as it is a public-private conflict.

Interviewee 2: Exactly. Yes, they are working very well, and so we give them support from here. That is in the community of Santa Lucia. That doesn't happen; there is no one from the ministry who is in the territory working on the team with us. There are those who work on the team with us, and there is another culture of work and what is coming. There are also those who are very old; they always threw the effluents into the river, and nothing ever happened. Now go and tell them that this should no longer be done. All we are talking about is change.

Interviewee 1: Yes, sure. It's not a one-time thing to implement buffer, but it is something more complex, Alfred; it's a continuous gesture. A small transition is missing from the recording due to hardware issues.

Interviewer 1: How do you experience climate change in the Santa Lucia River basin?

Interviewee 1: It’s very noticeable. Especially the current drought. Hectares of soya have been lost, dairy farmers don't have food or water for their animals, and production has fallen. At the moment, since January, there has been a European livestock emergency in a large part of the country, obviously in the basin, and climate change has affected it a lot, and not in the best way.

Interviewer 1: Sorry, could you repeat the first part? I forgot to turn on the recording.

Interviewee 1: All right, we were talking about the water deficit. We would have to review the values, but since the winter of 2022, rainfall has been lower than the historical average. This has meant that the availability of water for crops in the soil has been very low. Going into the summer, this low availability of water in the soil, coupled with high evapotranspiration due to the high temperatures that have been recorded well above historical levels, has caused many hectares of crops planted for winter reserves to be lost. Some of these were planted for grain harvesting, which means that both current and future production are compromised. At the moment, producers are facing difficulties with food and water. In fact, the population of the country is also struggling to access drinking water.

Interviewer 1: How do you think these changes will affect the performance of the buffer zones in the Santa Lucia Basin?

Interviewee 1: Well, for example, the watering of animals in the watercourses is not controlled because it's the only option that the producers have. We can't tell them not to do it. As there is low water flow in some areas, high temperatures, and high nutrient content, many blooms are occurring. However, when the rains come, these high loads that were suddenly concentrated can be diluted in the course, and those that are on the ground in the basin's area will likely run off into the course, which could be problematic. In summary, recent times have probably seen an increase in nutrient concentrations, and this has negatively affected the buffer zones. Although the nutrient content in that strip is not being controlled by satellite, we need to be sensitive to the situation. We cannot go and tell producers that regulations do not allow animals that have access to the strip to take water from the river, as it's not the most politically correct thing to say. In other words, the production system is not ready to face a drought such as the one that occurred in Uruguay's history. It could be faced, but we need to adapt, foresee it, and make the necessary changes. Many producers are suffering and having a hard time due to the lack of water for their animals.

Interviewee 2: I believe that the effects of climate change on buffer zones will be significant, even if the changes proposed in the previous question are not implemented. Alfred, could you repeat what the proposed changes were? I don't remember. Do you recall what they were?

Interviewer 1: No, I can't recall either.

Interviewee 1: However, it occurs to me that this situation could be foreseen by generating water reserves, multi-predial reserves, and food reserves. Currently, there are only around 110,000 hectares in the country that can be used for summer crops. By implementing regulations to promote better management, more hectares could be cultivated to prevent these situations from happening. There are things we can do to improve and be better prepared for these changes, but we are still relatively new to dealing with events of this magnitude. This is one of the biggest droughts in history, and it has caught the country off guard. As a result, there is a need for policies and contingency measures to be put in place quickly.

Interviewer 1: Guillermo, do you have another question?

Interviewer 2: Yes, you already mentioned it above, but how are the controls carried out by the Environmental Control Directorate? What are the means of control?

Interviewee 1: Well, in reality, we carry out systematic control, which includes satellite control. Currently, we do it using the images available from Google Earth, but we are working on developing an algorithm so that we don't have to do it manually. Currently, we do it visually, register by register, looking at each one. Then, there is control in the territory, which includes complaints from actors in the basin who report situations that we must control. This is not systematic and generally arises due to a conflict, triggering a complaint. We also have systematic satellite monitoring, which we conduct every six months.

Interviewee 2: As mentioned, we look at images, no different from Google, and create a list of standards that we consider red, indicating noncompliance with the measure, farming activity, or other issues visible in the strip. With this list, we conduct inspections in the field. We prepare an inspection plan and then go around the territory. In other words, there is a periodic inspection plan that we use in conjunction with the input from satellite control.

Interviewer 2: Regarding the second package of measures, which raises the possibility of grazing or rotational, intermittent management, do you see any possibility of satellite control over that? How do you see that?

Interviewee 1: No, satellite control is not possible. We cannot see the animals. What we could do, I imagine, is ask the producer to provide data on soil bulk density and phosphorous in the soil. If the bulk density decreases over time, it indicates excessive compaction, and obviously the loads are not being respected. If phosphorus levels in the soil increase, it doesn't necessarily have to be all from grazing, but management needs to be checked. That's where it comes into play. The inspections are also unannounced, so you arrive, and if the job isn't being done well, you can see that the nutrient load isn't as agreed upon. It's difficult to control everyone with the number of producers and lists that exist, so a sample would be put together for control. It would be voluntary at first and then mandatory. Someone mentioned the use of drones, since nowadays this technology is advanced. It could help to reach producers who are difficult to contact, especially on irrigated properties. With a drone, we could easily access the buffer zone and monitor compliance without the need for the producer's authorization or presence. It's not that far-fetched of an idea.

Interviewer 2: Do you see any limitations in terms of budget or anything like that?

Interviewee 1: It's an issue that I imagine is relevant for the ministry, and there are some more. They try to manage the quality of work of people when they do all the water monitoring and that kind of thing and train people to act sustainably.

Interviewee 2: Yes, the biggest thing, I think, is the budget and that. The budget issue is also about who is responsible—the municipality or the social welfare bank? I mean, here they used this control mechanism as a tool. The Social Welfare Bank handles the controls.

Interviewee 1: Yes, the issue of works and things like that, like construction works, is that contributions are being made for that work. They do it with drones. And nobody discussed it; that was not discussed. The legal part is also an issue, but I don't think it's very difficult because it was already implemented.

Interviewee 2: No, because you do it at a certain point that is no longer private property. It belongs to the state, let's say.

Interviewer 2: Okay. Another question that comes to mind Do you have a satellite analysis division within the ministry?

Interviewee 1: Oh, yes, there is a division of environmental information systems, which is like a geographic information system.

Interviewer 2: Oh, who does that depend on?

Interviewee 1: That depends not on DINACEA but on the planning area. DINACEA is divided into planning, control, and impact divisions. Anyway, I was thinking that with an NDVI index, they could also see sudden changes in a low area that should be still or in a certain way. The working group is developing an algorithm based on NDVI that takes into account the phenological changes of the crops. The algorithm is still in the training phase.

Interviewer 2: You mentioned the soil management plan as a possible tool to bring the implementation to another level of the buffer zone. How do you see it? What emitters (users, red) do you see for that tool?

Interviewee 1: It could work in an alliance. It's technically possible and already in place. We just need to add a module to it. Regarding watering, measure 7 (of the Santa Lucia River Basin Action Plan, red) projects watering.

Interviewer 2: Do you have any idea of the percentage of prices that are in compliance or non-compliance? What happens with this measure?

Interviewee 1: The plan had a battery of 11 measures, but none of them are regulated. They can be sanctioned, but there is no regulation to back up that suggestion. Measure 3 states that the 31 parts per million of phosphorus are also not regulated. Work is being done to regulate these measures. If something is regulated but can't be controlled, it's a big issue. And well, actually, in the case of phosphorus levels, the most direct thing is sampling soils. Obviously, I have done it with my trained technical personnel who know how to do sampling and a reference laboratory. Then, I think it would be more a matter of education and working on responsible nutrient management. In other words, applying what is needed for the crop Yes, there is a very nice example there in the eerie lake.

Interviewer 2: I don't have any more questions.

Interviewer 1: Good. Me too; I love it.

Interviewee 1: Well, nice to meet you, Alfred. We're at your service for anything you need.

Interviewer 1: Thank you very much for your time.

Interviewee 1: The schedule changes and all, but don't worry; we're at your service. You have our email address, so feel free to send it to us, and we'll coordinate.

Interviewer 1: That's great. Thank you very much for your time.

Interviewer 2: Thank you very much for your time.

Interviewees 1 and 2: Bye-bye. Good day.

Interviewers 1 and 2: Same to you. Good day.

*Interviewer 1 is the student, and interviewer 2 is the supervisor.*

# Interview 13

**Interviewee: Producer            Date: 16-03-2023**

Interviewer 1: Are you familiar with riparian buffer zones?

Interviewee: Yes, I am a dairy engineer who runs and owns a dairy farm. When I graduated 30 years ago, I had the opportunity to travel to Europe and witness the environmental issues and new European Union requirements. Upon returning, it became clear to me the importance of buffer areas, the location of sand mining, wells, and effluent management, which was in its infancy in Uruguay at the time. I have always attempted to implement preventive and management measures. This is an atypical dairy farm, as it has been in my family for generations, originally owned by my studious grandfather (who was a doctor) from Porongos. He had a clear vision of the importance of buffer areas, avoiding ploughing flood areas, and recycling organic matter and manure. Since its establishment in 1923, they have always separated solid and liquid manure and recycled it in meadows or on the farm. Maintaining these practises has been a challenge in the Santa Luca river basin due to cultural issues, not only among producers but also in Uruguay's agronomic training, which is lacking.

Interviewer 2: Where did your grandfather learn these practises?

Interviewee: Well, he was the son of a Catalan immigrant. In Catalonia, for example, he had an older brother and received a good education but had no money. He came to Uruguay, married an Uruguayan woman in Montevideo, and then settled in Porongos. However, he was always concerned about his children's education. Believe it or not, he received a good primary education in Porongos at the end of the 19th century. He then attended high school in Coroa Valdense, where Father Armanduón taught. This high school is now recognised as the best high school in Uruguay. He was a studious guy, and as a medical doctor, he graduated with honours from the Faculty of Medicine. He had the chance to go on a three-year scholarship to Europe and the United States. As a doctor, he was always clear about the whole issue of hygiene and the environment, and he had a concept of a single health: the health of the animal, the health of the environment, and human health. Even when he started the dairy farm, tuberculosis was a serious animal and human health problem. By 1927, for example, he had contacts with the Institut Pasteur in Paris and was already studying the problem of brucellosis. He applied the VSG to the calves, and a year later, he was able to eliminate all the positive cases and have the first tuberculosis-free herd in Uruguay. This was quite unprecedented because he read the latest work in Europe and immediately applied it before the VSG was used on children in Uruguay. He was also concerned about environmental, human, and animal health. He was the first scientist in Uruguay to discover brucellosis, which is a disease that causes abortions in cows but is not known to affect humans. He published the first papers on brucellosis. He was always a very advanced person, a doctor and a great surgeon, but he was interested in a broad culture. His interest went beyond medicine, and he believed that a doctor who only knows about medicine knows nothing. He studied agronomic issues, environmental issues, political issues, and more. But it's surprising that there was already a separation of solids, something that people are now trying to implement 100 years later. Of course, one must also look at the culture that European immigrants brought with them. My family is of Catalan origin, and Catalonia is a relatively poor land. Compost and many of the new concepts that we now talk about in agroecology were already incorporated into the cultures of European countries. Compost was valuable and was used frequently. Uruguay was a country where there was a rapid process of acculturation, and we lost the European culture that we brought with us. We became brutalised, but I am sure that your ancestors had important rural education at that time. Because back then, there were few big cities, and they were not so populous. Until the 19th century, there were many people of rural origin who practised subsistence agriculture. They had chickens and pigs and lived rurally (quinta, red). Food had to be preserved because there was no refrigerator. There was know-how and knowledge that were transmitted from generation to generation. This culture was brought by the immigrants and is preserved by many people from the Canelones area. However, the mentality of the gauchos (cowboys, red) of the ranch, where the cow was just to marry and eat, represented a significant change in culture. José Artigas brought about that change, but I do not want to distract you.

Interviewer 1: Do you have buffer zones on your land?

Interviewee: Yes.

Interviewer 1: Do they generate any problems for you?

Interviewee: No, I see the buffer area as a necessity. One protects the watercourse with vegetation, and it is also beneficial in the sense that it protects against erosion. The natural field preserved as a buffer area also has its resilience and advantages. I am an advocate for improving buffer zones by adding leguminous plants, controlling fertilisation in reasonable doses, and preserving gallery (native, red) forests. I have a park-like formation that I have been advancing and trying to maintain through pruning. I showed you an aerial photo on your mobile phone of what this area looked like so that you could see how invasive exotic species, such as hawthorn and blackberry, among others, have advanced. If you compare it with Google today, it is impressive how the vegetation has advanced. In reality, it is often a response to poor grazing management, where sometimes, due to overgrazing, invasive species that may be native, such as hawthorn, start to appear or advance.

Interviewer 2: Is the hawthorn here also from this area?

Interviewee: Yes, it is native to Uruguay. On the northern coast, where I graze, the Espinillo (*Vachellia caven*) and the Pajonal (*Paspalum quadrifarium*) are considered native, invasive species. There are invasive exotic species like the Ligustro (*Ligustrum lucidum*), Gleditsia (*Gleditsia triacanthos*), and blackberry (Zarzamora). They are all around here. This is also a product of the fact that suddenly, many years ago, grazing was not as rational as it is now. Now, grazing has become more rational because you have the electric shepherd. At a certain moment, you had more fodder reserves. Then the cattle would starve and overgraze. By making good fodder reserves, you can keep the cattle well-fed, give the land a rest, and avoid overgrazing, which is a source of pasture degradation. Are you trained as an agronomist or an agronomy engineer?

Interviewer 1: No, I am not. I am not educated to be a farmer either. Wageningen is a university focused on agriculture. With my studies in water management, it's a combination of soil, land, and water management. Do you know what that is?

Interviewee: Yes, it's suitable for agronomics.

Interviewer 1: Okay, good. You mentioned different purposes for a buffer zone, for example, to reduce erosion and protect the soil. What characteristics of the buffer zone do you associate with the current buffer zones in the Santa Lucia River Basin that support these functions? What characteristics of the buffer zone meet those benefits that you mentioned?

Interviewee: First, protecting the soil from erosion Then, if you conserve the native forest, thinking of silvopasture management, the cattle have shade, and you can have firewood; although I don't understand the use of firewood, it can be part of the management.

Interviewer 1: As you were explaining, buffer zones generate benefits, such as the reduction of erosion. What other benefits does it generate?

Interviewee: What I see is that when you make a traditional pasture, in general, you plant exotic species of clover and grasses. However, buffer zones have permanent pastures that are more resilient. During a time when the natural pasture gives you less forage or you can't use it, you can use the buffer area. This field is medium-sized, but for example, I have a part of the field where I have a designated forest. The forest had advanced, so I preferred to designate it, and I tried to maintain it with pruning and clearing, controlling the Zarzamora and the Ligustro. Then I have a part that is actually called low fields, which are floodable fields when there are large floods. There, I maintain the native vegetation, but I have added, for example, lotus, which is a leguminous plant that improves the nutritional quality of the native mix. And then in that natural field, when well-managed, the meadows give me a lot of forage in spring, but when I get to late spring, I make good use of that area, which is a barren area, but at the same time, I have a good response in terms of forage production. The cattle take advantage of it. These are productive benefits. In reality, what it does is produce sustainable benefits because the permanent grassland is conserved, and all you do is collect the growth of forage to encourage regrowth because the forage plants have a growth rate that is called a certain rate. So, after reaching a certain level, if you use it, it's good because you take advantage of that fodder. But the plant returns with more vigour and captures more carbon, and in short, more is produced. I think the buffers are very important, firstly because they are a filter for the watercourse. I've been to the basin commission and talked to some agronomists from the ministry, and I'm a bit annoyed because they suddenly told producers to implement them. But they should visit and explain certain things to them, but I see that they don't do anything. Here, for example, it's also important to understand a bit of history. In these areas, for instance, the Uruguayan Society developed in the Santa Luca River basin not only because of the water supply but also because the basin supplied Montevideo with fruit, milk, vegetables, and so on. In the past, dairy farms were located quite close to Montevideo, and at certain times, dairy farms were banned in Montevideo. So, there was a train in this area, and that was an important development for small dairy farms. For example, if you look at them later, if you want, I'll show you, but within a few kilometres, you have a lot of small dairy farms that existed, and those people managed them. They were close to the river; they had water; they had fodder; and they had the train nearby to take the milk. Many of those dairy farms have been disappearing. Now, what has happened? Many more dairy farms have disappeared than is desirable, and suddenly foreign investors have come—Argentinians, for example, who have a completely different vision. They come, for example, and start up all the forestry areas. Did you see in the wired fences where they grow trees for felling? Those who, well, place the wired fences, make large farms with monocultures, and apply pressure to the edge of the watercourse One tries to do things right, but we have a commission that does nothing. When people from the ministry are seen, I then show you aerial photos of these developments. For example, I have a neighbour here who, at a certain point, began to do that kind of management. One day, I called him and asked him, "Please do not start on the Ombú " (*Phytolacca dioica*, native tree, red). There was a big, beautiful Ombú against the gate, bigger and prettier than the one that appears in a profile picture. So I called him and asked him, "Please don't start it, and they started everything—mills, ranches, so that was also like, "It's a big problem. One sees that it's good that there is agribusiness, but there are limits. I see that the ministry is not doing anything now; for example, the ministry changed the regulations to benefit OPM (organización de prevención meteorológica, red.) with certain sanctions. I don't know if you heard about that. These are the kinds of things that make some people feel indignant. So many government employees are trained but locked up in offices. They could easily take Google Earth and go out into the countryside to see the barbarities that are being committed. As a child, I used to come here to the dairy farm in the area because it is also an effective issue, and I want my family to come and have a good time. But suddenly, a foreigner who lives somewhere else and doesn't care about anything comes and uproots trees along the course of a ravine. The gully that had meanders that made the water flow more slowly eliminated the normal flow and made a kind of drain. The water there passes very quickly, leaving traces and applying resistance to the same watercourse. The people from the ministry should be told about it, not so that they will sanction him. I think that the image of the agricultural producer is mentally discredited by a lot of people who do bad things and also by a lack of education.

Interviewer 1: What purpose do you believe the buffer zones should have for you or your company?

Interviewee: In my case, I try to differentiate the environments. That is to say, the property has different environments, and according to the environment, one can make an active, productive theme, and in certain environments, another one. So, in reality, I kind of accept certain areas where it will not be productive to try and make sustainable management efforts. I mean, you also have to differentiate between different degrees of buffer zones. For example, here against the river, properly speaking, I call it the "coast guard", where the cattle cannot enter under any circumstances. The animal does not enter the riparian forest, so it does not take water from the river. Then, I have water distribution in the plot through which water arrives. Now I am waiting for them to come and fix a pipe that broke, so I have all the cattle concentrated here. But there are degrees of buffer zones. There are buffer and restricted areas where the cattle do not enter, where nothing is done in the riparian forest, and then you have the formation that I call the "park type", where normally there is no water. I have three levels: the riparian forest, then the park-like forest, and then what I call a "bajo" (meaning: low, red), which is the twelve hectares where there are practically no trees, which is a field that I manage. In the natural area, I added another one, which is a kind of summer pasture. I don't know if you understood the concept; they are three different buffer areas or three different degrees of buffer intensity.

Interviewer 2: In terms of management, what is the management of this first riparian woodland that is built with the exclusion of cattle?

Interviewee: In reality, it is a riparian forest where there is practically no useful ground cover, as the ground is covered by the shading of the vegetation. Not that there is no grass; there are species that are not forage species. Then I will give them (government personnel, red) a look because it is also good for them to see. But what you see is a large number of desk technicians who have no idea of reality. My father was a doctor, and I was in charge of trying to raise the cattle. There are low fields with drainage problems, and I was always interested in the whole subject of earthworms. I read Darwin's works at the time, and I don't know if you have ever heard of André Boazán. André Boazán is perhaps the father of rotational grazing. And although it may seem strange to you, André Boazán was a great scholar, and he had published several books. Agronomy students did not study them here, and I had to go to the veterinary faculty to photocopy them because there is a lot of philosophy behind Boazán. Boazán emphasised a lot the topic of earthworms, and I remember that when I just graduated, I went backpacking around Europe, and I didn't know a bit what it was going to be because I went through Wageningen. I remember going to talk to a soil professor to ask him about the topic of earthworms, but that was all forgotten. What is your name? Alfred? Then with Alfred, maybe I can look for it, and we can take it up again and learn how, or not? But the role of earthworms and the approach of agronomy are more agroecological. And do you study agroecology there?

Interviewer 1: A little bit.

Interviewee: Because I think that we need to have a more agroecological approach, and the training of agronomists in this area is not very good. Matt Bowman is an agroecologist who specialises in pharmacies in Holland. I think she will give you her number, and I think she will help you a lot.

Interviewer 1: That would be amazing. Thank you for the recommendation. What purpose should buffer zones have for society?

Interviewee: Well, I think, first of all, it's like a soil protection zone for filtering the water that goes to the river and a biodiversity conservation area, which is extremely important to me. I don't want to get too romantic, but there was a Catalan lady who was a friend of my grandmother and was exiled in Uruguay during the Franco era. This woman fell in love with the flora of Uruguay and considered it a treasure. She dedicated herself to painting all the flora of Uruguay, the beauty of the plants, and some of the plants she painted here, in my grandfather's field. She was here during the Spanish Civil War and was exiled because she was Catalan. Franco would have killed her if she had stayed. But she was here in 1940, a very cultured woman with great sensitivity, and she had contacts with a Uruguayan botanist from whom she learned about taxonomy. She painted all the flowers of Uruguay, and when she was old, before she died, she chose to publish a book for the public. But I can give you a photocopy of the introduction and all the beautiful stuff. All of these resources are valuable to me. For example, my daughters love to go to the river, and this small field has value as a recreational area. It hurts me to see the river put under pressure. I don't know if you have studied the issue of the sand pits (e.g., sand mining in the Santa Lucia River, red). I haven't studied it, but I know that in life, you have to live and let live. The issue is about order, and it's brutal to see the damage that has been caused to the river in just a few kilometres from Santa Lucia to Paso Pache. It starts at the new bridge. I don't know if you saw this new bridge that goes to San Jose and how good it is, but they cut down all the trees.

Interviewer 2: And on what route?

Interviewee: Here, on Route 11, Then we have another bridge, which is also good, but there's a big upheaval of the gullies and sand pits. There are eight of them in just a few kilometres. The sand pits have to make a path to get to the river, and at a certain point, the riparian forest starts, but then they extract sand in an industrial way without any control. All of this affects the watercourse. Meanders are erased, and the river loses its meandering nature. If you ask me, I don't know, but science should tell you how to remove the sand without damaging the environment. Now I can take you for a ride in the truck and show you. But first, take a look at the photo I gave you. Look at the field where I live. There were all these sandy areas. I never removed a gramme of sand from this white area, right? (While showing the photos of the sand mines in red, This is the home field; all this white was sand, and all of it disappeared. Then there were meanderings. I will now pass you all the photos. I know the area well; all this forest is to improve river management. And this is why I tell you: this is like the civilization that existed around the Nile.

Interviewer 1: What features should be changed or added to the design of the buffer zones to support these functions?

Interviewee: I think that in order to support all these functions, educating people could play an important role. For example, the improvement of lowlands is important where the natural vegetation is conserved, and that can be improved from a productive point of view while conserving biodiversity and avoiding overexploitation of the resource. I think that in the end, the essential thing is biodiversity and soil protection. The big problem, for example, is that Uruguay is a livestock-farming country. The issue arises when 100% of farmers come and want to farm where they shouldn't. So when you have a livestock farming establishment, for example, a dairy farm, And you farm in order to have good pastures; you rotate the corn and then go back to the pasture. So there is an agricultural phase of a year where you suddenly have two crops that are called "verdeos": oats or corn, and then you move on to the pasture. Oats or corn, and then you go through a phase of 4 years and 5 years of permanent grassland. So this is agricultural and livestock integration. If you have agricultural and livestock integration, you can at some point use the buffer areas for livestock farming in a sustainable and well-managed way. We have to bear in mind that before the introduction of livestock farming in Uruguay, instead of having millions of sheep and cattle, there were millions of ruminants that were deer. In other words, there were always ruminants. We should not look at them as enemies. I think it is an ally that should be well managed. I don't know if you heard more or less about Uruguay before the introduction of livestock. Before the Europeans came, there were millions of other ruminants, such as deer, that also grazed. There is an observation that, in terms of methane emissions, if we look at before and now, I don't know if the difference is so clear in terms of the problem of methane emissions from cows. I think it was Walter Baethgen who said that. Come if you want me to show you a little bit (interviewee commences to show around his farm, red).

(As the interview continues, click red.) Interviewee: Over here, the water and the fertilisers go to a trap, where the solids are separated, and then they are thrown onto the field as fertiliser. The water goes to a lagoon, and from the lagoon, it is irrigated. Now, I'll show you how. You see, I have also worked on the topic of effluent management, especially the economic part.

Interviewer 2: How is the yield? Does the liquid, for example, have any effect that generates any effect on the farm?

Interviewee: I haven't really evaluated it, but fertiliser is fundamental because it is organic matter, it has micronutrients, and that has to be returned to the soil. Because it is concentrated in one place, it pollutes, but when it is distributed, it is a virtuous circle. It is fertility that you return, and I think that what they should look at is what facilities or how they give the producer facilities to make these investments because they are very expensive investments. At a certain moment, I took the decision to do this, and my fellow producers in the group told me that it was nonsense because I had put in all this extra work to make the lagoons, etc. So what happened? I started, and I ran up against the lack of those desk agronomists who don't know anything, who don't go to the countryside, and who wanted to block my approval. So, well, I had to justify it, so I sort of drew a little something to get it in.

Interviewer 2: I have worked on this issue two or three times, and it is discussed whether it is necessary to enter, and I think it is because these are investments that don't generate income, do they?

Interviewee: They are very long-term investments. I mean, these investments not only involve putting money in but also ensuring their maintenance. Moreover, they are part of Uruguay's patrimony that needs to be preserved because the people who produce them, such as those working on a dairy farm, are treasures. They produce for their offspring, their farm, and the community. For example, on a dairy farm, there are three families working on 20 hectares of land. However, there are various people involved in the production process, such as those who control the land, the veterinarian, the agronomist, the seller of inputs, the seller of nitrogen, and the seller of fertiliser. There are so many externalities involved in dairy farming that it is an intensive sector. It has spill over effects that are not present in other sectors, especially in natural fields. Therefore, it is important to preserve this sector. For instance, a solid separator and irrigation system alone cost around $40,000, which is a considerable investment. There are also other expenses, like the pickup truck and the iron structures. However, despite all these expenses, I jumped into the dairy farming business 30 years ago after seeing its potential in Europe. I thought it would be a 20-year investment. So I hired a company and made the necessary preparations. I was aware of the challenges but believed it would be worth it. The investment in dairy farming involves many challenges, and one of them is the lack of automation. One of my neighbours does everything by hand, and it can be overwhelming. Therefore, it is crucial to have a team of professionals and experts to help with the production process. He is located in a more complicated place than mine because he is closer to the river. But he, for example, sees what I do and tries to copy it. He also made a shade for cattle, but he did it by hand. When we were kids, we heard the story of the three little pigs who built houses out of brick, wood, and straw. I always tried to make things out of straw. For example, I used the shades that dairy farmers make for their cattle, but they are usually handmade and become unstable after two years. I also needed electricity, so I made a shade with solar panels, but I couldn't afford it. Instead, I planted a few solar panels in the field and used them to make shade for the cows. Now, theoretically, I am generating electricity while the cows have shade. The electrical engineer from other countries who designed the system calculated that I would be at a break-even point, meaning that I wouldn't have to pay for more energy than I generate. In the summer, when I produce more energy, it credits me for the winter, when I produce less. I don't want to pay 40 or 50 thousand pesos every month because there is a family of dairy farmers and three other houses that use electricity as well. It's interesting to see those figures. There are a couple of points I want to discuss. The first one is the issue of shadow, which has a direct impact on production. It was quite a challenge for me to manage water and manure while also ensuring the welfare of the animals and workers on the farm. I aim to make it a circular economy and utilise everything to its fullest potential. As you specialise in water management, I would like to know if there are any areas for improvement that you can suggest.

Interviewer 2: Moving on to my question, how many milking occasions do you have per year on average?

Interviewee: I have around 200 per year, but during peak season, which is spring, I have around 220. However, I am limited by the amount of land, as it is a dairy farm with many sheds from my grandfather's time. On another note, have you heard of a product called EM (efficient microorganisms, red)? It is said to be effective in managing odours and the composition of matter, and I have started using it. This ties in with the concepts of microbiology and agroecology. What are your thoughts on EM? They are Japanese cultures that can be used in agriculture. I'm interested in trying new approaches to farming that are more sustainable and agroecological. There is a group of dairy farmers who are part of this movement, and they use EM a lot. I got connected with them and started using it too. The dairy farming industry is often focused on producing more at the expense of the environment and the farmer's quality of life. But there are alternatives that are more circular and sustainable. I want to learn more about this approach and make changes to my property. One of the challenges is the recent drought that devastated everything. It's like putting out fires, and it's hard to plan for the future. But I want to educate myself and work with the ministries to make reforms that promote sustainability and diversity. Regarding the buffer zones, I'm interested in learning more about their physical aspects and functions. I also want to know more about the preferred attributes for farming and how they can be incorporated into my farm. Maybe we can meet up and discuss more about EM and agroecology. I have a few ideas: we could either go home or we could go visit someone. Alternatively, we could go somewhere else entirely. However, before we decide where to go, I have a question about Holland. Specifically, I know that the environmental requirements there are very strict. Apparently, the government is planning to expropriate and shut down three thousand farms to reduce emissions. But what kind of farms are they targeting? In Holland, the Dutch dairy cow is a symbol of their culture, much like how closing a dairy farm here would be a big deal. I'm curious about what kind of reasons are behind it.

Interviewer 1: It seems like the government, the policy sector, and banks have invested heavily in the productivity of these in Holland, and they've encouraged farmers to build more milking parlours and sheds. Now, however, they must comply with EU regulations, and the capacity has reached its limit. This focus on productivity and growth is something that is often challenged by the principles of agroecology, which promote a calmer, more sustainable approach to agriculture.

Interviewer 2: Speaking of agriculture, I'm concerned about a trend in Uruguay where dairy farms are increasingly overloaded with work. Instead of constantly striving for more production, maybe we should consider a more sustainable and less demanding approach that allows for a better quality of life.

Interviewee: During these times of drought, it's important not to swing too far in the opposite direction and demand too much from livestock farming. Instead, we should focus on improving animal welfare and overall management practises. For instance, there's a project called Climate Livestock Farming that I'm involved with, along with my colleague and 60 producers. We're working to reduce stocking rates, improve pasture quality, and enhance productivity even during dry seasons. By doing this, we can reduce the overall workload on producers and help them become more resilient to changes in prices or other factors. As an agronomist, I understand the importance of economic independence. My family runs a small business, and I've worked in various industries, including marketing and pharmaceuticals. I noticed that doctors tend to learn primarily from what the laboratories tell them, and this can sometimes lead to a focus on producing new drugs rather than considering other factors. A similar issue occurs in agronomy, where research is often funded by multinationals and focused on specific areas of interest, such as agro-ecology. While private sector funding is important, it can sometimes lead to research that is not entirely independent or objective.

Interviewer 2: Can you tell us about your experience in marketing?

Interviewee: Yes, I worked on the marketing idea for a while, but it didn't suit me. It's a fact that decisions in fields like agronomy and medicine should be more objective and less biased by economic interests.

Interviewer 1: Let's talk about buffer zones. What physical aspects of buffer zones support functions like soil protection, biodiversity, and productivity?

Interviewee: Vegetation can protect the soil and promote biodiversity. When climate problems arise, native vegetation can be more resilient than productive exotic species. All of these functions are important to me, as they help to protect the environment and future generations.

Interviewer: Can you rank these functions or physical aspects in order of importance?

Interviewee: I can't really rank them, as they are all important. It's a cultural issue, and we need to coexist with nature. For example, my family enjoys going to the forest for recreation. So, I realise that now, for example, tree baths are in fashion. Have you heard of it? Tree bathing, also known as forest bathing, In short, there are many treasures that we tend to overlook or neglect, which is a shame. Now, the buffer zone provides cultural and recreational benefits as well as protection for the river, watercourse, and soil. If I had to rank the importance of these benefits from recreation to nutrient retention, I believe that the protection of the river, watercourse, and soil is the most crucial one, as humans can spread everywhere. Although economic benefits, such as generating fodder, are also important, for me, the process and fodder are valuable. As an economist, could you help me propose a way to sell firewood from sustainably managed forests?

Interviewer 2: Producers who conserve the forest in a park-like manner can sell firewood with a seal of sustainable management of native forests. The forest is conserved through pruning, firewood removal, and grazing. I hope I made myself clear. By the way, do you have a guide with you today? I mean, are you selling it?

Interviewee: No, I'm not using it, actually. What also happens is that the personnel issue is also difficult. So right now, I have a person who is doing pruning, and I'm taking firewood for personal use, but they are all pruning.

Interviewer 2: Yes, yes. And where is it on the map? Because I was comparing the photo of that one that showed me, and the area you have is the largest.

Interviewee: Of course, it is large. I mean, I tell you, at a certain point, I said, 'What do I do with this?' I asked to uproot the bush because it was invasive, so I registered it. Maybe I was wrong to do it, but I registered it with no idea of maintaining it or controlling the Espinillo (*Vachellia caven*), but just trying to manage it as a silvopasture. But I still don't have that oiled, but it would be good if I could. Because if you are seen cutting down a tree or pruning, it's as if you are a sinner. In fact, if this is rational management, The issue is precisely how to manage these renewable resources in a sustainable way, from an economic and environmentally friendly point of view. So it's a whole issue. And there you are, doing some kind of new management. At a certain moment, for example, I gave it to a person. I took him to gather firewood, but he didn't do a good job, so I said, "Don't fill up anymore.' What did I do if he went a bit overboard? He overdid it a bit, and he didn't do the pruning the way I wanted him to. I made, for example, a passage against the wire fence, another passage here, and then the passage is like this. But if you compare, for example, you have your mobile phone there, and I'm going to show you here so you can see the difference.

Interviewer 2: (While comparing the map on his phone) Is this pond this?

Interviewee: Yes. Do you see it there?

Interviewer 2: Yes.

Interviewee: Look how the bush has advanced. And at the same time, see how the sand has disappeared?

Interviewer 2: Yes.

Interviewee: Yes? But I'll show you... See, for example, here my neighbour has a sand deposit, and he razed everything to the ground, see? Here, there was still vegetation, but it was all blown up to get the sand. And then... Here, we have another sand deposit. The river meanders here. There is another sand deposit here, and it's full. Another sandpit can be found here. So, I think you have to live and let live, but here is another sand pit. There is no control. So, by mining the sand, you take out the riparian forest. Suddenly, you had dunes that were fixed with vegetation. They took out the vegetation and sand, and then this patch happened. You can see a sand trap, two sandpits, three sandpits, four sandpits, five sandpits, six sandpits, seven sandpits, and eight sandpits, and here is another one. Then you come here and see the bridge. So, in a few kilometres, everything that has been damaged is from the river. My field is thin and long, and this is a neighbour’s property. Here, you have this first strip, which is the riparian forest, and there is no access for cattle. Then there is this part, which is like a kind of park. Suddenly, there is this part here that is native vegetation.

Interviewer 2: In other words, you have almost a kilometre of buffer. How many metres do you have, exactly?

Interviewee: Ah, yes, quite a lot.

Interviewer 2: Do you know how much buffer the regulation requires?

Interviewee: I think it's 50 metres; I remember now.

Interviewer 2: You have a kilometre and a half; the field is thin and long. It's not up to the coast, but it's still quite large.

Interviewee: Yes, I mean, I actually have areas where I don't have a fence; I have a kilometre and a half. But my neighbour has a different reality. For example, all of this that I have declared a forest, he has declared a field. And here you have declared woodland, but he does cattle farming.

Interviewer 2: So, is the forest declared for income exemption?

Interviewee: That's another issue that I would have to clarify. I have it registered, but every year they force you to do the paperwork and other things. All the bureaucracy makes it difficult for the producer who does good management, has a buffer area, and maintains the park-like formation. The paperwork for not paying for the real estate construction in that area should be automated. This is something that should be suggested. Where are you going to present it?

Interviewer 2: The idea is to present it to as many producers and decision-makers as possible. We will present it both in the Netherlands and here. I imagine that it will be interesting. At least we want to do a newspaper article or something like that. I'm here because I'm interested in the Rio Santa Lucia Basin Commission. Sometimes, you see a lot of bureaucracy and things that are on the cover of the book that the Minister of Agriculture doesn't tackle all of a sudden. Are you in Montevideo too, or where do you live?

Interviewee: My wife is a doctor; she lives in Montevideo; she comes here on weekends; and today in the afternoon, I'm going to Montevideo.

Interviewer: Alfred wants to bring you a little present from Holland, so maybe we'll catch up with him in Montevideo.

Interviewee: Well, I have a lot of sympathy for Holland. Well, for the Dutch cows Many years ago, I was in Holland. I had a Dutchman who looked after me very well, a certain van Belsen, who was an agronomist and worked in the field of genetic improvement. I have sympathy for Holland. I really like the pottery of Delft Blauw.

Interviewer 2: Ah, yes, yes. It's very nice.

Interviewer 1: I have three more questions.

Interviewee: Yes, tell me.

Interviewer 1: What would be the benefits of these functions in the buffer zones for you or for society?

Interviewee: First, I theoretically own the field, and I intend for my daughters to keep it. So, well, I think those benefits are sustainable and long-term. And I think that for society, the agricultural producer becomes a kind of park ranger, a kind of well-educated and responsible protector of the soil.

Interviewer 1: And what obstacles do you think we may encounter in implementing these functions and physical aspects?

Interviewee: In my opinion, if people are properly educated and things are done well, water quality will improve. However, the challenge is to take action and put plans into motion. For instance, the ministry will have to educate, monitor, and draw attention to things that need improvement. Although I am not skilled in detecting inconsistencies, it is essential to understand that certain soils have specific agronomic attitudes. It is not suitable for an Argentinean with an agricultural mindset to come here and try to farm in a way that does not align with Uruguay's practises. Regarding the issue of erosion, I can show you an aerial photo of the location. Although it is not clear in the photo, there is a gully with meanders that has been covered by a channel. This modification hinders the natural water cycle, causing problems downstream. The ministry can control and monitor these issues as they have access to aerial photos and Google Maps. They can detect areas where things are being done well and areas where things are being done poorly. Instead of immediately issuing sanctions, the ministry can explain to the offenders what they are doing wrong and encourage them to make amends. For instance, let's consider this location. An Argentinean came and destroyed the buffer zone by tearing down trees and fences, including a beautiful native tree. Additionally, they removed the gully, which was a significant loss. If the soil is not purely flat and agricultural, it serves livestock. If you do not consider that they lost many, we are counting that we cut down trees that provided shade at some point. This is a pity.

Interviewer 1: For the last question, what possible solutions do you think can be found for these obstacles?

Interviewee: The solution, first of all, for me is to educate and to look for some kind of tangible benefit for the producer to act as a guardian or protector. For example, being exempt from property tax would be a good thing. The problem is that I don't do it because you have to do all the paperwork over again. In other words, it gives the feeling that they want to block everything instead of making it easier for you. So, the government should say, "These are the buffer zones,' and identify areas that could have certain productive uses. The producer should be incentivized by offering certain exemptions or benefits for doing good for society. But the government should also give them something in return because being an agricultural producer in this country is difficult, and you have to constantly invest and fight against the climate, for instance. So, I think it is important to prioritise the role of the agricultural producer for the good of society and, in some way, to seek some kind of reward for doing things well in these buffer areas. Perhaps an exemption would suffice to motivate the producer. As I have to manage the financial and human resources parts, as well as several organisations, there are many demands placed on the producer in terms of health and environmental requirements. They need to find a way to assist the producer and get them on board. I won't take up any more of your time now. You can go to my neighbour, and then, if you want, I can give you a tour another day and show you around. Are you able to continue with my neighbour?

Interviewer 2: Yes.

Interviewee: Alright. First, I want to express my appreciation. I enjoy everything about the environment, history, and fauna in the area. I had a friend who was a biologist and worked with me in the medicine lab. He was an excellent scholar, and I brought him along to conduct a survey of all the fauna here. The number of bugs is extraordinary; you cannot imagine it. Here, the Santa Luca River meets the Santa Luca Chico. There are rabbits, deer, and a bit of everything, which I think is also important in terms of fauna and birds.

Interviewer 2: Do you have any concerns about the area being offered in that sense? Any problems that you have

Interviewee: Well, for example, the fox transmits a Neospora disease that causes abortions in cows, so it would be good not to have foxes, but unfortunately, they exist.

Interviewer 2: Do you see any relationship between having a buffer zone and that kind of feedback?

Interviewee: Culturally, I am very aware of it because, when I was a child, I liked to go fishing and bathe in the river. I see it as something I like; that is, I appreciate the fact that there is a productive agricultural part and also that there is a kind of jungle. And the children like it, you see? My daughters enjoy it. So, I also enjoy the countryside. For example, at a certain point, Conaprole (a dairy company) had launched a programme called "Tampo Seguro," which was an excellent milk quality programme, but unfortunately, it was discontinued. One of the programme's goals was to preserve areas where some biodiversity was maintained.

Interviewer 2: Conaprole could be an implementer of change, couldn't it? How do you see something like this being implemented, if not by the Ministry of Environment?

Interviewee: Look, I love Conaprole. My grandfather was a founding member of Conaprole, and I think it's a good thing. Unfortunately, it is often managed by politicians or managers who lack vision or are in their comfort zone and don't go beyond their noses. But for me, a marketing policy that Conaprole should have is to say, "Well, we produce dairy, but we also take care of the environment." So what happens? When the dairy was first produced, it was to supply Montevideo, so it was for Uruguay. But now it is a high-level agro-supplier. So we have to make sure that we don't go too far and that what happened in Holland doesn't happen again, where family farms became industries and then had a negative boomerang effect. Now, for example, there is much sensitivity to the issue of water because of all the restrictions that Conaprole has imposed on Montevideo. For me, it should have a proactive approach to put more teeth into it. Conaprole has done many things, and their actions must not be negative. It has done things, but there are times when it seems to me that Conaprole's staff lacks technicians. That is, each area of Uruguay has its own "milk basin", and there are about ten regional agronomists. It seems to me that these regional agronomists could work hand in hand on critical issues with certain producers who do not follow the guidelines. For example, they could ensure that the guidelines are respected. For example, in Friesland Campina, which is a fairly large company in Holland, there is one agronomist named Pablo (Modernel, red), who is also well-known. He's working there; he went to do his doctorate there; he stayed there; and he worked a lot with pastures here. They have a quality indicator here, and they pay you for quality there. Let me show you, for example, that this is a case on dairy farm X. There's a stream that comes down here, and the dairy farm is located here. It's brutal (the interviewee shows a picture of large algae blooms in the surface waters of this property in red). So what happens? Here, we could look for certain things, and I don't see that the agronomists who advise the producer, who is a simple farmer, should give him a hand or guide him on this. A few things could be done to mitigate this, but do you see what it is? It's clear, isn't it? The interview gets disrupted as the interviewee gets called.

Interviewer 1: Perfect; I have everything. Thank you.

Interviewee: That's very kind. Do you work in San Javier?

Interviewer 2: Yes. I'm working in San Javier. I work for an NGO in wildlife monitoring. I'm looking for a bug called Aguara guazu (maned wolf), which is a fox.

Interviewee: Ah, yes, Aguara Guazu.

Interviewer 2: My responsibility is to coordinate the project to find it. The Aguara guazu has appeared several times in Argentina, and here in Uruguay too, it is the long-legged one. I'm looking for that, and I'm working on that too, along with all the wildlife monitoring work that I do.

Interviewee: I have to introduce you to a biologist who is divine, an expert in wildlife who knows everything—the noise of the bugs, the dung, the footprints, everything. She is a specialist in felines. It's amazing how humans and animals can connect. I don't want to bore you. Come, I'll show you this place because I don't have much water here. I like the story of this drinking fountain, which was gradually being fixed up. It was originally here, and he was working on it little by little. And after going to see it several times, it was finally repaired.

Interviewer 2: And how do you handle exotic species on the farm?

Interviewee: For Ligustrum and Gleditsia (*Gleditsia triacanthos*), we cut them and apply herbicides.

Interviewer 2: And what about Parcapien? Can you use herbicides on that?

Interviewee: Actually, I have a lot of Paspalum (Paspalum notatum, red). It's a long bean that has become invasive, and we want to prevent it from spreading. The most complicated thing is the blackberry, and we use Tordon, an herbicide, and try to use a rotavator to maintain the stump. But there's still a lot to do.

Interviewer 2: Do you feel like you're improvising or following recommended management practises?

Interviewee: I think the management I'm doing is reasonable. There may be management books or recommendations, but I believe in pruning the vegetation to allow cattle to graze below and to promote better plant growth. It's all about managing the natural pasture, and it's something that can be seen now with the paintbrush.

Interviewer 1: Thank you for your time. I will end the recording here.

*Interviewer 1 is the student, and interviewer 2 is the supervisor.*

# Interview 14

**Interviewee: Producer            Date: 16-03-2023**

Interviewer 1: Have you heard of the Santa Lucia River Action Plan?

Interviewee: No, I haven't.

Interviewer 1: The Santa Lucia River Action Plan is a policy that aims to improve water quality in the Santa Lucia River. It consists of several measures, such as a water treatment programme and the implementation of buffer zones in the basin. Do you know about riparian buffer zones?

Interviewee: Yes, I understand your question. More or less, I don't have much information on it.

Interviewer 2: What comes to mind when you hear people talk about it?

Interviewee: Well, I think cattle shouldn't be allowed to move within the Santa Lucia Chico river margin, but I'm not sure. In the buffer zone, you can graze without moving the land, right? Word of mouth has oriented us there, but I'm not sure about the exact number of metres. Some say 70, others say 50, and some say 100. It varies depending on the location.

Interviewer 2: Are you a member of a rural group or rural development society?

Interviewee: No, I'm not. But I am part of a group of tamberos (dairy farmers, red) that is being formed here, but I'm not sure if you can find that network. The city also gives counter-traction to the Santa Lucia River... Anyway, what was your question again?

Interviewer 2: Are you alone in that group?

Interviewee: At the beginning of the formation of that group.

Interviewer 2: So far, have you been in another grouping?

Interviewee: No, in my case, it's my first time. I mean, my old man had more experience, but unfortunately, he passed away. Now I'm telling myself that I'm the one, but I'm not seasoned. I'm trying to do what they ask me to do about the issue of tributaries on the dairy farm so that no water goes into the river.

Interviewer 2: And someone from the ministry asked you to do that?

Interviewee: Yes, we are a bit on edge because we are close to the coast. The ministry has come in for environmental projects related to tributaries.

Interviewer 2: Did they help build an effluent treatment plant, or was there any money that came for that?

Interviewee: Yes, they provided that kind of help—70-30, 60-40, or something like that—regarding the percentage of financial support for the total cost of the project. In our case, it has given us a big hand because we built a drainage basin, treated the solids, or did a lot of little things, and nowadays we apply it for treatment. The cleaned liquid drains at the end, and we throw the solids into the farm. This is the first time that the pool has been cleaned; it's been 10 years since it was built, and it's the first time that we've cleaned it to zero again. There it goes. Yes, they applied it in the field. This is just a test to see how far the ministry will go, and they're doing it again. We all have some experience here, and I'm partly seasoned but also partly inexperienced.

Interviewer 2: What are you addressing?

Interviewee: The buffer zones.

Interviewer 2: And what kind of business is this? How many cows do you have, and how big is the enterprise?

Interviewee: We have around 120 cows in milking, and there are three farms here on 100 hectares.

Interviewer 2: Are they private or some kind of organisation?

Interviewee: They are private—from my uncles and aunts.

Interviewer 1: Do you have buffer zones on your land?

Interviewee: There are about 18 hectares of native woodland, which we don't touch more than with the cattle. It's not without cattle grazing at all, but they don't move or anything like that.

Interviewer 1: Do they (the buffer zones, in red) generate any problems for you?

Interviewee: What causes us problems is the flooding of the river. The water comes up to there, and then we have to be careful because it is a low area.

Interviewer 2: And how often does the river flood?

Interviewee: Well, it's been about three years now, and it hasn't risen. But there have been three years of drought as well. However, before those three years, there were two big floods that reached us, leaving 85–90% of the countryside underwater. But we don't know what's going to happen with the railway track now. The track used to follow the river's path, but now they've put it through an embankment, and we don't know what's going to happen because of the dam. We already saw the effect of the embankment on Route 11. It's a terrible embankment, and it could be a problem if the water rises. Let's hope not. As for what happens further downstream, let's hope the engineers and hydraulic experts have thought about it. If there is a problem, you can make a complaint to whoever is responsible. It's not just me who's scared, but until it floods, it's just a worry. On the other hand, it can also be helpful.

Interviewer 2: Now, about the buffer zones that you can't touch: does it create a problem for you? Can you not plant any trees or something else?

Interviewee: I have no problem, but it's limiting because it says maybe I could put them there. If they (the buffer zones) stayed there, I could take advantage of them, even if they were stationary, without movement, or anything. It limits the field, that's all. You could say I could take advantage of them with pasture, excuse me, with the cows being there. I mean, taking advantage of them in that sense, without limitations, so that today or tomorrow the cattle can't enter or not.

Interviewer 2: How is it today?

Interviewee: There is a forest area where I have cattle, and they can enter there. No, we have a fence called a "coast guard" in front of us, so they can't enter the water. That's where it is. For two reasons: it's very dense and dirty, and sometimes cattle can get lost, so we can't find them, and they can't get into the water. And then there's a pond, and the river flows to provide water.

Interviewer 2: How do the cattle take water?

Interviewee: There are reservoirs in the field. Yes, we use them in cases of long dry spells like now. We bring water to them from a tank that has a pump. That's where it goes, and it's distributed in the field. I extract 3000 litres and put it in the tanks, but they don't have access to the river. That's how it is. I can't give them access because sometimes I find them and sometimes I don't. That's the problem.

Interviewer 2: So, how many metres is that fence from the river? Do you have an idea? From the riverbed, it's about 60–70 metres.

Interviewee: That's where it is (interviewee shows the location of the fence on Google Earth in red). From the water, in other words, it would be within the metres required by the regulations, theoretically.

Interviewer 2: The wired fence is located in what belongs to the state, i.e., in the riverbed. As far as I understand, it belongs to the river, right?

Interviewee: Yes, that's correct. In theory, if you weren't complying with regulations, they would have come to ask. The regulations would surely have been complied with, because if not, the ministry could not have allowed it. They would have come to tell you, "Ah, you are not complying; how do we go about regulating?" We suppose so, because according to the agronomist engineer that we have, she is in charge of this, and we are 1,700 metres from the water, right? That's approximately 1,800 metres from the river water intake in Santa Lucia.

Interviewer 1: What purposes do you think you currently have for the buffer zones in the Santa Lucia estuary for you or your company? If you could change them, how would you improve them? I don’t know. I am unsure if, with the law today or tomorrow, that area can't be grazed in. In our case, it would kill us because it would be almost all those 18 hectares that we couldn’t use. In other words, You can't touch the forest. That is to say, today it has the purpose that the cattle enter at least up to there, up to the fence.

Interviewer 2: And is it among the trees?

Interviewee: Yes, the bush is still relatively clean. It wasn't invaded by Zarzamora (Blackberry, red), which is what kills the most here. And at the same time, the cattle had no access to the riverbed. There are some invasive species that you can identify, such as the Zarzamora (Blackberry) and the Ligustro (*Ligustrum lucidum*). The Lugustro moves forward in the field and expands. It is considered invasive here, but it doesn't bother us in the riverbed. We don't do any kind of management with the ligustrum or anything else. What I fight against a little bit is the Zarzamora that is coming up in the field. I don't touch what is in the riverbed because it doesn't bother me. We are dealing with the spots where the Zarzamora appears, which then start to close and eat up the field. It becomes impenetrable. So far, we have kept it at bay. There are 100-hectare fields, but only 25 are usable. The rest is a whole forest. Zarzamora and other invasive species such as Espinillo *(Vachellia caven*, red) are taking over. Even the Zarzamora is drowning out the Espinillo now. They are like curtains. The man leasing the land from me uses it for dry cattle, but there are parts that the cattle don't use. I am paying rent for 100 hectares, but I only use about 20–30 hectares. It's impressive how overgrown it is, and we are far from the river. Even from the fence, you can see that it's super overgrown in the forest. The solution I see is to make a piece of field in the future. It's a long way off, but we have to transform the land by tying up the Espinillo and clearing things little by little. I would have to do it myself because the one I’m renting from is not very interested. I'm still paying for the hectare of land, but I'm only taking advantage of 20–25 hectares.

Interviewer 2: Are you talking about making a pasture for the cows to have access to? Because it's about a kilometre away and they can't get there.

Interviewee: We could make a pasture with fences or something that doesn't require much maintenance. It's a shame that there's so much land that could be used, right? Besides, the area hasn't been tended to and is becoming overgrown. But now there are also invasive species, such as the wild boar. They have taken shelter there, and because of this, it's becoming a problem. If we don't tend to that area, we could create problems not just on our property but also on others’ since the bush is much thicker there. The pigs bring in blackberries, hawthorn, and other things. Mostly it's the pigs, though. They hide there and attack cows that are giving birth. We've had a lot of problems with that in the field next door.

Interviewer 2: Have you ever had to kill a cow or a pig?

Interviewee: The pigs are carnivorous and will eat cows that are in labour. We sometimes let our pigs roam around so that they can eat some plants or whatever, but they end up destroying everything. They'll eat the corn and the cow's feed. You can't see them along the perimeter, but they're in the middle of the field, chewing away.

Interviewer 2: How do you fight them? Do you have any hunting friends or anything?

Interviewee: I don't fight them, but sometimes I get mad and shoot at them.

Interviewer 1: And what characteristics or physical aspects of the buffer zones serve these purposes? For you and for society?

Interviewer 2: You told us that the riverbank has the purpose of giving you fodder for cattle to graze on. And for society, do you think it has some purpose or some function that it fulfils?

Interviewee: I don't think so. It even favours those who are in the field. I don't know; I think it favours us in terms of grazing without ploughing.

Interviewer 2: And what purposes are created for society? And, for example, if you had to describe that buffer zone along the river, what are its characteristics?

Interviewee: There are hills. And there is some Ligustro (*Ligustrum lucidum*). That's where the ash tree is invading.

Interviewer 1: If you were to change anything about that area, what would you change about it? Interviewee: You can do tillage. And try to keep it as clean as possible. There's not much else.

Interviewer 2: And to keep it clean, you leave an opening for livestock to pass through?

Interviewee: No, I mean I'm saying it's the Zarzamora. Try not to let it invade any more than it has. Well, the trees, lift them up a bit so that the cattle can use their shadow, because it also helps, doesn't it?

Interviewer 2: And what benefits would it have for you to take the Zarzamora out, or to be able to control it?

Interviewee: It means that it doesn't kill the bush. Because the Zarzamora, you see, kills the tree, and if it doesn't, it doesn't make it grass down there. Let's say you are standing half a metre from the edge of the field. Everything beyond that point is soil, and it would be beneficial if you could clear the area and turn it into pasture. However, the problem is that the vegetation is so dense that it doesn't just let you in; it chokes the forest and overruns the trees. If you were not allowed to manage a larger area, you would have difficulties with the reproduction of certain plants. The immediate area around the buffer is already being used for grazing, but there are small hawthorn trees that they are trying to preserve. There is a riverbed about 300 metres away, and the area closer to it is filled with dense vegetation that is not suitable for livestock. One of my neighbours is renting a field that has been invaded by unwanted plants, leaving only small patches of usable land for livestock.

Interviewer 2: He suggests that it would be beneficial to utilise more of the land in their field to produce fodder. He acknowledges that there are drawbacks to this plan and is unsure of what changes would be necessary to improve it. He believes that leaving a margin of forest to prevent pollution from the fields is important, but he understands that there are farmers who need to utilise the land near the shore. He wishes that he could clean up a portion of the land in their field to make room for more fields for production. He also prefers a natural landscape.

Interviewee: Let's say half a metre from the circumference, it's soil. And it would be advantageous to be able to let it become something more usable. Invasive species don’t just invade; they choke the forest. It overruns the trees. In other words, if you were forbidden to manage a larger area, you would have problems because of the reproduction of Zarzamora, for example. I don't know; maybe I'm talking nonsense about dismantling or clearing up the bush so that you can plant other species there. Maybe I can show you the map so we can look at it together (the interviewee proceeds to show his land on Google Earth in red). This is my field, basically. Here is the street, and here we are coming this way. That this is already inaccessible here. And well, here we are taking advantage of it with grazing because the issue is that there are big trees, but there are also small hawthorn trees, which we are trying to insulate. I'm telling you, on this side, which is the closest one, this must be 300 metres from the riverbed. And, well, here we have a lot more metres than with invasives. You see, these are the 24 hectares that we've been able to use, and this is all the other land.

Interviewer 2: This is the riverbed, isn't it?

Interviewee: Yes, but it is impenetrable. The only thing I've been able to use for livestock are these patches. It's clear. There you go; this is clear. And here... And here in the middle, there's no... It's a crime; I mean, it's a shame. That's all Zarzamora, Ligustrum, and Espinillo. Unbelievable. I feel like it's a loss, but it's drying up. The land is low and flat, like a sandy soil that filters water well. Unfortunately, the meadows are gone, so there's no grass for the animals. I'll have to trick them by putting them on the land until I can regenerate the grass, but I've run out of area to plant more.

Interviewer 2: And do you think that the effects of climatic changes—droughts, for example—on the buffer zones would be different if the changes that you are implementing were different?

Interviewee: I believe that the enclosed areas will suffer more since the sun is strong and the soil dries out faster. Removing the blackberry plant could help since it takes up space and prevents grass growth. However, removing it is difficult since it reproduces quickly. Herbicides might be necessary to prevent it from coming back stronger. It seems to me that by removing this invasive plant, in some way, as it takes hold more on its own, to see more grass, the ground will be more covered. I mean, nowadays, you don't have any tools; you just put in more money and hope for the best. The thing is, in this part, you could still do it with a backpack or something because it's still in the forest. There are acres and acres that you can't enter. And the vines are so big that they are as thick as a finger. You see, it's not an insignificant little thing. I don't know if it's not worse because it invades, climbs, and even has thorns. I was on one of my neighbours' land, and I had an acquaintance who was opening a path, and he was crazy. He put on a shirt, a shin jacket, and a knife, and he got in with that. He was already saying that he couldn't; he would get hooked on everything, and the shin jacket was the only way to get in. And that's why I say that it's a great shelter for pigs, because if you don't go in, no dogs, no cattle, nothing goes in. They just get in there.

Interviewer 1: And how do the invasive species affect your work?

Interviewee: Just because they can't fight it, it's a wasted field. If you can't fight it with a tool or whatever, it's a lost field. It's strange that you can't make it productive.

Interviewer 2: And if you had to ask for something, if you had to say, "What do I need? What would be good for this?" Whether it's from government support or whatever, what do you think would help you?

Interviewee: Permission to enter the buffer zone So that you can move forward a little bit. I mean, a little bit and not be fined, you know. I mean, I understand that you have to leave the water, but a little bit to fields that are very close to the street, right? To combat the invasive species.

Interviewer 2: And do you think that will be easy?

Interviewee: No, I don't imagine that. I don't know much about the regulations either, and I don't think so. Can you ask to move it forward? Because it's an invasive species. And in that case, if I gave you permission, you would move it forward, take it out, and put me in the field that you are renting. You would enter it, right?

Interviewer 2: And what do you pay for the rent of this land?

Interviewee: To put it in simple terms, I pay about 15,000 pesos a month for 10 fields. This would help me grow my farm a little, as I have 120 cows that are currently confined. I mean, I have a total of 120 cows, not just the milking ones. Of course, some are insured, and some are for milking. It's very cramped, and we can't make better use of the natural fields.

Interviewer 1: How do you think the new zones will affect the species?

Interviewee: There won't be a buffer zone, so invasive species like blackberries and hogs may be affected. The government doesn't allow you to manage certain strips of land, so this might have an impact on the reproduction of pigs or other animals.

Interviewer 2: Regarding the Santa Lucia action plan, did you have any relationship with the implementation of the plan or the basin commissions?

Interviewee: I was involved in that part, and I remember someone from the ministry coming to see us about the affluent treatment plan that came in because of an instruction from a trade union. The National Association of Milk Producers and the Association of Dairy Farmers.

Interviewer 2: Did the union come with an affluent management plan?

Interviewee: It is through these unions that the ministry arrives more quickly or gives priority to the establishments we are working on. They kind of facilitate the arrival of the ministry, let's say, through the two unions that I'm talking about.

Interviewer 2: And they only talked about affluent management; they never told you about buffer zones; you only found out about that from your neighbour?

Interviewee: Yes, it was more or less discussed here; more than anything else, they discussed what is not possible. The trade union is always the one that advises you in that sense. If a project comes out, we are going to say from the ministry that the ministry is already going to the union first so that the union can disseminate it. That's where it goes—to the producer.

Interviewer 2: Are you OK with that?

Interviewee: I'm fine. I'm fine.

Interviewer 1: There are no more questions to ask you, so thank you very much for your time.

Interviewee: In my case, I thank you for listening to us and for talking about it, because it's a good idea that at least I listen to the state, but that the state also listens to the small producer, more than anything else, who has limitations in the field, you know.

Interviewer 2: And in general, as an enterprise, what things would you need to improve, or what do you think you need more field area for?

Interviewee: In my case, at least I would need to be able to exploit a little bit more of what is in the forest.

Interviewer 2: And if there are no invasive species, is that more like the native riparian forest that is in the riverbed?

Interviewee: If you were to clean that area and do livestock farming, for example, it could be useful instead of just being a pasture. My fear is that the pasture will become overgrown with thornbushes, as there is little or no access to the forest. Over the years, the situation has been getting worse because we are all here but don't dare touch it. There was a rumour that we couldn't even use a rotavator, so we couldn't use it on the grasslands or keep it clean with weedkillers. However, I can tell you that these are just rumours. I have seen my old man, who has been here for 40 years, control the rotavator, or at least what is already here, but not leave it. Out of those 100 hectares, I am renting a hectare of natural field for around 70 dollars, or less depending on the number I have.

Interviewer 2: Would you pay for a good field or pay 100 and say that 25 is enough?

Interviewee: I won't lie to you; I am currently paying the owner of the field 30,000 pesos for those 100 hectares. I haven't calculated it for years, so I don't know how much I have left in the hectare more generally. I don't have any kind of problem, though, so we can work it out together. I don't know what the costs could be, but not for the field itself.

Interviewer 2: Unfortunately, I don't have a calculator with me, but we have 30,000 pesos per year over 40 pesos to the dollar, which is 750 dollars per hectare per year for the 100 hectares, right? But you have to pay 30,000 pesos per month. I'm sorry, I was thinking of annuals. Forgive me; I said it wrong. 30,000 is 9,000 dollars per year for the 100 hectares, which is around 90 dollars per hectare. So, yes, you are paying for a hectare that is a little more than average. Of course, let me clarify that if you were among the 25 who took advantage of this offer, you would have paid $60, right? That's an interesting price, don't you think?

Interviewee: Honestly, I can't tell you off the top of my head how much money I'm making, and I've never actually done the calculations. However, in my case, this offer is limiting because I can't expand my business since there aren't any farmers coming to the area. On the other hand, I have a contract, but I haven't been able to come to an agreement in 10 years.

Interviewer 2: This is also limiting for you, right?

Interviewee: I mean, unfortunately, there are only four of us here, and my neighbour probably told you that we have lost a lot of business on the coast. We don't have the means to expand much, and it's affecting agriculture. I see it as an opportunity to advance agriculture, as they are paying good money for it, but we can't afford to pay as much as $360 or $400 like we did this year.

Interviewer 2: Thank you for sharing your vision and insights. For now, that's all.

Interviewee: If I come across any projects or something interesting, I'll let you know. I'm in contact with someone who will come and do a survey so that we can be more informed. We don't know the exact area that the buffer zone covers, but we try to respect it by eye. We must be mindful of protecting the environment, especially our water resources. However, some areas have too much forest coverage, which limits their use. But that's a special case.

Interviewer 1: Thank you for your time. As I mentioned earlier, I will stop the recording of the interview.

*Interviewer 1 is the student, and interviewer 2 is the supervisor.*

# Interview 15

**Interviewee: INIA            Date: 16-03-2023**

Interviewer 1: A buffer zone is a strip of vegetation along a watercourse. Its purpose is to capture and retain agricultural nutrients to improve water quality. The interview is about your experiences with buffer zones. So, what do you do? What's your job?

Interviewee: Ah, what do I do? Well, I am a researcher in what is now called the "Environmental Sustainability Programme". It's now called the "Area of Sustainable Development, Production, and Environment". Essentially, I work on biodiversity and ecosystem services, but I also touch on other environmental issues. There is a strong emphasis on biodiversity and ecosystem services, mainly in livestock systems, but I work on almost all systems nowadays. INIA has divided its research into various systems, which are agro-sensitive, agricultural, livestock, dairy, and forestry. I have activities in practically all of them, which makes this environmental area transversal to the whole of INIA. There is a high demand for work on biodiversity issues, and there are no people trained at INIA for that.

Interviewer 1: And what organisation do you represent?

Interviewee: INIA stands for "Instituto Nacional de Investigación Agropecuaria," which is where we are. This is an experimental station called "Las Brujas," one of five throughout the country. Although the emphasis of this experimental station is on intensive vegetable production, horticulture, and fruit production, people like me work all over the country in many production systems. There are several of us who work not only here but also with producers and production systems. We work mainly with producers and real production systems and monitor environmental variables as a result of the system or as a result of management changes. There are no experiments conducted with producers, such as treatment control. I am an agronomist with a PhD from the Polytechnic University of Valencia in the environment and animal behaviour department.

Interviewer 1: And what are the responsibilities and objectives of your organisation in the design and management of the buffer zones?

Interviewee: As for the responsibilities and objectives of my organisation in the design and management of buffer zones, in general terms, INIA has become aware of their importance recently. Colleagues have recently developed activities to restore or maintain better buffer zones. But in itself, it is an objective that is institutionally very new and does not have many precedents.

Interviewer 1: And what are INIA's interests with buffer zones?

Interviewee: To minimise the arrival of nutrients in watercourses, which is the main impact of agricultural activities. But personally, I have gone a little bit further, because if you look back, this is a tool that we developed, called the Index of Ecosystem Integrity (the interviewee shows a poster about the index, red.). This part of the formula focuses on riparian zones, where we also consider the status of riparian zones in a broader s, se, including conservation of biodiversity, and protection of particulate matter and nutrient inputs to watercourses. The final result expected is that the aquatic ecosystems are in the best condition.

Interviewer 2: And what part of the area of riparian zones on the property was analysed?

Interviewee: Each paddock is analysed, and if the paddock has a watercourse, that watercourse is analysed, including the riparian strip itself, the 10 metres on each side of the watercourse, and the watercourse itself. This accounts for a quarter of the total value of the Ecosystem Integrity Index, which takes into account vegetation structure, species, soil, and riparian zones.

Interviewer 2: So, what do you define as riparian zones in the index?

Interviewee: It refers to the strip of woodland or the 20 metres that accompany the watercourse. In fact, the index gives more importance to the first 10 metres and a little less weight to the next 10 metres. We also consider the vegetation structure, ground cover, and other factors. In other words, this is in the fundamentals, and it is also explained in the article in Ecological Indicators, which discusses the index and what we measure in the riparian zone area.

Interviewer 2: Do you have it measured? Have you categorised some fields? How many? Up to where? How far do you have this index, or do you have to go and measure it?

Interviewee: We have to go and measure it. All of this is done in the field, where variables are visually assessed without the use of instruments. It was designed for basic training purposes, but it involves evaluating characteristics that have enough scientific information to contribute to the improvement of water quality. These include vegetation, certain structures, more soil cover, the prevention of gully erosion, and many other factors that may not even be directly related.

Interviewer 2: We can also apply economics, which is a useful tool.

Interviewee: The great thing about this approach for economists is that it uses a quantifiable scale from zero to five, which was created specifically for the purpose of comparing the evolution of economic, social, and environmental indicators in a project. By using this scale, we could measure progress economically from one to two while also being able to determine whether environmental progress had improved or declined by one point. In fact, the project showed improvements in productivity and, in several cases, stability in the ecosystem, and even some cases of improvement. Of course, a lot of water has flowed under the bridge since then, from the time it was created until the version we are using today throughout the country, where it has been greatly improved, especially in terms of calculations. But today, we use it almost all the time as the base indicator for everything we analyse, and we can add other things to it as needed.

Interviewer 1: And what is the relationship of your organisation to other stakeholders in the process of designing riparian buffer zones?

Interviewee: We haven't actually worked on buffer zone design. Our work has focused on assessing the state of buffer zones, but we haven't done any actual restoration work yet. I am currently writing a project that will explore alternative approaches to restoring buffer zones, but as far as I know, our institution hasn't done any restoration work yet, except for the efforts of some colleagues who worked on planting native species in agricultural areas in the lowlands to cover the soil and prevent the arrival of nutrients and particles. While this approach may restore some of the functions of the buffer zone, it does not constitute restoration of the original vegetation, as only a single species is planted. There are many varieties of native species that could be used, but so far the institution has not taken any action towards restoration. We have minimal restoration work here, mostly for demonstration purposes, in the riparian forest. We are also working on another project in the protected area, which is no longer a riparian zone. However, my mission or way of working is to evaluate the state of integrity of riparian zones mainly through this tool. We have data from many establishments in various parts of the country. For the Ecosystem Integrity Index, we are working with producer unions, the Ministry of Livestock, the Ministry of Environment, the University of the Republic, some NGOs, some civil organisations, the Instituto Plan Agropecuario, the SUL, and the Uruguayan Secretary of LANA. Everything is related to the environment. I mean, I don't teach them, but if there is an environmental relationship, it has to look for relationships with people. Through this, they begin to introduce concepts about the state of the riparian areas. Some people may wonder what this is since they don't even know what to look at or what could be considered right or wrong. This doesn't mean that we might not have to revise it in the future and say, "Well, let's see, this thing that we evaluated has to be evaluated differently, or it has more or less weight." But now, at least they know that there is an environmental dimension and that it is not just land. Through the index, it has been incorporated as an evaluation practise.

Interviewer 1: These buffer zones can be designed in many different ways. For example, using different types of ground cover or limiting livestock access What function do you think buffer zones currently have in the Santa Lucia River basin?

Interviewee: The Santa Lucia River basin, in particular, is rather more production-intensive than the rest of the country. Therefore, the functions of buffer zones are the same as everywhere else. But I believe that these natural functions are affected by use. There are no restrictions on livestock entering watercourses, for example, except by decree around the Severino Pass dam and some watercourses of the Santa Lucia River, but that is very recent. The rest of the cattle have free access. And where there are no cattle in the water, the tributaries of the river have either agricultural or dairy activity, which is quite intensive. As far as I know, there are not many measures to protect the functions of the buffer zone, apart from the fact that if, for example, with the law on soils and so on, they require a certain amount of ground cover, they should not be cultivated, and so on. But I think that compliance with this is irregular.

Interviewer 1: And what characteristics or physical aspects in the buffer zones support these functions?

Interviewee: For me, the structure of the vegetation is very important, which is something that is not normally given much importance. The structure is not in terms of the structure of biodiversity as the interrelationships between the components, but in terms of the presence of the important plant strata, low vegetation, medium vegetation, and trees where appropriate, because not all buffer zones here are the same. Some have forests, others don't, and they don't necessarily have to have forests. Today, most of them have grassland. But perhaps in some of them, there should have been a forest, but it was either cut down or, because of repeated grazing or trampling, has disappeared or is of very low quality. For me, the important thing is the structure of the vegetation: good cover and diversity, because if it is only cover, obviously it is going to be an important physical aspect to stop drag particles, but it is not going to allow all the complete biological processes to take place. That requires a diversity of organisms, and that diversity of organisms, whether fauna or flora, depends on the diversity of structure that is present, many species, and different strata of vegetation.

Interviewer 2: And what vegetation structures do you prefer for the buffer zones?

Interviewee: I don't prefer anything; I prefer it to be natural and correspond to that area. If it is a forest, it should be a forest and not something else. And if naturally there were, for example, high grasslands or grasslands, that is the vegetation that is there. But I don't like the design, saying that's what I'm going to plant here and that's what has to be there, but that it is as similar as possible to the original ecosystem, that is, the vegetation that corresponds to each site.

Interviewer 1: And what functions do you think the buffer zones in the Santa Lucia river basin should have?

Interviewee: In the Santa Lucia river basin, the protection functions of the buffer zones are most important, as is the function of retaining nutrients. I know that it is only partial, but the retention of nutrients and particles and the generation of biological corridors for wildlife That function, as it is not directly related to water, is minimised, but for me, it is as important or more important than the protection of water quality.

Interviewer 2: Is it all about diversity?

Interviewee: Yes, that's precisely why I emphasised the issue of diversity and structure. We can plant a buffer zone with Paspalum notatum, which creates a physical barrier and consumes nutrients in the summer. However, we need diversity in plant species so that nutrients are consumed all year round, not just in the summer. This also promotes growth and provides habitat for wildlife, insects, pollinators, and other natural species.

Interviewer 1: What features should be changed or added in the buffer zones to prevent undesired changes?

Interviewee: It's difficult to give a generic answer as it depends on the specific situation. However, the goal is to restore the area to its natural state, which may include measures such as no soil tillage, no application of agrochemical fertilisers, and maintaining natural vegetation. While passive measures may seem desirable, total non-management is not always effective, especially in areas near metropolitan areas. Invasive exotic species can colonise and eliminate native biodiversity. Grazing is one tool that can help control invasive species, but it can also negatively affect riparian zones. Therefore, it's important to consider the specific situation and take appropriate measures to maintain biodiversity. Someone asked why it's important to maintain diversity, especially for a biologist working on biodiversity. As an agronomist, I believe it's important because maintaining diversity helps prevent colonisation by invasive species and promotes the evolution of ecosystems. It may also provide other services that we may not have considered. That's why it's important to consider what you mean by "service." Whether it's just a physical barrier, such as a forest of native or exotic species, or the chemical barrier of nutrient absorption, it's essential to understand the annual activity cycles of these species. If there are only a few species involved, there may be periods of time when they are not active, and therefore, nutrient absorption may be deficient. From the perspective of the third function, which is the conservation of biodiversity and the connectivity of ecosystems, it is vital to maintain diversity. Invasive species often dominate and displace other species, reducing food and refuge sources for wildlife. This can lead to overpopulations of certain species, disrupting the balance of faunal populations. The invasion of the Ligustro (*Ligustrum lucidum*), an Asian tree and the main invasive species in Uruguay, generates a bare undergrowth with no vegetation. This can cause erosion, as there is no soil cover to prevent it. In contrast, the ecosystems surrounding watercourses in Uruguay are normally very diverse, with tree and herbaceous species and an undergrowth with a lot of herbaceous plants. When these areas are invaded with exotics, this diversity is lost, and the integrity of the riparian zone is not always maintained.

Interviewer 1: And can you rate the characteristics of riparian buffer zones regarding what is most important from 1 to 4?

Interviewee: For me, vegetation structure is 4, as it is the most important thing in the structure. Land cover is 3. Perhaps the landscape is another important characteristic. For many people, the soil cover is a 1 or a 2, but for me, it's a 4—it depends on the individual (aesthetics, red). This is important for many older people. I don’t know for younger people, but probably.

Interviewer 2: Do you think the ground cover is a visual aspect?

Interviewee: Yes, it's a visual aspect. For the vegetation cover, it is particularly important. For many people, it's important because they believe it is essential. I'm not sure about younger people, but probably yes.

Interviewer 2: What changes would you suggest? What would be an acceptable passive intervention? What would be an ideal intervention for you?

Interviewee: To implement restoration measures, it's important to acknowledge that going back to the original state is not feasible. Instead, we should focus on litter-free exotic pest control measures. This is one of the main problems within the catchment and is the most significant active measure as it doesn't happen alone. We have evidence that, at least on a human scale, it takes 30–50 years to restore. To me, this seems to be the most important active intervention. Another active intervention could be introducing missing species and giving them the possibility to establish themselves. If there are controls on exotics, these species can occupy the spaces and prevent exotics from taking over again.

Interviewer 1: What obstacles do you think will be faced during the implementation of these changes?

Interviewee: Well, one of the main obstacles is the cost. Controlling invasive exotic species is expensive and tedious, as those of us who have done it here know. Depending on who faces those costs, it can be more or less viable. If it's left up to the owner or private owner of each thing, it's not going to be difficult, except in the case of some people who are already aware of how important it is and make interventions in their establishment because they want to, not because anyone rewards them. A prize (financial incentive, red) would be a good start. For this to happen, it depends on public policies that provide incentives, such as economic incentives or payments for ecosystem services, or on direct action from the state. For example, even if it is in private establishments, the restoration action is carried out by a third party that goes and does things and does not depend on the owner of the land. This is one of the first obstacles for me. Even with the economic obstacle removed, another obstacle can be knowledge. There are not many people trained in these issues, and even fewer with experience. But I believe that this is a less relevant obstacle if the resources to implement these measures are available. There is another obstacle, for example, which could be the partial closure of watercourses. Not a permanent closure, but one that allows entry only when it is deemed convenient. However, this limitation on entry, like the one that exists today, also has a cost. This infrastructure limits the entry of livestock. There may be cases where it is people who cause deterioration due to public use, but they are much smaller in scale. Livestock, on the other hand, is omnipresent everywhere and causes deterioration of the buffer zones around watercourses due to their unrestricted access. Installing physical barriers such as fencing would require significant investment, and in areas where livestock drink water from the watercourse, additional investment in drinking facilities would be necessary. However, even with the installation of drinking troughs, animals may not use them, and this measure may not be enough to prevent access. Therefore, a combination of both measures would be easier to manage. Regarding the access of livestock to the area, some people argue that it helps to remove nutrients that build up due to the lack of herbivorous fauna. However, we are investigating this issue to determine when and at what time vegetative build-up occurred in our ecosystems. Domestic animals can replace herbivorous fauna, and the advantage of managing them is that we can control when to enter, when to remove biomass, and when not to enter. However, if animals are left permanently in an area, they do not remove anything, and the nutrient build-up becomes more challenging to address. Therefore, removing nutrients may require mechanical harvesting. Another alternative I have often suggested to my colleagues, theoretically, is to have a double buffer zone. One is an area where the natural vegetation tries to be maintained and is not touched mechanically, and where there are no large extractions. The other is a nutrient-trap crop, which is like a strip that borders the natural vegetation and acts as a nutrient trap. This crop can be harvested, but it must be taken out to remove as much biomass as possible, which traps all the nutrients that come through runoff. That would be ideal. It also has another cost, but it can be used to provide fertiliser. By recovering everything that was lost upland, it helps in revaluing the fertiliser. However, in the strip itself, you have to see. I think zero grazing is not good from the perspective of vegetation because it evolves with grazing. If there is zero grazing, a lot of dry matter accumulates, which sometimes increases the risk of fire, and it generates conditions of competition that favour some species over others. So, biomass removal by grazing should be there. As I said, there is no fauna that has been able to do it or is in a minimal state; the alternative is that it should not be freely accessible and that it should be managed. However, we don't know enough to say which is the best time. There are empirical experiences, but we are trying to document and measure them. So far, we have used a trial-and-error routine. It's not that there is a recipe for the best function. For system management, it's better to adapt the tool to each phase and environmental conditions. It's important to activate that next. If you don't document and measure things, you won't know if there are no possibilities. You're managing an ecosystem, which is a dynamic thing, and today, after a tremendous drought, may be the correct measure. If it had been a rainy year, maybe the right measure would have been another one. Not the one we have to apply for today. The important thing is to do and document, and the key is to be able to manage. If you don't have access restrictions, you can't manage grazing.

Interviewer 2: Does the grazing come in when it wants to come in?

Interviewee: Well, the amount will depend on the rest of the system's management.

Interviewer 1: What possible solutions do you think there are for these obstacles?

Interviewee: The solution is to have resources to cover the costs of restoration or management of the buffer zone. Generating knowledge is also an investment, perhaps a smaller one, but it allows us to provide recommendations afterwards, not strict rules but guidelines. In these cases, and not only in these cases, we need to have some kind of payment for environmental services, some kind of scheme to make it viable. Because it has costs, and if it's left to the owner, nothing will happen. The owner is not directly affected, or at least not aware, of the state of that buffer zone. If that does not happen and there is no market price, the incentive does not come from public policy but from the fact that the products they produce are worth more because they are in better conditions. But precisely the production systems that are in good condition today, in my opinion, are not those that are in the Santa Lucia River basin. There are mainly dairy and agricultural land uses in the buffer zones. As ganadero’s (cowboys; farmers, red) they are based on the natural countryside, which has other possibilities. In fact, they are already doing it with generative livestock, especially sheep. Now, the producers there are being paid, and I have been implementing it recently. But that could be taken to a much larger sector of the place, which is meat, which is even more important. But here in the Santa Lucia River basin, it has some livestock, but mainly dairy and agriculture, and some horticulture. The incentives on the market side are more challenging. It has to come from public policy and resources to support that. A first step in paying for ecosystem services is tax exemption.

Interviewer 2: What do you see as the difference there? In Uruguay, there is no such culture for agriculture. But there is an exemption for the forest, and there is an exemption for the real estate.

Interviewee: What I see is that this weighs very little in the producers' economies, and much less for intensive producers. So it's not really the tax exemption that weighs in. They may have other types of taxes that weigh more heavily on them, but they are not related to the environment's management or the management of their establishment. There has to be something more, like the soil law today, that obliges the declaration of a use plan and everything else that is mandatory, not optional. I know that measures that are more coercive, i.e., more of an obligation than anything else, are not very attractive. But in some cases, there will be no other option than this type of measure. The ideal would also be to have measures such as payment for ecosystem services or some other type of tax exemption. It could be part of things, but it seems to me that economically, it doesn't weigh so much. That is why it is not so successful. Even so, you can register native woodlands. For example, if the area is woodland and, ecologically, that woodland has very poor quality, it is a green stain. It is there, and they exonerate you. However, you end up paying, at least, for an incomplete ecosystem service. That is the same question as the producer who is suffering from this. It is like a repeated bandage that the same person has today to deal with. With the producers in mind for advice, the truth is that I don't know what to do. I don't know how much advice there is on the control of exotic species without herbicides. We did it ourselves for a few years, and we continue to do it, but I would say that it is almost clandestine. We did chemical control and so on, but there is little written. Now, there is some more experience with control, for example, in the estuaries of Farrapos, and some that has been done by private companies, for example, UPM, etc. That is what there is.

Interviewer 2: I don't know how much advice there is from people who advise them because there is a need. But as we have been saying, the native Espinillo has overtaken the fields, and there is a need to control it.

Interviewee: It is mainly Zarzamora (blackberry) and Espinillo (*Vachellia caven*). All the Espinillo was taken out. The issue is that when they cut it, hundreds of trees come out, and it becomes unmanageable. The solution we are doing here in an area of Espinillo is not cutting, just the opposite; what they do manage under the Espinillos is that very productive fields are generated. Look, just next week, I have a meeting for this issue. For three reasons, it is a leguminous plant that has a lax shade canopy that allows light to pass through. So, the grass grows underneath without problems, but it protects against winter frosts and desiccation in the summer. So, you always look under the Espinillos; the grass is better there than elsewhere. So, in reality, people are bothered by the Espinillo because the Espinillo bothers them. It is a cultural issue. It is not productive. As long as you put a number on it, it is productive. The problem is also that if it bothers them, they start to generate problems. If it begins to be a productive problem, it is generated as a pincushion of low bushes and all things thorny, not a tree as it would be in its natural environment. That does not prevent access below, but such is the case with other invasive species. For example, Ulex europaeus is a tree, not a shrub. It is a plant that invades natural fields; it is especially thorny, and if they burn it, it activates the germination of seeds. Almost all the measures worsen the situation, and here there are many trees. Both American and European ash trees.

Interviewer 1: Another question: How do you experience climate change in the Santa Lucia River basin? Think of a change in temperature or precipitation, droughts, or floods that you have noticed over the years.

Interviewee: Yes, I think that the main thing that I have noticed, and now we are going through a situation, is the irregularity of rainfall and moments of drought, but above all, drastic droughts are what is most noticeable as an impact. The issue of temperatures can be either natural variation or something called climate change. Climate change implies other things, but I think that what one can perceive is just what the specialists say is going to happen in the coming years. What the specialists say is going to happen is irregularity, more high temperatures, more low temperatures, more drought conditions, more water building, and more torrential rains. I think that in recent years, this has not been exclusive to the Saint Lucia river basin but to the entirety of Uruguay, but this can be seen, and we could attribute it to climate change. What is real, for example, statistically, is that in the last 100 years, rainfall has increased. However, if we look at the statistics of the last 3 years, we can see that rainfall has been well below the historical average. When one considers this and looks at the near future, it is hard to believe that the tendency is to increase. But that is what the longer-term statistics and models predict for climate change. In reality, in the near future, the precipitation will be more dramatic, especially in basins like this one with a very important use of water. Although excess rainfall is probably much more problematic for the issue of nutrient and particle retention,  the more it rains, the greater the possibility of erosion and runoff. But these droughts, for example, are going to leave the vegetation with very little protection. Everything will be left with very little vegetation, and any rain that comes later will find these buffer areas totally unprotected or in poor condition. Then, there will be a peak in erosion and particle entrainment. In this case, perhaps because it is winter, it is not so noticeable. But on other occasions, this situation has happened in the spring, and then comes the summer. The temperature rises, the nutrient load increases, everything is full of cyanobacteria, and the drought normally occurs. It is not that it rains less in the summer, but when there is more demand, that is when the deficit is noticed. This winter, it did not rain here either. And this hasn't happened for a long time, and of course, it's not noticeable in winter because the water demand and evapotranspiration are low. But now, as the evapotranspiration is high, we are paying for the winter deficit. The watercourses did not fill, and the aquifers did not flow.

Interviewer 1: And how will these developments affect the functioning of the buffer zones?

Interviewee: Although our flora has been evolving for millennia with variable conditions, we have records, but perhaps not as many. And this can affect the vegetation in these areas and generate moments of knotty soil with very little vegetation cover, which is a negative effect that is likely to be the main negative consequence.

Interviewer 1: How do invasive species affect the buffer zones?

Interviewee: Well, I think we talked about it earlier, if you review the recording, but I repeat, the loss of diversity is the main effect. They become dominant and become pure communities of one or a few species, and diversity is lost. That is the main effect.

*Interviewer 1 is the student, and interviewer 2 is the supervisor.*

# Interview 16

**Interviewee: Producer            Date: 16-03-2023**

Interviewer 1: What do you do? And are you involved with the Santa Lucia River Basin Committee?

Interviewee: Yes, I have also participated in some instances during the early stages of the Santa Lucia River Basin Commission. I am familiar with the areas along the river. They had raised concerns about extending the buffer zones to the secondary and tertiary tributaries of the watercourses, which cover almost all of Uruguay in streams and ravines. This extension could have a negative impact in certain areas. There was talk of having 150-metre buffer zones on either side of the tertiary tributaries, which would leave practically half of the area unusable from a productive perspective. As for my job, I am a producer, horticulturalist, and fruit grower. I cultivate carrots and tomatoes, among other things. The apples and tomatoes did well, except for the second phase. I work alone and then give the plants away because I run out of water.

Interviewer 1: And do you have buffer zones on your property?

Interviewee: Yes. If the buffer zones are extended to the tertiary courses, they will affect my fields, as a whole stream runs through them.

Interviewer 1: And do they generate any problems for you?

Interviewee: These buffer zones can be problematic if they prevent agricultural work in the area. Therefore, it is more of a tragedy than a problem.

Interviewer 1: And what is your role in the management of the buffer zones?

Interviewee: While I don't have an assigned role in buffer zone management, we are all aware of the problem and try to take care of the area as much as possible. We don't work up to the watercourses since they are very dynamic and can be one and a half metres above the water level and 100 metres wide in winter because of two converging streams.

Interviewer 1: There are many different ways to design buffer zones, such as using different types of land cover and management strategies. The next question will focus on how to describe buffer zones.

Interviewee: Sure, based on the characteristics of the areas that I am mentioning to you, they are areas where there is special vegetation with a lot of grass throughout the year, and that is what protects them. As family producers, we try not to get too close to the watercourses because every particle of land that we lose in a flood feels like a piece of us being torn away. So we try to be careful. We haven't planted trees in the buffer areas, but we do try to maintain the natural vegetation.

Interviewer 1: And what functions do you think the buffer areas currently serve for you and your business?

Interviewee: Well, without a doubt, we take care of the watercourses, and from a productive standpoint, we work to the extent that we don't respect those 150 metres that have been established. Yes, I thought that it wasn't regulated. It's not regulated, of course, but it's what should be regulated, and we try not to touch the area affected by the peak rainfall floods. In many cases, there is no productive activity in these areas due to the characteristics of the terrain, which can generate annoyance on the land.

Interviewer 2: Usually, your lands are very good, and that's where we have the excavations to take out water, and all that is done precisely in that area because that's where the water sources are, right?

Interviewee: Right.

Interviewer 2: And, for example, the cattle don't have access to that area, right?

Interviewee: No, because for much of the year, when it's very wet, we're talking about fractions of 8 hectares, so we couldn't do much. But since the soil is very soft, the cattle end up causing much worse damage than if I were to use the land. So we try to keep it as clean as possible without too many interventions, precisely because they are small units.

Interviewer 1: And what physical aspects of the buffer zones support these functions?

Interviewee: Well, there are large grasslands all year. They also serve as a type of wildlife reserve. They are important grasslands, not just for grazing but also for generating the typical humidity of the area.

Interviewer 2: Are they like bogs or wetlands?

Interviewee: There were bogs, but of course, wetlands are the best. And the buffer zone, in some way, captures water reserves, which later, when we extract coal, seep out. It is like a huge sponge. So, it also serves certain types of water services, right? For example, it can be a water reserve, right? And, for example, it affects how fast the water flows away, right? It would be worse if there were nothing, because the water damages the soil when it accelerates at too much speed. So, that mass of grass is also what slows it down; its growth more or less controls the runoff in some way, right?

Interviewer 2: For you, would it be a problem if it slowed down or not?

Interviewee: It hasn't been a problem.

Interviewer 1: Because it could be that the buffer zone grows and does something to you.

Interviewee: No, because we are also careful to work with the drainage of the land plots, and in that area, we have been doing a lot for a long time. The water reaches there, and we don't touch the buffer zones. But no, it's a characteristic of the buffer zone; you have to live with it.

Interviewer 1: What obstacles do you have with the implementation of buffer zones by your company?

Interviewee: Well, the number one obstacle would be the definition of the distance that the buffer zone itself is going to have, right? From the watercourse, how many metres would it be? Ignoring that, there is no problem because we have already been managing it; it is part of the land. Of course, in a unit of 8 hectares or having 2 hectares of wetland, you have a significant percentage of land that will produce for us, but we have water, so... On the one hand, it can be a problem; on the other, not so much. It ceases to be a problem and is a blessing, especially this year. The blessing of this year is that in the drought, the buffer provides me with more water. I mean, I am more than saved; I made a new reservoir, and what I could save, I saved, thanks to that plus. So, when you don't have water, you surely have it.

Interviewer 1: And what do you think the current functions of the riparian buffer zones are in the Santa Lucia River basin?

Interviewee: Well, I think we are talking about the only source of potable water supply for more than half of the country, including the city and metropolitan area, and it will be very important. However, I am also interested in pointing out that when talking about the sources of pollution in the Santa Luca River, production has been systematically attacked. In the case of Canelones, which has more than 25 populated centres, including industries, I don't hear about their pollution problems. I think it pollutes a lot. You have to see the streams that come out of the rocks in this city of 100,000 inhabitants. You have nylon bags stuck in the riverbeds for 5 metres. So, it seems to me that the big villain of the story is not the productive areas. Especially in recent years, there has been a lot of progress in the biological protection of crops, where the amount of insecticides has been significantly reduced and the use of herbicides has been much better managed. I know that there are highly polluting activities such as dairy farming, which generates a lot of water throughout the process, but I think we need to broaden our perspective. We are not the worst in history, nor are we saints, and we need to recognise that.

Interviewer 1: Okay, and what physical characteristics or aspects of buffer zones do you associate with supporting these purposes? Let's say what physical characteristics buffer zones have.

Interviewee: Well, it seems that buffer zones are very natural areas without cultivation. So, that implies what we were talking about earlier, such as wetlands, reeds, and everything natural in an uncultivated and undeveloped area. It is quite natural.

Interviewer 1: I have different images for different types of buffer zones. Which one is it like? For example, with or without cows?

Interviewee: Of course, for us, it would be without cows, and it would look more like this photo you have, with vegetation, water, and no trees. And no cows either. There you go.

Interviewer 1: What purposes do you think buffer zones should have for you?

Interviewee: Well, first, the preservation of the environment, taking care of the water resources from the point of view of human consumption, because it is the same for us, and we must absolutely respect them.

Interviewer 2: In other words, you wouldn't change anything about their functions that are currently being fulfilled?

Interviewee: No.

Interviewer 2: You wouldn't say, for example, that I need to produce in that zone?

Interviewee: No, because if you start exploiting the production of buffer zones, you don't look at the characteristics of the area. Now, it would be better if it were a buffer area.

Interviewer 2: How many metres should the buffer zone be?

Interviewee: Well, here, it's 20 metres wide in some parts, and there are others that are hundreds or 50 metres wide, where there are two wetlands that are hidden. It's an area that has almost permanent water for six months of the year, and the watercourse is more important than the wetland itself. But that part is important. There are 8 hectares, and maybe 2 hectares are affected by the wetland. That's the reality of that unit. The field in front of it is not even 2 metres wide; there is no stream passing through it, and there is no problem. But it's dynamic, because when it rains a lot, I tell you, there's one next to it that is 100 metres wide, and with so much water, it has passed over the road where people pass and has caused all sorts of damage. But that's natural; you can't avoid it, control it, or put up the fence that should be there. It works like that.

Interviewer 1: And what other functions do you think the buffer zones should have for you or for society?

Interviewee: Well, one option could be cattle farming for part of the year, but as I said today, that would only be possible for a few months of the year, and that's impractical.

Interviewer 2: What about the rest of the year?

Interviewee: For me, it should be left as it is and not touched.

Interviewer 2: Keep it as it is?

Interviewee: Today they said that the buffer zones cause problems where they are absent because of the condition of the watercourse. Of course, it doesn't cause any inconvenience for me. It's there. It's there in part because of the physical characteristics of the watercourse, and so on. From my point of view, there is nothing to be done in that sense. And I tell you, it's the water source that saves us less-rich people.

Interviewer 1: What are the most important characteristics if you had to rate them on a scale from 1 to 4?

Interviewee: If we consider water (quality, red), 4 would be the best. If we consider the possibility that it contributes water, I would place it in the best place.

Interviewer 2: And the retention of nutrients, for example, or the retention of pollutants?

Interviewee: Yes, also, without a doubt. But it's less important than water, so 3. For me, the issue of water would be more important than anything else.

Interviewer 2: If you had to say intermittent grazing at some points of the year, maybe it's less important, or would you put it on the same level as water?

Interviewee: No, no, no. Water comes first. Also, because of the characteristics of the terrain, the nutritional quality of what it generates is very low. It's not a high-quality pasture that you can say, "Let's take advantage of this." No, because the contribution it can make is relatively low. You couldn't do cattle farming there if you don't have a part of the area also dedicated to life."

Interviewer 1: And what do you think would be the benefits of these changes to the buffer areas for you or for society?

Interviewee: Well, if there were interventions, I don't think they would contribute more than they do now, right? It's the environmental point of view and what I insist on with water. As long as, and I'm being heavy-handed with this, the buffer area remains specifically the area of high moisture content, If not, it wouldn't be beneficial.

Interviewer 2: And in case it were to be expanded, for example, would there be any conditions for you to say that you would do it, or what would you require to do it?

Interviewee: The thing is, it would take away a large part of the land we are working on because the intervention in the buffer area, according to my interpretation, is to determine rules under which you cannot do a huge amount of things, including moving the earth. Well, in a productive unit that is 300 metres wide, they put 100 metres on each side of the watercourse, and there goes more than half of the farm. It would make it unviable. That is, economically, it would have an absolutely negative impact. And I am talking mainly about tertiary tributaries, right? If you tell me about secondary tributaries, like the Las Brujas stream, well, that's another thing. That's a different story.

Interviewer 2: And if, for example, let's imagine that the government comes and says, "Well, but I'll rent exactly what I'm going to ask for in the buffer zone, would you be able to say, "With this number, we'll do whatever you want, per year, or not?

Interviewee: Sure. There we have to see the number. There, don't believe the government would come and tell me that. I think if we give them the opportunity to propose it, the government wouldn't accept it. And especially this government. It's anonymous, so it could be anyone. Let's see, I don't believe this government in anything. No, I think we should determine more about the use and management of the soil. I totally agree that there would be things you can't do here (in the buffer zones, red.). It's now taken to the extreme that you can't work it. There was a disturbance during the recording.

Interviewer 1: Ah, 25 minutes; ok, maybe we missed 4 minutes or something. Ok, we missed a little bit of recording because the memory filled up.

Interviewee: (The interviewee was talking about the rurality of the area, in red.) It remains there, but it seems to me that there is change that the local authorities cannot stop. But for the local authorities, it is also an important business as the contribution increases, but it also requires maintenance as it is not the same as before. The productive area and the suburban area From my perspective, it may be an exaggeration, but the rurality of this area has changed.

Interviewer 1: Besides the loss of productive land, what other obstacles do you think were experienced with the implementation of the buffer zones?

Interviewee: No, I don't think I see any obstacles in implementing them. If you keep the consistency of respecting the watercourses and the zones, as in the river, up to where the last crescent reaches, this will not cause any inconvenience to the producers. They are the natural conditions that we have always lived with; I don't think there is any kind of inconvenience in these buffer zones.

Interviewer 1: Ok. And for the loss of productive land, what could be a possible solution for this? For example, if we expand the buffer zone, you said that there will be a productive loss for sure. Interviewee: Yes. There is some other problem that would be generated in addition to the productive loss.

Interviewer 2: And if that were to happen, what solutions could you develop? Or what would you ask for?

Interviewee: What alternatives?

Interviewer 2: Right. If I said to you, tomorrow we have to do this because we can't do anything else and because we have no other choice regarding the buffer zone.

Interviewee: Of course. It's very difficult because if I told you, well, this is going to eat you up. If they are going to give you three hectares of land somewhere else, it's no good because today, what do you do with the irrigation structure? You have to start all over again from scratch. It seems to me that we would leave the production. In some cases and in some units, they are all the same reality. There are many different realities. But in some cases, they would have to stop production. Yes, the buffer zone would have to be forested. But there is no simple alternative to that. You take two hectares of land from me here and give me twenty hectares in Colonia, and I say no. Because it is impossible to manage it from a productive point of view. The state could give the land to me, and I could sell it. But from a productive point of view, it is not a solution. Neither the lease nor me For me, it would be necessary to study more, study better, understand better, and see what you can do and what measures can be implemented to keep that area healthy. With the issue of conduits and everything else, you are also touching a productive system that works well. It wouldn't be that way. In other words, there is already a whole investment in the property, which is also affected. Of course, and it's also efficient if you respect the integrity and chain of all those investments that go from working on a road to making a cutwater to laying irrigation pipes. This is associated with the machinery you have, the effort you have to make, and the tools you have bought. It is such an integral concept that if you go into it and think about it, you can deflate it completely. And the family producer, who has no credit, in 105% of the cases you don't have any savings either; to face a crazy idea of, well, I'm going to transform everything That producer will probably leave. Or sell the rest of the land to another larger producer. You take him out of the game that way.

Interviewer 1: And how do you experience climate change in the Santa Lucia river basin?

Interviewee: Complicated, very complicated. Here, as far as we see seasonal change, we have already gone from summer to winter. The issue of drought is nothing new; Uruguay has periods of drought relatively close to each other, but unlike this one, which has been going on for three years now, if there is no water recharge this winter, the coming summer could be chaotic, much more so than this one. We have had a winter with almost no cold, and in horticulture, this does not generate the process that gives the fruit its bud. The truth is that it is quite worrying, and you cannot think about what you are going to do in the future because it is unpredictable. But ‘seasonalisation’ (e.g. changes in the seasonal patterns/cycles) is very clear. And the change in rainfall. We went through an important series last year. On the 15th of January, 400 millimetres of rain fell in 20 days; it was a disaster, then it didn't rain any more. So it's unmanageable; you get ready to irrigate, and the drought kills you. It's very complicated, but climate change is evident. Even though my father died when he was 95 years old and he told me about the drought of 1900, and I don't know how long ago, the frosts of October, he always had episodes of that kind. Of climatic variability and out-of-season events. But not with the frequency, especially not with the harshness. Now you live in a climatic emergency every year for one reason or another. I don’t know what normal is. I don't have much of a record; I don't remember when it was a normal year.

Interviewer 1: And how do you think these changes will affect the buffer zones?

Interviewee: In rainfall excesses in buffer zones, it has much more impact than in water deficits. Then I have a lot of problems. But in buffer zones, if we don't talk about productivity, there is no impact except when it rains too much, which can give me a bit of flooding on the field.

Interviewer 2: Does it not work to transport too much, or why would it change? Because the courses get out of hand and anything can happen there. It doesn't work, basically.

Interviewee: Of course, it doesn't work. But any other kind of impact, I don't foresee.

Interviewer 1: And do you have any experience with invasive species?

Interviewee: Invasive, yes, the parrots have been in recent years. It (invasive species) has had a productive development. Parrots and bugs are some of them. With the drought and all that, the crops in the country practically disappeared due to this, while the animals needed to eat. So there have been major problems. Also, Uruguay has some practical regulations that were used before with Carbodan, a very powerful toxic, which was used to control pests. That was banned. These invasive species have practically no predators, and just like that, the population exploded. And in fruit-growing areas, it has been a very important problem. And in recent years, another species has also been hitting hard. breaking plants and eating fruit. The apple trees have been smashed to pieces in the night. And it has no natural predators either; it's not native. And well, the population has skyrocketed. It has nothing to do with production. But there have been a lot of accidents with the fruit. Because of the ciervo (deer). A girl was killed here on a motorcycle in the early hours of the morning. Nobody hears the ciervo, but the rim of the motorcycle is full of hair. Almost certainly true. Of course, there have been several. It hits hard. But the productive impact, from a productive point of view, is also creating a problem. There you go.

Interviewer 1: And how do they affect your work?

Interviewee: Well, in the case of the parrots and the apples, when the apple starts to take colour, they eat it. There you have the direct loss of reduction. And the ciervo has not left... Well, now it's going to be defined whether it's good or not. But as it continues to grow, we see that every year there is more and more impact from eating fruit and breaking plants. That's becoming routine now. To clean the water. Sure, but they break them. And in the new eucalyptus trees, so much so, without the husk. But it's all about the plant. But that's not a productive issue. Well, it is. Yeah.

Interviewer 1: Do you have a last question?

Interviewer 2: Yeah, I have one last question. How do you think the buffer zones affect these species?

Interviewee: I don't think it has any effect on those species. Of course, the more undeveloped areas there are, the more areas of development there are. But there are a number of abandoned units here that have nothing to do with those buffer zones. So I don't think that has an impact either for or against.

Interviewer 2: So the ciervo also live off the abandoned areas?

Interviewee: Yes. It's one more refuge than they have, but I have a friend who has 4,000 lemon plants. There are 8 hectares planted with lemons. And you go through the headwaters, and the serfs live there. They settle in without any problems. But I wouldn't establish a direct relationship. A buffer zone for birds or plague animals. I wouldn't link to it.

Interviewer 2: I have no more questions.

Interviewer 1: Me neither. I will end the recording.

*Interviewer 1 is the student, and interviewer 2 is the supervisor.*

# Interview 17

**Interviewee: MGAP        Date: 21-03-2023**

Interviewer 1: What do you do? What's your job? And what organisation do you represent?

Interviewee: I work for the Ministry of Livestock, Agriculture, and Fisheries, the Directorate General of Natural Resources. I am a delegate for the Ministry of Livestock, the Santa Lucia River Basin Commission, and some other representations. My background is in agricultural engineering, with a Master's degree in soil science. And our work is all that has to do with the use and management of natural resources, soils, and water. I don't know if you want more specificity.

Interviewer 1: That's fine. The Santa Lucia River Basin Action Plan introduced the use of buffer zones in the Santa Lucia River Basin. The next questions are about your understanding of your organisation's role in the use of buffer zones in the Santa Lucia River Basin. What are your or your organisation's responsibilities and objectives in the design and management of buffer zones?

Interviewee: The Santa Lucia Basin has an approved second-generation unrestricted plan. The Ministry of Livestock, Agriculture, and Fisheries was actively involved in the design of that second-generation buffer plan. I and other colleagues were delegates for the design and monitoring of the plan. We actively participated in the plan, and, from the point of view of the organisation I represent, through the plans for responsible land use and management, the plans for sustainable farming, or the plans for the use and management of soil and water, we watch over the conservation of the resource, in other words, responsible management. For farming and irrigation, we have to design a rotation, a use, and a management of the soil in time and space so that erosion is not greater than what is tolerable for that soil. I don't know if that was clear.

Interviewer 2: I have a question for you. Of these tools that you mentioned—the soil use and management plan and the sustainable farming plan—I don't know if you mentioned any others that I'm missing.

Interviewee: Irrigation.

Interviewer 2: How do you see those tools for working with buffer zones, at least the design and control of buffer zones? Is it feasible to work with a sustainable management plan or a land use and management plan as a downstream tool?

Interviewee: At the level of these procedures regarding land use and rainfed agriculture today, they are done online. It is done by an accredited agronomist for rainfed agriculture. And at the system level, we use geo-services for when a field intercepts a buffer or exclusion zone. For another reason, we also have not only buffer zones but also restrictions. For example, buffer zones, which are actually the safe distance for the application of agrochemicals and are led by the General Directorate of Agricultural Services, or any other area where care must be taken, Well, when monitoring the field, the warning comes out at the system level if certain land is not the designated type. They can be the buffers at the watercourses, which are Measure 8 in the Santa Luca River Action Plan, or in one of the lagoons. I don't remember if it is the one of the Sauce or the one of the Laguna del Cisne; they also have buffers according to the ministerial resolution. But the system warns the user. At the moment, it is not defined as a restriction when submitting a plan, but I don't know if I answered the question.

Interviewer 2: Yes, for me, because many people mention, above all, from technicians who are not from the Ministry of Livestock, that they look favourably on the use, that is, those measures that are already in place that are coercive with the producers, because they can somehow decide what to do and what not to do within the buffer zones. In case these buffer zones are not passive zones but active zones, for example. So that's where the question came from.

Interviewee: Yes, there are tools for possible control over regularisation. Good ones.

Interviewer 1: And what relations do you or your organisation have with other parties involved in this process?

Interviewee: I understand that there are many institutions or organisations that can be affected by the implementation of a buffer zone. The Ministry of Livestock is involved in the whole country; it has one or two, in some cases, offices per department. At the level of producer organisations or even coordination with municipalities and other institutions, there are the Rural Development Committees (farmers unions). At the level of the ministry, it obviously participates actively in the River Basin Commission. We are now participating in the management plan in the Santa Lucia Basin and in other basins, here with DINOT and with the National Directorate of Territorial Development. We participated in the elaboration of the land-use planning decree at the national level, which has defined a buffer zone for several watercourses at the national level.

Interviewer 2: Is it another one of the measures for the Rio Santa Lucia?

Interviewee: Yes, it is a regulation of Law 19.525 that approves the national guidelines for territorial planning and sustainable development at the national level. And there is an article, Article 28, which defines buffer zones. Well, I asked DINOT, the Dirección Nacional de Realamiento Territorial, if the buffer zones are already available to update the services to the level of the second generation of measures, but so far I have not had an answer. But based on Alfred's question, I mean, we have a relationship with all the institutions involved, with DINASEA, which is the National Directorate, which was the former environmental directorate, which is now in the Ministry of Environment, with DINAGUA, and with DINOT.

Interviewer 2: With the producers, what is the relationship, for example?

Interviewee: From the Ministry of Livestock? Yes. And well, it is continuous because the Ministry of Livestock interacts with them through, for example, the General Directorate of Natural Resources. I don't know, now with the agricultural emergency because of the drought, through the General Directorate of Rural Development, I mean, the link is very close for a lot of reasons.

Interviewer 2: Yes, but I wanted to ask you about the channels of communication. In other words, how is the policy, or the measures, communicated? There are many channels for dissemination, communication, and control, aren't there?

Interviewee: For example, the development committees have several channels of communication.

Interviewer 2: Are the rural development committees articulated by the municipalities or are they from the Ministry?

Interviewee: No, it comes from the Ministry of Livestock. It is coordinated by the Departmental Director of the Ministry of Livestock. At the departmental level, there are various levels of coordination, such as the European Departmental Councils, which are led by the Ministry with the participation of the municipalities and various institutions. It depends on each department of the country and has its own variations.

Interviewer 1: Good, very good. These buffer zones can be designed in many different ways. For example, using or promoting different types of land cover and management strategies So there are different ways of defining these buffer zones. The following questions are about how you would define buffer zones. What functions do you think buffers currently have in the Santa Lucia River Basin?

Interviewee: Let's see, in 2013, when the first Basin Action Plan came out, it was an emergency measure. Buffer zones were defined where absolutely nothing could be done. And what I recall was the involvement of the Ministry of Housing, Land, and Environment. Today, control was in the Ministry of the Environment. They had already made an analysis of all the information gathered through various interviews and the standards that were set for this buffer zone. And I would like to highlight one thing here. In other words, the objective of this zone was that nothing should be done and that whatever nutrients might arrive due to erosion should be retained. That was the objective that was set out there. Afterwards, there was work at the level of the Santa Lucia Basin working groups, where there was a lot of exchange, and it became clear that several institutions, including the Ministry, had the idea to not prohibit use but rather to seek adequate management. I would like to highlight something here. Most of our watercourses are bordered by native riverine forests. In other words, in most cases, we have natural protection where there is none. There may be a need for livestock, shade, and shelter in the forest. That is, as part of the functions of the forest ecosystem, there are areas near the watercourse where the forest area has been altered and reduced. In other words, there may be an area of productive use. But I think we have, for example, a defined buffer zone in Santa Luca that is 100 metres from the watercourses of the Paso Severino Lake, San Francisco, and Canelón Grande. We are talking about 100 metres, the area, the property, and the producer. In other words, it is being defined as not using an important percentage of the area of a farm. This makes it difficult to implement the measure. Especially in those cases where there is no native forest. I don't have an update on the status of the situation in those cases or on what control has been taken.

Interviewer 2: And why do you say it is difficult to implement?

Interviewee: No, because if you look at, for example, the Paso Severino dam, there are many producers whose land reaches the edge of the lake, and we have constructions in that 100-metre buffer established by the resolution of the 2013 action plan, areas of farms, pastures, and estates. Which is an important percentage of the area of the farm for the producer, and you're saying that they cannot use it anymore because, in other words, if we follow the strict rules set out in this resolution, nothing can be done. That's why I say it's difficult to implement. In other words, there have to be other tools to implement a measure like this. In other words, this is asking the private sector to sacrifice part of their field and stop using it. I don't know if I'm making myself clear. That is why I understand that it is more important not to prohibit doing anything but to look for a responsible use of those areas and that they really fulfil the function of extracting or retaining nutrients. But if we are going to implement them without economic resources, without expropriation of land from the state, and if we are going to implement them so that the private sector assumes the responsibility for the maintenance of that area, we have to use common sense and see how he can continue to exploit that area so that he can continue to contribute to the integrated management of the property but not exclude it.

Interviewer 2: It is very clear and very interesting, I think.

Interviewee: You have to understand that the native forest is protected by law, and the producer, in addition to the law, if he registers that forest, can have tax benefits such as generation of contribution, primary tax, etcetera. So, in a way, he is not paying taxes for that area, and you can make sustainable use of the forest and even get a logging permit. In other words, the law is already protecting the forest, but it is also giving it tools to really protect it with some economic stimulus, such as tax refunds or tax exemptions. In the case of the buffer zones, no; they were implemented without any incentive tools, for example.

Interviewer 1: Apart from the dimensions of the buffer zone, what physical features or aspects support these functions? I thought that the main function that you mentioned was that of nutrient retention. First, I would like to ask you if you think there are any other functions that the buffer zones fulfil. And then what characteristics support or cause those functions to be fulfilled?

Interviewee: There is a lot of literature on this. I mean, when they were implemented at the watershed level, it was to retain nutrients and, in other cases, other services. For example, in the buffer zones around Paso Severino Lake, there was an initiative on the part of a citizen to plant native trees. In other words, it depends on the use of the producers' properties. What we have to try to do is ensure that in this area, a buffer zone is defined. We at the ministry wanted to articulate that attempt, and then it was put on standby, but that does not mean that it is not something that has been forgotten or that it can't be done. In its definition, the goal is to establish objectives so that the retention of nutrients can reach a point where they begin to become saturated if you don't manage or extract them in some way. Or it would have to be expanded, or some kind of management would have to be allowed. From the ministry, we tried to define management plans for these areas. There you go.

Interviewer 1: And do you have an idea as to the characteristics that sustain the function of nutrient retention? I mean, ground cover or the use.

Interviewee: The thing is that it is quite a broad topic. What I do emphasise is that most of our watercourses have an area of native forest that protects the river. If they're in good condition, if there's vegetation growing, if there are crashing nutrients, they're reaching that area. And well, in the places where there is no native forest or riverine forest, any management that is done has to think in terms of the characteristics of the basin. For instance, to look for what crops to grow In other words, to be able to first detect what the problems are, what nutrients are arriving, what to plant to extract them, and how to harvest them. Also regarding nutrient supply, fertilisation, and agrochemical management. There are a lot of things to be defined. There is not a single answer for it. Right. What I gather from it is that each farm is going to have its own variant. There is no recipe. On each property, you have to know if there is native forest or not and whether there are wetlands or not. The ecosystems of wetlands and grasslands that you see in the country are not protected by law. Many times they are burned, and they try to advance on them in order to replace them with a more productive pasture for livestock feed. There are things, but it depends on what area of the river or what watercourse we are talking about. The larger the watercourses (Santa Luca, Ro San José), the wider the native forest is from the river bank into the field. The watercourses, the tributaries, and the streams often do not have the protection of a riverine forest. So, more than each property, it depends on the area. I don't know if you understand.

Interviewer 1: Yes, And if the buffer zones could be improved, what functions would you change or add to the buffer zones?

Interviewee: It all depends on what kind of watercourses they are defined for, which is why I was telling you about the characteristics. At the headwaters of a river or stream, where you don't have the natural protection of a native forest, you have to think about what management is done, at least in the surrounding soils. From the point of view, for example, of agrochemical applications, there is a regulation that prohibits application within 10 metres of the watercourse. It all depends on where they are to determine what is added or not. But my understanding is that if progress can be made with management plans, you can define in more detail what would or would not be added to implement buffer zones. It is not black and white. I think it's not on a site-by-site basis, but it is for the characteristics of the watercourses that you want to protect. That is fine. From the Ministry of Livestock, for example, from 2006 to 2012, a major promotion was carried out, especially in the northern part of the country, but at a national level, for the construction of reservoirs to provide water for livestock grazing. But these dams had to have certain characteristics to ensure water quality. The depth could not be less than 2.5 metres, and it also had to be fenced off all around it so that the animal could not access the water body. And that is where the drinking water comes from. At the level of our country, it has the characteristic that, if you look at the map and activate the layer of watercourses, it's full of streams and watercourses. So, there is no farm or establishment that does not have a watercourse. This has to do with livestock management. That is to say, fencing off the water zones is something that's kind of insane because there would be many kilometres of wire fencing. I don't know if it's done in the dimension of what I'm saying, but it would be insane. But there is one characteristic: if you offer the animals the needed quality and quantity of water at a drinking location in plots, the animal is not going to choose to go to the water course to drink water, but it will go to that drinking facility. So, when it comes to thinking about the buffer zone or the integrated management of the property with respect to that buffer zone, there are many things that can be done without being too extreme, like fencing it off or prohibiting its use.

Interviewer 2: What problems do you think there might be with that exclusion, apart from being costly, as you said?

Interviewee: It's very expensive. I don't know who's going to pay for it because there are many kilometres of wire fence. I mean, a lot of weeds would grow, and then when it comes to the level of a property, some can be good and others bad. That is, it is also an issue to be taken into account; otherwise, it would not be desirable for any crop to do harm. In addition to the loss of area that can be compensated in some way with some production at the farm level, there is also a loss of access to water for the farm. Many of the fields are managed for livestock farming. In the Santa Lucia basin, we have agricultural areas and livestock areas, and farmers need to be able to ensure water in all the paddocks that the farm has for adequate management and animal welfare. Wiring, apart from being expensive, involves a lot of investment in terms of ensuring water distribution. And not everywhere, but we can have enough water in sufficient quantity and quality to ensure this at the level of the establishments. For example, if one thinks about drilling in a fissured aquifer, not all of them provide the amount of water that one would like. Drill for many metres, and you can get 300 litres per hour if you are lucky. It's not easy; in other words, from my point of view, it's not viable. That is to say, it is not viable on many properties. Whether we like it or not, on farms where livestock is managed, if there is a good stream or watercourse, the animal has access. To the extent that there is no other source of water or distribution to drinking facilities, which is not possible everywhere. Sometimes, from a different point of view, it is not always possible to ensure the quantity of water, but also from the point of view of investments, it is not easy.

Interviewer 1: What do you think would be the benefits of these additions to the buffer zones?

Interviewer 2: I would like to make a comment here. You mentioned that the most important addition is to put water troughs outside the buffer zone to reduce the load. As I understand it, this is an alternative measure to exclusion.

Interviewee: From a production point of view, having water in sufficient quantity and quality is beneficial for animal welfare and even for production, considering the way the animals graze. So, it's very good from that point of view. To ensure water quality, animals must not be allowed access when they drink water. That is to say, a lot of issues would be avoided, but it's not a cheap measure.

Interviewer 1: And what obstacles do you think will be experienced with the implementation of these additions to the buffer zones?

Interviewee: The main obstacle is obtaining economical water distribution. It may not be feasible to have an intake in the watercourse that has permanent water all year round with the depth. In other words, investments are required in the design, at the level of the property, and in the form of electricity provision. In addition, gaining support for these measures is important, which would need funding from the ministry, the World Bank, or IDB funds for water for animal production. Even some projects also had to implement actions in what were called buffer zones, such as preventing the entry of animals, with some future measures. However, these are not subsidies that have reached 100% of the producers, and it does not mean that those producers have obtained any benefit or have managed to implement 100% on the whole farm.

Interviewer 1: And how do you think these obstacles can be overcome?

Interviewee: There are many ways to overcome these obstacles. Part of implementing a buffer zone should, in some way, be legislated with tax exemptions. A lot of dissemination to producers is needed to have a guide, a manual, on what to do according to the characteristics that I have. The buffer zones that we want to define must have measures, and they have to be supported in some way with normative regulations that not only prohibit but also provide other tools to move towards that goal. For example, native forests are protected by law, but some uses are exempt.

Interviewer 2: And that's good, what you mentioned, but how do you see this in regard to control? Because it's good to have a manual, but as far as control is concerned, how do you see it?

Interviewee: Corresponding government organs can regulate control in the field.

Interviewer 2: Today, does the control of the buffer zones fall under the Ministry of the Environment?

Interviewee: The native forests are under the control of the Ministry of Livestock, specifically the general forestry department. The registry of logging permits already has a monitoring programme, and soil management is part of the federal directorate of natural resources. There are several executing units that have control over buffer zones, depending on the regulation. Currently, control falls under the Ministry of Environment. However, thinking about buffer zones implies the use and management of a certain land cover, and it also involves the management of a forest that is probably present in that buffer zone. In other words, they are all integrated into the same geographical area. It is difficult to enforce control, especially in cases of violations such as cutting down native forest, soil erosion, or unauthorised water usage. One of the ways to control this is through complaints from citizens, who can report these violations, and authorities can take the necessary action. There are other mechanisms, such as the obligation to present a use plan at the directorate of natural resources, which includes the interpretation of satellite images to monitor land use plans.

Interviewer 1: How has climate change affected the Santa Lucia River Basin?

Interviewee: Climate change has resulted in changes in temperature, precipitation, droughts, and floods in the river basin. There have been instances of flooding, which have affected livestock production and the population's access to water. The shortage of water has impacted the survival of crops and pastures. Floods are monitored by a directorate within DINAGUA, and in rural areas, producers are responsible for monitoring the water levels. There is no mapping of it today in rural areas. Climate change has resulted in extreme events that are related to inter-annual variations, which can affect milk production and quality. Precautionary measures are necessary to deal with the climatic event.

Interviewer 1: Sure. How do you think these changes will affect the performance of the buffer zones in the Santa Lucia River basin?

Interviewee: Well, regarding whether it's in a flood area, I'm not sure. You would need to ask experts about the water flow and what it carries and leaves behind, because if it's in a flood area, it will likely be flooded. Additionally, the recent droughts have caused watercourses to dry up, leaving them without water, which can also harm vegetation, crops, and pastures. In other words, even with an excellent management plan, extreme events like heavy rainfall can cause significant damage that can't be prevented. I'm not saying that buffer zones don't help, but they too can be affected by extreme events, and soil erosion can still occur, leading to pollution of the watercourse.

Interviewer 1: Do you have any experience with invasive species?

Interviewee: Yes, my degree is in forestry, and from 2006 to 2012, I coordinated a project on responsible forest production with the General Forestry Department. As part of that project, we worked on controlling invasive exotic species in native forests. For instance, we worked on a management project with the Municipality of Florida to control Ligustro (*Ligustrum lucidum*), which heavily invaded the Santa Lucia Chico in the Tierras Fiscales area. There are also areas in the basin towards Lavalleja that have invasive Tojo (*Ulex europaeus*), which affects the natural field and forest. However, there are other invasive species that are more prevalent in the countryside, and I am not familiar with them. One problematic invasive species is *Gleditsia triacanthos*, which can cause a loss of biodiversity as it invades native woodlands as well as edges and fields as its seeds are dispersed by animals. It's also a thorny species, which can make it challenging to control as it spreads over the edge of the field.

Interviewer 2: I have a question. You mentioned that when creating a plan for human use of soils, a warning appears when you are in a buffer zone. However, does this warning not appear in the first or second package of measures? As I understand it, it's not in the second package. At least, there is no official decree that allows fines for violating it, but I don't know if it appears to you or during the control process.

Interviewee: No, the second package of measures does not regulate the buffer zone, so no warning or regulation is set at the system level. However, implementing a warning at the system level is simple, even with the use plan issue. We are currently updating the system, and we have added all the dairy and irrigation information to the use plan management system. Although it is not yet in production, the advantage is that all information regarding the fields or any use made at the level of irrigation, dairy, or agriculture will be available in the system. The system does not have to limit the use of a field when it is intercepted by a buffer zone. Nonetheless, this is not the only stage that is being controlled. There are others that I mentioned, but it is easy to provide information to improve control. The idea is to create a report and identify where to go or what is being done. It is even a tool to implement management measures at the level of buffer zones that could be used to define and control the system. The tools are a question of defining what is being done or where it is going. I think the land use planning is added in Decree 30.0202, which discusses buffer zones, and 19.125. If this is correct, I don't believe it's not there, but I don't know if anyone is monitoring it. It's a decree that was approved by the administration at the end of the previous administration, but I don't think it's been implemented.

Interviewer 1: Thank you very much for your time.

Interviewee: I hope I have provided a different perspective.

Interviewer 2: It's good to have different perspectives. In this sense, it was good.

Interviewee: Impeccable. Have a good day anyway.

Interviewer 1: Bye-bye.

Interviewer 2: Thank you, bye.

*Interviewer 1 is the student, and interviewer 2 is the supervisor.*

# Interview 18

**Interviewee: Scientist        Date: 21-03-2023**

Interviewer 1: What are your responsibilities, objectives or your interests in the design and management of the riparian buffer zones, mainly with the use of geographic training system and remote sensing?

Interviewee: In what was my master's work, I worked with water quality modelling in the department of Canelones, which is an important part of the water discharges for the Santa Lucia basin. And from the work I had, I identified an important role of the buffer zones in contributing to water quality, mainly my work focused on nitrogen-diphosphorus, coming from point and diffuse sources and in that sense, I identified that buffer zones play an important role in the retention of these nutrients.

Interviewer 2: And what were you working with, what methodology or what model?

Interviewee: With empirical models, I did not apply a particular model, but I worked with empirical models, mainly with generalised additive models.

Interviewer 1: And what relationships do you have with other stakeholders in this process?

Interviewee: Well, this process arose from sampling that had been initiated by the municipality of Canelones, which at that time had done it for the years 2008 and 2009, but then continued it. And one of the researchers responsible for that programme, which was called PEDCA, of water quality in Canelones, was one of my co-tutors, with whom I continue to work on those models for Canelones. So the link we have is mainly with the municipality of Canelones.

Interviewer 2: And in terms of the Santa Lucia action plan, do you have any relationship with them? Because I think we came to you through a scientist.

Interviewee: Yes, that's what I got in the mail. No, not with the development of the measures and not with the participation in the discussion of the measures either. Yes, the link is indirectly through the Canelones municipality, not a direct link with the MVOTMA, nor with the ministry of environment at the moment.

Interviewer 2: Okay, so are you working within the basin? Because Canelones has the Rio Santa Lucia watershed and the Laguna del Cisne watershed. I don't know if it's different.

Interviewee: Well, yes, actually it has a part that drains directly into the La Plata river, which is holy to these basins. And then it has the whole area to the north and west that drains into the Santa Lucía. Actually, as I'm working with the whole department, yes, I'm including part of the Santa Lucía basin. And then I worked with other approaches that were not so much linked to water quality, but to changes in the soil, in what is the basin of Paso Severino and the Santa Lucía Chico. That was some years ago. Well, you know very well the function of the Paso Severino Dam.

Interviewer 1: Okay. These buffer zones can be designed in many different ways. For example, by using and promoting different types of land cover and management strategies. So there are different ways of defining these buffer zones. The next questions are about how to actually define buffer zones. What functions do you think buffer zones currently have in the Santa Lucia river basin?

Interviewee: Well, that's a complicated question, because there is a great diversity of buffer zones and there is a great diversity of uses that they have. Well, the function, I understand that it is fundamental, from the point of view of, I don't know if you are interested in water quality or if it goes beyond water quality, but well, in terms of water quality it is fundamental. Not only as control of point and diffuse sources, but also as control of erosion of the land itself. The functions they are fulfilling, I believe, are different depending on what type of areas we are considering, and the uses that are given to them. There are areas that are well conserved and are in livestock farming areas, and there are areas that have been completely modified for agricultural use. So, it is difficult to answer this question for the whole of the Santa Lucía basin. I think there are very contrasting situations.

Interviewer 2: And what do you think of measure 8 of the action plan? It is about this, which I imagine you are familiar with from the action plan; about the definition of buffer zones in Santa Lucía. Well, in general terms, we know that they fulfil the functions of retaining nutrients and some other functions.

Interviewer: Well, I think that with respect to the measures, I think that they have been a good start in that they have put the importance of the buffer zones on the political agenda, but they have been timid in terms of their definition and the stringency with which they are being monitored and supervised. One thing is what is said to be being done and another thing is when you go out in the field and see what is really happening. In other words, we have come across areas where, in theory, cattle cannot enter, where there are 200 cattle that are grazing directly on the course. I have not taken it upon myself to do an evaluation or an accurate monitoring of what is also working. The impression I have is that I have gone through different areas for other purposes and not exclusively for monitoring the riparian zones. I see this as a good start, but it is completely insufficient and has problems. Firstly in defining the buffer zones, in not considering geomorphological and operational criteria that can solve some of the problems, such as the definition of the boundaries that are defined in certain zones or with different distances. But also I think they have major difficulties in terms of design and then in the control of whether they are fulfilling what they are designed to do.

Interviewer 1: What characteristics or physical aspects support these functions?

Interviewer: Well, there are characteristics in terms of delimiting, let's say, considering the geomorphology of the area. Basically I'm referring to the flood plain that you can consider it in its entirety. And secondly, issues that have to do with vegetation, the recovery of native vegetation. Basically, well, in some areas it is wetland, in other areas it is grassland and in other areas it is riparian forest. So, the first criterion would be to restrict activities to the extent that certain activities could be carried out according to geomorphological criteria and, on the other hand, to restore the natural vegetation according to the type of riparian zones that we should find, right?

Interviewer 2: Well, and there in the restrictions, what restrictions do you see, for example?

Interviewer 1: If the buffer zones could be improved, what features would you change or add to the buffer zones? I'm thinking of measure 8, right? So the restrictions of modifying the vegetative cover, not applying agrochemicals and fertilisers in the buffer zone. What functions did you think the buffer zones should have in the Santa Lucia River basin?

Interviewee: Well, the functions go along those lines, don't they? Primarily the control of the materials and the compounds that are coming in. Secondly, the control of other functions, such as the control of erosion of the river banks. And then, in other cases, they could also fulfil functions of supplying raw materials or playing some other role, but with control, right? In other words, at present, buffer zones are acting as or are interpreted as a nuisance for the development of agriculture. And well, I think we are at the time to make that leap and to understand what other functions they have for some linked to mental quality and others that may have functions, either thinking in terms of ecosystem services of provision, support and even cultural.

Interviewer 1: And what characteristics do you think should be changed or added to the design of buffer zones to support this change of function?

Interviewee: Well, some of the ones I already mentioned linked to the definition of the buffer zone itself and the recovery of natural vegetation. And then also with the design of the land adjacent to the buffer zones. If we cannot yet make progress on a good design of the buffer zone, we should think about a good design of the adjacent zones, but it is also unreasonable that there should be processes of recovery of buffer zones with intensive use that start on the other side of the buffer zone. So, initially, progress should be made in this recovery, but not ignoring the fact that the areas adjacent to the buffer zones will have an impact on this recovery process, either as a possible complement or as a possible reduction of a source, or both? No, more as a possible decrease. I can't imagine a recovery process of some buffer zones without the control of the adjacent agricultural zones. We are thinking about recovering native vegetation, but next to it there is industrial agriculture that uses high volumes of agrochemicals, which would be impossible to think about. It would be impossible to think about it.

Interviewer 1: Can you rate these characteristics according to what you consider most important on a scale of 1 to 4? You mentioned that the nutrient retention is important and the work in the surrounding areas is also important. Which is more important on a scale of 1 to 4?

Interviewee: If you want to move forward with an efficient proposal, I can't consider it as independent. I cannot consider moving forward with the basin without considering the rest of the uses that are being done in the basin, and particularly those that are being done in the area adjacent to the buffer zone. I understand that political policy has to have a beginning, and that the beginning should be the design of the basin. Well, in fact, that is how the Ministry of Environment has gone about it with that intention, not focusing basically on the final phase of the processes and advancing more on mitigation than on planning. So, well, actually the two should have a value of 4. I can't imagine them as independent, but, well, if we had to start with something, maybe the beginning should be, at least, with a good delimitation of the buffer zone with geomorphological criteria. Good. Going a little deeper into this, let's say, if the definition criteria are geomorphological, part of the area adjacent to the buffer zone would already be included within that zone. For example, well, if we imagine a classic planning of the basin, well, we have a whole part in which, well, we are going to find water, with native woodland and then other parts that we are going to find with pastures that are going to be flooded during part of the year. If the criterion for defining the buffer zone includes this flooding area, at least for part of the year, this area could act as a protection zone adjacent to the wetland that is flooded on a more permanent basis or where there is tree vegetation. So, well, that reinforces my conviction that the beginning is the broad delimitation of the riparian zone from the floodplain.

Interviewer 1: The next questions are about the implementation of these different features. What do you think would be the benefits of these changes or additions to the buffer zones?

Interviewee: Benefits for whom?

Interviewer 1: For society. In terms of what would be new?

Interviewee: First I'm going to explain a little bit. First there are the benefits and then there are the problems, that is, there are going to be disadvantages that arise from this implementation, which perhaps will frame the question a little bit more. The point is that the benefits for society in the buffers is a bit difficult to answer because within society there are different interests and there is not going to be a single answer, a unique answer. For example, for those who are in charge of the development of agricultural production in Uruguay, the benefits are going to be different from, for example, those of us who get our drinking water from the watercourse. Even at the governmental level, the Ministry of Livestock has different interests than the Ministry of the Environment. Beyond the fact that, well, that in part of the discourse they are aligned and that they make some agreements to move forward in some areas. Or in some aspects, the interests are completely different, even opposed in many ways. For the Ministry of Livestock and Agricultural Fisheries, the development of the buffer zones was a problem, it generated inconveniences some years ago in any attempt to reinforce the regulation. So, as far as the benefits are concerned, I would divide it into two, maybe two large blocks, at least those that are aligned with the logic of agricultural development and the frustration of the basin and those that aim to improve the environmental quality of the basin for different purposes, or because they live there, or because they are supplied with drinking water from the basin, and so on. So, for the former, the benefits would be that the development of the buffer zones would continue in a typical way as at present. And for the latter, the benefits would be a strict consideration of buffer zones and a control and monitoring of the evolution of these buffer zones. And again, surpassing the initial proposal of the Ministry of Environment of several years ago and moving on to more profound issues, let's say, of not only considering the buffer zones based on certain distances, but also considering the defined distances from the Paso Severino reservoir, but from the main bodies of water that later drain towards these reservoirs and towards the Santa Lucía River.

Interviewer 2: And you mentioned there that there is cattle ranching or agriculture and forestry development in those surrounding areas, maybe they benefit from leaving it as it is, that is, without expanding. Do you think it could have? Do you think it is somehow a cost or a disadvantage for them to have a larger buffer zone? Do you think that there could be any benefit for these types of people if there are more buffers? Or is it just a loss?

Interviewee: At the moment I see it only as a loss for them, because it implies a reduction of the area, and that translates into a reduction of income, or it implies in some cases a technological change that also in the short term is going to imply costs. You are thinking about it in a context that today it would be difficult for me to think about in Uruguay, and the alternatives go in two directions, right? Well, on the one hand, to convince us that these activities are incompatible with what is expected for the basin, and therefore there would be many activities that should be prohibited in certain areas. Or on the other hand, as happens in other parts of the world, that these producers would obtain some benefit for not carrying out these activities in these areas. I don't see this second instance in Uruguay today. In other words, compensation, shall we say? Yes, or, well, the framework of payment for ecosystem services, or, whatever framework you want, let's say, so that basically they no longer carry out those activities or with those characteristics in those areas. Today, in the Uruguayan context, I see it as a long way off.

Interviewer 1: Yes, what obstacles do you think will be experienced with the implementation of these changes or additions to the buffer zones?

Interviewee: Well, the main obstacles are the clash of interests, mainly due to an agricultural sector that has great influence and importance in Uruguay and that through different mechanisms is trying to advance in the Santa Lucía basin. The current context in Uruguay is to allow these actors to continue advancing, to allow industrial agricultural production to continue advancing in the Santa Lucía basin, and even to allow forestry to continue advancing in the Santa Lucía basin. So, the first obstacle I find is the political weight that these actors have, the participation they have in the national economy to allow these types of measures to move forward on the one hand. On the other hand, these types of measures, as I mentioned, which are concentrated in the final phase of the process, can also be seen by these actors as an opportunity in the sense that they would not prohibit them from continuing to develop their activities. Well, if one scenario is not to be able to do it, other scenarios are to do it as I am, and another scenario is to allow these conservation measures to move forward without jeopardising the development of the activity itself. So, although it could be an obstacle, at some point it could also be understood as an opportunity for these actors to be able to continue with these activities without questioning the development of the activity itself. For example, I don't know if you are aware of this, but a couple of years ago, the Canelones municipal government prohibited the development of industrial agriculture in some areas. Well, it is moving forward for the whole basin. I understand that it would be a strategic solution for some actors, including industrial agriculture, to move forward in the implementation of buffer zones and not to compromise the development of their activity in a certain area.

Interviewer 2: Would it be something you would be content with is this vision is forwarded? Because what I feel you are saying, you are showing a more systemic view, that the problem is that we go beyond the buffer zones and that it is not solved by that alone. Would it be something like a piece of candy to make you satisfied, and I'm sticking to this vision?

Interviewee: Yes, completely. I am quite critical of the buffer zones as the ultimate solution to the problems. Yes, I understand that they add up and that they are a great contribution and that we have to make progress on that, but that it is a middle or final part of the process and that there is a set of problems that are linked both to agriculture and also to the lack of sanitation in some areas, excessive consumption or the application of water measures in other areas, where the buffer zones can contribute but cannot solve all the problems. So, well, yes, maybe the candy analogy can work well in that sense. And that could be problematic, but you insisted that it's good that they develop, because you can say, no, this could be that in this power struggle, it could end up not advancing what you want because you're giving a piece of candy. That's why, of course. But it's still better than what we have now. That's why I believe that it is difficult to make progress overnight in the solution, and that different measures that come together to solve the problem are welcome. And in this sense, I understand that the development of the buffer zones is fundamental. Now, if it is the only measure that is going to develop, it will be insufficient. But, of course, it's preferable that it develops where it doesn't develop.

Interviewer 1: That's perfect. And how do you think these obstacles can be overcome or what possible solution can be implemented to overcome them?

Interviewee: Well, possible solutions for these obstacles I see as complicated in the sense that they are opposing interests. The solution to this obstacle could be, on the negotiation side, at least to start by defining some priority areas where it is certain that things cannot be done. Well, for example, a strategy similar to the one that the municipality of Canelones has adopted. Well, industrial agriculture can be banned in the whole department. Well, at least it should not advance in these areas. In that sense, not strictly for the industrial agriculture, but for the organisation of activities. I think that the solution has to go that way, to think about planning in the basin and then to deepen or concentrate the efforts of the buffer zones in those places where it is more complicated to advance with this planning. Maybe that's the way I would think about it.

Interviewer 2: Good, good idea. Tell us a little bit, do you have information you can share it with us regarding the implementation of the restrictions on the farms of Canelones? How it was implemented, and if it can be implemented and what problems it caused. I am interested, not only in these dimensions, but in this more systemic solution that you are proposing. I find it interesting.

Interviewee: Well, that was a process that is still going on, let's say, it's not closed because it has brought quite a few problems. Around 2011, 2012, I'm not sure of the date, that process was initiated between the rural development agency and the sustainable development department of Canelones to try to move forward in that sense due to different problems they were having. Some were linked to water quality, others were linked to conflicts over the use of agrochemicals, others were linked to the development of agriculture itself. So they began a process of dividing the department into different management zones, and in those management zones they began to analyse what activities were currently being developed and what activities should not be developed in that zone. So, well, in some areas there must be a high concentration of population and where family farming was also very important. What is being prevented is the advance of feedlot livestock farming, industrial agriculture, mainly linked to summer agriculture; soya and maize production. So, in the entire metropolitan area, which is more densely populated, new feedlot farms should be prohibited around the whole area around La Paz and Las Piedras. Of course, there were some difficulties with some buffer zones that were already planned to move forward in that sense, and that is what has brought many complications in the development of the plan. I will share the document with you later if you are interested, it is the Canelones land-use plan and, well, at the moment it is a set of proposals that have many implementation problems. But beyond the implementation problems, the spirit in which it is created and the logic and some of the principles behind the development of the plan are very interesting, and it would be good if you could consider them. I'm not sure how much weight is given to the issue of buffer zones and riparian zones, but it is intended more as a more integrated approach to the watersheds and the development of agricultural and livestock farming in the watersheds.

Interviewer 2: It would be great if you could send it to us.

Interviewee: Yes, I know. Because it's interesting as a case.

Interviewer 2: I'm going to ask one more question. There's no mention of any reduction in agrochemicals or fertilisers. What about the role of small-scale farming? Thinking about it, if you were planning, thinking about the decision-maker, you are at least lowering the key to industrial agriculture, but also family agriculture, fruit farming, and horticulture have a fairly significant use of pesticides that not only pose a risk downstream, but also for the applicators themselves, the people who are just a few metres away from their crops. The other day we went to visit a horticulturalist, and he has a house there, and he puts glyphosate on the apple trees.

Interviewee: Well, this is an issue that transcends industrial agriculture and that basically takes place in almost all the activities in the area. The big difference are the volumes and the scale with which these products are used, and the scale of production, and that determines that. In general, industrial agriculture is a bigger problem for the environment than family agriculture. Not to mention the fact that the toxicity of these products goes beyond the activity itself. In fact, there are health problems in almost all of Uruguay due to the use of these products. In this sense, the municipality of Canelones has also been one of the sectors at governmental level that has had more weight in the promotion of other types of practises. For example, the Laguna del Cisne with the proposal for an agro-ecological transition and also with support for family producers who decide to go down this path. So, as an option, yes, it is the problem, let's say, but it is mainly developed in industrial agriculture and within the municipality there are some proposals to advance in the reduction of these types of products and to move towards sustainable modes of production. If you are interested in this issue, it would be a good idea to get in touch with them. I will then pass on the contact details of Matías Carambula, who was in charge of the Rural Development Agency in Canelones. Now he is no longer here, he is in the Faculty of Agronomy, he is an agronomist, and he was one of the people who led this process and who was also closely linked to the proposal for agro-ecological transition in Laguna del Cisne. So, if you are particularly interested in this, this is the person to talk to.

Interviewer 2: This goes off topic a little bit, but I was curious, since you are already scratching this topic, I think it is important to at least discuss it and see the other experiences.

Interviewer 1: How do you experience climate change in the Santa Lucia River Basin? Think about changes in temperature, precipitation, droughts, or floods that you have noticed over the years?

Interviewee: Okay, well, the topic is pretty much a moot point, isn't it? And the boundaries between climate change and climate variability are quite contentious as to whether or not we can talk about the incidence of climate change, particularly in the basin. What I think is that we are not prepared to work with the uncertainty that both climate change and climate variability offer. That I think is the main problem, that almost all the functioning of the basin is designed for scenarios in which these things do not happen and these are issues that do happen. I don't know if you are there now, but we are facing one of the most important droughts of the last ten years. So, how much is climate change, climate variability? Well, that's the discussion, but I think the solution is not to discuss it and put a name to what it is, but to get used to the fact that it is possible that these events may occur and that they may occur more frequently or be more severe. And that a large part of the impacts that are occurring now, of course, have to do with the decrease in precipitation, but they also have to do with the changes that have been promoted in the basin and that have not been made with the possibility of these events occurring in mind. Water consumption in the basin is much higher than it was 20 years ago. So, in the face of a decrease in rainfall patterns, the impacts we are going to have are much stronger. So, I think that the impacts of climate variability change are increasing, as a result of the actions that we are developing in the basin, and are generating a scenario of greater vulnerability. We are more exposed and much more sensitive to values that are far from the average rainfall, on either side, for drought, but also for floods. For example, floods in the city of San José or floods in Santa Lucía or in other localities, generate more and more impacts. There are more people affected for a longer period of time. Now because of the drought, more products are affected, and again, thinking about it as a system and going back to what I was saying today, there are many of these problems that could be solved with management at the basin level, where the buffer zones could have an impact. But they understand that they are limited and that today they are not being thought of as such. In other words, they are being thought of as a good thing, whether it happens or not, and no measures are being taken in terms of the uses that are being developed. And something that is worrying, I don't know if you are aware, but there is a whole group of land units that historically have been under discussion whether they can or should be added as forestry priority zones or not. There is a very important lobby by the companies that are pushing for the growth of forestry in Uruguay, such as UPM, for these soil units to be included within the forestry priority zones so that forestry can advance in these areas. And these units are very present in the Santa Lucía Basin, mainly in the headwaters of the Santa Lucía Basin. So, in the event that these areas are finally integrated as priority forestry areas, it is expected that forestation will grow in these areas.

Interviewer 2: What specifically are these areas?

Interviewee: The 502B. And they occupy a very important area in all the headwaters of the basin, especially in the north. Well, all of this is being discussed, and the discussion is more about the growth of forestation and not so much about the role that these soils and these buffer zones should play in terms of the catchment. No more and no less than for one of the basins where water catchment is the most important situation for a large part of the country's population and which is now evident with this drought we have had, where the Canelón Grande dried up, a severe step that at some point was doubted as to the possibility of providing water by running water. Well, in a scenario of increased forestation, this situation could be much more critical. Here we are not talking about quality, but about water flow. We are talking about quantity, and that gives you the idea that the considerations about the change of climatic inheritance that exist in the Basin function are more a discourse than a real concern. Well, at certain times there are some projects with international funding that focus on climate change and there is some progress, but in practise I understand that it is not a real concern.

Interviewer 1: And how do you think these changes will affect the performance of buffer zones?

Interviewee: I don't know. If I tell you I would be making something up, I don't have an answer.

Interviewer 1:  Do you have any experience with invasive species in the Basin?

Interviewee: No, I am a geographer and I have not worked on invasive species. I have, let's say, some notions from working with people who work with that, but it wouldn't be serious to put me over time at the end. But well, at a diagnostic level it's a growing concern. The issue of mainly woody species is a growing concern and there are several studies, but there are many here in the Faculty of Science, some more linked to the estimation of surface area and others more linked to changes in ecosystem services. Then, if that is a topic of interest, I can also pass some of them on to you.

Interviewer 2: Yes, please, I don't know if I can do it now, but I would be interested to know who is working on these issues later on. Well, I don't have any more questions either.

Interviewer 1: Me too. Thank you very much for your time.

Interviewee: Well, very good work. We are also at your disposal for anything we can contribute.

Interviewer 2: And we will inform you when the result of Alfred's thesis is ready to be disseminated.

Interviewee: Very good.

Interviewer 1: Thank you, have a good time.

Interviewer 2: Thank you very much.

*Interviewer 1 is the student, and interviewer 2 is the supervisor.*

# Interview 19

**Interviewee: MGAP    Date: 23-03-2023**

Interviewer 1:  What do you currently do for a living and how were you involved with the implementation of the buffer zones in the past?

Interviewee: You're talking about the implementation of the Santa Lucia River Action Plan?

Interviewer 1: Yes.

Interviewee: Now, I'm living on a farm, in the countryside, 40 kilometres from Durazno City, but the farm is in Florida, and I'm working on the farm and I'm teaching and doing some research at the University in Durazno. My area of expertise is soil management. I am an agronomic engineer and I did my master's in soils.

Interviewer 1:  Perfect. And do you know of the Santa Lucía River Basin Action Plan?

Interviewee: Yes, because when I used to be at the ministry of agriculture, I started going to the Santa Lucia river basin commission for about two years, or maybe a year and a half, and then a colleague of mine continued going to the commission. And I remember the action plan and I discussed it at that moment with all the participants of the Comisión de Cuenca, and it was, I don't say, a difficult plan when we made it, because there are a lot of different visions about things, no? Kind of a fight, but between producers and farmers, between people who live in the city and have this vision that agriculture and farmers are doing awful things for the water quality, and all those things were difficult to discuss at that moment.

Interviewer 1: Perfect. And what were the MGAP's responsibilities and objectives in the design of this Action Plan?

Interviewee: Well, there were people from the government that were MGAP, and then at that moment there were also people from DINAGUA and the ministry of environment. Our objective was to try to understand what things from the production area and also conservation, for example, soil conservation, can be represented in the plan, you know, and to find an equilibrium between those visions. The ministry of agriculture had at that moment a very important soil conservation policy that we think that it's very related to water quality because of the erosion, no? The erosion of soils has a very important influence here on the water quality because of the sediments that end in the water with maybe some nutrients and so it was very important to talk about those plans and that you can be soil conservative or water conservative and also try to reach high production in agriculture and cattle farming and all. So when we took that message to the river basin commission and tried to discuss and understand all the processes and you know that the plan, we can put everything that is already being done by the ministries and society into the plan. That it's not, it was not to build another brand new plan, you know, but we can consider it part of the plans, all the things that other people were doing already, no? Like this soil conservation policy, all the policies in the ministry about using pesticides and you know that it was important to get all those things into the plan. That was a very important thing that we did at that moment.

Interviewer 1: Okay, and what tasks did those responsibilities encompass? What tasks were in this soil conservation policy?

Interviewee: Not only for the Santa Lucia river basin, but for the entire country. They were some plans in some regions at that moment, but it's a plan to be applied all over the country. Those two were the most important because they are the ones that have more incidents on soil and water quality, pesticide use, responsible pesticide use, soil management policy and all that. La Dirección de La Granja has a lot of policy about also using pesticides and they have all the little farms that have vegetables and fruit with identification and geolocalisation and they have a map with all the farms are, for example, to protect the applications of pesticide to the fields. In schools, they're all georeferenced to have control of the applications of these products, pesticides in these vegetable farms and fruit farms that are very intensive.

Interviewer 1: Okay, thank you. I am really grateful for your ability to do it in English because in Spanish it would be very difficult for me, so thank you very much for the possibility to do it this way, because otherwise Guillermo would be here and he would be kind of my backup in Spanish, so it's really great that we can do it this way. One of the specific measures in the Santa Lucia River Action Plan were riparian buffer zones. These buffer zones can be designed in many ways, for instance, with different types of land use or land covers, or management strategies. There are just different ways to characterise these buffer zones, for instance, with or without a fence, or with trees or with grass to name a few. The next few questions are about how these buffer zones are characterised, according to what you think with your background as a soil manager could be best characterised. What functions do these buffer zones currently have in the Santa Lucia River Basin?

Interviewee: I think buffer zones are to catch the possibility of nutrients and pesticide residues or things that come from the productive land. On their trip to the river, these buffer zones catch them, so they have a very important function to impede all these nutrients and pesticides get to the water. That is the most important thing. If there were no things to get to the water, they will have no function. And it's an existing situation, so they are important to catch all the things that go from the land to the river. Because of that, it's very important to choose which spices or vegetables to have there, because all these nutrients are going to accumulate in that buffer zone. And once they are there, or nutrients are there, they are accumulated. You have to consider what species, with what growth, at what time of the year, you know, to have in that buffer zone. Do you understand?

Interviewer 1: In order to improve the implementation of these buffer zones, we have talked to different farmers, for instance, and it became fairly apparent that some farmers implement them and some farmers don't, for several reasons. How do you think that these buffers can be changed in order to increase their implementation? Should they, for instance, include a function of productivity for the farmer to make it preferable for them to implement them, or should there be a change in management of them, or how do you think that this could be achieved?

Interviewee: For example, thinking about the buffer zone and its objective, I think that there have to be plants that have a lot of production, because if you have, for example, phosphorus and nitrogen, you need a plant that uses it, so the accumulation there is not very high. And then you can plant or sow, I don't know how to say it, some productive species, and maybe with good management, producers can use it for cattle alimentation, for example. Because if they're in the buffer zone, it's grass that doesn't produce. For example, in native grass, maybe there is a risk of accumulation, so a lot of nitrogen, for example, or phosphorus is there, and sooner or later, maybe that phosphorus ends at the river. So if you plant there, for example, a pasture with high production, that pasture can consume those nutrients, and maybe the farmers can use it to feed their cattle. Maybe in a way so that the cattle don't excrete at the buffer zone, maybe getting into the buffer zone like four or five hours a day, not every day and all the day, that they can use that buffer zone. Maybe they can do it that way. There has to be a little study on which one is the best way to do it, to do the grazing, maybe three hours a day, maybe some days. There has to be some plan of grazing for the farmers, because like we say here, "la cura es peor que la enfermedad" (the cure is worse than the disease, red.), figuratively speaking. But maybe that way they could be. The other thing that I remember at the river basin commission is that the cattle were not able to get into the river, so the farmers have to have drinking stations where the cattle go and drink water far away from the river, and that has a lot of costs, no? They have to get a pump, and also they didn't like to do that because of the cost and the complications, no? But maybe if we can reach something good for both, for water quality in the buffer zones and the farmer can do something there, carefully, thoughtfully. Maybe it's a way to solve these problems that are between the farmers and the policymakers.

Interviewer 2: When we talk to other people, for instance, you already mentioned someone, we already talked to her about the soil use and management plan from the MGAP. How do you think that this instrument can be used as a tool to enforce or control buffer zones?

Interviewee: Well I think that the plan is excellent, that plan was developed when I was in the direction of natural resources, but I think that there's a problem, it's more social maybe, no? Because in technical discussions, maybe you can agree, like what is the importance, for example, of the soil management plan or other policies or other actions, but sometimes it was difficult because the discussion was not technical, it was more like, let's say, philosophical. I think that agriculture is a bad thing, so anything that comes from there will work, and sometimes the discussion was more like that and not technical. This commission was very big, with too many people there, so sometimes it was difficult to agree on actions because of that. But I think that everything that improves the river basin management, not only the buffer zones, the entire basin, it will be good for the quality of the river. Because it's not only if you do everything bad and you put the buffer zones, you don't have all the impact that you need for the water quality. The most important things in my vision are to do the proper things in all the basin or the watershed, the land use, the best management practises, the fertilisation, good use of all products, crop rotations, and all the land management in all the watersheds. And to improve all that, you have the buffer zones to help and to support all other things. But if you only focus on the buffer zones, maybe you are not improving, you are not attacking the whole problem. Understand?

Interviewer 2: Yes. I have two follow-up questions for this. The first one is; you talked about how it would be necessary to look further than just buffer zones. In the action plan, there are, I think, 12 measures or 10 measures taken that focus on improving the water quality. How do you think that this plan can be improved with the adaptation of different measures or changes in these measures?

Interviewee: I didn't understand the question. You say what else to consider in the plan. I don't remember these or 10 or 12 measures.

Interviewer 1: Okay. No, then let me try to rephrase it because it's a difficult question. What measures do you think can work in synergy with the buffer zones to improve the water quality?

Interviewee: The measures, you mean like things to do?

Interviewer 1: Yeah, management actions or...

Interviewee: All the use of land, the use of soil, for me, is the most important thing to help the buffer zone because the thing is all the nutrients and soil particles and some pesticides that go out from the land can be retained. If all these things stay on the land, you wouldn't need a buffer zone because there's nothing to catch, but that is impossible because every human action on the land makes some problems in soil loss. But the erosion of soil, for example, is one of the most important problems that we have in agriculture. If you have plans to lower the soil erosion, to mitigate it, it's a very important thing that helps all the water quality in the basin. This was at the plant level, but maybe you have to do a lot of laws and plans and policies and some people do it and some people don't. And for example, if your focus may be on the Santa Lucia Basin adjust the monitoring and try to let people do things well, not in the whole country. Soil or farm planning, with good practises, making land use plans, that for example, adjust the fertilisation of the crops in soil analysis. All this good management will be improving all the buffer zones, they are in most of the laws, but it also has to make sense regarding fiscalisation. Maybe in these basins that are as more vulnerable or more important, but everything is land use; proper land use, and management, would help a lot. And then when all the industries and all the, what is point sources, but it's not so difficult because you know exactly the location of the contamination. For example, if you're talking about a diffuse source, the contamination is more difficult to avoid, no?

Interviewer 1: You already mentioned one potential obstacle which could be, you said, the social process with the implementation of these measures. Do you think there are any other obstacles that might be relevant to keep in mind when looking at the implementation of buffer zones?

Interviewee: knowledge, like studies, because sometimes when we talk about buffer zones, we say, no, let's plan, let's say, native species or like it's a buffer zone, there has to be something completely natural and we don't know that. For example, I think that there have to be some high, as I said, high-yielding plants, but we need some investigation. Local, national maybe, to design this buffer zone, because it depends, for example, the width, it depends on the land use, the slope, and other things, and sometimes we need to know more to make good designs, no? Because sometimes we are applying things for the research institution, or there is a very good work that made USDA a lot of years ago that helped a little bit in design, but it's very important, I think, for small farmers, fundamentally, that those buffer zones have a good design, no? For example, 100 metres, maybe it's too much for a farmer that has a small farm. So I think that we need to know more about these buffer zones, in size, in species that we can use, and how frequently we can maybe put some cattle on them; maybe two hours a day, I don't know. I'm thinking this, but I think that it would be better to have more knowledge about them.

Interviewer 1: And if you think about these obstacles, how do you think they can be overcome? Or what solutions do you think there might be?

Interviewee: Honestly, I don't know, because I've been there almost three years, and I didn't see what to do with some of the obstacles when there are these hard discussions where nobody hears what the other one is saying. And I think this, and I'm not going to move from my way of thinking. I think that that is very difficult. I thought that maybe with this knowledge and studies, and because when we did the soil management plan, we had a lot of knowledge generated by the university, all the farmers agree with the erosion process, and all the farmers agreed with the importance of the control of soil erosion. So that process was very construed, we build it with all the farmers because the farmers had to do a lot of things that they didn't want to. But in this case, it was very difficult, because people from the city, most of the time didn't know what happens in the countryside. They think that farmers are enemies, that farmers are contaminating everything, and that they don't care about the environment, they think that all that. And in that situation, I think that it's very, very difficult to solve the problems. I think the one thing that will be good, it's to make some sub-commissions with not so many people. So maybe sub commissions with 10 people that maybe when people are getting buffer zones, they are going to supply information, to share the what and how, things like that. Because sometimes in this big meeting, there are maybe 50 people or more from different organisations, so maybe it would help to make sub commissions where these different things or topics can be discussed, and then in the plenary, make the presentation, I don't know, maybe. Because in my opinion, to take decisions you need knowledge if you can get it. You have to take action because you know, sometimes you don't know, but there is knowledge because there is a group in university, a group of people that study this for 40 years, and they can tell that this is working like this. And if you don't have the knowledge, try in the middle of the process to generate it, support research, and things like that.

Interviewer 1: Okay. Thank you. I have one last topic I want to discuss, and it's about how you experience climate change in Uruguay or the Santa Lucia River Basin. Of course, as a soil scientist, you don't have to call on hard scientific evidence, but more so on your perceptions of what has changed. How do you think climate, or how did you experience climate change in Santa Lucia River Basin in regards to, for instance, temperature, precipitation, droughts, or floods?

Interviewee: Well, what I heard in the IPCC and other groups and things, that occur because of climate change there are increased frequency and intensity events. Uruguay has forever this high variability in its climate, you know. For example, the average precipitation in Uruguay, we never have a year where the average precipitation occurs, it's like 60% of the variability between years in precipitation. But that's what I heard and then I think that it's occurring, the intensity of the events increase and more frequent, you know. For example, we are suffering three years of drought this year and two years prior. And the droughts are more frequent and the events are more intense. Just like the precipitation, but not the floods. We have, I think that that occurs, but I know some people say that this is because of climate variability and not climate change. But I think that you can see every day that the intensity of events increases.

Interviewer 1: Okay. And from your experience, how do you think these changes might affect the buffer zones in their functioning or their management?

Interviewee: See, I think that, okay, if there are increased frequency of rains and more intensity of rain, it will be more important to accompany these buffer zones with all the other things noted, because maybe they will not be enough alone. So, in a scenario of climate change, I think that it's more important to make all the package, to use the buffer zones, but especially also the land use properly, and use good practises on all the land. Those things will be very important both in the land use and the buffer zones, if there was a scenario where, for example, the rain will be more intensive and more, more frequent, I think. But as a colleague always says, "if we are prepared for what is happening now, we will be more prepared for what will happen in the future". So we have to do things right for our situation, and that will get us better prepared for what is coming.

Interviewer 1: Okay. And then one last question that I have is, we haven't touched upon this, but do you have any experience with invasive species or exotic species that might influence the buffer?

Interviewee: No, I don't have experience with what I've been reading since a few years ago. It needs to have species with high production, so they can consume all the nutrients that are retained in the buffer zone. But I don't have experience, but I imagine crops or, or plants that are high, dense, and with high productivity that can grow with all the nutrients that we have there at the buffer zone. In Uruguay, I didn't see anything, I don't have any experience with it. I don't know. But, as I said, I think we have to do some research about how to simulate buffer zone, plant different species, and monitor there the level of, for example, photosynthesis and whatever we want; to monitor and to see if, if the species there have effect in on the nutrients there or whether it's the same. What we need is only some metres with no cropping or with no cattle. And that's enough. I don't know that.

Interviewer 1: Okay. Excuse me. I forgot to ask one thing. In regards to improving the buffer zones, you mentioned increasing knowledge and research. You mentioned the creation of a possible new commission.

Interviewee: See, not new commissions, you know, smaller commissions. Not a new one. For example, the 60 people, I don't know how, but sometimes when, when I used to be there, there were maybe 70 people there. It's very hard to discuss with too many people. So sub-commissions, you know, maybe 20 people working buffer zone, other 20 in, so can say for example, other stuff, things, and then once, for example, in six months or, or once in a year, all the sub-commissions get together in the big commission and then maybe present the results of their work and the, yeah, not a new one because we have a lot already of things.

Interviewer 1: My mistake, I misunderstood.

Interviewee: You know, when you finish the interview, if you have some questions or things, you can reach me again and then we can, you know, have a second talk about this.

Interviewer 1: Of course. So as improvements, we, you said, increased research and knowledge, the creation of sub-commissions, and sustainable soil management, and also I think the combination with other measures on a larger scale to improve the synergy of the implementation. If you had to score these solutions on a scale from one to four, where one is unimportant and four is most important, what score would you give them?

Interviewee: The first three would be most important in the buffer zones, maybe the sub commissions, but in thinking about technical things, number one is soil use management in the watershed, and to generate more knowledge, to acknowledge the buffer zones and it's possible to impact in here in the landscape. And the other thing that could help, it's number three to make some sub commissions in the river basin commission, to get better discussions of them.

Interviewer 1: Okay. And so the scores were on a scale of one to four.

Interviewee: Ah, the score, the importance of land and soil use four, knowledge four, and sub-commissions two.

Interviewer 1: Okay. Clear. Thank you very much. Um, do you have any other questions?

Interviewee: No, no. No, I just remember all my, my period sharing the commission that Cuenca that some days were so, so hard, but a very good experience.

Interviewer 1: Indeed. Thank you. I don't have any questions item. I want to thank you very much for your time.

Interviewee: No, thank you. And if you think that some topics were not clear or something, you can write me again and no problem.

Interviewer 1: Thank you so much. But I think that most of it was very clear. It was very, very great that we could do it in English. It was very nice to do it. And we really appreciate your input.

Interviewee: Okay. Okay.

Interviewer 1: Thank you. Then I will stop the recording here and, yes, I'll stop the recording here.

*Interviewer 1 is the student, and interviewer 2 is the supervisor.*

# Interview 20

**Interviewee: Vida Silvestre    Date: 23-03-2023**

Interviewer 1: You already mentioned this, but how exactly was Vida Silvestre involved in the early stage of the proposal of buffer zones, or I guess more in a broader sense, the Santa Lucia River Action Plan?

Interviewee: Yeah, well, it was actually before that, it has to do with the proposal of incorporating Santa Lucia in the National System of Protected Areas, so I don't know. But we were doing a lot of on-the-ground work with environmental actors, we were working in the area on different topics from environmental education, to doing some research on effective rate physiology and invasive species, invasive tree species. So we're doing a range of different activities in the area, so when the idea of presenting the area to be incorporated into the National System of Protected Areas, we got engaged, and we participated in the preparation of several of the technical documents that supported the proposal. And actually, we do participate in the advisory committee that the area has. And then we also got engaged in, you know, there's a layer of commissions that have been created to manage different aspects of that territory, so we are also participating in the commission that has to do with the management of the water basin (Santa Lucia river basin commission, red.), and so we do have a permanent involvement in the parties that access to discuss how to promote sustainable development in the area, although the process has slowed a lot in the last three or four years, and we haven't been pushing much, so we've participated in the meetings where we are requested, but we are not pushing any specific proposal. We do work with some landowners that have productive farms there that are concerned about the need to incorporate the conservation dimension in the way that they produce. So we provide some advice to them, we help them to improve the way they produce, especially cattle farming or in some cases agriculture, by trying to leave areas that enable biodiversity conservation as well. But that again, that's with some specific landowners helping them to implement conservation measures within the properties. To create more and more integrated participation in promoting a common vision and actions at the scale of the whole protected area. That's I think the summary.

Interviewer 1: And what exactly are the goals of Vida Silvestre in this process?

Interviewee: Well, as I said, we don't have a dedicated programme right now. It's more like there are several things. One of our main activities has to do with promoting private land conservation. So we have a range of stakeholders throughout the country with whom we work in trying to help them to incorporate conservation as a dimension of the management process of the properties. And some of those stakeholders, of landowners, are located in the Santa Lucia basin. So what has more to do with this national scale view of the need to promote private initiatives and incorporate conservation in the way the productive properties are being managed. So, our activities right now have more to do with that. It's a national scale issue that has some participation of landowners in the Santa Lucia area. And within that, one of the things we are doing is trying to promote experiences in restoration activities. So, a couple of years ago, we got funding from the Nature Conservancy to do some experiences trying to recover riparian forests and one of those properties. I think our basic goal is to develop experiences that can be then replicated in other sites and more than a strategic vision of what we want to do in the Santa Lucia basin.

Interviewer 1: Do you have any knowledge of the implementation of riparian buffer zones in the Santa Lucia River basin as a part of the action plan?

Interviewee: I know there are some ongoing initiatives and I'm not aware of how effective they have been or how extensive they have been.

Interviewer 1: Do you know perhaps what the current functions of these buffer zones are in the river basin?

Interviewee: Let me go one step backward. What are you referring to when you talk about the buffer zones? Because it's, at the end a buffer zone is an area around a line, so it's, so what are you meaning when you ask me this?

Interviewer 1: No, of course, measure eight in the Santa Lucia River basin action plan is the implementation of riparian buffer zones along the water courses of the tributaries of the water of the Santa Lucia River. These buffer zones are strips of vegetation that can consist of different types of species that can either be trees or grassy vegetation, can either be fenced or not fenced, and can either be, for instance, productive or non-productive in the sense that cattle can access these buffers or not, and their initial purpose was to catch and retain the nutrients that run off from agricultural land so that they do not end up in the water course. Do you have any experience with these buffer zones? And if so, what functions other than nutrient retention do you think they have?

Interviewee: Again that can be a more general question or a very specific one. Concerning that measure of the action plan, we haven't been involved in any initiative aimed at fulfilling that action in the line with the plan, regarding the need to promote the recovery of native ecosystems in areas around riparian zones. We do have some experience. We do think that areas have a lot of ecological functions and sociological functions that go way beyond just retaining the extra input of nutrients from agricultural activities and have to deal with maintaining a range of other ecosystem services, patches for biodiversity systems, and enjoyment. I mean, they are patches of native ecosystems that allow a very wide range of ecosystem services and ecosystem services are always depending on what the specific stakeholders are expecting from them, or are needing or are using from those patches of nature. And then there's a huge range of views of what they can be useful for. I do understand that the main reason for the government to push forward this initiative had to do with water quality, but as I said, before that, there was already a protected area that needed management to increase biodiversity persistence and maintain the long-term ecosystem services and human well-being in that area and, so restoring patches of the natural ecosystem within that protected area has a lot of different contributions to a range of needs. That's what I mean. So, if your question is what are those needs and what they provide, the least is very long. I will say a few things that come to my mind directly in this conversation, but I will not remember most of them, so I don't know if I can answer the question you want or whether I've done it, I don't know.

Interviewer 1: You already did, since you mentioned that there are several ecosystem services that they can provide, that they increase the biodiversity persistence, that they can provide enjoyment or aesthetic values, for instance, and that they indeed contribute to water quality improvement.

Interviewee: And what I would say is that ecosystem services, it's a concept that it's meaningful as long as it also recognises that different stakeholders obtain different things from nature. So it also depends on the views, needs, and values of the stakeholders. So what I do understand is that the actual plan is focused on one issue, which is water quality, but that plan, as I said, comes after a more general and overarching plan that is the management of the area as a protected area within a larger national system of protected areas that is developed to provide proper management to certain areas of the country that are valuable because of what they do in terms of maintaining certain ecological processes that are key for human well-being in a sociological system. Do you know what I mean? The plan is part of a lot of other initiatives that are superimposed and that have local and national scale objectives or aims.

Interviewer 1: Yes, and regarding the view of Vida Silvestre, what characteristics of these protected areas, so to say, or buffer zones in this case, are important? Is it the conservation of native species, for instance, or are there other things that play a part in it?

Interviewee: The buffer zones are tiny strips of land within a larger protected area because the whole of Santa Lucia is a huge area in terms of what the protected areas are in Uruguay. If you're talking about that tiny strip, again, what was the question?

Interviewer 1: What characteristics are important concerning the view of Vida Silvestre? What characteristics should those buffers have?

Interviewee: Well, they need to resemble as much as possible the native ecosystems that were there. We do understand that once you introduce changes in a landscape, you are not able to return to the regional conditions, but what you do is leave natural ecological processes to restore natural ecological systems, enabling the recovery of the composition and structure of those ecosystems. But it doesn't mean reintroducing an ecosystem with the same conditions because those conditions have changed both locally but also around. So you are managing an adaptive system that it currently is, and what we are trying to do is to restore some of its original conditions, but it won't be the same because the system is not the same. But you do want to, in general, contribute to maintaining the same processes and services when you see it from a human perspective that were there before you remove that ecosystem. And so, as we are talking about an ecosystem, we are not just talking about the floristic characteristics, but the whole range of interactions among species from different fields, I mean from the different ranges of species you will find there; plants and animals and fungi and other components of diversity. So we don't see just the forest and the function of retaining nutrients, we understand we need to recover the original native ecosystem as much as possible, but also, again, the buffer zone is just a tiny strip of a larger landscape, you need to manage to maintain the provision of services and the conditions for natural ecosystems to evolve and respond to changes.

Interviewer 1: And how do you think that management can be done in the best way?

Interviewee: It's a very general question, I suppose. Yeah, well, that's the reason why we propose creating a protected area. A protected area is an area that has a specific set of rules that has a goal in mind, the sustainable development of that region in a manner that articulates private interests and enables productive activities, but it also enables the protection of certain areas within that area, that overall ensure that certain sites are key to maintain ecological processes at the scale of the whole area. So that these areas are maintained and protected from some of those productive activities. So there is not one single answer to your question, what we think is that you need to manage the landscape in an integrated way, put in biodiversity conservation and ecosystem service maintenance as a goal that has to be achieved as well as other productive objectives, taking into account both private initiatives and interests and collective initiatives. So it's the challenge of sustainable development, it's just you have an area in which you have specific rules that enables you to have those dialogues to find which action should be promoted and which action should be not promoted in different sites of the area. Because not all the area is homogenous, it is a very general question. Regarding the buffer zones, the key issue is to enable the recovery of the original structure preferably with the same or with a similar composition of species, but in some areas, you may need different strategies as you have productive activities producing an impact. So you may need to also reinforce those functions and somehow counteract the effects of the productive activities that have been conducted next to the buffer zone. So again it's not a simple question, it depends on where. And the main gist is to treat the entire area as a protected area as it is right now and it should be managed in an integrated way that takes into account the productive aspects of the producers and also the nature conservation aspects of the native species.

Interviewer 1: What benefits do you think the system would gain from this kind of integrated management?

Interviewee: Actually, you go from a single objective, you change the kind of function you want to optimise rather than trying to maximise individual gains with regards to one dimension of development, you go into a multiple objective goal of integrating interests from different stakeholders and trying to maximise gain in a range of dimension of development. So it's a much more complicated scenario on which you have to take decisions, but the whole idea of going from individual isolated management of land into an integrated management of land that takes into account the different kinds of interests and needs. I mean what we understand is needed if we are going to be able to maintain healthy landscapes in a productive country as to what we have. I'm not sure if I'm answering your question but the question seems simple but it's not.

Interviewer 1: No all these questions are quite complicated because the discussion we're having goes from the original purpose of buffer zones to a higher dimension of protected areas.

Interviewee: And for us, a protected area is basically another thing that's interesting because for us a protected area is a productive landscape that is managed in a way that enables economic development, social development but also ecological resilience and nature conservancy. So all those objectives have to be put together and weighed and at the same time taking into consideration the specific individual needs of the landowners and the collective interests and needs of the country, or at least the people from the three departments that are involved with the management of the area. The departments in terms of administrative regions of that are trying to maximise short-term gains but also long-term gains. So what we do is by creating a protected area where we are forcing ourselves to think in this multidimensional way and that's the challenge. Because protected areas were promoted with one specific goal in mind but that promotion came after a more general decision of promoting nature conservancy in the protected area and then ecosystem restoration and another intervention on the landscape to promote resilience in the landscape. And so it's the question of the buffer zones in the camps, there are some decisions and some visions that were already set there when the government added another layer and said we need to restore buffer zones to maintain water quality, but there were already discussions on what interventions we need to do in that landscape to enable the production but also the health of the landscape. So for me, it's very difficult to just think and answer in terms of the specific buffer zones, 200 metres or 100 metres around the river.

Interviewer 1: Of course and maybe then it's better to look at it from your perspective. And what do you think are the main obstacles with the management of such a protected area in regards to either the social processes or the physical management?

Interviewee: I think the social process is much more complicated than the physical one. I will give you a specific answer regarding the buffer zone and then a more general one regarding the area in general, we don't have good experiences in terms of recovering ecosystems, so we've been trying to do a range of initiatives without proper research and proper testing and long-term funding so the easiest part should be restoring the ecosystem. And I'm not saying that it is easy but I think it is the easiest, we are not doing that right because we don't have experience and we haven't taken the decision of investing in research and experiences that can enable us to do this properly, but that's one issue. Then the challenge of an integrated management of a region of an area of the size of Santa Lucia; it's a typical wicked problem that needs to integrate views, so it has all the challenges of a wicked problem and you know how a wicked problem has to be managed. And that's one of the advantages of the protected area approach, protected areas use adaptive management as a method of the management approach, but in order to do that you need amongst other things, institutions that can take decisions and adaptively change the decisions. And we have institutions that are more used to a traditional management approach, so you need to also change the way the institutions take decisions and monitor the decisions and modify the decisions based on the evidence they generate. That's one issue from a more institutional perspective. But then again a range of stakeholders seeing the problem from different perspectives expecting different things from that landscape with different, how would you say, competence in terms of being the ones that have the right to take the decision so it's just at a smaller scale; the challenge of managing a country or a rainy piece of land when you integrate several persons and several stakeholders so I wouldn't be able to say what's the most difficult part of that. If you try to make this simple and think okay this is a sociological system, the social component of the sociological system is a mess, we don't know how to manage that properly, we don't know how to agree, how to build consensus and that's where the challenge is. We don't have experience in ecosystem management but we can gain that and there's a lot of experience elsewhere we can put to work here. But the social subsystem is a complicated one, a very complicated one.

Interviewer 1: That's also what we found in our research so far, that there's a very large variety of different visions on for instance the management or the involvement of certain actors, between actors, and even within stakeholder groups there's like a very large variety of visions so it's very difficult to find a common denominator in that.

Interviewee: But I think if I have something specific to say in this conversation is that we need to understand this problem as a wicked problem and wicked problems have very specific ways of being approached, with adaptive management being a key tool. But adaptive management it's not widely incorporated into the way we manage the landscape and countries. It's a way of managing, of taking decisions that understand that there is uncertainty, that understands there are values in conflict, and that then it plans, it acts, it learns, it changes and plans again and acts again. And that approach involves not only the actions focused on the biophysical part of the system but also in the social part of the system, so we need to manage this social complexity also in an adaptive way and understand that different stakeholders have different values that manage differently uncertainties that are perceived differently. The uncertainties that are present in the system and hence the challenge is in that part of the drawing in the social aspect and that you won't get the necessary consensus, and that you won't solve this problem that you would just manage it in a way that harms you the least possibly. You navigate a permanent problem, it's not that you solve a problem and move away from that and that there is not one single solution. What I mean is that for me the problem is that we are not seeing the problem or approaching the problem properly, because we are thinking of the problem as a more trained problem that has technical solutions and we are in a different arena, in a different scenario. And again there is a lot to gain from the wicked problem literature on adaptive management as a useful approach to handling this kind of problem.

Interviewer 1: And as you said before it's like an iterative process, right? You implement it, you monitor it, you evaluate it, you adjust it, you assess it, and you plan again. Then you implement it again. It's like a cycle. How do you see the implementation of such a management strategy?

Interviewee: Well that's why we created a protected area. That's how protected areas are managed.

Interviewer 1: Okay perfect.

Interviewee: So the idea of having a protected area involves two things many things. First, it gives you some specific rules that apply to that piece of territory. You have some regulations that are different from how you have to take decisions and what you put forward in the rest of the country because protected areas are areas that have a specific set of considerations and laws that enable you to do things that elsewhere are much more difficult. That's one issue. That's one of the advantages of having protected areas in Santa Lucia, and the second one is that protected areas are managed adaptively, so they are developed to handle this kind of problem. The problem is that most of the stakeholders involved do not see this in that way. The whole idea of the protected area has not been put forward appropriately. It's more like it's seen more like initiatives to protect certain pieces of native ecosystems and leading to more than that, but the whole idea of having a protected area from my point of view is to put together in that territory the tools that have been developed elsewhere to handle wicked problems through an adaptive management process. But I think no one is seeing this and that's I think, I don't know if it's a problem, it's maybe putting this forward and seeing this clearly might help to evolve to this area in a different direction. But then the first step would be to understand that there is a category of problems that are wicked problems that need to be approached differently, and I don't think the concept of a wicked problem is around in the mind of most of the people involved.

Interviewer 1: No, but now that you describe it is exactly what we see doing this research as well. We see that it's a combination of for instance communication and education problems for certain stakeholder groups, that are combined with unclear management practises in the field combined with climate change impacts that are disregarded. There are a lot of different inputs causing different problems all leading to basically the same observation in the field that buffer zones are not implemented correctly, or in this case, maybe even protected areas. I don't know on a larger scale whether that's true but it is indeed a very good perspective and it's very useful for us to take into account so thank you very much for that.

Interviewee: I can send you this one paper I use very often with my students which has to do with ecosystem management as a wicked problem, and I think you can expand that to a larger idea of sociological systems management as a wicked problem. And yeah, I can send it to you and maybe it helps.

Interviewer 1: That would be amazing if it's possible. I think that this is a topic that would be worthwhile to look into because our goal is eventually to make a policy brief for the improvement of riparian buffer zones, and from what I hear this could be a very good foundation as a way of thinking for it.

Interviewee: But yeah I'm telling you what my understanding of the challenge we have is. I mean beyond buffer zones and Santa Lucia, for me it's the challenge of properly managing and regulating the country's landscape, bearing in mind that we live in a production-focussed country. So we need to find a way to integrate those. Well in that sense it's not a different example from the Netherlands. We need to actively manage the whole landscape and it's simply we promote different goals in different specific sites to be able to maintain a multifunctional landscape that provides resilience, both for the social system and also for the ecological infrastructure that is needed to maintain that social system. And from that perspective for us, protected areas are a tool that enables us in certain parts of the country to do certain things that are needed to properly articulate the needs of the population there and the needs of the ecological systems to keep on functioning properly, and that's what for us are protected areas. And if they work and if we have good examples to tell on how we manage the biophysical system but also how we should work the discussions and the dreaming in the social system, then we can explore that idea with the rest of the country and actually manage the country in general from that point of view with this approach. So somehow institutions of sustainable management of the landscape are just in the protected areas, you have certain regulations and certain laws that enable you to do that and then you cannot do it outside right now. And also you can put money and effort to make those examples, those specific sites, to work, and then as they work you can show; okay we can do the same elsewhere. That's the way we see it always, and within that, the buffer zone is a specific issue. Interviewer 1: Yes I understand. At the moment I don't have any other questions. Do you have any questions for me in that regard?

Interviewee: No.

Interviewer 1: So I think that for this interview we're done. I think that your inputs are very valuable for research, especially in regards to the bigger picture vision that should be or could be handled with the management of these buffer zones for instance. For my research, the next step will be to process the results of all the interviews and to eventually come up with a policy brief that if you're interested then we can share with you to see if you have any feedback on it if you want.

Interviewee: I will be happy, I would like to. Yes, definitely. I will send you a couple of articles and papers, and if you find there's something else you would like to see or discuss or whatever just let me know, just send me an email or text me or whatever.

Interviewer 1: Okay of course thank you so much.

Interviewee: You're welcome, bye bye.

Interviewer 1: Good bye have a good day.

*Interviewer 1 is the student, and interviewer 2 is the supervisor.*

# Interview 21

**Interviewee: DINOT    Date: 23-03-2023**

Interviewer 1: What do you do, and what organisation do you represent.

Interviewee: Well, I am an architect with a specialty, a master's degree in territorial planning and urban development from the University of the Republic of Uruguay. And, well, I have been working for quite some time in territorial planning in a broad sense because, well, territorial planning is very broad and, well, I have experience in the design and development of territorial plans; urban planning, and territorial planning in general on regional or departmental scales. Then I was director, in the public sphere I was director of territorial planning and territorial management of a department which is Canelones, which is a department of Uruguay. And then also in the public sphere, I was the national director of territorial planning in the Ministry of Housing, territorial planning, and the environment (MVOTMA). The national directory of land-use planning is one of the executing units of the Ministry, which is now a ministry that has been divided into two parts. So now land-use planning is separate, before it was also with the environment, which also had a special significance for your specific topic at that time and well. Perhaps now with the change, the ministry has less daily contact with the environmental area, and the planning of the environmental area has naturally a necessary function. Having been in the same ministry at that time allowed us to do very deep, interesting work that we can talk about now. Currently, I work as the director of a park, the largest public park in Uruguay, which is Roosevelt Park. That is my current job. I am not in a different job, although in terms it has a part of my profile, it is a different management job. It is for the intention. But I also work in the private sector. I am part of a non-governmental situation called IVI and we do consultancies. The focus of IVI has to do with sustainable development and the transition towards a future with better levels of resilience and adaptation to climate change, that is a little bit the idea of IVI. For example, in the field of advising the public sector, and international cooperation; it is an NGO. And well, I'm also a lecturer at the University of the Republic and a lecturer at ORT University.

Interviewer 1: And what is your relationship with the buffer zones in the Santa Lucia River Basin?

Interviewee: Well, in my previous job in the municipality of Canelones, we worked on land use plans as a planning director. At that time, from 2011 to 2015, Uruguay started to use buffer zones as a management tool for the water system. Not only in terms of water quality, but also in terms of regulating runoff and flooding, so it was a very integrated thing. We discussed buffer zones as ecologically necessary space for the quality and functioning of the watercourse. That was in the context of flooding at that time. Later, already in the Ministry of Housing, Spatial Planning and the Environment, we started to deal with the Santa Lucía Basin, because events had occurred that marked a long time in Uruguay. There were problems with drinking water and we were looking for the causes and possible causes of the floods. We were looking for the causes and possible strategies to solve this problem. One of the functions of the Santa Lucía basin is to channel water to the drinking water treatment plant, which supplies drinking water to the whole of Montevideo and a large part of the metropolitan area, almost half of the population. Therefore, after these events, the focus was put on what happens to the water of the Santa Lucía River, where the problems are and how they can be solved. And in my opinion, there has been an interesting development, but not at the speed required for the problem: namely, the understanding of the river not as a watercourse, but as an area integrated by different productive activities of land without productive use or destination, without intensive industrial activity. The urban areas are also part of the Santa Lucia basin and its tributaries, so all this is a territorial system. So we can see the basin at this depth, and this is the point where the tool of buffer zones appears on the river. The area where the river course connects or intersects with the rest of the territory and the rest of the activities of the territory. How they face the different situations and how this buffer zone can actually be a tool to find a solution to some of the problems. I personally led a very interesting project called Atlas de la Cuenca de la Río de Santa Lucía, which I don't know if you know about. It was a project of the Dirección Nacional de Ordenamiento, the main purpose of which was to show all the technicians who were working on the issue, together with other technical teams who weren't involved in thinking about the water problem in the Santa Lucía River, that the Santa Lucía basin isn't just a mess that we have to regulate technically, but that there is also an economy upstream. There is a society that has a long history and productive activities that have also left their mark and culture. In short, to deal with the water problem in the Santa Lucía basin, we need to understand that it's not just a mess where we need to sort out some technical issues. To get a grip on the water problem, we need a profound vision with a vision of spatial planning and linking it to the development of productive activities. It also needs to be a strategic productive framework, it's not just about technical measures. That was part of the idea of the Atlas, to show a zero point for this work and compare it with the new generation measures, after the 2013-2015 measures. The first generation was in a way a set of measures that aimed to address the problems with a good goal, but they weren't necessarily connected. But there was a slightly deeper look at the area you were dealing with, what was the land activity, what was the land use, what were the cities, where were these cities going, what was the role of these cities in the urban plan, what was the metropolitan system. This is a set of things that were very important to look for sustainable and workable solutions that would be more suitable over time. So this new generation of policies not only included an update of the provisions that were already from the first set of policies, but also introduced the issue of land use planning and started a series of policies to link urban plans with departmental strategies and to develop a regional strategy for the Santa Lucia Basin, which is one of the land use planning tools in Uruguay, although it's not approved. Because it wasn't examined in the previous period and now in this government, it's still there to be approved. It was a problem that no national land use planning was decided for the sustainable development of the Santa Lucía Basin. I don't know if you know this, but it led to a programme called the National Land Use Planning Programme for the Santa Lucía Basin. That was an instrument that was initiated within the framework of these second generation measures by the Council of Ministers in an area called the National Committee for Spatial Planning, which is made up of the ministries that have to do with the territory, industry, transport, the environment, spatial planning and OPP, as well as the congresses of mayors. That's where this national programme was created, and I mention it because it's good that they're involved. I've some presentations of the things that were worked out and I think it was a very rich committee because the whole environmental sector was represented there, but also the whole field of irrigation dynamics, the speakers from spatial planning, the Ministry of Livestock and also the Ministry of Industry with a technician. The aim of this national programme was to develop strategic guidelines and a set of definitions for this type of planning, which will then be linked to the actions that can be carried out in the municipalities, ministries and others. In doing so, we worked on an innovation of looking at the territory as a whole and bringing many dimensions to the study table that hadn't been considered before. We tried to create a map that we called the "Map of Preferential Use Areas". In a way, as a tool to guide land use with a focus on the objectives of the programme, which is the quality of water for human consumption, but also the protection of the river ecosystem. So with this focus we have tried to define areas of preferential use. When we talk about preferential use areas, it means that we map the area, i.e. what is the destination of this place? What is the strategic orientation? And sometimes it turns out to be yes or no in that place. So the Ministry of Economy's support programme plans, the Ministry of Livestock's land use plans and the Intendencia's territorial plans were coherent in that sense. And we worked very well, and I'd like to highlight that, because we identified the dairy farms in the catchment area, where the dairy farms were causing a problem, and how to proceed there. And we identified a number of measures to act in accordance with the second generation of measures. But most importantly, the so-called preferred zero land use area was identified, which is the area where the ecosystem of the river should be protected above all. So the 30-metre and 20-metre buffer zone was changed into another area, which was, let's say, a bit wider than the smaller strip of one metre, when the different conditions of soil, geomorphology and historical use were clarified. And that is a zero-use area where the conservation area was established. I don't remember if that's exactly what the text said, but I'll submit it later. It was a preferential use area, the Santa Lucia River Water Quality and Ecosystem Protection Area.

Interviewer 2: Do you remember it was a bit cut off but we pursued the idea. Do you remember where the priority areas were? Which part of the basin was that? I guess from the upstream sub-basin, but I don't know.

Interviewee: I imagine it with an area planner, this preferential area. I'll open a map, no, it would be great to open a map. Let me see if I've something here, because I think it's much clearer if you can see it on a map. Let me check as we go. Yeah, it's not there, I can tell you that. Also, there is a difference in the definition in which the programme worked, because in the first line of upstream water measures, it worked from the mouth of the river into the La Plata River. But there is no separation. In the research I separate what is upstream from the rest, and that wasn't done, the policy didn't make that separation. If this is zone A, it was zone B, if I remember correctly. In other words, the wetlands in the protected area and so on are important. Of course, but for drinking water it's right that it has less impact. From a more ecosystem perspective, it would be very difficult not to have definitions for that like I've here.

Interviewer 2: Before I go, one more question: you had two experiences: the experience of the municipality of Canelones, which is committed to sustainable development, which I don't see in any other municipality. At least as far as I know, there could be a vocation for it in Rocha and at the national level. That is, we also know that there are two ways to implement, for example, buffer zones: one is through the environmental law, as was the case with the Santa Lucia Action Plan, and the other is through departmental land use planning. Can you briefly summarise for me the advantages of the two ways, one through the municipality and the other through the general law, for example, in terms of control and implementation? Sorry for not being there.

Interviewee: The main advantage is that the two ways aren't complementary, but I'll tell you a little bit about the responsibilities now. The parliamentary governments are responsible for regulating land use in their areas, and they're definitely the ones who directly determine what is allowed and what isn't allowed in the departmental area and with what occupation factors or with what intensity of land use activities can be carried out. These are the parliamentary governments, and for this they have instruments that are laid down in the law, namely the departmental guidelines and the local plans. On the other hand, responsibilities, especially in rural areas, are somewhat distributed by the presence of the Ministry of Livestock. In the case of use and management plans, which also play an important role, there is a very strong national framework in which the entire environmental sector of the country emerges, which is the dynamic of DINACEA and DINAGUA. The National Water Directorate and the Environment Directorate have also made the protection of ecosystems and watercourses their missions. That is a constitutional task, and the laws that these national institutions have created So there is an overlap of responsibilities. Even the Atlas questions this and has a separate chapter on it, trying to show that there are complementary and, in some cases, overlapping responsibilities and that there is a problem when they conflict. So my vision is that national programmes are necessary to define strategic orientations based on concrete actions but then implemented in a coherent, regional approach through the responsibilities of each national body and through the responsibilities of the ministries. This is why we liked the idea of working within the framework of national strategies and national spatial planning programmes. This is where the intention of the national programme comes from. So I think that for the traditional regulation of land use, departmental instruments are much more effective, but there are national responsibilities that the departmental instrument mustn't contradict, because otherwise it would be ineffective. So we need a cross-departmental instrument that at least defines a strategic working basis that prevents contradictions. This is where regional spatial planning instruments or national spatial planning programmes come into play.

Interviewer 2: And as far as control is concerned, you said something about implementing the departmental scale instruments, for example, but as far as control is concerned, is there any kind of possibility of control? Or is there a control mechanism for compliance? That seems to me to be outside the scope of national policy. What control tools and mechanisms do you have?

Interviewee: The Planning Act creates the idea of territorial policing, which gives authorities the power to set up territorial policing precisely for planning instruments. It's clearly a great tool that is little used, but it seems to be a very necessary tool that needs to be complemented, as I said today, by the control of the use and management plans, which is the first time in Uruguay. And also the territorial structure for the control of protected areas and DINACEA is still very weak in this respect. In Uruguay, action is reactive and not proactive, people react to complaints, whereas it's necessary to act proactively. Inter-institutional coordination is essential. If you want, I can show you a presentation I gave. I don't know if you have any questions, but if not, I'll tell you a little bit about St Lucia.

Interviewer 2: No, no, with that, you have answered what I wanted. Alfred has more questions.

Interviewee: Going back to the methodology of the National Programme for the Santa Lucia Catchment, how they went about identifying. As you can see in the blue rectangle in the middle, the areas were identified for preferred use and that wasn't approved. That was very unfortunate and also shows how difficult it's to deal with things that are a bit deep, doesn't it? But they went all the way through the inter-ministerial agreement and I think that's a useful tool. Now we're not talking about the same post, because we've been working on it for a year and a half. So, well, some inputs, soil characteristics, land cover, soil types, geomorphology, biodiversity, no? We have been working on a more ecological dimension, as it says there, to identify or characterise homogeneous areas with these inputs. And we're talking about the ecological thumbnail of a set of areas, right? This is a first approximation map. Then comes the territorial dimension that I told you about in the atlas, right? The capacity, the strategic value of the Santa Lucia River basin, a set of inputs, and then the land uses, the productive activities that go along with the development, the growth, so that after that combination, we can come up with a map of the areas of preferred use. We came up with 7 areas that we had to define in a programme. After this strategic definition, the plans, the productive development programmes and even the institutional organisation could take these areas into account in their actions. And also the departmental management plans, so that there is coherence between the southern part of Santa Lucia and the northern part. For Canelones and Florída, things that are obvious, we needed an interdepartmental instrument that would give them coherence. So an Area 1 was defined, which I am going to tell you about now, and which you might imagine very well. And I will also talk to somebody else because he was very involved in it. That is the area that is focused on the conservation of natural resources and maintaining their ecological functionality for the conservation of water, for the provisioning of the population, and as a source of ecosystem services. So, now we will see.

Interviewer 2: What did they come up with?

Interviewee: That would be so; that's the yellow. So you are starting to see, right? It's not a continuous buffer of 50, 20, or 30 feet anymore, right? It adjusts to the topography and the flood pressure of the river. It's obviously the definition of the river, not the definition of the river.

Interviewer 2: You are talking about a variable buffer zone?

Interviewee: Yes, of course, including some of the streams, the places where it was deemed necessary to protect them. Because that's where they provide the amount of fresh water that then also protect places, not only the plains, but also higher places where the river water is supplied in a way, also gives some sense of protection.

Interviewer 2: And in terms of implementation mechanisms, you can see that there is enough space over there for the buffers. But there it's on productive land. How did you implement it on that land with the producers?

Interviewee: Yes, so, I see a line of work here that is being reviewed as opposed to the buffer zone, the immediate area, the area of maximum protection that prevents any kind of activity. The programme has challenged that in some ways, so progress has been made in terms of what actions are compatible with that area of preferred use.

Interviewer 2: How can that area continue to protect the natural resource and its ecological functionality, but still have a productive function?

Interviewee: And there was a particular line of work that was directed towards that, you know, that did not have, let us say, an end product, but it was one of the actions that was derived from that. A number of actions were derived from that, including this one. It was about promoting a productive programme to make it viable. So there were several hypotheses with the secret of livestock, with most environmental engineers and agricultural engineers to figure that out, right? But there was never any talk of a reduced instrument to implement, because I can imagine that there is a case, for example, that I have in Flores, when the idea of a protected forest was implemented, that this would be something in between, between a protected area and what you want to do. Today there is a conflict because people want to use their land freely, right? In other words, it's something that needs to be ingrained in the culture of the producers, right? They thought of some kind of instrument to bring the producers more down to earth, so that there is a strong change, let us say. And the basic tool is the use and management plans, right? That is, for all lands that are identified as ecologically vulnerable, there needs to be a use and management plan, similar to what we have already done, beyond the size of the land. We have been working with this idea of a use and management plan and trying to align the areas that are impacted by this plan so that they still serve a productive purpose. It would still have a productive purpose, but within the context of a use and management plan for that land with its own activities. And then there are the incentives, right? I used to live here, and I saw that there was this presentation that I do not remember, right? Because they say, well, preferential use area, right? Here I said compatibility, adaptability with incompatibility conditions. That is, everybody does the work of the basin, for each preferred use area, they determine the compatibilities and which ones can be, which ones can be compatible with conditions, because there's white, black, then grey, right? Depending on the current activities they have identified, right? Current activities in the preferential use area. Well, which of these can be compatible. And for that, criteria for management and coordination for regulatory management are needed. And then, I show you the other slide which is, well, in the framework of this programme we say that, on the one hand, the tools, the plans are the ones that are going to limit and promote the activities in that territory, orienting the productive activities. And this is where the incentives appear, the promotion of incentives by the ministry of economy, of the different economic incentives that always exist. And obviously, once you have the approved instrument, you have to agree to finish implementing the limitations, to finish implementing the location of the productive activities through the plans. But, on the other hand, you also have to finish orienting the livestock programmes, the economic incentive programmes, and the promotion of productive activities. The environmental permits also appear.

Interviewer 2: Yes, yes, I understand.

Interviewee: It is a transversal policy because I have touched on everything from the Ministry of Livestock to DINACEA, to DINOT, to the Intendencia. Of course, it is also good to say that a territorial programme that deals with land use is not sinic, isn't it? That is clear, it is not sinic, it touches interests.

Interviewer 2: Yes, well, since I'm an economist, with a background in economics, it's the first one that jumps out. I expect that they're going to complain that they're going to lose money.

Interviewee: I remember that the idea was put forward of making a kind of evaluation of the economic impacts of these measures, right? And, well, that, they had even started a project with some economists that were not completed, but it was interesting. Because, well, you don't necessarily do it when you design it and then see the impact. You have to accept that you are going to do that report not to find a zero balance, because you are not going to find it. That is a situation of economic impact that you will try to imitate it. But the economic impact for certain activities will be different because you restrict them. What happens is that you, in the economic impact, have to have sufficient integration of the economic benefits that the country has. Then, it costs in millions to make the water drinkable. Well, we have to have ecosystem services in balance so that this territory, as well as this soil, ends up pouring the water that is then made drinkable. What happens? This, with that, is decisive for the investment that you have to make afterward and to have all the economic benefits of having treated it well from a niche; otherwise, you pay for them afterward, right? So this issue is not so easy; it's not the typical impact of, well, how much does it cover me? How much does this area cover me? So I have a productive limitation on 30% of my land, and therefore my profitability dropped by 20%, right? It's like a global equation, which at a legitimate level has to be done, right? You need to account for private interests to understand the equation. To me, I always go into the issue of when they're just going to come at you, and they're going to bring you lawsuits. For example, if I own my field, and they're taking it, you have to look at the issue or implement a payment for ecosystem service, or you will be sued. Or the regulatory framework is integrated in such a way that you are not violating property rights, like the buffer zone. What I know is to throw it out, like the buffer zone. You are going to tell me the whole legal chapter of this, because the expulsions of ordinance always start with the Parliament, and this is unconstitutional because, of course, it touches interests, doesn't it? Here, the line of reasoning is the following: the planning law allows that, through the instruments of territorial planning—this is not an isolated measure; this is an instrument—one can orient and limit the use of the land of the territory. So, it seems to me that what is potentially unconstitutional is to prevent any activity from being carried out on rural land, i.e., to limit any type of activity on that rural or urban land. But to guide the activities with restrictions that are in the sense of what is in the construction with respect to the care of the environment and the care of water or resources, especially the water that will later be made drinkable, Those restrictions and limitations on land use and on the use of private property are in the construction law and the law on land use planning. So, this is the framework within which land use planning works, really generating definitions of land use and compatibilities of some activities. But obviously any planning instrument also has to think that these limitations have to be viable for productive activity, which is why the whole issue of incentive programmes is promoted. To guide them and give them viability.

Interviewer 2: But it is not illegal to prevent some activities and place restrictions on activities on the land, right?

Interviewee: But of course, this is clearly a difficult point because the history of Uruguay is a history that is marked in some way by productive activity in the development of the free will of private activity in the territory. So, well, environmental laws are from the 21st century; they are not from 50 years ago, so we are in a kind of maturation of the issue, aren't we? It is being imposed, but the legal and continental frameworks are in place, so don't come here to be judgmental because you're going to disagree.

Interviewer 2: Yes, what it may have is a political cost, at least. But I loved this integrated vision. Alfred, I'm sorry to take this time. It's great with everything you told us. I was playing devil's advocate for the instrument because I'm interested in knowing how to get down to earth about all these things about territorial planning, which are good.

Interviewee: Well, you see, the St. Lucia Basin programme didn't get approved. I'm not going to tell you on this recording what the reasons were and who put the stick in the wheel and everything. I'm going to admit that we didn't get the time to try it, but it clearly didn't get approved. I'm not going to make a general allegation, but it didn't pass.

Interviewer 2: The current government has failed to make progress, hasn't it? It would be good if you could interview the current national director of territorial planning to see how far this has progressed, right?

Interviewer 1: Yes, yes. With regard to the buffer zones, if they could be improved, how would you improve them?

Interviewee: Well, I think that in line with what I was saying, to me the buffer zone is an area of absolute restriction. For me, it has to be deconstructed a little bit to create an understanding. So let's put them there, and we understand that the buffer zone is that space of the territory where the river, as a unit, needs to be in a situation like that. I think that for me it is a mistake to think that after that the buffer zone is an immediate line; thinking of the buffer zone as that reduced space around the river, after that there is a border, and after that there is this homogeneous productive territory. To me, in the middle there is a necessary protection area, so surely the buffer zone also needs its more intensive, more traditional land uses, both urban and rural. So I think it is precisely the fact that the buffer zone is limited; that is to say, we cannot extend it because if we affect 30% of a property, 40% of it becomes unusable and the property is subject to lawsuits. However, in the buffer zones in some parts, you can do productive activities, and things can happen, it is not that it is a zone that has no profitability. Obviously, this goes for touristic, productional, and recreational activities. Then, it seems to me that the buffer zone is not something homogeneous, but that it is something that has to be studied according to the geomorphology in which it is located; the type of soil that there is there to see the kind of historical productive activity and the productive vocation of the place. I don’t know, you have to know several things about how to design a buffer area in a more complex way. It's not black and white.

Interviewer 1: And what features do you think should be changed or added to the buffer zones design to support this change of function?

Interviewee: For the design, well, regarding the framework, there is a lot of analysis input to, justly, redesign the buffer zones. We follow those who were there, which I told you, that I am going to pass on in the presentation with those slides, which justly corporate all the geomorphological and ecosystem issues, so it can be both productive and also socioeconomic. There is a series of new information that you have to gather for the redesign of the buffer zones. And well, I think that there obviously is an activity of a technical component, that the biggest problem is that it is only an analysis of environmental technicians and that there is no incorporation of all the agronomic part, infrastructure, territorial planning, or urbanism. Because also the buffer zones are located in rural areas and in the city. We can’t interfere in the city, because that’s not possible. But I don't agree with that. I believe that the plans can work to define urban buffer zones, just like in the rural buffer area. It may involve removing housing, relocating people, or involving changing the uses of the soil of those areas and having there a linear park, and starting to define some compatible activities. And there are many examples in the country that how that has been resolved, right? The situation in Paysandú, in Durazno, but also in Canelones. Now the plan of Canelons, for example, is working on that situation, in full capacity. That is, different plans that have incorporated the urban buffer zone with a tool also of action for water courses, right?

Interviewer 1: What obstacles do you think will be experienced with the implementation of these changes to the buffer zones?

Interviewee: And well, I think that the obstacle has to do with how it has to be incorporated into a complex public institutionality, because many actors are involved with the issue, so articulating that is very difficult. That's why they are that using instruments that have legal validation in such a way that they are binding with the policies, although it is not enough with the political will. There must be approved instruments that are binding with the policies so that it is not enough with the laws and the instruments for things to happen. Of course, the articulation is needed, but you have to be bound by some norm, by some instrumental framework. Because if not, things end up being weak due to the situation. You have to overcome that situation, that on the one hand, that watercourses and their buffer zones, one has to refer to local governments, municipalities, departmental governments, various ministries and also commissions for public-private integration or social integration, such as basin commissions, rural development commissions. You have to overcome that complex institutionality. Look in the atlas because there is a specific chapter that seeks precisely to put all that complexity on the table. And that is one aspect. The other seems to me that is what we talked about today, that is, when the measures end up affecting the private land in Uruguay. There is a story of what we are talking about now, there’s a history of this; the use without limitations of the rural productive soil. Regulatory bodies have never been in a position to restrict, although there are examples, right? During the twentieth century these policies, which in a way put forward principles that are tangible, but also are a bit intangible, of general interest, raise limitations to the free use of private property. Then, here is the main obstacle that appears is damage compensation, completely out of time of another time, but that today in Uruguay is very present. Every time one poses limitations, even if they are general environmental objectives that are considered by the constitution, damages compensation appears in the discussion. If you are limiting me, compensate me, because you are restricting all my free use and my economic possibility. Therefore, there is harm without harm. If I have to compensate, it is unreal and has no support. At this time there is no legal support, And well, that’s why there is culture and it’s a big obstacle to the advance of this because ultimately, those obstacles then end up being political. And ultimately, they end up affecting these political issues.

Interviewer 1: And I have one last question. How do you think these obstacles can be overcome?

Interviewee: Well, I think that, this, with time, with the evolution of things, its difficult. That’s why I worry, and now I ask you if they had consulted you to the Ministry of Environment or the Department of Planning, if he had advanced in the instruments for the basin of Santa Lucía, right? If you did not, I recommend that you do it, because how it is overcome, is that it becomes a public policy that, regardless of how the parties alternate in power, is a continuous political item, and that it evolves. And the other seems to me that there is a construction of an agenda that appears the education and social organisations. The priority of the topics, the dissemination of the problem, the understanding of citizenship, of the ecological state, of understanding of how it affects our territory, and that the drinking water is not endless. But that there is a whole system that ends. One example was the road that passes through the west of Florida. The route that was built there and some activities connected to the route were allowed, the route was actually an artery to those productive activities. I am left with one thing to say. In the end, the problem is that understanding all these lines of causes and consequences is not necessarily direct. But yes, if one can connect them, that is where the need to have an integrated perspective appears. In this case, at the level of the river basin, or at the socio-territorial level. If you want to say it in some way, the need is precisely for learning, in the deepest sense, to incorporate people to defend and fight fairly to protect water quality, which is not just investing in carbon for the water treatment plant, it is much more than that. There is one more ecological viewpoint necessary in these things, right? It is not, it is not only with technology that it is solved, because if it's not like running a problem you're never going to reach, isn't it? That is, that's more and more present. I work in a park and we work with that idea and we try that we have a programme for children and young people who, who understand this look of ecology and ecosystems, isn't it? For the contribution of an agenda, which ultimately implies the construction of a culture that incorporates this systemic and ecological perspective, especially of the water programme at multilevel; at the academic level, of schools, of the educational cycle, but also of the organisations, of the guard commissions, of the social commissions, the areas of the productive commissions, perhaps that is super necessary.

Interviewer 2: Thank you so much. I have to go, the truth is that I appreciate what you talked about here and I thank you for your vision. I didn't know about that territorial planning project. I think it's a socioecological vision from the point of view of understanding the framework and not just one or the other. So good, nothing else.

Interviewee: Fine, okay. Perfect.

Interviewer 1: Thank you so much.

*Interviewer 1 is the student, and interviewer 2 is the supervisor.*

# Interview 22

**Interviewee: Scientist            Date: 23-03-2023**

Interviewer 1: What do you do for a living, and how are you involved with or what experience do you have with riparian buffers?

Interviewee: Okay, so now I am a professor at the university here in Uruguay, in Maldonado, and I am also working as an advisor to the United Nations as the resident coordinator in Uruguay, and I work mostly on issues of ecosystem transitions, the academic part, and also societal transitions to sustainability, more or less. So ecosystem states are also mostly about terrestrial ecosystems, and in particular, I've been working on the grassland-forest transition. That's what I did my thesis on. So yeah, that's it, and I've been involved with issues related to the role of forests in grasslands from an academic perspective. So the role or distribution of forests in grasslands and their implications in terms of ecological and societal implications And I've been working on that from the academic perspective in my thesis, and then some work on modelling the distribution of forests and doing an implication for ecological restoration and rewilding, and also looking here in Uruguay but also in the tropics. And more recently, I've been working in the central Anatolia region. So I'm a little bit looking at why there are forests in grasslands or not and what that means for different ecosystem services, benefits to people, etc. And then, at some point, I was working with the IUCN on forest landscape restoration, more or less—not a lot, but I've been involved with people since then. And then, more recently, I was working with the Ministry of Environment on a strategy for the restoration of a water dam that provides water in the main capital of Uruguay and where most of the people live, called Paso Severino, and we did some restoration activities there. So that's my practical experience; that would be mostly that aspect of trying to plant trees in the riparian zone of a reservoir.

Interviewer 1: And in that regard, what were your responsibilities and objectives for these projects?

Interviewee: I was kind of an advisor to the natural director of environment, and I was kind of overseeing that strategy. I was working with the teams in the ministry. By the way, there's an article in a publication on water quality that I can send to you. We need some publication about that.

Interviewer 1: That would be lovely.

Interviewee: It’s about the dissemination of the experience. It's not very thorough.

Interviewer 1: Okay. This buffer zone can be designed in many different ways, for instance, with different types of land cover or with different management strategies. And therefore, there are different ways to characterise these buffer zones. What functions do you think riparian buffer zones currently have in the Santa Lucia River Basin?

Interviewee: Well, the main function would be, as agreed at the Ministry, to protect the quality of the water by acting as a buffer or filter to catch runoff from agriculture. So that's the idea, and there have been some studies done to assess their performance as filters for filtering environmental runoff. And that's the main issue. And then you have, I think, more of a secondary issue, which is biodiversity and natural corridors. But in general, the first service would be more appreciated, or it would be the main goal that most of the national plans are after. And I must say that there are two types of intervention. So there's an intervention that's required, but, you know, there's a plan for the basin, there are measurements, and it's called the Santa Lucia River Action Plan. So the first measure is no labour or tillage, so no agricultural activity in those areas. And then there's the second intervention, which is to actively restore or rewild some of the areas.

Interviewer 1: Okay. And do they serve any other functions other than those that are specified in the legal documents?

Interviewee: Well, like, do you ask me if I see other functions or if the authorities do? Well, I see a lot of functions, but, like, I think these are one of the most useful things you can do in terms of a myriad of ecosystem services that go from biodiversity to carbon sequestration to, I don't know, protected river banks, etc. But also, an important function that I see is that once you plant trees, you reclaim the land from agriculture. So they are like, you know, a natural limit to agriculture, to certain types of agricultural activity, because then you have the whole issue of livestock getting into the water courses, and that is not easy to solve because, basically, when you don't want livestock getting into the river, it's very difficult. And even when there is forest, there is livestock. So yeah, I think that, but yeah, more or less.

Interviewer 1: And what characteristics or physical aspects of these buffers support these different functions?

Interviewee: Oh, that's a difficult question to answer. I wouldn't know how to, I mean, different, I don't know, there are structural aspects, community composition, soil interactions with soil. Can you be more specific?

Interviewer 1: Of course. For instance, the first thing you mentioned was nutrient retention in agricultural fields. Are there specific characteristics in buffer zones regarding types of vegetation or management practises, for instance, that are mainly used currently in the basin?

Interviewee: Well, I think that there's not enough knowledge about that. Because there's a discussion about whether you know that Uruguay has grasslands as its main kind of biome. So basically, there's a discussion on how efficient forests are versus grasslands, and it depends a lot on, for example, the age of the forest and the state of conservation, as well as, for example, the understory vegetation. So there are a lot of issues, and at some point, I think that it's a mistake to only approach the issue through one variable, like water retention. At some point, what you want is ecological integrity, and then you start looking at different reasons for having different ecosystems in different places. So there's no single answer, and I think it would be wrong to say, "Okay, we are going to build the forest that filters more water alone. You also need to look at ecological integrity, which are the forests that kind of belong there and the surrounding vegetation, so you need to have some principles of restoration and also look at other dimensions of biodiversity, things like that. But I wouldn't know what to say regarding which specific forest formation is better for a filter in water; honestly, I don't know. What I know is that it's not solved the question in any way because, for example, pasture can be a good filter, and the other thing is that, due to different temporal dynamics, at some point, things will get to the point where the capacity to store nutrients or remove nutrients is kind of saturated. So it also depends on the stage of growth of the different ecosystems.

Interviewer 1: And do you think that it would be beneficial for the implementation of these buffer zones if the policy were changed in a way that looked beyond just nutrient retention and also looked at, for instance, these other functions? And regarding these dimensions or functions that go beyond solely nutrient retention, which ones do you think are most relevant for such a policy?

Interviewee: Well, biodiversity is one, for sure, and then it depends on the system because, for example, in some places there could be stabilisation of riverbanks. But mostly, I would say that you need to take into account surrounding expectations, compatibilities with uses in the surrounding areas, or at least social acceptance, to say it in a broader way.

Interviewer 1: And does that also include, for instance, if you're implementing a buffer on the productive land of a producer, does it also include productive land use in some capacity? Or could it?

Interviewee: Well, we had this discussion at some point, and I, look, yes and no, because in a way, for example, to have perennial pastures or some sort of, do you mean only forested uses or also other types of uses? There are also other types of uses because, like you said, it's very difficult. Because there was a discussion, for example, instead of having crops, just put perennial pastures, and then you, you know, reduce the amount of work. And for me, the problem here is that we are discussing peanuts because, honestly, these are very narrow streams, like we are talking about a few metres. And in Uruguay, land is not so small, like usually you have hundreds of hectares, sometimes more, or, you know, so I am a little bit sceptical in terms of saying "we take the productive uses, like right next to the river". For me, it doesn't make sense. You first have to have some sort of natural vegetation, which could imply natural pastures in some places; another will be more forests, shrublands, or wetland vegetation. And for me, there's no question about that. But then, of course, you can have secondary buffers or buffers of buffers, which could be less intensive uses of the land. But I am against it, and there was a discussion, for example, of doing agroforestry in a field just next to the river. And I'm like, I mean, really, we are discussing peanuts, you know, and if you're talking about tens of metres, why would you be using complex solutions? Well, I could understand if you want to do like 30-metre buffers and then do a kilometre; that's fine. But if you're just talking about tens of metres, then, in my opinion, you should just go for a basically ecological ecosystem service.

Interviewer 1: Yeah. And a little bit before you said that, one of the things that should be taken into consideration with these different functions could be the surrounding expectations or the surrounding locality.

Interviewee: Yes. There are two things that are not necessarily linked to the structure of the vegetation or the type of vegetation you have or your soil cover, i.e., your land cover, which is basically what determines your capacity to enforce some of the measures. For example, in the Santa Lucia Basin, in Paso Severino, the idea was that these areas were the property of the water management company, right? It's a public water management company, the OSE. It was; it owned the buffer until a certain limit, which was the maximum flooding area of the reservoir, right? But traditionally, it has been offered to the farmers, so most of the farmers were, you know, actually in that region, feeling that it belonged to them, right? So when the OSE went there and the Minister of Environment wanted to implement this buffer, what they did was put a wire all around their lands, which was first interpreted or at least perceived by many of the landowners as a kind of "now you are taking away land from me". And that was done quite abruptly, so, you know, you could argue that otherwise you would never have done it; that was the Ministry's perspective. And so then you have this stripe, which is that you have this exclosure in many places, so you have this natural regeneration process of passive restoration, right? In many cases, you have mostly grasslands; in some places, you see a lot of shrubs coming in; in other cases, you have some native woodlands or trees, but also some invasive species coming in, so there is not a lot of management. And then you have these programmes I was involved with, where you would try to include trees based on some kind of spatial configuration that reflects the kind of environmental conditions for trees of different lengths, right? So more hydrophilic next to the water are known associations of trees, which would eventually give way to forest, but not everywhere, just in certain regions that had more suitable conditions for forest. So then you have that kind of thing, and of course you don't have a lot of management because the state, once the programme ends, doesn’t really take care of it. And then you have all these sorts of problems where, for example, the owner of the land, which is the water company, doesn't really do land management, so what you have is one guy there who takes care of the reservoir, but he owns livestock, and he actually kind of uses the place for his own good, you know? The other, which starts complicating things because then you have all these other landowners who felt that their land was removed for them, and they knew the persons or the people who were in charge of taking care of the reservoir, So, they ask, "Hey, you know, can I also put a gate in the fence, and then I come from time to time and use it to feed my cattle, blah, blah, blah, so what can I say? Then you kind of start failing in doing proper management and control of the area, and we are not very, like, environmentally conscious, just for environmental sake. It's very difficult to have a piece of land and leave it alone, you know, just for some forest restoration or something. Thus, in one way, for example, in the programme I was with, there were several things going on. You had to plan trees, but you were also involved in schools and talking to people from other places about where your water comes from. And so you have volunteer programmes, and there were also people from the surrounding areas that were providing the seedlings, so you were generating some sort of local benefit. And you would try to promote some sort of tourism by doing some basic infrastructure, you know, and people trying to use those places for recreational use. So, I would say that you were trying to do some involvement with the community, but I would also say that the interaction with the productive neighbours, like the local producers, was never very well thought out or sold. For example, it's costly to take water, you know, to place out of the reservoir, and some of the plants require it, but that's also some exponential costs, and, yeah, so, and you are not fully compensated; maybe you have some programmes to partially compensate it, but then you are taking land away from them; I mean, it's difficult. And then you can do it, but then you need to have some sort of management capacity and enforcement capacity to make, you know, a different equilibrium, like, move the system to a different state or something. I do not know if I was clear; this is a long story, and I am arising for like one minute, but...

Interviewer 1: No, it's clear to me; I am just thinking about how to follow up from here and what is best. You mentioned that, like the very short version, a more integrated view of the buffer zones and the policy is needed to make them more sustainable and to look beyond just nutrient retention. What do you think would be the benefits if the policy were changed in that way?

Interviewee: The benefits, in general, or benefits for?

Interviewer 1: In general, for either producers or the implementation of buffers, or even the ecological values that can potentially increase.

Interviewee: Well, yeah, like, if you look, Uruguay has, as, honestly, I am more concerned about ecological problems than productive issues, like Uruguay has a big problem of water quality, but that is also linked to the health and general health of the watersheds, the basins, so basically what you are having... I mean, we are not alone in the fact that, like you, you are quickly approaching a collapse in many waterways or aquatic ecosystems; at the same time, you have a weakening of the whole biodiversity of life in the watersheds, erosion, etc. And the agricultural regulations are very poor in Uruguay; like, there is almost no regulation on the amount of fertiliser that you can throw into the land. So basically, there are some regulations with different levels of, how do you say, compliance? I don't know. So they are regarding soil erosion, okay, soil loss, and then you have some regulations that are not very well enforced, but at least you have some, but we have almost no regulation in terms of fertiliser, no regulation, or very few regulations in terms of agrochemicals in general. The environmental regulations for the agricultural sector are very old and they are actually very loose, so basically what you need is to restore, recover, or protect some environmental health in ecosystems, and this seems to be the minimum you can do to protect the river buffer areas. So I think that would be the priority, and that involves, yeah, a myriad of ecological functions that can go from, like, filtering, nutrient filtering, or chemical filtering to, I don't know, even shadowing over the rivers to cool them off. I mean, biodiversity has all sorts of benefits, but in my opinion, you need to prioritise ecological functions in that type of exchange, which leads you to ask, "How do we do it?" Because then, of course, for example, you can, it's relatively easy to have an oversight of agricultural activities by remote sensing, so you can see if there's tillage or, you know, there are planting crops on the land, and the ministry has been doing that. But it's very difficult to know if these are natural grasslands or not, and then people are putting cows there or not, just like the livestock issue is very difficult to enforce. And that's something that you need to work on with the people, like the agricultural issue. I think you can solve it. You could have some problems with planted pastures, but the problem is having livestock on the land. And that is a really difficult challenge, because in that way, at least forests can help a little bit more than grasslands, but still, you could have livestock going to drink at the course, so that's not easy. And then you need to have some sort of programme that makes the thing work; otherwise, you will be in a different place, like a difficult place, if you want to extend or do large programmes of buffer protection, restoration, or rewilding in the area.

Interviewer 1: Yes. I see that Guillermo has joined again. Do you currently have any questions?

Interviewer 2: I listened while I was coming by car. I have; you can go on. I will answer some questions if I can.

Interviewer 1: Of course, regarding the implementation of these policy changes, what do you think are the biggest obstacles to their implementation?

Interviewee: Well, enforcement capacity for one, I think there's already an acceptance that you have to leave at least about buffer zones alone, slowly, especially in some watersheds like Santa Lucia or even Laguna del Cisne, so that the main drinking watersheds are not disturbed. I think there's already some acceptance by actors, different types of actors too, because at different places you have different actors, but I think that in general you have very low enforcement capacities at the state level, and then you have problems like the whole livestock management issue, which is complicated to enforce; I would say it's very difficult. I do not know; you need some sort of engagement with the landowners’ programme to engage with the landowners, but also some sort of support probably from at least some of them; there already are some actions. I am not fully aware of the latest development of Tanques australianos (water storage tanks, red) or other ways to provide water to livestock, and I do not know how extended their use is, but it is always easier to let the cattle go to the river, which is pretty bad. So, you say that outside; I do not know if it's watering or if outside watering is an important solution. Otherwise, will you always have livestock going into the river? I mean, in my opinion, what you should do is mostly exclosures and then let woody vegetation come back, which will happen not in every place but in many places. However, even if you manage to do that, like an exclosure, people will keep sending their livestock in, and it is very difficult to avoid that, which will keep pressure on the forest, for example, because they eat the seedlings and trample on them. Even when you have the forest, then, okay, you are better, but then they will keep pressuring the regeneration because they will eat the seedlings of the forest regeneration and they will still access the river. So, that is difficult to answer, but you could do it, but you need some sort of enforcement. The other issue is that I forgot what I was going to say. Yeah, I do not know, but I forgot.

Interviewer 1: Okay, and how do you think that these obstacles can be overcome? Are there any potential solutions you see as fitting that could help in this process?

Interviewee: For example, in my opinion, you need to have some sort of presence on the ground. So, there are two different things, right? One is, for example, if you want to achieve some restoration of forests or buffer zones, and so you want to regulate, for example, livestock rights. I think it is fairly easy to do it regarding croplands, right? Because satellites can be monitored, it is easier, but in that regard, the only thing you need is some sort of political will to control and some control capacity of the state, right? But you can do it. In my opinion, with livestock, it is more difficult, and you could do some sort of support for others, for example, by providing drinking water to livestock outside of these areas so that livestock do not have to get in, but then you need to either build exclosures, which are expensive, or provide water outside, which is expensive. However, for me, the other thing is that if you can get people to agree on all these things, another thing is to have some sort of physical presence on the ground. Similar to passive restoration, for example, people see it as abandoned land. But the moment you start planting trees, even if it is something symbolic, and you take care of trees, whatever people understand that you are doing something, they are your trees, and the cow is going to eat your trees, you know? At some point, this makes a difference—for example, what happened to us in Paso Severino. In the moment you were present, and you were planting trees, and you made something, well, some neighbours were angry, and they just put their cows there, and they did not care. And they were fighting the state because they took their land away or something. As such, it is very difficult to see something happening and some active stewardship of the land versus just, you know, being excluded, and then that's like a barren site or dirty, you know, "sucio" (dirty, red). It's like, for me, that it's very different. When they see that you are actively managing or having some sort of stewardship over the land, people respect it more. You know? And then, of course, you are there from time to time, so if you come and you say, "Hey, your cow ate my trees, people will understand, "You know, they're grazing on my trees; please remove them, or what?" Therefore, I think that there are different phases. You first need acceptance; people need to say okay and agree. I think we are, in my opinion, already more or less there because people know that the water quality is poor and the problem is worsening. Then, you need some economic support because you need to build physical barriers, but you also need to provide some solutions. For example, to have water solutions for livestock Finally, you need to have some presence on land. I do not know if what I am saying is useful or if it's just absolutely going in the opposite direction of what you guys are thinking.

Interviewer 2: No, no, no. I think it's open. What this process is doing is bringing all visions into the circle. Thus, we have some complementary views and solutions that are rich. So, what you are saying is that I think it's very interesting. The question that comes with that is, what kind of people do you see controlling that? Are there extensionists from the Ministry of Agriculture, or are they part of the Ministry of Environment that are controlling?

Interviewee: In general, the Ministry of Agriculture is the Ministry of Agriculture for landowners and producers. You already have an institutional infrastructure in place. I mean, there are places where people discuss their issues. Thus, the problem is that the Ministry of Agriculture is not active in terms of environmental restrictions. They did not want the Ministry of the Environment. There is normally a fight, and that is also political. You have different layers. I think that technically, people can agree between the two ministries. I was working on the National Environmental Plan, one of my previous jobs, and we actually, most of them, were the richest, I would say, and the most difficult part of the plan was to sit together with the technicians of the Ministry of Environment and the Ministry of Agriculture. In fact, we have made many agreements. However, if politically there is no will, like right now, I do not think there is much room for any of those things because the political leverage of the large producer associations, be it the ARU or the Federacion Rural, is too big. They own the place right now: the Ministry and the Minister. What is the incentive to do something against your productivity? Why do they invest more in the environment? Right now, in my opinion, there is no equilibrium, but that is a political issue. However, if you have political willingness, then you have other things to overcome, like technical agreements. You can easily have technicians from the Ministry of Environment go together with the Ministry of Agriculture and try to work on a solution at that level. Then, you will have some sort of engagement, and it will be easier to develop solutions. Of course, you need the resources that I was talking about, and then you need to have a physical presence on the ground, because otherwise what happens is, oh. This is a new programme; you know, now they want to plug in the water. Then you put some fencing up, and ten years later, nobody's there, and there were cattle there five years ago because nobody came, right?

Interviewer 2: So, I am afraid that it is something that is already happening with Measure Eight in Santa Lucia. The farmers who visited were like, "Oh, okay, I do not know what it is or if it is working or not.

Interviewee: Yeah, exactly. See, that is terrible, but the government works in an environmental field and in a spasmodic way, you know? It's like, "Oh, there's a project, a global environmental facility project. We are going to do this, and then you have 20 technicians, 20 meetings, everybody, and one year later, two years after the project ends, nobody remembers what was done and people do not care anymore. Yeah. This is a real and true challenge for all these things. Thus, there is good luck in finding solutions.

Interviewer 1: I have a few more questions: On a different topic, how have you experienced climate change in Uruguay with regard to temperature, precipitation, droughts, or flood trends over the years?

Interviewee: Of course, let me think that there is an increase in precipitation, there is a more intense increase in temperature, and, for sure, there is more intensity. Therefore, we have slightly more precipitation, but most variability has increased, so you have more concentrated, intense precipitation periods and droughts. Uruguay is traditionally a region with high variability, so you have something that is fairly constant over the year; there is not much annual variability, but there is a lot of inter-annual variability. Traditionally, you have droughts every two years or something, and then a big drought every ten years, which is not very clear, but it is supposed to worsen with climate change. Therefore, if you have more extreme events in terms of droughts and precipitation and more concentrated precipitation, that is bad news. And then, for example, right now, we are coming out of a drought, and it's linked to El Nino. There's no conclusive evidence for what I've read, but it's likely that it's connected to climate change. This intensification of El Nino cycles is one thing, and then the other could be that you have more mild seasons, fewer freezes during the winter, which includes disruption of biological cycles and different pests, and then you have an increase in sea level, for example, and then that would mean salinization in certain cases or an increase in levels of salinity in certain aquatic environments, and I think I might forget it, but yeah, I think that for all these things you will have From the ecological perspective, you could have differences in seasonal cycles; you could have differences in inter-annual and also in annual variability; and from the productive perspective, you will have problems because of this high-intensity precipitation of the drought. But the other thing that's going on is that because of this expansion of crops, because of international prices, and also because of some technological packages, for example, soybean, you have new frontiers of risk. Like you are expanding your agriculture to regions that are less suited for agriculture, and of course they are more prone to risk, and what happens is that because the agricultural sector has such high leverage and political power, you will be financing that increased risk through public investments or public incentives, so basically what you do is you expand your risk and you reduce your adaptation capacity because you are planting soybeans in huge regions that are not very well suited for agriculture. And then you have to follow that up with irrigation or just absorb the damage as a country, but that's a different issue, but that's basically what's happening right now. Yeah, irrigation has a solution to the problem. Yeah, but the problem is, you know, we have one pond, basically one pond per square kilometre; we have almost 200,000 ponds, and they have all sorts of problems because they act as reservoirs for algae. Therefore, you have all these blooms, but they also emit carbon dioxide and methane, which means that it is quite a problem in terms of water quality, which will intensify because you will try to build more ponds right now.

Interviewer 1: How do you think that buffer zones might be affected by these trends or changes? Do you mean directly or through human intervention?

Interviewee: The direct consequences are direct feedback. I am totally guessing here, but in terms of the use of the plant, that's why I am asking, because you could have problems because of the use of the buffers; if you need more water, then you will use the water resources more often, and you will be trying to access them more often. If you have droughts, nobody cares about buffers or whatever; when you have droughts, you know, it's a priority to take the cows to the river or whatever. This has an indirect impact. In terms of directing, I am unsure because, on the one hand, you will have more variability in water availability, but on the other hand, you could have more temperature and more precipitation. And then you can even have particular conditions for the expansion of forests, which is happening in some regions of Uruguay, like in the hills where agriculture is less prevalent. You are seeing some expansion of forests, sometimes because of changes in land use and less cattle and also some conversion to tourist use, but also because you can have these pulses of precipitation in some years, and then you have windows of opportunity for trees to escape herbivory. And then you can even have good conditions for the expansion of forested areas next to rivers, provided that there is no agriculture or land conversion, right? Was I clear?

Interviewer 1: Yes.

Interviewee: However, I am unsure about this, which is a serious remark.

Interviewer 1: Okay, perfect, Guillermo, Do you have any questions?

Interviewer 2: Yes, I do not know how I got to this, but at some point you brought up the idea of declaring riparian buffers, or at least the margins of rivers, as protected areas.

Interviewee: Yeah, well, they are currently protected by some plans.

Interviewer 2: And to include them in the system of the national system of protected areas, is it a possible solution or not?

Interviewee: Look, Uruguay has figures for forest protection right now that are not in the protected area systems, and I am not sure how it will work as a protected area. I think... I was especially telling you because you talked about presence in the place that you were thinking of, a park conservation style, or something like that, or extensionist park conservation. And well, or a type of programme of active restoration, let's say, where people go every once in a month and it runs in the area and it supervises the vegetation, it removes exotics, it happens that it is expensive and there is no will to politics or to finance a programme like that. Now, there is a new project that I do not know how it is working, and the World Economic Fund, which is part of the thing, is to cover the restoration activity of that project, but I do not think that it should be active. How is it called for to continue? I do not agree, but if you look for Santa Lucia, the last project of Uruguay, let us say, in terms of biodiversity and money, I think it is Santa Lucia and areas of activity, and it is quite focused on that type of action, restoration, at least part of it. It also has other things with the system of air pollution, but I do not know if the best one is the system of air pollution or, well, what is used the most are these plans of Cuenca, because it is the Cuenca de Santa Lucia, the Cuenca del Sauce, the Cuenca del Ro Negro, and now with the UPM, they are political instruments associated with the management of Cuenca. The other is some kind of, I imagine, more of a type of norm, like the protection of native grasslands. For some ecosystems, you do not have to forget that Uruguay does not have protection of any type for natural grasslands, which are the most valuable and large ecosystems. There is no sort of protection for conversion, no protection for wetlands of any kind, well, very few protections for marine areas, and no protection up to now. And even the forest, which is the only thing that is protected, is protected by a law from the 1970s, and it does not take into account the integrity of the forest, just the surface, so we are in a really bad place regarding the protection of ecosystems, so this could easily go into that type of protection of buffer areas by law, you know.

Interviewer 2: Then I have no questions.

Interviewer 1: Me neither; all my questions are answered, so, all right, that's perfect.

Interviewer 2: Thank you so much for your time and for your input; it's really helpful.

Interviewee: Good luck, then let me know whatever progress you have; it's really interesting.

Interviewer 1: Of course, thank you so much.

*Interviewer 1 is the student, and interviewer 2 is the supervisor.*

# Interview 23

**Interviewee: Scientist            Date: 24-03-2023**

Interviewer 1: How did you get involved with the Santa Lucia River Basin Commission?

Interviewee: I have worked, as I was telling you at the beginning, on many things, let's say. When environmental problems arose in 1990, one of the things that was accused at the time was that fertilisers were a cause of contamination as diffuse sources. And that was an explanation of what could be happening in the surface waters of the basin. We started to do some work where we somehow demonstrated that the pollution, for example, with nitrate, was in deep waters and that the main cause was not the fertiliser, or that the fertiliser could have some influence, of course, if it were badly managed. After we started to work on that, we were involved every time in the process of the commission, which was our part because since 1970 I have been working on soil fertility, plant nutrition, and fertilisers. I have a lateral relationship, but it is a very important relationship, and I have a different view from the biology people. To all those people who, let's say, as I'm going to tell you, have a very different view, here is what he will understand: when you go to buy vegetables, they have a little sign that says, "Look, don't touch me; don't touch the fruit. They apply the same thing to natural systems because, in the end, that is their objective—to try not to touch anything and for everything to stay that way. That is the bottom line of their vision. I don't agree; I think we have to touch it, but with a lot of forethought and a lot of effort to make it sustainable, it's a slightly different vision. Well, then, and when it came up in 2000, after a water quality crisis in 2013, I was an assistant at the faculty of agronomy, so they asked for the faculty—I was an assistant there for 17 years—and they asked for a representative, and I in turn met the two conditions. I went with another person, and then I stayed until I retired from the faculty. I am from the situation of agricultural engineers, which is what I currently represent, but for a short time because I am already saturated by the unfounded criticisms. As they say, I don't feel like being against it because I have people I know from both sides with different approaches, and I don't want to be arguing if there are no supporting issues. So this is an example of the buffer zone, where there is no data. It is a bit more philosophy than science. I explain it to say it, and then when this begins to predominate and the issues become politicised, which is another big problem, Listen, water problems are politicised; it is very difficult, and your opponent, the representative of the opposition in the most important water consumption body in Uruguay, is a history professor. I have nothing against the history professor; please, I have the best will, but he is not a suitable person to represent. Unless he has studied a lot in the last stage, the mayor of Canelones, who also continuously gives his opinion on water and environmental problems, is a history professor. So he starts to distort visions with political visions, and I do not like that. I do not like it because the water problem is a national problem; it is a problem of state policies; it does not belong to one party or another. When it becomes biased, we lose strength, and, unfortunately, in recent times there has been a tendency towards this, and I do not participate in this; I want to retire.

Interviewer 2: Good. That's fine. But 10 years ago, did the Basin Commission cease to exist?

Interviewee: We say at the university that we have continued to be educated for the last 20 years, since I've been on a number of general commissions. You see, I like to discuss it.

Interviewer 1: Sure. And these buffer zones can be designed in many different ways. For example, by using or promoting different types of land cover and management strategies, there are different ways of defining these buffer zones. What functions do you think buffer zones currently have in the basin?

Interviewee: Currently, I have to explain some previous things. In accordance with what was happening, a sub-commission was formed within the basin that discussed the issue a lot. So the problem is that it was discussed for the most important reservoir, which is the Paso Severino, then the main, secondary, and tertiary watercourses. So in the primary and secondary basins, they are talking about 50 metres. I don't want to make a mistake, but I don't remember the exact number. But I don't agree with that. I think that the assumption is, to give you an example, that Paso Severino has a high part. I did a little work that is available, and I presented it in the commission, where you have a part with a high slope and a flat part. In the steep part, it is essential to make a basin area, but in the flat part, it is the other way around; it is the water that advances over the ground and not the runoff over the reservoir. So the criteria should be different, because there, the one that can even bring phosphorus to the soil is not from above; it is the water itself. It is very common in Uruguay that there are different areas where the phosphorus stays and forms solids that are in the shallows. There are different categories, which I will refer to in a generic way as sodium phosphates, that you should have in the whole area where you are, especially in the lowland area; those are very common. Well, those phosphates are quite impermeable due to their structure, and it is because the sodium is deposited there with the advancing water and generates negative structural conditions. So the solution would not have to be homogeneous, but as in all things to reach an agreement, it was put aside. Now what happens is that you have the buffer zone in the valley or on the slope, which I don't agree with. You have different soils, different structures, and even the vegetation that you mention—there are areas that have dense vegetation and others that don't. The river is even more complicated when you go to the secondary tributaries because they are watercourses. You say that in terms of the river's order, the first level is the headwaters, and the second level is the next level. Up to that point, what is done with the buffer zone seems fine to me, but I think that in this case, the same criteria should be maintained as for the buffer zone. The river is created with three orders, which have not yet been implemented for buffer zones. What happens is that Order 3 is made up of small producers, even the watercourses, and sometimes they are not even permanent. They are watercourses that are cut, so those small producers—if you take away the 50 metres, which is the length of their field—you take away a lot. So I was proposing that nobody give me any advice; it was even with the director of DINAGUA, with whom we have been friends for many years. He didn't agree with me, so I'll tell you that because the discussion was public. I told him that there had to be an agronomist to sign and ensure that the use and management plan were not going to endanger those producers. That was better than taking out that productive area; for those producers, it was very important. It resulted in nothing; neither was the area forced into the buffer zone, nor did they stop doing what I was saying; it is in a state of nothingness. But once you decide to make the buffer zone, having different options can be an option, as it is defended as restoration. When they say restoration, they are telling you to go back to what was there before. And that is the tremendous confusion, because you are an anthropologist; you are the director of the TEC.

Interviewer 2: I'm an economist specialising in environmental issues, and I've been working in ecology for a while now, so I understand the things I hear by ear. So, let's see how Uruguay's vegetation used to be. Before the arrival of cattle ranching, Uruguay was made up of bushes and shrubs. It was a fairly short vegetation; it was not an important vegetation, and the rest was grassland; it was natural.

Interviewee: So, when you say, We are going to create a buffer zone, and you say, You have to restore the native forest, there are two problems. Firstly, the native woodland is not as native as they say, but to make it so that it is, to make it what it was before, the problem is that it becomes a species that grows very slowly. So, when you want to cover soil, you have that problem. I'm going to give you an example that is about to come up. Supposedly, the environmental groups say that the big problem is the production systems. The current production systems, that is, above all, deforestation. Let me give you an example: do you know Canelón Grande?

Interviewer 1: Yes, I know about it.

Interviewee: Well, that dam was built to complement Paso Severino in 1955, right? In the year 55 of the last century, there were no soybeans, and there was no forestation. And how did the dam end up? totally full of sediment. Did you see what came out of it?

Interviewer 2: Yes, mud.

Interviewee: I've bathed there. I can tell you that I already knew that it was all muddy at the bottom. Why? Because they built smallholdings next to it, take the cattle there. Furthermore, they use direct sowing, cover crops, and crop rotations. Then, of course, the sediments from all the fields end up in the dam. Now, luckily, they have the opportunity to get those sediments out. So, when they ask me, What do we do with the sediments? Well, we go back to where they came out, as close as possible. But what's the problem? You have to reupholster it quickly. Do you understand? You have to cover it quickly.

Interviewer 2: How is it done, or did you see the roads when they were made? Do you have to somehow fix the slope at the farms?

Interviewee: Yes. That's what you have to do. You have to fix the slope quickly from where you throw your sediment. And there, you can use grass or plant species that quickly cover the soil. So, nobody thinks of making a tree that grows very slowly. You have to make a plant that grows fast. I mean, you can take a risk. Moving the soil is already a risk, but it will be less of a risk than if you were to grow a tree like that. Look, I was there the other time when they were going to plant at Paso Severino. They did a pilot project there, and they planted all kinds of trees. Well, I don't have anything when the trees are there. I love them; they are beautiful trees. You go to the flat area where they did the restoration to see how the trees are. See if what I say is right or wrong. And I don't need data; I just need to visualise it. Go and see what is left. Ask the person responsible for it, who is also in the state and was on the meeting commission. Let her tell you how she is doing. Because, as a politician, it is like voluntarism. To solve real problems, you don't do it with good will or by dreaming. You have to do it with your feet on the ground, and that doesn't work. You have to have a buffer zone; that's why I made that joke—that you even have to throw a little nitrogen in to test it. That way, the pasture would settle faster. But look, I'm convinced that's what it is. Because I've worked with nitrogen, and then there's nothing left afterwards. This is another mistake made by people who know little, which is to say that the problem is phosphorus. The problem is phosphorus, but there is an older law that we teach in the fertility course. Let's see how European Liebig's Law is. It says that the local yield of terrestrial plants should be limited by the nutrient that is present in the environment in the least quantity relative to its demands for plant growth. So it is very feasible that in the water to produce cyanobacteria, the phosphorus is important and decisive. But if there is no nitrogen, it is cut off. So often the blooms occur because both are there; otherwise they don't occur. And there we go back to the subject of Measure 8 in general. This measure defines margins of a certain width with a measure of not moving the plant cover or vegetation and not applying fertilisers or agrochemicals. Basically, there are three things. After that, it doesn't do much else. Some people say it should have won, and some people say it shouldn't have won. But, well, what functions does that way of doing things fulfil? I agree with all the cattle, no. Because the cattle are the first mess that comes into the buffer. You know it's because when cattle go through their feet, through a watercourse, they throw their dung because, when they feel the cold water, it's like a natural reaction. The cattle loosen up, don't they? There are studies that prove it.

Interviewer 2: If you have any work on that, I think it would be interesting.

Interviewee: Well, there is a work that my friend, a lifelong friend of mine, did in the United States in Iowa called Antonio Maiorino, which is a very nice work, and which is how they increase the amount of that around the water troughs. So, he does like concentric circles of phosphorus levels around that. But what I'm telling you was said by a Dutchman who came to INALE many years ago, and I recorded it mentally; I don't have any data. But it's obvious if you're from the countryside and you look at the cattle: it's a long time when their feet are in cold water. It's logical. Well, human beings too, for that matter. Well, then, having animals also creates another problem: the distribution of animal faeces is very heterogeneous. So, you are creating an additive effect, which means that you are going to have to distribute it again. In other words, it is not advisable. I don't agree with that. I think the best thing to do is to have a permanent pasture, harvest it into bales, and take it out.

Interviewer 2: Do you know why?

Interviewee: First, because you can't have a jungle. You have to keep it more or less in check. But secondly, there is a very important effect, and we still haven't answered it, which is that it is said that when the buffer zones are saturated, so you're going to ask me later, there is a risk of saturation, and then the effect of the buffer zones can be much worse because all the phosphorus that is concentrated there is next to the watercourse. So, it would be much easier to get there if that were the case. And according to my knowledge of the soils in Uruguay, at least, this could happen in Brazil or other basins. In Uruguay, we do not run that risk because we have soils with a high phosphorus retention capacity. In other words, we do not run that risk. I think that if you run those 50 metres, you will surely not have a problem. There is some work done by a girl from the faculty of science. Lorena Rodriguez is the one who was there. This girl, whom I also know, is very serious, and they have already done the work. But they didn't get to the point that I'm telling you about, to see the saturation; no, they answered some different questions, which I don't remember exactly now. But I know they did the work; they are one of the few jobs that are working on this.

Interviewer 2: And then, why is it important to harvest?

Interviewee: Because you are taking the phosphorus out. And by taking that out of there and distributing it in the upstream field, you are lowering the concentration in that place. Because if you fertilise it and harvest it, in the end, even if you don't get much of it annually, it will be so important that it will be even more difficult to saturate it. Well, I tell you, this has not been studied; it would be good to study it when they are planted, or it is going to be a follow-up, but I am sure that the best way for the buffer zones to work is to have a pasture. It should be a pasture that is always there, a fescue (grass type, red). I tell you, there is the name of the plant: a fescue that can be maintained. The problem is that the law that I was explaining, Liebig's Law, will not allow much growth because it lacks nitrogen. That's why I jokingly said that to start with, we had to put a little nitrogen in because for the fescue to establish itself well and to take root well, it had to have strong growth at the beginning. And as they don't know the dynamics of the element, nitrogen in the soil practically doesn't accumulate. If you put it in a measured amount directly with moisture into the soil and into the plant, the plant absorbs almost all of it. It is different from phosphorus; phosphorus does accumulate. And these are things that you don't account for when you make a decision. And that's why I try to speak my truth in some way, but there's not much that I can think of.

Interviewer 1: What are the benefits of these characteristics in the buffer zones?

Interviewee: The benefits, well, the objective is to try to intercept and that in the runoff. It was historically assumed that the cause was erosion, i.e., sediment. This is what I recommend to you from the guy who has worked the most. He was a tall guy, but very smart. He was the first one to even adjust the different methods for measuring total phosphorus in water, which are not the same as the methods we used for soil. Well, Jasper was the first, in a way, to show that not only did phosphorus come through erosion but that there was phosphorus that was soluble, let's say, and when the water passed through, it was also carried along with that runoff water. In other words, the runoff water carried the sediments, but it also carried phosphorus. That's a bit different. And well, for that to happen, there has to be a lot of phosphorus in the soil, especially at the top.

Interviewer 2: What happens when it starts?

Interviewee: When you do direct sowing, you start to see a stratification of phosphorus. There is a lot of phosphorus at the top because it is not reincorporated. This is also going to change. Look, it is already changing. You have probably already interviewed one of my students. Well, I've worked with him in a workshop of mine. He was a student of mine, but we worked together for a long time. In this one, he started to see what was going on with the incorporation. And I'm starting now. Do you know what encalado (liming, red) is?

Interviewer 2: No, it sounds familiar. It seems similar to soil inversion, but what would it be?

Interviewee: Liming is adding a calcareous material to modify the reaction of the soil. In other words, everything to do with hydrogen, oxygen, and lime has to do with neutralising the synaeresis, if there is any. Well, what happens? With this system, the soil above the ground has become much more assertive. So we are proposing to invert it. I told him to include the lime, but he didn't give me a chance. We ourselves, who were working on the same side, have different views. He didn't want to complicate things because it was just one more complication. But now, I give you a prize: your products are emerging from industries that can replace conventional liming, which is a technique that I have worked on since 1975. Well, then, this is an opportunity, because there are also very strong compaction problems because the soil is always unturned. So now, people, don't wait any longer. In the plans of use, it is going to say that the soil is going to be turned to break the stratification and change the pH of the soil for some crops, right? Any of those crops that need, let's say, a pH above 5 So, I think that the objective of the buffer zones is very important because they are like a physical, biological, and chemical barrier to possible excess phosphorus so that it does not reach the basin. So, of course, if you ask me today, the most important effect is to cut erosion as well, because having a vegetation layer is very important so that the sediments don't reach the bottom. Do you understand?

Interviewer 1: You mentioned phosphorus retention and erosion control. Do you see any other benefit or advantage that buffer zones have?

Interviewee: Another advantage—well, another advantage—could be to produce bales at the moment if you find yourself in a buffer zone. If you are making a forage reserve and if you make bales, you have an additional reserve for the winter, but that depends a lot on what area you are in and what that buffer zone is. If you follow the river, I will tell you that there will be no excess production for the winter because those soils are different. That is to say, it is different from what you can find in different areas. But in general, I think that's the most appropriate management, isn't it? That the dry matter production can be extracted. It has a cost, of course; it's not free, and since you don't have that area, it's a bit like the protected area criterion.

Interviewer 2: It's a bit like the protected area; you can do very restrictive things, right?

Interviewee: Yes.

Interviewer 1: And what would be the obstacles to changing the buffer zones for their optimal implementation?

Interviewee: Well, there is some controversy at the level of the environmental police; they receive contrary messages, and also, most of those who do the environmental police are not really agro-police. So, what would be important is that these measures at the field level are taken by environmental organisations. I have nothing against others, against other professions, but that they know, let's say, how the producers are managed and how it can affect the producer's pocket and the producer's balance sheet, for example, with the buffer zones in the third-order watercourses. You are going to make many producers have to sell their land because if you take away, I don't know, 50 metres in front of the watercourse, in other words, have 2 hectares that are not going to produce to make more money, It's 25 thousand square metres, 50 wide by 500 long. Well, it's 2 and a half hectares, and for a producer who has 10, 12, or 15 hectares, that's a lot. It's the same account that I make, you see, when they make, for example, a dam; now the Paso Pache dam is in vogue. Don't forget that when they built the dam, they converted a lot of land that is productive today into non-productive land. When you build roads here together with those huge sheds, where there was vegetable production, now what you have is a shed that produces—I don't know what it produces—but what you see around the shed is more soil because they have degraded it. And that was signed by the Canelones municipality because the Environmental Police didn't go in there; they should have gone in there. So, the Environmental Police has to have a more agronomic approach. Secondly, once the decisions have been made, whether they are good, bad, or regular, we have to try to implement them. And in order to implement them, the producer, or the majority of producers, do not distract their resources and time on things that they do not consider important; that is, they do not see their direct importance. So you have to look for incentives and ways to compensate them for what they are supposed to lose. Because if he doesn't, he doesn't do it. So, you have to take advantage of the fact that when you give loans or make a kind of policy to consider these areas, you are going to make a long-term investment that suddenly is not going to produce what you expected, because if you don't allow them to fertilise, you don't leave anything. The other day, I laughed a little bit when he said that he was going to make protected areas at the will of the producers. I never saw that. Nobody likes to have just Bambi and then eat the Bambi; that's what I'm saying. It is very nice that these areas are being protected. But what is produced there? If you bought land to produce, and if not, you can't plough, you can't do this, you can't do that. What do you think it does? You have to understand that not everyone can have hives. So I tell you, you have to be careful when people take that measure, especially the people I know that I do not know what they live on, because the truth is that they have a very conservationist look; it has not been anything else but very conservationist. If it's okay, deep down I always say that if there hadn't been some pressure on the bin, the bin would be a disaster. But thanks to those people, much more restrictive measures were taken. They have an important function in society, but we cannot give them a say in everything because, obviously, their scientific knowledge and their approach are more spiritual than rational.

Interviewer 2: Alfred talked about beekeepers. He said not everyone can live on hives in buffer zones. It is not feasible to think of buffer zone production based on hives, for example.

Interviewee: I told you more in regard to the protected area than the buffer zones because these are low and I do not know if the hives have problems. But the truth is that they can be suitable areas for hives because you do not apply anything to the visit. In that respect, it is good. Even there, it has an advantage; the native forest has good flowers for the hive, and that's why it defends itself. I think you can also make native forests, but below them they put a good permanent pasture base. I'm not saying don't do it, but first make me the pasture, and then put the little trees that grow slowly. Although it is very good wood, it cannot be used as firewood. And it is very difficult to work for other things.

Interviewer 1: I forgot to ask—a while ago, you mentioned the different benefits. Nutrient retention, erosion control, and forage for cattle.

Interviewee: Yes, right.

Interviewer 1: And you can rate these benefits according to what you consider most important on a scale of 1 to 4.

Interviewee: There is no doubt that the central objective is to stop the nutrient shift and control erosion. Then, to isolate the buffers from the presence of animals (3), and lastly, the production aspect (2).

Interviewer 1: Sure, thank you. And another question: how do you experience climate change in the Santa Lucia River basin?

Interviewee: Flooding is a factor that should be considered. There are floods because, as I told you, on the one hand, Santa Lucia has a slope to the river, and on the other side, it has a flat area that is where that filling occurs, which includes precisely the basin of Santa Lucia. The Santa Lucia basin was actually defined by geologists because, because geology was formed there, its sediments were left there because it was all water. So, when the water somehow goes down the river, the soils that are next to it are very differentiated, and that could perfectly be made for rice production. Why isn't rice made? Because there is a priority for drinking water. If you had a lot of drinking water from somewhere else, you could make a rice basin that is very good in Uruguay. Regarding cultivation, historically, Uruguay was a pioneer. We compete globally, and of what rice is produced, 95% is exported. It is a negotiation. However, here in this river, you cannot do so. But the soils that are there, the river advances on them and then recedes. What is the danger, for example, when you are very close to the river? Or that everything flows and all that, especially the dung of the cows and all that, is very close to when they ask for the flood and then take it away. And I'm going to tell you about another case that you know, Alfred, which is the most enigmatic case of another important river that is now being discussed. Very important. There is a city where the river, when it rises, floods the cemetery. What happens in the cemetery? There is a lot of phosphorus.

Interviewer 1: And as a final question, how do you think these changes will affect the performance of buffer zones in the Santa Lucia River basin?

Interviewee: Well, the most serious problem you have is flooding, because droughts and floods are frequent. The problem is that if you have an area where clutter has accumulated, it can overflow. Do you understand? And the water drags back what you accumulated. I, in those zones, would not buffer zones either. Did I tell you what they did about the low areas on this side, the Canelones area? But I don't want to get into that discussion because most don't even know what's on this side. When you meet people on the 18th of July (the main road in Montevideo, red), in Centro Montevideo, most of the people who are there have never walked in the countryside. Do you understand? Because nobody went out on the field when people showed you that there was another Uruguay. And that's where they started. Now, when they go to the agronomic part, logically, they have no preparation. And they pass the same on to engineers. My boss is an engineer—a great engineer. And yet, she worked for the basin. She was the one who put together the first action plan. Well, she worked at Antel. I don't know if you've met her. Now he works alone, as he is. Because the calibration was done in the faculty of science. Here goes. I didn't see myself by any name. Where are you in the Faculty of Science?

Interviewer 2: I am in the Faculty of Economics.

Interviewee: Ah, economics. I gave a speech at a conference that I attended, in which I presented a paper on the import and export of nutrients in Uruguay. Yes, I am at the Faculty of Economic Science. There it goes. And I don't remember that there were two women; I remember that they were the ones who organised it. That was very good because, as I said, I'm an advocate. I always said that there had to be people from economic science. I think it's very good that you're there. With your training. We are doing the best we can here. I'm very glad. Well, I didn't tell you about the study of the fertilisers that are used in organic inclusion. You use phosphorite. Phosphorite is the one that has the most influence on us here. And yet, the environmental groups say, Oh!" And you know why in Europe they don't bring in that phosphorite anymore? There is a law that says it has to have less than that. And do you know where they get it? In the province of Kola, in Russia. When they went east, they saw that there were much better deposits. So they took out a decree. Shady, you Europeans. Look how shady!

Interviewer 2: I withdraw because I have another meeting now. So thank you for the time and for your visions and experiences.

Interviewee: No, excuse me.

Interviewer 1: Thank you very much for your ideas and your time. It's very interesting. Do you have any questions?

Interviewee: No, I'm fine. See you later.

Interviewer 1: Thank you. Goodbye.

*Interviewer 1 is the student, and interviewer 2 is the supervisor.*

# Interview 24

**Interviewee: CAF            Date: 28-03-2023**

Interviewer 1: What do you do for a living?

Interviewee: I have worked in daily production for more than 35 years, and I also teach at the university. I was also an advisor in dairy production at the university for the last time, and I work a lot with effluent. When I work daily, I work a lot in effluent in daily production, which is all related to the buildings and the structure of the dairy farms, and I work a lot in administration because I am an anaerobic engineer, but I was moved to the centre of the cooperative to work as an administrator. It is not my business, but I need to learn a lot, and I have adapted well. But I always try to go outside of the office and be with the farmers because the content is always important. If you want to be the reality, to know the reality, you have to be with the farmers all the time and live with them and their problems every day; that was a way for me to work. An alternative I decided to always do, and that was my intention, was to never lose the perspective of the farmer because it is important. We move the money for the farmers, the payment for the farmers, and the relationship with the farmers and the cooperative because it is an important cooperative in Uruguay (Conaprole). And I also work a lot on the modifications and changes the cooperative made before 1999. I will be in contact with other cooperatives in Uruguay and also around the world, and I will display a different kind of work because it is necessary, not for other reasons but because it is important. I knew the effluent problem was really important when I visited New Zealand in 1997, and I worked on this issue for a long time with the government, with authorities, and with an institution from another country, especially Australia. I learned a lot from Australia, and then I had the good opportunity to learn a lot more, visit New Zealand again, and visit Australia again. With Santa Lucia, we have a lot of problems because, at the beginning, they accused the dairy farmers of being responsible for the problem. But that's not true because we have made a lot of controls, and effluent isn't the problem. If I try to explain to you what the big problem in Uruguay is in relation to Santa Lucia, you have three reasons. The first is that our soils are very clay-ish and have a lot of clay, and infiltration is very low, especially when wet. Here, you have better soils and an infiltration rate of five millimetres per hour. What happens when you have a rainstorm? In Uruguay, sometimes you get 100 millimetres of rain in one hour. Since 95 millimetres of water run outside of the soil, you have no opportunity to retain this soil. It's really quite different from Argentina, as Argentina had an infiltration rate of 50 millimetres per hour and the rain could infiltrate the soil. You have no rivers, you don't have creeks; it's really quite different from Uruguay, which has a lot of water run outside. The second problem is that we have a slope of more or less than 3-5 percent, which increases the risk of movement of small particles of greater size in the soil that contain a lot of phosphorus, which is very, very, very complicated. And then these particles end up in the water, and the big problem is that the rivers and small creeks are full of mud and soil, and when you have them in the summer, they are not deep. What do these do? They increase the movement of the phosphorus in the solution, and if you have a little rain, this phosphorus will move to the Santa Lucia River, contaminating it. The third reason is our rainfall conditions. In Uruguay, you either have no rain or a lot of rain. It's different from other countries, where you have seasonal rain, but here it may rain on any day. This is very difficult because, for example, in Australia, there is a station, Ellenbank, where they have studied a lot about this problem of phosphorus in the river because the Moral River is the only source of water for the population of Melbourne. Australia has a big problem because the only source of water is the Moral River, and they made a control about the foot for the movement to the river; they have a station called Ellenbank.

Interviewer 1: That's a very comprehensive and clear explanation. I have a question. For instance, you already said that farmers were seen as evildoers in this process, while that's not necessarily the case. How can an organisation like the CAF play a role in the dissemination of the positioning of the farmers in such a problem?

Interviewee: Actually, no, this programme is more conducted by us, from the cooperative and the Ministry of Environment. I have personal contact with the person who is the Minister of Environment, and when I told him we needed to resolve this problem, he helped us and gave us all the support we needed. It really was very, very important. I have a good relationship with him, and we work together, but we're also probably going to work on this problem, and there is no political condition. First, based on what was learned from the cooperative, we decided that we needed to resolve this problem because we needed people to live in a better environment. That was the idea, and also, the farmers don't need a law to do things. They understand it is important to be in a better environment for them, for the cows, and for the people who live around them. And we received very little help from the government—little money and very few assessment advisors—to help the farmers. That's the reality, and probably because some of the advisors coming from the industry don't understand how the farmers work differently, they don't have the opportunities and abilities to work a system that was created for the industry. I have a very good conflict with some of them because, as I say to them, the farmer has no time to work in an effluent system; they need to make things that are quite simple. More simple than what you propose because they don't work. Don't give them any money to make a complex system; they will try to resolve it simply. Okay, that was the very reason, and the problem for me is important because we need to try to control the pollution in the water if you want my condition to work out for a long time. You need to reduce the speed of the water on the slope. That's one of the things you need to do: make terraces and systems that control the speed of the water. Keep the areas where you collect the water with grass or with a cover that reduces the flow of the water, and keep the areas near the river without the soil crops. That is very important. Also in Uruguay, we have some places where there is groundwater, and a lot of farmers are actually destroying the dams and the water reserves because the quality of the water is not the same if you have underground water. And I told them this is not good because the dams are regulators of the flow of the water, and if you have a very strong rain, the dam reduces the speed of the water. They are a regulator of the flow and also retain the small particles; they are very rich in phosphorus. It is a good idea to keep the dams rather than eliminate them, which is a good idea in some places. Probably in the future, you will need to clean the small rivers of mud to increase the water quality. Actually, the farmers minimise the use, especially of phosphorus, because it's expensive, they have the opportunity to analyse the soil, and they don't use it in excess of phosphorus. It's not a big problem, but you also have the problem because if you put phosphorus in the soil and you have rain immediately, you have the 20 days that Ellenbank recommends, for example. Ellenbank said you need to put the phosphorus in the soil 20 days before the rain because, in your way, you never know if the rain is coming tomorrow or in 20 days, and if you put the phosphorus in the soil and you have rain immediately, part of the phosphorus will probably go out of the soil. This is one of the realities: people need to learn more about how to include phosphorus in the soil. We are using direct tilling; we don't move the soil, and we don't plough the soil. Try to reduce the effect of the rain; if you move the soil using the plough, the land remains more open, and you have the problem of moving away from the water. We are using direct irrigation on a lot of farms; actually, that's the reality; they try to keep part of the grass or crop in the soil to reduce the effect of the drops of water under the rain. That's the idea.

Interviewer 1: Okay, one of the possible solutions that the government is currently working on is riparian buffer zones.

Interviewee: There are no laws in Uruguay about that. For example, in Ellenbank, they may have control over the slope, the amount of phosphorus, and the amount of rain you have in Uruguay, but we do not have this kind of information. I have a lot of conflicts with the technician of the ministry sometimes because, okay, everything is okay, but you need to meet first. We need to know first what happened with the rain and what happened with the amount of phosphorus coming outside of the crops. But they have no information, okay, even when I tell them to ask Ellenbank because they have a lot of information. It's not similar to the New York library because it's in Australia, and Australia is quite different, but they have the methodology and the idea of how to do things to ensure this problem. But no, they suppose they know, but I have no real information, and this information doesn't exist. It doesn't exist, including the impact you have sometimes on the cities, because a lot of cities are near rivers, and all the water in the city is coming from the river, including the water from the streets. There, nobody knows where the water from the street and from the houses goes, and nobody has measured the impact of phosphorus related to the cities, which is also important.

Interviewer 1: Okay, and apart from consulting other experts that have more experience in this field, what other recommendations, for instance, would you, if you had the chance, give to the government to improve this process and reduce the number of nutrients that end up in the river?

Interviewee: The first is that you need to take control of the amount of phosphorus you have on your farm as a farmer. A second try at reducing the speed of the water using the terraces of the river failed. Third, you need to keep the areas where you collect the water protected because in one place the water ends, and you need to keep this area protected as well as the areas near the rivers. It depends on the slope. If you have a very low slope, you probably need 50 metres or less, but if the slope is very steep, you need probably 200 metres. It depends on the condition. It's not only one measure; maybe more than one. The other condition is to try to put the phosphorus under control. If you have the equipment, you can put the phosphorus under control while still controlling the effluent problems. We are doing this now, but maybe you try to keep your attention on the use of phosphorus in the effluent to control it and not go to the world. That's the recommendation. I suppose I'm very important now, and if I try to tell the government the other things to do, maybe go study the impact you have with the water, with the rainwater, on the movement of the phosphorus. We have no information about that; we have very little information, and the only information I have about this issue is related to Iowa, where they made a study with an agronomic engineer who was working in the United States for a long time ago and had a study about this and on the station in Australia. I don't know how our country does this study; everybody talks about things but not with information. I need the information to be a scientist, and if I told you that's the only thing, I'm not a scientist; I'm a speaker.

Interviewer 1: Of course, if the government were to implement these changes, for instance, how do you think they can be best disseminated or communicated to farmers in a way that they understand and are also willing to implement them?

Interviewee: First of all, I believe the farmers know how important it is; nobody wants to put money into the river. It's not a good idea, but probably they need more information about the crops and how to use the fertiliser better; maybe that's the idea. They don't listen to any information of this kind; for example, you try to tell your forefathers not to worry if the rain is coming and to wait at least 10 or 20 days before it starts. They never listen; the government says something like this, but I read in a lead bank that a lead bank says okay to the farmers; it's playing. It's important not to put the forefathers in the field if the rain is coming, but here nobody in the government talks about this, and there are a lot of easy things that you can advise the farmers about. Our farmers are quite intelligent—more intelligent than people think, really—and they quickly understand and improve things. I'll give you an example: Before the law, I built over 700 effluent systems. I don't need the law to convince the farmers to install an effluent system. They understand quickly, they know it's a problem, they try to resolve it, and they don't need a loan. I was an assistant advisor for a long time, and I know if a person understands what is important. They do things quickly. I always talk to the government; it's easy to make a loan, but it's more difficult to make culture. It's important; you make culture with the farmers, but when you have the culture, people keep the things. I was telling you I made a programme many years ago; I don't know how it's now, but they made a programme called Lanter, and a group of farmers from Onaria keep the trees, keep the rivers, keep the lands, and from them they promote the environment of this area. This was a good idea because the government provided some help, but the farmers do the work, and the farmers are implicated in this world. I don't know how it is now because the person I knew in Australia died years ago, and I have no contact with Australia. I lost contact with Australia during my retirement. I am still in contact with another person, but I'm retired. This problem is important for the government to say, "Okay, we try to keep the soils safe and try to recover the rivers and creeks that are contaminated with the soil, so that is the important thing. We need to persuade the farmers. Okay, you have very high receptivity from the farmers. But they need to understand that the problem we have in our soils in Uruguay is quite different from the problem, for example, in the soils of Argentina, Europe, or Australia. It's really very different. That's the idea; I will explain. Okay, it's clear. Any questions?

Interviewer 1: Yes, I do; you referred to it before as one of the potential measures that should be combined. One of them was terraces, for instance, to reduce the slope of the river bank, so to speak. How do you think that these terraces can be designed and used for productive purposes? So do you think that natural vegetation, for instance, would be better?

Interviewee: No, the farmers have a lot of experience using it, but probably since the last time, the importance of this measure has diminished because they suppose using direct drilling is enough. But it's not enough, and I know it's not enough; I believe it's not enough. I have no information, but I believe it's not enough. We try to reintroduce them to keep the dams, and I suppose they do not have a real idea of how to improve this. Also, if you are drilling, you must keep the surface level. When you are drilling, you try to drill at the same level to reduce the washing off of puffers. Okay, these kinds of things are probably undervalued by the farmers because nobody explained them to them. You must do these things better, but there are little things to improve to reduce this. I also probably included some programmes to clean the rivers because, in some areas where I have worked for a long time, you have small rivers that are very deep—three metres or two metres of depth. But actually, you walk up this side, in some parts, and you have small lakes and small areas with water that have a high amount of phosphorus in them, including a dam made from the government's administrative water in Canelones. Canelones is a city near Montevideo, and the government made a dam years ago to retain water for the population of Montevideo. This dam is actually full of mud. The amount of puffers in the water of this dam is normally 2,000 parts per million, when you need 75. When the water revives the dam, you contaminate the river. The origin is very clear; the dam is full of mud. The water has one or two metres of depth, and in summer, puffers move into the water and go to the river. They probably need to change a lot of things; they made another. Do you know about Paso Severino? It is another dam they made to keep the water, and they, for good reason, made a fence all around this dam to keep it safe, something they don't do in the other dam. It's a big difference between Paso Severino and Canelones. I suppose the big problem is that, for example, in the winter you have no big problem with puffers. Why is that? You have an illusion of a problem because rain washes away all the puffers, but in the summer there is less rain, a higher temperature, and a larger number of puffers. From the soil, which moves to the water, if the rain is very low, the concentration is very high. If you look at the registration of the OSIC, which is the organisation that is regulating this, rain falling in summer and puffers are closely related. It's a problem that you've needed to resolve for a long time, and it's not easy to resolve quickly. You need to take care of this problem for a long time; it's a political challenge to resolve over a long time. That's my impression. For the reason I started working in 1997, that's the idea; it's not easy work; it's permanent work; small pieces; all are important in this; okay, that's my idea; okay.

Interviewer 1: Okay, and I have a question that is related but a little bit about a different topic: How do you think that potential climate change can play a part in these developments?

Interviewee: In my opinion, you need to make different cases. For example, with the dairy farmers, you have the dairy cooperative, which is the most important year-round. I work with the cooperative directly, okay? I made an arrangement and worked together; that's a better idea because you don't need a lot of personal information; you have a contact, and the relationship is made. Actually, it's easy to do that, and I work with dairy farmers. Agriculture is quite different because it is sometimes more dangerous. I suppose it's more dangerous than dairy; in dairy, you move the land less frequently than in agriculture. With agriculture, you probably need to make the work more intensive and more controlled, and you probably need to make some regulations about how you do things, like how you protect areas and how you take care of the amount of food you use. But really, I don't know because I've never worked with this kind of problem. But it's necessary to introduce some investigation into crops of this type in the sun, particularly because they are integrated. They know a lot; they are quite intelligent, and it's easy to introduce the problem. In crops, it's quite different because sometimes it's a person who rents the land and knows how the future is not so important, like a farmer or a dairy farmer, who is thinking the land is from the child. It's the future of their children, and then it's a different division related to a person who made a crop. I planted a crop on rented land. I don't take care; perhaps you should pay more attention to this person. This is my idea, but for me, it's very important to try to connect the people that are in production, have them, and make control over them what is important in this job. For me, culture is the most important thing in everything you try to introduce. You've made a law, but people try to escape from it; it's fine; we have a Spanish capacity; maybe it exists in English, but you've made the law; okay, it will always exist in this way to escape; okay?

Interviewer 1: I don't have any questions left at the moment. Do you have any questions for me, or is there anything you want me to know from your side?

Interviewee: No, I suppose. I think very well that this issue is important for the country, but also that they must take the actions, not the law. The actions—this is a big difference between the law and the action, and you need to introduce actions and help and advisors that help people improve and resolve this problem. In the agronomic engineering area, we have a lot of people who know a lot about how to reduce the flow of water on the slope. We have a lot of people who probably never worked on this problem, especially in this area, but it's a good opportunity to introduce them and to help the farmers understand what happened with the forces they put inside. That's the real thing; we need some more work on that. We need more work on that. We need to regulate how the water will flow; it will always flow to the river, but we need to control this flow to reduce the space, to really reduce the movement of the land, and to reduce the contamination of the river. That's the idea; how to do that is the important thing to teach the farmers.

Interviewer 1: And I know that the government is currently working on an action plan that involves buffer zones that have this exact purpose.

Interviewee: Yes, they made a programme related to the use of the soils in the area, but, for example, you as an agronomic engineer and an advisor from a farmer need to fill out a form that says we are going to do this. Okay, complete this, but more than this, it is important that the farmer and the advisor understand how to do the bad things. It's not only to fill out a paper; it's not easy. You can fill a paper; it's easy to do, but it's more complex when you go to the farm directly to the field, to the ground. They need to understand, and you need to train the people who are going to do the job on how to reduce this flow of water. For me, this part of the work is not done; that's my impression.

Interviewer 1: And it's very valuable to have that impression, so thank you very much for it. Interviewee: Okay, no, if you need something more, please contact me, okay?

Interviewer 1: Okay, thank you. Thank you so much. Again, I don't have any questions at the moment; is there anything else you would like to discuss?

Interviewee: No, if I have other information or another idea, I will send it to you, okay?

Interviewer 1: It would be perfect. Okay, thank you. Goodbye.
