# Supplementary material for: Understanding Stakeholder Perspectives on the Implementation and Management of Riparian Buffer Zones in the Santa Lucía River Basin, Uruguay
Source: Environ Manage. 2025 Jul 26;75(10):2596–613. doi: 10.1007/s00267-025-02230-1 (PMC12457512; doi:10.1007/s00267-025-02230-1)
Supplement: Supplementary file 2 — Supplementary_Materials_Codebook [file 267_2025_2230_MOESM2_ESM.docx]

Supplementary material: Code book

*This document provides an overview of the coded segments used from the interview transcripts, and their respective code used for the analysis of the interviews.*

| **Code** | **Coded segments** |
| --- | --- |
| Current ecosystem services riparian buffers > Current ecosystem services CAF > Water flow alteration | You need to reduce the speed of the water on the slope. That's one of the things you need to do: make terraces and systems that control the speed of the water.  Interview 24 English: 5 - 5 (0) |
| Current ecosystem services riparian buffers > Current ecosystem services CEUTA > Biodiversity | but also, to operate the buffer zone system in multiple ways to bring in pollinators,  Interview 2 English: 9 - 9 (0) |
| Current ecosystem services riparian buffers > Current ecosystem services CEUTA > Nutrient retention | Then, there (on the buffer, red.) it works as a bio-filter, it works by trapping sediments and active particles from agricultural activity. It works by slowing down the velocity of water, and therefore, increasing that capacity to retain sediment, and therefore, preventing all these particles from agricultural and livestock activities, also organic matter, to end up in the bodies of water.  Interview 2 English: 7 - 7 (0)  not only enriching its capacity to filter and slow down sediments and trap nutrients,  Interview 2 English: 9 - 9 (0)  fix nitrogen,  Interview 2 English: 9 - 9 (0) |
| Current ecosystem services riparian buffers > Current ecosystem services CEUTA > Watercourse protection | we also included the enrichment with native woody species that were more hydrophilic with the profile closer to the watercourse, to support the riverbank.  Interview 2 English: 9 - 9 (0) |
| Current ecosystem services riparian buffers > Current ecosystem services CEUTA > Sediment retention | not only enriching its capacity to filter and slow down sediments and trap nutrients,  Interview 2 English: 9 - 9 (0) |
| Current ecosystem services riparian buffers > Current ecosystem services CNFR > Biodiversity | What is clear is that what is intended is to respect the areas that rivers naturally occupy and the vegetation that is nurtured there; its flowers and fauna. In other words, all the biological diversity that is nourished by that riverbed.  Interview 5 English: 18 - 18 (0) |
| Current ecosystem services riparian buffers > Current ecosystem services CNFR > Erosion reduction | preventing erosion  Interview 5 English: 18 - 18 (0) |
| Current ecosystem services riparian buffers > Current ecosystem services CNFR > Limiting agricultural frontier | limit of what seems to me to be the agricultural frontier or the productive frontier  Interview 5 English: 18 - 18 (0) |
| Current ecosystem services riparian buffers > Current ecosystem services CNFR > Nutrient retention | start using less fertiliser in one place  Interview 5 English: 16 - 16 (0)  cleaning the water,  Interview 5 English: 18 - 18 (0) |
| Current ecosystem services riparian buffers > Current ecosystem services CNFR > Watercourse protection | the vegetation that was protecting me and that acted as a containment of the riverbed.  Interview 5 English: 18 - 18 (0) |
| Current ecosystem services riparian buffers > Current ecosystem services CNFR > Sediment retention | Not to mention all the ecosystem services they fulfil for society in terms of cleaning the water, the conservation of soils  Interview 5 English: 18 - 18 (0)  transporting soil soluble  Interview 5 English: 18 - 18 (0) |
| Current ecosystem services riparian buffers > Current ecosystem services DINAGUA > Erosion reduction | their most important function in Uruguay is to act as a barrier to mitigate the transport of nutrients through erosion.  Interview 6 English: 20 - 20 (0) |
| Current ecosystem services riparian buffers > Current ecosystem services DINAGUA > Nutrient retention | The function was to mitigate the impact of runoff, of nutrients, in the watercourse.  Interview 6 English: 10 - 10 (0)  their most important function in Uruguay is to act as a barrier to mitigate the transport of nutrients through erosion.  Interview 6 English: 20 - 20 (0)  retention function of the buffer zone  Interview 6 English: 27 - 27 (0) |
| Current ecosystem services riparian buffers > Current ecosystem services DINAGUA > Watercourse protection | The buffer zone protects the coast of the main watercourses of our country.  Interview 6 English: 20 - 20 (0) |
| Current ecosystem services riparian buffers > Current ecosystem services DINAGUA > Runoff reduction | The function was to mitigate the impact of runoff, of nutrients, in the watercourse.  Interview 6 English: 10 - 10 (0) |
| Current ecosystem services riparian buffers > Current ecosystem services DINAMA > Biodiversity | While buffer zones can serve other purposes, such as biological protection, we've primarily focused on pollutant filtering.  Interview 10 English: 13 - 13 (0)  biodiversity protection  Interview 10 English: 19 - 19 (0) |
|  | The area was designed with other ecosystem services, and indirectly, there is conservation of biodiversity in that area by not modifying it, but that is not the main objective.  Interview 12 English: 13 - 13 (0)  Today, the issue of diversity as biological corridors is being discussed.  Interview 12 English: 13 - 13 (0)  The lowlands are very degraded and can act as biological corridors with more ecosystem ecosystem services that we are not currently seeing  Interview 12 English: 13 - 13 (0) |
| Current ecosystem services riparian buffers > Current ecosystem services DINAMA > Erosion reduction | erosion protection  Interview 10 English: 19 - 19 (0) |
| Current ecosystem services riparian buffers > Current ecosystem services DINAMA > Nutrient retention | For us, the primary function of the buffer zone is to retain pollutants, primarily nutrients, from the soil and prevent them from reaching the watercourse.  Interview 10 English: 13 - 13 (0)  nutrient retention  Interview 10 English: 19 - 19 (0) |
|  | main objective was to prevent nutrients from entering the city's waterways  Interview 12 English: 3 - 3 (0)  The original idea was to use it as a barrier to prevent nutrients from reaching the course.  Interview 12 English: 13 - 13 (0) |
| Current ecosystem services riparian buffers > Current ecosystem services DINOT > Biodiversity | inputs, soil characteristics, land cover, soil types, geomorphology, biodiversity,  Interview 21 English: 13 - 13 (0) |
| Current ecosystem services riparian buffers > Current ecosystem services DINOT > Watercourse protection | At that time, from 2011 to 2015, Uruguay started to use buffer zones as a management tool for the water system. Not only in terms of water quality, but also in terms of regulating runoff and flooding, so it was a very integrated thing.  Interview 21 English: 5 - 5 (0) |
| Current ecosystem services riparian buffers > Current ecosystem services DINOT > Runoff reduction | At that time, from 2011 to 2015, Uruguay started to use buffer zones as a management tool for the water system. Not only in terms of water quality, but also in terms of regulating runoff and flooding, so it was a very integrated thing.  Interview 21 English: 5 - 5 (0) |
| Current ecosystem services riparian buffers > Current ecosystem services DINOT > Water quality regulation | At that time, from 2011 to 2015, Uruguay started to use buffer zones as a management tool for the water system. Not only in terms of water quality, but also in terms of regulating runoff and flooding, so it was a very integrated thing.  Interview 21 English: 5 - 5 (0) |
| Current ecosystem services riparian buffers > Current ecosystem services producers > Biodiversity | They also serve as a type of wildlife reserve.  Interview 16 English: 19 - 19 (0) |
| Current ecosystem services riparian buffers > Current ecosystem services producers > Erosion reduction | The purpose is to maintain that zone, which is a filter zone, right? In other words, to preserve the quality of the water.  Interview 4 English: 39 - 39 (0) |
|  | One protects the watercourse with vegetation, and it is also a benefit in the sense that it protects against erosion.  Interview 13 English: 9 - 9 (0)  First, protecting the soil from erosion  Interview 13 English: 15 - 15 (0) |
|  | It would be worse if there were nothing, because the water damages the soil when it accelerates, when it has too much speed. So, that mass of grass is also what slows it down, its growth more or less controls the runoff in some way, right?  Interview 16 English: 21 - 21 (0) |
| Current ecosystem services riparian buffers > Current ecosystem services producers > Nature protection | and it ecosystem services as a natural barrier.  Interview 11 English: 11 - 11 (0) |
|  | Well, first, the preservation of the environment, taking care of the water resource from the point of view of human consumption, because it is the same for us, and we must absolutely respect them.  Interviewer 2: In other words, you wouldn't change anything about their ecosystem services, that are currently being fulfilled?  Interviewee: No.  Interview 16 English: 35 - 37 (0) |
| Current ecosystem services riparian buffers > Current ecosystem services producers > Nutrient retention | The purpose is to maintain that zone, which is a filter zone, right? In other words, to preserve the quality of the water.  Interview 4 English: 39 - 39 (0)  Well, the presence of species, that is, in order for the buffer to work well, it has to have species that are active in their uptake, right? In other words, (species that, red.) photosynthesize, that take nutrients from the soil, right?  Interview 4 English: 42 - 42 (0) |
|  | and it ecosystem services as a natural barrier.  Interview 11 English: 11 - 11 (0) |
|  | I think the buffers are very important, firstly because they are a filter for the watercourse.  Interview 13 English: 17 - 17 (0) |
|  | And the retention of nutrients, for example, the retention of pollutants?  Interviewee: Yes, also, without a doubt. But its less important than water, so 3. For me, the issue of water would be more important than anything else.  Interview 16 English: 50 - 51 (0) |
| Current ecosystem services riparian buffers > Current ecosystem services producers > Water flow alteration | It is like a huge sponge. So, it also serves certain types of water services, right?  Interview 16 English: 21 - 21 (0)  And for example, it affects how fast the water flows away, right?  Interview 16 English: 21 - 21 (0) |
| Current ecosystem services riparian buffers > Current ecosystem services INIA > Recreation | Yes, it's a visual aspect. For the vegetation cover, it is particularly important. For many people it's important as they believe it is essential. I'm not sure about younger people, but probably yes.  Interview 15 English: 35 - 35 (0) |
| Current ecosystem services riparian buffers > Current ecosystem services INIA > Biodiversity | I know that it is only partial, but retention of nutrients and particles, and of generating biological corridors for wildlife.  Interview 15 English: 27 - 27 (0) |
| Current ecosystem services riparian buffers > Current ecosystem services INIA > Nutrient retention | obviously, it is going to be an important physical aspect to stop drag particles  Interview 15 English: 23 - 23 (0)  in the Santa Lucía river basin; the protection ecosystem services of the buffer zones are most important; the function of retaining nutrients.  Interview 15 English: 27 - 27 (0) |
| Current ecosystem services riparian buffers > Current ecosystem services locals | For example, there are many people who need firewood, and there is a lot of tree felling going on.  Interview 1 English: 11 - 11 (0) |
| Current ecosystem services riparian buffers > Current ecosystem services locals > Nature protection | Due to the fact that it was a place for birds. In other words, there are many migratory birds in the area.  Interview 1 English: 19 - 19 (0) |
| Current ecosystem services riparian buffers > Current ecosystem services locals > Erosion reduction | Furthermore, also for the containment of erosion.  Interview 1 English: 19 - 19 (0) |
| Current ecosystem services riparian buffers > Current ecosystem services locals > Wood production | For example, there are many people who need firewood, and there is a lot of tree felling going on.  Interview 1 English: 11 - 11 (0) |
| Current ecosystem services riparian buffers > Current ecosystem services MGAP > Erosion reduction | the objective of this zone was that nothing should be done and that whatever nutrients that might arrive due to erosion should be retained.  Interview 17 English: 23 - 23 (0) |
|  | If all these things stay on the land, you wouldn't need a buffer zone because there's nothing to catch, but that is impossible because every human action on the land makes some problems in soil loss. But the erosion of soil, for example, is one of the most important problems that we have in agriculture. If you have plans to lower the soil erosion, to mitigate it, it's a very important thing that helps all the water quality in the basin.  Interview 19 English: 23 - 23 (0) |
| Current ecosystem services riparian buffers > Current ecosystem services MGAP > Wood production | So, in a way, he is not paying taxes for that area, and you can make a sustainable use of the forest, even get a logging permit.  Interview 17 English: 27 - 27 (0) |
| Current ecosystem services riparian buffers > Current ecosystem services MGAP > Nutrient retention | the objective of this zone was that nothing should be done and that whatever nutrients that might arrive due to erosion should be retained.  Interview 17 English: 23 - 23 (0)  That is why I understand that it is more important not to prohibit doing anything, but to look for a responsible use of those areas, and that they really fulfil the function of extracting nutrients or retaining nutrients.  Interview 17 English: 25 - 25 (0)  I mean, when they were implemented at the watershed level, it was to retain nutrients,  Interview 17 English: 29 - 29 (0)  In its definition the goal is to establish the objectives so that the retention of nutrients can reach a point where it begins to become saturated if you don't manage or extract it in some way.  Interview 17 English: 29 - 29 (0) |
|  | I think buffer zones are to catch the possibility of nutrients and pesticide residues or things that come from the productive land. On their trip to the river, these buffer zones catch them, so they have a very important function to impede all these nutrients and pesticides get to the water. That is the most important thing. If there were no things to get to the water, they will have no function. And it's an existing situation, so they are important to catch all the things that go from the land to the river.  Interview 19 English: 13 - 13 (0)  It needs to have species with high production, so they can consume all the nutrients that are retained in the buffer zone.  Interview 19 English: 33 - 33 (0) |
| Current ecosystem services riparian buffers > Current ecosystem services MGAP > Microclimate regulation | for shade and shelter in the forest.  Interview 17 English: 23 - 23 (0) |
| Current ecosystem services riparian buffers > Current ecosystem services MGAP > Shelter livestock | for shade and shelter in the forest.  Interview 17 English: 23 - 23 (0) |
| Current ecosystem services riparian buffers > Current ecosystem services scientists > Biodiversity | Then we also talk about riparian zones as a habitat for fauna and flora.  Interview 8 English: 7 - 7 (0) |
|  | Additionally, they have an extremely important function, which is providing a refuge for biodiversity.  Interview 9 English: 15 - 15 (0) |
|  | And then you have, I think, more of a secondary issue, which is biodiversity and natural corridors.  Interview 22 English: 9 - 9 (0) |
| Current ecosystem services riparian buffers > Current ecosystem services scientists > Corridor invasive species | There are also negative ecosystem services, such as being corridors for invasive species, which is a significant problem in the Paso Severino reservoir. If you don't have livestock, invasive species are likely to thrive.  Interview 9 English: 15 - 15 (0) |
| Current ecosystem services riparian buffers > Current ecosystem services scientists > Erosion reduction | the diffuse effect would be very large at the beginning and the erosion would be very high. Despite being less effective during this whole period of drought, the buffer zone would be essential at the onset of rainfall to control the inflow.  Interview 3 English: 36 - 36 (0) |
|  | We talk about how the riparian zone also affects erosion rates, and maintenance of the stream margins. So, it has a lot to do with geomorphology of streams and preventing soil erosion.  Interview 8 English: 7 - 7 (0) |
|  | Then, they play a role in protecting the native zone of the watercourse from erosion and other factors.  Interview 9 English: 15 - 15 (0) |
|  | Well, the function, I understand that it is fundamental, from the point of view of, I don't know if you are interested in water quality or if it goes beyond water quality, but well, in terms of water quality it is fundamental. Not only as control of point and diffuse sources, but also as control of erosion of the land itself.  Interview 18 English: 13 - 13 (0)  Primarily the control of the materials and the compounds that are coming in.  Interview 18 English: 20 - 20 (0)  Secondly, the control of other ecosystem services, such as the control of erosion of the river banks.  Interview 18 English: 20 - 20 (0) |
|  | the most important effect is to cut erosion as well, because having a vegetation layer is very important so that the sediments don't reach the bottom.  Interview 23 English: 27 - 27 (0) |
| Current ecosystem services riparian buffers > Current ecosystem services scientists > Locality-based ecosystem services | The ecosystem services they are fulfilling, I believe, are different depending on what type of areas we are considering, and the uses that are given to them. There are areas that are well conserved and are in livestock farming areas, and there are areas that have been completely modified for agricultural use. So, it is difficult to answer this question for the whole of the Santa Lucía basin. I think there are very contrasting situations.  Interview 18 English: 13 - 13 (0) |
| Current ecosystem services riparian buffers > Current ecosystem services scientists > Microclimate regulation | We talk about riparian zones as modifying the microclimate and affecting stream temperature, temperature extremes, temperature regimes.  Interview 8 English: 7 - 7 (0) |
| Current ecosystem services riparian buffers > Current ecosystem services scientists > Nutrient retention | But it would need to be a sufficient area to retain a significant percentage of the nutrients produced by agriculture in the basin, and also suspended particles. In other words, both should be retained in a way that is neither absolute nor total, but significant, with a reduction.  Interview 3 English: 14 - 14 (0)  Of course, from a chemical-biological point of view, buffers are walls where we only focus on nutrient dynamics.  Interview 3 English: 44 - 44 (0) |
|  | The movement of phosphorus with water flowing down in the surface will be contained  Interview 7 English: 17 - 17 (0)  because the phosphorus reduction in these streams will result in less phosphorus in the reservoirs from where cities are taking water for consumption.  Interview 7 English: 19 - 19 (0) |
|  | So, we talk about riparian zone function in terms of nutrient sequestration, nutrient absorption.  Interview 8 English: 7 - 7 (0) |
|  | Yes, and as also the natural field have a smaller footprint regarding nutrient level not only with us, but also for small farmers.  Interview 9 English: 5 - 5 (0)  There are some studies, not many, that show that when you have strips of vegetation interspersed with the crops, it is more efficient in retaining nutrients than when everything is limited to one buffer zone around the stream.  Interview 9 English: 13 - 13 (0)  the buffer zones fulfil the primary function of reducing the nutrients and sediments that come from more intensive land uses, such as agriculture and pastures, and to a lesser extent for the natural countryside.  Interview 9 English: 15 - 15 (0) |
|  | Well, the function, I understand that it is fundamental, from the point of view of, I don't know if you are interested in water quality or if it goes beyond water quality, but well, in terms of water quality it is fundamental. Not only as control of point and diffuse sources, but also as control of erosion of the land itself.  Interview 18 English: 13 - 13 (0)  Primarily the control of the materials and the compounds that are coming in.  Interview 18 English: 20 - 20 (0) |
|  | Well, the main function would be, as agreed at the Ministry, to protect the quality of the water by acting as a buffer or as a filter to catch runoff from agriculture.  Interview 22 English: 9 - 9 (0) |
|  | the objective is to try to intercept and that in the runoff. It was historically assumed that the cause was erosion, i.e., the sediment. This is what I recommend to you from the guy who has worked the most. He was a tall guy, but very smart. He was the first one to even adjust the different methods for measuring total phosphorus in water, which are not the same as the methods we used for soil. Well, Jasper was the first, in a way, to show that not only did phosphorus come through erosion but that there was phosphorus that was soluble, let's say, and when the water passed through, it was also carried along with that runoff water. In other words, the runoff water carried the sediments, but it also carried phosphorus  Interview 23 English: 23 - 23 (0) |
| Current ecosystem services riparian buffers > Current ecosystem services scientists > Recreation | In terms of recreation, you can also go for a swim in a stream in the summer, which today you can't do because agriculture up to the edge is not going to make you feel like getting into a stream or going fishing in a place where you are among the leaves.  Interview 9 English: 15 - 15 (0) |
| Current ecosystem services riparian buffers > Current ecosystem services scientists > Wood production | But these buffer zones could also be used for gathering fire wood.  Interview 7 English: 17 - 17 (0) |
| Current ecosystem services riparian buffers > Current ecosystem services Vida Silvestre > Biodiversity | We do think that areas have a lot of ecological ecosystem services and sociological ecosystem services that go way beyond just retaining the extra input of nutrients from agricultural activities and have to deal with maintaining a range of other ecosystem services, patches for biodiversity systems, and enjoyment. I mean, they are patches of native ecosystems that allow a very wide range of ecosystem services and ecosystem services are always depending on what the specific stakeholders are expecting from them, or are needing or are using from those patches of nature.  Interview 20 English: 11 - 11 (0)  but as I said, before that, there was already a protected area that needed management to increase biodiversity persistence and maintain the long-term ecosystem services and human well-being in that area and, so restoring patches of the natural ecosystem within that protected area has a lot of different contributions to a range of needs.  Interview 20 English: 11 - 11 (0) |
| Current ecosystem services riparian buffers > Current ecosystem services Vida Silvestre > Recreation | We do think that areas have a lot of ecological ecosystem services and sociological ecosystem services that go way beyond just retaining the extra input of nutrients from agricultural activities and have to deal with maintaining a range of other ecosystem services, patches for biodiversity systems, and enjoyment. I mean, they are patches of native ecosystems that allow a very wide range of ecosystem services and ecosystem services are always depending on what the specific stakeholders are expecting from them, or are needing or are using from those patches of nature.  Interview 20 English: 11 - 11 (0)  maintaining certain ecological processes that are key for human well-being in a sociological system.  Interview 20 English: 13 - 13 (0) |
| Current ecosystem services riparian buffers > Current ecosystem services Vida Silvestre > Nutrient retention | We do think that areas have a lot of ecological ecosystem services and sociological ecosystem services that go way beyond just retaining the extra input of nutrients from agricultural activities and have to deal with maintaining a range of other ecosystem services, patches for biodiversity systems, and enjoyment. I mean, they are patches of native ecosystems that allow a very wide range of ecosystem services and ecosystem services are always depending on what the specific stakeholders are expecting from them, or are needing or are using from those patches of nature.  Interview 20 English: 11 - 11 (0)  So, we don't see just the forest and the function of retaining nutrients, we understand we need to recover the original native ecosystem as much as possible, but also, again, the buffer zone is just a tiny strip of a larger landscape, you need to manage to maintain the provision of services and the conditions for natural ecosystems to evolve and respond to changes.  Interview 20 English: 17 - 17 (0) |
| Preferred ecosystem services riparian buffers > Preferred ecosystem services CAF > Water resource protection | They are a regulator of the flow and retain and also retain the small particles; they are very rich in phosphorus.  Interview 24 English: 5 - 5 (0) |
| Preferred ecosystem services riparian buffers > Preferred ecosystem services CAF > Nutrient retention | The other condition is to try to put the phosphorus under control, if you have the equipment you need to put the phosphorus under control and remain controlling the effluent problems.  Interview 24 English: 9 - 9 (0) |
| Preferred ecosystem services riparian buffers > Preferred ecosystem services CAF > Water flow alteration | A second try at reducing the speed of the water using the terraces of the river failed.  Interview 24 English: 9 - 9 (0) |
| Preferred ecosystem services riparian buffers > Preferred ecosystem services CEUTA > Agricultural productivity | I think it should include possibilities of agroecological management linked to the buffer zones that would be productive.  Interview 2 English: 16 - 16 (0)  It is a battery of agroecological practices that together can even improve the buffer zone, producing co-benefits to the productive system.  Interview 2 English: 16 - 16 (0) |
| Preferred ecosystem services riparian buffers > Preferred ecosystem services CNFR > Climate resilience | to prevent complicated situations from occurring, especially in times of climatic crises or climatic situations of overflow.  Interview 5 English: 20 - 20 (0) |
| Preferred ecosystem services riparian buffers > Preferred ecosystem services CNFR > Erosion reduction | and prevent soil erosion.  Interview 5 English: 20 - 20 (0) |
| Preferred ecosystem services riparian buffers > Preferred ecosystem services CNFR > Watercourse protection | prevent rivers from overflowing,  Interview 5 English: 20 - 20 (0) |
| Preferred ecosystem services riparian buffers > Preferred ecosystem services CNFR > Agricultural productivity | And to be able to develop activities, whether they are productive or rational management, is very important  Interview 5 English: 24 - 24 (0)  thinning  Interview 5 English: 24 - 24 (0) |
| Preferred ecosystem services riparian buffers > Preferred ecosystem services CNFR > Recreation | Those who like to collaborate with nature have it better than ever in those places. In other words, they also have cultural, recreational, and natural value, which is almost intangible.  Interview 5 English: 24 - 24 (0)  For me, the value goes beyond the fact that you can put an economic value on it as the world we live in demands it. There is a value that is not transferable. And that is worth gold. That, I believe, is what human beings can never lose sight of. Life is at stake.  Interview 5 English: 24 - 24 (0) |
| Preferred ecosystem services riparian buffers > Preferred ecosystem services CNFR > Natural ecosystem restoration | areas where the nature of the place can be rebuilt so that they can fulfil their ecosystem services.  Interview 5 English: 20 - 20 (0)  let things be the way they naturally are and give up part of the productive area to avoid running the risk of generating problems as serious as those that are being generated.  Interview 5 English: 20 - 20 (0) |
| Preferred ecosystem services riparian buffers > Preferred ecosystem services CNFR > Shade for livestock | I've seen areas cleared, not in that area, but in other places, some people destroy the forest and then go around planting trees so that the animals have shade.  Interview 5 English: 24 - 24 (0) |
| Preferred ecosystem services riparian buffers > Preferred ecosystem services DINAGUA > Erosion reduction | their most important function in Uruguay is to act as a barrier to mitigate the transport of nutrients through erosion.  Interview 6 English: 20 - 20 (0) |
| Preferred ecosystem services riparian buffers > Preferred ecosystem services DINAGUA > Nutrient retention | The function was to mitigate the impact of runoff, of nutrients, in the watercourse.  Interview 6 English: 10 - 10 (0)  their most important function in Uruguay is to act as a barrier to mitigate the transport of nutrients through erosion.  Interview 6 English: 20 - 20 (0)  retention function of the buffer zone.  Interview 6 English: 27 - 27 (0) |
| Preferred ecosystem services riparian buffers > Preferred ecosystem services DINAGUA > Water flow alteration | regulation of water quantity.  Interview 6 English: 24 - 24 (0) |
| Preferred ecosystem services riparian buffers > Preferred ecosystem services DINAGUA > Water recharge | In short, what they are trying to do is to protect the water harvesting zones with the function of recharge.  Interview 6 English: 24 - 24 (0) |
| Preferred ecosystem services riparian buffers > Preferred ecosystem services DINAGUA > Water resources protection | This buffer zone is for the protection of water sources and should also be implemented for the protection of sources used for consumer use and irrigation, etc.  Interview 6 English: 22 - 22 (0) |
| Preferred ecosystem services riparian buffers > Preferred ecosystem services DINAMA > Biodiversity | conserving the areas that have priority from the point of view of the biodiversity of species.  Interview 12 English: 15 - 15 (0) |
| Preferred ecosystem services riparian buffers > Preferred ecosystem services DINAMA > Biological corridor | It can also have other ecosystem services such as biological corridors, but our design is not primarily intended for that purpose.  Interview 10 English: 17 - 17 (0) |
| Preferred ecosystem services riparian buffers > Preferred ecosystem services DINAMA > Natural ecosystem restoration | Other measures, such as restoration (of native forests, red.) in some parts, are also being considered.  Interview 12 English: 13 - 13 (0) |
| Preferred ecosystem services riparian buffers > Preferred ecosystem services DINAMA > Nutrient retention | , we think that it should serve as a pollutant filter.  Interview 10 English: 17 - 17 (0) |
| Preferred ecosystem services riparian buffers > Preferred ecosystem services DINAMA > Agricultural productivity | possibly with a wider range of land use. This might include activities such as grazing or harvesting, but we are still discussing the specifics.  Interview 10 English: 21 - 21 (0)  We may allow some uses that do not go against nature in any way.  Interview 10 English: 26 - 26 (0)  there is the question that it should also benefit the producer.  Interview 10 English: 34 - 34 (0) |
|  | The issue is that the producer may lose an (productive, red.) area when applying this buffer zone.  Interview 12 English: 17 - 17 (0) |
| Preferred ecosystem services riparian buffers > Preferred ecosystem services DINOT > Agricultural productivity | It would still have a productive purpose, but within the context of a use and management plan for that land with its own activities.  Interview 21 English: 21 - 21 (0)  However, in the buffer zones in some parts, you can do productive activities, and things can happen, it is not that it is a zone that has no profitability. Obviously, this goes for touristic, productional, and recreational activities.  Interview 21 English: 32 - 32 (0) |
| Preferred ecosystem services riparian buffers > Preferred ecosystem services DINOT > Recreation | However, in the buffer zones in some parts, you can do productive activities, and things can happen, it is not that it is a zone that has no profitability. Obviously, this goes for touristic, productional, and recreational activities.  Interview 21 English: 32 - 32 (0) |
| Preferred ecosystem services riparian buffers > Preferred ecosystem services DINOT > Tourism | However, in the buffer zones in some parts, you can do productive activities, and things can happen, it is not that it is a zone that has no profitability. Obviously, this goes for touristic, productional, and recreational activities.  Interview 21 English: 32 - 32 (0) |
| Preferred ecosystem services riparian buffers > Preferred ecosystem services INIA > Recreation | Yes, it's a visual aspect. For the vegetation cover, it is particularly important. For many people it's important as they believe it is essential. I'm not sure about younger people, but probably yes.  Interview 15 English: 35 - 35 (0) |
| Preferred ecosystem services riparian buffers > Preferred ecosystem services INIA > Biodiversity | From the perspective of the third function, which is the conservation of biodiversity and the connectivity of ecosystems, it is vital to maintain diversity.  Interview 15 English: 31 - 31 (0) |
| Preferred ecosystem services riparian buffers > Preferred ecosystem services INIA > Nature protection | the goal is to restore the area to its natural state,  Interview 15 English: 31 - 31 (0) |
| Preferred ecosystem services riparian buffers > Preferred ecosystem services INIA > Nutrient retention | Yes, that's precisely why I emphasized the issue of diversity and structure. We can plant a buffer zone with Paspalum notatum, which creates a physical barrier and consumes nutrients in summer.  Interview 15 English: 29 - 29 (0) |
| Preferred ecosystem services riparian buffers > Preferred ecosystem services MGAP > Livestock productivity | From a production point of view, having water in sufficient quantity and quality is beneficial for animal welfare and even for production, regarding the way the animals graze.  Interview 17 English: 38 - 38 (0) |
| Preferred ecosystem services riparian buffers > Preferred ecosystem services MGAP > Agricultural productivity | And then you can plant or sow, I don't know how to say it, some productive species, and maybe with good management, producers can use it for cattle alimentation, for example. Because if they're in the buffer zone, it's grass that doesn't produce. For example, in native grass, maybe there is a risk of accumulation, so a lot of nitrogen, for example, or phosphorus is there, and sooner or later, maybe that phosphorus ends at the river. So, if you plant there, for example, a pasture with high production, that pasture can consume those nutrients, and maybe the farmers can use it to feed their cattle. Maybe in a way so that the cattle don't excrete at the buffer zone, maybe getting into the buffer zone like four or five hours a day, not every day and all the day, that they can use that buffer zone. Maybe they can do it that way.  Interview 19 English: 15 - 15 (0) |
| Preferred ecosystem services riparian buffers > Preferred ecosystem services MGAP > Watercourse protection | So, when it comes to thinking about the buffer zone or the integrated management of the property with respect to that buffer zone, there are many things that can be done without being too extreme, like fencing it off, like prohibiting the use.  Interview 17 English: 33 - 33 (0) |
| Preferred ecosystem services riparian buffers > Preferred ecosystem services producers > Wood production | at least I would need to be able to exploit a little bit more of what is forest.  Interview 14 English: 88 - 88 (0) |
| Preferred ecosystem services riparian buffers > Preferred ecosystem services producers > Shade for livestock | So, the ecosystem of the trees with the pasture below for the cattle is wonderful. And I'm particularly interested in preserving it because it provides comfort for the livestock in both winter and summer. It's a very sheltered area in winter, and it's cool in summer because of the shade. Even at the driest times, there is grass. In other words, the combination of trees and pasture is great. I want to preserve it and improve it because it has direct benefits for the livestock, without a doubt.  Interview 4 English: 27 - 27 (0) |
| Preferred ecosystem services riparian buffers > Preferred ecosystem services producers > Animal welfare | So, the ecosystem of the trees with the pasture below for the cattle is wonderful. And I'm particularly interested in preserving it because it provides comfort for the livestock in both winter and summer. It's a very sheltered area in winter, and it's cool in summer because of the shade. Even at the driest times, there is grass. In other words, the combination of trees and pasture is great. I want to preserve it and improve it because it has direct benefits for the livestock, without a doubt.  Interview 4 English: 27 - 27 (0) |
| Preferred ecosystem services riparian buffers > Preferred ecosystem services producers > Biodiversity | Another one is for the birds. I think that's a good idea.  Interview 4 English: 52 - 52 (0) |
|  | I think that in the end, the essential thing is biodiversity and soil protection.  Interview 13 English: 29 - 29 (0) |
| Preferred ecosystem services riparian buffers > Preferred ecosystem services producers > Agricultural productivity | if you conserve the native forest, thinking of a silvopasture management  Interview 13 English: 15 - 15 (0)  In my case, I try to differentiate the environments. That is to say, the property has different environments, and according to the environment, one can make an active productive theme, and in certain environments, another one. So, in reality, I kind of accept certain areas where it will not be productive, to try and make sustainable management efforts. I mean, you also have to differentiate between different degrees of buffer zones.  Interview 13 English: 19 - 19 (0)  The animal does not enter the riparian forest, so the animal does not take water from the river. Then, I have water distribution in the plot through which water arrives. Now I am waiting for them to come and fix a pipe that broke, so I have all the cattle concentrated here. But there are degrees of buffer areas. There are buffer and restricted areas where the cattle do not enter, where nothing is done in the riparian forest, and then you have the formation that I call the “park type”, where normally there is no water. I have three levels: the riparian forest, then I have the level of the park-like forest, and then I have what I call a “bajo” (meaning: low, red.), which is the twelve three hectares where there are practically no trees, which is a field that I manage. In the natural area, I add another one, which is a kind of summer pasture. I don't know if you understood the concept; they are three different buffer areas or three different degrees of buffer intensity.  Interview 13 English: 19 - 19 (0) |
|  | If they (the buffer zones) stayed there, I could take advantage of them, even if they were stationary, without movement, or anything. It limits the field, that's all.  Interview 14 English: 31 - 31 (0)  And it would be advantageous to be able to let it become pasture, something more usable.  Interview 14 English: 62 - 62 (0) |
| Preferred ecosystem services riparian buffers > Preferred ecosystem services producers > Recreation | Look, the Santa Lucia River is a river that is very popular for canoeing and other recreational activities. I think it's great. And the way we can all have that is good, I think it's more enjoyable for everyone.  Interview 4 English: 52 - 52 (0)  The benefits of these changes are numerous and include a more aesthetically pleasing environment, a decrease in the economic loss of grazing areas, and the prevention of access by animals to restricted areas.  Interview 4 English: 54 - 54 (0) |
|  | Now, the buffer zone provides cultural and recreational benefits, as well as protection for the river, watercourse, and soil.  Interview 13 English: 45 - 45 (0) |
| Preferred ecosystem services riparian buffers > Preferred ecosystem services producers > Erosion reduction | I think that in the end, the essential thing is biodiversity and soil protection.  Interview 13 English: 29 - 29 (0) |
| Preferred ecosystem services riparian buffers > Preferred ecosystem services producers > Water resource protection | the preservation of the environment, taking care of the water resource from the point of view of human consumption  Interview 16 English: 35 - 35 (0)  Well, if there were interventions, I don't think it would contribute more than it does now, right? It's the environmental point of view and what I insist on with water. As long as, and I'm being heavy-handed with this, the buffer area remains specifically the area of high moisture content. If not, it wouldn't be beneficial.  Interview 16 English: 55 - 55 (0) |
| Preferred ecosystem services riparian buffers > Preferred ecosystem services scientists > Biodiversity | And the other utility is biodiversity, right? Trying to recover the biodiversity of not only the river itself but of a margin of flooding area, which is in continuous contact with the water.  Interview 3 English: 17 - 17 (0) |
|  | and also, the biodiversity in these areas will be very important  Interview 7 English: 19 - 19 (0)  he buffers zone near the river should be the natural vegetation that always grew there, because these plants are adapted to the ecology of the region  Interview 7 English: 21 - 21 (0) |
|  | I think these are one of the most useful things you can do in terms of a myriad of ecosystem services that go from biodiversity to carbon sequestration to, I don't know, protected river banks, etc.  Interview 22 English: 11 - 11 (0) |
| Preferred ecosystem services riparian buffers > Preferred ecosystem services scientists > Carbon sequestration | I think these are one of the most useful things you can do in terms of a myriad of ecosystem services that go from biodiversity to carbon sequestration to, I don't know, protected river banks, etc.  Interview 22 English: 11 - 11 (0) |
| Preferred ecosystem services riparian buffers > Preferred ecosystem services scientists > Ecosystem connectivity | it also ecosystem services as a biological corridor, that is to say, to recover the system from an environmental point of view  Interview 3 English: 17 - 17 (0) |
| Preferred ecosystem services riparian buffers > Preferred ecosystem services scientists > Water recharge | They saw that rooting depth caused the entry of the water from the stream at depth into the soil,  Interview 3 English: 15 - 15 (0) |
| Preferred ecosystem services riparian buffers > Preferred ecosystem services scientists > Limiting agricultural frontier | But also, an important function that I see is that once you plant trees, in a way you reclaim the land from agriculture. So, they are like, you know, a natural limit to agriculture, to certain types of agricultural activity,  Interview 22 English: 11 - 11 (0) |
| Preferred ecosystem services riparian buffers > Preferred ecosystem services scientists > Nutrient retention | whereas the more shrubby or grassy areas retained a lot of suspended particles.  Interview 3 English: 15 - 15 (0) |
|  | Because it will retain the available peak and the leaching.  Interview 7 English: 17 - 17 (0) |
|  | it seems to me like the most important role is creating that buffer of vegetation and soil microbes that are able to capture, sequester and process these different forms of nutrients before they get to the water. That seems like goal number one.  Interview 8 English: 15 - 15 (0) |
|  | So, I think that would be the priority and that involves, yeah, like a myriad of ecological ecosystem services that can go from what, like, filter, nutrient filtering or chemical filtering to, I don't know, even shadow over the rivers to cool them off.  Interview 22 English: 25 - 25 (0) |
| Preferred ecosystem services riparian buffers > Preferred ecosystem services scientists > Agricultural productivity | the type of cultivation that is done on another strip above the buffer zone, which in many cases the in the U.S. is unfertilized areas, which are harvested to remove nutrients from that site.  Interview 3 English: 15 - 15 (0) |
|  | The only thing that I can say is that I have seen experiences like in the United States where people try to have buffer zones that also have some kind of economic benefit. Like it might be a zone where you can't plant crops, but you can plant trees that have some other positive economic impact.  Interview 8 English: 17 - 17 (0)  You could have species that also have some kind of economic value. I mean, native and exotic species is a big deal in the Santa Lucia basin as well as many other regions. So as much as you could have riparian buffers that weren't dominated by exotic species, that would also be beneficial.  Interview 8 English: 19 - 19 (0) |
|  | And then I also think that buffer zones have to be have to have management adapted to the environmental realities of the ecosystems and the productive uses of each locality.  Interview 9 English: 13 - 13 (0) |
|  | they could also fulfil ecosystem services of supplying raw materials or playing some other role,  Interview 18 English: 20 - 20 (0) |
| Preferred ecosystem services riparian buffers > Preferred ecosystem services scientists > Recreation | And you would try to promote some sort of tourism, doing some basic infrastructure, you know, and people trying to use those places for recreational use.  Interview 22 English: 21 - 21 (0) |
| Preferred ecosystem services riparian buffers > Preferred ecosystem services scientists > Watercourse protection | I think these are one of the most useful things you can do in terms of a myriad of ecosystem services that go from biodiversity to carbon sequestration to, I don't know, protected river banks, etc.  Interview 22 English: 11 - 11 (0) |
| Preferred ecosystem services riparian buffers > Preferred ecosystem services scientists > Sediment retention | whereas the more shrubby or grassy areas retained a lot of suspended particles.  Interview 3 English: 15 - 15 (0) |
| Preferred ecosystem services riparian buffers > Preferred ecosystem services scientists > Erosion reduction | There would have to be recovery of soil structure. I mean, you can't just have the vegetation without having the bacteria that are going to be playing those roles.  Interview 8 English: 19 - 19 (0) |
| Preferred ecosystem services riparian buffers > Preferred ecosystem services scientists > Microclimate regulation | So, I think that would be the priority and that involves, yeah, like a myriad of ecological ecosystem services that can go from what, like, filter, nutrient filtering or chemical filtering to, I don't know, even shadow over the rivers to cool them off.  Interview 22 English: 25 - 25 (0) |
| Preferred ecosystem services riparian buffers > Preferred ecosystem services Vida Silvestre > Accounting for both private and collective interests | put in biodiversity conservation and ecosystem service maintenance as a goal that has to be achieved as well as other productive objectives, taking into account both private initiatives and interests and collective initiatives.  Interview 20 English: 19 - 19 (0) |
| Preferred ecosystem services riparian buffers > Preferred ecosystem services Vida Silvestre > Biodiversity | put in biodiversity conservation and ecosystem service maintenance as a goal that has to be achieved as well as other productive objectives, taking into account both private initiatives and interests and collective initiatives.  Interview 20 English: 19 - 19 (0) |
| Preferred ecosystem services riparian buffers > Preferred ecosystem services Vida Silvestre > Nature protection | Yeah, well, that's the reason why we propose creating a protected area.  Interview 20 English: 19 - 19 (0) |
| Current characteristics riparian buffers > Current characteristics CAF > Grassy vegetation | Keep the areas where you collect the water with grass or with a cover that reduces the flow of the water, and keep the areas near the river without the soil crops.  Interview 24 English: 5 - 5 (0) |
| Current characteristics riparian buffers > Current characteristics CEUTA > Native vegetation | But also in these strips, we proposed as far as possible to enrich these strips with native flora that is strongly adapted to local circumstances, and that has the capacity to maintain a deep rooting depth and also increase the volume of roots per square meter.  Interview 2 English: 9 - 9 (0)  and generate fruit (i.e., enriching the biodiversity of the buffer zone). It is important that the floristic origin of the species should be linked with the basin in which the work is being done.  Interview 2 English: 9 - 9 (0)  we also included the enrichment with native woody species that were more hydrophilic with the profile closer to the watercourse, to support the riverbank.  Interview 2 English: 9 - 9 (0) |
| Current characteristics riparian buffers > Current characteristics CEUTA > No-management zone | Currently, I think what's implemented is a very zone-based scheme of the riparian buffer zones in which an area is delimited and exempt from human management. I don't know if in the end if there is an issue of mowing, but basically, they (the riparian buffers, red.) are surfaces that are protected from soil management.  Interview 2 English: 7 - 7 (0) |
| Current characteristics riparian buffers > Current characteristics CEUTA > Vegetative rooting depth | and that has the capacity to maintain a deep rooting depth and also increase the volume of roots per square meter.  Interview 2 English: 9 - 9 (0) |
| Current characteristics riparian buffers > Current characteristics CEUTA > Vegetation density | Then, in addition, at one point we also worked on the possibility enrich the vegetation to increase the density of individuals per square meter  Interview 2 English: 9 - 9 (0) |
| Current characteristics riparian buffers > Current characteristics CNFR > Decreased fertiliser use | start using less fertiliser in one place  Interview 5 English: 16 - 16 (0) |
| Current characteristics riparian buffers > Current characteristics CNFR > Native vegetation | the vegetation that is nurtured there; its flowers and fauna  Interview 5 English: 18 - 18 (0) |
| Current characteristics riparian buffers > Current characteristics DINAGUA > Dimensions | They determined that 20 metres, 50 metres, 75 metres, was a reasonable length.  Interview 6 English: 10 - 10 (0) |
| Current characteristics riparian buffers > Current characteristics DINAGUA > No-tillage management | The ban on tilling was implemented  Interview 6 English: 20 - 20 (0) |
| Current characteristics riparian buffers > Current characteristics DINAGUA > Reduction agricultural production | some producers are going to be affected by a potential reduction in their production.  Interview 6 English: 10 - 10 (0) |
| Current characteristics riparian buffers > Current characteristics DINAMA > Dimensions | The width of the buffer zone in Uruguay varies according to the sub-basin's load and what is expected to arrive (in terms of sediments and nutrients, red.).  Interview 12 English: 11 - 11 (0) |
| Current characteristics riparian buffers > Current characteristics DINAMA > Elimination exotic species | One of the few things we allow is the elimination of exotic species  Interview 10 English: 15 - 15 (0) |
| Current characteristics riparian buffers > Current characteristics DINAMA > Native vegetation | The natural (e.g., native, red.) vegetation is the most suitable for this purpose  Interview 12 English: 11 - 11 (0) |
| Current characteristics riparian buffers > Current characteristics DINAMA > Natural soil structure | In order to retain contaminants, we believe the buffer zone should maintain good soil conditions, specifically a natural soil structure.  Interview 10 English: 15 - 15 (0) |
|  | The lowlands are very degraded  Interview 12 English: 13 - 13 (0) |
| Current characteristics riparian buffers > Current characteristics DINAMA > No-management zone | it should be a strip with no intervention,  Interview 10 English: 15 - 15 (0)  we define the buffer zone as an untouched area without tilling the soil  Interview 10 English: 15 - 15 (0)  or using agrochemicals.  Interview 10 English: 15 - 15 (0) |
|  | The regulation prohibits any modifications to be made in the buffer zones, including agrochemical application, modification of vegetation cover, and soil loosening.  Interview 12 English: 5 - 5 (0)  They are not allowed to make modifications to the buffer zone or apply agrochemicals or tillage in these areas.  Interview 12 English: 9 - 9 (0)  and there is no management of the buffer zone to improve the uptake of nutrients or solids.  Interview 12 English: 11 - 11 (0) |
| Current characteristics riparian buffers > Current characteristics DINAMA > No-management zone > Prohibition productive activity | Currently, the regulations don't permit it, so legally, it cannot be done.  Interview 12 English: 21 - 21 (0) |
| Current characteristics riparian buffers > Current characteristics DINAMA > No-management zone > Access cattle | Ideally, there should be no livestock in the buffer zones, but in practice, there is often livestock grazing in these areas.  Interview 12 English: 11 - 11 (0) |
| Current characteristics riparian buffers > Current characteristics DINOT > Geomorphology-based designs | inputs, soil characteristics, land cover, soil types, geomorphology, biodiversity,  Interview 21 English: 13 - 13 (0) |
| Current characteristics riparian buffers > Current characteristics DINOT > Inputs | inputs, soil characteristics, land cover, soil types, geomorphology, biodiversity,  Interview 21 English: 13 - 13 (0) |
| Current characteristics riparian buffers > Current characteristics DINOT > Land cover | inputs, soil characteristics, land cover, soil types, geomorphology, biodiversity,  Interview 21 English: 13 - 13 (0) |
| Current characteristics riparian buffers > Current characteristics DINOT > Soil characteristics | inputs, soil characteristics, land cover, soil types, geomorphology, biodiversity,  Interview 21 English: 13 - 13 (0) |
| Current characteristics riparian buffers > Current characteristics INIA > Access cattle to watercourse | There are no restrictions on livestock entering watercourses, for example, except by decree around the Severino Pass dam and some watercourses of the Santa Lucia River, but that is very recent. The rest of the cattle have free access.  Interview 15 English: 21 - 21 (0)  And where there are no cattle in the water, the tributaries of the river have either agricultural or dairy activity, which is quite intensive.  Interview 15 English: 21 - 21 (0) |
| Current characteristics riparian buffers > Current characteristics INIA > Prohibition cultivation | they require a certain amount of ground cover; they should not be cultivated and so on.  Interview 15 English: 21 - 21 (0)  But I think that compliance with this is irregular.  Interview 15 English: 21 - 21 (0) |
| Current characteristics riparian buffers > Current characteristics INIA > Soil cover | It was designed for basic training purposes, but it involves evaluating characteristics that have enough scientific information to contribute to the improvement of water quality. These include vegetation, certain structures, more soil cover, prevention of gully erosion, and many other factors that may not even be directly related.  Interview 15 English: 15 - 15 (0)  they require a certain amount of ground cover; they should not be cultivated and so on.  Interview 15 English: 21 - 21 (0) |
| Current characteristics riparian buffers > Current characteristics INIA > Vegetation structure | We also consider the vegetation structure, ground cover, and other factors.  Interview 15 English: 13 - 13 (0)  It was designed for basic training purposes, but it involves evaluating characteristics that have enough scientific information to contribute to the improvement of water quality. These include vegetation, certain structures, more soil cover, prevention of gully erosion, and many other factors that may not even be directly related.  Interview 15 English: 15 - 15 (0)  they require a certain amount of ground cover; they should not be cultivated and so on.  Interview 15 English: 21 - 21 (0)  For me, the structure of the vegetation is very important, which is something that is not normally given much importance. The structure, not in terms of the structure of biodiversity as the interrelationships between the components, but in terms of the presence of the important plant strata, low vegetation, medium vegetation, trees where appropriate, because not all buffer zones here are the same.  Interview 15 English: 23 - 23 (0)  For me, the important thing is the structure of the vegetation: good cover and diversity, because if it is only cover  Interview 15 English: 23 - 23 (0) |
| Current characteristics riparian buffers > Current characteristics INIA > Vegetation structure > Grassy vegetation | Today, most of them have grassland  Interview 15 English: 23 - 23 (0) |
| Current characteristics riparian buffers > Current characteristics MGAP > Dimensions | But I think we have, for example, a defined buffer zone in Santa Lucía is 100 metres from the watercourse of the Paso Severino Lake, San Francisco and Canelón Grande. We are talking about 100 metres, the area, the property, the producer. In other words, it is being defined not to use an important percentage of the area of a farm.  Interview 17 English: 23 - 23 (0) |
| Current characteristics riparian buffers > Current characteristics MGAP > Locality-based buffer zones | And well, in the places where there is no native forest or riverine forest any management that is done has to think in terms of the according to the characteristics of the basin. For instance, to look for what crops to grow. In other words, to be able to first detect what the problems are, what nutrients are arriving and what to plant to extract them and how to harvest them. Also, regarding nutrient supply, fertilisation, and with agrochemical management.  Interview 17 English: 31 - 31 (0)  What I gather from it is that each farm is going to have its own variant. There is no recipe. More than each property, you have to know if there is native forest or not, whether there are wetlands or not.  Interview 17 English: 31 - 31 (0) |
| Current characteristics riparian buffers > Current characteristics MGAP > No access cattle to watercourse | The other thing that I remember at the river basin commission is that the cattle were not able to get into the river  Interview 19 English: 15 - 15 (0) |
| Current characteristics riparian buffers > Current characteristics MGAP > No-management area | Buffer zones were defined where absolutely nothing could be done.  Interview 17 English: 23 - 23 (0) |
| Current characteristics riparian buffers > Current characteristics MGAP > No-management area > Prohibition agrochemicals | For example, for buffer zones, which is actually the safe distance for the application of agrochemicals, which is led by the General Directorate of Agricultural Services, or any other area where care must be taken.  Interview 17 English: 9 - 9 (0)  From the point of view, for example, of agrochemical applications, there is a regulation that prohibits application within 10 metres of the watercourse.  Interview 17 English: 33 - 33 (0) |
| Current characteristics riparian buffers > Current characteristics MGAP > Productive vegetative species | Because of that, it's very important to choose which spices or vegetables to have there, because all these nutrients are going to accumulate in that buffer zone. And once they are there, or nutrients are there, they are accumulated. You have to consider what species, with what growth, at what time of the year, you know, to have in that buffer zone. Do you understand?  Interview 19 English: 13 - 13 (0)  I think that there have to be plants that have a lot of production, because if you have, for example, phosphorus and nitrogen, you need a plant that uses it, so the accumulation there is not very high.  Interview 19 English: 15 - 15 (0)  It needs to have species with high production, so they can consume all the nutrients that are retained in the buffer zone.  Interview 19 English: 33 - 33 (0)  I imagine crops or, or plants that are high, dense, and with high productivity that can grow with all the nutrients that we have there at the buffer zone.  Interview 19 English: 33 - 33 (0) |
| Current characteristics riparian buffers > Current characteristics MGAP > Woody vegetation | Most of our watercourses are bordered by native riverine forest. In other words, in most cases we have natural protection where there is none.  Interview 17 English: 23 - 23 (0)  there are areas near the watercourse where the forest area has been altered and reduced.  Interview 17 English: 23 - 23 (0)  in the buffer zones, in the Paso Severino Lake, there was an initiative on the part of a citizen to plant native trees.  Interview 17 English: 29 - 29 (0)  What I do emphasise is that most of our watercourses have an area of native forest that is protecting the river. If they're in good condition, if there's vegetation growing, if there's is crashing nutrients if they are reaching that area.  Interview 17 English: 31 - 31 (0) |
| Current characteristics riparian buffers > Current characteristics producers > Access cattle to buffer zone | In the buffer zone, you can graze without moving the land, right.  Interview 14 English: 7 - 7 (0)  There are about 18 hectares of native woodland, which we don't touch more than with the cattle. It's not without cattle grazing at all, but they don't move or anything like that.  Interview 14 English: 25 - 25 (0)  today it has the purpose that the cattle enter at least up to there, up to the fence.  Interview 14 English: 41 - 41 (0)  I don't think so. It even favours those who are in the field. I don't know, I think it favours us in terms of grazing without ploughing.  Interview 14 English: 52 - 52 (0) |
| Current characteristics riparian buffers > Current characteristics producers > Dense woody vegetation | For two reasons, because it's very dense and dirty,  Interview 14 English: 33 - 33 (0) |
| Current characteristics riparian buffers > Current characteristics producers > Dimensions | For now, we are focusing on promoting and studying the issue of limiting land use to a margin of 40, 50, 100 meters, so that neither ploughing nor fertilizer application can take place and it ecosystem services as a natural barrier.  Interview 11 English: 11 - 11 (0) |
|  | Word of mouth has oriented us there, but I'm not sure about the exact number of meters. Some say 70, others say 50, and some say 100. It varies depending on the location.  Interview 14 English: 7 - 7 (0) |
|  | and from a productive standpoint, we work to the extent that we don't respect those 150 meters that have been established. Yes, I thought that it wasn't regulated.  Interview 16 English: 13 - 13 (0)  Well, here, it's 20 meters wide in some parts, and there are others that are hundreds or 50 meters wide, where there are two wetlands that are hidden.  Interview 16 English: 41 - 41 (0) |
| Current characteristics riparian buffers > Current characteristics producers > Exotic vegetation | We have some invasive species.  Interview 4 English: 15 - 15 (0) |
|  | There are some invasive species that you can identify, such as the Sarsamora and the Ligustro (Ligustrum lucidum). The Ligustro moves forward in the field and expands. It is considered invasive here, but it doesn't bother us in the riverbed. We don't do any kind of management with the ligustrum or anything else. What I fight against a little bit is the Zarzamora that is coming up in the field.  Interview 14 English: 43 - 43 (0)  Just because they can't fight it, it's a wasted field. If you can't fight it with a tool or whatever, it's a lost field. It's strange that you can't make it productive.  Interview 14 English: 68 - 68 (0)  Over the years, the situation has been getting worse because we are all here but don't dare to touch it. There was a rumour that we couldn't even use a rotavator, so we couldn't use it on the grasslands or keep it clean with weedkillers.  Interview 14 English: 90 - 90 (0) |
| Current characteristics riparian buffers > Current characteristics producers > Fencing | With regard to management, we have the obligation to have everything fenced so that the cattle cannot go to the river.  Interview 4 English: 5 - 5 (0)  it's very dirty. I have two boundaries, at the wire fences with my neighbours, that we have to fix all the time and so on.  Interview 4 English: 9 - 9 (0) |
|  | A 15-meter line was established on the banks of the streams, in which the producer had to put a wired fence or a movable wired fence, limiting the access of the cattle directly.  Interview 11 English: 13 - 13 (0) |
| Current characteristics riparian buffers > Current characteristics producers > Grassy vegetation | they are areas where there is special vegetation, with a lot of grass throughout the year, and that is what protects them.  Interview 16 English: 11 - 11 (0)  They are important grasslands, not just for grazing, but also for generating the typical humidity of the area.  Interview 16 English: 19 - 19 (0)  and it would look more like this photo you have, with vegetation, water, and no trees. And no cows either.  Interview 16 English: 33 - 33 (0) |
| Current characteristics riparian buffers > Current characteristics producers > No-tillage management | For now, we are focusing on promoting and studying the issue of limiting land use to a margin of 40, 50, 100 meters, so that neither ploughing nor fertilizer application can take place and it ecosystem services as a natural barrier.  Interview 11 English: 11 - 11 (0) |
|  | In the buffer zone, you can graze without moving the land, right.  Interview 14 English: 7 - 7 (0)  There are about 18 hectares of native woodland, which we don't touch more than with the cattle. It's not without cattle grazing at all, but they don't move or anything like that.  Interview 14 English: 25 - 25 (0) |
|  | we don't touch the buffer zones.  Interview 16 English: 25 - 25 (0)  according to my interpretation, is to determine rules in which you cannot do a huge number of things, including moving the earth.  Interview 16 English: 57 - 57 (0) |
| Current characteristics riparian buffers > Current characteristics producers > No access cattle to watercourse | I think cattle shouldn't be allowed to move within the Santa Lucia Chico river margin, but I'm not sure.  Interview 14 English: 7 - 7 (0)  There are reservoirs in the field. Yes, we use them in case of long dry spells like now. We bring water to them with a tank that has a pump. That's where it goes, and it's distributed in the field. I extract 3000 litres, put it in the tanks, but they don't have access to the river. That's how it is. I can't give them access, because sometimes I find them and sometimes I don't. That's the problem.  Interview 14 English: 35 - 35 (0) |
|  | And for example, the cattle don't have access to that area, right?  Interviewee: No, because for much of the year, when it's very wet, we're talking about fractions of 8 hectares, so we couldn't do much. But since the soil is very soft, the cattle end up causing much worse damage than if I were to use the land. So, we try to keep it as clean as possible, without too many interventions, precisely because they are small units.  Interview 16 English: 16 - 17 (0) |
| Current characteristics riparian buffers > Current characteristics producers > No cultivation | You can't farm there. I don't do any activity in that whole area and therefore I don't do anything in the buffer either.  Interview 4 English: 7 - 7 (0) |
|  | it seems that buffer zones are very natural areas without cultivation.  Interview 16 English: 31 - 31 (0) |
| Current characteristics riparian buffers > Current characteristics producers > Water streams and reservoirs | and it would look more like this photo you have, with vegetation, water, and no trees. And no cows either.  Interview 16 English: 33 - 33 (0) |
| Current characteristics riparian buffers > Current characteristics scientists > Dimensions | the buffers at the reservoirs should be 100 meters long  Interview 3 English: 11 - 11 (0)  the dimensions  Interview 3 English: 15 - 15 (0) |
|  | But in the Santa Lucia, I know that they ended up having an area where they just excluded cattle at 100 meters with a fence due to whatever densities that I suppose were high enough that it was still causing erosion and whatever undesirable effects the cattle had on the functioning of the riparian zone.  Interview 8 English: 17 - 17 (0) |
|  | For me the idea would be that every time you have a crop, its transition zone or its crop boundary would have buffers in between. You could always have on the entirety of the slope interspersed with the buffer zone, and that could be forests, pastures, whatever you want and that would be much more efficient than waiting here for a buffer zone of only 20, 30, 50 metres along a watercourse.  Interview 9 English: 13 - 13 (0) |
| Current characteristics riparian buffers > Current characteristics scientists > Exclusion cattle to buffer zone | for a part of the reservoirs the entry of livestock was prevented.  Interview 3 English: 11 - 11 (0) |
|  | the cattle are going to the water to drink water.  Interview 7 English: 17 - 17 (0) |
|  | In other words, we have come across areas where, in theory, cattle cannot enter, where there are 200 cattle that are grazing directly on the course.  Interview 18 English: 15 - 15 (0) |
|  | I agree with all the cattle, no. Because the cattle are the first mess that comes into the buffer. You know it's because when cattle go through their feet, through a watercourse, they throw their dung because, when they feel the cold water, it's like a natural reaction. The cattle loosen up, don't they? There are works that prove it.  Interview 23 English: 15 - 15 (0) |
| Current characteristics riparian buffers > Current characteristics scientists > Exclusion cattle to buffer zone > Fencing | In one area, they put fences in our reservoir  Interview 7 English: 17 - 17 (0) |
|  | So, when the OSE goes there and the Minister of Environment wants to implement this buffer, what they did was put a wire all around their lands,  Interview 22 English: 21 - 21 (0) |
| Current characteristics riparian buffers > Current characteristics scientists > Grassy vegetation | I was on the committee of a study that looked at how different vegetation covers affect these different roles. So, one kind of mistake is that we might think that it's mostly forests that are playing a key role in all of these ecosystem services, but so far what the research is finding is that you can also have grassland cover, native grasslands, and obviously they don't behave exactly the same way as forests do with tree cover, but they do play a critical role in capturing sediments.  Interview 8 English: 9 - 9 (0) |
| Current characteristics riparian buffers > Current characteristics scientists > Irregular implementation | What we and our colleague Gilles Aguichet-Morsellado were suggesting was that in many cases the flood zones were not being included. That is to say that the buffer zone in many cases, in which it is obviously very complex to map a zone of this type in a flood zone, was not included. So, we proposed that the buffer zone had to be outside the flood zone and that the flood zone was a zone of its own.  Interview 3 English: 9 - 9 (0)  That there are so many gaps between its implementation in the field. As a zone, it is too permeable.  Interview 3 English: 11 - 11 (0)  . From what they told me, there has been no intensive monitoring of these places, nor have there been any fines. No fines, no warnings, I don't know. I have the feeling that these buffer zones, and their control, have been abandoned.  Interview 3 English: 11 - 11 (0)  Each producer makes their buffer zone if they want to, or not.  Interview 3 English: 11 - 11 (0)  it has been a bad implementation that has fallen by the wayside. It has been abandoned, at least, that's my opinion  Interview 3 English: 14 - 14 (0)  In some buffer zones, the vegetation is actively reclaimed, in others, it is left to recover.  Interview 3 English: 15 - 15 (0)  what I saw of the Santa Lucía river basin was that it was a buffer zone with many punctual inflows (causing point sources, red)  Interview 3 English: 15 - 15 (0) |
| Current characteristics riparian buffers > Current characteristics scientists > No-tillage management | no-tillage planting management practice  Interview 7 English: 13 - 13 (0) |
| Current characteristics riparian buffers > Current characteristics scientists > Prohibition cultivation near watercourse (+) | the main objective is to keep the intensive agricultural activity away from the watercourse and to make a vegetative barrier so that everything that runs off the surface passes through that vegetation barrier.  Interview 3 English: 15 - 15 (0)  Similarly, there are regulations for this regarding the use of agrochemicals.  Interview 3 English: 17 - 17 (0) |
| Current characteristics riparian buffers > Current characteristics scientists > Vegetation density | In regards to the main characteristics of buffers, that would be plant density.  Interview 3 English: 15 - 15 (0) |
| Current characteristics riparian buffers > Current characteristics scientists > Vegetation recovery | the vegetation area to recover, right? For that recovery to be active, for it to be significant.  Interview 3 English: 11 - 11 (0)  In some buffer zones, the vegetation is actively reclaimed, in others, it is left to recover.  Interview 3 English: 15 - 15 (0) |
|  | And then there's the second intervention, which is to actively restore or rewild some of the areas.  Interview 22 English: 9 - 9 (0) |
| Current characteristics riparian buffers > Current characteristics scientists > Vegetation structure | there are structural aspects, community composition, soil interactions with soil.  Interview 22 English: 13 - 13 (0) |
| Current characteristics riparian buffers > Current characteristics scientists > Woody vegetation | I was on the committee of a study that looked at how different vegetation covers affect these different roles. So, one kind of mistake is that we might think that it's mostly forests that are playing a key role in all of these ecosystem services, but so far what the research is finding is that you can also have grassland cover, native grasslands, and obviously they don't behave exactly the same way as forests do with tree cover, but they do play a critical role in capturing sediments.  Interview 8 English: 9 - 9 (0) |
|  | the native woodland is not as native as they say, but to make it so that it is, that it is what it was before, The problem is that it becomes a species that grows very slowly. So, when you want to cover soil, you have that problem.  Interview 23 English: 9 - 9 (0) |
| Current characteristics riparian buffers > Current characteristics Vida Silvestre > Adaptive ecosystem management | they need to resemble as much as possible the native ecosystems that were there. We do understand that once you introduce changes in a landscape, you are not able to return to the regional conditions, but what you do is leave natural ecological processes to restore natural ecological systems, enabling the recovery of the composition and structure of those ecosystems. But it doesn't mean reintroducing an ecosystem with the same conditions because those conditions have changed both locally but also around. So, you are managing an adaptive system that it currently is, and what we are trying to do is to restore some of its original conditions, but it won't be the same because the system is not the same. But you do want to, in general, contribute to maintaining the same processes and services when you see it from a human perspective that were there before you remove that ecosystem.  Interview 20 English: 17 - 17 (0)  And so, as we are talking about an ecosystem, we are not just talking about the floristic characteristics, but the whole range of interactions among species from different fields, I mean from the different ranges of species you will find there; plants and animals and fungi and other components of diversity.  Interview 20 English: 17 - 17 (0) |
| Current characteristics riparian buffers > Current characteristics Vida Silvestre > Native vegetation | they need to resemble as much as possible the native ecosystems that were there. We do understand that once you introduce changes in a landscape, you are not able to return to the regional conditions, but what you do is leave natural ecological processes to restore natural ecological systems, enabling the recovery of the composition and structure of those ecosystems. But it doesn't mean reintroducing an ecosystem with the same conditions because those conditions have changed both locally but also around. So, you are managing an adaptive system that it currently is, and what we are trying to do is to restore some of its original conditions, but it won't be the same because the system is not the same. But you do want to, in general, contribute to maintaining the same processes and services when you see it from a human perspective that were there before you remove that ecosystem.  Interview 20 English: 17 - 17 (0) |
| Preferred characteristics riparian buffers > Preferred characteristics CAF > Controlled phosphorus use | First is that you need to take control of the amount of phosphorus you have on your farm as a farmer you have in your farm.  Interview 24 English: 9 - 9 (0) |
| Preferred characteristics riparian buffers > Preferred characteristics CAF > Protection water storages | Third, you need to keep the areas where you collect the water protected because in one place the water ends, and you need to keep this area protected, as well as the areas near the rivers.  Interview 24 English: 9 - 9 (0) |
| Preferred characteristics riparian buffers > Preferred characteristics CAF > Slope-based dimensions | It depends on the slope. If you have a very low slope, you probably need 50 meters or less, but if the slope is very steep, you need to keep probably 200 meters. It depends on the condition. It's not only one measure, maybe more than one.  Interview 24 English: 9 - 9 (0) |
| Preferred characteristics riparian buffers > Preferred characteristics CEUTA > Integrated buffer zones | Not only to make the buffer zone work, but to start integrating the producer into the design, and into the management. We think that this is the first step to an agroecological transition, which obviously can take many, many years.  Interview 2 English: 3 - 3 (0) |
| Preferred characteristics riparian buffers > Preferred characteristics CEUTA > Access cattle to buffer zone | For example, by using a combine harvester when going into harvesting, at some point, or letting cattle enter the buffer to do intermittent grazing.  Interview 2 English: 9 - 9 (0)  And that is where the possibility of intermittent grazing comes up, and how it would be regulated. One possibility would be that the producer would have to notify the municipality each time they make grazing, or whatever the local authority is.  Interview 2 English: 11 - 11 (0) |
| Preferred characteristics riparian buffers > Preferred characteristics CEUTA > Boundary management | That's why we started to play a little bit with it. Because we could propose setting the boundaries of the buffer zone itself. That is to say, we could determine where the boundaries are established of where you can till the soil.  Interview 2 English: 11 - 11 (0) |
| Preferred characteristics riparian buffers > Preferred characteristics CEUTA > Fodder production | they could have the purpose produce food as a fodder bank.  Interview 2 English: 9 - 9 (0) |
| Preferred characteristics riparian buffers > Preferred characteristics CNFR > Alternative fertiliser use | try to promote other types of fertilisation agents or to change some crops that are very demanding in terms of agrochemical use for others that are not so demanding, but that the producer family can manage and that production is really viable.  Interview 5 English: 16 - 16 (0)  Others are trying to avoid permanent fertilisation with chemicals through the incorporation of green manures or legumes that fix Nitrogen in the soil.  Interview 5 English: 16 - 16 (0) |
| Preferred characteristics riparian buffers > Preferred characteristics CNFR > Elimination exotic species | elimination of exotics  Interview 5 English: 24 - 24 (0) |
| Preferred characteristics riparian buffers > Preferred characteristics CNFR > Woody vegetation | The bush allows the cattle to have access to a place where they can suddenly put a watering trough so that, by drawing a little bit of water from the river or from some springs, the cattle can drink in the shade and it's more comfortable.  Interview 5 English: 24 - 24 (0) |
| Preferred characteristics riparian buffers > Preferred characteristics CNFR > Mulching | Other growers are avoiding the use of herbicides by using mulch.  Interview 5 English: 16 - 16 (0) |
| Preferred characteristics riparian buffers > Preferred characteristics CNFR > Nature restoration plan | a strict conservation or restoration plan can be implemented in those areas so that they can comply with those ecosystem services  Interview 5 English: 20 - 20 (0)  If we also implement the practices, we were talking about today in the areas close to the buffer zones at the level of agriculture or livestock farming, we will be helping to minimise the problem.  Interview 5 English: 20 - 20 (0)  protected areas within each property.  Interview 5 English: 20 - 20 (0) |
| Preferred characteristics riparian buffers > Preferred characteristics CNFR > Restrictive access cattle to watercourse | allow some cattle passages, so that they can get to the stream and drink water so that they have shade in the summer.  Interview 5 English: 24 - 24 (0) |
| Preferred characteristics riparian buffers > Preferred characteristics DINAGUA > Dimensions | We wanted to broaden the spectrum up to the courses, expanding in some places like Arroyo de la Virgen by 25 metres on the side.  Interview 6 English: 12 - 12 (0)  it is very important that the buffer zones cover as many areas as possible in order to improve the overall water quality and quantity of the Rio Santa Lucia. The reservoirs directly associated with the intake are protected, but protection is only provided in the easternmost areas closest to the water intake.  Interview 6 English: 27 - 27 (0) |
| Preferred characteristics riparian buffers > Preferred characteristics DINAGUA > Extensive management | it should be extensive  Interview 6 English: 22 - 22 (0) |
| Preferred characteristics riparian buffers > Preferred characteristics DINAGUA > Layered vegetation | cultivation of certain plants that not only mitigate but also reinforce the function of the buffer zone.  Interview 6 English: 27 - 27 (0)  implementing layers with vegetation would enable a greater advantage to the owner of the area and improve its use.  Interview 6 English: 27 - 27 (0) |
| Preferred characteristics riparian buffers > Preferred characteristics DINAGUA > Grassy vegetation | A meadow is not the same as a eucalyptus forest in relation to water absorption, which are affecting the water harvesting zone.  Interview 6 English: 24 - 24 (0) |
| Preferred characteristics riparian buffers > Preferred characteristics DINAGUA > Prevention weeds | If certain weeds were prevented, it could strengthen the retention function of the buffer zone.  Interview 6 English: 27 - 27 (0) |
| Preferred characteristics riparian buffers > Preferred characteristics DINAGUA > Protection buffer zones | Unfortunately, the farthest areas are not protected, but if they were protected to the full extent, there would be a more profound improvement.  Interview 6 English: 27 - 27 (0) |
| Preferred characteristics riparian buffers > Preferred characteristics DINAGUA > Regulated control | and it should be regulated.  Interview 6 English: 22 - 22 (0) |
| Preferred characteristics riparian buffers > Preferred characteristics DINAMA > Biomass harvesting | let's say we harvest the biomass to reduce the nutrients.  Interview 10 English: 15 - 15 (0)  biomass can perhaps be harvested  Interview 10 English: 26 - 26 (0) |
|  | Benefits of these changes or additions to buffer zones include moving animals away from the water source and vegetation to harvest nutrients  Interview 12 English: 21 - 21 (0)  In the case of vegetative felling, harvesting the grain or plant and taking the nutrients for food or sale outside the farm or watershed would be ideal. It would also decrease the nutrient preservation that the buffer has (e.g., nutrient gathering), which would be a positive outcome of that kind of management.  Interview 12 English: 21 - 21 (0)  So, you need another regulation or ministerial resolution that enables grazing to be downgraded, or enables the sowing of maize and legumes for the extraction of phosphorus.  Interview 12 English: 25 - 25 (0) |
| Preferred characteristics riparian buffers > Preferred characteristics DINAMA > Lower-order stream buffer zones | one of the proposals I came across was to extend the plan to lower-order streams, as stated in the document.  Interview 10 English: 21 - 21 (0)  The main difference is that we are extending the buffer strip to smaller courses of smaller size. It's something like a 5 to 1 ratio in length. In other words, we want to propose adding about 5 times more length of buffer than we did in the first phase.  Interview 10 English: 26 - 26 (0) |
| Preferred characteristics riparian buffers > Preferred characteristics DINAMA > Low-stocking grazing | we understand that grazing would be allowed at low stocking rates. Low stocking means minimal grazing, and we don't worry too much about it if the activity is not intensive.  Interview 10 English: 17 - 17 (0) |
|  | Benefits of these changes or additions to buffer zones include moving animals away from the water source and vegetation to harvest nutrients  Interview 12 English: 21 - 21 (0)  For instance, asking the producer to graze animals at a low stocking rate means fencing or delimiting that area.  Interview 12 English: 23 - 23 (0) |
| Preferred characteristics riparian buffers > Preferred characteristics DINAMA > Restoration native forests | Other measures, such as restoration (of native forests, red.) in some parts, are also being considered.  Interview 12 English: 13 - 13 (0) |
| Preferred characteristics riparian buffers > Preferred characteristics DINAMA > Variable width | The main characteristic is the variable width.  Interview 12 English: 15 - 15 (0) |
| Preferred characteristics riparian buffers > Preferred characteristics DINOT > Locality-based buffer zones | But most importantly, the so-called preferred zero land use area was identified, which is the area where the ecosystem of the river should be protected above all. So, the 30-metre and 20-metre buffer zone was changed into another area, which was, let's say, a bit wider than the smaller strip of one metre, when the different conditions of soil, geomorphology and historical use were clarified. And that is a zero-use area where the conservation area was established.  Interview 21 English: 5 - 5 (0)  It's not a continuous buffer of 50, 20, 30 feet anymore, right? It adjusts to the topography and the flood pressure of the river. It's obviously the definition of the river, not the definition of the river.  Interviewer 2: You are talking about a variable buffer zone?  Interviewee: Yes, of course, including some of the streams, the places where it was deemed necessary to protect them. Because that's where they provide the amount of fresh water that then also protect places, not only the plains, but also higher places where the river water is supplied in a way, also gives some sense of protection.  Interview 21 English: 15 - 17 (0)  Here I said compatibility, adaptability with incompatibility conditions.  Interview 21 English: 21 - 21 (0)  I think that in line with what I was saying, to me the buffer zone is an area of absolute restriction. For me, it has to be deconstructed a little bit to create an understanding. So, let's put them there, and we understand that the buffer zone is that space of the territory where the river, as a unit, needs to be in a situation like that. I think that for me it is a mistake to think that after that the buffer zone is an immediate line; thinking of the buffer zone as that reduced space around the river, after that there is a border, and after that there is this homogeneous productive territory. To me, in the middle there is a necessary protection area, so surely the buffer zone also needs its more intensive, more traditional land uses, both urban and rural.  Interview 21 English: 32 - 32 (0)  Then, it seems to me that the buffer zone is not something homogeneous, but that it is something that has to be studied according to the geomorphology in which it is located; the type of soil that there is there to see the kind of historical productive activity and the productive vocation of the place.  Interview 21 English: 32 - 32 (0) |
| Preferred characteristics riparian buffers > Preferred characteristics DINOT > No intensive agriculture | And in my opinion, there has been an interesting development, but not at the speed required for the problem: namely, the understanding of the river not as a watercourse, but as an area integrated by different productive activities of land without productive use or destination, without intensive industrial activity.  Interview 21 English: 5 - 5 (0)  But most importantly, the so-called preferred zero land use area was identified, which is the area where the ecosystem of the river should be protected above all. So, the 30-metre and 20-metre buffer zone was changed into another area, which was, let's say, a bit wider than the smaller strip of one metre, when the different conditions of soil, geomorphology and historical use were clarified. And that is a zero-use area where the conservation area was established.  Interview 21 English: 5 - 5 (0) |
| Preferred characteristics riparian buffers > Preferred characteristics INIA > Native vegetation | While this approach may restore some of the ecosystem services of the buffer zone, it does not constitute restoration of the original vegetation, as only a single species is planted.  Interview 15 English: 19 - 19 (0)  But perhaps in some of them, there should have been a forest and it was either cut down or because of repeated grazing or trampling, it has disappeared or is of very low quality.  Interview 15 English: 23 - 23 (0)  I prefer it to be natural, what corresponds to that area. If it is forest, it should be forest and not something else. And if naturally there were, for example, high grasslands or grasslands, that is the vegetation that is there. But I don't like the design, saying that's what I'm going to plant here, and that's what has to be there, but that it is as similar as possible to the original ecosystem, that is the vegetation that corresponds to each site.  Interview 15 English: 25 - 25 (0)  Another active intervention could be introducing missing species and giving them the possibility to establish themselves. If there are controls on exotics, these species can occupy the spaces and prevent exotics from taking over again.  Interview 15 English: 37 - 37 (0) |
| Preferred characteristics riparian buffers > Preferred characteristics INIA > No-tillage management | which may include measures such as no soil tillage, no application of agrochemical fertilizers, and maintaining natural vegetation.  Interview 15 English: 31 - 31 (0) |
| Preferred characteristics riparian buffers > Preferred characteristics INIA > Prohibition use agrochemicals | which may include measures such as no soil tillage, no application of agrochemical fertilizers, and maintaining natural vegetation.  Interview 15 English: 31 - 31 (0) |
| Preferred characteristics riparian buffers > Preferred characteristics INIA > Vegetation structure | That requires a diversity of organisms, and that diversity of organisms, whether fauna or flora, depends on the diversity of structure that is present, many species, and different strata of vegetation.  Interview 15 English: 23 - 23 (0) |
| Preferred characteristics riparian buffers > Preferred characteristics INIA > Vegetative diversity | Is it all about diversity?  Interviewee: Yes, that's precisely why I emphasized the issue of diversity and structure. We can plant a buffer zone with Paspalum notatum, which creates a physical barrier and consumes nutrients in summer. However, we need diversity in plant species so that nutrients are consumed all year round, not just in summer. This also promotes growth and provides habitat for wildlife, insects, pollinators, and other natural species.  Interview 15 English: 28 - 29 (0)  As an agronomist, I believe it's important because maintaining diversity helps prevent colonization by invasive species and promotes the evolution of ecosystems. It may also provide other services that we may not have considered.  Interview 15 English: 31 - 31 (0)  we should focus on litter-free exotic control measures. This is one of the main problems within the catchment and is the most significant active measure as it doesn't happen alone.  Interview 15 English: 37 - 37 (0) |
| Preferred characteristics riparian buffers > Preferred characteristics MGAP > Drinking water facilities for cattle | But there is one characteristic, if you offer the animals the needed quality and quantity of water at a drinking location in plots, the animal is not going to choose to go to the water course to drink water, but it will go to that drinking facility.  Interview 17 English: 33 - 33 (0) |
| Preferred characteristics riparian buffers > Preferred characteristics MGAP > Intermittent grazing | and maybe with good management, producers can use it for cattle alimentation, for example. Because if they're in the buffer zone, it's grass that doesn't produce. For example, in native grass, maybe there is a risk of accumulation, so a lot of nitrogen, for example, or phosphorus is there, and sooner or later, maybe that phosphorus ends at the river. So, if you plant there, for example, a pasture with high production, that pasture can consume those nutrients, and maybe the farmers can use it to feed their cattle. Maybe in a way so that the cattle don't excrete at the buffer zone, maybe getting into the buffer zone like four or five hours a day, not every day and all the day, that they can use that buffer zone. Maybe they can do it that way.  Interview 19 English: 15 - 15 (0) |
| Preferred characteristics riparian buffers > Preferred characteristics MGAP > Locality-based buffer zones | It all depends on where they are, to define what is added or not. But my understanding is that if progress can be made with management plans, you can define in more detail what would or would not be added to implement in buffer zones. It is not black and white. I think it's not on a site-by-site basis, but it is for the characteristics of the watercourses that you want to protect. That is fine.  Interview 17 English: 33 - 33 (0)  So, when it comes to thinking about the buffer zone or the integrated management of the property with respect to that buffer zone, there are many things that can be done without being too extreme, like fencing it off, like prohibiting the use.  Interview 17 English: 33 - 33 (0) |
| Preferred characteristics riparian buffers > Preferred characteristics MGAP > No access cattle to watercourse | To ensure water quality, animals must not be allowed access when they drink water. That is to say, a lot of issues would be avoided, but it's not a cheap measure.  Interview 17 English: 38 - 38 (0) |
|  | What we need is only some meters with no cropping or with no cattle  Interview 19 English: 33 - 33 (0) |
| Preferred characteristics riparian buffers > Preferred characteristics MGAP > No fencing | That is to say, fencing off the water zones is something that's kind of insane, because there would be many kilometres of wire fencing. I don't know if it's done in the dimension of what I'm saying, but it would be insane.  Interview 17 English: 33 - 33 (0) |
| Preferred characteristics riparian buffers > Preferred characteristics MGAP > Productive vegetation | And then you can plant or sow, I don't know how to say it, some productive species, and maybe with good management, producers can use it for cattle alimentation, for example.  Interview 19 English: 15 - 15 (0) |
| Preferred characteristics riparian buffers > Preferred characteristics MGAP > Smaller sub-commissions | : See, not new commissions, you know, smaller commissions. Not a new one. For example, the 60 people, I don't know how, but sometimes when, when I used to be there, there were maybe 70 people there. It's very hard to discuss with too many people. So sub-commissions, you know, maybe 20 people working buffer zone, other 20 in, so can say for example, other stuff, things, and then once, for example, in six months or, or once in a year, all the sub-commissions get together in the big commission and then maybe present the results of their work and the, yeah, not a new one because we have a lot already of things.  Interview 19 English: 35 - 35 (0) |
| Preferred characteristics riparian buffers > Preferred characteristics producers > Agroecological production | The latent idea is that producers can differentiate their production. In other words, it could not be a designation of standard agriculture, but it could favour organic or agroecological production.  Interview 11 English: 11 - 11 (0)  We proposed beekeeping with native forests, livestock farming in reconversion as far as possible, and organic and seed production.  Interview 11 English: 11 - 11 (0) |
| Preferred characteristics riparian buffers > Preferred characteristics producers > Access cattle to buffer zone | It seems to me that direct access to water for animals through watercourses would have to be prohibited, but grazing would have to be allowed. Otherwise, the strip of land would not be managed, which is a problem. Who manages that strip of land? Nobody. I think the argument that there may be grazing in that strip is insignificant compared to the contributions of agriculture or a production area. Obviously, there is no feedlot, concentration of livestock, chicken farm, agricultural cultivation, or fertilization, but grazing should be allowed on a low carpet with some trees that does not interfere with the runoff.  Interview 11 English: 15 - 15 (0) |
|  | The animal does not enter the riparian forest, so the animal does not take water from the river. Then, I have water distribution in the plot through which water arrives. Now I am waiting for them to come and fix a pipe that broke, so I have all the cattle concentrated here. But there are degrees of buffer areas. There are buffer and restricted areas where the cattle do not enter, where nothing is done in the riparian forest, and then you have the formation that I call the “park type”, where normally there is no water. I have three levels: the riparian forest, then I have the level of the park-like forest, and then I have what I call a “bajo” (meaning: low, red.), which is the twelve three hectares where there are practically no trees, which is a field that I manage. In the natural area, I add another one, which is a kind of summer pasture. I don't know if you understood the concept; they are three different buffer areas or three different degrees of buffer intensity.  Interview 13 English: 19 - 19 (0) |
|  | Permission to enter the buffer zone. So that you can move forward a little bit. I mean, a little bit and not be fined, you know. I mean, I understand that you have to leave the water, but a little bit to fields that are very close to the street, right? To combat the invasive species.  Interview 14 English: 70 - 70 (0) |
| Preferred characteristics riparian buffers > Preferred characteristics producers > Allowing combatting exotic species | Removing the blackberry plant could help since it takes up space and prevents grass growth. However, removing it is difficult since it reproduces quickly. Herbicides might be necessary to prevent it from coming back stronger. It seems to me that by removing this invasive plant, in some way, as it takes hold more on its own, to see more grass, the ground will be more covered, right.  Interview 14 English: 66 - 66 (0) |
| Preferred characteristics riparian buffers > Preferred characteristics producers > Fencing | We could make a pasture with fences or something that doesn't require much maintenance.  Interview 14 English: 45 - 45 (0) |
| Preferred characteristics riparian buffers > Preferred characteristics producers > Wood production | But they (the government) won't let us sell the timber generated at that buffer. There is none. In other words, the bush is registered. Supposedly, the first step to selling the timber with a permit is to have the forest registered, but they don't give us the permit to sell the timber. That is a big limitation to being able to have better things.  Interview 4 English: 9 - 9 (0)  active felling is necessary for nutrient uptake.  Interview 4 English: 54 - 54 (0) |
|  | and you can have firewood,  Interview 13 English: 15 - 15 (0) |
| Preferred characteristics riparian buffers > Preferred characteristics producers > Grassy vegetation | the ecosystem of the trees with the pasture below for the cattle is wonderful.  Interview 4 English: 27 - 27 (0)  Pastures should be taken into account, as vegetation, shrubs, grass, and trees need to be present.  Interview 4 English: 54 - 54 (0) |
|  | all you do is collect the growth of forage to encourage regrowth because the forage plants have a growth rate that is called a certain rate.  Interview 13 English: 17 - 17 (0) |
|  | I have no problem, but it's limiting because it says maybe I could put them there. If they (the buffer zones) stayed there, I could take advantage of them, even if they were stationary, without movement, or anything. It limits the field, that's all. You could say I could take advantage of them with pasture, excuse me, with the cows being there. I mean, taking advantage of them in that sense, without limitations  Interview 14 English: 31 - 31 (0)  And it would be advantageous to be able to let it become pasture, something more usable.  Interview 14 English: 62 - 62 (0) |
| Preferred characteristics riparian buffers > Preferred characteristics producers > Intermittent grazing | Yes, I could make a solution, with controlled, intermittent grazing, a certain number of animals, set thresholds, number of animals per path, certain times yes, other times no, perhaps in flood zones. Not in winter periods, in summer yes.  Interview 11 English: 17 - 17 (0) |
| Preferred characteristics riparian buffers > Preferred characteristics producers > Locality-based buffer zones | The animal does not enter the riparian forest, so the animal does not take water from the river. Then, I have water distribution in the plot through which water arrives. Now I am waiting for them to come and fix a pipe that broke, so I have all the cattle concentrated here. But there are degrees of buffer areas. There are buffer and restricted areas where the cattle do not enter, where nothing is done in the riparian forest, and then you have the formation that I call the “park type”, where normally there is no water. I have three levels: the riparian forest, then I have the level of the park-like forest, and then I have what I call a “bajo” (meaning: low, red.), which is the twelve three hectares where there are practically no trees, which is a field that I manage. In the natural area, I add another one, which is a kind of summer pasture. I don't know if you understood the concept; they are three different buffer areas or three different degrees of buffer intensity.  Interview 13 English: 19 - 19 (0) |
| Preferred characteristics riparian buffers > Preferred characteristics producers > Native vegetation | When climate problems arise, native vegetation can be more resilient than productive exotic species.  Interview 13 English: 43 - 43 (0) |
| Preferred characteristics riparian buffers > Preferred characteristics producers > No-management zone | it should be left as it is and not touched.  Interview 16 English: 45 - 45 (0) |
| Preferred characteristics riparian buffers > Preferred characteristics producers > Permanent groundcover | Ground cover needs to be permanent  Interview 4 English: 54 - 54 (0) |
| Preferred characteristics riparian buffers > Preferred characteristics producers > Pruning low vegetation | I believe in pruning the vegetation to allow cattle to graze below and to promote better plant growth. It's all about managing the natural pasture, and it's something that can be seen now with the paintbrush.  Interview 13 English: 97 - 97 (0) |
| Preferred characteristics riparian buffers > Preferred characteristics producers > Tillage | You can do tillage. And try to keep it as clean as possible. There's not much else.  Interview 14 English: 56 - 56 (0) |
| Preferred characteristics riparian buffers > Preferred characteristics producers > Use leguminous plants | I am an advocate for improving buffer zones by adding leguminous plants, controlled fertilization in reasonable doses, and preserving gallery (native, red.) forests.  Interview 13 English: 9 - 9 (0) |
| Preferred characteristics riparian buffers > Preferred characteristics producers > Woody vegetation | Yes, the buffer zone would have to be forested.  Interview 16 English: 70 - 70 (0) |
| Preferred characteristics riparian buffers > Preferred characteristics scientists > Control invasive species | But from a conservation point of view, it is important that there is control of invasive species to sustain biodiversity and conserve the biodiversity of the system.  Interview 3 English: 44 - 44 (0) |
|  | With grazing, you have them more under control, but it could be a problem. But with the Fresno (Franxinus lancelota), it is a problem and surely for other is a problem, and surely for other species it could also happen for the Ligustro (Ligustrum lucidum), in many places, and for the Gleditsia (Gleditsia triacanthos) and for other herbaceous plants. Another function that I was going to mention but forgot is ecosystem services or ecosystem services.  Interview 9 English: 15 - 15 (0) |
| Preferred characteristics riparian buffers > Preferred characteristics scientists > Dimensions | But it would need to be a sufficient area to retain a significant percentage of the nutrients produced by agriculture in the basin, and also suspended particles.  Interview 3 English: 14 - 14 (0)  Something ideal would be that that buffer zone would cover all the margins of the watercourses that enter the system.  Interview 3 English: 17 - 17 (0)  it needs to be defined topographically as well, which also includes the flood areas. This way, the buffer zone should not be within the flooding area, but should be in the dry zone, which receives the surface runoff from the land (e.g., on the transition between the dry zone and the floodplain, red.).  Interview 3 English: 17 - 17 (0) |
|  | the geomorphology of the area  Interview 18 English: 17 - 17 (0) |
| Preferred characteristics riparian buffers > Preferred characteristics scientists > Exclusion cattle to watercourse | So, the cattle should not be allowed to go there to drink this water.  Interview 7 English: 17 - 17 (0) |
|  | because then you have the whole issue of livestock getting into the water courses, and that is not easy to solve because basically, when you don't want livestock getting into the river, it's very difficult.  Interview 22 English: 11 - 11 (0) |
| Preferred characteristics riparian buffers > Preferred characteristics scientists > Exclusion cattle to watercourse > Fencing | They should put a fence there to avoid this.  Interview 7 English: 17 - 17 (0) |
| Preferred characteristics riparian buffers > Preferred characteristics scientists > Geomorphology-based designs | a buffer zone has to be adjusted to the local conditions. It has to be evaluated according to the type of basin, the type of slopes, the type of runoff, and the topography of the terrain.  Interview 3 English: 14 - 14 (0) |
|  | So, the first criterion would be to restrict activities to the extent that certain activities could be carried out according to geomorphological criteria  Interview 18 English: 17 - 17 (0)  And then also with the design of the land adjacent to the buffer zones. If we cannot yet make progress on a good design of the buffer zone, we should think about a good design of the adjacent zones, but it is also unreasonable that there should be processes of recovery of buffer zones with intensive use that start on the other side of the buffer zone. So, initially, progress should be made in this recovery, but not ignoring the fact that the areas adjacent to the buffer zones will have an impact on this recovery process, either as a possible complement or as a possible reduction of a source, or both?  Interview 18 English: 22 - 22 (0)  For example, well, if we imagine a classic planning of the basin, well, we have a whole part in which, well, we are going to find water, with native woodland and then other parts that we are going to find with pastures that are going to be flooded during part of the year. If the criterion for defining the buffer zone includes this flooding area, at least for part of the year, this area could act as a protection zone adjacent to the wetland that is flooded on a more permanent basis or where there is tree vegetation. So, well, that reinforces my conviction that the beginning is the broad delimitation of the riparian zone from the floodplain.  Interview 18 English: 24 - 24 (0) |
| Preferred characteristics riparian buffers > Preferred characteristics scientists > Grassy vegetation | . I think the best thing to do is to have a permanent pasture and to harvest it into bales and take it out.  Interview 23 English: 17 - 17 (0)  It should be a pasture that is always there, a fescue (grass type, red). I tell you, there is the name of the plant, a fescue that can be maintained.  Interview 23 English: 21 - 21 (0) |
| Preferred characteristics riparian buffers > Preferred characteristics scientists > Biomass harvesting | the type of cultivation that is done on another strip above the buffer zone, which in many cases the in the U.S. is unfertilized areas, which are harvested to remove nutrients from that site.  Interview 3 English: 15 - 15 (0) |
|  | I believe that buffer zones do not have to be complete exclusion zones. In fact, a master's thesis conducted by a student with us in Paso Severino showed that buffer zones can also be potential sites for nutrient export, particularly for nitrogen in the form of ammonium and nitrate. This is because the biomass of herbaceous plants in the buffer area, which accumulates nutrients such as nitrogen, decomposes and releases nutrients almost as high as what is brought to agriculture, especially nitrogen. Well, I don't remember whether it was ammonium or nitrates. So, for me the buffer zones have to be managed, and especially in Uruguay. And then I also think that buffer zones have to be have to have management adapted to the environmental realities of the ecosystems and the productive uses of each locality.  Interview 9 English: 13 - 13 (0) |
|  | . I think the best thing to do is to have a permanent pasture and to harvest it into bales and take it out.  Interview 23 English: 17 - 17 (0)  Interviewer 2: And then, why is it important to harvest?  Interviewee: Because you are taking the phosphorus out. And by taking that out of there and distributing it in the upstream field, you are lowering the concentration in that place. Because if you fertilize it and harvest it, in the end, even if you don't get much of it annually, it will be so important that it will be even more difficult to saturate it.  Interview 23 English: 20 - 21 (0) |
| Preferred characteristics riparian buffers > Preferred characteristics scientists > Intermittent grazing | So, in Uruguay, where livestock production is the basis of everything, I believe that buffer zones should be managed with livestock at certain times of the year. That is to say that you put a high load of cattle, I don't know, once or twice a year to reduce the height of the vegetation, to remove nutrients and, well, some dung is going to be left there, but they're going to remove some of the biomass.  Interview 9 English: 13 - 13 (0) |
| Preferred characteristics riparian buffers > Preferred characteristics scientists > Intermittent vegetative strips | To me that would be very healthy if you had buffers within the crops or on their vegetation boundary beyond the fact that streams should maintain a buffer zone around the stream. This is for many reasons, first of all because the buffer zone is often in the elusiveness of the stream. It is dependent on the intensity of agricultural use and because of that it is also going to be the last filter that this body of water is going to have from what end up in the stream, so for me, the design should incorporate strips of vegetation inside the crops and. Buffer zones are most important at the headwaters and in small streams, as large streams have a greater surface area, agricultural use, and nutrient arrival at the watercourses.  Interview 9 English: 13 - 13 (0) |
| Preferred characteristics riparian buffers > Preferred characteristics scientists > Liming | Liming is adding a calcareous material to modify the reaction of the soil. In other words, everything to do with hydrogen, oxygen, and lime has to do with neutralizing the synaeresis when there is any. Well, what happens? With this system, the soil above the ground has become much more assertive. So, we are proposing to invert it.  Interview 23 English: 27 - 27 (0) |
| Preferred characteristics riparian buffers > Preferred characteristics scientists > Native vegetation | it would be important to get the natural vegetation that grows along the rivers, that has native vegetation, and there are also animals that live there.  Interview 7 English: 17 - 17 (0)  he buffers zone near the river should be the natural vegetation that always grew there, because these plants are adapted to the ecology of the region  Interview 7 English: 21 - 21 (0)  Not change the vegetation, just replant these species there, and let them recover these areas, have the same vegetation that was there when the Indians were there.  Interview 7 English: 21 - 21 (0) |
|  | I think we're just having one, a mix of different vegetation, so we know that grasses can play an important role. I'd have to look back at the data from different theses that have been done in the past couple years, but I think that it seems like a mix of forest cover and grassland cover and native grassland cover, would be important.  Interview 8 English: 19 - 19 (0) |
|  | And secondly, issues that have to do with vegetation, the recovery of native vegetation. Basically, well, in some areas it is wetland, in other areas it is grassland and in other areas it is riparian forest.  Interview 18 English: 17 - 17 (0)  to restore the natural vegetation according to the type of riparian zones  Interview 18 English: 17 - 17 (0) |
| Preferred characteristics riparian buffers > Preferred characteristics scientists > Nutrient depletion zone | before the buffer zone, we should put areas where the farmers can plant crops but not let animals there either, only for these areas that are also contaminated, going from the river up. In this area, only silage and this kind of stuff should be produced because they will remove the buffer of nutrients with the dry matter of the biomass that they have. In this way, there will be an area low in peak P near the water.  Interview 7 English: 17 - 17 (0)  use another practice near the border of these buffer zones to reduce the impact of agriculture and the amount of pee and other nutrients that reach the buffer zone  Interview 7 English: 21 - 21 (0) |
|  | And then also with the design of the land adjacent to the buffer zones. If we cannot yet make progress on a good design of the buffer zone, we should think about a good design of the adjacent zones, but it is also unreasonable that there should be processes of recovery of buffer zones with intensive use that start on the other side of the buffer zone. So, initially, progress should be made in this recovery, but not ignoring the fact that the areas adjacent to the buffer zones will have an impact on this recovery process, either as a possible complement or as a possible reduction of a source, or both?  Interview 18 English: 22 - 22 (0) |
| Preferred characteristics riparian buffers > Preferred characteristics scientists > Nutrient depletion zone > Application iron chloride | probably applying iron chloride in these areas could be added. Because it will retain the available peak and the leaching. The movement of phosphorus with water flowing down in the surface will be contained much less in the buffer. I think this will be a good management practice, especially in areas that have heavy contamination.  Interview 7 English: 17 - 17 (0) |
| Preferred characteristics riparian buffers > Preferred characteristics scientists > Tillage management | We then ploughed the soil and found that the losses disappeared, were zero, or less than those in the natural area. We also compared the differences between putting fertilizer on top or a few centimetres into the soil and found that the difference was huge too.  Interview 7 English: 13 - 13 (0) |
| Preferred characteristics riparian buffers > Preferred characteristics scientists > Vegetation transition | So, it is possible to think about management, beyond natural recovery, as had also been proposed, to have transitions of certain types of vegetation.  Interview 3 English: 15 - 15 (0) |
|  | to restore the natural vegetation according to the type of riparian zones  Interview 18 English: 17 - 17 (0) |
| Preferred characteristics riparian buffers > Preferred characteristics scientists > Woody vegetation | But also, an important function that I see is that once you plant trees, in a way you reclaim the land from agriculture.  Interview 22 English: 11 - 11 (0) |
| Preferred characteristics riparian buffers > Preferred characteristics Vida Silvestre > Integrated ecosystem management | what we think is that you need to manage the landscape in an integrated way,  Interview 20 English: 19 - 19 (0) |
| Preferred characteristics riparian buffers > Preferred characteristics Vida Silvestre > Native and original vegetation structure | Regarding the buffer zones, the key issue is to enable the recovery of the original structure preferably with the same or with a similar composition of species, but in some areas, you may need different strategies as you have productive activities producing an impact.  Interview 20 English: 19 - 19 (0) |
| Preferred characteristics riparian buffers > Preferred characteristics Vida Silvestre > Nature conservation | And for us, a protected area is basically another thing that's interesting because for us a protected area is a productive landscape that is managed in a way that enables economic development, social development but also ecological resilience and nature conservancy. So, all those objectives have to be put together and weighed and at the same time taking into consideration the specific individual needs of the landowners and the collective interests and needs of the country, or at least the people from the three departments that are involved with the management of the area.  Interview 20 English: 23 - 23 (0) |
| Preferred characteristics riparian buffers > Preferred characteristics Vida Silvestre > Productive land use | A protected area is an area that has a specific set of rules that has a goal in mind, the sustainable development of that region in a manner that articulates private interests and enables productive activities, but it also enables the protection of certain areas within that area, that overall ensure that certain sites are key to maintain ecological processes at the scale of the whole area.  Interview 20 English: 19 - 19 (0)  And for us, a protected area is basically another thing that's interesting because for us a protected area is a productive landscape that is managed in a way that enables economic development, social development but also ecological resilience and nature conservancy. So, all those objectives have to be put together and weighed and at the same time taking into consideration the specific individual needs of the landowners and the collective interests and needs of the country, or at least the people from the three departments that are involved with the management of the area.  Interview 20 English: 23 - 23 (0) |
| Preferred characteristics riparian buffers > Preferred characteristics Vida Silvestre > Sustainable development | A protected area is an area that has a specific set of rules that has a goal in mind, the sustainable development of that region in a manner that articulates private interests and enables productive activities, but it also enables the protection of certain areas within that area, that overall ensure that certain sites are key to maintain ecological processes at the scale of the whole area.  Interview 20 English: 19 - 19 (0) |
| Scores preferred ecosystem services buffer zones > CEUTA > Biodiversity | And then I could put a three to the enrichment of other ecosystem ecosystem services well.  Interview 2 English: 18 - 18 (0) |
| Scores preferred ecosystem services buffer zones > CEUTA > Integrated buffer management | Let's see, if we add up and if they fit in with my vision of the buffer zone that has to incorporate boundary management and a systemic vision of the property, I would also add three or four to the production function.  Interview 2 English: 18 - 18 (0) |
| Scores preferred ecosystem services buffer zones > CEUTA > Nutrient retention | nutrient retention would be a 4. It is obviously the most important.  Interview 2 English: 18 - 18 (0) |
| Scores preferred ecosystem services buffer zones > CEUTA > Agricultural productivity | four to the production function.  Interview 2 English: 18 - 18 (0) |
| Scores preferred ecosystem services buffer zones > CNFR > Erosion reduction | I would give avoiding soil erosion at least a 3.  Interview 5 English: 22 - 22 (0) |
| Scores preferred ecosystem services buffer zones > CNFR > Nutrient retention | Avoiding soil erosion and transporting fertiliser, and transporting nutrients are the same, let's say. Exactly, and I think we can give it a 3 there as well.  Interview 5 English: 22 - 22 (0) |
| Scores preferred ecosystem services buffer zones > CNFR > Agricultural productivity | For me, that's a 4. In other words, this area is useful. They are. Whoever needs firewood has it. Whoever needs shade has it. Anyone who needs access to water has it.  Interview 5 English: 24 - 24 (0) |
| Scores preferred ecosystem services buffer zones > CNFR > Restoration natural ecosystems | Furthermore, recreate the natural system, which I see more as something associated with the diversity pathway. For me, 4, that's the most important thing of all.  Interview 5 English: 22 - 22 (0) |
| Scores preferred ecosystem services buffer zones > DINAMA > Biodiversity | followed by erosion protection and biodiversity protection. However, we believe that protecting fauna and flora requires something different from the buffer zone we designed.  Interview 10 English: 19 - 19 (0) |
| Scores preferred ecosystem services buffer zones > DINAMA > Erosion protection | followed by erosion protection and biodiversity protection. However, we believe that protecting fauna and flora requires something different from the buffer zone we designed.  Interview 10 English: 19 - 19 (0) |
| Scores preferred ecosystem services buffer zones > DINAMA > Nutrient retention | As our design is made to filter nutrients, I rank nutrient retention as the most important  Interview 10 English: 19 - 19 (0) |
| Scores preferred ecosystem services buffer zones > Farmers > Agricultural productivity | Although economic benefits, such as generating fodder, are also important, for me, the process and fodder are valuable.  Interview 13 English: 45 - 45 (0) |
|  | Also, because of the characteristics of the terrain, the nutritional quality of what it generates is very low. It's not a high-quality pasture that you can say, "let's take advantage of this." No, because the contribution it can make is relatively low. You couldn't do cattle farming there if you don't have a part of the area also dedicated to life."  Interview 16 English: 53 - 53 (0) |
| Scores preferred ecosystem services buffer zones > Farmers > Nutrient retention | If I had to rank the importance of these benefits from recreation to nutrient retention, I believe that the protection of the river, watercourse, and soil is the most crucial one, as humans can spread everywhere. Although economic benefits, such as generating fodder, are also important, for me, the process and fodder are valuable.  Interview 13 English: 45 - 45 (0) |
|  | And the retention of nutrients, for example, the retention of pollutants?  Interviewee: Yes, also, without a doubt. But its less important than water, so 3. For me, the issue of water would be more important than anything else.  Interview 16 English: 50 - 51 (0) |
| Scores preferred ecosystem services buffer zones > Farmers > Water resource protection | If I had to rank the importance of these benefits from recreation to nutrient retention, I believe that the protection of the river, watercourse, and soil is the most crucial one, as humans can spread everywhere. Although economic benefits, such as generating fodder, are also important, for me, the process and fodder are valuable.  Interview 13 English: 45 - 45 (0) |
|  | If we consider water, 4 would be the best.  Interview 16 English: 49 - 49 (0) |
| Scores preferred ecosystem services buffer zones > INIA > Recreation | Perhaps landscape is another important characteristic. For many people, the soil cover is a 1 or a 2, but for me, it's a 4 - it depends on the individual (aesthetics, red.). This is important for many older people. I don’t know for younger people, but probably.  Interview 15 English: 33 - 33 (0)  Yes, it's a visual aspect. For the vegetation cover, it is particularly important. For many people it's important as they believe it is essential. I'm not sure about younger people, but probably yes.  Interview 15 English: 35 - 35 (0) |
| Scores preferred ecosystem services buffer zones > INIA > Biodiversity | For me, vegetation structure is 4 as it is the most important thing in the structure. Land cover is 3. Perhaps landscape is another important characteristic. For many people, the soil cover is a 1 or a 2, but for me, it's a 4 - it depends on the individual (aesthetics, red.).  Interview 15 English: 33 - 33 (0) |
| Scores preferred ecosystem services buffer zones > INIA > Restoration natural ecosystems | Land cover is 3  Interview 15 English: 33 - 33 (0) |
| Scores preferred ecosystem services buffer zones > INIA > Nutrient retention | vegetation structure is 4 as it is the most important thing in the structure.  Interview 15 English: 33 - 33 (0) |
| Scores preferred ecosystem services buffer zones > Scientists > Recreation | I mean, you could give nutrient retention a four, watercourse erosion protection a three, biodiversity a three, and aesthetics at the same level as these other two (a 3, red.).  Interview 9 English: 17 - 17 (0) |
| Scores preferred ecosystem services buffer zones > Scientists > Agricultural productivity | Interviewer: Okay. And if we take also into account the potential productivity of riparian zones to producers?  Interviewee: Oh, the potential productivity of riparian zones for producers, you mean for landowners or people?  Interviewer: Yes. And how important is that?  Interviewee: I mean, it's kind of a difficult question because I would say three because it's not really that important per se that these are highly productive zones. You know; however, the productivity of the trees will directly influence the other roles. It directly influences nutrient retention. It directly influences the development of the soil because if you have highly productive forest, then you're going to have more production of the detritus. So, it's not unimportant. If everything grows faster, your riparian zone will recover more quickly.  Interview 8 English: 30 - 33 (0) |
|  | Then, to isolate the buffers from the presence of animals (3), and lastly, the production aspect (2).  Interview 23 English: 39 - 39 (0) |
| Scores preferred ecosystem services buffer zones > Scientists > Biodiversity | but the other ecosystem services, in my heart, I think that they are very important, that the nature goes back there, and the water will not only be clean and transparent, but also there are animals there, different vegetation and fish there.  Interview 7 English: 27 - 27 (0) |
|  | Interviewer: And species diversity or biodiversity?  Interviewee: I mean for management; I would say I don't equivalent biodiversity. And just having native species. So, I would say two or three. Okay. I think biodiversity is important, but it's not really the principal goal of the riparian zone. And when you have water that you can't drink, I think that having about a burst forest is going to contribute to many of those roles, but it's not really the principal goal.  Interview 8 English: 28 - 29 (0) |
|  | I mean, you could give nutrient retention a four, watercourse erosion protection a three, biodiversity a three, and aesthetics at the same level as these other two (a 3, red.).  Interview 9 English: 17 - 17 (0) |
|  | Then, to isolate the buffers from the presence of animals (3), and lastly, the production aspect (2).  Interview 23 English: 39 - 39 (0) |
| Scores preferred ecosystem services buffer zones > Scientists > Ecosystem connectivity | Otherwise, the functioning of the ecosystem as a connected system, would be second, let's say. So that one a 3. That goes more to the conservation of the system or the ecosystem than to a practical end, right?  Interview 3 English: 21 - 21 (0) |
| Scores preferred ecosystem services buffer zones > Scientists > Erosion reduction | I mean, you could give nutrient retention a four, watercourse erosion protection a three, biodiversity a three, and aesthetics at the same level as these other two (a 3, red.).  Interview 9 English: 17 - 17 (0) |
| Scores preferred ecosystem services buffer zones > Scientists > Locality-based buffer zones | So, well, actually the two should have a value of 4. I can't imagine them as independent, but, well, if we had to start with something, maybe the beginning should be, at least, with a good delimitation of the buffer zone with geomorphological criteria.  Interview 18 English: 24 - 24 (0) |
| Scores preferred ecosystem services buffer zones > Scientists > Nutrient retention | Yes, I think on a practical level, the retention of nutrients and pesticides would be the most important for the water quality of the river. So those a 4.  Interview 3 English: 21 - 21 (0) |
|  | the most important is that they reduce the amount of P that goes into the water because this is the more immediate problem  Interview 7 English: 27 - 27 (0) |
|  | Interviewer: The ability to retain and capture nutrients.  Interviewee: Yeah. Four is the most important or the least important? The most, four.  Interview 8 English: 24 - 25 (0) |
|  | I mean, you could give nutrient retention a four, watercourse erosion protection a three, biodiversity a three, and aesthetics at the same level as these other two (a 3, red.).  Interview 9 English: 17 - 17 (0) |
|  | So, well, actually the two should have a value of 4. I can't imagine them as independent, but, well, if we had to start with something, maybe the beginning should be, at least, with a good delimitation of the buffer zone with geomorphological criteria.  Interview 18 English: 24 - 24 (0) |
|  | There is no doubt that the central objective is to stop the nutrient shift and erosion control.  Interview 23 English: 39 - 39 (0) |
| Scores preferred ecosystem services buffer zones > Scientists > Sediment retention | Yes, I think on a practical level, the retention of nutrients and pesticides would be the most important for the water quality of the river. So those a 4.  Interview 3 English: 21 - 21 (0) |
| Scores preferred ecosystem services buffer zones > Scientists > Erosion reduction | Interviewer: And construction of soils?  Interviewee: I would say four. Okay.  Interview 8 English: 26 - 27 (0) |
| Barriers preferred ecosystem services > CAF > Legislative complexity land-use | Agriculture is quite different because it is sometimes more dangerous. I suppose it's more dangerous than dairy; in dairy, you move the land less frequently than in agriculture. With agriculture, you probably need to make the work more intensive and more controlled, and you probably need to make some regulations about how you do things, like how you protect areas and how you take care of the amount of food you use  Interview 24 English: 15 - 15 (0)  they made a program related to the use of the soils in the area, but for example, you as an agronomic engineer and an advisor from a farmer need to fill out a form that says we are going to do this. Okay, complete this, but more than this, it is important that the farmer and the advisor understand how to do the bad things. It's not only to fill a paper; it's not easy. You can fill a paper; it's easy to do, but it's more complex when you go to the farm directly to the field, to the ground. They need to understand, and you need to train the people who are going to do the job on how to reduce this flow of water. For me, this part of the work is not done; that's my impression.  Interview 24 English: 19 - 19 (0) |
| Barriers preferred ecosystem services > CAF > Lack of information | We need to know first what happened with the rain and what happened with the amount of phosphorus coming outside of the crops. But they have no information, okay, including when I tell them to ask Ellenbank because they have a lot of information.  Interview 24 English: 7 - 7 (0)  I suppose I'm very important now, and if I try to tell the government the other things to do, maybe go study the impact you have with the water, with the rainwater, on the movement of the phosphorus, we have no information about that, we have very little information, and the only information I have about this issue is related to Iowa, where they made a study with an agronomic engineer who was working in the United States for a long time ago and had a study about this and on the station in Australia. I don't know how our country does this study; everybody talks about things but not with information. I need the information to be a scientist, and if I told you that's the only thing, I'm not a scientist, I'm a speaker.  Interview 24 English: 9 - 9 (0)  Okay, these kinds of things are probably undervalued by the farmers because nobody explained them to them.  Interview 24 English: 13 - 13 (0) |
| Barriers preferred ecosystem services > CAF > Need for local adaptation of measures | This problem, it's important for the government to say okay, we try to keep the soils safe and try to recover the rivers and the creeks that are contaminated with the soil, so that is the important thing. We need to persuade the farmers, okay, you have a very high receptivity from the farmers. But they need to understand that the problem we have in our soils in Uruguay is quite different from the problem, for example, in the soils of Argentina, Europe, or Australia. It's really very different. That's the idea,  Interview 24 English: 11 - 11 (0) |
| Barriers preferred ecosystem services > CAF > Runoff-susceptible environmental conditions | If I try to explain to you what the big problem in Uruguay is in relation to the Santa Lucia, you have three reasons. The first is that our soils are very clay-ish, have a lot of clay, and infiltration is very low, especially when wet. Here, you have better soils, and an infiltration rate of five millimetres per hour. What happens when you have a rainstorm? In Uruguay, sometimes you have a rain of 100 millimetres in one hour. The 95 millimetres of water run outside of the soil; you have no opportunity to retain this soil. It's really quite different from Argentina, as Argentina had an infiltration rate of 50 millimetres per hour and the rain can infiltrate the soil. You have no river, you don't have creeks, it's really quite different from Uruguay, Uruguay has a lot of water run outside. The second problem is that we have a slope of more or less than 3-5 percent, which increases the risk of movement of small particles of greater size in the soil that contain a lot of phosphorus, which is very, very, very complicated. And then these particles end up in the water, and the big problem is that the rivers and small creeks are full of mud, full of soil, and when you have them in summer, they are not deep. What do these do? They increase the movement of the phosphorus in the solution, and if you have a little rain, this phosphorus moves to the Santa Lucia River, contaminating it. The third reason is our rainfall condition. In Uruguay, you either have no rain or a lot of rain. It's different from other countries, where you have seasonal rain, but here it may rain on any day. This is very difficult because, for example, in Australia, there is a station, Ellenbank, where they have studied a lot about this problem of phosphorus in the river because the Moral River is the only source of water for the population of Melbourne. Australia has a big problem because the only source of water is the Moral River, and they made a control about the foot for the movement to the river; they have a station called Ellenbank.  Interview 24 English: 3 - 3 (0) |
| Barriers preferred ecosystem services > CAF > Time-scale of problem structure | It's a problem that you need to resolve for a long time, and it's not easy to resolve quickly. You need to take care of this problem for a long time; it's a political challenge to resolve over a long time, that's my impression.  Interview 24 English: 13 - 13 (0) |
| Barriers preferred ecosystem services > CAF > Traditional views producers | I believe the farmers know how important it is; nobody wants to put money into the river. It's not a good idea, but probably they need more information about the crops and how to use the fertilizer better; that's maybe the idea. They don't listen to any information of this kind; for example, you try to tell your forefathers not to worry if the rain is coming and to wait at least 10 or 20 days before it starts. They never listen; the government says something like this, but I read in a lead bank that a lead bank says okay to the farmers; it's playing. It's important not to put the forefathers in the field if the rain is coming, but here nobody in the government talks about this thing, and there are a lot of easy things that you can advise the farmers about. Our farmers are quite intelligent—more intelligent than people think, really—and they quickly understand and improve things.  Interview 24 English: 11 - 11 (0) |
| Barriers preferred ecosystem services > CAF > Willingness of producers to implement buffer zones | They know a lot, they are quite intelligent, and it's easy to introduce the problem. In crops, it's quite different because sometimes they are a person that rents the land and knows how the future is not so important, like a farmer or a dairy farmer, who is thinking the land is from the child. It's the future of their children, and then it's a different division related to a person who made a crop. I planted a crop on rented land. I don't take care; perhaps you should pay more attention to this person.  Interview 24 English: 15 - 15 (0) |
| Barriers preferred ecosystem services > CEUTA > Costs buffer zones | and some of these changes can be costly. An investment needs to be made in time, energy, resources, and training. The process is slow, and you are not going to know the results immediately  Interview 2 English: 22 - 22 (0) |
| Barriers preferred ecosystem services > CEUTA > Costs buffer zones > Trust fund sustainable management | the creation of a trust (financial trust fund, red.) that is in charge of the care of the basin that provides drinking water to Quito. This trust receives a part of the water tax paid by the residents. From this trust, restoration, conservation, and agroecological management of the watershed can be managed.  Interview 2 English: 24 - 24 (0) |
| Barriers preferred ecosystem services > CEUTA > Ineffectiveness adaptation via goodwill | I don't believe that goodwill has proven to be effective in these things. I wouldn't leave it up to goodwill. Because of goodwill, we have a percentage of people who will do it, and the rest will not, causing counterproductive effects for both the ecological system as well as for the human action that is being undertaken to restore the native forests (the riparian buffers, red.). Because if one producer sees that another producer is doing it better, that's where it feeds back to the environment, doesn't it?  Interview 2 English: 11 - 11 (0) |
| Barriers preferred ecosystem services > CEUTA > Technical obstacles implementation buffer zones | There are technical obstacles to scientific information to this. There is a need for locally adapted technical obstacles because we do not have proven technologies,  Interview 2 English: 22 - 22 (0) |
| Barriers preferred ecosystem services > CEUTA > Technical obstacles implementation buffer zones > Development local implementation | I am sure this is achievable, but it has to be implemented on a case-by-case basis, according to the shape of the property, the location of the property, and according to the socioeconomic level of the property.  Interview 2 English: 11 - 11 (0) |
| Barriers preferred ecosystem services > CEUTA > Technical obstacles implementation buffer zones > Control system intermittent grazing | And that is where the possibility of intermittent grazing comes up, and how it would be regulated. One possibility would be that the producer would have to notify the municipality each time they make grazing, or whatever the local authority is. And there could be a control system that could be subject to inspection. But it would seem to be that it could be a possibility, to have that limited number of times you can do it on an annual basis.  Interview 2 English: 11 - 11 (0) |
| Barriers preferred ecosystem services > CEUTA > Time-scale problem structure | A certain level of faith is needed to carry this process out. You have to believe that there is a joint act of involvement with everyone.  Interview 2 English: 22 - 22 (0) |
| Barriers preferred ecosystem services > CEUTA > Time-scale problem structure > Pilot studies | Let's see, I believe that you simply have to follow the path of experimenting with pilot projects, to take the small steps that can be taken to do this research to let it permeate into public policy.  Interview 2 English: 24 - 24 (0) |
| Barriers preferred ecosystem services > CNFR > Expansion agricultural frontier | I don't know if this drought has not been an opportunity, perhaps, for what I was saying earlier, to expand the agricultural frontier in some places, I don't know, but probably yes. In general, when watercourses retreat, humans take advantage of this to gain ground, but I don't know if that's happening. And well, I think it should also be an opportunity for people to become aware, right? What always happens, I have the problem identified and I run off to look for a solution. I need water, I need a dam, I need food for livestock, I need water to fix the crops. Well, maybe what we always talk about, and it's the big discussion I saw today, is you have to get ahead of those kinds of problems.  Interview 5 English: 34 - 34 (0) |
| Barriers preferred ecosystem services > CNFR > Lack of information | Not all farmers know about them and not all farmers apply them.  Interview 5 English: 16 - 16 (0)  It has been very common in Uruguay that producers, in their eagerness, conquer new areas to produce or to build or to develop projects, advance on very fragile areas and this then generates problems. Now, for example, when people naturally start to get into the areas that they can't enter when the causes are normal. But what happens? I cultivate, I do something, I intervene and then when the water regime normalises, everything floods. and it floods even more because I eliminated the vegetation that was protecting me and that acted as a containment of the riverbed. In addition to that is the fact that I affected the biological diversity, the fauna, and the services provided by these natural environments, especially in terms of containing the overflow of the rivers.  Interview 5 English: 18 - 18 (0)  it seems to me that it's more of a cultural issue because people understand that this is necessary, that what they may see as something that is going to harm them in the short term. That it has no benefit for them, that's why it has already been studied  Interview 5 English: 26 - 26 (0)  And furthermore, many of these management practices within the buffer area and outside the most productive areas, have also been studied to have been beneficial. Not only for the environment and biodiversity but also for the producer family. These are longer-term processes.  Interview 5 English: 26 - 26 (0) |
| Barriers preferred ecosystem services > CNFR > Lack of information > Empowering (younger) producers | There has to be the will that this process of developing the Santa Lucía river basin has been going on for 10 years, it has been going on for 15 years. We have to aim to give more strength to the younger people, who are the ones who are going to last the longest and will be the future managers of the area.  Interview 5 English: 28 - 28 (0) |
| Barriers preferred ecosystem services > CNFR > Lack of information > Providing producers with technical and economic support | If I want to reach out to producers so that they start implementing measures in the areas that we define as buffer zones and they need support, give them technical support, and some economic support.  Interview 5 English: 28 - 28 (0) |
| Barriers preferred ecosystem services > CNFR > Implementation costs | Another obstacle could be the lack of resources or the timing of the processes for these things to happen. There are bound to be conflicts in the middle, there always are. But if we have patience and we know that these are long-term processes and we manage to sustain them over time and provide the necessary resources, we should get a better result in the long run. So, here I added two more obstacles, in addition to the cultural aspect, the availability of resources to be able to implement the plans that are agreed on in these areas  Interview 5 English: 26 - 26 (0) |
| Barriers preferred ecosystem services > CNFR > Traditional views producers | And to be able to develop activities, whether they are productive or rational management, is very important. Because if not, otherwise it's annoying. That is a lost area. People call the native bush "la mugre" (grime; dirt; filth; muck, red.). That's what bothers me. I can't go into that pasture because I have native bush and the cows get lost, they get punctured, and they get caught by snakes. It's like something that gets in the way.  Interview 5 English: 24 - 24 (0)  I think the first obstacle is the cultural barrier of the people. I don't think all people are willing, or at least willing to comply in a very rigorous way with these kinds of things.  Interview 5 English: 26 - 26 (0) |
| Barriers preferred ecosystem services > CNFR > Traditional views producers > Dialogue between stakeholders | But I believe that the solution should always be through dialogue  Interview 5 English: 28 - 28 (0) |
| Barriers preferred ecosystem services > CNFR > Willingness of producers to implement buffer zones | And they should not be imposed, because if you say to someone who has a dairy farm, "no, you can't do dairy farming anymore because you are polluting with effluents”, a person who has dedicated themselves, a family that has dedicated their whole life to it, you leave them out in the cold. In short, this reconversion is not so simple.  Interview 5 English: 16 - 16 (0)  I think the first obstacle is the cultural barrier of the people. I don't think all people are willing, or at least willing to comply in a very rigorous way with these kinds of things.  Interview 5 English: 26 - 26 (0)  If I were a rural producer, I don't know what I would think. I would imagine I would because it's a matter of conscience, but I'm becoming aware of someone else's resources. So, I think there might be an obstacle there, to say well, maybe not all the people who reside in those areas are willing to comply with a plan of conservation.  Interview 5 English: 26 - 26 (0)  The first obstacle is going to be to get so many people in agreement that we have to begin to intervene in these areas and that something, as a first thing, we may come to believe that we are going to lose. I lose a part of the productive area; I lose sovereignty over a piece of territory that belongs to a different entity. Well, there is often the fear that if I leave the forest up there, then the forest is going to come and will continue to advance toward where I am.  Interview 5 English: 26 - 26 (0) |
| Barriers preferred ecosystem services > CNFR > Willingness of producers to implement buffer zones > Provision producers with technical support | We have to provide resources. Organisations are there for a reason. They can also be good channels for those resources.  Interview 5 English: 28 - 28 (0)  If I want to reach out to producers so that they start implementing measures in the areas that we define as buffer zones and they need support, give them technical support, and some economic support.  Interview 5 English: 28 - 28 (0)  So, sustaining a long-term process by providing resources and giving time  Interview 5 English: 28 - 28 (0)  And when I mean resources, I don't just mean money, but also knowledge, technological alternatives, and technical support to be able to support producer organisations.  Interview 5 English: 28 - 28 (0) |
| Barriers preferred ecosystem services > DINAGUA > Communicating and informing | the challenge lies in communicating the correct information to the public. For instance, there was a discussion in a watershed commission that blamed the OSE for not managing the flooding effectively, which the OSE quickly defused. Unfortunately, the situation is polarised, and it is challenging to change people's opinions. Environmental issues, particularly water issues, are highly polarized. There is a kind of complexity in the Santa Lucía basin, and the margin for change of opinion of the parties is very minimal.  Interview 6 English: 33 - 33 (0) |
| Barriers preferred ecosystem services > DINAGUA > Imbalances between institutions | The Ministry of Environment does not have the resources to implement these measures, while the Ministry of Agriculture does. The biggest obstacle is clearly the ministry, not the producers. There are interests that are taking precedence over the general ones, and this is conspiring against the environment. There is a reason why they don't have the resources. Same thing happens with the newly created minister, which is given practically no facility. So, the money is going somewhere else. The political power doesn't care about justice or strengthening the judiciary. OSE has a lot of budget difficulties, and their budget is only a social tariff (non-privatised, red). While it is good that it is social, they don't pass on general revenues to implement the policies that have to be implemented. Therefore, drinking water and the protection of watercourses are not as important as they say they are. The budget needs to go to hire technicians for the ministry, and the political power needs to care about these issues. The budget was 9 for housing and one for the environment and water when the MVOTMA was in charge (ratio of 9 to 1, red.).  Interview 6 English: 29 - 29 (0)  The treatment of OSE is also problematic, as they are not given sufficient investment. This issue is not whitewashed, and it is frustrating that opportunities are missed to address it properly.  Interview 6 English: 31 - 31 (0) |
| Barriers preferred ecosystem services > DINAGUA > Lack of economic incentives for implementation buffer zones | The subsidy policy is my responsibility, and the policy of subsidies or compensation or expropriations, is not there.  Interview 6 English: 14 - 14 (0)  There are no economic incentive instruments as the literature tells us.  Interview 6 English: 18 - 18 (0)  The Ministry of Environment does not have the resources to implement these measures  Interview 6 English: 29 - 29 (0) |
| Barriers preferred ecosystem services > DINAGUA > Management problems buffer zones | The ban on tilling was implemented, which led to problems of buffer zone management because the lack of tillage led to weeds.  Interview 6 English: 20 - 20 (0)  First, it is a challenge for the management that is clearly not prepared for it.  Interview 6 English: 29 - 29 (0)  However, no measures are being taken to address this issue  Interview 6 English: 33 - 33 (0) |
| Barriers preferred ecosystem services > DINAGUA > Misalignment with property rights-culture | Uruguay does not have a culture of property rights for reasons of general interest, and it is complied with and it is enforced. That's like the standard of the institutional culture of ours. The same thing happens in the east, well, it's like a cultural issue of Uruguay’s environmental policy management, because it is historically one of regulatory management; there are no economic incentive instruments as the literature tells you.  Interview 6 English: 14 - 14 (0)  This type of instrument is down the sleeve of environmental economics, and Uruguay needs to implement a paradigm shift. For example, in the river basin commission, the most radical people say that the basin must be expropriated and turned into a protected area, like the Hudson River in New York. However, in Uruguay, this is not even considered. Neither incentives nor exemptions were foreseen  Interview 6 English: 18 - 18 (0) |
| Barriers preferred ecosystem services > DINAGUA > Political priorities | it seems that the political power doesn't care about these issues and that the agenda is not prioritizing them.  Interview 6 English: 31 - 31 (0)  Despite the severity of environmental issues, the press fails to take a serious approach towards them.  Interview 6 English: 31 - 31 (0) |
| Barriers preferred ecosystem services > DINAGUA > Stakeholder perceptions | For me, they are a qualitative change in agricultural management. Several argued that it was a tool for the Ministry of Environment to get control, because they problems with getting control, regarding satellite monitoring systems and other things that were implemented, but well, they were only half-implemented.  Interview 6 English: 12 - 12 (0)  There is a whole group of people that have taken it to the basin commission who believe that there should be no forestry at the headwaters of rivers because the eucalyptus trees affect absorption of water (infiltration).  Interview 6 English: 24 - 24 (0) |
| Barriers preferred ecosystem services > DINAMA > Climate resilience of the agricultural system | This has meant that the availability of water for crops in the soil has been very low. Going into the summer, this low availability of water in the soil, coupled with high evapotranspiration due to the high temperatures that have been recorded well above historical levels, has caused many hectares of crops planted for winter reserves to be lost. Some of these were planted for grain harvesting, which means that both current and future production is compromised. At the moment, producers are facing difficulties with food and water. In fact, the population of the country is also struggling to access drinking water.  Interview 12 English: 34 - 34 (0)  We cannot go and tell producers that regulations do not allow animals that have access to the strip to take water from the river, as it's not the most politically correct thing to say. In other words, the production system is not ready to face a drought such as the one that occurred in the history of Uruguay.  Interview 12 English: 36 - 36 (0) |
| Barriers preferred ecosystem services > DINAMA > Climate resilience of the agricultural system > Creation water-, multi-predial-, and food reserves | it occurs to me that this situation could be foreseen by generating water reserves, multi-predial reserves, and food reserves.  Interview 12 English: 39 - 39 (0) |
| Barriers preferred ecosystem services > DINAMA > Climate resilience of the agricultural system > Promotion best-management practices | By implementing regulations to promote better management, more hectares could be cultivated to prevent these situations from happening.  Interview 12 English: 39 - 39 (0) |
| Barriers preferred ecosystem services > DINAMA > Costs buffer zones | Ultimately, one should pay for the environmental service that the site provides to those who in some way own it and carry the burden of it. But it is a concept that economists find quite difficult to understand. In other words, on one hand, you lose money, but on the other, you gain. However, sometimes you need to create an environmental account. Environmental accounts are not popular, but in Uruguay, I have seen a few people do it,  Interview 10 English: 34 - 34 (0)  Then the downstream land is purely public management. At least we can do the calculations to see if it is enough or not, if it can be explored or not. However, there are issues of whether it is taxed as general income. It cannot be. It is a legal mess.  Interview 10 English: 34 - 34 (0)  There are issues of justice to consider as well. The consumer pays for a lot of things, including all the input and OSE tariff, which includes the cost of the water, employees, treatment, and inputs. As a result of the use, it is a circuit.  Interview 10 English: 34 - 34 (0)  It's not like that tax that the government announced in the last few months, the low network of its tax reduction bill. You won't see the improvement process tomorrow or even after three months. We are talking about processes, and raising the cost of water by one hundredth, and the truth is that I will see the improvement process within five years from then onwards.  Interview 10 English: 34 - 34 (0)  We have discussed it before, I imagine, in all committee meetings. In all the committee meetings and all the governments, nobody quits. I think it is a mistake - I say this sincerely - that no government, no matter what kind of government, assumes that water, for example, should be charged for. It should be charged for, even for ethical reasons. Because you can use a public good as precious as water for free and still, in some cases, sell it like a premium water, bottling it and selling it, right? I mean, it's not even water for irrigation, for the plants, for later selling a product. No, no, it's water that has already been bottled and sold.  Interview 10 English: 36 - 36 (0)  The problem is that this is complex to implement and has much higher costs.  Interview 10 English: 43 - 43 (0)  The state has to assume it has a cost, and it will require a higher cost in terms of people or instruments to protect the environment and water.  Interview 10 English: 45 - 45 (0)  How many are willing to protect the environment despite the higher public spending costs? I really don't know. I don't think anybody knows, and nobody can answer that, especially when it comes to things that have invisible benefits, right?  Interview 10 English: 45 - 45 (0) |
|  | Today, only the producer is paying for it.  Interview 12 English: 17 - 17 (0)  There is no support from the government to exempt the contribution. The only precedent that is appearing and that we are seeing are some situations carried out by the National Institute of Colonisation, which has asked its settlers to apply a differential rate in the cost if their registration is within the buffer zone. It is not yet general but rather on a case-by-case basis where each settler goes and asks the National Institute of Colonisation to apply the differential rate in the cost. They pay rent for that area. So, if the registered area instead has a rent of 8,80 dollars, so to speak, they multiply it by one. But if it has an average rent of 8, they multiply it by 0.75. In other words, they receive a discount that somehow doesn’t feel like a payment for them, but a waiver of rent in that area.  Interview 12 English: 17 - 17 (0)  the biggest thing, I think, is the budget and that. The budget issue is also about who is responsible; who decides, the municipality, or the social welfare bank? I mean, here they took this control mechanism as a tool. The Social Welfare Bank does the controls.  Interview 12 English: 48 - 48 (0) |
| Barriers preferred ecosystem services > DINAMA > Costs buffer zones > Fertiliser tax | I have a proposal for water: put a tax on fertilisers. I am exploring the theoretical possibility of putting a tax on fertilisers that attacks the source, the cause of the pollution, and transfers it somehow.  Interview 10 English: 34 - 34 (0) |
| Barriers preferred ecosystem services > DINAMA > Costs buffer zones > Water tax | We discussed the possibility of charging for water in the Santa Lucía river, at least. We even charged OSE for the water. In other words, it may be a public company, but it uses a public asset. It could pay, for example, one peso per cubic metre or one cent per cubic metre. That gives around two million dollars a year, and you can subsidise all the protection of the watercourse itself. It's an idea that closes on all sides. Of course, the consumer pays, but the amount is minimal, right?  Interview 10 English: 34 - 34 (0) |
| Barriers preferred ecosystem services > DINAMA > Economic loss producers | It is important for producers to have full knowledge of the importance of the function they fulfil before applying them. The issue is that the producer may lose an (productive, red.) area when applying this buffer zone. And as a consequence, it loses economic income, let's say.  Interview 12 English: 17 - 17 (0)  There is no support from the government to exempt the contribution. The only precedent that is appearing and that we are seeing are some situations carried out by the National Institute of Colonisation, which has asked its settlers to apply a differential rate in the cost if their registration is within the buffer zone. It is not yet general but rather on a case-by-case basis where each settler goes and asks the National Institute of Colonisation to apply the differential rate in the cost. They pay rent for that area. So, if the registered area instead has a rent of 8,80 dollars, so to speak, they multiply it by one. But if it has an average rent of 8, they multiply it by 0.75. In other words, they receive a discount that somehow doesn’t feel like a payment for them, but a waiver of rent in that area.  Interview 12 English: 17 - 17 (0) |
| Barriers preferred ecosystem services > DINAMA > Complexity economic incentives | What happens is that the Ministry of Economy tells you that it’s not possible. We've had all kinds of things, but in general, they are not very open to it.  Interview 10 English: 34 - 34 (0) |
| Barriers preferred ecosystem services > DINAMA > Complexity economic incentives > Political openness incentives | Although now there is a certain openness because the Ministry of Economy has brought up the issue of carbon financing. Maybe there can be a little more vision.  Interview 10 English: 34 - 34 (0) |
| Barriers preferred ecosystem services > DINAMA > Lack of information | Knowledge needs to be generated to establish a baseline, which we don't have. The question we frequently ask is whether the buffer zones are working today. This initiative began in 2015, eight years ago. Are the buffer zones retaining or contributing nutrients to the water resource? Are they sources or sinks? We lack this information at the ministry or country level.  Interview 12 English: 21 - 21 (0) |
| Barriers preferred ecosystem services > DINAMA > Lack of monitoring and control | As we don't have any form of control, we understand that grazing would be allowed at low stocking rates.  Interview 10 English: 17 - 17 (0) |
|  | No, satellite control is not possible. We cannot see the animals.  Interview 12 English: 45 - 45 (0)  The plan had a battery of 11 measures, but none of them are regulated. They can be sanctioned, but there is no regulation to back up that suggestion. Measures 3 states that the 31 parts per million of phosphorus are also not regulated. Work is being done to regulate these measures. If something is regulated and can't be controlled, it's a big issue.  Interview 12 English: 58 - 58 (0) |
| Barriers preferred ecosystem services > DINAMA > Lack of monitoring and control > Development monitoring algorithm | The working group is developing an algorithm based on NDVI, which takes into account the phenological changes of the crops. The algorithm is still in the training phase.  Interview 12 English: 54 - 54 (0) |
| Barriers preferred ecosystem services > DINAMA > Lack of monitoring and control > Implementation drones for monitoring | Someone mentioned the use of drones, since nowadays this technology is advanced. It could help to reach producers who are difficult to contact, especially in irrigated properties. With a drone, we could easily access the buffer zone and monitor compliance without the need for the producer's authorization or presence. It's not that far-fetched of an idea.  Interview 12 English: 45 - 45 (0) |
| Barriers preferred ecosystem services > DINAMA > Legislative complexity | Currently, the regulations don't permit it, so legally, it cannot be done.  Interview 12 English: 21 - 21 (0)  First, legal - that's the first one.  Interview 12 English: 23 - 23 (0)  In order to apply new things, the Ministerial resolution that is regulated today says that you can't do anything. So, you need another regulation or ministerial resolution that enables grazing to be downgraded, or enables the sowing of maize and legumes for the extraction of phosphorus. There needs to be a regulation that allows it. All of this takes time. You have to create new regulations, disseminate them, incorporate the producers into the change of culture, and get all the actors in society and the government to agree, so that when they reach the authorities, they can be signed. Management is not easy, and agreements between political forces can be complicated. It takes time to get all the actors to agree.  Interview 12 English: 25 - 25 (0)  The legal part is also an issue, but I don't think it's very difficult also because it was already implemented.  Interview 12 English: 49 - 49 (0) |
| Barriers preferred ecosystem services > DINAMA > Long timescale before noticing the benefits | Benefits that cannot be monetized or that are very long-term escape the attention of one or two governments. So, let's say I make a great plan for Santa Lucia river, but the benefits of that will only be seen in 20 years. You may think that's funny, but it's true. We are talking about decades here. When we published the first action plan for Saint Lucia, I was one of the people in charge of making it clear that the results would only be seen in a decade or two. That was demarcating and devastating, especially for decision-makers and politicians. They are doing something, but they are being told that the benefits may only be seen 10, 15, or 20 years down the line. It's true that things may improve after that time, but maybe not, because if we only focus on that one thing and neglect other important factors, the deterioration will continue.  Interview 10 English: 45 - 45 (0)  We need to be nuanced and balance visible actions with necessary actions that may not be immediately visible but are important for future generations. This issue is a clear case of intergenerational concern. What we do may not be immediately visible, but it's important for the future.  Interview 10 English: 45 - 45 (0) |
| Barriers preferred ecosystem services > DINAMA > Overconfidence in the resilience of the water system | I think that we are ill-prepared, everyone is ill-prepared, but Uruguay is very confident in its water resources, thinking that it has surplus water resources. I think that this is a mistake. In fact, the drought is proving it, it is dry. There is no water anywhere.  Interview 10 English: 41 - 41 (0)  When you protect a watercourse, you are talking about the biology, the quality, but also the quantity. For example, there are uses of water that are perhaps excessive today. Maybe you are making excessive use of the source for drinking water that can't hold any more. And then comes the whole other way of life, from another source that doesn't give me enough to put in. Climate change permeates this and distorts all of this.  Interview 10 English: 41 - 41 (0)  We can enter into an issue of deterioration of land use, sediment runoff, and nutrient runoff, in short.  Interview 10 English: 41 - 41 (0) |
| Barriers preferred ecosystem services > DINAMA > Selective communication with producers | we don't have a survey of all the opinions. We only receive opinions from isolated people. The producers who don't like the measure are the ones who reach out to me. Sometimes it's someone whose own private life limits them, but not always.  Interview 10 English: 23 - 23 (0) |
| Barriers preferred ecosystem services > DINAMA > Time-scale problem structure | Political timing is another major constraint when it comes to environmental measures. The environment has this great disadvantage of being subject to political timing that doesn't always fit in with other things. I may be making a monetary policy, but it's very difficult to see the results of environmental policies within political timeframes.  Interview 10 English: 45 - 45 (0) |
| Barriers preferred ecosystem services > DINAMA > Unclear legislative responsibilities | However, it's difficult to pinpoint whose responsibility it is to drive this change. From the regulatory standpoint, the Ministry of Environment should take control and ensure proper dissemination and communication of regulations, but this was not the case in 2015.  Interview 12 English: 27 - 27 (0)  we give them support from here. That is in the community of Santa Lucia. That doesn't happen, there is no one from the ministry who is in the territory working in the team with us. There are those who work in the team with us, and there is another culture of work and what is coming.  Interview 12 English: 28 - 28 (0) |
| Barriers preferred ecosystem services > DINAMA > Willingness of producers to implement | Many of these points are difficult because putting a measure in place can be unfeasible in practice. It's not easy, especially given the idiosyncrasy of the Uruguayan countryside. There is a strong culture of land appropriation by producers, as if they can do what they want. Not everywhere, but we do need to work against that culture a little bit.  Interview 10 English: 21 - 21 (0)  When you approach small watercourses, you are essentially interfering with a producer's private property. They don't see it as just a small course, but rather as their property.  Interview 10 English: 26 - 26 (0)  It is not going to be easy to admit interference with a public good. There is a problem. It is clearly stated in the constitution of Uruguay that water is public, but the little course of water that passes private land is not protected as such. So, we have a problem of competition. The land is private property, the water is public, but the coastal border is public because the border is also part of the water.  Interview 10 English: 32 - 32 (0) |
|  | And then, changes in habits. People have adapted to doing their work and tasks in a certain way, and they will have to adapt to the proposed changes. For instance, asking the producer to graze animals at a low stocking rate means fencing or delimiting that area. The producer will not be able to move all the cattle that they had in a lot to the new grazing area, but they will have to rotate some animals. This will mean more work. Change is always difficult, and I think that the obstacle is changing the culture as a whole. It's not just the people, but the animals also have a certain culture. They are used to taking on a certain behaviour with the water, and it's natural. In the river, in the streams, nobody sees it as a problem, and in reality, I don't know if it is a problem, but it is an important nutrient source for the body of water when the animals are there. We were talking about it today, and it seems crazy to say that cows can't drink directly from the river without getting sick. So, there needs to be a pretty important cultural change.  Interview 12 English: 23 - 23 (0) |
| Barriers preferred ecosystem services > DINAMA > Willingness of producers to implement > Economic incentive | We need to provide a subsidy or in some way a lower rate of taxation on land, at least in the area that has been set aside as a farming zone. It seems to me that it is even fair because you are changing the rules of the game a little bit for a producer. Until recently, nobody said anything to you, although you had to protect the course with a margin. Now we come to say that you have to do it, and then I think that the state should at least give you an exemption for the payment of the tax that you have to pay for the land. I think that would be the way to go.  Interview 10 English: 34 - 34 (0) |
| Barriers preferred ecosystem services > DINOT > Complex public institutionality | I think that the obstacle has to do with how it has to be incorporated into a complex public institutionality, because many actors are involved with the issue, so articulating that is very difficult. That's why they are that using instruments that have legal validation in such a way that they are binding with the policies, although it is not enough with the political will. There must be approved instruments that are binding with the policies so that it is not enough with the laws and the instruments for things to happen. Of course, the articulation is needed, but you have to be bound by some norm, by some instrumental framework. Because if not, things end up being weak due to the situation. You have to overcome that situation, that on the one hand, that watercourses and their buffer zones, one has to refer to local governments, municipalities, departmental governments, various ministries and also commissions for public-private integration or social integration, such as basin commissions, rural development commissions. You have to overcome that complex institutionality.  Interview 21 English: 36 - 36 (0) |
| Barriers preferred ecosystem services > DINOT > Complexity economic incentives | But the economic impact for certain activities will be different because you restrict them. What happens is that you, in the economic impact, have to have sufficient integration of the economic benefits that the country has. Then, it costs in millions to make the water drinkable. Well, we have to have ecosystem services in balance so that this territory, as well as this soil, ends up pouring the water that is then made drinkable. What happens? This, with that, is decisive for the investment that you have to make afterward and to have all the economic benefits of having treated it well from a niche; otherwise, you pay for them afterward, right? So, this issue is not so easy; it's not the typical impact of, well, how much does it cover me? How much does this area cover me? So, I have a productive limitation on 30% of my land, and therefore my profitability dropped by 20%, right? It's like a global equation, which at a legitimate level has to be done, right? You need to account for private interests to understand the equation  Interview 21 English: 25 - 25 (0)  The other seems to me that is what we talked about today, that is, when the measures end up affecting the private land in Uruguay. There is a story of what we are talking about now, there’s a history of this; the use without limitations of the rural productive soil. Regulatory bodies have never been in a position to restrict, although there are examples, right? During the twentieth century these policies, which in a way put forward principles that are tangible, but also are a bit intangible, of general interest, raise limitations to the free use of private property. Then, here is the main obstacle that appears is damage compensation, completely out of time of another time, but that today in Uruguay is very present. Every time one poses limitations, even if they are general environmental objectives that are considered by the constitution, damages compensation appears in the discussion. If you are limiting me, compensate me, because you are restricting all my free use and my economic possibility. Therefore, there is harm without harm. If I have to compensate, it is unreal and has no support. At this time there is no legal support, And well, that’s why there is culture and it’s a big obstacle to the advance of this because ultimately, those obstacles then end up being political. And ultimately, they end up affecting these political issues.  Interview 21 English: 36 - 36 (0) |
| Barriers preferred ecosystem services > DINOT > Legislative complexity | And obviously, once you have the approved instrument, you have to agree to finish implementing the limitations, to finish implementing the location of the productive activities through the plans. But, on the other hand, you also have to finish orienting the livestock programmes, the economic incentive programmes, and the promotion of productive activities. The environmental permits also appear.  Interview 21 English: 21 - 21 (0)  Interviewer 2: But it is not illegal to prevent some activities and place restrictions on activities on the land, right?  Interviewee: But of course, this is clearly a difficult point because the history of Uruguay is a history that is marked in some way by productive activity in the development of the free will of private activity in the territory. So, well, environmental laws are from the 21st century; they are not from 50 years ago, so we are in a kind of maturation of the issue, aren't we? It is being imposed, but the legal and continental frameworks are in place, so don't come here to be judgmental because you're going to disagree.  Interview 21 English: 26 - 27 (0) |
| Barriers preferred ecosystem services > DINOT > Weak institutional structures for environmental protection | And also, the territorial structure for the control of protected areas and DINACEA is still very weak in this respect. In Uruguay, action is reactive and not proactive, people react to complaints, whereas it's necessary to act proactively. Inter-institutional coordination is essential. If you want, I can show you a presentation I gave. I don't know if you have any questions, but if not, I'll tell you a little bit about St Lucia.  Interview 21 English: 11 - 11 (0) |
| Barriers preferred ecosystem services > Producers > Buffer zone saturation | The plant has to grow. If it's a tree, it grows. If it's a pasture, somebody has to eat it and it has to grow again. If it doesn't, that circle comes to the point where it stops. In other words, if there is no permanent growth, it gets to the point where the nutrients are no longer removed from the soil, because there is no one to consume them, do you understand?  Interview 4 English: 44 - 44 (0) |
| Barriers preferred ecosystem services > Producers > Climatic pressures on agriculture | One of the challenges is the recent drought that devastated everything. It's like putting out fires, and it's hard to plan for the future.  Interview 13 English: 36 - 36 (0) |
| Barriers preferred ecosystem services > Producers > Complex bureaucratic process registration buffer zones | That's another issue that I would have to clarify. I have it registered, but every year they force you to do the paperwork and things. All the bureaucracy makes it difficult for the producer who does good management, who has a buffer area, who maintains the park-like formation. The paperwork for not paying the real estate construction for that area should be automated. This is something that should be suggested.  Interview 13 English: 63 - 63 (0) |
| Barriers preferred ecosystem services > Producers > Complexity implementation productive solutions | If they are going to give you three hectares of land somewhere else, it's no good because today, what do you do with the irrigation structure? You have to start all over again, from scratch. It seems to me that we would leave the production. In some cases, and in some units, they are all the same reality. There are many different realities. But in some cases, they would have to stop production. Yes, the buffer zone would have to be forested. But there is no simple alternative to that. You take two hectares of land from me here and give me twenty hectares in Colonia and I say no. Because it is impossible to manage it from a productive point of view. The state could give the land to me and I sell it. But from a productive point of view, it is not a solution. Neither the lease, nor me.  Interview 16 English: 70 - 70 (0)  In other words, there is already a whole investment in the property, which is also affected. Of course, and it's also efficient if you respect the integrity, the chain of all those investments that go from working on a road, to making a cutwater, to laying irrigation pipes. This is associated with the machinery you have or the effort you had to make, the tools you have bought. It is a very integral concept that if you go into it and think about it, you can deflate it completely. And the family producer, who has no credit, in 105% of the cases you don't have any savings either; to face a crazy idea of, well, I'm going to transform everything. That producer will probably leave. Or sell the rest of the land to another larger producer. You take him out of the game that way.  Interview 16 English: 70 - 70 (0) |
| Barriers preferred ecosystem services > Producers > Complexity of locality buffer zones | The thing is that the buffer in other areas is located in a cleaner area of the countryside. And maybe today people have it integrated into an area of farmland. And maybe that's where you have an impact more from the point of view that there is an area that you used to have integrated, that you planted corn, sorghum, and that today you can't plant. I mean, the buffer doesn't affect me very much, because of the proportion I have of coastline in relation to the area. If the field had a different shape, and I had a lot of coastlines, maybe those 50 meters would imply a larger area. In other words, the first thing that stands out for me is the importance of differentiation. Not all situations are the same, and there are certain circumstances where production remains unaffected, as is the case here, for example. However, in other places, production may be impacted. This topic is crucial in relation to the previous one, as until now, we have been managing it within the crops and the area that is typically sown.  Interview 4 English: 54 - 54 (0) |
|  | if people are properly educated and things are done well, water quality will improve. However, the challenge is to take action and put plans into motion. For instance, the ministry will have to educate, monitor, and draw attention to things that need improvement. Although I am not skilled in detecting inconsistencies, it is essential to understand that certain soils have specific agronomic attitudes. It is not suitable for an Argentinean with an agricultural mindset to come here and try to farm in a way that does not align with Uruguay's practices.  Interview 13 English: 74 - 74 (0) |
| Barriers preferred ecosystem services > Producers > Complexity sustainable livestock management | the issue of shadow, which has a direct impact on production. It was quite a challenge for me to manage water and manure, while also ensuring the welfare of the animals and workers on the farm.  Interview 13 English: 34 - 34 (0) |
| Barriers preferred ecosystem services > Producers > Complexity use of cattle in wetland zones | but as I said today, that would only be possible for a few months of the year and that's impractical.  Interview 16 English: 43 - 43 (0) |
| Barriers preferred ecosystem services > Producers > Costs buffer zones | It is difficult to maintain if you can't sell the firewood. If they tell you, look, you can sell the firewood, maybe the costs will balance out. Because in reality that's the biggest cost, it's not the fencing (the maintenance, red). It's having to clean the forest.  Interview 4 English: 54 - 54 (0) |
|  | They are very long-term investments. I mean, these investments not only involve putting money in but also ensuring their maintenance.  Interview 13 English: 34 - 34 (0) |
|  | I won't lie to you; I am currently paying the owner of the field 30,000 pesos for those 100 hectares. I haven't calculated it for years, so I don't know how much I have left in the hectare more generally. I don't have any kind of problem though, so we can work it out together. I don't know what the costs could be, but not for the field itself.  Interview 14 English: 92 - 92 (0)  Honestly, I can't tell you off the top of my head how much money I'm making, and I've never actually done the calculations. However, in my case, this offer is limiting because I can't expand my business since there aren't any farmers coming to the area. On the other hand, I have a contract, but I haven't been able to come to an agreement in 10 years.  Interview 14 English: 94 - 94 (0) |
| Barriers preferred ecosystem services > Producers > Costs buffer zones > Economic incentives | The solution, first of all, for me, is to educate and to look for some kind of tangible benefit for the producer to act as a guardian or protector. For example, being exempted from property tax would be a good thing. The problem is that I don't do it because you have to do all the paperwork all over again. In other words, it gives the feeling that they want to block everything instead of making it easier for you. So, the government should say, 'these are the buffer zones,' and identify areas that could have certain productive uses. The producer should be incentivized by offering certain exemptions or benefits for doing good for society. But the government should also give them something in return because being an agricultural producer in this country is difficult, and you have to constantly invest, and fight against the climate for instance. So, I think it is important to prioritize the role of the agricultural producer for the good of society and, in some way, to seek some kind of reward for doing things well in these buffer areas. Perhaps an exemption would suffice to motivate the producer  Interview 13 English: 76 - 76 (0) |
| Barriers preferred ecosystem services > Producers > Disturbance from exotic vegetation | No, not really. It doesn't benefit me in any way. We have some invasive species.  Interview 4 English: 15 - 15 (0)  Interviewer 2: I see. So, you can't really control the invasive species, right?  Interviewee: That's correct. In fact, it's an impediment to good management. If you can't get there, you can't control the invasive species effectively.  Interview 4 English: 18 - 19 (0) |
|  | If you compare it with Google today, it is impressive how the vegetation has advanced. In reality, it is often a response to poor grazing management, where sometimes due to overgrazing, invasive species that may be native, such as hawthorn, start to appear or advance.  Interview 13 English: 9 - 9 (0)  For Ligustrum and Gleditsia (Gleditsia triacanthos), we cut them and apply herbicides.  Interview 13 English: 93 - 93 (0)  Actually, I have a lot of Paspalum (Paspalum notatum, red.). It's a long bean that has become invasive, and we want to prevent it from spreading. The most complicated thing is the blackberry, and we use Tordon, an herbicide, and try to use a rotavator to maintain the stump. But there's still a lot to do.  Interview 13 English: 95 - 95 (0) |
|  | What I fight against a little bit is the Zarzamora that is coming up in the field. I don't touch what is in the riverbed because it doesn't bother me. We are dealing with the spots where the Sarsamora appears, which then start to close and eat up the field. It becomes impenetrable. So far, we have kept it at bay. There are fields that are 100 hectares, but only 25 are usable. The rest is a whole forest. The Sarsamora and other invasive species such as Espinillo (Vachellia caven, red.) are taking over. Even the Zarzamora (Black berry, red.) is drowning out the Espinillo now. They are like curtains. The man leasing the land from me uses it for dry cattle, but there are parts that the cattle don't use. I am paying rent for 100 hectares, but I only use about 20-30 hectares. It's impressive how overgrown it is, and we are far from the river. Even from the fence, you can see that it's super overgrown in the forest. The solution I see is to make a piece of field in the future. It's a long way off, but we have to transform the land by tying up the Espinillo and clearing things little by little. I would have to do it myself because the one I’m renting from is not very interested. I'm still paying for the hectare of land, but only taking advantage of 20-25 hectares.  Interview 14 English: 43 - 43 (0)  , the area hasn't been tended to and is becoming overgrown. But now, there are also invasive species such as the wild boar. They have taken shelter there, and because of this, it's becoming a problem. If we don't tend to that area, we could create problems not just on our property, but also on others since the bush is much thicker there. The pigs bring in blackberries, hawthorn, and other things. Mostly it's the pigs, though. They hide there and attack cows that are giving birth. We've had a lot of problems with that in the field next door.  Interview 14 English: 45 - 45 (0)  the Zarzamora, you see, it kills the tree, and if it doesn't, it doesn't make it grass down there. Let's say you are standing half a meter from the edge of the field. Everything beyond that point is soil, and it would be beneficial if you could clear the area and turn it into pasture. However, the problem is that the vegetation is so dense that it doesn't just let you, it chokes the forest and overruns the trees. If you were not allowed to manage a larger area, you would have difficulties with the reproduction of certain plants. The immediate area around the buffer is already being used for grazing, but there are small hawthorn trees that they are trying to preserve. There is a riverbed about 300 meters away, and the area closer to it is filled with dense vegetation that is not suitable for livestock. One of my neighbours is renting a field that has been invaded by unwanted plants, leaving only small patches of usable land for livestock.  Interview 14 English: 60 - 60 (0)  Invasive species don’t just invade, it chokes the forest. It overruns over the trees. In other words, if you were forbidden to manage a larger area, you would have problems because of the reproduction of Zarzamora, for example. I don't know, maybe I'm talking nonsense, dismantling or clearing up the bush so that you can plant other species there. Maybe I can show you the map so we can look at it together (the interviewee proceeds to show his land on google earth, red.). This is my field, basically. Here is the street, and here we are coming this way. That this is already inaccessible here. And well, here we are taking advantage of it with grazing because the issue is that there are big trees, but there are also small hawthorn trees, which we are trying to insulate. I'm telling you, on this side, which is the closest one, this must be 300 meters from the riverbed. And, well, and here we have a lot of meters more than with invasives. You see, these are the 24 hectares that we've been able to use, and this is all the other land.  Interview 14 English: 62 - 62 (0)  Unfortunately, the meadows are gone, so there's no grass for the animals. I'll have to trick them by putting them on the land until I can regenerate the grass, but I've run out of area to plant more.  Interview 14 English: 64 - 64 (0)  It seems to me that by removing this invasive plant, in some way, as it takes hold more on its own, to see more grass, the ground will be more covered, right. I mean, nowadays you don't have any tools, you just put in more money and hope for the best. The thing is, in this part, you could still do it with a backpack or something because it's still around the forest. There are acres and acres that you can't enter. And the vines are so big that they are as thick as a finger, you see, it's not an insignificant little thing.  Interview 14 English: 66 - 66 (0)  Just because they can't fight it, it's a wasted field. If you can't fight it with a tool or whatever, it's a lost field. It's strange that you can't make it productive.  Interview 14 English: 68 - 68 (0) |
| Barriers preferred ecosystem services > Producers > Increased risk to fires | there is a lot of biomass that's there, and I thought about it this year because of the fires. That's a lot of biomass that has a high fire risk.  Interview 4 English: 33 - 33 (0) |
| Barriers preferred ecosystem services > Producers > Legislative complexity | the next challenge is implementing the measures and ensuring that they are followed by producers. This can be difficult because each producer may encounter different difficulties depending on the specific situation, they are in.  Interview 4 English: 64 - 64 (0)  Another potential obstacle is determining who deserves support and who does not, as there may be situations that require support and others that do not. This requires differentiating between situations with more or less change and considering the area that is affected, as this can have a significant impact on productivity  Interview 4 English: 64 - 64 (0) |
| Barriers preferred ecosystem services > Producers > Limited interactions government and farmers | What happened is that it seems to me, I lost track of this because after 2014, I stopped participating. I think that in the case of the protected area of Santa Lucia, they didn't manage to approve a management plan that other protected areas in the country have. Because if one enters the Ministry of Environment's website, which has now changed a bit, I never saw that the management plan had been approved and that it had the signature of the authorities, unlike, for example, the management plan for the Laguna Rocha basin or other protected areas. So, it seems to me that we worked on it, we participated in an action plan, agreements were made, but then the executive and the government institutions had to move forward and design the management plan, which obviously the government approved by decree, but now I was checking, and I did not find the management plan. I don't know if a management plan was approved at the national governmental level in the case of Santa Lucia. I am saying this because from everything I am going to talk about later, it seems to me that what started off with a lot of strength later became deflated, and that is my final summary.  Interview 11 English: 7 - 7 (0)  It seems to us that the work has not been completed and approved because when one enters the presidential web page of gub.uy, enters DINAGUA or DINAMA, searches for riparian buffer zones or the protected area and gets to the Santa Lucía river, it says credit proposal presented, but it doesn't say management plan approved, while in others, it does. But well, that is my impression, that the implementation of a plan was slowed down and it was not really implemented and carried forward.  Interview 11 English: 7 - 7 (0)  However, my impression is that no regulation or legislation has been disseminated regarding the buffer zones. I have never seen a formal presentation of the management plan for the Santa Lucia River. Secondly, there is no capacity or proposal to develop each of the projects contained in what could be a management plan to achieve the final objectives of not having a negative impact on the quality of the water. I did not see the Ministry of the Environment or the Ministry of Agriculture promote projects to help producers implement ways of producing and reconverting the way in which each of the chakras is being worked. In other words, there is a lack of proactive attitude to generate changes in development projects.  Interview 11 English: 11 - 11 (0) |
|  | I've been to the basin commission and talked to some agronomists from the ministry, and I'm a bit annoyed because they suddenly told producers to implement them. But they should visit and explain certain things to them, but I see that they don't do anything. Here, for example, it's also important to understand a bit of history. In these areas, for instance, the Uruguayan Society developed in the Santa Lucía River basin because of water supply, but also because of the basin that supplied Montevideo with fruit, milk, vegetables, and so on. In the past, dairy farms were located quite close to Montevideo, and at certain times, dairy farms were banned in Montevideo.  Interview 13 English: 17 - 17 (0)  One tries to do things right, but we have a commission that does nothing.  Interview 13 English: 17 - 17 (0)  At a certain moment, I took the decision to do this, and my fellow producers in the group told me that it was nonsense because I had put in all this extra work, make the lagoons, etc. So, what happened, I started, and I ran up against the lack of those desk agronomists who don't know anything and who don't go to the countryside and who wanted to block my approval. So, well, I had to justify it, so I sort of drew a little something to get it in.  Interview 13 English: 32 - 32 (0) |
| Barriers preferred ecosystem services > Producers > Negative impacts sand mining on the watercourse | there's a big upheaval of the gullies and sand pits. There are 8 of them in just a few kilometres. The sand pits have to make a path to get to the river, and at a certain point, the riparian forest starts, but then they extract sand in an industrial way without any control. All of this affects the watercourse. Meanders are erased, and the river loses its meandering nature.  Interview 13 English: 27 - 27 (0)  But I'll show you... See, for example, here my neighbour has a sand deposit, and he razed everything to the ground, see? Here, there was still vegetation, but it was all blown up to get the sand. And then... Here, we have another sand deposit. The river meanders here. There is another sand deposit here, and it's full. Another sandpit can be found here. So, I think you have to live and let live, but here, there is another sand pit. There is no control. So, by mining the sand, you take out the riparian forest. Suddenly, you had dunes that were fixed with vegetation. They took out the vegetation and sand, and then this patch happened. You can see a sand trap, two sandpits, three sandpits, four sandpits, five sandpits, six sandpits, seven sandpits, eight sandpits, and here is another one. Then, you come here and see the bridge. So, in a few kilometres, everything that has been damaged is from the river.  Interview 13 English: 55 - 55 (0) |
| Barriers preferred ecosystem services > Producers > Non-informed producers | As a producer, I've never received any official communication, neither from the commission nor from the ministry of agriculture. I don't know what I have to do in that area. Does that make sense? To me, that's a major flaw in the system. There's no recommendation, suggestion, or obligation. What is well-known by the ordinary people here, some comply with it and many others don't, is that animals cannot go to the river to drink water. This was publicized when the Santa Lucia measures were taken (when the Santa Lucia River Basin Action Plan was implemented, red.). But then, compliance with these measures depends on the individual. There are people here who don't have fencing. These regulations were already in place before, such as the fact of fencing against the rivers.  Interview 4 English: 48 - 48 (0) |
|  | One problem, for example, was that the river basin committees stopped functioning, and civil organizations were no longer invited to participate. Another issue was that I don't think they ended up approving a management plan. In those projects, I imagine there were plans for reconversion, information, and training for producers in the area on how to manage their productive systems in the buffer zones, and support for productive alternatives.  Interview 11 English: 27 - 27 (0)  There has never been any monitoring, training, or awareness-raising on this issue. No workshops or alternative measures have been put in place. That is to say, in the commission or development society in my area, no one has ever provided training or information from the government. There are three rural development societies: one in Lunarejo on Route 49, another in Bella Vista, and another in Velázquez. There are producers and directors who are there, and as far as I understand it, this issue was very popular at a certain moment, but then it didn't move forward. Obviously, if you take a tractor against the coast of the Santa Lucía river and work all over, and someone takes a photo of you and publishes it, the municipality comes, sees the dynamics, and fines you. But that's not how things work because you have to be caught or reported by someone.  Interview 11 English: 28 - 28 (0) |
|  | I am unsure that with the law, today or tomorrow that that area can't be grazed in. In our case it would kill us because it would be almost those 18 hectares that we can’t use.  Interview 14 English: 41 - 41 (0)  I don't know much about the regulations either, and I don't think so. Can you ask to move it forward? Because it's an invasive species. And in that case, if I gave you permission, you would move it forward, take it out, and put me in the field that you are renting. You would enter it, right?  Interview 14 English: 72 - 72 (0) |
| Barriers preferred ecosystem services > Producers > Over-productivity of producers | The big problem, for example, is that Uruguay is a livestock farming country. The issue arises when 100% of farmers come and want to farm where they shouldn't.  Interview 13 English: 29 - 29 (0) |
| Barriers preferred ecosystem services > Producers > Political focus on agriculture | The problem that we have in Santa Lucia is the same problem that the rich areas of the world have. Any highly productive watershed in the world, even in Europe, has associated pollution and nutrient recycling problems. So, in the case of Santa Lucia, the management plan should take these circumstances into account. We have to be aware that we are in a highly food-producing area. On a different societal level, it's difficult to balance because I always ask myself, why do we producers have to be responsible? Many times, we are identified as polluting watercourses when people throw rubbish out of the window or in a dump truck, throwing whatever they want. Producers often produce food for a thousand families. So of course, the system produces some surplus, but we have to make a balance of what it also contributes  Interview 11 English: 23 - 23 (0)  If we are going to question the production model and the responsibility of society as a whole, not just the producers, then we should also question the consumption model in cities. We should ask ourselves how society views producers, for example. No citizen thinks that they are a potential polluter when they flush the toilet or put waste in the bin, but if a producer drops in three pebbles more fertilisers, they are polluting. If someone orders twice as much food as they need and puts half of it in the bin, nobody questions it, but that's phosphorus and nitrogen going somewhere. It's going to destroy some water source or the sea, yet nobody questions it. When someone turns on an air conditioner all day long, or public bodies leave all the lights on, nobody questions it. But on the other hand, if a farmer is driving a tractor and burning fuel, people think it's horrible. But we have to understand that if the consumer likes to consume such things, they also have to realize that in order to produce it, there was an energy cost and waste left over. And that's why, although I think awareness is important, I don't mention it often. When it comes to dealing with the problem, many of the measures to mitigate the impacts of water quality have costs. So ultimately, who bears that cost at the state and society level? Does the producer have to bear it by moving 100 meters away from the stream? Why? Society as a whole consumes what that producer produces. It's like saying we will remove 50 square meters from your flat so that you can grow trees or an organic vegetable garden, so you don't pollute as much. I don't think we've matured as a society in Uruguay in terms of questioning whether watersheds and production systems are polluting. This message hasn't reached the city level yet.  Interview 11 English: 24 - 24 (0) |
|  | Well, I think we are talking about the only source of potable water supply for more than half of the country, including the city and metropolitan area, and it will be very important. However, I am also interested in pointing out that when talking about the sources of pollution in the Santa Lucía River, production has been systematically attacked. In the case of Canelones, which has more than 25 populated centres including industries, I don't hear about their pollution problems. I think it pollutes a lot. You have to see the streams that come out of the rocks in the city of 100,000 inhabitants. You have nylon bags stuck in the riverbeds for 5 meters. So, it seems to me that the big villain of the story is not the productive areas. Especially in recent years, there has been a lot of progress in the biological protection of crops, where the number of insecticides has been significantly reduced, and the use of herbicides has been much better managed. I know that there are highly polluting activities such as dairy farming, which generates a lot of water throughout the process, but I think we need to broaden our perspective. We are not the worst in history, nor are we saints, and we need to recognize that.  Interview 16 English: 29 - 29 (0) |
| Barriers preferred ecosystem services > Producers > Political non-willingness economic incentives | But I don't believe the government would come and tell me that. I think if we give them the opportunity to propose it, the government wouldn't accept it. And especially not this government. It's anonymous, so it could be anyone. Let's see, I don't believe this government in anything. No, I think we should determine more about the use and management of the soil. I totally agree that there would be things you can't do here (in the buffer zones, red.). Its now, taken to the extreme that you can't work it.  Interview 16 English: 59 - 59 (0) |
| Barriers preferred ecosystem services > Producers > Economic loss producers | We agree with the action plan that was drawn up, as it was the result of a negotiation process. For instance, we saw our producers making a pact there. If, for example, I have a farm that is 200 meters by 300 meters, and a gully runs along one edge, a 100-meter measure would mean that I cannot use one-third of my farm. This has a significant impact on a productive unit that has 4 or 5 hectares to work with. Our participation aimed to establish measures that would not affect the structure or productive matrix of the farms. It's one thing to have a property in front of the first line of action, which has a coastline against the Santa Lucía River. It's another thing to have a property with a coastline against a main stream, and there are also many properties on secondary tributaries or streams that are further away, and that are part of the basin, and in which there are many smallholdings and small properties. When it came to regulating, our concern was that putting very large distances between small plots could render this productive structure unviable. There are even smaller plots, such as 3 or 4 hectares. If you establish a 100-meter measure, it could mean that half of the plot cannot be used. I hope you understand. The last part was a bit difficult to explain.  Interview 11 English: 13 - 13 (0) |
| Barriers preferred ecosystem services > Producers > Technical obstacles implementation | the number one obstacle would be if the definition of the distance that the buffer zone itself is going to have, right? From the watercourse, how many meters would it be?  Interview 16 English: 27 - 27 (0)  Of course, in a unit of 8 hectares or having 2 hectares of wetland you have a significant percentage of land that will produce for us  Interview 16 English: 27 - 27 (0)  it would take away a large part of the land we are working on, because the intervention in the buffer area, according to my interpretation, is to determine rules in which you cannot do a huge number of things, including moving the earth. Well, and in a fraction, in a productive unit that is 300 meters wide, they put 100 meters on each side of the watercourse, and there goes more than half of the farm. It would make it unviable. That is, economically, it would be an absolutely negative impact.  Interview 16 English: 57 - 57 (0) |
| Barriers preferred ecosystem services > Producers > Traditional views producers | Because what happens if you are seen cutting down a tree or pruning, it's as if you are a sinner. When in fact, if this is rational management. The issue is precisely how to manage these renewable resources in a sustainable way, from an economic and environmentally friendly point of view. So, it's a whole issue. And there you are doing some kind of new management.  Interview 13 English: 49 - 49 (0) |
| Barriers preferred ecosystem services > Producers > Unclarity management responsibilities | But it seems to me that there should be more emphasis on informing and providing management guidelines. That's what I think is lacking today, especially in areas where the buffer is part of a very large area with the same management. If I were to fence the buffer, I'd have to clear out a whole area of bushland to put up a wire fence. That's actually a whole area that I manage. And in case you were told, for example, to put up a wired fence and you couldn't have access to cattle, you would be losing something. In my case, the buffer is 100 meters, but over there it's only 50. It's a little bit of an alteration. From a business point of view, it's not much, and it wouldn't affect me. However, it would be a tremendous expense because I would have to fence 250 meters, as you can see, in length by 10 meters wide. When we made the fencing, we calculated 10 meters wide what you have to do to make an exclusion. In other words, experimental fences are obligatory. And it allowed you to have a strip of 50 meters on each side. In concrete terms, the implementation did not affect me from a productive point of view. It's a very small area. If it has to be done under the conditions as they are now, then, if I do it, it's going to cost more.  Interview 4 English: 50 - 50 (0)  The first obstacle is ensuring that there is a clear and adaptable guideline in place for what needs to be done in different situations. Without this, it is impossible to effectively convey ideas and fulfil them.  Interview 4 English: 64 - 64 (0) |
|  | That's actually theoretically fine, but practically it has some implications. For example, land between 15 and 20 meters in front of a small watercourse far from the Santa Lucía, a secondary or tertiary tributary. If the producer doesn't put livestock on it, who looks after it? Who maintains it? Because it turns into forest, it becomes semi-woodland. The water source doesn't flow because plant debris starts to collect, and the streams become clogged. Who takes care of this maintenance? It's easy to say, but then there has to be maintenance of the watercourse. Because if the producer cannot exploit it, then who cuts the grass there and who maintains the tapestry? Because then it becomes bushy and attracts pests like foxes, and hares. Many animals interact with the cultivated land. So, with small and intensive farms, it is difficult to see who manages that area. If the producer cannot access or manage it, then who does? This has always been a latent issue for me.  Interview 11 English: 13 - 13 (0)  Nowadays, the regulations, as they are, nobody controls them, and in some situations, they do not contribute to the objective and can have the opposite effect. For instance, if I'm not allowed access to my strip of land in the last 15 or 20 meters against the stream, in 4 or 5 years it will become a jungle of trees and everything. When the water comes, it will strip it, flooding my land and reaching farming areas it didn't reach before, taking all the phosphorus and soil, which could circulate freely due to its natural cause if it were kept with a low cover.  Interview 11 English: 19 - 19 (0)  For me, an aspect that is very important is the governance of the plan. Who is responsible for all of this that we are talking about? Ultimately, it is the DINAGUA and the Ministry of the Environment. Who is responsible for livestock farming? What is the executing unit in charge of this nowadays? For example, one of the measures in the plan was that producers could not fertilize if the soil analysis of the plots within the zone showed more than 32 ppm of phosphorus. However, nobody has ever checked that in their lives. It is wrong because producers do not even hire a technician? They just apply whatever they want.  Interview 11 English: 27 - 27 (0) |
| Barriers preferred ecosystem services > INIA > Buffer zone saturation | Regarding the access of livestock to the area, some people argue that it helps to remove nutrients that build up due to the lack of herbivorous fauna. However, we are investigating this issue to determine when and at what time vegetative build up occurred in our ecosystems. Domestic animals can replace herbivorous fauna, and the advantage of managing them is that we can control when to enter, when to remove biomass, and when not to enter. However, if animals are left permanently in an area, they do not remove anything, and the nutrient build-up becomes more challenging to address.  Interview 15 English: 39 - 39 (0) |
| Barriers preferred ecosystem services > INIA > Buffer zone saturation > Double buffer zone | Another alternative I have often suggested to my colleagues theoretically is to have a double buffer zone. One is an area where the natural vegetation tries to be maintained and is not touched mechanically, and where there are no large extractions. The other is a nutrient-trap crop, which is like a strip that borders the natural vegetation and acts as a nutrient trap. This crop can be harvested, but it must be taken out to remove as much biomass as possible, which traps all the nutrients that come through runoff. That would be ideal. It also has another cost, but it can be used to provide fertilizer. By recovering everything that was lost upland, it helps in revaluing the fertilizer. However, in the strip itself, you have to see.  Interview 15 English: 39 - 39 (0) |
| Barriers preferred ecosystem services > INIA > Buffer zone saturation > Mechanical harvesting | Therefore, removing nutrients may require mechanical harvesting.  Interview 15 English: 39 - 39 (0) |
| Barriers preferred ecosystem services > INIA > Closure watercourses | There is another obstacle, for example, which could be the partial closure of watercourses. Not a permanent closure, but one that allows entry only when it is deemed convenient. However, this limitation of entry, like the one that exists today, also has a cost. This infrastructure limits the entry of livestock. There may be cases where it is people who cause deterioration due to public use, but they are much smaller in scale. Livestock, on the other hand, is omnipresent everywhere and causes deterioration of the buffer zones around watercourses due to their unrestricted access. Installing physical barriers such as fencing would require significant investment, and in areas where livestock drinks water from the watercourse, additional investment in drinking facilities would be necessary. However, even with the installation of drinking troughs, animals may not use them, and this measure may not be enough to prevent access.  Interview 15 English: 39 - 39 (0) |
| Barriers preferred ecosystem services > INIA > Costs buffer zones | Well, one of the main obstacles is the cost. Controlling invasive exotic species is expensive and tedious, as those of us who have done it here know. Depending on who faces those costs, it can be more or less viable.  Interview 15 English: 39 - 39 (0)  There is another obstacle, for example, which could be the partial closure of watercourses. Not a permanent closure, but one that allows entry only when it is deemed convenient. However, this limitation of entry, like the one that exists today, also has a cost. This infrastructure limits the entry of livestock. There may be cases where it is people who cause deterioration due to public use, but they are much smaller in scale. Livestock, on the other hand, is omnipresent everywhere and causes deterioration of the buffer zones around watercourses due to their unrestricted access. Installing physical barriers such as fencing would require significant investment, and in areas where livestock drinks water from the watercourse, additional investment in drinking facilities would be necessary. However, even with the installation of drinking troughs, animals may not use them, and this measure may not be enough to prevent access.  Interview 15 English: 39 - 39 (0) |
| Barriers preferred ecosystem services > INIA > Costs buffer zones > Economic incentives | A prize (financial incentive, red.) would be a good start. For this to happen, it depends on public policies that provide incentives, such as economic incentives or payment for ecosystem services, or on direct action from the state. For example, even if it is in private establishments, but that the restoration action is carried out by a third party that goes and does things and does not depend on the owner of the land.  Interview 15 English: 39 - 39 (0)  The solutions are to have resources to cover the costs of restoration or management of the buffer zone.  Interview 15 English: 43 - 43 (0)  In these cases, and not only in these cases, we need to have some kind of payment for environmental services, some kind of scheme to make it viable. Because it has costs, and if it's left to the owner, nothing will happen. The owner is not directly affected or at least not aware of the state of that buffer zone. If that does not happen, and there is no market price, the incentive does not come from public policy, but from the fact that the products they produce are worth more because they are in better conditions.  Interview 15 English: 43 - 43 (0)  The incentives on the market side are more challenging. It has to come from public policy and resources to support that. A first step of payment for ecosystem services is tax exemption.  Interview 15 English: 43 - 43 (0) |
| Barriers preferred ecosystem services > INIA > Disturbance from exotic vegetation | Invasive exotic species can colonize and eliminate native biodiversity.  Interview 15 English: 31 - 31 (0)  Invasive species often dominate and displace other species, reducing the food and refuge sources for wildlife. This can lead to overpopulations of certain species, disrupting the balance of fauna populations.  Interview 15 English: 31 - 31 (0)  The invasion of the Ligustro (Ligustrum lucidum)F, an Asian tree and the main invasive species in Uruguay, generates a bare undergrowth with no vegetation. This can cause erosion, as there is no soil cover to prevent it. In contrast, the ecosystems surrounding watercourses in Uruguay are normally very diverse, with tree and herbaceous species, and an undergrowth with a lot of herbaceous plants. When these areas are invaded with exotics, this diversity is lost, and the integrity of the riparian zone is not always maintained.  Interview 15 English: 31 - 31 (0) |
| Barriers preferred ecosystem services > INIA > Effectiveness economic incentives | What I see is that this weighs very little in the producers' economy, and much less for intensive producers. So, it's not really the tax exemption that weigh in. They may have other types of taxes that weigh more heavily on them, but they are not related to the environment's management and the management of their establishment. There has to be something more, like the soil law today, that obliges the declaration of a use plan, and everything else that is mandatory, not optional. I know that measures that are more coercive, i.e., more of an obligation than anything else, are not very attractive. But in some cases, there will be no other option than this type of measure. The ideal would also be to have measures such as payment for ecosystem services or some other type of tax exemption measures. It could be part of things, but it seems to me that economically, it doesn't weigh so much. That is why it is not so successful. Even so, you can register native woodlands. For example, if the area is woodland and ecologically, that woodland has very poor quality, it is a green stain. It is there, and they exonerate you. However, you end up paying at least for an incomplete ecosystem service. That is the same question as the producer who is suffering from this. It is like a repeated band-aid that the same person has today to deal with. With the producers in mind for advice, the truth is that I don't know what to do.  Interview 15 English: 45 - 45 (0) |
| Barriers preferred ecosystem services > INIA > Lack of knowledge producers | If it's left up to the owner or private owner of each thing, it's not going to be difficult, except in the case of some people who are already aware of how important it is and make interventions in their establishment because they want to, not because anyone rewards them.  Interview 15 English: 39 - 39 (0)  Even with the economic obstacle removed, another obstacle can be knowledge. There are not many people trained in these issues, and even fewer with experience. But I believe that this is a less relevant obstacle if the resources to implement these measures are available.  Interview 15 English: 39 - 39 (0)  So, in reality, people are bothered by the Espinillo because the Espinillo bothers them. It is a cultural issue. It is not productive. As long as you put a number on it, it is productive. The problem is also that if it bothers them, they start to generate a problem. If it begins to be a productive problem, it is generated as a pincushion of low bushes and all thorny, not a tree as it would be in its natural environment. That does not prevent access below, but such is the case with other invasive species. For example, the Ulex europaeus, which is a tree, not a shrub. It is a plant that invades natural fields, it is especially thorny, and if they burn it, it activates the germination of seeds. Almost all the measures worsen the situation, and here there are many trees.  Interview 15 English: 47 - 47 (0) |
| Barriers preferred ecosystem services > INIA > Lack of knowledge producers > Education | Generating knowledge is also an investment, perhaps a smaller one, but it allows us to provide recommendations afterwards, not strict rules, but guidelines.  Interview 15 English: 43 - 43 (0) |
| Barriers preferred ecosystem services > MGAP > Access water for livestock | In addition to the loss of area that can in some way pay back with some production at the farm level, there is also loss of access to water for the farm. Many of the fields are managed for livestock farming. In the Santa Lucía basin we have agricultural areas, livestock areas, and farmers need to be able to ensure water in all the paddocks that the farm has for adequate management with animal welfare.  Interview 17 English: 35 - 35 (0) |
| Barriers preferred ecosystem services > MGAP > Complexity exclusion cattle from buffer zones | Wiring, apart from being expensive, involves a lot of investment in terms of ensuring water distribution. And not everywhere, we can have enough water in sufficient quantity and quality to ensure this at the level of the establishments.  Interview 17 English: 35 - 35 (0) |
| Barriers preferred ecosystem services > MGAP > Conflicting visions between stakeholders | Well, I think that the plan is excellent, that plan was developed when I was in the direction of natural resources, but I think that there's a problem, it's more social maybe, no? Because in technical discussions, maybe you can agree, like what is the importance, for example, of the soil management plan or other policies or other actions, but sometimes it was difficult because the discussion was not technical, it was more like, let's say, philosophical. I think that agriculture is a bad thing, so anything that comes from there will work, and sometimes the discussion was more like that and not technical.  Interview 19 English: 17 - 17 (0)  This commission was very big, with too many people there, so sometimes it was difficult to agree on actions because of that.  Interview 19 English: 17 - 17 (0)  Honestly, I don't know, because I've been there almost three years, and I didn't see what to do with some of the obstacles when there are these hard discussions where nobody hears what the other one is saying. And I think this, and I'm not going to move from my way of thinking. I think that that is very difficult.  Interview 19 English: 27 - 27 (0)  But in this case, it was very difficult, because people from the city, most of the time didn't know what happens in the countryside. They think that farmers are enemies, that farmers are contaminating everything, and that they don't care about the environment, they think that all that. And in that situation, I think that it's very, very difficult to solve the problems.  Interview 19 English: 27 - 27 (0) |
| Barriers preferred ecosystem services > MGAP > Conflicting visions between stakeholders > Smaller sub-commissions | I think the one thing that will be good, it's to make some sub-commissions with not so many people. So maybe sub commissions with 10 people that maybe when people are getting buffer zones, they are going to supply information, to share the what and how, things like that. Because sometimes in this big meeting, there are maybe 50 people or more from different organisations, so maybe it would help to make sub commissions where these different things or topics can be discussed, and then in the plenary, make the presentation, I don't know, maybe. Because in my opinion, to take decisions you need knowledge if you can get it. You have to take action because you know, sometimes you don't know, but there is knowledge because there is a group in university, a group of people that study this for 40 years, and they can tell that this is working like this. And if you don't have the knowledge, try in the middle of the process to generate it, support research, and things like that.  Interview 19 English: 27 - 27 (0)  See, not new commissions, you know, smaller commissions. Not a new one. For example, the 60 people, I don't know how, but sometimes when, when I used to be there, there were maybe 70 people there. It's very hard to discuss with too many people. So sub-commissions, you know, maybe 20 people working buffer zone, other 20 in, so can say for example, other stuff, things, and then once, for example, in six months or, or once in a year, all the sub-commissions get together in the big commission and then maybe present the results of their work and the, yeah, not a new one because we have a lot already of things.  Interview 19 English: 35 - 35 (0) |
| Barriers preferred ecosystem services > MGAP > Costs buffer zones | It's very expensive. I don't know who's going to pay for it, because there are many kilometres of wire fence.  Interview 17 English: 35 - 35 (0) |
|  | The other thing that I remember at the river basin commission is that the cattle were not able to get into the river, so the farmers have to have drinking stations where the cattle go and drink water far away from the river, and that has a lot of costs, no? They have to get a pump, and also, they didn't like to do that because of the cost and the complications, no?  Interview 19 English: 15 - 15 (0) |
| Barriers preferred ecosystem services > MGAP > Costs buffer zones > Tax exemption legislation | Part of implementing a buffer zone should, in some way, be legislated with tax exemptions.  Interview 17 English: 42 - 42 (0) |
| Barriers preferred ecosystem services > MGAP > Lack of monitoring and control | It is difficult to enforce control, especially in cases of violations, such as cutting down native forest, soil erosion, or unauthorized water usage.  Interview 17 English: 46 - 46 (0)  the second package of measures does not regulate the buffer zone, so no warning or regulation is set at the system level.  Interview 17 English: 54 - 54 (0) |
| Barriers preferred ecosystem services > MGAP > Disturbance from exotic vegetation | There are also areas in the basin towards Lavalleja that have invasive Tojo Ulex europaeus, which affects the natural field and forest. However, there are other invasive species that are more prevalent in the countryside, and I am not familiar with them.  Interview 17 English: 52 - 52 (0) |
| Barriers preferred ecosystem services > MGAP > Economic loss producers | But I think we have, for example, a defined buffer zone in Santa Lucía is 100 metres from the watercourse of the Paso Severino Lake, San Francisco and Canelón Grande. We are talking about 100 metres, the area, the property, the producer. In other words, it is being defined not to use an important percentage of the area of a farm. This makes it difficult to implement the measure. Especially in those cases where there is no native forest. I don't have an update on the status of the situation in those cases, nor an update on what control has been done.  Interview 17 English: 23 - 23 (0)  if you look at, for example, the Paso Severino dam, there are many producers whose land reaches the edge of the lake, and we have constructions in that 100-metre buffer established by the resolution of the 2013 action plan, areas of farms, pastures, estates. Which is an important percentage of the area of the farm, of the producer, and you're saying that they cannot use it any more, because, in other words, if we follow the strict rules set out in this resolution, nothing can be done. That's why I say it's difficult to implement.  Interview 17 English: 25 - 25 (0) |
|  | knowledge, like studies, because sometimes when we talk about buffer zones, we say, no, let's plan, let's say, native species or like it's a buffer zone, there has to be something completely natural and we don't know that. For example, I think that there have to be some high, as I said, high-yielding plants, but we need some investigation. Local, national maybe, to design this buffer zone, because it depends, for example, the width, it depends on the land use, the slope, and other things, and sometimes we need to know more to make good designs, no? Because sometimes we are applying things for the research institution, or there is a very good work that made USDA a lot of years ago that helped a little bit in design, but it's very important, I think, for small farmers, fundamentally, that those buffer zones have a good design, no? For example, 100 meters, maybe it's too much for a farmer that has a small farm. So, I think that we need to know more about these buffer zones, in size, in species that we can use, and how frequently we can maybe put some cattle on them; maybe two hours a day, I don't know. I'm thinking this, but I think that it would be better to have more knowledge about them.  Interview 19 English: 25 - 25 (0) |
| Barriers preferred ecosystem services > MGAP > Economical water distribution | The main obstacle is obtaining an economical water distribution. It may not be feasible to have an intake in the watercourse that has permanent water all year round with the depth. In other words, investments are required in the design, at the level of the property, and investments in the form of electricity provision.  Interview 17 English: 40 - 40 (0) |
| Barriers preferred ecosystem services > MGAP > Management problems buffer zones | I mean, a lot of weeds would grow, and then when it comes to the level of a property, some can be good and others bad. That is, it is also an issue to be taken into account, otherwise, it could not desirable for any crop that it could do harm  Interview 17 English: 35 - 35 (0) |
| Barriers preferred ecosystem services > MGAP > Narrowed focus on buffer zones | And to improve all that, you have the buffer zones to help and to support all other things. But if you only focus on the buffer zones, maybe you are not improving, you are not attacking the whole problem. Understand?  Interview 19 English: 17 - 17 (0) |
| Barriers preferred ecosystem services > MGAP > Willingness of producers to implement | In addition, gaining support for these measures is important, which would need funding from the ministry, the World Bank or IDB funds, for water for animal production. Even some projects also had to implement actions in what were the buffer zones, such as preventing the entry of animals, with some future measures. However, these are not subsidies that have reached 100% of the producers, and it does not mean that those producers that have obtained any benefit, or have managed to implement 100% on the whole farm.  Interview 17 English: 40 - 40 (0) |
| Barriers preferred ecosystem services > MGAP > Willingness of producers to implement > Dissemination to producers | A lot of dissemination to producers is needed, to have a guide, a manual on what to do according to the characteristics that I have. The buffer zones that we want to define must have measures, and they have to be supported in some way with normative regulations that not only prohibit, but provide other tools to move towards that goal.  Interview 17 English: 42 - 42 (0) |
| Barriers preferred ecosystem services > Scientists > Buffer zone saturation | And you prevent all that biomass from decomposing and will end up dissolved or associated with dissolved organic matter. That could end up in the water as well. So, you need not only install buffer zones, but you also need to have a mechanism for extracting the nutrients that are in that zone. Because those buffer zones are permanently receiving nutrients from the upstream zones and, well, the nutrients don't disappear.  Interview 9 English: 13 - 13 (0) |
|  | there is a very important effect, and we still haven't answered it, which is that it is said that when the buffer zones are saturated, so you're going to ask me later, there is a risk of saturation, and then the effect of the buffer zones can be much worse because all the phosphorus that is concentrated there is next to the watercourse.  Interview 23 English: 19 - 19 (0) |
| Barriers preferred ecosystem services > Scientists > Buffer zone saturation > Intermittent grazing | They are associated with the soil, or they are associated with the biomass, or they leave in a particular form, or they leave in a soluble form, but they go somewhere. Except for the denitrification, which is the only process of loss, the is the only process of loss. So, we need to do some management. This management has to be well thought out and designed with the objectives of the buffer zone in mind, not the productive ones. You can't put cattle in the buffer zone when it's wet and muddy or during heavy rainfall. Because of this, you have to wait until the soil is firm, so that the cattle don't trample on it, which is a problem of cattle trampling, and you have to manage it in a certain way. You could do it with machinery, but it's not practical and it's expensive. For me, there has to be intelligent management of livestock in the buffer zones. This could be once a year, once every two years, or twice a year, but we will have to assess and test that. There are ways in which you can have cattle without them grazing in the buffer zone. For instance, you can put mobile watering places and shade, so the animals will go there to drink and eat. They will go to the most comfortable place, which is where they have access to better quality water. They don't like to go into the water so much if they have a better supply of water somewhere else. So, they can leave the buffer zone to rest. However, you don't have to define or decide what the best way is.  Interview 9 English: 13 - 13 (0)  Agroforestry often promotes diversity in managing loads and also reduces the risk of invasives, especially with trees and many herbaceous plants in livestock farming, which controls dispersal and maintains populations of invasive species. So, these management measures for buffer zones would also be important. Yes, I think the biggest benefit is that you're going to reduce nutrient inputs. You're going to make the buffer zones more efficient. To me, that's the most significant advantage.  Interview 9 English: 19 - 19 (0)  Well, that's one of the things I discussed with colleagues when we wrote the report, and they suggested that livestock management should be implemented. In addition, one thing that I suggested was to have staff in the field, similar to park rangers, especially in Paso Severino. These people would work with the producers, go around the area and verify certain things, making it easier to monitor management in different ways. For example, if a producer plans to put cattle in the buffer zone, they would have to submit an affidavit stating when they plan to do it and under what conditions. With little in-field monitoring and satellite monitoring, we could keep track of what is happening in the buffer zones. It's not enough to simply look at the buffer zones and say, "something happened here" or "something happened there." We need to explore other alternatives to ensure that they are feasible. You could have some kind of grazing service, for example, so that the cattle that would graze in the buffer zone would not be owned by the producer, but that there would be a kind of lessee who would be the owner of the cattle that are eating in the buffer zone.  Interview 9 English: 31 - 31 (0) |
| Barriers preferred ecosystem services > Scientists > Buffer zone saturation > Vegetative harvesting | another advantage could be to produce bales at the moment if you find yourself in a buffer zone. If you are making a forage reserve and if you make bales, you have an additional reserve for the winter, but that depends a lot on what area you are in and what that buffer zone is.  Interview 23 English: 29 - 29 (0) |
| Barriers preferred ecosystem services > Scientists > Challenges academic culture | Some people here, you know, that work in science, don't know what science is, really, because they think that science is, if they say something, they are the authority, and nobody could, you know, doubt their assertion, and they say, well, I am the professor, you know, who are you? But this has nothing to do with science. Science is data, and one student could challenge a professor, he has new data, more credible data, but many people don't know that. And if you tell this to them, they think, who is this crazy person. I mean, real science is always controversial and new findings are tested with what the people consider is true or not, under some variables. So, it's sometimes frustrating to work in science, especially in some other places in Uruguay where there is still a knowledge gap and scientific authority is not recognized.  Interview 7 English: 31 - 31 (0) |
| Barriers preferred ecosystem services > Scientists > Challenges changes in agricultural management | it's really hard to manage in terms of the ecology, you don't really have a diversity of species that are able to, you know, play multiple roles and roles at different times of the year, because it's kind of dominated by one exotic species. And then the other role is like, while you only have one species that flowers at one certain time of year, and so in terms of, you know, honey production, that's pretty important here in terms of riparian zones. And you're going to have a big part of the year where there's no honey production because there's no flowers. So, I think exotic invasive species are really a problem, both ecologically, but also economically, unless I'm mistaken.  Interview 8 English: 45 - 45 (0) |
|  | The other issue is the change in management of the farm, which implies another variable. Whether I am going to put them here, whether I am not going to be able to, whether I keep them, whether the cattle do not go to the buffer zone, which is something else, and really the traditional farmer who does cattle ranching has less, he has simpler management, unless they are very organized producers, Like these new natural pasture producers, who manage the loads and categories to improve the natural pasture, which are few, the others basically keep cows and go every now and then to see how they are, so everything that involves buffer zones implies not only wire fences but also open drinking troughs. The water troughs have to be maintained; they break; the cows break them, so it is not only an economic cost but there is also a management cost in that you have to do more things. You have to be aware of more variables, and that is another thing that many times Montevideo does not understand or the institutions, especially the environments, do not understand. The part that the minister would understand the most, and well, on the contrary, they get a bit paralyzed because of that, but the environment minister often does not understand that for the producer, it is an extra management that implies work, not only money but work that sometimes is not so easy to do, and then the cost is the cost of decisions, and the cost is above all the resistance that the producer has to the management.  Interview 9 English: 21 - 21 (0) |
| Barriers preferred ecosystem services > Scientists > Challenges research and culture | But there are no resources in education. It's only some sporadic efforts, and the people don't go.  Interview 7 English: 15 - 15 (0)  And they make a meeting, and only all people go because they don't have anything to do with the people that are involved in agricultural tasks. They don't have time, and then they bring, you know, food and refreshments. And some people go for the refreshments, but it's not to work the education process. They don't know what you are talking about, and they also are intelligent. Because if we go and ask something about the buffered zone, they will say to you, "Oh, it is very important." I know this, but they don't want to care about that.  Interview 7 English: 15 - 15 (0) |
| Barriers preferred ecosystem services > Scientists > Communicating and informing | So, there is a very big resistance, and for me, one of the biggest mistakes made by OSE and the Minister of the Environment with his severe steps is that they did not do any kind of work with the producers. That is, they went one day and put up a wire fence; you can't work like that, even though the land belonged to OSE and people had been using it for 30 years. You can't just go one day and put up a wire fence, or you have to work with the producers and explain to them the importance of things. They don't consider that the impact of the water quality is their responsibility, so you can't just make a measure obligatory one day for the next. You have to work with the producers, and the producers in Uruguay are very organized because they are all linked through some rural association or some group; it is not that they are all loose around. They are very organized, so sometimes it is not so difficult to start working with producers; of course, you have to put people in who know how to work with producers.  Interview 9 English: 22 - 22 (0) |
|  | So, I would say that you were trying to do some involvement with the community, but I would also say that the interaction with the productive neighbours, like the local producers, was never very well thought or sold. For example, it's costly to take water, you know, to place out of the reservoir, and some of the plants require it, but that's also some exponential costs, and, yeah, so, and you are not fully, maybe you have some programs to partially compensate it, but then you are taking land away from them, I mean, it's difficult. And then you can do it, but then you need to have some sort of management capacity and enforcement capacity to make, you know, a different equilibrium, like, to move the system to a different state or something.  Interview 22 English: 21 - 21 (0) |
| Barriers preferred ecosystem services > Scientists > Complexity economic incentives | I don't know what kind of economic incentives there are, if there are any. Something else that I've seen that is deterring is when one's landowner protects their riparian zone and then they feel frustrated because the landowner next to them, for example, cut the whole thing down and there were no consequences. So, there can be incentives that encourage people to maintain riparian zones, but also kind of the opposite, where they see that there are consequences or penalties for people who remove riparian zones. That's an incentive too.  Interview 8 English: 39 - 39 (0) |
|  | At the moment I see it only as a loss for them, because it implies a reduction of the area, and that translates into a reduction of income, or it implies in some cases a technological change that also in the short term is going to imply costs. You are thinking about it in a context that today it would be difficult for me to think about in Uruguay, and the alternatives go in two directions, right? Well, on the one hand, to convince us that these activities are incompatible with what is expected for the basin, and therefore there would be many activities that should be prohibited in certain areas. Or on the other hand, as happens in other parts of the world, that these producers would obtain some benefit for not carrying out these activities in these areas. I don't see this second instance in Uruguay today. In other words, compensation, shall we say? Yes, or, well, the framework of payment for ecosystem services, or, whatever framework you want, let's say, so that basically they no longer carry out those activities or with those characteristics in those areas. Today, in the Uruguayan context, I see it as a long way off.  Interview 18 English: 30 - 30 (0) |
| Barriers preferred ecosystem services > Scientists > Complexity of locality buffer zones | a buffer zone has to be adjusted to the local conditions. It has to be evaluated according to the type of basin, the type of slopes, the type of runoff, and the topography of the terrain.  Interview 3 English: 14 - 14 (0) |
|  | so, there is not a lot of management. And then you have these programs I was involved with, where you would try to include trees based on some kind of spatial configuration that reflects the kind of environmental conditions for trees of different lengths, right? So more hydrophilic next to the water are known associations of trees, which would eventually give way to forest, but not everywhere, just in certain regions that had more suitable conditions for forest. So, then you have that kind of thing, and of course you don't have a lot of management because the state, once the program ends, don’t really take care of it. And then you have all these sort of problems where, for example, the owner of the land, which is the water company, doesn't really do land management, so what you have is one guy there who takes care of the reservoir, but he owns livestock, and he actually kind of uses the place for his own good, you know? The other, which starts complicating things because then you have all these other landowners who felt that their land was removed for them, and they knew the persons or the people who were in charge of taking care of the reservoir. So, they ask, hey, you know, can I also put a gate in the fence, and then I come from time to time, use it to feed my cattle, blah, blah, blah, so what can I say? Then you kind of start failing in doing proper management and control of the area, and kind of, we are not very, like, environmentally, just for environmental sake. It's very difficult to have a piece of land and leave it alone, you know, just for some forest restoration or something.  Interview 22 English: 21 - 21 (0) |
| Barriers preferred ecosystem services > Scientists > Conflicting visions between stakeholders | The point is that the benefits for society in the buffers is a bit difficult to answer because within society there are different interests and there is not going to be a single answer, a unique answer. For example, for those who are in charge of the development of agricultural production in Uruguay, the benefits are going to be different from, for example, those of us who get our drinking water from the watercourse. Even at the governmental level, the Ministry of Livestock has different interests than the Ministry of the Environment. Beyond the fact that, well, that in part of the discourse they are aligned and that they make some agreements to move forward in some areas. Or in some aspects, the interests are completely different, even opposed in many ways. For the Ministry of Livestock and Agricultural Fisheries, the development of the buffer zones was a problem, it generated inconveniences some years ago in any attempt to reinforce the regulation.  Interview 18 English: 28 - 28 (0)  Well, the main obstacles are the clash of interests, mainly due to an agricultural sector that has great influence and importance in Uruguay and that through different mechanisms is trying to advance in the Santa Lucía basin. The current context in Uruguay is to allow these actors to continue advancing, to allow industrial agricultural production to continue advancing in the Santa Lucía basin, and even to allow forestry to continue advancing in the Santa Lucía basin. So, the first obstacle I find is the political weight that these actors have, the participation they have in the national economy to allow these types of measures to move forward on the one hand  Interview 18 English: 32 - 32 (0)  possible solutions for these obstacles I see as complicated in the sense that they are opposing interests.  Interview 18 English: 36 - 36 (0)  And something that is worrying, I don't know if you are aware, but there is a whole group of land units that historically have been under discussion whether they can or should be added as forestry priority zones or not. There is a very important lobby by the companies that are pushing for the growth of forestry in Uruguay, such as UPM, for these soil units to be included within the forestry priority zones so that forestry can advance in these areas. And these units are very present in the Santa Lucía Basin, mainly in the headwaters of the Santa Lucía Basin. So, in the event that these areas are finally integrated as priority forestry areas, it is expected that forestation will grow in these areas.  Interview 18 English: 45 - 45 (0) |
|  | There are two things that are not necessarily linked to the structure of the vegetation or the type of vegetation you have or your soil cover, i.e., your land cover, which is basically what determines your capacity to enforce some of the measures. For example, in the Santa Lucia Basin, in this Paso Severino, the idea was that these areas were the property of the water management company, right? It's a public water management company, the OSE. It was; it owned the buffer until a certain limit, which was the maximum flooding area of the reservoir, right? But traditionally, it has been offered to the farmers, so most of the farmers were, you know, actually, in that region, feeling that it belonged to them, right? So, when the OSE goes there and the Minister of Environment wants to implement this buffer, what they did was put a wire all around their lands, which was first interpreted or at least perceived as the land of many of the landowners as a kind of: “now you are taking away land from me”. And that was done quite abruptly, so, you know, but you could argue that otherwise you would never have done it; that was the Ministry's perspective. And so, then you have this stripe, which is, you have this exclosure in many places, so you have this natural regeneration process of passive restoration, right?  Interview 22 English: 21 - 21 (0)  Thus, the problem is that the Ministry of Agriculture is not active in terms of environmental restrictions. They did not want the Ministry of the Environment. There is normally a fight, and that is also political. You have different layers. I think that technically, people can agree between the two ministries. I was working on the National Environmental Plan, one of my previous jobs, and we actually, most of them, the richest, I would say, and the most difficult part of the plan was to sit together with the technicians of the Ministry of Environment and the Ministry of Agriculture. In fact, we have made many agreements. However, if politically, there is no will, like right now, I do not think there is much room for any of those things because now the political leverage of the large producer associations, be it the ARU or the Federacion Rural, is too big. They own the place right now, the Ministry, and the Minister. What is the incentive to do something against your productivity? Why do they invest more in the environment? Right now, in my opinion, there is no equilibrium, but that is a political issue.  Interview 22 English: 33 - 33 (0) |
| Barriers preferred ecosystem services > Scientists > Conflicting visions between stakeholders > Negotiations | The solution to this obstacle could be, on the negotiation side, at least to start by defining some priority areas where it is certain that things cannot be done. Well, for example, a strategy similar to the one that the municipality of Canelones has adopted.  Interview 18 English: 36 - 36 (0) |
| Barriers preferred ecosystem services > Scientists > Costs | There is an economic one, in that making wire fences is very expensive. Although electric fences are cheaper, they are still very expensive, and you have to do a lot of maintenance. So, what you save on the installation, you then have to invest over time in maintenance, which is something that people are not aware of. For example, the environmental mystery is not aware of how expensive it is to make fences and how expensive it is for a producer. Even for a not-so-small producer, a medium-sized producer, or a large producer, it is expensive anyway.  Interview 9 English: 21 - 21 (0) |
| Barriers preferred ecosystem services > Scientists > Costs > Tax exemption | The other benefit is that here the tax does not apply because of the number of hectares. The other benefit that gives you today, is that you are exempted from the contribution of that area, and they exempt you.  Interview 4 English: 59 - 59 (0) |
| Barriers preferred ecosystem services > Scientists > Lack of monitoring and control | The obstacle is that there has to be active work, on the Ministry's part, the control, and a system that supports this work from a legal point of view,  Interview 3 English: 28 - 28 (0) |
|  | So, the authorities, for example, DINAMA or the military, they don't have any power to do something different. You know, because all these practices go along with a very large territory, nobody could control that. There are not enough means to control or it will be very expensive to control that.  Interview 7 English: 13 - 13 (0)  the farmers cut the fence and nobody reacted.  Interview 7 English: 17 - 17 (0) |
|  | Uruguay has a big problem of water quality, but that is also linked to the health and general health of the watersheds, the basins, so basically what you are having... I mean, we are not alone in the, like, you have, you are quickly approaching a collapse, but in many waterways or aquatic ecosystems; at the same time, you have a weakening of the whole biodiversity of life in the watersheds, erosion, etc. And the agricultural regulations are very poor in Uruguay, very poor, like, there is almost no regulation on the number of fertilizers that you can throw into the land. So basically, there are some regulations with different levels of, how do you say, the compliance would be, I don't know. So, they are regarding soil erosion, okay, soil loss, then you have some regulations which are not very well enforced, but at least you have some, but we have almost no regulation in terms of fertilizer, no regulation or very few regulations in terms of agrochemicals in general. The regulations environmental for the agricultural sector are very old and they are actually very loose  Interview 22 English: 25 - 25 (0)  but and I think that in general you have very low enforcement capacities at the state level, and then you have problems like the whole livestock management issue is complicated to enforce; I would say it's very difficult.  Interview 22 English: 29 - 29 (0) |
|  | Well, there is some controversy at the level of the environmental police; they receive contrary messages, and also, those who do the environmental police, most of them are not really agro-police. So, what would be important is that these measures at the field level are taken by environmental organizations. I have nothing against others, against other professions, but that they know, let's say, how the producers are managed and how it can affect the producer's pocket and the producer's balance sheet, for example, with the buffer zones in the third-order watercourses.  Interview 23 English: 33 - 33 (0) |
| Barriers preferred ecosystem services > Scientists > Lack of monitoring and control > Legislative support | If there is no support of that kind at the legal level with some kind of, I think it does not work completely.  Interview 3 English: 28 - 28 (0) |
| Barriers preferred ecosystem services > Scientists > Lack of monitoring and control > On-the-ground presence | the other thing is that if you can get people to agree on all these things, another thing is to have some sort of physical presence on the ground. Similar to passive restoration, for example, people see it as abandoned land. But the moment you start planting trees, even if it is something symbolic, and you take care of trees, whatever people understand that you are doing something, they are your trees, and the cow is going to eat your trees, you know? At some point, this makes a difference, for example, what happened to us in Paso Severino. In the moment you were present, and you were planting trees, and you made something, well, some neighbours were angry, and they just put their cows there, and they did not care. And they were fighting the state because they took their land away or something. As such, it is very difficult to see something happening, and some active stewardship of the land, versus just, you know, we excluded, and then that's like a barren site or dirty, you know, “sucio” (dirty, red.). It's like, for me, that it's very different. When they see that you are actively managing or having some sort of stewardship over the land, people respect it more. You know? And then, of course, you are there from time to time, so if you come and you say, hey, your cow ate my trees, people will understand, you know, they're grazing on my trees, please remove them, or what.  Interview 22 English: 31 - 31 (0) |
| Barriers preferred ecosystem services > Scientists > Disturbance from exotic vegetation | the control of invasive plant species is also a very important problem.  Interview 3 English: 44 - 44 (0) |
|  | And this is one of the reasons that in many areas, there is no more natural vegetation.  Interview 7 English: 17 - 17 (0)  there are some exotic plants that produce seeds, which are spread by birds, you know, and then they put these seeds into these natural areas, and they start to dominate over the natural species. After some years, the native species disappear from there, and instead of a diversity of species that always grow together, you know, in these natural areas, some trees and shrubs always grow together like a group. They disappear and only these species from Japan or from other places start to grow. And I think it's very bad, but I don't have any numbers or anything scientific about that.  Interview 7 English: 25 - 25 (0)  I never studied this, and the government knows that this is happening, and they try to eradicate these species, but the efforts are very small. It's more for the press than for real (combating exotic species, red.).  Interview 7 English: 25 - 25 (0) |
|  | In many cases, you have mostly grasslands; in some places, you see a lot of shrubs coming in; in other cases, you have some native woodlands or trees, but also some invasive species coming in  Interview 22 English: 21 - 21 (0) |
| Barriers preferred ecosystem services > Scientists > Lack of information | To make this happen, more information on what the river order limit would be, the size of the river, and the size of the buffer zone is needed.  Interview 3 English: 17 - 17 (0) |
|  | one would be having more information, more local examples where people can go and see how other land users have implemented riparian zones, learning from other people that live not from a biologist, but from another person who has successfully been able to incorporate riparian zones into their own area, and that should be a teaching laboratory.  Interview 8 English: 39 - 39 (0) |
|  | Well, I think that there's not enough knowledge about that. Because there's a discussion about whether you know that Uruguay has grasslands as its main kind of biome. So basically, there's a discussion on how efficient forests is versus grasslands, and it depends a lot on, for example, the age of the forest and the state of conservation, and also, for example, the understory vegetation. So, there are a lot of issues, and at some point, I think that it's a mistake to only approach the issue by one variable, like water retention.  Interview 22 English: 15 - 15 (0) |
| Barriers preferred ecosystem services > Scientists > Legislative complexity | One obstacle is the Neptuno project, which diverts attention away from the Santa Lucía river and generates a shift in the interests of the River Basin Commission. It's an issue that needs to be addressed as it has stopped discussions about the Santa Lucía river. The Neptuno project is being studied by another basin, possibly the River Plate Basin Commission or the Maritime Basin, but it seeks to take water from the Río de la Plata, prepare it for consumption, and store it in a reservoir. This diverts attention from the Santa Lucía river and could lead to it being forgotten.  Interview 3 English: 32 - 32 (0) |
|  | I think it's very difficult because the government wants to have good relations with the researcher on one side, and they act like they support us. But on the other hand, they don't want to fight with the farmer because they need their votes in the election. They want to be on good terms with both sides, so it's like a charade. Because the people in political positions want you to be happy there, and then they move to a different scene. It's very difficult. Maybe some people are more conscious and want to do more real stuff, but they are very few.  Interview 7 English: 15 - 15 (0) |
| Barriers preferred ecosystem services > Scientists > Political priorities | I think that they have been a good start in that they have put the importance of the buffer zones on the political agenda, but they have been timid in terms of their definition and the stringency with which they are being monitored and supervised.  Interview 18 English: 15 - 15 (0)  I understand that political policy has to have a beginning, and that the beginning should be the design of the basin. Well, in fact, that is how the Ministry of Environment has gone about it with that intention, not focusing basically on the final phase of the processes and advancing more on mitigation than on planning.  Interview 18 English: 24 - 24 (0)  I am quite critical of the buffer zones as the ultimate solution to the problems. Yes, I understand that they add up and that they are a great contribution and that we have to make progress on that, but that it is a middle or final part of the process and that there is a set of problems that are linked both to agriculture and also to the lack of sanitation in some areas, excessive consumption or the application of water measures in other areas, where the buffer zones can contribute but cannot solve all the problems.  Interview 18 English: 34 - 34 (0) |
|  | I told him to include the lime, but he didn't give me a chance. We ourselves, who were working on the same side, have different views. He didn't want to complicate things because it's one more complication. But now, I give you a prize: your products are emerging from the industries that can replace conventional liming, which is a technique that I have worked on since 1975. Well, then, this is an opportunity, because there are also very strong compaction problems because the soil is always unturned. So now, people, don't wait any longer. In the plans of use, it is going to say that the soil is going to be turned to break the stratification and change the pH of the soil for some crops, right?  Interview 23 English: 27 - 27 (0) |
| Barriers preferred ecosystem services > Scientists > Politicisation of environmental management | Listen, water problems are politicized, it is very difficult, and your opponent, the representative of the opposition in the most important water consumption body in Uruguay, is a history professor. I have nothing against the history professor; please, I have the best will, but he is not a suitable person to represent. Unless he has studied a lot in the last stage, The mayor of Canelones, who also continuously gives his opinion on water and environmental problems, is a history professor. So, he starts to distort visions by political visions, and I do not like that. I do not like it because the water problem is a national problem; it is a problem of state policies; it does not belong to one party or another. When it becomes biased, we lose strength, and, unfortunately, in recent times there has been a tendency towards this, and I do not participate in this; I want to retire.  Interview 23 English: 3 - 3 (0) |
| Barriers preferred ecosystem services > Scientists > Shortcomings current design | I see this as a good start, but it is completely insufficient and has problems. Firstly, in defining the buffer zones, in not considering geomorphological and operational criteria that can solve some of the problems, such as the definition of the boundaries that are defined in certain zones or with different distances. But also, I think they have major difficulties in terms of design and then in the control of whether they are fulfilling what they are designed to do.  Interview 18 English: 15 - 15 (0)  Now, if it is the only measure that is going to develop, it will be insufficient.  Interview 18 English: 34 - 34 (0) |
|  | But I don't agree with that. I think that the assumption is, to give you an example, that Paso Severino has a high part. I did a little work that is available, and I presented it in the commission, where you have a part with a high slope and you have a flat part. In the steep part, it is essential to make a basin area, but in the flat part, it is the other way around; it is the water that advances over the ground and not the runoff over the reservoir. So, the criteria should be different, because there, the one that can even bring phosphorus to the soil is not from above; it is the water itself. It is very common in Uruguay that there are different areas where the phosphorus stays and forms solids that are in the shallows. There are different categories, which I will refer to in a generic way as sodium phosphates, that you should have in the whole area where you are, especially in the lowland area; those are very common. Well, those phosphates are quite impermeable due to their structure, and it is because the sodium is deposited there with the advancing water and generates negative structural conditions. So, the solution would not have to be homogeneous, but as in all things to reach an agreement, it was put aside. Now what happens is that you have the buffer zone in the valley or on the slope, which I don't agree with. You have different soils, different structures, and even the vegetation that you mention—there are areas that have dense vegetation and others that don't. The river is even more complicated when you go to the secondary tributaries because they are watercourses.  Interview 23 English: 7 - 7 (0) |
| Barriers preferred ecosystem services > Scientists > Susceptibility to agricultural activities and changes | The buffer zones are not systems that can function in a stable way. They are natural biological systems that will have certain moments of more optimal and less optimal functioning.  Interview 3 English: 38 - 38 (0)  The river is very susceptible to agricultural activities and seasonal changes, and the nutrients behave quite closely coupled with what is happening in the soil.  Interview 3 English: 40 - 40 (0)  On the other hand, in the reservoirs, there is an accumulation effect. It is an upward trend in the concentration of nutrients that we have seen over the years. They are received, processing, and recycled, and it is accumulating in the sediments or in the water column. There is less renewal.  Interview 3 English: 40 - 40 (0) |
|  | We found that due to the no-tillage planting management practice, which is the practice that is used in most of Uruguay and involves not ploughing the soil, but planting seeds and applying pit fertilizer on the soil top, the concentration of phosphorus (P) in the first centimetre of soil was incredibly high. We measured the differences in losses between areas under agricultural management and those under natural rainfall, and the losses were much higher in agricultural soils.  Interview 7 English: 13 - 13 (0) |
|  | So, I think that the impacts of climate variability change are increasing, as a result of the actions that we are developing in the basin, and are generating a scenario of greater vulnerability.  Interview 18 English: 45 - 45 (0)  No more and no less than for one of the basins where water catchment is the most important situation for a large part of the country's population and which is now evident with this drought we have had, where the Canelón Grande dried up, a severe step that at some point was doubted as to the possibility of providing water by running water. Well, in a scenario of increased forestation, this situation could be much more critical. Here we are not talking about quality, but about water flow. We are talking about quantity, and that gives you the idea that the considerations about the change of climatic inheritance that exist in the Basin function are more a discourse than a real concern.  Interview 18 English: 47 - 47 (0) |
|  | from the productive perspective, you will have problems because of this high intensity precipitation of the drought. But the other thing that's going on is that because of this expansion of crops, because of international prices, and also because of some technological packages, for example, like soybean, you have new frontiers of risk. Like you are expanding your agriculture to regions that are less suited for agriculture, and of course they are more prone to risk, and what happens is because the agricultural sector has such a high leverage and political power, you will be financing that increased risk through public investments or public incentives, so basically what you do is you expand your risk, you reduce your adaptation capacity because you are planting soybeans in huge regions that are not very well suited for agriculture.  Interview 22 English: 37 - 37 (0) |
| Barriers preferred ecosystem services > Scientists > Susceptibility to agricultural activities and changes > Banning industrial agriculture near the watercourse | Well, industrial agriculture can be banned in the whole department. Well, at least it should not advance in these areas. In that sense, not strictly for the industrial agriculture, but for the organisation of activities. I think that the solution has to go that way, to think about planning in the basin and then to deepen or concentrate the efforts of the buffer zones in those places where it is more complicated to advance with this planning. Maybe that's the way I would think about it.  Interview 18 English: 36 - 36 (0) |
| Barriers preferred ecosystem services > Scientists > Susceptibility to agricultural activities and changes > Legislative support | They have to be accompanied by other measures to improve their functioning, such as reducing productive farms, overfertilization, and overloading the soil with chemical products.  Interview 3 English: 38 - 38 (0) |
| Barriers preferred ecosystem services > Scientists > Susceptibility to agricultural activities and changes > Legislative support > Reduce overfertilization | They have to be accompanied by other measures to improve their functioning, such as reducing productive farms, overfertilization  Interview 3 English: 38 - 38 (0) |
| Barriers preferred ecosystem services > Scientists > Susceptibility to agricultural activities and changes > Legislative support > Reduce overloading soils with chemicals | They have to be accompanied by other measures to improve their functioning, such as reducing productive farms, overfertilization, and overloading the soil with chemical products.  Interview 3 English: 38 - 38 (0) |
| Barriers preferred ecosystem services > Scientists > Susceptibility to agricultural activities and changes > Legislative support > Reduce productive farms | They have to be accompanied by other measures to improve their functioning, such as reducing productive farms  Interview 3 English: 38 - 38 (0) |
| Barriers preferred ecosystem services > Scientists > Traditional views producers | It's very difficult to change the farmer practice unless there is a very coercing set of missions or put the police in the field.  Interview 7 English: 13 - 13 (0)  I think one problem is that the people, in general, in these areas don't see that water as a threat. There were few events that have happened with in the Santa Lucia river basin (flood events, rec.). I mean, one in 2011, I think, and another in 2013, and then there was no more problem. So, they never had a real problem with the water. And what they think, what they perceive is that what we are trying to do is complicate their lives, make their work harder and more difficult.  Interview 7 English: 15 - 15 (0)  And also, for example, if a large dairy farm is going to be installed, it could bring more cows to the area. But if the government starts to place constraints, probably the farm will not be installed, and then there will be fewer cows available for the people. And they think that we are a problem for them, not a solution.  Interview 7 English: 15 - 15 (0) |
|  | Culturally it might be something that people are not used to doing, especially with the drought that we're having now access to water is really, really important.  Interview 8 English: 17 - 17 (0)  I mean, when you have cattle, let's say that you have a riparian zone, I mean, if you had people sometimes referred to as like “mugre” (muck; grease; filth, red.), you know, so I mean, whenever you have a riparian zone, especially one that's recovering that might not have like nice tall trees and an open understory, you can have all sorts of problems. I suppose you can have snakes. I suppose that if it's an area where you do have cattle ranching with high density forest, I know that people have lost cattle in the forest. It can be more difficult to manage livestock if you have closed vegetation.  Interview 8 English: 37 - 37 (0) |
|  | The other day we went to visit a horticulturalist, and he has a house there, and he puts glyphosate on the apple trees.  Interviewee: Well, this is an issue that transcends industrial agriculture and that basically takes place in almost all the activities in the area. The big difference are the volumes and the scale with which these products are used, and the scale of production, and that determines that. In general, industrial agriculture is a bigger problem for the environment than family agriculture. Not to mention the fact that the toxicity of these products goes beyond the activity itself. In fact, there are health problems in almost all of Uruguay due to the use of these products. In this sense, the municipality of Canelones has also been one of the sectors at governmental level that has had more weight in the promotion of other types of practices.  Interview 18 English: 41 - 42 (0) |
|  | Well, we had this discussion at some point, and I, look, yes and no, because in a way, for example, to have perennial pastures or some sort of, do you mean only forested uses or also other types of uses? There are also other types of uses because, like you said, it's very difficult. Because there was a discussion, for example, instead of having crops, just put perennial pastures, and then you, you know, reduce the amount of work. And for me, the problem here is we are discussing over peanuts, because honestly, these are very narrow streams, like we are talking about a few meters. And in Uruguay, land is not so small, like usually you have hundreds of hectares, sometimes more, or, you know, so I am a little bit sceptical in terms of saying “we take the productive uses like right next to the river”. For me, it doesn't make sense. You first have to have some sort of natural vegetation, which could imply natural pastures in some places, and another will be more forests, shrublands, or wetland vegetation. And for me, there's no question about that. But then, of course, you can have secondary buffers or buffers of buffers, which could be fewer intensive uses of the land. But I am against, and there was a discussion, for example, of doing agroforestry in a field just next to the river. And I'm like, I mean, really, we are discussing over peanuts, you know, and if you're talking about tens of meters, why would you be using complex solutions? Well, I could understand if you want to do like 30-meter buffers and then you do a kilometre; that's fine. But if you're just talking about tens of meters, then, in my opinion, you should just go for a basically ecological ecosystem service.  Interview 22 English: 19 - 19 (0)  you need some sort of engage with the landowners’ program to engage with the landowners, but also some sort of support probably from at least some of them; there already are some actions, I am not fully aware of the latest development of Tanques australianos (water storage tanks, red.) or other ways to provide water to livestock, and I do not know how extended their use is, but it is always easier to let the cattle go to the river, which is pretty bad.  Interview 22 English: 29 - 29 (0) |
| Barriers preferred ecosystem services > Scientists > Weak institutional structures for environmental protection | Uruguay has a big problem of water quality, but that is also linked to the health and general health of the watersheds, the basins, so basically what you are having... I mean, we are not alone in the, like, you have, you are quickly approaching a collapse, but in many waterways or aquatic ecosystems; at the same time, you have a weakening of the whole biodiversity of life in the watersheds, erosion, etc. And the agricultural regulations are very poor in Uruguay, very poor, like, there is almost no regulation on the number of fertilizers that you can throw into the land. So basically, there are some regulations with different levels of, how do you say, the compliance would be, I don't know. So, they are regarding soil erosion, okay, soil loss, then you have some regulations which are not very well enforced, but at least you have some, but we have almost no regulation in terms of fertilizer, no regulation or very few regulations in terms of agrochemicals in general. The regulations environmental for the agricultural sector are very old and they are actually very loose  Interview 22 English: 25 - 25 (0) |
| Barriers preferred ecosystem services > Scientists > Willingness of producers to implement | It’s a bit silly, but it's like taking antibiotics. One thing hurts you, let's say it harms you and you take antibiotics. It does nothing to you if you take too little, it doesn't achieve the objective. And, well, the other thing, yes, if it works, it might hurt you a little bit, but it does achieve the objective, right? The objective is to control bacteria. It can only be effective when taken properly. In this case, what we were afraid of was that the buffer zones would be left with half of them non-compliance, and the other half with compliance (of landholders, red.). So, it's really the incomplete implementation that would have been reflected by inconsistent and unencouraging results. This has led to the pressures that led to the abandonment of the buffers because they (landholders, red.) considered them to be useless, and they were losing land to cultivate. So, by not really moving towards more complete and controlled areas, we were going to be left at a turning point and we're going to go backward.  Interview 3 English: 24 - 24 (0)  It's like a chain reaction, isn't it? The one who didn't perform, who never complied, suffered no harm. Then, the next person sees this and decides not to do anything, assuming that they don't have to, and so begins the chain of non-compliance. It's an issue that's often neglected and overlooked, and it's something that we as producers must work hard on to combat, often by word of mouth.  Interview 3 English: 30 - 30 (0) |
|  | Farmers didn't want to change their practices because it was cheaper and more convenient for them. The other recommendation I have is time-consuming and much more expensive. So, they never applied anything, they just kept doing what they were doing.  Interview 7 English: 13 - 13 (0) |
|  | buffer zones are acting as or are interpreted as a nuisance for the development of agriculture. And well, I think we are at the time to make that leap and to understand what other ecosystem services they have for some linked to mental quality and others that may have ecosystem services, either thinking in terms of ecosystem services of provision, support and even cultural.  Interview 18 English: 20 - 20 (0) |
|  | What happens is that order 3 is made up of small producers, even the watercourses, and sometimes they are not even permanent. They are watercourses that are cut, so those small producers—if you take away the 50 meters, which is the length of their field—you take away a lot.  Interview 23 English: 7 - 7 (0)  You are going to make many producers have to sell their land because if you take away, I don't know, 50 meters in front of the watercourse, in other words, have 2 hectares that are not going to produce to make more money, It's 25 thousand square metres with the 50 wide or 500 long. Well, it's 2 and a half hectares, and for a producer who has 10, 12, or 15 hectares, that's a lot.  Interview 23 English: 33 - 33 (0)  Secondly, once the decisions have been taken, whether they are good, bad, or regular, we have to try to implement them. And in order to implement them, the producer, or the majority of producers, do not distract their resources and time on things that they do not consider important, that is, they do not see their direct importance. So, you have to look for incentives and ways to compensate them for what they are supposed to lose. Because if he doesn't, he doesn't do it. So, you have to take advantage of the fact that when you give loans or make a kind of policy to consider these areas, you are going to make a long-term investment that suddenly is not going to produce what you expected, because if you don't allow them to fertilize, you don't leave anything. The other day, I laughed a little bit when he said that he was going to make protected areas at the will of the producers. I never saw that.  Interview 23 English: 33 - 33 (0) |
| Barriers preferred ecosystem services > Scientists > Willingness of producers to implement > Provide drinking water alternatives livestock | in my opinion, you need to have some sort of presence on the ground. So, there are two different things: right? One is, for example, if you want to achieve some restoration of forest, or buffer zones, restoration, and so you want to regulate, for example, livestock, rights? I think it is fairly easy to do it regarding crop lands, right? Because satellites can be monitored, it is easier, but in that regard, the only thing you need is some sort of political will to control and some control capacity of the state, right. But you can do it. With livestock, in my opinion, it is more difficult, and you could do some sort of support for others, for example, to provide drinking water to livestock outside of these areas, so that livestock do not have to get in, but then you need to either build exclosures, which are expensive, or provide water outside, which is expensive.  Interview 22 English: 31 - 31 (0) |
| Barriers preferred ecosystem services > Vida Silvestre > Conflicting visions between stakeholders | And what I would say is that ecosystem services, it's a concept that it's meaningful as long as it also recognizes that different stakeholders obtain different things from nature. So, it also depends on the views, needs, and values of the stakeholders.  Interview 20 English: 13 - 13 (0)  you change the kind of function you want to optimize rather than trying to maximize individual gains with regards to one dimension of development, you go into a multiple objective goal of integrating interests from different stakeholders and trying to maximize gain in a range of dimension of development. So, it's a much more complicated scenario on which you have to take decisions, but the whole idea of going from individual isolated management of land into an integrated management of land that takes into account the different kinds of interests and needs.  Interview 20 English: 21 - 21 (0)  The departments in terms of administrative regions of that are trying to maximize short-term gains but also long-term gains. So, what we do is by creating a protected area where we are forcing ourselves to think in this multidimensional way and that's the challenge. Because protected areas were promoted with one specific goal in mind but that promotion came after a more general decision of promoting nature conservancy in the protected area and then ecosystem restoration and another intervention on the landscape to promote resilience in the landscape. And so, it's the question of the buffer zones in the camps, there are some decisions and some visions that were already set there when the government added another layer and said we need to restore buffer zones to maintain water quality, but there were already discussions on what interventions we need to do in that landscape to enable the production but also the health of the landscape. So, for me, it's very difficult to just think and answer in terms of the specific buffer zones, 200 meters or 100 meters around the river.  Interview 20 English: 23 - 23 (0)  I think the social process is much more complicated than the physical one. I will give you a specific answer regarding the buffer zone and then a more general one regarding the area in general, we don't have good experiences in terms of recovering ecosystems, so we've been trying to do a range of initiatives without proper research and proper testing and long-term funding so the easiest part should be restoring the ecosystem. And I'm not saying that it is easy but I think it is the easiest, we are not doing that right because we don't have experience and we haven't taken the decision of investing in research and experiences that can enable us to do this properly, but that's one issue.  Interview 20 English: 25 - 25 (0)  That's one of the advantages of having protected areas in Santa Lucia, and the second one is that protected areas are managed adaptively, so they are developed to handle this kind of problem. The problem is that most of the stakeholders involved do not see this in that way. The whole idea of the protected area has not been put forward appropriately. It's more like it's seen more like initiatives to protect certain pieces of native ecosystems and leading to more than that, but the whole idea of having a protected area from my point of view is to put together in that territory the tools that have been developed elsewhere to handle wicked problems through an adaptive management process. But I think no one is seeing this and that's I think, I don't know if it's a problem, it's maybe putting this forward and seeing this clearly might help to evolve to this area in a different direction.  Interview 20 English: 31 - 31 (0) |
| Barriers preferred ecosystem services > Vida Silvestre > Complex public institutionality | And we have institutions that are more used to a traditional management approach, so you need to also change the way the institutions take decisions and monitor the decisions and modify the decisions based on the evidence they generate. That's one issue from a more institutional perspective. But then again, a range of stakeholders seeing the problem from different perspectives expecting different things from that landscape with different, how would you say, competence in terms of being the ones that have the right to take the decision so it's just at a smaller scale; the challenge of managing a country or a rainy piece of land when you integrate several persons and several stakeholders so I wouldn't be able to say what's the most difficult part of that. If you try to make this simple and think okay this is a sociological system, the social component of the sociological system is a mess, we don't know how to manage that properly, we don't know how to agree, how to build consensus and that's where the challenge is. We don't have experience in ecosystem management but we can gain that and there's a lot of experience elsewhere we can put to work here. But the social subsystem is a complicated one, a very complicated one.  Interview 20 English: 25 - 25 (0) |
| Barriers preferred ecosystem services > Vida Silvestre > Political short-term vision | The departments in terms of administrative regions of that are trying to maximize short-term gains but also long-term gains. So, what we do is by creating a protected area where we are forcing ourselves to think in this multidimensional way and that's the challenge. Because protected areas were promoted with one specific goal in mind but that promotion came after a more general decision of promoting nature conservancy in the protected area and then ecosystem restoration and another intervention on the landscape to promote resilience in the landscape. And so, it's the question of the buffer zones in the camps, there are some decisions and some visions that were already set there when the government added another layer and said we need to restore buffer zones to maintain water quality, but there were already discussions on what interventions we need to do in that landscape to enable the production but also the health of the landscape. So, for me, it's very difficult to just think and answer in terms of the specific buffer zones, 200 meters or 100 meters around the river.  Interview 20 English: 23 - 23 (0) |
| Barriers preferred ecosystem services > Vida Silvestre > Wicked problem structure | Then the challenge of an integrated management of a region of an area of the size of Santa Lucia; it's a typical wicked problem that needs to integrate views, so it has all the challenges of a wicked problem and you know how a wicked problem has to be managed. And that's one of the advantages of the protected area approach, protected areas use adaptive management as a method of the management approach, but in order to do that you need amongst other things, institutions that can take decisions and adaptively change the decisions.  Interview 20 English: 25 - 25 (0)  The uncertainties that are present in the system and hence the challenge is in that part of the drawing in the social aspect and that you won't get the necessary consensus, and that you won't solve this problem that you would just manage it in a way that harms you the least possibly. You navigate a permanent problem, it's not that you solve a problem and move away from that and that there is not one single solution. What I mean is that for me the problem is that we are not seeing the problem or approaching the problem properly, because we are thinking of the problem as a more trained problem that has technical solutions and we are in a different arena, in a different scenario.  Interview 20 English: 27 - 27 (0) |
| Barriers preferred ecosystem services > Vida Silvestre > Wicked problem structure > Adaptive management focus | But I think if I have something specific to say in this conversation is that we need to understand this problem as a wicked problem and wicked problems have very specific ways of being approached, with adaptive management being a key tool. But adaptive management it's not widely incorporated into the way we manage the landscape and countries. It's a way of managing, of taking decisions that understand that there is uncertainty, that understands there are values in conflict, and that then it plans, it acts, it learns, it changes and plans again and acts again. And that approach involves not only the actions focused on the biophysical part of the system but also in the social part of the system, so we need to manage this social complexity also in an adaptive way and understand that different stakeholders have different values that manage differently uncertainties that are perceived differently.  Interview 20 English: 27 - 27 (0) |
| Opportunities preferred ecosystem services > CAF > Adaptive capacity farmers | Our farmers are quite intelligent—more intelligent than people think, really—and they quickly understand and improve things.  Interview 24 English: 11 - 11 (0)  This is my idea, but for me, it's very important to try to connect the people that are in production, have them, and make control over them what is important in this job. For me, culture is the most important thing in everything you try to introduce. You've made a law, but people try to escape from it;  Interview 24 English: 15 - 15 (0)  this issue is important for the country, but also that they must make the actions, not the law. The actions—this is a big difference between the law and the action, and you need to introduce actions and help and advisors that help people improve and resolve this problem. In the agronomic engineering area, we have a lot of people who know a lot about how to reduce the flow of water on the slope. We have a lot of people who probably never work on this problem, especially in this area, but it's a good opportunity to introduce them and to help the farmers understand what happened with the forces they put inside. That's the real thing; we need some more work on that. We need more work on that. We need to regulate how the water will flow; it always will flow to the river, but we need to control this flow to reduce the space, to really reduce the movement of the land, and to reduce the contamination of the river. That's the idea; how to do that is the important thing to teach the farmers.  Interview 24 English: 17 - 17 (0) |
| Opportunities preferred ecosystem services > CEUTA > Appropriation of buffer zone to increase involvement producer | the appropriation of the system. With the appropriation of the buffer zone, the farmer is going to be much more involved. They could become more in favour of the implementation of the buffer zones.  Interview 2 English: 20 - 20 (0) |
| Opportunities preferred ecosystem services > CEUTA > Benefits of increased biodiversity > Enhancement agricultural ecosystem services | but it also works for the improvement of the performance of the production of the agricultural systems. An area with enriched biodiversity with pollen production will enhance certain ecosystem services of agricultural and livestock systems.  Interview 2 English: 20 - 20 (0) |
| Opportunities preferred ecosystem services > CEUTA > Benefits of increased biodiversity > Increased root volume | As I said before, this enriched biodiversity has a larger root volume capacity. And it has more biological activity, and it is going to improve the ecological status of the area.  Interview 2 English: 20 - 20 (0) |
| Opportunities preferred ecosystem services > CEUTA > Benefits of increased biodiversity > Increased skills producers | And then, the enrichment of other ecosystem ecosystem services; the producers would have to do training (leading to more skilled producers, red.)  Interview 2 English: 20 - 20 (0) |
| Opportunities preferred ecosystem services > CEUTA > Implementation based on the independence producers | My advice would be to have an initial phase of 3 or 4 years with agroecological soil management with this approach. I believe that this is the beginning of a virtuous circle of the production of more biomass, generating better animal welfare, and producing better results, better productivity, and better yields.  Interview 2 English: 11 - 11 (0) |
| Opportunities preferred ecosystem services > CNFR > Development of a National Agroecology Plan | For example, today, I think you should know that it is a great opportunity, not only for the Santa Lucia river basin but for the whole country, that we are working on the implementation of a national agroecology plan. This national agroecology plan, which is being influenced by public bodies such as producers' institutions or technical institutions, is beginning to generate agreements on the practices that need to be promoted. And these practices that they are trying to promote, especially for production systems, are already validated and it is already known that they give better results.  Interview 5 English: 26 - 26 (0) |
| Opportunities preferred ecosystem services > CNFR > Expansion pilot studies on buffer zones | So, it is no longer just a matter of starting in pilot areas or with pilot experiences, but that at some point, to the extent that these results work, we have to scale them up.  Interview 5 English: 36 - 36 (0)  But the processes, let's say, of implementation of good practices on the farms go hand in hand with long periods, where the farmer can try them out, where the farmer can see the results, where they can be encouraged, where they have someone to accompany them in making decisions and where they have someone to measure the results. And say to me, look, I used to use such and such a thing and I got so much product. For instance, now with the change we are achieving 20% more production with less spending, which is often what ends up happening. They (producers, red.) save resources on inputs and produce the same or more. This is what many of the experiences that have been developed say.  Interview 5 English: 36 - 36 (0) |
| Opportunities preferred ecosystem services > CNFR > Potential for climate change mitigation | So, I think that I don't know if this is what is happening in the area, but it should be seen as, a threat or a weakness, and as a future opportunity, to start taking mitigation measures, right? And it surely has to do with, using more efficient risk systems, and better pasture management, in the case of people who work with livestock, to begin to take a little more care of the soils so that when the dry season comes, they have more capacity to retain water. And this goes hand in hand with certain practices, which I believe always provide opportunities for change.  Interview 5 English: 34 - 34 (0) |
| Opportunities preferred ecosystem services > CNFR > Potential integrated environmental management | I think that is perhaps the most important thing, that people learn how to make good use of these spaces and integrate them as such, as part of the productive system. Not as something that is on the outside because it is better.  Interview 5 English: 24 - 24 (0) |
| Opportunities preferred ecosystem services > CNFR > Promotion sustainable agriculture | many of these management practices within the buffer area and outside the most productive areas, have also been studied to have been beneficial. Not only for the environment and biodiversity but also for the producer family.  Interview 5 English: 26 - 26 (0)  We know that substituting chemical inputs with biological inputs avoids water and soil pollution.  Interview 5 English: 36 - 36 (0) |
| Opportunities preferred ecosystem services > DINAGUA > Integration National Land Use Planning Programme | The land-use plans are the right tool to control this measure because when you do the control of crop rotation, you control the compliance of buffer zones.  Interview 6 English: 12 - 12 (0)  The issue of management should be studied and regulated.  Interview 6 English: 22 - 22 (0) |
| Opportunities preferred ecosystem services > DINAGUA > Potential for paradigm shift private to public culture | That's what I want to tell you, that the environmental protection law has important powers to limit the right of property in the general interest, which was the protection of the water quality of the St Lucia River. I don't know what your opinion is, but well, I understand that in our legal system, let's say, there is a paradigm shift with the last battery of laws that have been passed, where the general interest begins to impose itself over private law. I believe that in the first century that the civil code was in force in our country, it was particularly privatist. And the latest regulations, the ones that have been passed in the last 20 years, have made a change to the property rights and makes it more limited.  Interview 6 English: 10 - 10 (0)  The right to property for the general interest is above private interest, and it is enforced as the institutional culture of ours.  Interview 6 English: 18 - 18 (0) |
| Opportunities preferred ecosystem services > DINAGUA > Willingness of producers to implement | the producer is the first one who wants to take care of the soil and carry out sustainable management. They are the first interested party because they want their soil to have the properties to pass on to their children and grandchildren. Producers are very interested in incorporating new practices and changing old paradigms of property rights of use, enjoyment, and abuse of the land. They are no longer in the business of using and abusing the land but rather want to adopt sustainable management practices.  Interview 6 English: 18 - 18 (0)  Producers and the administration are seeing the need to use this protection because it also goes hand in hand with the law of territorial planning and the regulation of land use.  Interview 6 English: 22 - 22 (0)  There is a technical change in the technical paradigm with integrated management and integrated visions of the approach to the territory. In parallel, there is a change in legislation. The law of territorial planning and the water policy law appears. And then at the individual level, at the producer level, the producer is realizing that it is reality, you don't plant leaves to the edge of the watercourse, and he loses a few hectares, but he gains in water quality and in quality of other things, right? There are mentions of the regulation of the quantity of water flow.  Interview 6 English: 22 - 22 (0) |
| Opportunities preferred ecosystem services > DINAMA > Collaboration of different stakeholders | The Ministry of Livestock should also support this effort. The Ministry of Livestock has the best relationship with agronomy producers and has more contact with them through development roundtables. They work together a lot and have crucial contact with the producers. Therefore, it's important to achieve an alliance between the Ministry of Environment and the Ministry of Livestock.  Interview 12 English: 27 - 27 (0) |
| Opportunities preferred ecosystem services > DINAMA > Generation of knowledge for all stakeholders | generating knowledge and providing education, awareness, and appropriation of the measures and knowledge is crucial so that whoever applies them has a full understanding of why they are doing so. I believe that's the most important thing.  Interview 12 English: 27 - 27 (0)  Additionally, gaining support from all parties involved is crucial. Of course, as environmental care is sometimes opposed to production, achieving a balance is necessary. One crucial aspect to achieving this balance is communication, dissemination, and cultural change.  Interview 12 English: 27 - 27 (0)  Additionally, communication and environmental education are critical, especially as we are entering a new era of Santa Lucia river action plan. There are small projects in different areas. For instance, in Media 5, which is located in the Southside lagoon, there was work done with producers and tremendous awareness was achieved. However, these are just small impulses in small groups. Other projects have achieved successful implementation, and it was implemented before Media 8. It has more history and is smaller, which changes the perception of the type of producer. In the Santa Lucía basin, there are many milk producers, which is another change. The Rocha lagoon (where these projects were located, red.), also known as the Swan lagoon, is the most progressive. The Ministry of Environment has taken that forward. There is also someone in the territory who works for the Ministry of Environment and is known to be in the Rocha lagoon. One colleague has been working for many years in the whole of Maldonado, for about 10 years in the ministry. She has managed to establish a good link with the livestock people and they work together. However, the Rocha lagoon is smaller, and there are more livestock producers and a bit of culture that sets it apart from other areas. Therefore, the conflict is probably not so big, as it is a public-private conflict.  Interview 12 English: 27 - 27 (0) |
| Opportunities preferred ecosystem services > DINAMA > Implementation lower-order stream buffer zones | the main benefit is that it would reduce the amount of nutrients that enter the water from the soil.  Interview 10 English: 28 - 28 (0)  By extending the buffer to smaller watercourses, we estimate that we could retain up to 40% of the nutrients.  Interview 10 English: 28 - 28 (0) |
| Opportunities preferred ecosystem services > DINAMA > Integrated environmental management | However, it's important to note that buffering alone won't solve the problem. We need to implement clear land use measures and practices to address the source of the problem. Buffers are a temporary solution, as they tend to become clogged over time and lose their effectiveness as filters. Therefore, we must address the root cause, which is land use.  Interview 10 English: 28 - 28 (0) |
| Opportunities preferred ecosystem services > DINAMA > Integrated environmental management > Buffer surveys | we should start with a survey of these buffer zones. This will enable consideration of different management approaches, such as animal grazing, authorizations for crops that extract greater amounts of nutrients, or vegetative felling.  Interview 12 English: 21 - 21 (0) |
| Opportunities preferred ecosystem services > DINAMA > Implementation complementary management tools | there is also this elegant algorithm that works half alone, and that is what the ministry is working on.  Interview 10 English: 45 - 45 (0) |
| Opportunities preferred ecosystem services > DINAMA > Implementation complementary management tools > Division environmental information systems | there is a division of environmental information systems, which is like a geographic information system.  Interview 12 English: 52 - 52 (0) |
| Opportunities preferred ecosystem services > DINAMA > Implementation complementary management tools > Participatory data collection | What we could do, I imagine, is ask the producer to provide data on soil bulk density and phosphorous in the soil. If the bulk density decreases over time, it indicates excessive compaction, and obviously the loads are not being respected. If phosphorus levels in the soil increase, it doesn't necessarily have to be all from grazing, but management needs to be checked. That's where it comes into play.  Interview 12 English: 45 - 45 (0) |
| Opportunities preferred ecosystem services > DINOT > Potential use of economic incentives | in the framework of this programme, we say that, on the one hand, the tools, the plans are the ones that are going to limit and promote the activities in that territory, orienting the productive activities. And this is where the incentives appear, the promotion of incentives by the ministry of economy, of the different economic incentives that always exist.  Interview 21 English: 21 - 21 (0) |
| Opportunities preferred ecosystem services > DINOT > Integration National Land Use Planning Programme | So, my vision is that national programmes are necessary to define strategic orientations based on concrete actions, but then implemented in a coherent, regional approach through the responsibilities of each national body and through the responsibilities of the ministries. This is why we liked the idea of working within the framework of national strategies and national spatial planning programmes. This is where the intention of the national programme comes from. So, I think that for the traditional regulation of land use, departmental instruments are much more effective, but there are national responsibilities that the departmental instrument mustn't contradict, because otherwise it would be ineffective. So, we need a cross-departmental instrument that at least defines a strategic working basis that prevents contradictions. This is where regional spatial planning instruments or national spatial planning programmes come into play.  Interview 21 English: 9 - 9 (0)  The Planning Act creates the idea of territorial policing, which gives authorities the power to set up territorial policing precisely for planning instruments. It's clearly a great tool that is little used, but it seems to be a very necessary tool that needs to be complemented, as I said today, by the control of the use and management plans, which is the first time in Uruguay.  Interview 21 English: 11 - 11 (0)  I have in Flores, when the idea of a protected forest was implemented, that this would be something in between, between a protected area and what you want to do. Today there is a conflict because people want to use their land freely, right? In other words, it's something that needs to be ingrained in the culture of the producers, right? They thought of some kind of instrument to bring the producers more down to earth, so that there is a strong change, let us say. And the basic tool is the use and management plans, right? That is, for all lands that are identified as ecologically vulnerable, there needs to be a use and management plan, similar to what we have already done, beyond the size of the land. We have been working with this idea of a use and management plan and trying to align the areas that are impacted by this plan so that they still serve a productive purpose. It would still have a productive purpose, but within the context of a use and management plan for that land with its own activities.  Interview 21 English: 21 - 21 (0) |
| Opportunities preferred ecosystem services > DINOT > Integration National Land Use Planning Programme > Generating definitions and clarity | You are going to tell me the whole legal chapter of this, because the expulsions of ordinance always start with the Parliament, and this is unconstitutional because, of course, it touches interests, doesn't it? Here, the line of reasoning is the following: the planning law allows that, through the instruments of territorial planning—this is not an isolated measure; this is an instrument—one can orient and limit the use of the land of the territory. So, it seems to me that what is potentially unconstitutional is to prevent any activity from being carried out on rural land, i.e., to limit any type of activity on that rural or urban land. But to guide the activities with restrictions that are in the sense of what is in the construction with respect to the care of the environment and the care of water or resources, especially the water that will later be made drinkable, Those restrictions and limitations on land use and on the use of private property are in the construction law and the law on land use planning. So, this is the framework within which land use planning works, really generating definitions of land use and compatibilities of some activities. But obviously any planning instrument also has to think that these limitations have to be viable for productive activity, which is why the whole issue of incentive programs is promoted. To guide them and give them viability.  Interview 21 English: 25 - 25 (0) |
| Opportunities preferred ecosystem services > DINOT > Integration National Land Use Planning Programme > Integrated local management buffer zones | So, my vision is that national programmes are necessary to define strategic orientations based on concrete actions, but then implemented in a coherent, regional approach through the responsibilities of each national body and through the responsibilities of the ministries. This is why we liked the idea of working within the framework of national strategies and national spatial planning programmes. This is where the intention of the national programme comes from. So, I think that for the traditional regulation of land use, departmental instruments are much more effective, but there are national responsibilities that the departmental instrument mustn't contradict, because otherwise it would be ineffective. So, we need a cross-departmental instrument that at least defines a strategic working basis that prevents contradictions. This is where regional spatial planning instruments or national spatial planning programmes come into play.  Interview 21 English: 9 - 9 (0)  Current activities in the preferential use area. Well, which of these can be compatible. And for that, criteria for management and coordination for regulatory management are needed.  Interview 21 English: 21 - 21 (0)  how it is overcome, is that it becomes a public policy that, regardless of how the parties alternate in power, is a continuous political item, and that it evolves. And the other seems to me that there is a construction of an agenda that appears the education and social organizations. The priority of the topics, the dissemination of the problem, the understanding of citizenship, of the ecological state, of understanding of how it affects our territory, and that the drinking water is not endless. But that there is a whole system that ends.  Interview 21 English: 38 - 38 (0)  In the end, the problem is that understanding all these lines of causes and consequences is not necessarily direct. But yes, if one can connect them, that is where the need to have an integrated perspective appears. In this case, at the level of the river basin, or at the socio-territorial level. If you want to say it in some way, the need is precisely for learning, in the deepest sense, to incorporate people to defend and fight fairly to protect water quality, which is not just investing in carbon for the water treatment plant, it is much more than that.  Interview 21 English: 38 - 38 (0) |
| Opportunities preferred ecosystem services > INIA > Benefits of additional services of biological diversity | As an agronomist, I believe it's important because maintaining diversity helps prevent colonization by invasive species and promotes the evolution of ecosystems. It may also provide other services that we may not have considered.  Interview 15 English: 31 - 31 (0)  The issue is that when they cut it, of each tree hundreds come out, and it becomes unmanageable. The solution we are doing here in an area of Espinillo, not cut, just the opposite, what they do manage under the Espinillos, that very productive fields are generated. Look, just next week, I have a meeting for this issue. For three reasons, it is a leguminous plant that has a lax shade canopy that allows light to pass through. So, the grass grows underneath without problems, but it protects against winter frosts and desiccation in summer. So, you always look under the Espinillos, the grass is better than elsewhere.  Interview 15 English: 47 - 47 (0) |
| Opportunities preferred ecosystem services > INIA > Opportunities for sustainable grazing | Grazing is one tool that can help control invasive species, but it can also negatively affect riparian zones. Therefore, it's important to consider the specific situation and take appropriate measures to maintain biodiversity.  Interview 15 English: 31 - 31 (0)  I think zero grazing is not good from the perspective of vegetation because it evolves with grazing. If there is zero grazing, a lot of dry matter accumulates, which sometimes increases the risk of fire, and it generates conditions of competition that favour some species over others. So, biomass removal by grazing should be there. As I said, there is no fauna that has been able to do it or is in a minimal expression, the alternative is that it should not be freely accessible and that it should be managed. However, we don't know enough to say which is the best time. There are empirical experiences, but we are trying to document and measure them.  Interview 15 English: 39 - 39 (0) |
| Opportunities preferred ecosystem services > INIA > Potential use ecosystem integrity index | By using this scale, we could measure progress economically from one to two, while also being able to determine whether environmental progress had improved or declined by one point. In fact, the project showed improvements in productivity and, in several cases, stability in the ecosystem and even some cases of improvement.  Interview 15 English: 17 - 17 (0)  Some people may wonder what this is since they don't even know what to look at and what could be considered right or wrong. This doesn't mean that we might not have to revise it in the future and say, "Well, let's see, this thing that we evaluated, it has to be evaluated differently, or it has more or less weight." But now, at least they know that there is an environmental dimension and that it is not just land. Through the index, it has been incorporated as an evaluation practice.  Interview 15 English: 19 - 19 (0) |
| Opportunities preferred ecosystem services > MGAP > Compliance control through citizen participation | One of the ways to control this is through complaints from citizens, who can report these violations, and authorities can take necessary action. There are other mechanisms, such as the obligation to present a use plan at the directorate of natural resources, which includes interpretation of satellite images to monitor land use plans.  Interview 17 English: 46 - 46 (0) |
| Opportunities preferred ecosystem services > MGAP > Current tax benefits native forests | You have to understand that the native forest is protected by law, and the producer, in addition to the law, if he registers that forest, he can have tax benefits; generation of contribution, primary tax, etcetera. So, in a way, he is not paying taxes for that area, and you can make a sustainable use of the forest, even get a logging permit.  Interview 17 English: 27 - 27 (0) |
| Opportunities preferred ecosystem services > MGAP > Implementation complementary management tools | In other words, there have to be other tools to implement a measure like this.  Interview 17 English: 25 - 25 (0) |
|  | And for example, if your focus may be on the Santa Lucia Basin adjust the monitoring and try to let people do things well, not in the whole country. Soil or farm planning, with good practices, making land use plans, that for example, adjust the fertilization of the crops in soil analysis. All this good management will be improving all the buffer zones, they are in most of the laws, but it also has to make sense regarding fiscalisation. Maybe in these basins that are as more vulnerable or more important, but everything is land use; proper land use, and management, would help a lot.  Interview 19 English: 23 - 23 (0)  And then when all the industries and all the, what is point sources, but it's not so difficult because you know exactly the location of the contamination. For example, if you're talking about a diffuse source, the contamination is more difficult to avoid, no?  Interview 19 English: 23 - 23 (0) |
| Opportunities preferred ecosystem services > MGAP > Monitoring of pesticide use | La Dirección de La Granja has a lot of policy about also using pesticides and they have all the little farms that have vegetables and fruit with identification and geolocalisation and they have a map with all the farms are, for example, to protect the applications of pesticide to the fields. In schools, they're all georeferenced to have control of the applications of these products, pesticides in these vegetable farms and fruit farms that are very intensive.  Interview 19 English: 11 - 11 (0) |
| Opportunities preferred ecosystem services > MGAP > Potential for studies on sustainable intermittent grazing | There has to be a little study on which one is the best way to do it, to do the grazing, maybe three hours a day, maybe some days. There has to be some plan of grazing for the farmers, because like we say here, "la cura es peor que la enfermedad" (the cure is worse than the disease, red.), figuratively speaking.  Interview 19 English: 15 - 15 (0) |
| Opportunities preferred ecosystem services > MGAP > Update current warning system | However, implementing a warning at the system level is simple, even with the use plan issue. We are currently updating the system, and we have added all the dairy and irrigation information to the use plan management system. Although it is not yet in production, the advantage is that all information regarding the fields or any use made at the level of irrigation, dairy, or agriculture will be available in the system. The system does not have to limit the use of a field when it is intercepted by a buffer zone.  Interview 17 English: 54 - 54 (0) |
| Opportunities preferred ecosystem services > Producers > Adaptive capacity producers | When we were kids, we heard the story of the three little pigs who built houses out of brick, wood, and straw. I always tried to make things out of straw. For example, I used the shades that dairy farmers make for their cattle, but they are usually handmade and become unstable after two years. I also needed electricity, so I made a shade with solar panels, but I couldn't afford it. Instead, I planted a few solar panels in the field and used it to make shade for the cows. Now, theoretically, I am generating electricity while the cows have shade. The electrical engineer from other countries who designed the system calculated that I would be at a break-even point, meaning that I wouldn't have to pay for more energy than I generate.  Interview 13 English: 34 - 34 (0)  On another note, have you heard of a product called EM (efficient micro-organisms, red.)? It is said to be effective in managing odours and composition of matter, and I have started using it. This ties in with the concept of microbiology and agroecology. What are your thoughts on EM? They are Japanese cultures that can be used in agriculture. I'm interested in trying new approaches to farming that are more sustainable and agroecological. There is a group of dairy farmers who are part of this movement, and they use EM a lot. I got connected with them and started using it too. The dairy farming industry is often focused on producing more at the expense of the environment and the farmer's quality of life. But there are alternatives that are more circular and sustainable. I want to learn more about this approach and make changes on my property.  Interview 13 English: 36 - 36 (0)  But I want to educate myself and work with the ministries to make reforms that promote sustainability and diversity. Regarding the buffer zones, I'm interested in learning more about their physical aspects and ecosystem services. I also want to know more about the preferred attributes for farming and how they can be incorporated into my farm.  Interview 13 English: 36 - 36 (0) |
| Opportunities preferred ecosystem services > Producers > Ecological resilience buffer zones | Yes, in general, I always saw them as greener than all the others, which don't cover as much. But today, everything that's dry, I don't know if it's going to die. All the invasive plants have dried up. It's super powerful, isn't it? Of course, that's wonderful, if you control it. In two years, there could be areas where the animal no longer enters. I mean, I emphasize this because I know that there are people who are of the opinion that this is not the case, that if you want to conserve, you don't have to manage. For me, that is a big mistake.  Interview 4 English: 35 - 35 (0) |
|  | What I see is that when you make a traditional pasture, in general, you plant exotic species of clover and grasses. However, buffer zones have permanent pastures that are more resilient.  Interview 13 English: 17 - 17 (0) |
| Opportunities preferred ecosystem services > Producers > Environmental impact of economic incentives | Well, if it is decided that an incentive should be given so that these areas are really implemented, it is possible that we have to make a contribution of monetary resources, let's say from somewhere else, in order for this to be carried out by way of subsidy, by way of incentive. This would be a disadvantage from the point of allocating economic resources. On the other hand, for this to be implemented and with the benefit of having better water quality.  Interview 4 English: 62 - 62 (0) |
| Opportunities preferred ecosystem services > Producers > Increasing awareness environmental problems | But, well, fortunately in Uruguay, it seems to me that over the last two or three years, the issues of agroecology and organic production are beginning to gain more awareness among producers, consumers, and decision-makers. They are starting to take on some elements that help to design public policies for the management of productive and natural resources, with a focus on taking care of natural resources. However, we are still very new to this in Uruguay and in the region, I would say that Uruguay is a leader in managing its resources.  Interview 11 English: 25 - 25 (0) |
| Opportunities preferred ecosystem services > Producers > Intermittent grazing | It builds up, like a clump of good, valuable trees, surrounded by invasive plants, and there are areas that cattle can no longer access. Cattle clean up the bush a lot, but they have to be able to access it. In order for this to be well-maintained, there has to be a well-managed human intervention.  Interview 4 English: 31 - 31 (0) |
|  | During a time when the natural pasture gives you less forage or you can't use it, you can use the buffer area.  Interview 13 English: 17 - 17 (0)  Then I have a part that is actually called low fields, which are floodable fields when there are large floods. There, I maintain the native vegetation, but I have added, for example, lotus, which is a leguminous plant that improves the nutritional quality of the native mix. And then that natural field, when well-managed, the meadows give me a lot of forage in spring, but when I get to late spring, I make good use of that area, which is a barren area, but at the same time, I have a good response in terms of forage production. The cattle take advantage of it. These are productive benefits.  Interview 13 English: 17 - 17 (0)  In reality, what it does is produce sustainable benefits because the permanent grassland is conserved, and all you do is collect the growth of forage to encourage regrowth because the forage plants have a growth rate that is called a certain rate. So, after reaching a certain level, if you use it, it's good because you take advantage of that fodder. But the plant returns with more vigour and captures more carbon, and in short, more is produced.  Interview 13 English: 17 - 17 (0)  So, when you have a livestock farming establishment, for example, a dairy farm. And you farm in order to have good pastures, you rotate the corn and then go back to the pasture. So, there is an agricultural phase of a year where you suddenly have two crops that are called "verdejos"; oats, or corn, and then you move on to the pasture. Oats or corn, and then you go through a phase of 4 years, and 5 years of permanent grassland. So, this is agricultural and livestock integration. If you have agricultural and livestock integration, you can at some point use the buffer areas for livestock farming in a sustainable and well-managed way.  Interview 13 English: 29 - 29 (0)  It is fertility that you return, and I think that what they should look at is what facilities, or how they give the producer facilities to make these investments because they are very expensive investments.  Interview 13 English: 32 - 32 (0) |
|  | Well, one option could be cattle farming for part of the year, but as I said today, that would only be possible for a few months of the year and that's impractical.  Interview 16 English: 43 - 43 (0) |
| Opportunities preferred ecosystem services > Producers > Opportunities for dialogue between stakeholders | Perhaps, there is a place where they (the government) could inform you about the native forests, and suggest a combination of shrubs and trees.  Interview 4 English: 54 - 54 (0) |
|  | Instead of immediately issuing sanctions, the ministry can explain to the offenders what they are doing wrong and encourage them to make amends.  Interview 13 English: 74 - 74 (0) |
|  | In my case I thank you for listening to us, for talking about it, because it's a good idea that at least I listen to the State, but that the State also listens to the small producer, more than anything else, who has limitations in the field, you know.  Interview 14 English: 86 - 86 (0) |
| Opportunities preferred ecosystem services > Producers > Pasture-conversion to combat exotic species | If you were to clean that area and do livestock farming, for example, it could be useful instead of just being a pasture.  Interview 14 English: 90 - 90 (0) |
| Opportunities preferred ecosystem services > Producers > Provision water resources for agriculture | On the one hand it can be a problem, on the other not so much. It ceases to be a problem and is a blessing, especially this year. Because of the blessing of this year is that in the drought, the buffer provides me more water. I mean, I am more than saved, I made a new reservoir, and what I could save, I saved, thanks to that plus. So, when you don't have water, you have it, surely.  Interview 16 English: 27 - 27 (0)  It's there in part because of the physical characteristics of the watercourse, and so on. From my point of view, there is nothing to be done in that sense. And I tell you, it's the water source that saves us fewer rich people.  Interview 16 English: 47 - 47 (0) |
| Opportunities preferred ecosystem services > Producers > Research and education | I think that in order to support all these ecosystem services, educating people could play an important role. For example, in the improvement of lowlands is important, where the natural vegetation is conserved, and that can be improved from a productive point of view while conserving biodiversity and avoiding over-exploitation of the resource.  Interview 13 English: 29 - 29 (0) |
|  | For me it would be necessary to study more, study better, understand better and see what you can do, what measures can be implemented to keep that area healthy. The issue of canalisations and everything else, you are also touching a productive system that works well  Interview 16 English: 70 - 70 (0) |
| Opportunities preferred ecosystem services > Producers > Self-financing buffer management | The only way to make that profit is to be able to sell some firewood. So, I make an arrangement with the people here. There are a lot of people who do that. And if you do, you keep the forest clean for me. You help me out here, and you cut a certain amount of firewood and take it with you. And with that, you do your work. But the ministry is currently refusing to do that. In other words, it would be like economic support for the management. And for me, just having that enabled, helps. It's allowing an activity for them to earn something and for me to control the buffers? Enabling an activity within the same ecosystem as self-financing. I’m waiting for the ministry of agriculture to support these management strategies. That's not good. Because do you know what the rule is? It's that people who already have a permit are still taking firewood. And they don't give out our new permits. They don't have them. We were saying that a more accurate control with technology would be something else that is needed. And I think it would be something that would make it feasible for you to allow new permits. To take the firewood out, to sell it legally, that the person who comes to work also is working in a place that is regulated. In other words, I think it's a win-win situation. Everybody wins. They should permit integrated management and that can be taken out.  Interview 4 English: 55 - 55 (0)  I think they are almost on the same level, I mean, in these situations where you are very close to the water intake of the main roads in the country, in a river that is very demanding. It is natural and logical that it should be controlled. It seems to me that today, maybe 20 years ago, it would have been unthinkable, but today you have technology that would allow you to do it in a reasonable way for everyone. That it is controlled. For instance, I'm going to remove the invasive ones. I'm going to remove some deadwood. It seems to me that with these things you can have proper control nowadays and allow the producer to maintain the buffer zone and areas surrounding the buffer zone, and have integrated management while the area is being cared for. That it is protected from invasive species. It seems to me that... what you have to do is incorporate a control technology. What needs to happen is that in order to reach that stage, today at least here in this situation as we are in it, it requires a lot of work. It requires a lot of work, and someone has to pay for it. Maybe some support would be needed at the beginning of the proper implementation when starting.  Interview 4 English: 57 - 57 (0) |
| Opportunities preferred ecosystem services > Producers > Technological possibilities for compliance control | Control is also crucial, as there are technological tools available to ensure compliance with guidelines and measures. However, control in Uruguay is currently lacking and needs to be improved. Overall, it is possible to overcome these obstacles by paying more attention to specific situations, applying resources effectively, and implementing better control measures.  Interview 4 English: 64 - 64 (0) |
| Opportunities preferred ecosystem services > Scientists > Advantages improved water quality | Not only farmers, but also other people from the cities, for fishing and swimming, and also the biodiversity in these areas will be very important.  Interview 7 English: 19 - 19 (0) |
| Opportunities preferred ecosystem services > Scientists > Bee keeping | You have to understand that not everyone can have hives. So, I tell you, you have to be careful when people take that measure, especially the people I know that I do not know what they live on, because the truth is that they have a very conservationist look; it has not been anything else but very conservationist. If it's okay, deep down I always say that if there hadn't been some pressure on the bin, the bin would be a disaster. But thanks to those people, much more restrictive measures were taken. They have an important function in society, but we cannot give them a say in everything because obviously their scientific knowledge and their approach are more spiritual than rational.  Interview 23 English: 33 - 33 (0)  I told you more in regard to the protected area than the buffer zones because these are low and I do not know if the hives have problems. But the truth is that they can be suitable areas for hives because you do not apply anything to the visit. In that respect, it is good. Even there it has an advantage; the native forest has good flowers for the hive, and that's why it defends itself. I think you can also make native forests, but below them they put a good permanent pasture base. I'm not saying don't do it, but first make me the pasture, and then put the little trees that grow slowly.  Interview 23 English: 35 - 35 (0) |
| Opportunities preferred ecosystem services > Scientists > Collaboration with producers | in my opinion, you need to prioritize ecological ecosystem services in that type of exchange, which leads you to say, how do we do it. Because then of course, for example, you can, it's relatively easy to have an oversight of agricultural activities by remote sensing, so you can see if there's tillage or, you know, there are planting crops in the land and the ministry has been doing that. But it's very difficult to know if these are natural grasslands or, and then people are putting cows or not, like the livestock issue is very difficult to enforce. And that's something that you need to work on with the people, like the agricultural issue, I think you can solve it.  Interview 22 English: 25 - 25 (0)  , I think there's already an acceptance of that you have to leave at least about buffer zones alone, slowly, especially in some watersheds like Santa Lucia or even Laguna del Cisne, so that the main drinking watersheds are not disturbed. I think there's already some acceptance by actors, different types of actors too, because at different places you have different actors  Interview 22 English: 29 - 29 (0) |
| Opportunities preferred ecosystem services > Scientists > Combating exotic species | So, they've had success with having goats to control regeneration, and they're able to chew the seeds, which prevents the germination that happens when you have cattle and cattle swallow the seeds, and then they all germinate in the cow pies. So, I know that they've had some reasonable success with managing with goats. There are a few examples of using girdling and then treating (these exotic species, red.) with an herbicide.  Interview 8 English: 47 - 47 (0) |
| Opportunities preferred ecosystem services > Scientists > Education of producers | And I think that the only way to advance slowly is through education.  Interview 7 English: 15 - 15 (0) |
|  | the solutions are to have long-term policies, so that Paso Severino is not, for example, an isolated issue, but rather that you have a long-term policy with a ministry and that you work in an articulated way with the other institutions, with the ministry, with the other institutions, with the Ministry of Livestock, with the municipalities, with the corresponding strategies, and that you hire the personnel that are doing this work with the producers. And that there is a follow-up of the buffer zones. And in addition, the other strategy for me is always information. Many producers always tell us, look, if you have to do so many things, tell me that what I am doing is helping someone. If you tell me water is getting better than I'll continue. Now, if I am doing all this and they are not really serving the buffer zones, or if I see that there are others that are not complying, why would I do it? So, I don't do it. So, information is extremely important, scientific information. So much money was invested, but only so many nutrients were removed, the quality of the water is the same, it is not golden, or it is slightly improving. So, they want information, and when there is information that says that things are working or that explains exactly how things are, people are willing to listen and they're willing to make improvements.  Interview 9 English: 25 - 25 (0) |
| Opportunities preferred ecosystem services > Scientists > Implementation process | Therefore, I think that there are different phases. You need first acceptance; people need to say okay, and agree. I think we are, in my opinion, we are already more or less there because people know that the water quality is poor and the problem is worsening. Then, you need some economic support because you need to build physical exclosures, but you also need to provide some solutions.  Interview 22 English: 31 - 31 (0)  what kind of people do you see controlling that is, are there extensionists from the Ministry of Agriculture, or are they part of the Ministry of Environment that are controlling?  Interviewee: In general, the Ministry of Agriculture is the Ministry of Agriculture for landowners and producers. You already have an institutional infrastructure in place. I mean there are places where people discuss their issues.  Interview 22 English: 32 - 33 (0) |
| Opportunities preferred ecosystem services > Scientists > Interaction government and producers | I think that what I've learned from living here is that it is interesting when either the government at some level, whether it's the Ministry of Environment or the Ministry of Agriculture. I mean, it is interesting when the government is able to provide examples or programs that support maybe innovative ways to treat exotic species. I have also seen subsidies where they are encouraging people, I think in Braveda, this would be Beatriz Sosa, but I think they have a project where people need fuel wood. I can't remember actually if it's government or not, but it's a project where people are incentivized to go cut down exotic species and they're paid somehow for that. But on the other hand, you have extensive areas where the government doesn't really have a huge presence. And so, in that sense, you really need both government and landholders that are working on this together.  Interview 8 English: 49 - 49 (0) |
| Opportunities preferred ecosystem services > Scientists > Local management of buffer zones | Around 2011, 2012, I'm not sure of the date, that process was initiated between the rural development agency and the sustainable development department of Canelones to try to move forward in that sense due to different problems they were having. Some were linked to water quality, others were linked to conflicts over the use of agrochemicals, others were linked to the development of agriculture itself. So, they began a process of dividing the department into different management zones, and in those management zones they began to analyse what activities were currently being developed and what activities should not be developed in that zone. So, well, in some areas there must be a high concentration of population and where family farming was also very important. What is being prevented is the advance of feedlot livestock farming, industrial agriculture, mainly linked to summer agriculture; soya and maize production. So, in the entire metropolitan area, which is more densely populated, new feedlot farms should be prohibited around the whole area around La Paz and Las Piedras. Of course, there were some difficulties with some buffer zones that were already planned to move forward in that sense, and that is what has brought many complications in the development of the plan.  Interview 18 English: 38 - 38 (0)  But beyond the implementation problems, the spirit in which it is created and the logic and some of the principles behind the development of the plan are very interesting, and it would be good if you could consider them. I'm not sure how much weight is given to the issue of buffer zones and riparian zones, but it is intended more as a more integrated approach to the watersheds and the development of agricultural and livestock farming in the watersheds.  Interview 18 English: 38 - 38 (0) |
|  | At some point, what you want is ecological integrity, and then you start looking at different reasons for having different ecosystems in different places. So, there's no single answer, and I think it would be wrong to say, okay, we are going to build the forest that filters more water alone. You also need to look at ecological integrity, which are the forests that kind of belong there and the surrounding vegetation, so you need to have some principles of restoration and also look at other dimensions of biodiversity, things like that.  Interview 22 English: 15 - 15 (0)  mostly I would say that you need to take into account surrounding expectations, compatibilities with uses in the surrounding areas, or at least social acceptance, to say it in a broader way.  Interview 22 English: 17 - 17 (0) |
| Opportunities preferred ecosystem services > Scientists > Potential for satellite monitoring | Monitoring noncompliance in the field of buffer zones with satellite monitoring is very easy to do nowadays.  Interview 3 English: 28 - 28 (0) |
| Opportunities preferred ecosystem services > Scientists > Potential influence of soil bacteria | And it's just been fascinating, and they have courses on the health of soil. And really, it's opened my eyes because I never worked with soil bacteria or mycorrhizae or plants, plant and microbe relationships. And I've started working in that area with a student of mine that's looking at growth-promoting bacteria that are associated with native woody species. And it's just, it's amazing, the diversity of organisms, the roles that they play. And Adriana Montanez is a professor that has a series of class material and different tools online on this concept of healthy soils. And I think in the riparian zones that we tend to think that it's the plants that are doing all the work, and really the soil microbes that are associated with plant, native plants, really have a huge role that they're playing, and we don't know a lot about it.  Interview 8 English: 21 - 21 (0) |
| Opportunities preferred ecosystem services > Scientists > Provision increased climate resilience | Because you do hear people saying that, this is not the question, but another positive aspect to having riparian zones is when you have cooler temperatures and riparian cover of the soil and water, and the water table is higher. So, some neighbours will, I've heard people say, oh, well, my neighbours are benefiting from the fact that I have riparian zones because I have more water underground than my neighbours do, which, don't have forest cover, for example, in the riparian zone.  Interview 8 English: 37 - 37 (0) |
|  | I think that what I proposed in the beginning will give more resilience to the buffer zones and make them more efficient in the face of increased climate variability. Buffer zones should be as wide as possible to provide more surface area, as this will give more resilience and efficiency. If the area is managed well and nutrients are removed from the area, they will be moved outward when there are floods, and the biomass will not be available for the stream to pull into the flood water pulse. This will make it less of a threat to the water pulse.  Interview 9 English: 29 - 29 (0) |
| Opportunities preferred ecosystem services > Scientists > Increased nutrient retention > Fast-growing vegetative species | You have to fix the slope quickly from where you throw your sediment. And there, you can use grass or plant species that quickly cover the soil. So, nobody thinks of making a tree that grows very slowly. You have to make a plant that grows fast. I mean, you can take a risk. Moving the soil is already a risk, but it will be less of a risk than if you were to grow a tree like that. Look, I was there the other time when they were going to plant at Paso Severino.  Interview 23 English: 15 - 15 (0) |
| Opportunities preferred ecosystem services > Scientists > Increased nutrient retention > Ploughing | We then ploughed the soil and found that the losses disappeared, were zero, or less than those in the natural area. We also compared the differences between putting fertilizer on top or a few centimetres into the soil and found that the difference was huge too. We applied iron, ferric chloride, on the top of the soil, at a low rate, to see if the iron interacting with phosphorus on the first few centimetres of soil could reduce the losses. We were successful, and the results were clear.  Interview 7 English: 13 - 13 (0) |
| Opportunities preferred ecosystem services > Scientists > Willingness of producers to implement | but some farmers that are more conscious are starting to change these practices and follow these recommendations and other people's recommendations. But it's a very small group, so there is no impact on the sources.  Interview 7 English: 13 - 13 (0) |
|  | thinking about the other cultural or social or reiterative values that can be generated by the buffer zones, what you're going to generate is less resistance to the implementation of the buffer zones or more support for them. Greater social support for the implementation of the buffer zones will reduce the resistance that producers have. Producers have a lot of resistance due to the increased costs and complexity of farm management, and above all, what they like the least is someone coming to say what they have to do, which is what bothers them the most. After they understand and see that the cost is not too high and that there are other benefits, they become more receptive. But what they like the least is someone from outside telling them what they have to do. So, if you have more social support because the workers in the locality see in the buffer zone an incentive to determine things that enrich or improve, you will have a counterbalance to reduce the resistance of the producers. Sometimes, producers are unaware of certain things. It happened to me, for example, while working at Paso Severino, that people would say, 'Ah, down there we used to camp with my grandfather, go fishing, and bathe in that place, and that's the place where very rough farming is now.'  Interview 9 English: 19 - 19 (0) |
|  | I would divide it into two, maybe two large blocks, at least those that are aligned with the logic of agricultural development and the frustration of the basin and those that aim to improve the environmental quality of the basin for different purposes, or because they live there, or because they are supplied with drinking water from the basin, and so on. So, for the former, the benefits would be that the development of the buffer zones would continue in a typical way as at present.  Interview 18 English: 28 - 28 (0)  And for the latter, the benefits would be a strict consideration of buffer zones and a control and monitoring of the evolution of these buffer zones. And again, surpassing the initial proposal of the Ministry of Environment of several years ago and moving on to more profound issues, let's say, of not only considering the buffer zones based on certain distances, but also considering the defined distances from the Paso Severino reservoir, but from the main bodies of water that later drain towards these reservoirs and towards the Santa Lucía River.  Interview 18 English: 28 - 28 (0)  On the other hand, these types of measures, as I mentioned, which are concentrated in the final phase of the process, can also be seen by these actors as an opportunity in the sense that they would not prohibit them from continuing to develop their activities. Well, if one scenario is not to be able to do it, other scenarios are to do it as I am, and another scenario is to allow these conservation measures to move forward without jeopardising the development of the activity itself. So, although it could be an obstacle, at some point it could also be understood as an opportunity for these actors to be able to continue with these activities without questioning the development of the activity itself.  Interview 18 English: 32 - 32 (0) |
|  | , I think there's already an acceptance of that you have to leave at least about buffer zones alone, slowly, especially in some watersheds like Santa Lucia or even Laguna del Cisne, so that the main drinking watersheds are not disturbed. I think there's already some acceptance by actors, different types of actors too, because at different places you have different actors  Interview 22 English: 29 - 29 (0) |
| Opportunities preferred ecosystem services > Vida Silvestre > Provision guidelines protected areas for buffer zones | So, the idea of having a protected area involves two things many things. First, it gives you some specific rules that apply to that piece of territory. You have some regulations that are different from how you have to take decisions and what you put forward in the rest of the country because protected areas are areas that have a specific set of considerations and laws that enable you to do things that elsewhere are much more difficult. That's one issue.  Interview 20 English: 31 - 31 (0)  I mean beyond buffer zones and Santa Lucia, for me it's the challenge of properly managing and regulating the country's landscape, bearing in mind that we live in a production-focussed country. So, we need to find a way to integrate those. Well in that sense it's not a different example from the Netherlands. We need to actively manage the whole landscape and it's simply we promote different goals in different specific sites to be able to maintain a multifunctional landscape that provides resilience, both for the social system and also for the ecological infrastructure that is needed to maintain that social system. And from that perspective for us, protected areas are a tool that enables us in certain parts of the country to do certain things that are needed to properly articulate the needs of the population there and the needs of the ecological systems to keep on functioning properly, and that's what for us are protected areas. And if they work and if we have good examples to tell on how we manage the biophysical system but also how we should work the discussions and the dreaming in the social system, then we can explore that idea with the rest of the country and actually manage the country in general from that point of view with this approach. So somehow institutions of sustainable management of the landscape are just in the protected areas, you have certain regulations and certain laws that enable you to do that and then you cannot do it outside right now. And also, you can put money and effort to make those examples, those specific sites, to work, and then as they work you can show; okay we can do the same elsewhere. That's the way we see it always, and within that, the buffer zone is a specific issue.  Interview 20 English: 35 - 35 (0) |
