# Supplementary material for: Understanding Stakeholder Perspectives on the Implementation and Management of Riparian Buffer Zones in the Santa Lucía River Basin, Uruguay
Source: Environ Manage. 2025 Jul 26;75(10):2596–613. doi: 10.1007/s00267-025-02230-1 (PMC12457512; doi:10.1007/s00267-025-02230-1)
Supplement: Supplementary file 3 — Supploementary material - interview guides [file 267_2025_2230_MOESM3_ESM.pdf]

Annex 4 Interview guides

4.1 Interview guide organisations

Using the decomposition as a starting point, the following interview guide is created. Although not intended to be utilized in a systematic manner during the interview, this interview guide will help steer the discourse. It is intended to direct the discussion on the interview's content and to suggest potential subjects, inquiries, and follow-up questions.

Introduction

Introduce yourself. Mention the purpose of the interview. Make clear that participation in this interview is voluntary and the decision to participate (or not) the interviewee. The interview should approximately last for one hour depending on how much information the interviewee would like to share. Mention that all responses will be kept confidential, and the interviewee will remain anonymous in the analysis and data management of this research. This means that the interview responses will only be shared with the research team members and the supervisors. Furthermore, the interviewer will ensure that any information included does not identify you as the respondent. Ask permission to record, and ask if the interviewee is willing to wear a microphone to improve the recording quality. Mention that the interviewee is free to refuse any question and is allowed to leave at all times. Explain the contents of the interview.

Questions

1. **Introduction questions:**
   a) What do you do for a living?
   b) What organization do you represent? (optional when talking to representatives).

The Santa Lucia River Action Plan introduced the use of riparian buffers in the Santa Lucia River Basin. The next few questions are about your (organisation’s) understanding, and your (organisation’s) role in the design and implementation of riparian buffers. (If needed, a small explanation about riparian buffers can be given here).

2. **What stakeholders are involved in the design and implementation of riparian buffer zones in the SLRB, and what is their interest and influence in these zones?**
   a) What are your/your organisation’s responsibilities and objectives in the design and management of riparian buffer zones?
      i) **Prompt:** What are your interests?
      ii) **Prompt:** What is your influence?
   b) What relationships do you/ your organisation have to other stakeholders in this process?
   c) How do your activities and responsibilities affect the process of designing/managing the riparian buffers?
      i) **Prompt:** How are other stakeholders affected by your activities?

These riparian buffers can be designed in many different ways, for instance by using different types of land cover and management strategies. There are thus different ways to characterise these riparian buffers. The next few pictures show some of the possible characteristics[1]. The next few questions are about how you would characterise riparian buffers. You can use the pictures if you wish, or you can mention/discuss other characteristics that come to your mind.

3. **What current attributes characterize riparian buffer zones in the SLRB from the perspective of stakeholders?**
   a) What functions do you think that riparian buffers currently have in the Santa Lucia River Basin?
      i) **Prompt:** Do they currently serve any (other) ecological, social or economic functions?
   b) What characteristics support these functions?
      i) **Prompt:** What aspects of the current riparian buffers do you think support these functions?
         (1) **Nudge:** Think of dimensions, land cover or land use.

4. **What are the preferred attributes of ideal riparian buffer zones in the SLRB from the perspective of stakeholders?**
   a) What functions do you think that riparian buffers should have in the Santa Lucia River Basin?
      i) **Prompt:** Can you rank these functions according to what you find most important?
   b) What characteristics do you think need to change, or be added, to (the design of) riparian buffers to support this change of function? (For example, presence or absence of livestock, implantation of new species, ecosystem management, etc.)
      i) **Prompt:** Can you score these characteristics according to what you find most important on a scale from 1 to 4 (1 = unimportant, 4 = most important)?

The next few questions are about the implementation of these different characteristics.

5. **Which barriers and opportunities to the implementation of preferred attributes in riparian buffer zones in the SLRB do stakeholders identify?**
   a) What do you think would be the benefits of these changes/additions to riparian buffers?
   b) What obstacles do you think will be experienced with the implementation of these changes/additions to riparian buffers?
      i) In regards to regulation, management, or any other process that you think is relevant for the implementation of these buffers…
      ii) **Prompt:** What downsides do you foresee for the implementation of these riparian buffers?
         (1) Are there any other difficulties you foresee?
         (2) Do you foresee any harm from the implementation of these riparian buffers?
   c) How do you think that these obstacles can be overcome?
      i) **Prompt:** What potential solutions do you think can be developed for this?

Optional when there is time left (more elaborated in 2nd round)

6. **How does climate change influence the preferred attributes of riparian buffer zones in the SLRB from the perspective of stakeholders?**
   a) How do you experience climate change in the Santa Lucia River Basin?
      i) **Prompt:** Think of changes in temperature, precipitation, droughts, or floods that you noticed over the years.
   b) How do you think these changes will affect the performance of riparian buffer zones in the Santa Lucia River Basin?
   c) Do you think that the effects of climate change on buffer zones would be different if the changes you proposed in the previous question are implemented?
   d) What do you think the effect of invasive animal or plant species is on the functioning of the buffer zones?
7. As a last question, do you have any other contacts of agricultural producers that might be relevant for this research?

Conclusion

Mention that the interview has ended. Ask the interviewee if they have any questions. Mention that the recording has ended. Thank the interviewee for their time.

4.2 Spanish translation interview guide organisations

Usando la descomposición como punto de partida, se crea la siguiente guía de entrevista. Aunque no pretende ser utilizada de manera sistemática durante la entrevista, esta guía de entrevista ayudará a orientar el discurso. Su objetivo es dirigir la discusión sobre el contenido de la entrevista y sugerir posibles temas, consultas y preguntas de seguimiento.

Introducción

Hola,

Me llamo Alfred,

Soy estudiante en la Universidad de Wageningen, y actualmente estoy haciendo mi tesis de máster sobre las percepciones de las partes interesadas a las zonas buffer en la cuenca del río Santa Lucía. Mi español no es fluido, así que discúlpenme por ello.

Más concretamente, estoy investigando la voluntad y la implicación de los distintos grupos de interesados, como agricultores e instituciones governalmente, a la hora de implantar zonas buffers, y cómo pueden mejorarse.

Por ello, he pensado que sería útil conocer su opinión al respecto, ya que los productores y las uniones de productores, como la CAF, desempeñan un papel importante en esta cuestión.Lo voy a entrevistar sobre sus experiencias con las zonas buffers. La entrevista debe durar una hora. Sus respuestas se documentarán de forma anónima. ¿Está bien que grabe esta entrevista?

When yes;

Las zonas de amortiguamiento ribereñas son franjas de vegetación a lo largo de los cursos de agua. Su propósito es captar y retener los nutrientes de las actividades agrícolas, para mejorar la calidad del agua. Esta entrevista tratará sobre las zonas buffer en la cuenca del río Santa Lucía. Y se centrará en el papel de su organización y en su percepción personal de la implantación de estas zonas buffers.

Preguntas

1. **Preguntas de introducción:**
   a) ¿A qué te dedicas?
   b) ¿Qué organización representa? (opcional al hablar con representantes).
   c) ¿cómo se involucró en el comité de cuenca del río santa lucia?

El Plan de Acción del Río Santa Lucía introdujo el uso de zonas de amortiguamiento ribereñas en la cuenca del río Santa Lucía. Las siguientes preguntas son sobre su comprensión (la de la organización) y el papel de su (la organización) en el diseño y la implementación de las zonas de amortiguamiento ribereñas. (Si es necesario, aquí se puede dar una pequeña explicación sobre las zonas de amortiguamiento ribereñas).

2. **¿Qué actores están involucrados en el diseño e implementación de zonas de amortiguamiento ribereñas en la SLRB? ?y cuál es su interés e influencia en estas zonas?**
   a) ¿Cuáles son sus responsabilidades y objetivos o los de su organización en el diseño y la gestión de las zonas de amortiguamiento ribereñas?
      i) **Alternativa:**¿Cuáles son sus intereses?

ii) **Alternativa:**¿Cuál es su influencia?

b) ¿Qué relaciones tiene usted o su organización con otras partes interesadas en este proceso?

c) ¿Cómo afectan sus actividades y responsabilidades al proceso de diseño/gestión de las zonas de amortiguamiento ribereñas?

i) **Alternativa:** ¿Cómo se ven afectadas otras partes interesadas por sus actividades?

Estas zonas de amortiguamiento ribereñas se pueden diseñar de muchas maneras diferentes, por ejemplo, utilizando o promoviendo diferentes tipos de cobertura del suelo y estrategias de gestión. Por lo tanto, existen diferentes formas de definir estas zonas de amortiguamiento ribereñas. Las siguientes preguntas son sobre cómo definiríalas zonas de amortiguamiento ribereñas.

3. **¿Qué atributos o cualidades actuales caracterizan las zonas de amortiguamiento ribereñas en la SLRB desde la perspectiva de las partes interesadas?**

a) ¿Qué funciones cree que tienen actualmente las zonas de amortiguamiento ribereño en la cuenca del río Santa Lucía?

i) **Alternativa:**¿Cumplen actualmente alguna (otra) función ecológica, social o económica?

b) ¿Qué características sustentan estas funciones?

i) **Alternativa:**¿Qué aspectos de las zonas de amortiguamiento ribereñas actuales cree que respaldan estas funciones?

(1) **Empujar:** Piense en las dimensiones, la cobertura del suelo o el uso del suelo.

c) ¿De qué manera cree que las zonas buffers pueden ser beneficiosas para los productores que forman parte de su organización?

d) ¿Qué funciones cree que tienen actualmente las zonas de amortiguación ribereñas de la cuenca del río Santa Lucía? ¿Forman actualmente las zonas buffers un problema para los productores de su organización? En caso afirmativo, ¿en qué sentido?

4. **¿Cuáles son los atributos preferidos de las zonas de amortiguamiento ribereñas ideales en la SLRB desde la perspectiva de las partes interesadas?**

a) Si las zonas buffers pudieran mejorarse, ¿qué funciones cambiaría o añadiría a las zonas buffers? ¿Qué funciones cree que deberían tener los amortiguadores ribereños en la cuenca del río Santa Lucía?

i) **Alternativa:** ¿Puede clasificar estas funciones de acuerdo con lo que le parezca más importante?

b) ¿Qué características cree que deben cambiarse o agregarse al (diseño de) las zonas buffers para respaldar este cambio de función? (Por ejemplo, presencia o no de ganado, implantación de nuevas especies, manejo del ecosistema, etc.)

i) **Alternativa:** ¿Puede calificar/valuar estas características de acuerdo con lo que considere más importante en una escala del 1 al 4 (1 = sin importancia, 4 = más importante)?

Las siguientes preguntas son sobre la implementación de estas diferentes características.

5. **¿Qué barreras y oportunidades para la implementación de los atributos preferidos en las zonas de amortiguamiento ribereñas en la SLRB identifican las partes interesadas?**

a) ¿Cuáles cree que serían los beneficios de estos cambios/adiciones a las zonas de amortiguamiento ribereñas?

b) ¿Qué obstáculos cree que se experimentarán con la implementación de estos cambios/adiciones a las zonas de amortiguamiento ribereñas?

i) En cuanto a la regulación, gestión, o cualquier otro proceso que creas relevante para la implementación de estos buffers…

ii) **Alternativa:**¿Qué inconvenientes prevé para la implementación de estas zonas de amortiguamiento ribereñas?
    (1) ¿Hay otras dificultades que prevé?
    (2) ¿Prevé algún daño o perjuicio por la implementación de estas zonas de amortiguamiento ribereñas?

c) ¿Cómo crees que se pueden superar estos obstáculos?
    i) **Alternativa:**¿Qué posibles soluciones cree que se pueden desarrollar para esto?

Opcional cuando quede tiempo (más elaborado en 2ª vuelta)

Las siguientes preguntas se refieren a su interpretación de las repercusiones del cambio climático y sus efectos.

6. **¿Cómo influye el cambio climático en los atributos preferidos de las zonas de amortiguamiento ribereñas en la SLRB desde la perspectiva de las partes interesadas?**
    a) ¿Cómo se vive el cambio climático en la cuenca del río Santa Lucía?
        i) **Alternativa:** Piense en los cambios de temperatura, precipitaciones, sequías o inundaciones que haya notado a lo largo de los años.
    b) ¿Cómo cree que estos cambios afectarán el desempeño de las zonas de amortiguamiento ribereñas en la cuenca del río Santa Lucía?
    c) ¿Cree que los efectos del cambio climático sobre zonas de amortiguamiento serían distintos si los cambios que propuso en la pregunta anterior se implementan?
7. ¿Tiene alguna experiencia con especies animales o vegetales invasoras en su predio?
    a) En caso afirmativo, ¿Como affectan su trabajo?
    b) Como piensa que las zonas buffer afectan a estas especies?

8. No tengo más preguntas. ¿Tiene alguna pregunta? Muchas gracias por su tiempo. Termino la grabación.

Conclusión

Mencione que la entrevista ha terminado. Pregúntele al entrevistado si tiene alguna pregunta. Menciona que la grabación ha terminado. Agradece al entrevistado por su tiempo.

4.3 Interview guide producers

Introduction

Introduce yourself. Mention the purpose of the interview. Make clear that participation in this interview is voluntary and the decision to participate (or not) the interviewee. The interview should approximately last for one hour depending on how much information the interviewee would like to share. Mention that all responses will be kept confidential, and the interviewee will remain anonymous in the analysis and data management of this research. This means that the interview responses will only be shared with the research team members and the supervisors. Furthermore, the interviewer will ensure that any information included does not identify you as the respondent. Ask permission to record, and ask if the interviewee is willing to wear a microphone to improve the recording quality. Mention that the interviewee is free to refuse any question and is allowed to leave at all times. Explain the contents of the interview. Explain that the concept of riparian buffers in the SLRB will be the main focus of the interview. If necessary, explain the concept of riparian buffers to the interviewee.

Questions

1. **Introduction questions:**
    a. What do you do for a living?
    b. What organization do you represent? (optional when talking to representatives).

The Santa Lucia River Action Plan introduced the use of riparian buffers in the Santa Lucia River Basin. The next few questions are about your (organisation's) understanding, and your (organisation's) role in the design and implementation of riparian buffers. (If needed, a small explanation about riparian buffers can be given here).

    c. What is your role in the management of riparian buffer zones?
        i. Alternatively, what tasks do you have regarding the management of riparian buffers?
    d. With whom do you work together in this process?

These riparian buffers can be designed in many different ways, for instance by using different types of land cover and management strategies. There are thus different ways to characterise these riparian buffers. The next few pictures show some of the possible characteristics. The next few questions are about how you would characterise riparian buffers. You can use the pictures if you wish, or you can mention/discuss other characteristics that come to your mind.

2. **What current attributes characterize riparian buffer zones in the SLRB from the perspective of stakeholders?**
    a. What purposes do you think that riparian buffers currently have in the Santa Lucia River Basin to your organisation, you personally or your business?
        i. Does it cause any pressures for you or your business?
        ii. **Prompt:** Do riparian buffers currently serve any (other) ecological, economic, or other purposes to you?
    b. What characteristics of the buffers do you associate to the current riparian buffers in that support those purposes?
        i. **Prompt:** What aspects of the current riparian buffers do you think provide these functions to you?
            1. **Nudge:** Think of dimensions, land cover or allowed land use.
    c. What purposes do you think that riparian buffers currently have in the Santa Lucia River Basin to society?

1. **Prompt:** Do riparian buffers currently serve any (other) ecological, social, economic, or other purposes?

d. What characteristics of the buffer do you associate to the current riparian that support those purposes?
   i. **Prompt:** What aspects of the current riparian buffers do you think provide these purposes to society?
      1. **Nudge:** Think of dimensions, land cover or allowed land use.

3. **What preferred attributes characterize riparian buffer zones in the SLRB from the perspective of stakeholders?**
   a. What purposes do you think that riparian buffers should have in the Santa Lucia River Basin to you personally or your business?
   b. What characteristics need to be changed or added, to (the design of) riparian buffers to support this?
   c. What purposes do you think that riparian buffers currently have in the Santa Lucia River Basin to society?
   d. What characteristics need to be changed or added, to (the design of) riparian buffers to support this change of purposes?
   e. Can you score these characteristics according to what you find most important from 1 to 4 (1 = unimportant, 4 = most important)?
4. **Which barriers and opportunities to the implementation of preferred attributes in riparian buffer zones in the SLRB do stakeholders identify?**
   a. What do you think would be the benefits of these changes/additions to riparian buffers to you personally?
      i. And to society?
   b. What downsides do you foresee for the implementation of these riparian buffers for you personally?
      i. In what way do you expect the implementation of these changes to make your work more difficult?
      ii. Are there any other difficulties you foresee?
         1. Do you foresee any harm from these riparian buffers to you personally?
   c. And what downsides do you foresee for the implementation of these changes to society?
   d. What obstacles do you think will be experienced with the implementation of these changes/additions to riparian buffers?
      i. In regard to regulation, management, or any other process that you think is relevant for the implementation of these buffers…
   e. How do you think that these obstacles can be overcome?
      i. **Prompt:** What potential solutions do you think can be developed for this?
5. **How does climate change influence the preferred attributes of riparian buffer zones in the SLRB from the perspective of stakeholders?**
   a. How do you experience climate change in the Santa Lucia River Basin?
      i. **Prompt:** Think of changes in temperature, precipitation, droughts, or floods that you noticed over the years.
   b. How do you think these changes will affect the buffer zones in any way?
   c. Do you think that the effects of climate change on buffer zones would be different if the changes you proposed in the previous question are implemented?

d. Do you have any experience with invasive animal or plant species in your farm?
    i. If so, how do they affect your work?
    ii. How do you think riparian buffers affect these species?

Conclusion

Mention that the interview has ended. Ask the interviewee if they have any questions. Mention that the recording has ended. Thank the interviewee for their time.

4.4 Spanish translation interview guide producers

Introducción

Presentarte. Mencione el propósito de la entrevista. Dejar claro que la participación en esta entrevista es voluntaria y decisión de participar (o no) del entrevistado. La entrevista debe durar aproximadamente una hora dependiendo de la cantidad de información que el entrevistado quiera compartir. Mencione que todas las respuestas se mantendrán confidenciales y que el entrevistado permanecerá en el anonimato en el análisis y manejo de datos de esta investigación. Esto significa que las respuestas de la entrevista solo se compartirán con los miembros del equipo de investigación y los supervisores. Además, el entrevistador se asegurará de que cualquier información incluida no lo identifique a usted como el encuestado. Pida permiso para grabar y pregunte si el entrevistado está dispuesto a usar un micrófono para mejorar la calidad de la grabación. Mencione que el entrevistado es libre de rechazar cualquier pregunta y puede irse en todo momento. Explique el contenido de la entrevista. Explique que el concepto de zonas buffer en SLRB será el enfoque principal de la entrevista. Si es necesario, explique el concepto de zonas buffer al entrevistado.

Preguntas

1. **Preguntas de introducción:**
    a. ¿A qué te dedicas?
    b. ¿Qué organización representa? (opcional al hablar con representantes).

El Plan de Acción del Río Santa Lucía introdujo el uso de zonas buffer en la cuenca del río Santa Lucía. Las siguientes preguntas son sobre su comprensión (la de la organización) y su role (de su organización) en el diseño y la implementación de las zonas buffer. (Si es necesario, aquí se puede dar una pequeña explicación sobre las zonas buffer).

    c. ¿Cuál es su papel en la gestión de las zonas buffer?
        i. ¿qué tareas tiene con respecto a la gestión de las zonas buffer?
    d. ¿Con quién trabajas en conjunto en este proceso?

Estas zonas buffer se pueden diseñar de muchas maneras diferentes, por ejemplo, utilizando diferentes tipos de cobertura del suelo y estrategias de gestión. Por lo tanto, existen diferentes formas de caracterizar estas zonas buffer. Las siguientes imágenes muestran algunas de las posibles características .Las siguientes preguntas son sobre cómo describeria de las zonas buffer. Puede usar las imágenes si lo desea, o puede mencionar/discutir otras características que le vengan a la mente.

2. **¿Qué atributos actuales caracterizan las zonas buffer en la SLRB desde la perspectiva de las partes interesadas?**
    a. ¿Qué propósitos cree que tienen actualmente las zonas buffers en la cuenca del río Santa Lucía (para su organización), usted personalmente o su empresa?
        i. Generan algun prejuicio para usted o su impressa?
            1. **Alternativa:**¿Las zonas buffer sirven actualmente a algún (otro) propósito ecológico, económico o de otro tipo para usted?

b. ¿Qué characteristicas de las zonas buffers asocia usted a las zonas buffer actuales en la cuenca del río Santa Lucía que respaldan esos propósitos?
    i. **Alternativa:**¿Qué aspectos de las zonas buffer actuales cree que le brindan estas propositos?
        1. **Empujar:**Piense en las dimensiones, la cobertura del suelo o el uso permitido del suelo.

c. ¿Qué propósitos cree que tienen actualmente las buffers en la cuenca del río Santa Lucía para la sociedad?
        1. **Alternativa:**¿Las zonas buffer sirven actualmente para algún (otro) propósito ecológico, social, económico o de otro tipo?

d. ¿Qué caracteristicas de la zona buffer asocia usted a las zonas buffer actuales en la cuenca del río Santa Lucía que respaldan esos propósitos?
    i. **Alternativa:**¿Qué aspectos de los actuales zonas buffers cree que brindan estos propósitos a la sociedad?
        1. **Empujar:**Piense en las dimensiones, la cobertura del suelo o el uso permitido del suelo.

3. **¿Qué atributos preferidos caracterizan las zonas buffer en la SLRB desde la perspectiva de las partes interesadas?**
    a. ¿Qué propósitos cree que deberían tener las zonas buffer para usted personalmente o para su empresa?
    b. ¿Qué caracteristicas deben cambiarse o agregarse en (el diseño de) las zonas buffer para respaldar este?
    c. ¿Qué propósitos cree que deberian tener las zonas buffers para la sociedad?
    d. ¿Qué caracteristicas deben cambiarse o agregarse en (el diseño de) las zonas buffer para respaldar este?
    e. ¿Puede calificar estas características de acuerdo con lo que considere más importante del 1 al 4 (1 = sin importancia, 4 = más importante)?

4. **¿Qué barreras y oportunidades para la implementación de los atributos preferidos en las zonas buffer en la SLRB identifican las partes interesadas?**
    a. ¿Cuáles cree que serían los beneficios de estos cambios a las zonas buffer para usted personalmente?
        i. ¿Y a la sociedad?
    b. ¿Qué inconvenientes prevé para usted con la implementación de estos cambios que menciona?
        i. ¿De qué manera espera que la implementación de estos cambios dificulte su trabajo?
        ii. ¿Hay otras dificultades que prevé?
            1. ¿Prevé algún daño de estos zonas buffers para usted?
    c. ¿Y qué inconvenientes prevé en la implementación de estos cambios para la sociedad?
    d. ¿Qué obstáculos cree que se experimentarán con la implementación de estos cambios a las zonas buffer?
        i. Por ejemplo para la regulación, gestión, o cualquier otro proceso que creas relevante para la implementación de estos buffers…
    e. ¿Cómo crees que se pueden superar estos obstáculos?
        i. **Alternativa:**¿Qué posibles soluciones cree que se pueden desarrollar para esto?

5. **¿Cómo influye el cambio climático en los atributos preferidos de las zonas buffer en la SLRB desde la perspectiva de las partes interesadas?**

a. ¿Cómo vive el cambio climático en la cuenca del río Santa Lucía?
    i. **Alternativa**: Piense en cambios de temperatura, precipitaciones, sequías o inundaciones que haya notado a lo largo de los años.

a. ¿Cómo cree que estos cambios afectarán a las zonas buffer?
b. ¿Cree que los efectos del cambio climático en las zonas buffer serían diferentes si los cambios que usted propone se implementar?
c. ¿Tiene alguna experiencia con especies animales o vegetales invasoras en su predio?
    a. En caso afirmativo, ¿Como affectan su trabajo?
    b. Como piensa que las zonas buffer afectan a estas especies?

Conclusión

Mencione que la entrevista ha terminado. Pregúntele al entrevistado si tiene alguna pregunta. Menciona que la grabación ha terminado. Agradece al entrevistado por su tiempo.

Annex 2 Example images riparian buffer zones

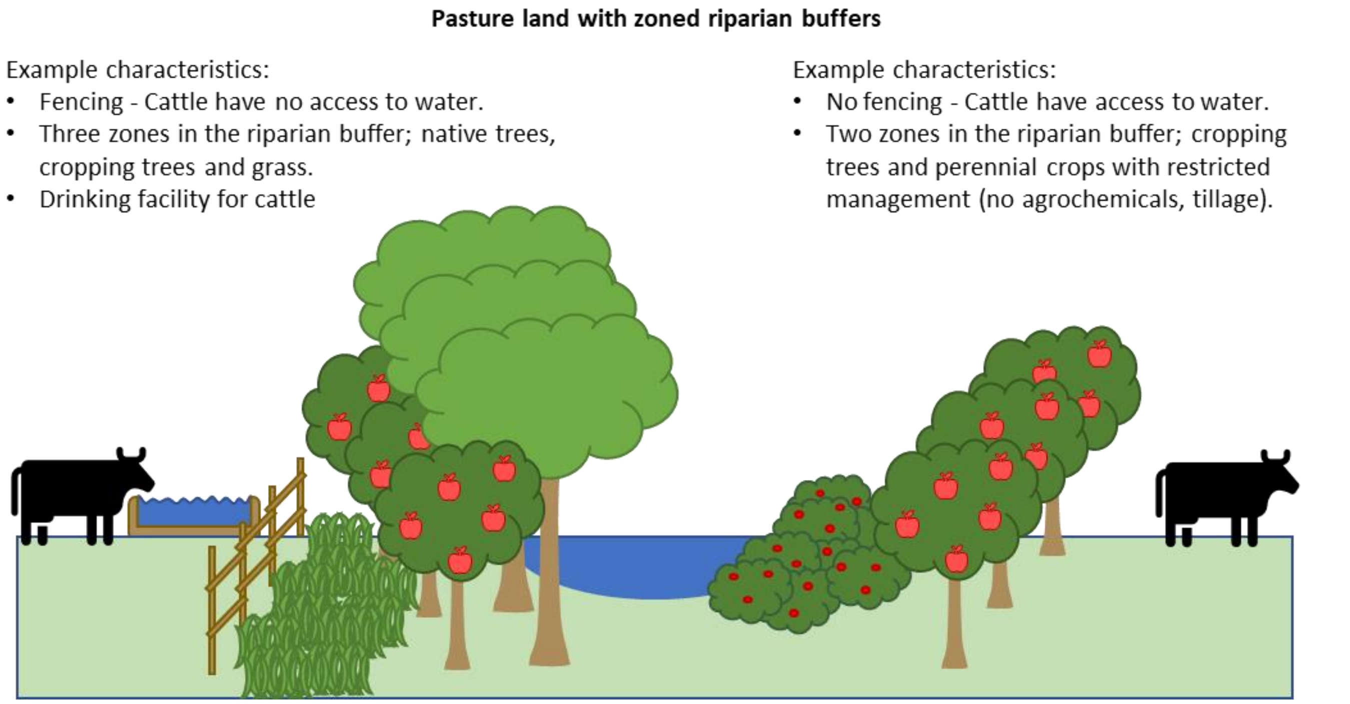

Figure 3.A. Example image riparian buffers in English.

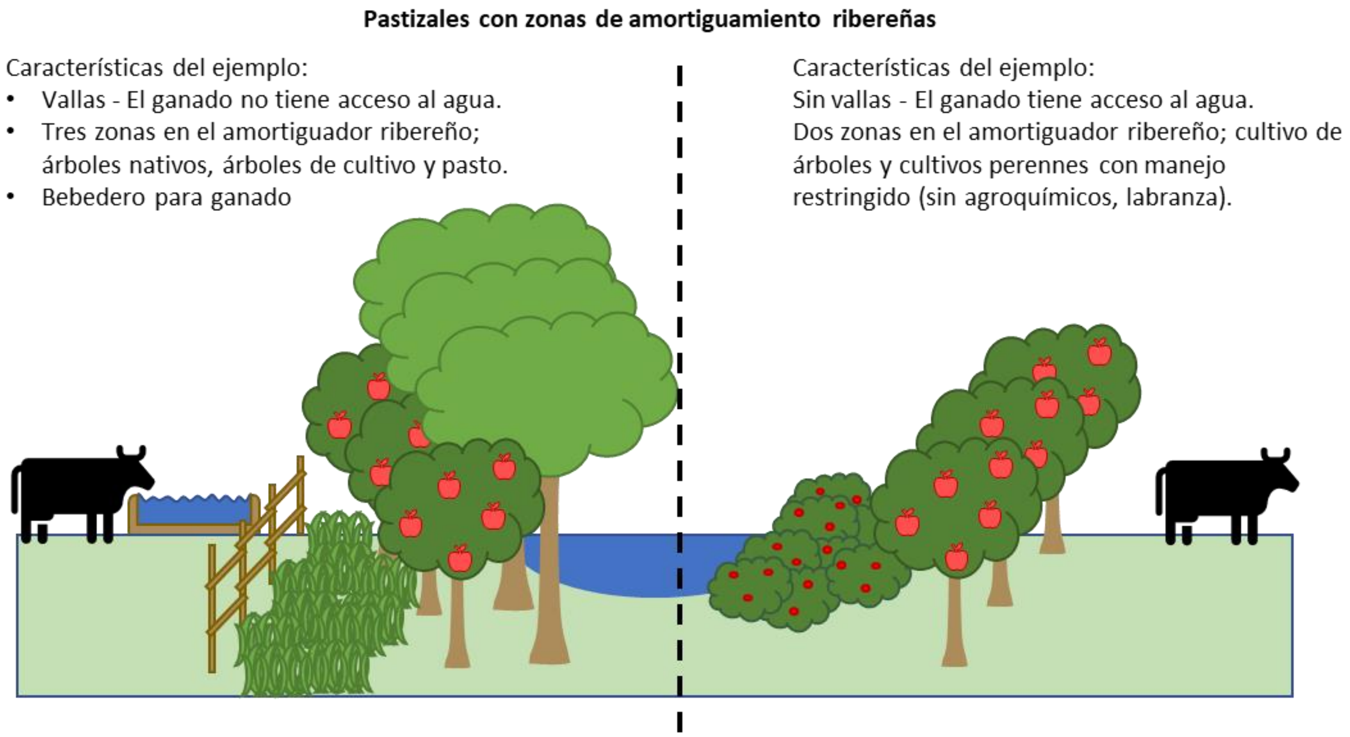

Figure 3.B. Example image riparian buffers in Spanish.
